# Proceedings of the 2023 International Maternal Newborn Health Conference

**DOI:** 10.1186/s12919-024-00289-y

**Published:** 2024-03-21

## F1.1. Networks of care for maternal and newborn health: an approach to strengthening relational elements for quality and respectful care

### Katherine Kalaris^1^, Allisyn Moran^2^

#### ^1^University of Oxford; ^2^World Health Organization

*BMC Proceedings 2024*, **18(5):**F1.1

Submission ID #: IMNHC96

Panel ID# (if applicable): DASKP1096

**Panel description**

Despite global progress in reducing preventable maternal and newborn deaths, high rates of maternal and newborn morbidity and mortality continue, mainly due to poor quality of care. Additional transformational and catalytic approaches are needed to provide high-quality respectful patient-centered care to further improve maternal and newborn well-being and survival. One documented successful approach is Networks of Care (NoC).

NoC are an innovative approach to optimize health system functioning, intentionally creating and strengthening health system relationships to support transformational change in maternal and newborn health. NoC emphasize relational elements, such as empowered multidisciplinary teams, respect, teamwork, trust, communication, collaboration, leadership, and supportive supervision and mentorship, which differentiate it from other health systems strengthening approaches. Functional NoC enable collaborative learning and coordinated continuity of respectful and quality care to optimize linkages for efficient and resilient health systems, and to ultimately improve maternal and newborn survival and well-being. Future opportunities include scaling up the NoC approach at national and sub-national levels and addressing gaps in the evidence base.

The NoC approach has been implemented in many contexts. This panel will provide an overview of the NoC approach, and highlight examples and results of NoC from three countries: Kenya (Makueni County), Nepal (One Heart Worldwide), and Zambia (integrated sexual, reproductive, maternal, and newborn health program).

## F1.2. Improving quality of maternal and newborn care through the creation of networks of safety in rural Nepal

### Surya Bhatta^1^, Katherine Kalaris^2^, Sibylle Kristensen^1^

#### ^1^One Heart Worldwide; ^2^University of Oxford

*BMC Proceedings 2024*, **18(5):**F1.2

Submission ID #: IMNHC204

Panel ID# (if applicable): DASKP1096

**Background**

Although improvements made in maternal and newborn health (MNH) have increased maternal and neonatal survival in Nepal, challenges remain for the country to reach Stage V of the obstetric transition. The international nongovernmental organization One Heart Worldwide (OHW) supports the Government of Nepal’s national plan to provide quality MNH services to pregnant women and their newborns living in rural areas. In collaboration with all levels of the health system, OHW forms Networks of Safety (NoS) to provide quality MNH care in some of Nepal’s most remote and difficult-to-access geographies. These NoS are examples of Networks of Care (NoC). All NoS exhibit core system-strengthening NoC functional elements, such as constructing new birthing centers and providing equipment, along with relational elements such as promoting local ownership, supporting mentorship, and connecting providers from the community level through to tertiary care.

**Methods**

NoS build local capacity through a participatory model and target infrastructure, local leadership, and local communities over a six-year period. Many network activities are co-financed by the municipalities leveraging a financially sustainable adoption-at-scale solution that addresses the systemic health care access disparities disproportionately affecting mothers and newborns in last-mile environments. NoS also spearhead innovative approaches such as a simulation-based mentorship program to maintain essential clinical skills among rural health care providers and an MNH helpline that supports these providers in clinical decision-making, particularly in emergencies.

**Results**

In 2022, OHW’s NoS have been expanded to 28 districts across Nepal, reaching a third of districts in the country. As of today, OHW is implementing NoS in 20 districts, covering a population of 6.6 million with 165,000 annual pregnancies, and has completed the program in eight districts. Since 2010, the NoS have improved quality MNH care for more than 414,000 pregnancies in rural Nepal.

**Conclusions**

OHW’s work shows how NoC are a sustainable approach to provide quality care for MNH in remote and rural areas through innovative strategies and solutions. We will continue to scale and adapt network interventions to ensure access and quality of MNH care and plan to reach 50% of districts by 2030.

## F1.3. Reduction of maternal and neonatal mortality within networks of care in Northern Province, Zambia

### Morrison Zulu^1^, Katherine Kalaris^2^, Andrew Storey^1^, Angel Mwiche^3^

#### ^1^Clinton Health Access Initiative; ^2^University of Oxford; ^3^Ministry of Health, Zambia

*BMC Proceedings 2024*, **18(5):**F1.3

Submission ID #: IMNHC203

Panel ID# (if applicable): DASKP1096

Note the full manuscript was published in 2022 in *PLOS Global Public Health*: https://www.ncbi.nlm.nih.gov/pmc/articles/PMC10021549/

## F2.1. Black Mamas Matter Alliance: advancing the black maternal health, rights, and justice movement

### Ayanna Robinson, Philicia Castillo, Angela Aina

#### Black Mamas Matter Alliance, Inc

*BMC Proceedings 2024*, **18(5):**F2.1

Submission ID #: IMNHC1642

Panel ID# (if applicable): DBXIN211642

**Panel Description**

The United States (U.S.) is experiencing a maternal health crisis. To meet Sustainable Development Goals, many countries are working to decrease their maternal mortality ratios (MMR), currently achieving a 38% global decrease between 2000 and 2017. However, the U.S. continues to report a significant MMR increase and racial inequities with Black women dying at nearly three times the rate of white women. Black birthing women are also twice as likely as white women to experience severe pregnancy complications.

The Black Mamas Matter Alliance (BMMA), founded in 2016, serves as a national voice and coordinating entity for stakeholders advancing Black maternal health, rights, and justice. We envision a world where Black Mamas have the rights, respect, and resources to thrive before, during, and after pregnancy. BMMA believes that community empowerment and mobilization are vital to its strategy to involve small-scale organizations, health workers, and everyday citizens interacting to increase education and engagement in preventive health promotion activities. Our approach utilizes the strengths of doulas, midwives, and other skilled maternal health actors working with the communities they serve.

This panel will provide an overview of BMMA; the work conducted within our four key pillars of policy, research, care, and culture shift; and the impact on maternal health in the U.S. Speakers will describe BMMA’s process for leveraging Black women’s lived experiences and scholarship to decolonize research, develop policy priorities, identify principles for holistic maternity care, and apply BMMA’s research principles across a variety of projects addressing Black maternal health.

## F2.2. Conducting research with, for, and by black birthing people and researchers: operationalizing black mamas matter alliance’s research principles

### Sang Hee Won, Philicia Castillo, Ayanna Robinson, Angela Aina

#### Black Mamas Matter Alliance, Inc

*BMC Proceedings 2024*, **18(5):**F2.2

Submission ID #: IMNHC1670

Panel ID# (if applicable): DBXIN211642

**Background**

Black women are three times more likely to die from pregnancy-related causes than white women in the United States (U.S.). Yet, research to understand the underlying causes of the inequities have historically marginalized and neglected Black birthing people. The Black Mamas Matter Alliance (BMMA), a national network of Black women-led organizations and professionals, developed best practices for conducting research with, for, and by Black Mamas to redefine how research can, and should, be done in the U.S. to improve Black maternal health outcomes.

**Methods**

Since 2017, BMMA has been applying its research principles to projects dedicated to addressing inequities in maternal health in the U.S. Two such projects include research examining the role of disrespectful maternity care among Black women in Atlanta, Georgia, and increasing community engagement efforts within state and local maternal mortality review processes. Key activities have included centering community needs, voices, and experts to lead the research; utilizing its alliance structure to uplift Black researchers; instituting regular feedback loops with communities participating in the research; and prioritizing communities in the dissemination of results.

**Results**

Applying these research principles has enabled BMMA to be responsive to community needs and embrace communities as equal partners in the conduct of research. Key results include elevating the voices of Black birthing people to share their experiences with disrespectful maternity care; empowering communities most affected to provide direct feedback to government entities and funders who have dictated how maternal mortality reviews should be done; and centering community-driven, innovative approaches to improving maternal health in the U.S.

**Conclusions**

BMMA’s research principles have led to a paradigm shift in how research is done with, for, and by Black Mamas. Application of the principles has transformed the ways in which research and programming around maternal health have traditionally been done in the U.S. by shifting decision-making power and ownership to communities that know best what they need. It has reframed the narrative of Black maternal health from that of deficit to asset-based thinking whereby the communities most affected have the expertise and solutions to fully address inequities in maternal health.

## F2.3. Often discussed, but never consulted: leveraging the expertise of the black perinatal workforce, scholars, and birthing people to develop principles and priorities addressing the maternal mortality crisis in the United States

### Philicia Castillo, Ayanna Robinson, Sang Hee Won, Angela Aina

#### Black Mamas Matter Alliance, Inc

*BMC Proceedings 2024*, **18(5):**F2.3

Submission ID #: IMNHC1656

Panel ID# (if applicable): DBXIN211642

**Background**

The United States (U.S.) is experiencing a maternal health crisis with maternal mortality ratios increasing over the last 20 years and disproportionately affecting Black women who are dying from largely preventable causes at three times the rate of white women. Addressing this crisis requires maternal health care, policy, and research that is led by the group most affected but often left out of solution creation, Black birthing people. Recognizing this equity, the Black Mamas Matter Alliance (BMMA) created working groups that use strategies grounded in Black birthing people’s lived experience, wisdom, and leadership to advance care, change policy, and cultivate research. These working groups created principles and priorities that the perinatal workforce, researchers, advocates, funders, and other stakeholders can use as guiding posts to combat maternal mortality and collectively achieve Black maternal health, rights, and justice.

**Methods**

Between 2017 and 2019, BMMA convened alliance partners who are leading experts in maternal and reproductive health and created three working groups: Holistic Care, Policy, and Research. The working groups consisted of Black perinatal professionals, research scholars, policy professionals, and advocates. The groups assessed the current landscape of maternal, sexual, and reproductive health care, policy, and research, and identified gaps.

**Results**

Principles were created for achieving holistic maternity care, engaging in equitable research with Black communities, and advancing policy priorities grounded in frameworks around reproductive justice and human rights to improve Black maternal health outcomes. Each working group published a report with historical background and framing of the current public health problem that is the maternal mortality crisis among Black birthing people with a set of principles and priorities for widespread use.

**Conclusions**

The holistic care and research principles and policy priorities created by BMMA take a multi-pronged approach to equipping the perinatal workforce, researchers, advocates, funders, and other stakeholders with the tools and guidelines to collectively support Black Mamas and reduce maternal mortality and morbidity. These principles and priorities are leading the way in a paradigm shift that highlights the expertise Black scholars and the perinatal workforce possess, while supporting and centering Black Mamas in care, policy, and research practices and decisions in the U.S. and globally.

## F2.4. Black Mamas Matter Alliance: advancing sexual, reproductive, and maternal health, rights, and justice

### Ayanna Robinson, Philicia Castillo, Sang Hee Won, Angela Aina

#### Black Mamas Matter Alliance, Inc

*BMC Proceedings 2024*, **18(5):**F2.4

Submission ID #: IMNHC1645

Panel ID# (if applicable): DBXIN211642

**Background**

The maternal mortality ratio for Black women in the United States is two to three times greater than white women, with 55.3 deaths per 100,000 live births in 2020. The Centers for Disease Control and Prevention notes that 80% of these deaths are preventable, acknowledging the role of racism and the need to address disparities in pregnancy-related mortality. The Black Mamas Matter Alliance (BMMA) is a national network of Black women-led organizations and professionals whose work is rooted in the birth/reproductive justice, respectful maternity care, and human rights framework. The alliance is made up of 40 community-based organizations and 25 individual collaborators.

**Methods**

BMMA approaches our work through four pillars: policy, research, care, and culture shift. We introduce and advance policy grounded in the human rights framework that addresses Black maternal health inequity and improves Black maternal health outcomes [Policy]; leverage talent and knowledge that exist in Black communities and cultivate innovative research methods to generate the evidence base for Black maternal health and inform the policy agenda to improve Black maternal health [Research]; promote holistic and comprehensive care for better outcomes for Black women, their children, families, and ultimately their communities [Care]; and redirect and reframe conversations on Black maternal health and amplify the voices of Black Mamas [Culture Shift].

**Results**

BMMA has made significant progress in advancing Black maternal health, rights, and justice in the United States. In 2018, BMMA founded the national Black Maternal Health Week, which led to the White House marking the first-ever federal Maternal Health Day of Action in 2021. BMMA also founded the Black Maternal Health Conference and Training Institute with more than 1,000 attendees in 2022. BMMA provides opportunities for professionals to gain continuing education credits and increase knowledge, skills, and competencies in Black maternal health through trainings and webinars. We convened our partners around strategic topic areas and created working groups, fellowships, and publications across our four focus areas.

**Conclusions**

By centering Black women’s leadership, BMMA’s work has the potential to change lives beyond maternal health and further support broader systems change by addressing racial and gender inequity and supporting holistic models of care.

## F3.1. My job, my voice: harnessing midwives’ demands at scale to strengthen the enabling environment for midwifery

### Kimberly Whipkey, Angela Nguku, Elena Ateva

#### White Ribbon Alliance

*BMC Proceedings 2024*, **18(5):**F3.1

Submission ID #: IMNHC651

Panel ID# (if applicable): DDPCP78651

**Panel Description**

Midwives play a pivotal role in reducing maternal and neonatal mortality and stillbirths in low- and middle-income countries and providing women with sexual and reproductive health services. Yet, the midwifery workforce is in crisis. There is an estimated global shortage of 900,000 midwives due to fewer intakes into educational institutions; inadequate investment in recruitment, deployment, and remuneration; and a weak supportive environment.

White Ribbon Alliance (WRA) pioneered an innovative programmatic approach that both increases political will to invest in midwives and accelerates national policy change, all in accordance with midwives’ priorities. Midwives’ Voices, Midwives’ Demands (MVMD) is a groundbreaking campaign launched in 2021 by WRA, the International Confederation of Midwives (ICM), and partners. The campaign was based on an open-ended question: “What do you want most in your role as a midwife?” WRA and ICM selected nine focus countries in Africa, Asia, and Europe for intensive outreach. Trained mobilizers queried health care providers and recorded responses using the What Women Want Chatbot—a novel digital tool that uses WhatsApp and artificial intelligence to rapidly capture demands and analyze results. Responses from non-focus countries were collected via an e-survey.

MVMD reached 56,000+ midwives in 100 countries. The two largest demands were for more and better-supported personnel, including increased salaries, and for supplies and functional facilities. This panel will explore how campaign partners have used the MVMD process and results to strengthen midwifery policy development and implementation in their countries, creating a more conducive environment based on what midwives say.

## F3.2. Amplifying midwives’ perspectives and priorities in the government of India’s rollout of midwifery-led care

### Chaitanya Tupaki Sreepoorna^1^, Kimberly Whipkey^2^

#### ^1^Centre for Catalyzing Change; ^2^White Ribbon Alliance

*BMC Proceedings 2024*, **18(5):**F3.2

Submission ID #: IMNHC1419

Panel ID# (if applicable): DDPCP78651

**Background**

The Nurse Practitioner in Midwifery (NPM) is a new cadre launched by the Government of India (GoI) in 2018. Rollout of NPMs at the national and state levels was significantly stymied due to COVID-19. The Midwives’ Voices, Midwives’ Demands (MVMD) campaign refocused attention on midwives, mobilizing more than 10,000 responses nationwide from NPMs and other cadres providing maternal and newborn health services. Top asks included better facilities and adequate supplies, more staffing and better remuneration, better respect and recognition of the role, and improved autonomy and gender norms.

**Methods**

Following the mobilization effort, in 2022, the Centre for Catalyzing Change/White Ribbon Alliance India (WRAI) implemented strategic advocacy activities to disseminate MVMD findings and support midwifery research and action agendas. For example, WRA India developed a Charter of Demands with a Call to Action to maternal health experts at the national and state levels, and disseminated campaign findings with midwifery multi-stakeholders in consultations and convenings. WRA India also brought midwives’ demands to technical discussions on implementation research and practice and launched a communications campaign to mobilize policy and programmatic support for their demands.

**Results**

The MVMD campaign helped fortify government efforts to integrate midwifery-led care in India’s public health system, with demands being particularly useful in supporting implementation of new policies. For example, in June 2021, the GoI launched a first-ever Scope of Practice for midwives. Although the scope of practice has been rolled out, awareness and understanding among practitioners have been uneven. WRA India used its MVMD Call to Action—which elevates midwives’ demands for better recognition of their role and increased autonomy—to socialize the scope of practice and call for empowering midwives to legally practice to their full scope. WRA India is now working with State Nursing Councils on additional licensure needed for midwifery practice that includes the full scope of practice.

**Conclusions**

MVMD has contributed significantly to making midwives’ demands central to ensuring the success of the GoI’s national rollout of its midwifery program. An MVMD campaign effort can be especially valuable, formative, and catalytic in countries where midwifery cadres are being newly instituted.

## F3.3. Creating a national vision and strategic framework for the practice of midwifery in Pakistan reflective of midwives’ priorities

### Rafia Rauf Shakeel, Kimberly Whipkey

#### White Ribbon Alliance

*BMC Proceedings 2024*, **18(5):**F3.3

Submission ID #: IMNHC1415

Panel ID# (if applicable): DDPCP78651

**Background**

Midwifery is at an inflection point in Pakistan. The country faces an eight-fold shortage of midwives, and the Community Midwives (CMWs) program has not been as successful as envisaged. The federal government, in response, has embarked on developing a new National Vision and Strategic Framework for Midwifery (Strategic Framework). At the same time, Forum for Safe Motherhood (FSM) launched its Midwives’ Voices, Midwives’ Demands campaign (MVMD). FSM heard from 6,145 midwives from across Pakistan including CMWs, Nurses, and Lady Health Visitors. More and better-supported personnel emerged as the top request.

**Methods**

To shape and align the Strategic Framework with midwives’ priorities, FSM first performed a deeper analysis of campaign demands, examining the geography and cadre. FSM augmented this analysis with midwives’ personal testimonials, especially those of CMWs, to further characterize their challenges and solutions for change. Next, FSM shared MVMD findings and stories with all key stakeholders from the public and private sectors. To build additional buy-in, especially for policy implementation, FSM and partners organized provincial and regional consultative sessions across Pakistan with major academic institutions, provincial departments of health, and directorates of nursing.

**Results**

MVMD campaign demands not only influenced the architecture of the Strategic Framework, but also shaped the policy actions contained therein. The largest asks from midwives, especially from CMWs, were for government jobs, better support and pay, and a career pathway—all of which were incorporated. Specifically, the Strategic Framework calls for regularization and creation of vacancies for midwives in the government health system; proper policy implementation of the pay scale, with time-scale promotion, remuneration, and upgradation with additional qualification; and development of a proper career ladder, job description, and monitoring mechanisms for midwives.

**Conclusions**

MVMD brought invaluable field evidence to policy discussions and compelled decision-makers to craft policy actions that responded directly to midwives’ concerns. It also enabled decision-makers to consider the perspectives of those rarely heard, such as CMWs working in rural and remote areas. In addition to guiding the national vision, the Strategic Framework will now serve as a roadmap for provincial midwifery strategies and midwives’ perspectives will once again serve as the evidence for action.

## F3.4. Midwives’ voices inform and catalyze a robust national midwifery agenda in Kenya

### Sandra Mwarania, Kimberly Whipkey

#### White Ribbon Alliance

*BMC Proceedings 2024*, **18(5):**F3.4

Submission ID #: IMNHC1406

Panel ID# (if applicable): DDPCP78651

**Background**

Although midwives and nurses make up most of the health workforce in Kenya, government policies and investments have historically undervalued midwives. As part of the Midwives’ Voices, Midwives’ Demands (MVMD) campaign, White Ribbon Alliance (WRA) Kenya captured the perspectives of more than 3,500 midwives across the country, with top asks related to supplies, increased salaries, and more staff. The campaign coincided with the national government’s push to develop a new national policy on nursing and midwifery, presenting an opportunity to infuse midwives’ priorities.

**Methods**

In 2021, prior to the launch of MVMD, WRA Kenya implemented a listening exercise with midwives in the Lake Region counties. The challenges they surfaced—shortage of midwives, lack of equipment and supplies, and absence of career pathways—were a harbinger of the MVMD findings to come. WRA Kenya shared these findings with decision-makers to inform early drafts of the national policy. WRA Kenya also organized workshops with MVMD participants to strategize and develop collective advocacy strategies. Several of these midwives were influential members of the taskforce that developed the national policy. Finally, as the national policy was being readied, WRA Kenya partnered with the Midwives Association of Kenya to host a national roundtable with 150 stakeholders and disseminate MVMD results.

**Results**

In May 2022, the Kenyan Government launched its first-ever National Nursing and Midwifery Policy recognizing midwifery in Kenya as an independent profession. The policy responds directly to the needs of midwives, calling for strengthened education, better remuneration, increased supplies and facility-level investments to enable midwives to deliver quality care, and professional development pathways for the midwifery workforce. MVMD, moreover, helped accelerate other policy outcomes including an expanded scope of practice for midwives and positive governance changes in the professional midwives’ association.

**Conclusions**

Although many actors contributed to midwifery policy action in Kenya, MVMD has proven to be both insightful and catalytic. The campaign also supported and emboldened midwives to self-advocate at decision-making tables and witness uptake of their voices in policy. The launch of this policy serves as an important first step to lay a foundation toward a sustainable future for the profession.

## F4.1. Innovations to support safe oxygen use with Continuous Positive Airway Pressure (CPAP) for the small or sick newborn care to achieve Every Newborn Action Plan (ENAP) target 4

### Leah Greenspan^1^, Tamah Kamlem^2^, Robert Neighbour^3^, Martha Franklin Mkony^4^, Harish Kumar^5^

#### ^1^United States Agency for International Development; ^2^Chemonics International; ^3^Diamedica (UK) Ltd; ^4^Muhimbili National Hospital, Tanzania; ^5^IPE Global Limited

*BMC Proceedings 2024*, **18(5):**F4.1

Submission ID #: IMNHC676

Panel ID# (if applicable): DDYBT47676

**Panel Description**

Eight years away from reaching the targets of the Sustainable Development Goals (SDGs), the UN Inter-agency Group for Child Mortality Estimation (UN IGME) database suggests that 63 countries are not on track to meet these targets, noting that no country will reach the SDGs without providing quality small and sick newborn care (SSNC) at the facility level 2, which includes high-impact interventions for the management of respiratory distress. Providing safe oxygen and effective pressures to newborns with respiratory distress and pneumonia is critical to providing quality care to 30 million newborns requiring inpatient care each year. The World Health Organization’s Standards call for specific medical equipment appropriate for SSNC. In response, global innovators have designed equipment for respiratory support that provides effective pressure generation, blended oxygen, and oxygen saturation monitoring to prevent oxygen toxicity in premature newborns.

ENAP sets national targets for 2025; 80% of districts to have at least one functional level 2 inpatient unit for small and sick newborns, including CPAP. To guide countries’ efforts toward achieving global targets, the World Health Organization and UNICEF have developed standards for improving the quality of SSNC in health facilities and a model of level 2 care for small and sick newborns.

This session will feature global innovators presenting innovative CPAP decision-making tools, three different categories of CPAP devices, and in-country user experiences from the perspectives of the patient, parent, and health care provider.

## F4.2. Oxygen concentrator driven baby Continuous Positive Airway Pressure (CPAP)

### Robert Neighbour^1^, Leah Greenspan^2^

#### ^1^Diamedica (UK) Ltd; ^2^United States Agency for International Development

*BMC Proceedings 2024*, **18(5):**F4.2

Submission ID #: IMNHC830

Panel ID# (if applicable): DDYBT47676

**Background**

The benefits of properly controlled CPAP for a range of clinical conditions in neonates is well established. In low-resource settings the cost and logistics of providing the required levels of oxygen and air must be factored into equipment provision. A 6,000 litre oxygen cylinder may need to be replaced more than once a day, resulting in prohibitively high lifetime costs.

The requirements for appropriate CPAP are: adequate flowrates of both oxygen and air; stable controlled pressure reflected at the patient interface; independence between flowrate and pressure; and minimal imposed work of breathing (iWOB). Warming and humidification of the gases is also an established benefit to help avoid septum necrosis, nasal obstruction, and hypothermia.

**Methods**

Oxygen concentrator technology is well established and accepted in most low-income settings. Diamedica (UK) Ltd utilized this base technology to develop a CPAP device that would deliver all the required elements of effective neonatal CPAP at lower running costs, namely the cost of electricity to run the concentrator. A novel design approach utilized the concentrator compressor to provide controlled flows of oxygen and air, warmed and humidified using waste heat from the concentrator. Two models were developed, providing either 5 or 10 L/min each of both oxygen and air, the full range of FiO2 is controllable. Everything required to deliver CPAP is provided in a single mobile unit, facilitating ease of setup and use. Voltage stabilization is supplied as standard, with optional battery backup.

**Results**

The device met all the requirements for appropriate CPAP listed above, and achieved regulatory approvals. Diamedica’s CPAP device is currently used in 30 countries with more than 2,700 units supplied. Extensively tested and validated, independently published research from Uganda established that the introduction of this equipment resulted in a substantial reduction in neonatal mortality

**Conclusions**

Diamedica’s CPAP device has fulfilled both technical and affordability requirements of the original design objective. The initial cost of the units was offset by their minimal running costs, providing a highly cost-effective device. An independent paper from Nigeria concluded that in circumstances of limited, or expensive oxygen provision, it was the only viable option for affordable neonatal CPAP.

## F4.3. Implementation of low-cost Effective Continuous Positive Airway Pressure (CPAP) and Bubble CPAP (bCPAP) in Low- and Middle-Income Countries (LMICs) to Address Respiratory Distress Syndrome (RDS) for small and sick newborns

### Kondwani Kawaza^1^, Leah Greenspan^2^, Harish Kumar^3^

#### ^1^Kamuzu University of Health Sciences (KUHES); ^2^United States Agency for International Development; ^3^IPE Global Limited

*BMC Proceedings 2024*, **18(5):**F4.3

Submission ID #: IMNHC829

Panel ID# (if applicable): DDYBT47676

**Background**

The U.S. Agency for International Dvelopment is currently demonstrating models for improving the quality of facility-based care for small and sick newborns (SSNB) being implemented in India and Malawi, with the use of innovative CPAP devices to improve newborn outcomes and to help the countries achieve Sustainable Development Goals. Studies have revealed challenges in health systems in the provision of safe oxygen use in LMICs. bCPAP is an evidence-based intervention for newborns with respiratory distress and if implemented safely can be an effective mode of therapy. Assessments have shown that there is a weak capacity among health care providers for the management of RDS, and limited availability of blenders and regulated devices.

**Methods**

Two bCPAP models, both designed with input from in-country expertise, were implemented in secondary-level hospitals in Malawi and India. In Malawi, the Pumani bCPAP device was rolled out in 24 district hospitals. This device includes a driver unit with a bubble bottle for pressure control, an inspiratory tube connected to Hudson prongs, and a port for an external oxygen source. In India, the Sanns was implemented in seven facilities across two states. With a design similar to Pumani’s, it is a locally manufactured and approved low-cost bCPAP device. Its unique features include external display unit and backup battery. Prior to implementation, facility assessments and staffing practices and gaps for standards were evaluated.

**Results**

The Sanns model was used for the care of 220 newborns with respiratory distress with no complications. Implementation of the Pumani model resulted in improvement of survival to discharge for neonates with respiratory distress (48.6% vs. 54.5%; *P* = .012). Providers are comfortable using the devices.

**Conclusions**

The implementation of CPAP devices in Malawi and India provide insightful learnings on ways to institutionalize a critical quality standard for newborn care.

## F4.4. Situational analysis of prevalence of improvised Bubble Continuous Positive Airway Pressure (bCPAP), 100% oxygen use, and pulse oximetry monitoring use in level 2 facilities in Ghana

### Tamah Kamlem^1^, Leah Greenspan^2^

#### ^1^Chemonics International; ^2^United States Agency for International Development

*BMC Proceedings 2024*, **18(5):**F4.4

Submission ID #: IMNHC693

Panel ID# (if applicable): DDYBT47676

**Background**

CPAP devices have been widely supported for introduction at scale to reduce neonatal mortality from respiratory distress syndrome (RDS), a common cause of neonatal deaths. Every Newborn Action Plan set a target of 80% for level 2 facilities to provide CPAP by 2025.

Although these efforts have increased the availability of CPAP devices, the global newborn community is concerned with the following: 1. A large number of bCPAPs in low- and middle-income countries do not have blenders or routine access to electricity and are relying exclusively on 100% O2; 2. Use of 100% oxygen can lead to oxygen toxicity, which can result in retinopathy of prematurity or chronic lung disease; and 3. Without gradual data to drive prioritization of updating respiratory guidelines, demand for investments in innovative safe delivery of oxygen, and continued creative solutions, neonates may continue to receive unsafe oxygen delivery.

**Methods**

A mixed method approach will be used for the assessment with random sampling of level 2 health facilities in Ghana. This quantitative approach will seek to provide data on prevalence of improvised bCPAP, 100% oxygen use and pulse oximetry monitoring use in level 2 facilities in Ghana through the collection and analysis of numerical data. The qualitative approach will include a semi-structured interview guide to obtain feedback from providers, experts, and key informants on the use of CPAP.

**Results**

The study will provide data on the prevalence of improvised bCPAP, 100% oxygen use, and pulse oximetry monitoring use in level 2 facilities in Ghana. Additionally, providers’ capacity, behavior, and availability and maintenance of devices will be investigated to determine potential gaps and areas of improvement.

**Conclusions**

The study will provide insight into the current state of respiratory mechanisms at level 2 facilities in Ghana for small and sick newborns and determine existing challenges regarding the supply chain of medical devices for newborn health. Results will inform the policy landscape of newborn health to stakeholders at the national level and beyond.

## F4.5. Novel high-quality Bubble Continuous Positive Airway Pressure (bCPAP) and oxygen blender systems for global access

### Martha Franklin Mkony^1^, Thomas Burke^2^, Leah Greenspan^3^

#### ^1^Muhimbili National Hospital, Tanzania; ^2^Vayu Global Health Foundation; ^3^United States Agency for International Development

*BMC Proceedings 2024*, **18(5):**F4.5

Submission ID #: IMNHC685

Panel ID# (if applicable): DDYBT47676

Note the full manuscript published in December 2022 in *PLOS ONE:*

(https://www.ncbi.nlm.nih.gov/pmc/articles/PMC9803298/)

## F5.1. Maternal mortality: levels, trends, and strengthening reporting

### Jenny Cresswell^1^, Lale Say^1^, Imbulana Jayaratne^2^, Affette McCaw-Binns^3^

#### ^1^World Health Organization; ^2^Ministry of Health, Sri Lanka; ^3^University of the West Indies, Mona

*BMC Proceedings 2024*, **18(5):**F5.1

Submission ID #: IMNHC1353

Panel ID# (if applicable): DFHYE111353

**Panel Description**

The reduction of maternal mortality has long been a global health priority and remains a prominent part of the Sustainable Development Goals (SDGs) agenda. The SDGs include a direct emphasis on reducing maternal mortality, while also highlighting the importance of moving beyond survival. SDG target 3.1—to reduce global maternal mortality ratio (MMR) to less than 70 per 100,000 live births by 2030—is an ambitious target and will require sustained commitments to ensure that women and adolescent girls get quality care and support when and where needed.

The United Nations Maternal Mortality Estimation Interagency Group, comprising the World Health Organization (WHO), United Nations Children’s Fund (UNICEF), United Nations Population Fund (UNFPA), the World Bank Group, and the United Nations Population Division, has collaborated with external academic teams and technical experts on a new round of maternal mortality estimates covering the period 2000–2020. These new MMR estimates supersede all previous estimates and are based on the most up-to-date data and methods, assessing national, regional, and global trends monitoring progress toward 2030. The panel will also report updated causes of maternal death estimates from WHO.

Having targets for maternal mortality reduction is important; however, accurate data remain the key to robust policy and programming. Authors from Sri Lanka and Jamaica will share data and processes to quantify and reduce missed and misclassified maternal deaths. Enhanced reporting will allow better data use, informing maternal cause of death attribution, relevant interventions, and tracking including interpretation of the gaps in coverage.

## F5.2. Structured processes built upon the original Maternal Death Surveillance and Response (MDSR) system: Sri Lanka

### Imbulana Jayaratne^1^, Jenny Cresswell^2^

#### ^1^Ministry of Health, Sri Lanka; ^2^World Health Organization

*BMC Proceedings 2024*, **18(5):**F5.2

Submission ID #: IMNHC1399

Panel ID# (if applicable): DFHYE111353

**Background**

Sri Lanka is a lower middle-income country with low maternal mortality, and is considered a success story in terms of both reducing maternal mortality and improving its measurement. Sri Lanka has an established history of maternal death monitoring. The MDSR system was started in 1959, with the notification of probable maternal deaths being made mandatory in 1985. A decade later, a structured review of probable maternal deaths was initiated, and a national database has been in place since 2000.

**Methods**

Mandatory death notification in Sri Lanka is a key strength of the MDSR system in ensuring high levels of completeness. This presentation will describe the steps and characteristics of the system, and how these features strengthen reporting and improve outcomes. For example, postmortems must be sought for all probable maternal deaths to confirm pregnancy or recent delivery or pregnancy loss/termination among women of reproductive age, contributing to delineating pathology and more accurately determining the cause of death. In addition, Sri Lanka also uses community health workers (field public health midwives) to notify of any potential maternal deaths that took place both inside and outside of health care facilities, minimizing the potential for underreporting.

**Results**

The surveillance process in Sri Lanka has a clear and established “no name, no blame” policy at all stages. This assurance of confidentiality helps to give confidence in accurate reporting without the fear of repercussions or reprisals, ensuring that appropriate, evidence based actions can then be taken to make improvements. One challenge for the system has been a time lag between the notification of the death and the availability of review outcomes, which are needed for decision-making. To help to address this issue, the Ministry of Health has introduced an immediate response system, whereby the Director General of Health Services (the highest level administrator) convenes a fact-finding discussion with all caregivers in cases where service gaps have been identified.

**Conclusions**

Robust systems with multiple checks and feedback loops strengthen maternal mortality reporting and help data improve outcomes to reduce preventable maternal mortality. These practices are demonstrated in the Sri Lanka model.

## F5.3. Vital registration as a reliable source of maternal mortality information: Jamaica’s experience with efforts to improve data quality and completeness, 2018–2021

### Affette McCaw-Binns^1^, Jenny Cresswell^2^

#### ^1^University of the West Indies, Mona; ^2^World Health Organization

*BMC Proceedings 2024*, **18(5):**F5.3

Submission ID #: IMNHC1390

Panel ID# (if applicable): DFHYE111353

**Background**

Reliable data systems are critical to monitoring survival for rare conditions like maternal deaths, given that small variations impact measurement accuracy. In 1998, Jamaica established an active maternal mortality surveillance system (MMS) and added death certification to the medical curriculum; however, between 1998–2007, just 126/428 (29%) of maternal deaths appeared in vital registration statistics. A pregnancy checkbox was added to the medical certificate of cause of death in 2014. We aim to determine if these interventions have improved retrieval of maternal deaths from vital registration.

**Methods**

Vital registration data for 2018–2021 were triangulated with MMS information to determine completeness.

**Results**

Of 227 maternal deaths identified for 2018–2021, 62% (*n*=140) were registered as maternal deaths, including 24 (11%) missed by MMS. The MMS accounted for 80% (*n*=183) of cases, with 51% (*n*=126) common to both sources. Another 16% (*n*=37) were registered, but misclassified as non-maternal deaths, and 13% (*n*=30) were not registered. Misclassification included both inflation due to certification of late maternal deaths as maternal deaths, and under-reporting, with true maternal deaths missed because of certification/coding errors, and non-registration. In 2021, 43% of maternal deaths were due to COVID-19 (cause-specific MR=115; total maternal mortality ratio=258/100,000); and two-thirds of COVID-19 cases were misclassified.

**Conclusions**

The pregnancy checkbox has improved maternal mortality case identification through vital registration from 29% to 70% in 15 years; however, gaps remain related to non-registration of community maternal deaths/coroner’s cases. Certification/coding of indirect maternal deaths should reflect the changing disease profile of antenatal women, including their susceptibility to infections such as COVID-19. Global reporting should reflect these risks alongside the traditional direct complications of pregnancy.

## F5.4. Global and regional causes of maternal deaths 2009–2017: a World Health Organization (WHO) systematic analysis

### Jenny Cresswell

#### World Health Organization

*BMC Proceedings 2024*, **18(5):**F5.4

Submission ID #: IMNHC1385

Panel ID# (if applicable): DFHYE111353

**Background**

The current level of maternal mortality remains unacceptably high and is not on track to meet the Sustainable Development Goal targets. To successfully reach targets, information about cause of death is needed to inform interventions and programs. The aim of this study was to develop estimates of the causes of maternal deaths at global and regional levels for the period from 2009 to 2017.

**Methods**

We conducted a systematic review of bibliographic databases, theWHO’s Member State websites, and WHO’s records for reports of maternal deaths, with no language restrictions. Civil registration and vital statistics data were extracted from the WHO Mortality Database. We assessed the reports according to pre-specified criteria. Regional and global estimates of the maternal cause of death distribution were estimated using a Bayesian hierarchical model.

**Results**

We included data from 142 countries and 950 country years. Globally, the most common cause of maternal death was hemorrhage (26%; 80% uncertainty interval [UI]: 22%–30%), followed by indirect obstetric deaths (23%, 80% UI: 19%–29%), and hypertension (16%; 80% UI: 14%–19%). There was substantial regional variation in the proportion of deaths due to hemorrhage. Data on maternal suicide and late maternal deaths were very inconsistently reported. Few countries reported deaths due to maternal suicide in developed regions, with only five countries had data recording one or more maternal suicide deaths. In low- and middle-income regions, we estimated between 3%–8% of maternal deaths were due to suicide The ratio of late maternal deaths to maternal deaths up to 42 days was 0 to 0.1 among those countries that reported at least one such death.

**Conclusions**

The global distribution of the causes of maternal deaths remained similar in broad terms between 2003 to 2009 and 2009 to 2017. This update includes for the first time an analysis attempting to report on maternal suicide as a cause of death. Hemorrhage remains the leading individual cause of death, despite the existence of effective clinical interventions. Improving access to emergency obstetric care provided by competent health care providers and good quality referral networks therefore remain vital.

## F5.5. Trends in maternal mortality 2000 to 2020: estimates by the World Health Organization (WHO), United Nations Children’s Fund (UNICEF), United Nations Population Fund (UNFPA), World Bank Group, and United Nations Population Division

### Lale Say, Jenny Cresswell

#### World Health Organization

*BMC Proceedings 2024*, **18(5):**F5.5

Submission ID #: IMNHC1364

Panel ID# (if applicable): DFHYE111353

**Background**

To monitor progress toward Sustainable Development Goal (SDG) indicator 3.1 of reducing the global maternal mortality ratio (MMR) to less than 70 by 2030, the UN’s Maternal Mortality Estimation Inter-Agency Group (consisting of WHO, UNICEF, UNFPA, World Bank Group, and UN Population Division) (UN-MMEIG) develop and disseminate internationally comparable estimates of maternal mortality. The aim of this research was to estimate comparable global, regional, and country estimates of maternal mortality levels and trends covering the period 2000–2020.

**Methods**

We updated the UN-MMEIG’s input databases using systematic searches. Data sources included were civil registration and vital statistics, censuses, special maternal mortality surveillance systems and studies, and national population-based surveys. We estimated maternal mortality levels and 80% uncertainty intervals using two statistical models: a Bayesian misclassification model to account for errors in reporting of maternal deaths and a Bayesian maternal mortality estimation model to estimate the MMR for each country year.

**Results**

Nearly all maternal deaths were found to be due to preventable causes. The lifetime risk of dying from a maternal cause varies substantially globally: a 15-year-old in Africa has around a 1 in 37 chance of eventually dying due to a maternal cause compared to around 1 in ~3,750 in Europe. Countries that are humanitarian or fragile settings have a particularly high burden of maternal mortality.The average rate of reduction of maternal mortality has slowed in recent years. This is a concerning trend: progress is not currently on track to meet the SDG targets.

**Conclusions**

Despite global progress in reducing maternal mortality, further action is needed to meet the ambitious SDG 3.1 2030 target, and ultimately eliminate preventable maternal mortality . There is a continued urgent need for maternal health and survival to remain high on the global health agenda—ensuring that women and adolescent girls have access to quality care when needed.

## F6.1. Enhancing maternal and newborn health in humanitarian and fragile settings with integrated service delivery

### Christopher Lindahl^1^, Renee Fiorentino^1^, Nancy Ibrahim^2^, Dan Wendo^1^, Samia Mohammed^3^, Grace Carina Viola^4^

#### ^1^MOMENTUM Integrated Health Resilience; ^2^Save the Children US; ^3^Building Foundation for Development; ^4^United Nations Population Fund

*BMC Proceedings 2024*, **18(5):**F6.1

Submission ID #: IMNHC1081

Panel ID# (if applicable): DHMBK931081

**Panel Description**

Integration of service delivery components is a challenge in the best of circumstances, not least because vertical funding streams often work against it. Yet, in all settings, truly patient-centered care is integrated, maximizing efficiency of contact between care-seeking families and their care teams. In settings characterized by conflict, post-conflict, the effects of climate change, frequent government turnover, and other shocks and stresses, quality integrated care becomes that much more of an imperative.

This panel will feature Save the Children speaking about the importance of supporting integrated primary health care in Somaliland; MOMENTUM Integrated Health Resilience will share immediate postpartum and post-abortion family planning trends in supported counties in South Sudan; Building Foundation for Development will present its lessons learned implementing mobile medical teams providing an integrated package of services in Yemen; and the United Nations Population Fund will speak about its success improving outcomes through the offer of cash assistance for healthy pregnancies and safe births in the Philippines.

Each panelist will highlight the ways in which integration helped overcome difficulties particular to the setting in which they work and improved holistic care for mothers and their newborns.

## F6.2. Cash assistance for healthy pregnancies and safe births in the Philippines

### Grace Carina Viola^1^, Christopher Lindahl^2^, Renee Fiorentino^2^, Alice Golay^1^

#### ^1^United Nations Population Fund; ^2^MOMENTUM Integrated Health Resilience

*BMC Proceedings 2024*, **18(5)**:F6.2

Submission ID #: IMNHC1094

Panel ID# (if applicable): DHMBK931081

**Background**

The United Nations Population Fund (UNFPA) Philippines provided cash assistance to pregnant women in conflict-and typhoon-affected areas to address the increased economic barriers women and girls faced, resulting in delays in accessing quality and timely maternal care. Thanks to the cash assistance program, facility-based deliveries and attendance at checkup visits increased. The Philippines program is part of UNFPA’s global commitment to use cash as a tool to enhance access to sexual and reproductive health (SRH) services for women and girls.

Cash assistance was provided in complement to interventions such as SRH medical missions and SRH, gender-based violence, and psychosocial support, and COVID-19 messaging, multiplying its impact. UNFPA is planning to implement similar approaches in Indonesia, Syria, and Yemen.

**Methods**

In addition to regular program monitoring and evidence building, eligible pregnant women and adolescents registered into the cash assistance program and a comparison group are surveyed after receiving the cash support on its impact on key outcomes: 1) attendance at antenatal care visits; 2) facility-based delivery; 3) attendance at postpartum care visits; 4) perception of the service in terms of quality; and 5) safety. Evaluation findings will be available by the end of 2022, in time for the conference. Evaluation findings are available here.

**Results**

Results of the internal program monitoring are as follows: out of 1,000 pregnant women registered in the program, pregnant women attending a first antenatal visit increased from 31% to 96%, women delivering in a facility increased from 28% to 68%, and women attending at least one postnatal consultation increased from 38% to 87%.

**Conclusions**

Integrating cash assistance into SHR programming appears to be highly successful. The evaluation with Johns Hopkins University complements ongoing monitoring of maternal, infant, and child programming, and expands program learnings beyond current indicators to provide a more comprehensive understanding of the impacts of cash for SRH in the Philippines context and understand how efficiently and effectively financial incentives can help change attitudes and behaviors.

## F6.3. Mobile medical teams responding to health emergencies in humanitarian settings via the provision of integrated health services

### Samia Mohammed^1^, Christopher Lindahl^2^, Renee Fiorentino^2^

#### ^1^Building Foundation for Development; ^2^MOMENTUM Integrated Health Resilience

*BMC Proceedings 2024*, **18(5):**F6.3

Submission ID #: IMNHC1087

Panel ID# (if applicable): DHMBK931081

**Background**

For women in Yemen, access to adequate and affordable health care has been significantly hampered by ongoing crisis and conflict. Most women from remote areas who suffer problems and complications during pregnancy or delivery are due to the poor health infrastructure capacity, lack of available facilities, and limited essential supplies and staff. To fill gaps in services, Building Foundation for Development, with funding from the United Nations Population Fund, is running emergency response mobile medical teams (MMTs) in Ma`rib, Yemen.

**Methods**

The MMTs aim to bring lifesaving services closer to those in need by using mobile clinics to service remote areas. Each MMT includes qualified health workers who have community knowledge: one general practitioner, two midwives, two nurses, one pharmacist, and one psychologist. The composition of the MMTs allows for provision of an integrated package of services at the point of care, including: primary health care, antenatal care, and reproductive health services, nutrition, immunization, MHPSS, and referral services. Additionally, the teams can support survivors of gender-based violence and provide psychological care to internally displaced persons and host communities with both first aid treatment and referral to specialists if required.

**Results**

In the first half of 2022, 55,391 people received services from the MMTs. This new program has allowed Building Foundation for Development to quickly adapt to population movements and conduct visits to multiple sites, ensuring that health services are equitably distributed across affected communities. The success of the MMTs has resulted in expanded access to high-quality services (including medicines and supplies). Moreover, the MMTs are appreciated by users as an effective strategy, particularly in the context of displacement and continuous conflict.

**Conclusions:** Thousands of people have been displaced following the conflict in and around Ma`rib. The majority of the displaced are women and children, many of whom have both physical and mental health care needs. MMTs have been an effective strategy for reaching populations in need in Ma`rib.

## F6.4. Immediate postpartum and postabortion family planning trends in counties supported by MOMENTUM integrated health resilience in South Sudan

### Lou Eluzai, Renee Fiorentino, Christopher Lindahl, Dan Wendo, Mary Jackson, Zechariah James

#### MOMENTUM Integrated Health Resilience

*BMC Proceedings 2024*, **18(5):**F6.4

Submission ID #: IMNHC1085

Panel ID# (if applicable): DHMBK931081

**Background**

South Sudan continues to experience decades-long humanitarian crises impeding access to quality maternal and neonatal services. Early marriages and childbirth, together with repeated closely spaced pregnancies, contribute to high maternal and infant deaths. Contraceptive use is low (4.3 %) and unmet need is high (29.7%); annually, 26% of pregnancies are unintended and 46% end in abortion. MOMENTUM Integrated Health Services (MIHR) is improving healthy timing and spacing of pregnancies by strengthening access to, and quality of, voluntary postpartum family planning (PPFP) services among women at facilities and in their communities.

**Methods**

MIHR supports demand creation at facility and community levels to improve care-seeking behaviors, and strengthens facility readiness to provide education, counseling, and PPFP services. MIHR implements a whole-site facility integration approach in which pregnant or postpartum women/men are reached at various clinics with information (including antenatal, prevention of mother-to-child transmission of HIV, antenatal, maternity, postnatal, nutrition, immunization, and outpatient). All but those in the maternity clinic, where FP services are provided immediately postpartum, are referred to the FP clinic for provision. Community-level Boma Health Workers conduct several integrated visits, before and after delivery, and provide/refer for FP.

**Results**

Routine data from 24 facilities in eight counties from October 2021 to June 2022 show contraceptive uptake remained relatively the same—on average 2.3% of postpartum women/couples counseled per month voluntarily accepted an FP method. Roughly three-quarters of these acceptors came from a facility and one-quarter from the community. Half were adolescents and/or youth. These routine data echo population-level survey data findings, indicating only 3% of postpartum women use contraceptives.

**Conclusions**

Causal factors behind high unmet need and low uptake of FP in South Sudan are particular. Social norms regarding abstinence after childbirth, large families, and home deliveries may be contributing. MIHR is using the demographics above and assessing factors contributing to low uptake (through an experience of care study and an assessment of facilitators and barriers among postpartum women), and testing innovations such as community-based immediate PPFP for women delivering at home.

## F6.5. Improving quality and availability of maternal newborn services in Somalia Puntland through delivery of adapted capacity-building packages to health care workers: Maternal and Newborn Health (MNH) program, save the children

### Nancy Ibrahim^1^, Christopher Lindahl^2^, Renee Fiorentino^2^, Sarah Ashraf^1^

#### ^1^Save the Children US; ^2^MOMENTUM Integrated Health Resilience

*BMC Proceedings 2024*, **18(5):**F6.5

Submission ID #: IMNHC1083

Panel ID# (if applicable): DHMBK931081

**Background**

In Somalia, more than 80% of newborn deaths are due to prematurity and asphyxia complications. Deaths can be prevented by well-trained midwives and nurses during antenatal and postnatal visits and delivering at a health facility.

The MNH program in Somalia focuses on improving the quality and availability of maternal and newborn care through contextualization and integration of the Helping Babies Survive (HBS) and Helping Mothers Survive (HMS) training programs.

**Methods**

Save the Children supported the translation into the Somali language of the adapted HMS curriculum obtained from Jhpiego and the HBS curriculum from the American Academy of Pediatrics. These translated training modules were endorsed by the Ministry of Health.

Twenty-eight master trainers were trained. The master trainers conducted cascade trainings for 154 health care providers. The trainees subsequently passed on knowledge gained to 300 colleagues through coaching and supportive supervision at their respective workplaces and provided needed care to 6,000 women and their newborn babies.

Save the Children then advocated for the scale-up of integrated newborn and maternal care training across the country to improve relevant practices and outcomes.

**Results**

The proportion of newborns who received two or more essential newborn care practices (including skin-to-skin contact, cord care, and early breastfeeding) improved from 19.9% to 94.7% post-intervention. The study also showed significant skills retention, and improvement in knowledge and confidence among providers.

Overall, the average change in knowledge was 22.54%, and 41.5% in skills. This indicates a huge baseline need among providers, crucial to fill in order to keep increasing the number of maternal and newborn lives saved. The skills retention score mean difference at 18 months after the trainings was 11.9 %.

**Conclusions**

Adequate training, supportive supervision, and on-the-job coaching of providers can prevent deaths by proper management of the critical events newborns face such as hemorrhage, obstructed labor, eclampsia, sepsis, pre-term birth, and respiratory distress. Scaling up trainings is recommended.

## F7.1. A 13-year initiative to expand access to high-quality Emergency Obstetric and Newborn Care (EmONC) and family planning services in Tanzania

### Sunday Dominico^1^, Neena Prasad^2^, Florina Serbanescu^3^, Samantha Lobis^4^

#### ^1^Thamini Uhai; ^2^Bloomberg Philanthropies; ^3^Centers for Disease Control and Prevention; ^4^Averting Maternal Death and Disability Program

*BMC Proceedings 2024*, **18(5):**F7.1

Submission ID #: IMNHC409

Panel ID# (if applicable): DIPOP39409

**Panel Description**

The Program to Reduce Maternal Deaths in Tanzania was a 13-year initiative that worked to expand mostly rural communities’ access to high-quality EmONC and family planning services. The Tanzanian Ministry of Health and its partners worked together to enhance the quality of maternal and perinatal care and improve health outcomes in Kigoma region.

Sub-Saharan Africa accounts for only 15% of the world’s population but bears nearly 70% of the global burden of maternal deaths and about 40% of global newborn deaths. More than half of maternal and newborn deaths result from complications during childbirth and are largely preventable. However, poor quality of care in low-resource settings is often a greater contributor to poor health outcomes than health coverage. The Tanzanian Program model of decentralized obstetric, reproductive, and newborn care from distant hospitals to more accessible, lower-level health facilities through infrastructure upgrades and training the non-physician health care providers staffing these facilities demonstrated substantial reduction in maternal and perinatal deaths. The panel will present the history of the Program, key Program interventions, a population-based multilevel decomposition analysis that shows how a rapid increase in facility deliveries is mostly attributable to the increase in quality of health facilities, and an innovative birth companionship model that was scaled up and led to a policy change in Tanzania.

Lessons learned from the Program can inform policymakers and program managers in settings where similar approaches could be used to improve and sustain the utilization of quality facility care at birth.

## F7.2. Introducing, sustaining, and scaling up birth companionship in Tanzania

### Alex Mputa^1^, Sunday Dominico^1^, Agness Mbanza^1^, Nguke Mwakatundu^1^, Dunstan Bishanga^2^, Shanon McNab^3^, Irene Mashasi^3^, Selemani Mbuyita^3^, Samantha Lobis^3^

#### ^1^Thamini Uhai; ^2^Muhimbili University of Health and Allied Sciences; ^3^Averting Maternal Death and Disability Program

*BMC Proceedings 2024*, **18(5):**F7.2

Submission ID #: IMNHC413

Panel ID# (if applicable): DIPOP39409

**Background**

Having a companion of choice who provides continuous emotional, practical, and informational support throughout childbirth is an essential component of good quality, respectful care. From 2016–2019, Thamini Uhai systematically introduced birth companionship as a pilot in nine government health facilities in Kigoma as part of a program to reduce maternal deaths in Tanzania. Building on the pilot’s positive results, birth companionship was integrated into national policy and scaled up in the Kigoma and Katavi regions. Implementation and sustainability lessons learned during the pilot were built into the scaled-up efforts.

**Methods**

Modifications included developing accountability mechanisms to ensure good-quality implementation, training health care providers to promote birth companionship to women during antenatal care, and putting more emphasis on supporting companions to provide comfort measures (e.g., use of different birthing positions, relaxation techniques, and non-medical pain relief strategies). Interviews and focus group discussions were conducted with women, health care providers, and government officials in both regions in 2021.

**Results**

Birth companionship is now being implemented in 23 government health facilities in two regions. By April 2021, more than three-quarters of women giving birth at facilities in Kigoma and 96% in Katavi had birth companions. Women who had recently delivered spoke of the benefits of having a companion: receiving a massage to relieve backaches, having their hands held as a way of extending psychological support, and escorting them to the washroom and bringing them back to bed to rest.

Thamini Uhai was able to take these results, leverage their strong relationships with the local-level government, and meet with national-level policymakers to advocate for the institutionalization of birth companionship in Tanzania. As a result of the program, birth companionship features in the national guidelines and training framework for respectful maternity care. Moreover, scale-up of birth companionship has received strong backing from the President of the United Republic of Tanzania and the Ministry of Health.

**Conclusions**

Birth companionship is feasible, scalable, and highly acceptable among communities and health care providers in Tanzania, and now there is a policy framework and political will for scale-up countrywide.

## F7.3. Changes in individual, community, and health facility factors associated with using facility delivery services in rural Tanzania: a repeat multilevel and decomposition analysis

### Florina Serbanescu^1^, Paul Stupp^1^, Alicia Ruiz^1^, Michelle Schmitz^1^, Sunday Dominico^2^, Samantha Lobis^3^, Jason Hsia^1^

#### ^1^Centers for Disease Control and Prevention; ^2^Thamini Uhai; ^3^Averting Maternal Death and Disability Program

*BMC Proceedings 2024*, **18(5):**F7.3

Submission ID #: IMNHC412

Panel ID# (if applicable): DIPOP39409

**Background**

Delivering in a health facility with skilled health care providers is a key component in improving maternal and newborn health and achieving the Sustainable Development Goals for health by 2030. It was also the goal of the Program to Reduce Maternal Deaths in Tanzania. Our study examined changes in individual, community, and health facility factors related to the use of facility delivery services between 2014 and 2018 in a predominantly rural region of Tanzania, with the goal of prioritizing interventions to increase use of such services.

**Methods**

We analyzed data from women with recent births who participated in 2014 (*n*=1,628) and 2018 (*n*=4,165) population-based surveys in Kigoma Region, Tanzania. We combined data from surveys with data from health facility assessments (2013 and 2018) to determine what remains to be done to ensure adequate delivery services at facilitiies. We used a mixed-effects multilevel logistic regression analysis to assess the influence of individual-, community-, and facility-level factors on delivering in a health facility. Oaxaca-Blinder decomposition analysis quantified the extent to which the difference in facility delivery rates between 2014 and 2018 can be explained by changes in these factors.

**Results**

Facility delivery rate increased by 68% (from 44.7% to 75.4%). In both 2014 and 2018, women had significantly greater odds of facility delivery if they were literate adjusted odds ratio (aOR): 1.52 and 1.51; had high socioeconomic status (aOR: 2.3 and 1.4); had no previous births (aOR: 4.3 and 3.3); planned for transport (aOR: 4.8 and 2.4); resided in Kigoma town (aOR: 3.8 and 2.4) or Kibondo district (aOR: 4.1 and 2.5); and resided within 0–10 km of an adequate health facility (aOR: 5.8 and 6.7). Changes in the individual, community, and health facility factors evaluated explained 49% of the increase in facility delivery rate between 2014 and 2018. Most of the explained increase was accounted for by changes in two factors: proximity of an adequate health facility (55%) and transport planning (32%).

**Conclusions**

Efforts to maximize increased use of facility delivery in rural Tanzania may benefit from focusing on transport planning for birth and provision of adequate delivery services close to women’s place of residence.

## F7.4. Improving access to quality emergency obstetric and newborn care in Kigoma, Tanzania

### Sunday Dominico^1^, Nguke Mwakatundu^1^, Mkambu Kassanga^1^, Samantha Lobis^2^

#### ^1^Thamini Uhai; ^2^Averting Maternal Death and Disability Program

*BMC Proceedings 2024*, **18(5):**F7.4

Submission ID #: IMNHC411

Panel ID# (if applicable): DIPOP39409

**Background**

A 13-year program in Tanzania worked to expand underserved communities’ access to high-quality emergency obstetric and newborn care (EmONC) in three regions, with a primary focus on Kigoma. To achieve this goal, the program decentralized and sustained high-quality EmONC to non-hospitals by training and mentoring doctor and non-doctor cadres and upgrading and equipping facilities. The program’s focus on interventions to ensure skills maintenance of providers contributed to its successes.

**Methods**

The program provided extensive clinical support, which included: frequent on-site supervision and mentorship visits; in-person continuing medical education workshops; weekly teleconferences led by Tanzanian obstetricians to discuss cases and challenges; emergency call system for providers to access expert obstetricians 24/7 and receive clinical advice; and creation of an E-Learning Platform for continuous education of health care providers. A low-dose high-frequency training with simulation and a robust system of clinical audits were introduced and sustained. Action-oriented feedback was provided to health care providers,along with facility, and council and regional health management teams, to ensure improved quality of care. The program was externally monitored for target-tracking and real-time feedback.

**Results**

Between 2013 and 2018, 14 of the 15 program-supported health centers started to provide obstetric surgery, a key goal of the program; supported dispensaries provided an average of five out of seven basic EmONC signal functions. The facility delivery rate increased by 73% (from 49% to 85%). Met need for EmONC increased to 61%, while the direct obstetric case fatality rate declined (from 1.8% to 1.4%). The institutional maternal mortality ratio across all health facilities declined from 303 to 174 deaths per 100,000 live births, and the intrapartum stillbirth rate declined from 14.4 to 6.0 per 1,000 births. Most institutional maternal deaths in Kigoma were averted through care provided by program-supported facilities. The program provided a major contribution to meeting the obstetric needs of the region.

**Conclusions**

Decentralizing high-quality EmONC delivered mostly by associate clinicians and nurses improved the availability and utilization of lifesaving high-quality EmONC in Kigoma. The program’s approach is being replicated in other regions in Tanzania and should be considered by governments working to decrease maternal and newborn mortality in similar settings.

## F7.5. What does it take to reduce maternal and neonatal deaths in under-resourced, rural settings? Lessons from a 13-year initiative in Kigoma, Tanzania

### Neena Prasad^1^, Sunday Dominico^2^, Samantha Lobis^3^, Florina Serbanescu^4^

#### ^1^Bloomberg Philanthropies; ^2^Thamini Uhai; ^3^Averting Maternal Death and Disability Program; ^4^Centers for Disease Control and Prevention

*BMC Proceedings 2024*, **18(5):**F7.5

Submission ID #: IMNHC410

Panel ID# (if applicable): DIPOP39409

**Background**

The Program to Reduce Maternal Deaths in Kigoma (“Program”) was a 13-year collaboration (2006–2019) between the Government of Tanzania (GoT), nongovernmental organizations, and donors that sought to make high-quality care more available, accessible, and utilized.

**Methods**

The Program decentralized obstetric, reproductive, and newborn care from distant hospitals to more accessible, lower-level health facilities through infrastructure upgrades and training the non-physician health care providers staffing these facilities. Regular evaluations allowed the Program to identify and respond to gaps, which meant the Program’s strategy evolved over time. Such evolutions included incorporating demand-generation activities; strengthening basic emergency obstetric care within dispensaries, where most deliveries occurred; targeting interventions to improve neonatal outcomes; and introducing birth companionship. Advocacy to ensure sustainability beyond Program tenure was incorporated in later years.

**Results**

The Program achieved significant improvements in maternal and perinatal health indicators, meeting or exceeding targets established by the GoT. By mid-2019, the Program had fully transitioned to GoT oversight.

**Conclusions**

Decentralizing high-quality obstetric, reproductive, and newborn health care is feasible and effective in under-resourced rural settings. Key lessons include:

- The necessity of multistakeholder partnerships. A host of demand- and supply-side interventions are required to meaningfully address maternal and neonatal mortality—leveraging the comparative strengths of a diverse set of stakeholders working in close collaboration will make success more likely.
- Program evaluation should be viewed not only as a tool to assess impact of interventions, but to inform program design. This approach requires flexibility on the part of donors to adapt the program’s design and a willingness to stay the course and invest longer-term.
- The presence of health services does not automatically translate to high-quality and accessible services. Quality of care can be furthered by a supportive work environment, including ongoing mentorship of health care providers. Access can be strengthened by addressing the barriers women face in reaching a health facility and ensuring they are treated respectfully.
- Sustainability cannot be an afterthought. Long-term sustainability requires centering government plans and priorities; strengthening the existing public health care system; and advocacy to ensures prioritization and funding.

## F8.1. Implementing Maternal and Perinatal Death Surveillance and Response (MPDSR): new solutions and opportunities

### Natasha Sobers^1^, Francesca Palestra^2^, Sylvia Deganus^2^, Mary Kinney^3^, Gloria Mutimbwa Siseho^3^

#### ^1^Pan American Health Organization; ^2^World Health Organization; ^3^University of the Western Cape, South Africa

*BMC Proceedings 2024*, **18(5):**F8.1

Submission ID #: IMNHC431

Panel ID# (if applicable): DITJK34431

**Panel Description**

The World Health Organization (WHO) and partners in the MPDSR technical working group are working together to strengthen implementation of MPDSR to enhance the quality of maternal and perinatal care and to improve health outcomes.

Our panel showcases practical solutions and innovative approaches to tackle specific challenges in documenting contributory factors leading to the deaths of women and their babies at facility, national, and regional levels in MPDSR implementation. Experiences will draw from the African and Latin American Caribbean region on health system’s quality improvements.

Our session includes contributions from the audience on what more the WHO, partners, and policymakers can do to ensure further improvement and implementation of the MPDSR cycle at country and global levels.

## F8.2. Monitoring timing and drivers of newborn deaths: the role of maternal and perinatal death surveillance and response intervention in reducing perinatal deaths, Northeast Namibia

### Gloria Mutimbwa Siseho^1^, Thubelihle Mathole^1^, Debra Jackson^1,2^

#### ^1^University of the Western Cape, South Africa; ^2^London School of Hygiene and Tropical Medicine, London

*BMC Proceedings 2024*, **18(5):**F8.2

Submission ID #: IMNHC436

Panel ID# (if applicable): DITJK34431

**Background**

Early newborn deaths (0–7 days) are driving a large proportion of perinatal deaths within the newborn period (0–28days) in Namibia. This study assessed the timing (when?) and drivers (what?) of newborn deaths and describes the maternal perinatal death surveillance and response (MPDSR) intervention system aimed to reduce maternal and newborn deaths.

**Methods**

A quantitative descriptive study design was applied. The intermediate hospital in northeast Namibia was purposively sampled as the only regional hospital in the area. An MPDSR review Excel performance monitoring tool with automated formulas was used to capture and analyze data from the hospital health information system. Analyzed data included deliveries, live births, maternal, perinatal, and newborn deaths from January 2019 to June 2020.

**Results**

Within the perinatal mortality rate of 32/1,000 total births, neonatal deaths within the first week (0–7 days) was the majority (80%). Stillbirths were 58% antepartum and 42% intrapartum, respectively. After completion of the MPDSR skills workshops, 20 recommendations were developed from June 2019 to June 2020. At the time of data analysis, 65% (13 of 20) were completed, 15% (three of 20) were in progress and 20% (four of 20) were delayed. Of the recommendations, 60% (12 of 20) related to reducing perinatal deaths. In the first six months of the MPDSR program, the perinatal mortality rate increased (27 to 35/1,000) but maternal mortality decreased (366 to 139/10,000). For the perinatal deaths, this finding suggests that the surveillance and reporting of stillborn and newborn deaths increased after the MPDSR skills workshop.

**Conclusions**

This is the first study in northeast Namibia to document implications of a functional MPDSR approach in reducing newborn deaths. The first week of life is when a majority of newborn deaths occur. Unless quality of care is improved, and evidence- based strategic interventions implemented during labor, childbirth and immediate postnatal care period, preventable perinatal deaths will not decrease. Improved knowledge and skills by the MPDSR team on what, how, and why capture certain variables, calculation of proportions, ratios and rates played a crucial role in data quality improvement. Results confirm that skills building before introducing or strengthening the MPDSR system is crucial.

## F8.3. From pre-implementation to institutionalization: lessons from sustaining a perinatal audit program in South Africa

### Natasha Rhoda^1^, Francesca Palestra^2^, Sylvia Deganus^2^, Mary Kinney^3^, Anne-Marie Bergh^4^, Robert Pattinson^4^, Asha S. George^3^

#### ^1^University of Cape Town; ^2^World Health Organization; ^3^University of the Western Cape, South Africa; ^4^University of Pretoria

*BMC Proceedings 2024,* **18(5):**F8.3

Submission ID #: IMNHC435

Panel ID# (if applicable): DITJK34431

**Background**

Understanding implementation of maternal and perinatal death surveillance and response (MPDSR) is critical for scaling up and strengthening the intervention process. Few countries report robust operational systems at scale in Africa. One exception is South Africa, which has been implementing a perinatal audit program, a form of MPDSR, to capture perinatal mortality, identify modifiable factors, and motivate change through a quality-of-care audit cycle. This study explores the history of initiating, scaling up, and institutionalizing this program in South Africa.

**Methods**

Data collection was undertaken from 2019 to 2020 involving 56 individual interviews with key stakeholders at national, subnational, and facility levels, a desk review, and 10 non-participant observations of meetings related to the perinatal audit program. Data analysis included thematic content analysis of transcripts, content analysis of relevant document, and process mapping. Lessons learned from nearly three decades of implementation were organized by phase and through use of the health policy analysis triangle framework.

**Results**

Multiple factors influence institutionalization of an audit program, including tangible factors, such as focal points, policies and tools, as well as the societal and systems factors, such as actor interactions and motivations, political priority, and adaption. For South Africa, the national and subnational structures evolved over time and interacted to support implementation benefiting from a continuity of actors, who were able to expand and nurture the network. The perinatal audit program was integrated into national policy and guidelines until recently. Intentional and continuous efforts to demonstrate the impact and enable local adaption allowed for more ownership and buy-in during all phases of implementation. Stagnating mortality reduction along with declining coverage of deliveries reported through the program in recent year’s signaled that backsliding is possible without continuous effort to support implementation.

**Conclusions**

Lessons from South Africa’s experience of implementing a perinatal audit program reveal enabling factors, such as core structures, continuous buy-in and policy integration, as well as demonstrates the vulnerabilities to sustainability. To monitor MPDSR uptake and sustainability, we need research approaches that allow exploration of context, local adaption, and underlying issues that support sustainability.

## F8.4. Understanding factors affecting implementation of perinatal death reviews in Latin America and the Caribbean: convergent mixed methods study

### Natasha Rhoda^1^, Francesca Palestra^2^, Sylvia Deganus^2^, Selvi M Jeyaseelan^3^, Pablo Duran^4^

#### ^1^University of Cape Town; ^2^World Health Organization; ^3^University of the West Indies, Barbados; ^4^Pan American Health Organization

*BMC Proceedings 2024*, **18(5):**F8.4

Submission ID #: IMNHC434

Panel ID# (if applicable): DITJK34431

**Background**

Tracking progress toward the Sustainable Development Goals requires timely, high-quality monitoring of data on maternal and newborn health (MNH). Studying perinatal death surveillance and response (PDSR) implementation can lead to continuous quality improvement and greater accountability in MNH. A recent scoping review of PDSR studies found that the use of implementation frameworks leads to more insightful findings that can better guide policy and health systems recommendations. In this paper, we describe country experiences of PDSR in Latin America and the Caribbean (LAC) region using the consolidated framework for implementation research (CFIR) and assess the barriers and facilitators in implementing regular perinatal death reviews in low- and middle-income countries and small island developing states.

**Methods**

We conducted a desk review of previous Caribbean-based maternal and perinatal death surveillance and response reviews, followed by a sequential mixed methods design in an effort to build a comprehensive assessment of the factors influencing PDSR implementation. We used the CFIR deterministic framework to promote consistent organization of findings and systematic analysis, which can inform policy and practice of MNH enhancement programs in other contexts. We developed the coding framework based on the select constructs and examined the audit review reports using a content analysis.

**Results**

In LAC, 22 countries have completed PDSR activities in the past five years. The desk review revealed that the inner setting (local clinics and hospitals) and outer settings (regional organizations and nongovernmental organizations) worked cohesively to implement infrastructural improvements and human resource capacity-building needed to enhance maternal and perinatal quality of care. Their interwoven structure allowed the completion of comprehensive audit reviews to assess maternal and perinatal deaths and near-miss cases. Competing priorities of the providers involved in MNH care, insufficient local human and technical resources, and the persistence of paper-based records were identified as major barriers to routine implementation of PDSR. Regional organizations were critical in the provision of technical and financial resources to facilitate PDSR processes.

**Conclusions**

Lowering perinatal mortality rates is highly dependent on the ability of health systems to learn from positive and negative outcomes through audits such as PDSR.

## F8.5. Analysis of National Maternal and Perinatal Death Surveillance and Response (MPDSR) reports from Sub-Saharan Africa: strengths and challenges

### Sylvia Deganus, Francesca Palestra, Triphonie Nkurunziza, Assumpta W Muriithi, Leopold Ouedraogo, Chilanga Asmani, Hayfa Elamin, Pamela Amaka Onyiah

#### World Health Organization

*BMC Proceedings 2024*, **18(5):**F8.5

Submission ID #: IMNHC432

Panel ID# (if applicable): DITJK34431

**Background**

The World Health Organization(WHO) and partners have promoted MPDSR as one of the key measures for accelerating progress toward reducing high rates of preventable maternal and newborn deaths. Countries in the African region have adopted the strategy and are at various stages of implementation. Surveys done have indicated that there are numerous challenges to overcome to maximize the benefits of MPDSR. Analyzing national MPDSR reports provides an opportunity to examine the successes, weaknesses, and threats to MPDSR in the region.

**Methods**

A desk review was conducted on readily available national MPDSR reports (2016–2022) that countries voluntarily shared with the WHO regional office. The review process focused on how countries implemented and reported on the outcomes related to the various MPDSR audit steps; the comprehensiveness and depths of analysis of findings; and program performance monitoring, and evaluation.

**Results**

A total of 23 national MPDSR reports were obtained and reviewed; about 50% of all potentially available reports in the region. The majority (56.5%) were from Eastern and Southern Africa. Countries in this these regions appeared to have achieved greater institutionalization of MPDSR systems compared to their Western and Central African counterparts. The release of national reports ranged in frequency from one to three per year. Five out of 23 national reports did not include data on perinatal deaths. Reports on PDSR were generally weaker in content compared to MDSR. The extent and depths of data analysis varied greatly. A notable observation was the finding that countries implementing the confidential enquiry systems had greater in-depth analysis of the contributory factors and risk conditions for the deaths. Some performance assessment of the country’s MPDSR program was included in 91% of reports.

**Conclusions**

The reviewed reports show that substantial progress has been achieved in the implementation of MPDSR in sub-Saharan Africa, particularly within the Eastern and Southern African zones, as compared to their Western and Central African counterparts. Stronger MPDSR performance was observed in countries implementing confidential enquiry approaches. Numerous opportunities exist for countries within the region to share best practices and support each other to strengthen country MPDSR.

## F9.1. Key Lessons from exemplars in Neonatal Mortality Rate/Maternal Mortality Ratio (NMR/MMR) and family planning: positive outliers in neonatal and maternal health and family planning

### Ryan Fitzgerald^1^, Asha S. George^2^, Oona Campbell^3^, Sylvain Landry Birane Faye^4^, Sudha Sharma^5^

#### ^1^Gates Ventures; ^2^University of the Western Cape, South Africa; ^3^London School of Hygiene & Tropical Medicine; ^4^Cheikh Anta Diop University; ^5^CIWEC Hospital and Travel Medical Center

*BMC Proceedings 2024*, **18(5):**F9.1

Submission ID #: IMNHC1674

Panel ID# (if applicable): DKZAX101674

**Panel Description**

This panel, led by in-country research partners, highlights key lessons emerging across nine countries that have been identified as positive outliers on the topics of maternal health, neonatal health, and family planning (FP). The three panelists focus on research findings from Bangladesh, Ethiopia, India, Kenya, Malawi, Morocco, Nepal, Niger, and Senegal, specifically spotlighting factors that have contributed toward progress in these countries. The presentations covers:

1. Interrelated distal, intermediate, and proximal drivers that contribute toward declines in neonatal and maternal mortality in Exemplar countries. Key drivers typically contribute toward progress in a stepwise manner as countries progress through a transition from higher mortality to lower mortality. Lessons from Exemplars can provide useful insights for peer countries looking to emulate the successes of Exemplar countries.
2. FP in low- and middle-income countries (LMICs) has generally made substantial progress in recent decades with select LMICs having demonstrated exemplary progress. This analysis considers drivers of change in FP within specific geographies, alongside a global analysis of drivers for FP. In tandem, this work contributes valuable insights from positive outliers that can be useful for other countries looking to achieve the third Sustainable Development Goal related to healthy lives and well-being.
3. Voluntary FP empowers people to freely decide whether and when to have children and is also a key upstream contributor to improved health outcomes for mothers and children. Lessons from Exemplars demonstrate the importance of empowering women and improving access to quality health care services along the continuum of reproductive health.

## F9.2. Family planning: a key driver of neonatal and maternal mortality reduction in select countries demonstrating exemplary progress

### Jordan-Tate Thomas^1^, Ryan Fitzgerald^1^, Asha S. George^2^, Oona Campbell^3^, Ties Boerma^4^, Sudha Sharma^5^, Agbessi Amouzou^6^, Allisyn Moran^7^, Ira Mrtopullo^1^, Uzma Syed^7^

#### ^1^Gates Ventures; ^2^University of the Western Cape, South Africa; ^3^London School of Hygiene & Tropical Medicine; ^4^University of Manitoba; ^5^CIWEC Hospital and Travel Medical Center; ^6^Johns Hopkins Bloomberg School of Public Health; ^7^World Health Organization

*BMC Proceedings 2024*, **18(5):**F9.2

Submission ID #: IMNHC1702

Panel ID# (if applicable): DKZAX101674

**Background**

Neonatal and maternal mortality are both valuable indicators that have historically been considered markers of health system strength. Although there have been substantial improvements in both the newborn mortality rate (NMR) and maternal mortality ratio (MMR) globally, certain countries have made particularly notable progress. This analysis—part of the Exemplars in Global Health program—presents findings related to neonatal and maternal health advancements in low- and middle-income countries over the last two decades. In particular, this novel analysis on positive outlier countries helps to expand the global evidence base showing that voluntary family planning helps to avert neonatal and maternal deaths.

**Methods**

To identify countries that achieved exemplary progress in improving neonatal and maternal mortality, we compared observed progress to advancements that would have been expected based on change in gross national income. Seven countries were chosen based on reductions of NMR and MMR, data availability, geographical representativeness, and existing partnerships. Once identified, in-depth mixed-methods research was conducted in each country to assess key contributors to NMR and MMR progress.

**Results**

Several themes emerged as key contributors to neonatal and maternal health progress across Exemplar countries. Improvements in access to care during pregnancy and childbirth drove substantial portions of the NMR and MMR reductions. The most important upstream driver identified was fertility decline. In Exemplar countries with particularly sharp fertility declines, such as Bangladesh, decreases in fertility were found to respectively contribute toward 44% and 47% of MMR and NMR reductions. Access to modern contraception, female education, delayed age at first marriage, declining NMR itself, and broadly evolving views about the role of women in society all influenced fertility rates. These results can be synthesized with emerging findings from Exemplars in Family Planning to highlight approaches to increasing modern contraceptive prevalence.

**Conclusions**

Interrelated distal, intermediate, and proximal drivers contribute toward declines in neonatal and maternal mortality in Exemplar countries. Voluntary family planning empowers people to freely decide whether and when to have children and is also a key upstream contributor to improved health outcomes for mothers and children. Lessons from Exemplars demonstrate the importance of empowering women and improving access to quality health care services along the continuum of reproductive health.

## F9.3. Key findings from exemplars in neonatal and maternal mortality

### Ryan Fitzgerald^1^, Ties Boerma^2^, Oona Campbell^3^, Sylvain Landry Birane Faye^4^, Sudha Sharma^5^, Agbessi Amouzou^6^, Allisyn Moran^7^, Jordan-Tate Thomas^1^

#### ^1^Gates Ventures; ^2^University of Manitoba, South Africa; ^3^London School of Hygiene & Tropical Medicine; ^4^Cheikh Anta Diop University; ^5^CIWEC Hospital and Travel Medical Center; ^6^Johns Hopkins Bloomberg School of Public Health; ^7^World Health Organization

*BMC Proceedings 2024*, **18(5):**F9.3

Submission ID #: IMNHC1701

Panel ID# (if applicable): DKZAX101674

**Background**

Neonatal and maternal mortality are both valuable indicators that have historically been considered markers of health system strength. Although there have been substantial improvements in the neonatal mortality rate (NMR) and maternal mortality ratio (MMR) globally, certain countries have made particularly notable progress. This analysis—part of the Exemplars in Global Health program—presents findings on neonatal and maternal health advancements in low- and middle-income countries over the last two decades.

**Methods**

To identify countries that achieved exemplary progress in improving neonatal and maternal mortality, we compared the rate of change in NMR/MMR to change in the gross national income to identify countries that performed exceptionally relative to their economic progress. Seven countries were chosen based on reductions of NMR and MMR, data availability, geographical representativeness, and existing partnerships. Once identified, in-depth mixed-methods research was conducted in each country to assess key contributors to progress, utilizing a mortality transition framework that includes five stages of mortality levels.

**Results**

Several themes emerged as key contributors to neonatal and maternal health progress across Exemplar countries. These contributors were identified to occur in successive stages along the transition from high-mortality to low-mortality states. Fertility decline was a critical driver of progress from high-mortality stage I to stage II, and was associated with higher rates of contraception use. Increased contact with key services such as antenatal care, institutional delivery, cesarean-section, and postnatal care were subsequently linked to further progress along the transition as countries entered stage III. Finally, improvements in equity gaps were associated with progress to the most advanced stages of the transition as quality interventions became available to poorer and more rural communities in stage IV. Together, Exemplar countries at distinct stages of this transition illustrate a stepwise path toward improved neonatal and maternal health outcomes.

**Conclusions**

New research shows that interrelated distal, intermediate, and proximal drivers contribute toward declines in neonatal and maternal mortality in Exemplar countries. The trajectory and drivers of change were not universal, but certain themes consistently emerged as contributors to success. Lessons from Exemplars in neonatal and maternal health provide useful insight for peer countries looking to emulate the successes that Exemplar countries have achieved.

## F9.4. What are we learning about exemplary progress and practices in family planning?

### Ira Mrtopullo^1^, Jordan-Tate Thomas^1^, Ryan Fitzgerald^1^, Allisyn Moran^2^, Sylvia Landry Birane Faye^3^

#### ^1^Gates Ventures; ^2^World Health Organization; ^3^Cheikh Anta Diop University

*BMC Proceedings 2024*, **18(5):**F9.4

Submission ID #: IMNHC1699

Panel ID# (if applicable): DKZAX101674

**Background**

There has been significant progress in family planning (FP) access and utilization in recent decades but it is not equitable across countries and regions. This Exemplars in Global Health analysis presents findings on FP advancements in low- and middle-income countries (LMICs) over the last decade, including linkages with reductions in maternal and neonatal mortality.

**Methods**

To select Exemplar countries in FP, we compared observed progress in modern contraceptive prevalence (mCPR) and demand satisfied for modern contraception to expected advancements based on improvements in the Human Development Index (HDI) from 2010–2020 for 130 LMICs. Six positive outlier countries were identified, including one of the Exemplars in maternal mortality ratio (MMR)/neonatal mortality rate (NMR), accounting for regional representation, data availability, population size, and existing partnerships. In-depth mixed-methods research is underway in the first three countries and will begin in three additional countries in 2023 to explain key drivers of improvements. A complementary global Shapley decomposition assessed key drivers of progress in the same FP metrics globally at population level.

**Results**

Positive outliers from multiple models were evaluated as potential Exemplars. The first round of country selection identified Malawi, Kenya, and Senegal; three more countries will be confirmed by end of 2022. In-depth qualitative and quantitative findings from each country are being processed to provide a holistic explanation of exemplary improvement in FP, including the role of high-impact practices in family planning. Senegal is also an Exemplar in MMR/NMR, and we are coordinating to investigate the effects of improvement in FP. The global decomposition found that income and knowledge of methods are important drivers of mCPR and demand satisfied globally; we are exploring these relationships further at the country level.

**Conclusions**

Our research describes FP advancements among LMICs, highlighting lessons learned from Exemplar countries. Considering drivers of progress in FP and NMR/MMR within specific geographies, alongside a global analysis of drivers for FP, can yield actionable insights toward achieving the third Sustainable Development Goal on healthy lives and well-being.

## F10.1. Re-visioning Emergency Obstetric and Newborn Care (EmONC): a revised EmONC framework and indicator set for the next two decades

### Wei Lu^1^, Caitlin Warthin^1^, Lynn Freedman^1^, Isabelle Moreira^1^, Martin Aluvaala^2^, Patience Afulani^3^

#### ^1^Coumbia University Mailman School of Public Health; ^2^University of Nairobi, Kenya, ^3^University of California, San Francisco

*BMC Proceedings 2024*, **18(5):**F10.1

Submission ID #: IMNHC1064

Panel ID# (if applicable): DLQWK681064

**Panel Description**

Over the last 20 years, the emergency obstetric care (EmOC) indicators and framework have been used extensively to plan and monitor EmOC services at national and subnational levels. The current framework and indicators have provided critical information to help develop data-driven approaches to reducing maternal mortality and set targets and track progress toward strengthening EmOC services. At the same time, health systems have evolved, the evidence base has expanded, and implementation lessons have been collected about what works and what remains challenging across different settings. With these developments in mind, a global initiative led by Columbia University’s Averting Maternal Death and Disability program, the London School of Hygiene and Tropical Medicine, UNFPA, UNICEF, and WHO is updating, improving, and expanding the EmOC framework and indicators to better meet the needs of countries for the next 20 years. This process has been designed using principles and practices of human centered design to ensure that the indicators meet the needs of key users, particularly national and subnational planners and managers. This includes learning from countries’ experiences using the EmONC framework and indicators and their ideas for improvement. A global modified Delphi study and in-depth studies in Senegal, Malawi, and Bangladesh using human-centered design methods generated insights on what is needed and wanted for planning and monitoring implementation. These findings were integrated into the Re-visioning process. This panel presents the near final draft of the revised EmONC framework and indicators and provides an opportunity for participants to share feedback.

## F10.2. Re-visioning new indicators that address current and future needs

### Patience Afulani^1^, Caitlin Warthin^2^

#### ^1^University of California, San Francisco; ^2^Coumbia University Mailman School of Public Health

*BMC Proceedings 2024*, **18(5):**F10.2

Submission ID #: IMNHC1095

Panel ID# (if applicable): DLQWK681064

**Background**

The *Monitoring emergency obstetric care: A Handbook* laid out a conceptual framework and eight indicators that when used together help inform national and subnational planning for maternal and newborn health (MNH) programs and assess progress in the reduction of maternal and newborn mortality. Yet, the MNH field has changed over the last 30 years, and as needs evolve, so should the indicators that we use.

**Methods**

The data for the handbook indicators were collected through health facility surveys that ranged from national censuses to smaller efforts, using standardized but adaptable modular questionnaires. To make the process more sustainable and nimble, the Re-Visioning process will propose a core set of indicators fully integrated into routine health management and information systems. A supplementary set of elaborated indicators will accompany the core to provide more detail and depth, but these may require additional data collection efforts.

**Results**

The Re-Visioning framework is organized around achieving: 1) an equitably distributed and accessible supply of well-functioning facilities that provide emergency obstetric and newborn care (EmONC); 2) a responsive and people-centered approach around those who use the system as well as the health workers who make the system work; and 3) the (contextual) effectiveness of EmONC. Proposed indicators for #1 address whether enough facilities provide EmONC, if they are well equipped and staffed, if they function as a network, and are well-linked across levels of care and equitably distributed geographically. Indicators addressing #2 determine where women are accessing care, whether users experience respectful care, and if health workers also experience respect and support in the workplace. Finally, under #3 we learn whether the care provided was appropriate, if women and newborns with complications receive the clinical care they need, and whether the EmONC system is addressing the key causes of maternal and newborn death.

**Conclusions**

The revised set of indicators reflects global advances in integrating MNH. In addition to fully incorporating the problems of small and sick newborns, the set highlights the care that all women and their newborns should receive, in addition to the well-being of the workforce in the health system.

## F10.3. New signal functions and levels of care to define Emergency Obstetric and Newborn Care (EmONC): integrating obstetric and small and sick newborn care

### Martin Aluvaala^1^; Caitlin Warthin^2^

#### ^1^University of Nairobi, Kenya; ^2^Coumbia University Mailman School of Public Health

*BMC Proceedings 2024*, **18(5):**F10.3

Submission ID #: IMNHC1084

Panel ID# (if applicable): DLQWK681064

**Background**

The Re-Visioning EmONC project seeks to review, rethink, and revise the EmONC framework and indicators. The existing framework is built around signal functions and designating basic and comprehensive levels of care. A central charge for Re-Visioning EmONC was to select which signal functions should be included in the revised framework, and how they should be organized into levels of care. A commitment to integrating obstetric and small and sick newborn care guided this undertaking.

**Methods**

Two working groups tackled defining signal functions and levels care, each co-led by a maternal health expert and newborn health expert. To facilitate a true “rethink” of the EmONC framework, the working groups conducted literature reviews on how signal functions have been used across disciplines; how levels of care have been defined/conceptualized, operationalized/implemented, and measured; and how referral and bypassing affect maternal and newborn health (MNH) care-seeking and outcomes. They conducted a global modified Delphi study to build consensus on MNH signal functions and levels of care. These inputs were fed back into the working groups, a separate newborn expert group, and the project’s Steering Committee to define the final framework.

**Results**

The criteria for selecting signal functions for the revised EmONC framework were: clinical interventions that are critical, frequent, tracers, and simple. The Delphi study included 212 respondents in round 1, 131 in round 2, and # in round 3, and identified prioritized emergency obstetric and small and sick newborn signal functions. The levels of care scoping review yielded 162 relevant reports, with facility-based MNH care most frequently described in three levels. The revised EmONC framework arranges MNH signal functions across levels of care as follows: Level 1 (signal functions x, y, z), Level 2 (signal functions x, y, z), etc. [Data forthcoming.]

**Conclusions**

The EmONC framework has been enormously influential over the last 30 years. We hope that the revised framework, which now integrates obstetric and small and sick newborn care, will continue to form the basis for planning around human resources, infrastructure, equipment, supplies, transport, and referral related to care during childbirth for decades to come.

## F10.4. Revising the Emergency Obstetric and Newborn Care (EmONC) indicator set to meet the needs of country-level users

### Isabelle Moreira, Caitlin Warthin

#### Coumbia University Mailman School of Public Health

*BMC Proceedings 2024*, **18(5):**F10.4

Submission ID #: IMNHC1074

Panel ID# (if applicable): DLQWK681064

**Background**

The EmONC framework and indicators have been used by government officials, health care providers, and other users in 80+ countries to improve the availability, use, and quality of EmONC. With decades of experience using the framework and indicators, it’s essential to ask what planners, implementers, and evaluators in countries want to change about the approach to enhance planning processes and highlight areas requiring priority actions.

**Methods**

The Re-Visioning EmONC initiative conducted studies using human-centered design techniques in Bangladesh, Malawi, and Senegal to learn about country-level users’ experiences with the framework and indicators and what revisions might better meet their needs. During focus group discussions and key informant interviews, government planners, health care providers, communities, and other key user groups explored challenges in providing and accessing good-quality EmONC and were asked what information they need for planning and monitoring that aren’t part of the current indicators. Participants were also presented iterative prototypes of the new framework and indicators for feedback.

**Results**

Initial findings show the indicators have been useful for planning and monitoring changes over time. However, most user types requested indicators flagging key system support failures such as insufficient staffing and stock-outs of key drugs/supplies, and indicators on patients’ experience of care, teamwork, provider job satisfaction and referral. Some providers found the current process of assessing EmONC status dispiriting since it has been challenging to achieve and fails to reflect intense efforts to improve and sustain good-quality EmONC in the face of limited resources. They want the revised set to not only identify problems, but also highlight positive changes in the health system. There were also requests for meaningful integration of maternal and newborn care, including organizing services to better promote collaboration among providers caring for women and newborns. For communities, key elements include how open and welcoming the system is to women and their families.

**Conclusions**

By integrating various user groups’ inputs into the Re-Visioning process, we expect the revised framework and indicators will better meet the needs of country actors and ultimately improve the uptake and utilization of the indicators to improve maternal and newborn well-being.

## F10.5. Re-visioning Emergency Obstetric and Newborn Care (EmONC) overview

### Lynn Freedman, Caitlin Warthin

#### Coumbia University Mailman School of Public Health

*BMC Proceedings 2024*, **18(5):**F10.5

Submission ID #: IMNHC1065

Panel ID# (if applicable): DLQWK681064

**Background**

The 1997 ‘*Guidelines for monitoring the availability and use of obstetric services’* describe a set of indicators to measure and monitor the availability, utilization, and quality of emergency obstetric care in low- and middle-income countries (LMICs). This framework has been enormously influential over the last 30 years. It is found in virtually all LMIC health plans for maternal and newborn health (MNH), and often forms the basis for planning around human resources, infrastructure, equipment, supplies, transport, and referral for care during childbirth. The indicators were updated once in 2009 (*’Monitoring emergency obstetric care: A Handbook’*). The Re-Visioning EmONC project is an initiative to review, rethink, and revise the framework.

**Methods**

The Re-visioning EmONC process must respond to many changes in the health systems and MNH landscape over the last 30 years: increased facility-based childbirth; a push to integrate maternal and newborn care, and to consider continuity of care across the MNH continuum; advances in technology that change what is feasible for data collection and analysis; increased focus on quality of care (clinical and experiential), including more nuanced thinking on quality-of-care measurement; and gender and equity becoming key lenses for health system functionality. The revised framework and indicators must respond to these changes, striking the balance between being comprehensive vs. parsimonious, and aspirational vs. realistic.

**Results**

The revised EmONC framework and indicators provide a robust picture of the facility-based EmONC system and how it functions on essential parameters, including whether care is timely and appropriate, respectful and equitable, and fulfills the rights and expectations of users and providers. It tells us if the system is well-connected via communication, transportation, governance and accountability to the communities it serves. Above all, it provides essential evidence and information to enable planners and managers to develop short-, medium-, and long-term MNH strategies and to solve problems.

**Conclusions**

The revised EmONC framework and indicators will be an important tool to support countries in creating facility-based EmONC systems in which all women and newborns who experience complications access care that gives them the best chance to survive and thrive.

## F11.1. Mother Newborn Care Unit (MNCU) experience in India: a paradigm shift in care of small and sick newborns

### Sugandha Arya^1^, Harish Chellani^2^, Pratima Mittal^3^, Kilangnaro T Nokdy^1^

#### ^1^Safdarjung Hospital; ^2^Center for Health Research and Development: Society for Applied Studies; ^3^Amrita Institute of Medical Sciences

*BMC Proceedings 2024*, **18(5):**F11.1

Submission ID #: IMNHC1535

Panel ID# (if applicable): DMAPY701535

Note the full manuscript was previously published in the Indian Journal of Pediatrics: https://link.springer.com/article/10.1007/s12098-022-04145-9

## F11.2. Mother newborn care unit: nurses’ experience

### Sugandha Arya^1^, Harish Chellani^2^, Pratima Mittal^3^, Kilangnaro T Nokdy^1^, Nitya Wadhwa^4^

#### ^1^Safdarjung Hospital; ^2^Center for Health Research and Development: Society for Applied Studies; ^3^Amrita Institute of Medical Sciences; ^4^Translational Health Science and Technology Institute

*BMC Proceedings 2024*, **18(5):**F11.2

Submission ID #: IMNHC1549

Panel ID# (if applicable): DMAPY701535

**Background**

Mother Newborn Care Unit (MNCU) is a Neonatal Intensive Care Unit (NICU) where the mother and baby dyad are cared for together, with active engagement of the mother. It provides facilities for mothers to stay with their preterm neonates 24 hours per day. The unit is equipped with central oxygen supply, suction machines, radiant warmers, continuous positive airway pressure (CPAP) machines and monitors (level II care facility). In the MNCU, neonatal nurses provide care to both small sick newborns and also postnatal care to mothers. We present here nurses’ experiences of working in the MNCU.

**Methods**

Nurses were trained in newborn care including neonatal resuscitation, assisted feeding, IV access, immediate and continuous provision of kangaroo mother care, monitoring of any sickness, oxygen saturation monitoring, and provision of respiratory support. They were trained to provide CPAP, provide tube feeding, give intravenous injections, check blood sugar through a dextrostix test, and monitor newborn’s heart rate and oxygen saturation in the kangaroo mother care position.

Nurses were also trained to provide maternal postnatal care including immediate monitoring for pallor, heart rate, blood pressure, bleeding p/v, breast conditions, providing oral/IV antibiotics/ supplements (iron, calcium), and assisting with episiotomy/perineal care.

**Results**

Nurses have experienced that mothers in the MNCU can be easily trained to follow asepsis routines, monitor the neonates, and be better prepared for post-discharge care of neonates. Mothers get more support for breastfeeding and expression of breast milk. There is less anxiety and stress among mothers by being with their baby in the MNCU all the time. Staff has less workload due to the mother’s presence as a care provider in the MNCU. Nurses have perceived better weight gain among babies cared for by their mother with zero separation.

**Conclusions**

There is positive feedback from nurses working in the MNCU that provides a platform to deliver holistic respectful care to both mothers and newborns.

## F11.3. Mother and newborn care unit: obstetrician perspective

### Sugandha Arya^1^, Harish Chellani^2^, Pratima Mittal^3^

#### ^1^Safdarjung Hospital; ^2^Center for Health Research and Development: Society for Applied Studies; ^3^Amrita Institute of Medical Sciences

*BMC Proceedings 2024*, **18(5):**F11.3

Submission ID #: IMNHC1544

Panel ID# (if applicable): DMAPY701535

**Background**

Robust evidence is available to support immediate kangaroo mother care (iKMC) as tool of zero separation of the mother and neonate from birth until discharge. From the obstetrician’s perspective, there are challenges in operationalizing iKMC. These include initiating KMC in the labor room, ensuring transportation in the KMC position, creating a mother-and-baby-friendly infrastructure in the neonatal intensive care unit (NICU) known as Mother Newborn Care Unit (MNCU)and ensuring coordination with the pediatrics team for providing postnatal care to mothers in the NICU.

**Methods**

The challenge of initiating KMC in the delivery room was addressed by having a surrogate next to the delivery room, convincing the family to shift the baby to the KMC position, and providing a supply of KMC garments with binders for safe and effective KMC in the labor room and during transport.

In the MNCU, infrastructure for the care of mothers includes an examination cubicle, washroom, bathing room, and facility to arrange for assisted feeding. Caring for mothers in the MNCU is a major challenge, given that these mothers have just delivered. An essential care package for mothers has been developed for immediate postnatal care, in which neonatal nurses are trained. An obstetrics team also visits to see and care for the mothers in the MNCU.

**Results**

With the involvement of obstetricians, the MNCU has emerged as a facility where small and sick newborns are cared for with their mothers 24 hours a day with all level II facilities providing newborn care and postnatal care to mothers. Facility is running successfully for last 5 years even in covid period.

**Conclusions:** Strong cooperation, coordination, and collaboration between pediatricians and obstetricians is the cornerstone of the MNCU to provide care to small and sick babies.

## F11.4. Mother newborn care unit: evidence to practice

### Sugandha Arya^1^, Harish Chellani^2^

#### ^1^Safdarjung Hospital; ^2^Center for Health Research and Development: Society for Applied Studies

*BMC Proceedings 2024*, **18(5):**F11.4

Submission ID #: IMNHC1541

Panel ID# (if applicable): DMAPY701535

**Background**

A multicountry, randomized, controlled trial coordinated by the World Health Organization (WHO) was conducted in five hospitals in Ghana, India, Malawi, Nigeria, and Tanzania to study effects of immediate kangaroo mother care (iKMC) on neonatal outcome. The study results show that intervention babies in the mother newborn care unit (MNCU) had 25% less mortality at 28 days of life, 35% less incidence of hypothermia, and 18% less suspected sepsis as compared to control babies cared in the conventional neonatal intensive care unit (NICU). Implementation of this trial led to the development of the Mother Newborn Care Unit (MNCU), a facility where small and sick newborns are cared for with their mothers 24 hours a day at all level II facilities providing newborn care. The question has been raised that in order to replicate this concept, what infrastructure or policy changes would be required.

**Methods**

After completion of the study, the MNCU facility has been continuing at Safdarjung Hospital, New Delhi, based on the evidence of substantial improvements in outcomes. This required system changes in the infrastructure and processes. Certain policy changes were also needed, such as allowing mothers/surrogates in the MNCU (same as that for family-centered care), shifting small babies from delivery areas to MNCU in the KMC position, and providing postnatal care to mothers in the MNCU by neonatal nurses and obstetricians.

**Results**

The MNCU at Safdarjung Hospital is a 12-bed level II NICU, where babies weighing less than 1,800 g are admitted and the concept of zero separation between mother and baby has been continued since January 2020 after completion of the study. The mother is not a mere visitor, but as a resident of MNCU, she becomes an active caregiver (changing diapers, feeding the baby, and monitoring oxygen saturation, thus providing developmentally supportive care.

**Conclusions**

There is enough evidence that the MNCU improves newborn outcomes. To bring this to practice, we need system changes including reorganizing level II NICUs to MNCUs, changing policy to allow mothers/surrogates in the MNCU, and collaborating with obstetricians to give postnatal care to mothers in the MNCU.

## F12.1. World Health Organization (WHO) research to Improve survival of small and sick newborns: impact and implications for program

### Suman Rao^1^, Anshu Banerjee^1^, Helga Naburi^2^, Ayesha De Costa^1^, Yasir Bin Nisar^1^, Rajiv Bahl^1^, Robinson Wammanda^3^

#### ^1^World Health Organization; ^2^Muhimbili University of Health and Allied Sciences; ^3^Ahmadu Bello University Teaching Hospital, Zaria

*BMC Proceedings 2024*, **18(5):**F12.1

Submission ID #: IMNHC863

Panel ID# (if applicable): DMCHF35863

**Panel Description**

The WHO has been supporting multicountry research to support newborns to survive and thrive. This panel discussion brings together critical new evidence for the survival of newborns. Four large WHO randomized controlled trials, some of which have been published recently have led to new ongoing implementation research. The aim of the panel is to make the participants aware of the research and to discuss the implications of the results for country-level program and scale-up.

## F12.2. Health, nutrition, psychosocial support, and water, sanitation, and hygiene interventions delivered during preconception, pregnancy, and early childhood periods on birth outcomes and on linear growth at 24 months of age

### Rajiv Bahl^1^, Suman Rao^1^, Helga Naburi^2^, Harish Chellani^3^

#### ^1^World Health Organization; ^2^Muhimbili University of Health and Allied Sciences; ^3^Center for Health Research and Development: Society for Applied Studies

*BMC Proceedings 2024*, **18(5):**F12.2

Submission ID #: IMNHC881

Panel ID# (if applicable): DMCHF35863

Note the full manuscript published in September 2022 in *BMJ*:

(https://www.bmj.com/content/379/bmj-2022-072046.long)

## F12.3. Improving management of Possible Serious Bacterial Infection (PSBI) in newborns in Low- and Middle-Income Countries (LMICs): evidence into policy and clinical practice

### Robinson Wammanda^1^, Suman Rao^2^, Helga Naburi^3^, Yasir Bin Nisar^2^, Shabina Ariff^4^

#### ^1^Ahmadu Bello University Teaching Hospital; ^2^World Health Organization, ^3^Muhimbili University of Health and Allied Sciences, Zaria, ^4^The Aga Khan University

*BMC Proceedings 2024*, **18(5):**F12.3

Submission ID #: IMNHC879

Panel ID# (if applicable): DMCHF35863

Note the full manuscript published in June 2022 in *PLOS ONE*:

(https://journals.plos.org/plosone/article?id=10.1371/journal.pone.0269524)

## F12.4. Improving the survival of low birthweight infants in Low- and Middle-Income Countries (LMICs): expanding the scope, reach, and impact of kangaroo mother care

### Shabina Ariff^1^, Suman Rao^2^, Helga Naburi^3^, Ayesha De Costa^2^

#### ^1^The Aga Khan University; ^2^World Health Organization; ^3^Muhimbili University of Health and Allied Sciences, Zaria

*BMC Proceedings 2024*, **18(5):**F12.4

Submission ID #: IMNHC876

Panel ID# (if applicable): DMCHF35863

**Background**

Facility-initiated kangaroo mother care (KMC) for stable low birthweight (LBW) infants is a proven high-impact intervention to reduce mortality. However, many LBW infants die before stabilization and the coverage of KMC is low globally. The World Health Organization (WHO)-led research has proved that initiating immediate KMC soon after birth (iKMC) before stabilization is a promising strategy to improve coverage, in addition to developing context-adapted implementation models. There is a need to understand how to scale up iKMC in LMCIs.

**Methods**

The iKMC trial, a hospital-based randomized controlled trial (RCT) in five countries in Asia and Africa, enrolled infants 1–1.799 kg to iKMC at birth before stabilization or control group. All care in the maternal-neonatal intensive care unit (M-NICU) including respiratory support was provided with the baby in the KMC position. The S-KMC study used mixed methods to develop scalable models to deliver KMC. Currently, theWHO is coordinating the iKMC implementation research (IR) where the initial models based on formative research will be developed in routine care settings and evaluated in a step-wedge trial.

**Results**

In the iKMC trial enrolling 3,211 infants, the median duration of skin-to-skin contact (STS) in the NICU was 16.9 hours/day in the intervention group. Neonatal death was reduced by 25%. In the S-KMC study, the implementation models developed achieved high population-based coverage in the enrolled 3,804 LBW infants < 2,000 g. KMC was initiated in 68%-87% of infants and at discharge, effective KMC (> 8 hours of STS and exclusive breastfeeding) was provided to 53%-68% of infants. The iKMC IR study is recruiting.

**Conclusions**

The iKMC and S-KMC studies have shown how to exponentially improve coverage of KMC and reduce mortality. These studies have led to a revision of LBW guidelines and introduced the new paradigm of M-NICU, which will be evaluated in the iKMC IR.

## F12.5. Improving the survival of preterm infants in Low- and Middle-Income Countries (LMICs): evaluating the role of and expanding access to Antenatal Corticosteroids (ACS) - The World Health Organization (WHO) coordinated ACS research program

### Suman Rao^1^, Helga Naburi^2^

#### ^1^World Health Organization; ^2^Muhimbili University of Health and Allied Sciences, Zaria

*BMC Proceedings 2024*, **18(5):**F12.5

Submission ID #: IMNHC871

Panel ID# (if applicable): DMCHF35863

**Background**

Preterm birth contributes significantly to neonatal mortality. ACS are proven to improve preterm survival in high-income settings and are widely used across the world. However, the 2015 ACT trial in LMICs suggested that ACS results in an increase in neonatal mortality and maternal adverse outcomes. To resolve this ambiguity and inform WHO guidelines, the WHO initiated the ACS research program comprising two multicountry placebo-controlled trials of ACS on neonatal outcomes in the early and late preterm periods (ACTION I, ACTION III), respectively, in LMICs; and the ACS implementation research project (ACS-IR), to establish and test a strategy for the safe and effective use of ACS in LMICs.

**Methods**

ACTION-I and ACTION-III are individually randomized trials of ACS versus placebo on neonatal mortality and morbidity in Bangladesh, India, Kenya, Nigeria, and Pakistan. The ACS-IR project focuses on developing a strategy through iterative cycles to improve safe and effective coverage of ACS.

**Results**

ACTION-I (completed) showed a clear reduction in neonatal mortality (RR 0.84, 95% CI 0.72-0.97) and morbidity (respiratory) in LMICs. No difference in maternal and neonatal adverse outcomes was detected. Baseline work to inform strategy for improved ACS use has been completed in the ACS-IR.

**Conclusions**

Implications for country/program: ACTION 1 has confirmed the effectiveness in LMICs. ACTION III (late preterm) is presently recruiting. ACS-IR will inform strategies to improve access in the country/program. The ACS research program, which this panel discusses, will inform guidelines on preterm birth, thereby promoting newborn survival.

## F13.1. Learning from the past, connecting to the future: insights from seven Maternal and Newborn Health (MNH) exemplar countries using a mortality transition model

### Agbessi Amouzou^1^, Ties Boerma^2^, Oona Campbell^3^, Cheikh Mbacke Faye^4^

#### ^1^Johns Hopkins Bloomberg; ^2^University of Manitoba, South Africa; ^3^London School of Hygiene & Tropical Medicine; ^4^African Population and Health Research Center

*BMC Proceedings 2024*, **18(5):**F13.1

Submission ID #: IMNHC718

Panel ID# (if applicable): DODJJ89718

**Panel Description**

Using results of the MNH Exemplars study conducted during 2020–2022 in seven countries in Africa and Asia (Bangladesh, India, Nepal, Morocco, Ethiopia, Niger, and Senegal), four presentations use a maternal mortality, stillbirth, and neonatal mortality transition model to address the key lessons learned from past progress for today’s programs and tomorrow’s strategies.

“Applying a Maternal and Neonatal Mortality Transition Model to Identify Drivers of Progress in India’s States” describes the transition model and its dimensions—causes of death, fertility, health systems, service delivery strategies, and inequalities—based on extensive analyses for 149 countries, and then applied to the seven MNH exemplar countries, with special attention to India’s subnational progress and present issues.

“Data Gaps in the Understanding of Progress in Maternal and Neonatal Mortality in the Exemplar Countries” synthesizes how major data gaps affect mortality and cause of death tracking and analyses, hampering the ability to assess trends and inequalities. High coverage of health facility births affords new opportunities to collect better data.

“Successful Strategies for Intrapartum Care: What Have Exemplar Countries Done and How Was This Achieved?” presents intrapartum service strategies, which vary considerably between countries. Future strategies can be informed by experiences of countries in more advanced stages of the transition model. High coverage of hospital deliveries with quality care and adequate cesarean section rates among women living in poverty appear to be key.

“Closing the Gap: Trends and Inequalities in Maternal and Newborn Health among Seven Exemplar Countries” analyzes the coverage and trends of inequalities in MNH interventions in the exemplar countries. Inequality patterns in neonatal mortality vary across the transition phases, but intervention coverage disparities decreased almost everywhere. A focus on women and neonates living in under-resourced areas is a critical driver, but strategies vary by transition stage.

The panel will conclude with invited comments from two policymakers on the utility of the transition model approach, followed by a moderated discussion with participants.

## F13.2. Successful strategies for intrapartum care: what have exemplar countries done and how was this achieved?

### Agbessi Amouzou^1^, Ties Boerma^2^, Oona Campbell^3^, Jessica King^4^, William Oswald^4^, Bouchra Assarag^5^, Shams El Arifeen^6^, Youssoufa Lamou^7^

#### ^1^Johns Hopkins Bloomberg; ^2^University of Manitoba, South Africa; ^3^London School of Hygiene & Tropical Medicine; ^4^London School of Hygiene & Tropical Medicine; ^5^École Nationale de Sante Publique Maroc; ^6^International Centre for Diarrhoeal Disease Research; ^7^Institut National de la Statistique

*BMC Proceedings 2024*, **18(5):**F13.2

Submission ID #: IMNHC1015

Panel ID# (if applicable): DODJJ89718

**Background**

Labor, delivery, and the first 24 hours postpartum pose high risks of late fetal, maternal, and neonatal death. Where women deliver, who attends them, and whether they can be managed in situ or need transport are critical drivers of survival. Many countries have adopted strategies to increase skilled birth attendance, primarily by increasing institutional deliveries. We explore the extent to which institutional delivery, facility-level, and cesarean section capability drive success, deriving lessons for current and future intrapartum care strategies.

**Methods**

In the transition model, and for seven exemplar countries (Bangladesh, India, Nepal, Morocco, Ethiopia, Niger, and Senegal), we used national survey data to characterize where childbirth took place. We also examined cesarean section overall and among the poorest quintile.

**Results**

In the transition model, the median level of institutional deliveries increased from 42% to 98% as countries moved from high mortality (phase I) to low mortality (phase V). Wealth-related inequalities in institutional delivery decreased strongly. The transition from phase I to II often involved increases in number of births in lower-level health facilities, while subsequent progress was characterized by rapid increases in hospital births. Median cesarean section rates increased from <1% to 17% among the poorest quintile (phase I to phase IV).

Exemplar countries increased institutional delivery over time, often dramatically (e.g., 9% to 78% in Nepal). Moreover, countries with the highest mortality levels in 2017 (e.g., Niger phase II) had low institutional deliveries (30%), hospital deliveries (0.4%), and cesarean section rates among the poorest quintile (0.5%). The reverse occurred for low mortality countries. Morocco (phase IV) had the highest institutional (86%) and hospital deliveries (57%) and cesarean section among the poorest quintile (13%). Hospital facilities matter because the proportion able to provide comprehensive emergency care was higher than the proportion of lower-level facilities able to provide basic emergency care. With exceptions (e.g., India’s 108 ambulance service), transport and referral systems were poorly characterized.

**Conclusions**

As countries progress through the transition, institutional delivery becomes near universal. While a range of facility types and transport modalities could theoretically result in low mortality, in practice, high coverage of hospital deliveries and adequate cesarean section rates among the poorest quintile

## F13.3. Closing the gap: trends and inequalities in maternal and newborn health among seven exemplar countries

### Cauane Blumenberg^1^, Agbessi Amouzou^2^, Luisa Arroyave^1^, Leonardo Ferreira^1^, Aluísio J. D. Barros^1^

#### ^1^International Center for Equity in Health; ^2^Johns Hopkins Bloomberg School of Public Health

*BMC Proceedings 2024*, **18(5):**F13.3

Submission ID #: IMNHC995

Panel ID# (if applicable): DODJJ89718

**Background**

Some countries have made important progress on maternal and newborn health (MNH), including seven countries that exceeded expectations on maternal and neonatal mortality reduction. Our objective was to analyze the coverage and trends of inequalities in MNH interventions in these exemplar countries.

**Methods**

The analyses included 30 population-based surveys from 2003 to 2021. We analyzed the MNH composite indicator, a score that ranges from 0 to 3 points, scoring 1 for each of the following interventions received: attending four or more antenatal care visits with any provider, institutional delivery, and postnatal care for the woman or baby within two days of delivery. Trends were analyzed calculating the annual average rate of change (AARC). Patterns of inequality were evaluated, and absolute measures of inequality were calculated by area of residence using the urban-rural gap, and by wealth quintiles using the slope index of inequality (SII).

**Results**

The median MNH score in the latest surveys from exemplar countries was 1.85, ranging from 1.07 in Morocco 2003 to 2.26 in Nepal 2019. The median MNH score gap was 0.56 in favor of urban areas, with the widest and narrowest gaps observed in Morocco (0.85 points) and India (0.24 points), respectively. Trend analyses showed that the urban-rural gaps are closing, with the largest reduction observed in India (−9.8% AARC). Regarding wealth quintiles, Bangladesh, Ethiopia, Nepal, and Niger went from a top to a linear pattern of inequality, while India and Senegal changed from a linear to an approximately bottom pattern of inequality. Although MNH scores increased over the years in all wealth quintiles, SII estimates showed that the gaps between the top and bottom of the wealth distribution, comparing the first and latest surveys, decreased only in India (from 2.47 to 0.72), Nepal (1.79 to 1.22), and Niger (1.27 to 0.89).

**Conclusions**

MNH scores increased in all exemplar countries, regardless of their phase on the mortality transition model proposed by Boerma and colleagues in 2023. Inequalities by area of residence and wealth also decreased, however, important gaps are still observed. Policies and interventions focused on those left behind might contribute to further progress of exemplar countries on the mortality transition model.

## F13.4. Applying a maternal and neonatal mortality transition model to identify drivers of progress in India’s States

### Usha Ram^1^, Agbessi Amouzou^2^, Ramesh Manjappa^3^, Himanshu Bhushan^4^, Andrea Blanchard^3^, Kerry Scott^3^, Prakash Kumar^1^

#### ^1^International Institute for Population Sciences; ^2^Johns Hopkins Bloomberg School of Public Health; ^3^University of Manitoba; ^4^National Health Systems Resource Centre

*BMC Proceedings 2024*, **18(5):**F13.4

Submission ID #: IMNHC722

Panel ID# (if applicable): DODJJ89718

**Background**

A five-phase maternal mortality, stillbirth, and neonatal mortality transition model was developed to benchmark the current situation, assess drivers of progress, and inform strategic planning and investments. This study aims to understand the rapid reductions in maternal and neonatal mortality in India, with a focus on subnational progress using the transition model.

**Methods**

India’s states were grouped into one higher and one lower mortality states cluster (HMS and LMS) to examine trends and inequalities since the 1990s in mortality, fertility, service coverage, and health system characteristics. We also assessed progress in two successful states, one in each mortality cluster: Maharashtra and Rajasthan. We analyzed Sample Registration System, National Family Health Survey, and District Level Household and Facility Survey data and collected qualitative data from key informant interviews and round-table discussions with experts (sub)nationally to understand contextual, health policy, and system changes.

**Results**

Maternal and neonatal mortality declined markedly during 1990–2018 in both state clusters, with the HMS having a 12- and 16-year lead, respectively, over the LMS. Indicators of change included fertility decline, antenatal and delivery care coverage, cesarean section rates among women living in poverty, health infrastructure and workforce strengthening, and policies that focus on reducing poverty—all with important differences between the HMS and LMS.

Maharashtra’s major progress was associated with near-universal reach of key interventions among disadvantaged populations, improving quality of care such as antenatal care with contents and reduced neonatal mortality in hospitals, and most women delivering in facilities with comprehensive emergency capacity, including sick newborn care units. Rajasthan’s rapid gains in coverage, including institutional delivery increases in both hospitals and lower-level facilities and cesarean sections among populations living in rural or under-resourced areas, came from much lower baselines. Critical health policy and system reforms could be identified, as well as the first steps toward more advanced maternal and neonatal care.

**Conclusions**

Major improvements in coverage of health services and reductions in inequalities during the past two decades led to major reductions in maternal and neonatal mortality in the Indian states. Reference values for key indicators of change according to phases of the transition allowed further identification of drivers of success and potential strategic priorities for further progress, providing a tool for systematic cross-state exchange and learning.

## F13.5. Data gaps in the understanding of progress in maternal and neonatal mortality in the exemplar countries

### Agbessi Amouzou^1^, Ties Boerma^2^, Oona Campbell^3^

#### ^1^Johns Hopkins Bloomberg; ^2^University of Manitoba, South Africa; ^3^London School of Hygiene & Tropical Medicine

*BMC Proceedings 2024*, **18(5):**F13.5

Submission ID #: IMNHC720

Panel ID# (if applicable): DODJJ89718

**Background**

The maternal and neonatal mortality exemplar study aimed to understand drivers of rapid mortality reduction in the period 2000–2020. Seven countries were identified in South-East Asia, the Eastern Mediterranean, and sub-Saharan Africa based on their exceptional progress. Their selection; analysis of levels, trends, and inequalities in maternal, late fetal, and neonatal mortality; identification of drivers; and conclusions faced major data challenges. We discuss these challenges and lessons for the understanding of current and future mortality trends.

**Methods**

The seven countries are Bangladesh, India, and Nepal in South-East Asia; Morocco in the Eastern Mediterranean; and Ethiopia, Niger, and Senegal in sub-Saharan Africa. The study systematically reviewed all available data for mortality and cause of death analysis, including empirical estimates from national surveys and model-based estimates from the United Nations and the Institute for Health Metrics and Evaluation.

**Results**

Enormous data availability gaps exist across all seven countries in maternal mortality, stillbirths, and cause of death, with higher challenges in African countries. On average, only three data points were available since 2000 across these countries for maternal mortality ratio (MMR) trends estimation, ranging from two in Bangladesh, Niger, and Senegal to seven in India. The wide uncertainty range of the MMR estimates challenges conclusions on trends and inequalities. Data on neonatal mortality rate (NMR) is better, with, on average, 19 data points used by the United Nations for trends estimation since 2000, ranging from three in Morocco to 39 in Bangladesh. However, data quality remains a challenge and, in many cases (as in Bangladesh, Ethiopia, and Niger), recent modeled estimates did not capture a suspected stall or rise in mortality indicated by empirical estimates. No country has satisfactory data for cause-specific maternal and neonatal mortality trends, forcing reliance on model-based estimates with limited empirical data inputs.

**Conclusions**

As countries progress through the transition for maternal, late fetal, and neonatal mortality, the need for better data on mortality and causes of death is critical for assessing current status and benchmarking future mortality decline and associated strategies. Countries with high coverage of facility births have opportunities to collect better mortality data, while linking community and facility information systems.

## F14.1. Interactive dialogue on quality of care measurement

### Francesca Conway^1^, Paul Dsane-Aidoo^2^, Margaret Titty Mannah^3^

#### ^1^World Health Organization; ^2^United Nations Children’s Fund; ^3^Ministry of Health and Sanitation of Sierra Leone

*BMC Proceedings 2024*, **18(5):**F14.1

Submission ID #: IMNHC1542

Panel ID# (if applicable): DOEMC101542

**Panel Description**

In February 2017, Côte d’Ivoire, Ghana, Ethiopia, Malawi, Nigeria, Sierra Leone, Tanzania, Uganda, India, and Bangladesh, led by the World Health Organization, United Nations Children’s Fund, and United Nations Population Fund and with the support of partners, established the Quality of Care (QoC) Network for improving QoC for maternal, newborn, and child health. Improving QoC measurement is one of the core priorities of the QoC Network’s work. With attendees representing lead United Nations agencies, governments, civil society, and others, the interactive dialogue will seek to share the QoC Network’s evidence and experiences at the global and national levels in addressing challenges around QoC measurement. The interactive dialogue will also aim to share current trends, good practices, innovative approaches, and lessons learned; identify common challenges across countries regarding QoC measurement; and promote linkages between national strategies and the global normative framework for QoC measurement. The outcomes of the dialogue will help to enable pathways to drive collaboration, coordination, cross-country learning, and alignment; and to share evidence within the maternal and newborn health community.

## F14.2. The journey to creating a robust data pipeline for common Maternal and Newborn Health (MNH) quality of care indicators in the quality of care network: challenges and prospects

### Martin Dohlsten, Francesca Conway, Moise Muzigaba, Gerard Lopez, Theresa Diaz, Blerta Maliqi

#### World Health Organization

*BMC Proceedings 2024*, **18(5):**F14.2

Submission ID #: IMNHC1563

Panel ID# (if applicable): DOEMC101542

**Background**

In February 2017, the Quality of Care (QoC) Network for improving QoC for maternal, newborn, and child health was established. The QoC Network is led by the World Health Organization, United Nations Children’s Fund, and United Nations Population Fund with support of partners. This paper describes how the QoC Network Secretariat has facilitated the collection, sharing, quality assurance, analysis, and use of common MNH QoC data from multiple health facilities and learning sites participating in the network, and some of the challenges faced in rolling out this strategy.

**Methods**

The QoC Network Secretariat went through the following process: (1) standardizing data collection forms for a minimum set of quality indicators, (2) disseminating and strengthening capacity on these tools and indicators, (3) developing a data exchange web-based knowledge hub to facilitate the exchange of data between global and country levels, (4) conducting data quality verifications to assess the completeness, accuracy, and currency of the data received, and (5) analyzing the data and utilizing and disseminating the findings.

**Results**

In most countries, the ministries of health and partners adopted these tools, resulting in a heightened culture of data use for MNH QoC across all levels. The tools were also seen as instrumental in capturing data on indicators outside of the District Health Information Software 2 (DHIS2) such as women’s experience of care and water, sanitation, and hygiene indicators. The results of the data quality assurance and verification process also saw an overall improvement in the completeness, accuracy, and currency of the data. Ultimately, data analysis results and results from independent reviews indicate positive improvements in some MNH indicators over time. Despite the good progress, challenges remain in ensuring optimal data quality and guaranteeing adequate financial and human resources for data management. Prospects include updating these tools to capture pediatric/small and sick newborn and women’s experience of care QoC indicators, as well as capturing other indicators that span the continuum of care. Improving data quality, use, and quality improvement documentation are also priorities.

**Conclusions**

More still needs to be done to institutionalize MNH QoC measurement for informed decision-making. Adequate financial and human resources and strategic partnerships will be vital.

## F14.3. Lessons learned in measuring and monitoring the World Health Organization (WHO)-recommended Maternal and Newborn Health (MNH) experience of care indicators for the quality of care network: the Ghana case study

### Francesca Conway^1^, Paul Dsane-Aidoo^2^

#### ^1^World Health Organization; ^2^United Nations Children’s Fund

*BMC Proceedings 2024*, **18(5):**F14.3

Submission ID #: IMNHC1552

Panel ID# (if applicable): DOEMC101542

**Background**

Access to high-quality person-centered experience of health care through effective communication with clients, respect and dignity, emotional support, and continuity of care during pregnancy and childbirth is a key priority advocated for by the Quality of Care (QoC) Network. This fundamental right influences health outcomes and future health care use. Despite its importance for accountability and action, measurement of experience of care is limited. This study analyzed the experiences of care of postnatal mothers received from primary and secondary health facilities in QoC Network regions in Ghana.

**Methods**

A multi-center cross-sectional survey was conducted among postpartum mothers in QoC Network implementing facilities (health centers, polyclinics, and district hospitals) in Ghana from April 2020 to December 2021. We adapted Ghana’s maternal and child health record book to develop a structured questionnaire using the Open Data Kit tool. We conducted pre-discharge postnatal clinic exit interviews of mothers, and collected sociodemographic and obstetric variables of mothers, health facility characteristics, and mothers’ feedback on the health care experience. We conducted descriptive analysis of quantitative data and, in the process of program implementation, shared monthly feedback with implementing facilities and districts.

**Results**

About 126 health facilities from 10 implementing regions submitted responses. Overall, 7,379 mothers were interviewed with 55% (4,059/7,379) receiving care in district hospitals. The median age of mothers was 28 years (12–52), 35.8% (2,644/7,379) had up to secondary-level education, and 30.5% (2,231/7,379) were first-time mothers. Of 5,502 mothers who wanted a birth companion, 68.9% (3,793) were granted one during labor and delivery. Up to 5.1% (379/7,379) reported maltreatment, with shouting, hitting, and insults being the predominant types of abuse experienced. As a measure of their overall satisfaction with care, approximately 99.0% (7,305/7,379) were willing to recommend the health facility to others for delivery.

**Conclusions**

Prevalence of mistreatment in labor was significantly lower in the QoC Network facilities across 10 regions in Ghana. One-third of facilities have challenges supporting birth companions as standard of care. An overwhelming majority of respondents were satisfied with the care experience and would recommend facility delivery to others. The QoC Network intervention in Ghana showed encouraging results for scaling up across all regions and other low- and middle-income countries.

## F14.4. The process of integrating new Maternal and Newborn Health (MNH) Quality of Care (QoC) indicators in health information systems: experiences from Sierra Leone

### Francesca Conway^1^, Margaret Titty Mannah^2^, Binyam Hailu^1^, Ambrose Agweyu^3^

#### ^1^World Health Organization; ^2^Ministry of Health and Sanitation of Sierra Leone; ^3^KEMRI-Wellcome Research Programme

*BMC Proceedings 2024*, **18(5):**F14.4

Submission ID #: IMNHC1547

Panel ID# (if applicable): DOEMC101542

**Background**

In February 2017, the Quality of Care (QoC) Network for improving QoC for maternal, newborn, and child health (MNCH) was established. The QoC Network is led by the World Health Organization, United Nations Children’s Fund, and United Nations Population Fund with support of partners. Improving QoC measurement is one of the core priorities of the QoC Network’s work.

**Methods**

To strengthen QoC measurement in supported countries, the QoC Network released a Measurement Monitoring and Evaluation Framework for MNH QoC along with specific guidance. This abstract summarizes the process and lessons learned by the Sierra Leone country team in operationalizing this guidance at the country level.

**Results**

The process of the Ministry of Health and partners involved ensuring governance structures and functional technical working groups were in place for QoC measurement in all learning districts and facilities to ensure a growing culture of data use. Further, the team developed and refined a minimum set of quality indicators; adapted and developed district and facility data collection tools to capture essential data on the new indicators; ensured the reporting systems for facility, district, and national levels captured the new indicators; and developed a QoC indicator dashboard, which is integrated in the District Health Information Software 2 (DHIS2). The MNCH QoC indicators in Sierra Leone were integrated in the reproductive, maternal, newborn, child, and adolescent health scorecard, and the country team developed a maternal death surveillance and response line listing, perinatal death surveillance and response line listing, and child death audit line listing tool to document health facility deaths. The audit line listing tool is integrated in the DHIS2, and efforts are underway to integrate the pediatric death audit line listing tool. The country team also hired a new cadre of committed district and hospital QoC Officers and strengthened their capacity to analyze and communicate quality indicators. Further data quality audits through periodic supportive supervision took place. Funding was secured for QoC and measurement efforts from the Swedish International Development Cooperation Agency (SIDA), Large Anonymous Donor (LAD), and MOMENTUM with funds from USAID.

**Conclusions**

Key challenges remain, including competing activities at the Ministry of Health, too frequent coordination meetings, poor data quality, and limited funding from the government to support QoC operations in facilities and districts. This is not unique to Sierra Leone, as other QoC Network countries have had similar experiences.

## F15.1. What matters to you: centering women’s voices in designing dignified maternity care

### Minara Chowdhury^1^, Nana Amma Yeboaa Twum-Danso^1^, Abiyou Alemayehu^1^, Birkety Jembere^2^, Shannon Welch^1^

#### ^1^Institute for Healthcare Improvement; ^2^United States Agency for International Development

*BMC Proceedings 2024*, **18(5):**F15.1

Submission ID #: IMNHC1450

Panel ID# (if applicable): DOKCH231450

**Panel Description**

The quality of the birthing experience is highly variable. Women describe how they are not listened to or believed when sharing concerns with their medical provider, and in turn do not feel valued or respected. While there has been much focus on supporting providers to deliver effective care, there has been less focus on the experience of care by the mothers themselves and the impact of the care they receive, the manner in which care is delivered, and the environment in which it is delivered. Designing a better care experience starts with asking mothers “what matters to you?” but their answers need to be turned into action. Those answers can drive the way facilities are designed, choice of care, and how care is co-designed and sensitively delivered. The presenters in this panel bring novel insights from Bangladesh, Ethiopia, and the United States about how women can be engaged to codesign safer, more dignified, respectful, and equitable care.

## F15.2. Supporting community collaborations to codesign systems to improve maternal health and equity

### Minara Chowdhury, Nana Amma Yeboaa Twum-Danso, Shannon Welch, Dorian Burks

#### Institute for Healthcare Improvement

*BMC Proceedings 2024*, **18(5):**F15.2

Submission ID #: IMNHC1460

Panel ID# (if applicable): DOKCH231450

**Background**

In the United States, the burden of maternal mortality and morbidity falls disproportionately on Black women. Black women are three to four times more likely to die from pregnancy and childbirth complications than White women due to gaps in care and poor treatment upon accessing the health system. Countless Black women have shared stories illustrating how they are not listened to or believed when sharing concerns with their medical provider, and in turn do not feel valued or respected. While maternity care bundles detail what care to provide and under what circumstances, there is a large gap between what care should be provided and how to provide that care optimally to everyone.

**Methods**

The Institute for Healthcare Improvement (IHI) Better Maternal Outcomes: Redesigning Systems with Black Women project, supported by Merck for Mothers, aimed to facilitate locally driven, co-designed, rapid improvements in four U.S. communities targeting the interface of health care delivery, the experience of Black people who birth, and community support systems. The initiative aimed to improve equity, dignity, and safety while reducing racial inequities in maternal outcomes for Black people who birth. The project used the Equity Action Lab model, which guides participants through a structured set of activities to set health equity goals and develop ideas to test during an action phase to make progress. Between action phases, the IHI team provided improvement coaching and measurement strategy support to the communities. Each community convened three improvement teams to design, test, and implement changes to care delivery across the continuum of birth, labor and delivery, and postpartum.

**Results**

Each participating community achieved positive results, ranging from the successful development and implementation of an unconscious bias training program, respectful care training and simulation experiences for medical providers, building trust between patients and providers, and advancing partnerships between providers and community-based doulas.

**Conclusions**

In each of the participating communities, the health system and community-based organization partners highlighted the value of co-designing solutions with Black persons who birth. The Equity Action Lab model centered the voices and experiences of Black persons who birth, identifying actions and solutions that built trust and advanced local partnerships and improvement efforts.

## F15.3. Quality birthing experience: implications of the built environment on opportunities for mothers’ pain management and choice of birthing options

### Minara Chowdhury, Nana Amma Yeboaa Twum-Danso, Sumona Ferdous, Stephen Luna-Muse, Samina Yasmin

#### Institute for Healthcare Improvement

*BMC Proceedings 2024*, **18(5):**F15.3

Submission ID #: IMNHC1455

Panel ID# (if applicable): DOKCH231450

**Background**

Through the U.S. Agency for International Development MaMoni Maternal and Newborn Care Strengthening Project, the Institute for Healthcare Improvement (IHI) has supported the implementation of maternal and newborn health (MNH) quality of care (QoC) bundles in Bangladesh to strengthen the capacity of service providers to support mothers through improvements in administrative and clinical quality. These efforts have been extended to introducing nonpharmacological methods of pain management during labor. These approaches include mobility of women during labor, accompanying birth companions, alternative birth positions, and physical comfort (e.g., massage). The implementation of these interventions may be hampered by lack of or suboptimal physical space in the maternity ward. IHI, in partnership with the MASS Design Group and International Centre for Diarrhoeal Disease Research, Bangladesh (icddr,b), engaged with newly postpartum mothers to understand the impact of maternity ward design on care experience and their willingness to seek skilled maternal care.

**Methods**

In October and November 2021, IHI engaged 44 postpartum mothers, eight birth companions, and 20 perinatal care providers in two districts in Bangladesh to learn about their experience delivering and receiving perinatal care in one of four selected health facilities. Additionally, local architects completed a comprehensive engineering assessment of the four facilities. These data were analyzed and used to produce designs for renovating the health facilities in order to improve the delivery and experience of care.

**Results**

Through the survey process, women, their companions, and providers articulated their desire to receive or support nonpharmacological methods of pain management. IHI and its architectural partners produced context-appropriate concept designs for the four participating health facilities that address each of the concerns raised and, if constructed, would facilitate use of the care bundles by creating sufficient space for companions, expanding and more fully equipping delivery rooms, and ensuring privacy and comfort for women to move about during labor as desired.

**Conclusions**

Women value nonpharmacological pain management, and providers have the skill and will to fulfill that need, but full implementation will require more than willing participants—it will require thoughtfully designed facilities that enable provision of the high-quality care that women deserve.

## F15.4. Scaling up effective approaches to improve dignity and respect of women around the time of birth

### Abiyou Alemayehu^1^, Nana Amma Yeboaa Twum-Danso^1^, Birkety Jembere^2^

#### ^1^Institute for Healthcare Improvement; ^2^United States Agency for International Development

*BMC Proceedings 2024*, **18(5):**F15.4

Submission ID #: IMNHC1454

Panel ID# (if applicable): DOKCH231450

**Background**

Mistreatment of women during facility-based childbirth is widespread and often deters women from attending skilled birth. In Ethiopia, nearly half of women giving birth in health facilities experience disrespectful or undignified care. Critical gender barriers obstruct women and teenage girls from receiving safe and dignified care, while poor communication and coordination across levels of care delivery hinder full implementation of quality strategies and stifle referral pathways, which are crucial for delivering care that is timely, safe, and effective. An initiative in three regions of Ethiopia showed that training on respectful care led to improvements in privacy and birth companionship. An effort is now underway to refine and scale up this work.

**Methods**

A systematic approach to addressing this basic human right includes deeply understanding what women want around the time of birth, surfacing behaviors and attitudes that perpetuate the problem, training and coaching, learning about what works, and scaling effective approaches across health systems. The successful interventions and tools developed in the prototype phase will be scaled across the district-based system of Ethiopia. Interventions include teaching curricula that sensitize caregivers to the experiences of women around the time of birth (e.g., video testimonials), accompanied by a set of “change ideas” for implementation. Specific measures (e.g., presence of a birth companion) will be routinely tracked and reflected back to care providers to improve site-based adherence to these actions.

**Results**

While respectful maternal care training addresses all seven categories of mistreatment, births with privacy and birth companion offered were found to be a good proxy for respectful maternal care, and these two measures will be included in routine district datasets. Time-series depiction of data trends will be reflected back to providers for learning and improvement.

**Conclusions**

A lack of respect and dignity for women around the time of birth is a global problem. Solving this problem will require national scale-up of approaches that have been demonstrated to address needs articulated by women.

## F16.1. Achieving country-wide high-quality Maternal, Newborn, and Child Health (MNCH) care: the path from innovation to scale and sustainability

### Pierre Barker^1^, Mamun Bhuiyan^1^, Paulo Borem^1^, Nebiyou Wendwessen Hailemariam^1^, Mazharul Hoque^2^, Abiyou Alemayehu^1^

#### ^1^Institute for Healthcare Improvement; ^2^Ministry of Health and Family Welfare, Bangladesh

*BMC Proceedings 2024*, **18(5):**F16.1

Submission ID #: IMNHC1490

Panel ID# (if applicable): DPJOM641490

**Panel Description**

Sustainable Development Goal targets for rapid reduction of maternal and newborn mortality and stillbirths will only be reached if known evidence-based interventions can be delivered by country health systems using designs that are effective, scalable, and sustainable. The content and methods for effective and scalable interventions must be simple, focused, and transferable. Sustainability requires minimal reliance on external technical resources. We show examples of how quality improvement methods have been adapted to include clinical and health system interventions, how focused clinical bundles can lead to rapid adoption of best practices at scale, and how country health systems can be supported to rapidly and independently scale successful MNCH interventions. This panel will showcase the journey from demonstration of effectiveness, to methods for scale and government-led scale-up. With examples from Brazil, Ethiopia, and Bangladesh, practioners and government planners will describe innovative approaches to improving safety of maternal care, the use of clinical and administrative bundles to improve reliability of care, rapid replication designs to improve uptake of best practice clinical care, and what it takes to integrate new implementation designs and quality improvement capability into government-led scale-up of MNCH programming.

## F16.2. Reducing institutional perinatal mortality across multiple district hospitals: applying clinical bundles in a Quality Improvement (QI) collaborative

### Abiyou Alemayehu, Pierre Barker, Mamun Bhuiyan, Gebremeskel Tamene, Nebiyou Wendwessen Hailemariam, Abdu Abera, Yeneneh Getachew

#### Institute for Healthcare Improvement

*BMC Proceedings 2024*, **18(5):**F16.2

Submission ID #: IMNHC1527

Panel ID# (if applicable): DPJOM641490

**Background**

Perinatal mortality in Ethiopia is one of the highest in Africa, with 33 per 1,000 births. Using data from District Health Information Software 2 (DHIS2), 10 hospitals in the Sidama region—one of the 11 administrative regions of Ethiopia—were selected based on the highest burden of perinatal mortality: the median institutional perinatal mortality (IPM) of these hospitals was 59 per 1,000 births. The Institute for Healthcare Improvement (IHI) supported the Sidama regional health bureau (SRHB)-led perinatal mortality reduction collaborative in 10 hospitals, aiming to reduce perinatal mortality by 30%, from the baseline, over six months.

**Methods**

IHI and SRHB provided basic QI training, continuous coaching support, and mentorship for over eight months. Three learning sessions were conducted. Baseline and follow-up data collected from DHIS2 were validated from charts and registers review. IHI designed a weekly electronic data collection and tracking dashboard, including both outcome and care bundle (tracking specific phase of care) indicators, and the hospital QI teams were trained on its application. Hospital QI teams collected six bundle indicators focusing on aspects of mothers and newborns relevant to prevention of early neonatal mortality and stillbirths. Clinical bundle indicators included neonatal intensive care unit general care, preterm with respiratory distress, sepsis detection, preterm labor, eclampsia, and postpartum hemorrhage.

**Results**

On average, IHI conducted five onsite coaching visits and six virtual coaching sessions for each hospital. IHI and SRHB delivered three learning sessions and one round of basic QI training. A total of 24 QI projects (2.4 per hospital) were designed and implemented. All bundle indicators except for pre-eclampsia/eclampsia management showed an average increment ranging from 5% to 59% in all hospitals. These process improvements were associated with a significant reduction of IPM in the 10 hospitals (59 to 41.5 IPM per 1,000 hospital births).

**Conclusions**

Focused QI approaches using bundled interventions and continuous bundle tracking in resource-limited hospitals can achieve significant reduction in IPM over a short period of time. While the greatest regional impact can be achieved by initially focusing on the few hospitals that contribute to most of the mortalities, scale-up of successful approaches to all hospitals in the project implementation regions.

## F16.3. Region-Scale Maternal, Newborn, and Child Health (MNCH) Improvement of Quality of Care (QoC) in resource-limited settings: lessons from Ethiopia

### Nebiyou Wendwessen Hailemariam^1^, Abiyou Alemayehu^1^, Pierre Barker^1^, Mamun Bhuiyan^1^, Yeneneh Getachew^1^, Desalegn Bekele Taye^2^

#### ^1^Institute for Healthcare Improvement; ^2^Ministry of Health, Ethiopia

*BMC Proceedings 2024*, **18(5):**F16.3

Submission ID #: IMNHC1522

Panel ID# (if applicable): DPJOM641490

**Background**

Improved outcomes for mothers and newborns across low- and middle-income country health systems require government-led testing and scaling up of evidence-based QoC interventions. In addition to essential inputs of human and physical resources and clinical competencies, national scale-up requires local capability for planning, testing, and scaling up a learning-based (quality improvement [QI]) approach.

**Methods**

The Institute for Healthcare Improvement (IHI) supported the Ethiopian Federal Ministry of Health (FMOH) efforts to test and scale up effective maternal and newborn quality of care in two phases: testing of MNCH change packages in a limited set of districts and scaling up the refined change packages across the country. The initiative built QI, financial, and technical skills for regional, zonal, and district leaders, and provided onsite and virtual coaching visits and biannual zonal-level QI coach review meetings. MNCH improvements were tested across a “scalable unit” that included the zonal health department (ZHD), a district health office, and the health facilities (primary hospitals, health centers, and health posts). The scalable unit was replicated during scale-up. Processes and outcomes were tracked using 11 District Health Information Software 2 (DHIS2) indicators and seven new bundle measures derived from client charts. The FMOH assumed responsibility for QI training during the scale-up.

**Results**

Between October 2019 and December 2021, the FMOH and regional, zonal, and district teams delivered the MNCH packages using QI methods to 40% (357) of the country districts within 27 zones and seven regions, involving 1,613 health facilities. IHI and regional health bureau (RHB) provided QI coaches training for 1,248 leaders and local QI coaches. RHB and ZHD provided basic QI training for 3,909 health providers. Almost all (93.8%) health facilities conducted a baseline assessment and most (98%) tracked their progress using 18 MNCH indicators during the project period. Facilities designed and tested 2,875 QI projects to implement the change packages sustainably and effectively. During the project implementation period, facilities reported improvement, on average, in three MNCH indicators.

**Conclusions**

Nationwide scale-up is possible using a planned, phased zonal-based scale-up approach that is owned and led by the government to effectively implement MNCH interventions in resource-limited settings.

## F16.4. Creating sustainable, government-led quality improvement capability through maternal-newborn programming in Bangladesh

### Jamal Uddin^1^, Pierre Barker^1^, Mamun Bhuiyan^1^, Mazharul Hoque^2^, Abiyou Alemayehu^1^, Minara Chowdhury^1^

#### ^1^Institute for Healthcare Improvement; ^2^Ministry of Health and Family Welfare, Bangladesh

*BMC Proceedings 2024*, **18(5):**F16.4

Submission ID #: IMNHC1518

Panel ID# (if applicable): DPJOM641490

**Background**

The Institute for Healthcare Improvement (IHI), through the U.S. Agency for International Development’s MaMoni Maternal and Newborn Care Strengthening Project, introduced quality improvement (QI) initiatives in 13 districts of Bangladesh through clinical and operational bundle testing and implementation. However, donor-driven approaches are challenging to sustain when resources are withdrawn. Sustainability of QI initiatives requires government ownership, capability to independently undertake the work, and use of the government’s own resources to sustain the work. The Directorate General Health Services (DGHS) in Bangladesh was supported to independently apply the learnings to non-project areas using government resources.

**Methods**

The project worked closely with the government to support their independent implementation of QI approaches in non-project areas, without reliance on nongovernmental organization (NGO) country-based resources, and using their own budgeted finances. DGHS incorporated the QI bundle approaches in their operational plan, allocating budget for training and implementation. Government-employed staff taught QI basics and supported antenatal care (ANC) bundle training in district hospitals outside the NGO-designated project areas using existing government resources. Maternal and newborn health process data and implementation learnings were shared regularly across participating sites using online social media platforms. Offsite follow-up coaching of the facilities within the system is provided virtually from DGHS with onsite follow-up by local authorities.

**Results**

With minimal further technical guidance from the IHI project, and to test the approach, three out of six district hospitals have already implemented the quality ANC bundle through the government support system. Before the implementation of the ANC bundle, there was negligible observed compliance with the core elements of the bundle in the selected district hospitals. Within four months of implementation of the bundle, the compliance with quality ANC increased to 48%.

**Conclusions**

Donor-based projects must have a plan to support government efforts to scale and sustain system improvements that have been demonstrated in the project areas. Traditional approaches to hand over plans, such “how to” guides, are not sufficient. Coaching and co-delivery with government health systems, and support for independent planning and implementation of new methods such as QI offer the opportunity for scale-up and sustainability of effective models of care.

## F16.5. Mother hug collaborative: reducing institutional maternal mortality in Brazil using early warning systems and bundles of care

### Paulo Borem^1^, Pierre Barker^1^, Mamun Bhuiyan^1^, Claudia Garcia De Barros^2^, Rodolfo Pacagnella^3^

#### ^1^Institute for Healthcare Improvement; ^2^Hospital Albert Einstein, ^3^University of Campinas

*BMC Proceedings 2024*, **18(5):**F16.5

Submission ID #: IMNHC1498

Panel ID# (if applicable): DPJOM641490

**Background**

Brazilian hospitals continue to experience high rates of maternal mortality, and the country is not on track to achieve its Sustainable Development Goal targets. The most prevalent life-threatening conditions during pregnancy—sepsis, eclampsia, and hemorrhage—can be averted and managed if detected early and treated promptly and effectively using available protocols and skills.

**Methods**

We used the “4Rs” approach (recognize early clinical deterioration, rescue when there is a life-threatening condition, reassess after rescuing, and refer the patient) to detect and manage obstetric complications. The Modified Early Obstetric Warning Score (MEOWS) is used to detect imminent or established clinical distress in obstetric patients based on vital signs. A bundle of evidence-based interventions was applied to clients for MEOWS > 4 or > 3 in one of the parameters and clinical suspicion of sepsis or pre-eclampsia. For hemorrhage, the trigger to apply the bundle was bleeding > 500 ml for vaginal birth and 1,000 ml for C-sections. We used Training Within Industry (TWI) to standardize processes of care. Eighteen public hospitals participated in an Institute for Healthcare Improvement Breakthrough Series Learning Collaborative (November 2019 to April 2021) that included two separate sessions for leadership and teams from participating hospitals (an executive leader, an obstetrician, and an obstetric nurse) received training on equity, quality improvement methods, TWI, and MEOWS. Teams designed initial tests of change to increase adherence to the bundles and shared successes and challenges in implementing the changes. Because of the COVID-19 pandemic, all interactions with participating organizations were virtual.

**Results**

Compliance with MEOWS across the 18 hospitals increased from 78% to 98%. The aggregated data on institutional maternal mortality showed a reduction of 62% in the three life-threatening conditions (sepsis, hemorrhage, and eclampsia). Mortality due to sepsis decreased by 95% and hemorrhage by 73%, but no reduction was observed in mortality due to eclampsia. Despite the COVID-19 pandemic, mortality from sepsis and hemorrhage did not increase, but institutional maternal mortality increased by 178% after February 2021.

**Conclusions**

The 4Rs strategy, including MEOWS, bundle of care application, equity training, and process standardization, effectively reduced institutional maternal mortality in the public sector in Brazil. The intervention was sufficiently resilient to withstand the additional disruption of the COVID-19.

## F17.1. Measuring quality of care and effective coverage for newborn health: progress and gaps

### Melinda Munos^1^, Ashley Sheffel^1^, Josephine Exley^2^, Kiersten Israel-Ballard^3^

#### ^1^Johns Hopkins Bloomberg School of Public Health; ^2^London School of Hygiene and Tropical Medicine; ^3^PATH

*BMC Proceedings 2024*, **18(5):**F17.1

Submission ID #: IMNHC1569

Panel ID# (if applicable): DPJSF961569

**Panel Description**

Data on intervention coverage (the proportion of individuals in need of an intervention who receive the intervention) and health service quality are essential for identifying under-served populations and gaps in service delivery, and for developing and evaluating quality improvement initiatives and maternal and newborn health programs. Traditional measures of intervention coverage do not account for the quality of the services received; effective coverage (EC) is an approach to incorporating quality in coverage measures. However, this area of newborn health metrics is rife with gaps: gaps in available data on coverage, readiness, and service quality for newborns, and gaps in the methods for assessing service quality and estimating EC. These gaps limit our ability to understand the care currently received by newborns across low- and middle-income countries, making it more difficult to develop evidence-based programs and interventions. This panel will present recent research that aims to define and address some of these gaps, including the development of methods and indices to assess service quality for newborn health; assessment of readiness and quality data on newborns in facility registers and routine data systems; and development of EC cascades and measures for postnatal care, care for small and sick newborns, and feeding small and sick newborns.

## F17.2. The potential of routinely available data to support the uptake of actionable effective coverage measures for newborn care in Northeast Nigeria

### Melinda Munos^1^, Josephine Exley^2^, Tanya Marchant^2^

#### ^1^Johns Hopkins Bloomberg School of Public Health; ^2^London School of Hygiene and Tropical Medicine

*BMC Proceedings 2024*, **18(5):**F17.2

Submission ID #: IMNHC1608

Panel ID# (if applicable): DPJSF961569

**Background**

There are few examples of effective coverage (EC) measures implemented in low- and middle-income countries and limited information about the availability of data needed to estimate EC and relevant to decision-makers working at different levels of the health system. Drawing on learning from the Gombe maternal and newborn partnership, we examine the newborn health data routinely available to decision-makers, identify gaps in data availability, and discuss implications for EC and for the utility of EC estimates for state-level decision-makers.

**Methods**

Measuring EC for newborn care relies on linking information on access to care from household surveys, with information on the quality of care from health facility datasets. We assessed the availability of data needed to construct measures of input- and quality-adjusted coverage in facility registers, District Health Information Software 2 (DHIS2), and household surveys in Gombe State, Nigeria.

**Results**

In Gombe, facility-level data is documented in 13 paper-based registers. Each month, a subset of data are included in DHIS2. DHIS2 is available monthly for all facilities. However, these data only allow measurement of input-adjusted coverage. Some data capturing aspects of intervention- and quality-adjusted coverage for newborns are collected in facility registers and could be added to DHIS2, but are insufficient to construct a comprehensive EC measure.

Data on newborn use of care are readily available from nationally representative household surveys, such as Demographic and Health Surveys (DHS) or Multiple Indicator Cluster Surveys. However, data collection is undertaken periodically, limiting utility where quality improvement is ongoing. The Nigeria DHS is representative at the national and state levels, enabling benchmarking between states, but does not provide granularity to support more targeted action within the state. Alternative sources of population data are needed. One proposal to overcome this limitation at present is to examine health facility readiness to provide high-quality care, as has been proposed for newborn resuscitation care.

**Conclusions**

There are considerable opportunities to better utilize routine data sources to support measurement of quality of care for newborn health interventions. Given the potential burden, any new data collection must be locally led to ensure it is relevant to local priorities.

## F17.3. Barriers and enablers to achieving effective coverage of feeding of the small and sick newborn in Low- and Middle-Income Countries (LMICs)

### Kiersten Israel-Ballard^1^, Melinda Munos^2^, Megan Parker^1^, Elan Ebeling^1^, Sadaf Khan^1^, Jessica Shearer^1^, Katharine Shelley^1^, Kimberly Mansen^1^

#### ^1^PATH; ^2^Johns Hopkins Bloomberg School of Public Health

*BMC Proceedings 2024*, **18(5):**F17.3

Submission ID #: IMNHC1577

Panel ID# (if applicable): DPJSF961569

**Background**

The World Health Organization (WHO) recommends mothers’ own milk or donor human milk for small and sick newborns (SSNB), however, how SSNB are fed and supported to receive human milk is not well understood. To inform future implementation strategies, the Asset Tracker project sought to identify and understand barriers and enablers to scale across six key nutrition interventions, including feeding of the small and sick newborn (FSSN).

**Methods**

The Asset Tracker six-stage scale-up framework toward effective coverage—global guidelines and market availability, national policy adoption, system integration and readiness, implementation and service delivery, availability, and coverage—anchored data collection, analysis, and synthesis across a multistep approach for each intervention. This included performing a literature review on barriers, enablers, and implementation; conducting 41 global and 53 country key informant interviews across Burkina Faso, Ethiopia, India, Kenya, and Nigeria; compiling 99 nutrition indicators relevant to six assets from Demographic and Health Surveys, Service Provision Assessments, Service Availability and Readiness Assessments, health management information systems, Every Newborn Action Plan, and WHO Policy Survey; and reviewing 277 policy documents, budgets, guidelines, and training curricula. Tableau data visualizations were created to aid interpretation as an interactive global good.

**Results**

Three Asset Tracker subgroups relating to barriers, enablers, and implementation reach along the framework toward effective coverage include (1) new and donor dependent, (2) complex and for specific cases, and (3) available/supported but not equitable. FSSN was categorized as new and donor dependent—lacking clear policies and consistent implementation guidance, country ownership and sustainability/transition plans, sustainable procurement options, and funding for political advocacy at every level. Most indicators for FSSN were indirect, not routinely disaggregated from term infants, and detailed feeding and lactation data are lacking. In-service FSSN trainings were identified only in Kenya and India.

**Conclusions**

Routine data systems do not currently document how SSNB are fed globally or how mothers are supported with specialized lactation needs. Despite increased global momentum to improve the quality of care for SSNB, inclusion of feeding as a core component of care is lacking. Data, implementation models, and operational standards are needed. Aligning priorities and investments is needed to shift FSSN to the center of improving quality of care, and as a link across kangaroo mother care, nurturing care, and respectful maternal care. More information on the Asset Tracker project can be found by visiting: https://www.path.org/programs/primary-health-care/tracking-the-journey-to-scale

## F17.4. Effective coverage for newborn health: operationalizing effective coverage cascades for small and sick newborn care in seven low- and middle-income countries

### Melinda Munos^1^, Ashley Sheffel^1^, Lori Niehaus^2^

#### ^1^Johns Hopkins Bloomberg School of Public Health; ^2^Centers for Disease Control and Prevention

*BMC Proceedings 2024*, **18(5):**F17.4

Submission ID #: IMNHC1573

Panel ID# (if applicable): DPJSF961569

**Background**

Standard coverage measures do not account for quality of health services received by newborns and therefore do not accurately reflect the impact of programs. Effective coverage cascades have been proposed to capture the quality of services in population-based measures of intervention coverage. While a conceptual framework for coverage cascades has been developed, this approach has not been operationalized to ensure the framework is responsive to the needs of decision-makers and provides actionable information for various users. The aim of this work was to operationalize the effective coverage cascade for small and sick newborn care (SSNC) using extant, publicly available data across low- and middle-income countries.

**Methods**

Using existing household survey and health facility assessment data from seven low- and middle-income countries, we estimated effective coverage cascades for SSNC. We developed theoretical coverage cascades for SSNC; defined facility readiness and provision of care scores; and linked readiness and quality scores to household survey data based on geographic domain and facility type. Finally, we estimated the steps of the coverage cascade for each country.

**Results**

Overall, coverage of institutional delivery varied across countries, from 38% in Bangladesh to 91% in Malawi. A substantial drop was seen in coverage of institutional delivery to overall SSNC readiness-adjusted coverage. The highest drop in coverage was 31 percentage points in Senegal (coverage of 63%, readiness-adjusted coverage 40%), while the lowest drop in coverage was 17 percentage points in Bangladesh (coverage of 38%, readiness-adjusted coverage 20%). Some interventions scored consistently low across countries, including kangaroo mother care, essential newborn care, and prevention of mother-to-child-transmission of HIV. Other interventions score consistently higher across countries, including general readiness items and resuscitation.

**Conclusions**

The cascade approach yielded summary measures that identified high-level barriers to effective coverage; however, detailed measures within the cascade may be more useful for evidence-based decision-making. Work is planned to understand whether and how these measures could be used by decision-makers at national and subnational levels. Data availability on quality of care for SSNC is scant, highlighting an opportunity to expand facility-based surveys to include referral-level SSNC services.

## F17.5. Advancing maternal and newborn measurement: developing quantitative measures of service quality for postnatal care and small and sick newborn care using extant data in low- and middle-income country health systems

### Ashley Sheffel^1^, Melinda Munos^1^, Lori Niehaus^2^

#### ^1^Johns Hopkins Bloomberg School of Public Health; ^2^Centers for Disease Control and Prevention

*BMC Proceedings 2024,* **18(5):**F17.5

Submission ID #: IMNHC1570

Panel ID# (if applicable): DPJSF961569

**Background**

Delivering high-quality health services to women and newborns around the time of delivery with a focus on birth, small and sick newborn care (SSNC), and postnatal care (PNC) is critical to making continued progress in reducing mortality in low- and middle-income countries (LMICs). LMICs are increasingly shifting focus toward improving the quality of health services delivered, which requires measuring quality of care (QoC) and monitoring progress. However, there are no standardized approaches to defining and measuring PNC and SSNC quality in these contexts. This study aims to systematically develop QoC indices for maternal PNC, newborn PNC, and SSNC.

**Methods**

To define QoC measures for maternal PNC, newborn PNC, and SSNC in LMICs, we followed a four-step process: (1) identify globally recommended interventions for inclusion in indices, (2) extract service readiness items from intervention-specific clinical and service implementation guidelines, (3) map the identified items from the guidance documents to available data in health facility surveys, and (4) develop QoC metrics informed by QoC frameworks, clinical guidelines, and data availability.

**Results**

The proposed maternal PNC QoC index reflects 11 interventions and contains 24 items. The proposed newborn PNC QoC index reflects three interventions and contains 16 items. The proposed SSNC QoC index reflects seven interventions and contains 37 items. Existing data sources have significant gaps for both PNC and SSNC, resulting in exclusion of some interventions and limitations on item inclusion. In addition, no provision of care data was available for PNC or SSNC, thus both indices reflect facility readiness only.

**Conclusions**

The indices provide a standardized measure for PNC and SSNC intervention readiness and can be adapted at the country level and operationalized using existing health facility assessment data, facilitating their use by LMIC decision-makers for planning and resource allocation. Revision of existing health facility assessments to address gaps in readiness and quality measurement for PNC and SSNC would bolster efforts to monitor and improve QoC for mothers and newborns.

## F18.1. The power of compassion in Maternal, Newborn, and Child Health (MNCH) service delivery

### Jessica Vandermark Moore^1^, Lynn Van Lith^2^, Chantalle Okondo^3^, Eva Lathrop^4^, Sandra Mwarania^5^

#### ^1^Camber Collective; ^2^Johns Hopkins Center for Communication Programs; ^3^Population Council; ^4^Population Services International; ^5^White Ribbon Alliance

*BMC Proceedings 2024*, **18(5):**F18.1

Submission ID #: IMNHC1008

Panel ID# (if applicable): DPNUE481008

**Panel Description**

Experience of care is a critical component of the Quality of Care Framework and is defined as effective communication, respect and preservation of dignity, and emotional support. Evidence suggests that person-centered care, comprised of kindness, empathy, and warmth, is critically important to a positive experience while also reducing burnout among providers. These factors play an important role in service delivery as they directly shape and influence how clients and providers experience one another. Practiced empathy and compassion can positively influence clients’ feelings and behaviors and are foundational to quality service provision, yet less focus has centered on these dimensions.

Panelists have conducted literature reviews, stakeholder consultations, interventions, and evaluations to further build evidence on improving the experience for both clients and MNCH providers. Objectives for this panel are to (1) accelerate learning around factors shaping experience of care and how best to inspire positive provider behavior change, and (2) advocate for an intentional focus on empathy and compassion to improve care for clients, while also addressing the needs of providers.

This panel highlights evidence in addressing and measuring compassionate care: the White Ribbon Alliance will showcase what providers need to enable them to provide such care; Breakthrough RESEARCH will share research findings from Kenya on experience of care among hospitalized children; MOMENTUM Private Healthcare Delivery will showcase a new evidence-based curriculum for private sector providers focused on building empathy; and Breakthrough ACTION will share progress on a compassionate care intervention for newborns and young children.

## F18.2. Emphasizing empathy and compassion to improve experience of care

### Jessica Vandermark Moore^1^, Lynn Van Lith^2^

#### ^1^Camber Collective; ^2^Johns Hopkins Center for Communication Programs

*BMC Proceedings 2024*, **18(5):**F18.2

Submission ID #: IMNHC1449

Panel ID# (if applicable): DPNUE481008

**Background**

Respectful, empathic, and compassionate care are critical components of experience of care (EoC). While respectful care has been advanced through the respectful maternity care agenda, less research and practice has centered around respect and EoC outside of labor and childbirth. Negative experiences may lead to reduced care-seeking behaviors and avoidance of health services. Growing evidence demonstrates that providers practicing empathy and compassion can positively impact clients’ feelings and behaviors and are foundational to quality service provision while also reducing provider burnout.

**Methods**

Breakthrough ACTION conducted a landscape review of 170+ sources (120 programmatic reports, more than 25 peer-reviewed articles, and eight systematic reviews) to understand factors that improve EoC for mothers, newborns/young children, and the providers who serve them. We then consulted 36 global experts to identify opportunities to enhance EoC through provider behavior change (PBC), using the PBC ecosystem map as a framework for categorizing influence factors, and co-designed a multifaceted facility-based intervention. By the time of the conference, we will have completed implementation of the pilot and evaluated its impact.

**Results**

Based on research and consultation, we identified actionable influence factors: (1) facility—infrastructure/equipment/staffing, leadership/team culture, trainings, and protocols; (2) individual provider—bias, stress/psychosocial well-being, training/competency, and work commitment/satisfaction; and (3) community—gender norms, social stigma, power dynamics, and inequities. We also identified opportunities to improve newborn and young child EoC by promoting health system and team culture (i.e., role modeling, mentorship, and peer support), accountability and feedback systems and capacity, and connectivity across departments and with the community.

**Conclusions**

EoC is a critical component for improved quality of care for postpartum mothers and their young children. Through understanding the key driving factors that influence EoC, interventions may better address provider behavior and improve EoC for both the client and the provider. In testing an intervention, we gain insight into effectiveness and additional areas of improvement, as well as map potential pathways to scale.

## F18.3. Perspectives on development and field testing of person-centered toolkit for private sector health care providers

### Eva Lathrop^1^, Lynn Van Lith^2^

#### ^1^Population Services International; ^2^Johns Hopkins Center for Communication Programs

*BMC Proceedings 2024*, **18(5):**F18.3

Submission ID #: IMNHC1445

Panel ID# (if applicable): DPNUE481008

**Background**

To accelerate progress toward ending preventable maternal, newborn, and child deaths, it is critical that health systems emphasize quality of care interventions that are responsive to people’s unique needs. Evidence has emerged around the importance of a person-centered care (PCC) approach to quality of care, but tools to strengthen PCC approaches that incorporate empathy are lacking. As part of the MOMENTUM Private Healthcare Delivery project, a novel toolkit was designed to elevate PCC across integrated family planning, reproductive health, and maternal, newborn, and child health (FP/RH/MNCH) services in the private sector.

**Methods**

A literature review was performed using agreed upon search terms, databases, and websites. To facilitate an objective review, a checklist in line with PCC principles was used to screen each resource. All resources were reviewed by one person; concerns were discussed and resolved with the project team. A toolkit was designed, followed by two consultations with a broad group of stakeholders to provide feedback on content, format, and usability.

**Results**

More than 100 resources were reviewed to identify person-centered resources across FP/RH and MNCH. There was a clear trend toward person-centered approaches and tools across MNCH, sexual and reproductive health and rights, youth, and gender, although resources specific to building empathy and trust were limited. Feedback from a global group of stakeholders included that content was focused on global guidelines and clinical protocols, with less of an emphasis on what makes care person centered. In response, the toolkit material was re-organized by PCC principles, including a section focused on empathy and respectful care, exercises were developed to give guidance on how materials can be applied in practice, and a facilitator’s guide was developed. Stakeholders agreed on a set of proposed core training modules focusing on PCC basics, respectful care across SRHR/MNCH, empathy and emotional intelligence, gender, and youth.

**Conclusions**

Care centered on people’s unique experiences and needs can elevate overall quality of care and could positively impact health seeking behavior and outcomes. Next steps include field testing the toolkit and training modules with a group of providers and coaches in Benin, followed by intervention research to assess provider perceptions of PCC training on their practices and client’ assessment of their experiences of care.

## F18.4. Improving experience of care for newborns and their parents: empathy and compassion for both parents and health care providers in Kenya

### Chantalle Okondo^1^, Lynn Van Lith^2^

#### ^1^Population Council; ^2^Johns Hopkins Center for Communication Programs

*BMC Proceedings 2024*, **18(5):**F18.4

Submission ID #: IMNHC1437

Panel ID# (if applicable): DPNUE481008

**Background**

Improving quality experience of care for hospitalized newborns requires a multi-pronged approach. Through a participatory approach with providers and parents, we co-developed and implemented a structural and provider behavior change intervention to improve inpatient experiences of care across five hospitals in Kenya. The intervention encompassed provider orientation on respectful and nurturing care and subsequent coaching of parents, emotional support for parents and providers, peer support for providers, and inter-provider feedback meetings.

**Methods**

A mixed-methods evaluation assessed the feasibility and acceptability of the interventions and measured parent and provider experiences between September 2020 and August 2021. With parents, we conducted short exit interviews (*n*=531) at midline, a follow-up survey (*n*=382), and qualitative in-depth interviews (*n*=15) at endline. Providers participated in a baseline (*n*=154) and endline survey (n=104), monitoring meetings a peer-feedback survey (*n*=105), and qualitative interviews (*n*=16). Qualitative data analysis included a combination of inductive and deductive thematic analyses while the quantitative data was analyzed descriptively, with factor and scale analysis and general linear regression models.

**Results**

Post-intervention, most parents (83%) reported feeling empowered in caring for their newborns, including receiving encouragement and reassurance from providers during management of their sick baby. Parents who received information on nurturing and responsive care also reported increased interpersonal communication with providers (β ²=0.32; *p*=0.000). Post-intervention, providers reported improved self-awareness, recognition of sources of stressors, and understanding of different coping mechanisms. The intervention enhanced teamwork to balance stress and workload, allowing providers to engage with parents more and provide responsive and respectful care. Provider burnout levels, such as emotional exhaustion, also decreased between baseline and endline (from 63% to 37%) due to peer-to-peer support and psychological debriefing sessions.

**Conclusions**

This intervention improved respectful and nurturing care for newborns in Kenya and supported providers working in high stress and heavy workload settings, despite persisting COVID-19 pandemic and infrastructure challenges. It is feasible and acceptable to implement a provider behavior change intervention to improve experiences of care for newborns and their parents.

## F18.5. What women and midwives want: integrated advocacy to enhance providers’ and women’s experience of care

### Sandra Mwarania^1^, Lynn Van Lith^2^

#### ^1^White Ribbon Alliance; ^2^Johns Hopkins Center for Communication Programs

*BMC Proceedings 2024*, **18(5):**F18.5

Submission ID #: IMNHC1429

Panel ID# (if applicable): DPNUE481008

**Background**

Health providers and clients alike have specific needs and require support to ensure the best care experience. However, they are rarely asked what they want and need, let alone have their demands acted upon. In response, White Ribbon Alliance (WRA) implemented two large-scale campaigns to hear women’s, girls’, and midwives’ concerns and aspirations for quality maternal and reproductive health care and by taking local action.

**Methods**

In 2018, WRA launched the What Women Want (WWW) campaign, which asked more than 1.3 million women worldwide about their one request for quality maternal and reproductive health care. In 2021, WRA and the International Confederation of Midwives embarked on Midwives’ Voices, Midwives’ Demands (MVMD), which queried more than 56,000 midwives in 100 countries about what they most need in their role as a midwife. Both campaigns used an open-ended question and both leveraged community mobilizers to collect responses. Demands for both campaigns were analyzed via hand-coding by trained WRA staff and artificial intelligence.

**Results**

Top-line analysis revealed women and midwives want the same things. Top WWW demands were for functional facilities, increased and better supported health providers, including midwives, and respectful and dignified care. Top MVMD demands were for more and better supported personnel and supplies and functional facilities. WRA supported country partners to advocate for policy and resource changes reflecting these demands. In Kenya, demands informed the National Nursing and Midwifery Policy (2022), which incorporates midwives’ requests for better remuneration and increased supplies and facility-level investments to enable midwives to deliver quality care. In Niger State, Nigeria, demands led to the hiring of 100 additional midwives and the inclusion of respectful maternity care and related provider training in its Quality of Care Framework. In Malawi, demands spurred the hiring of 3,959 midwives from 2018 to 2021 and the creation of leadership positions.

**Conclusions**

When providers and women feel heard and supported, providers are in a better position to offer respectful, compassionate, quality care and women are more likely to have a positive experience. WWW and MVMD has shown how to work both sides of the equation to improve the care experience.

## F19.1. Measurement issues and implications for improvements in stillbirth rates from retrospective surveys in low-income settings

### Li Liu^1^, Ane Baerent Fisker^2^, Lucia Hug^3^

#### ^1^Johns Hopkins Bloomberg School of Public Health; ^2^University of Southern Denmark; ^3^United Nations Children’s Fund

*BMC Proceedings 2024*, **18(5):**F19.1

Submission ID #: IMNHC1336

Panel ID# (if applicable): DPYAV411336

**Panel Description**

Stillbirth data are routinely collected through retrospective household surveys, e.g., the Demographic and Health Surveys, in low-income settings. However, such data are subject to a number of known biases, including those due to misclassification with neonatal deaths and retrospective self-reporting. Previous research has shown that the extent and direction of misclassification vary across context. Reproductive-aged women, the respondents of retrospective surveys, do not always have accurate information on their babies’ birth outcomes as they are not consistently informed by health care providers during and after delivery. Moreover, little is known about the under-reporting associated with retrospective surveys in stillbirths. This pre-formed panel offers new insights into these evidence gaps through three studies from Guinea-Bissau and Tanzania using mixed methods. Results from these studies could not only inform the interpretation and adjustment of retrospective survey and model-based stillbirth estimates for better progress tracking, but also identify suboptimal quality of care issues in delivery and postpartum care to further improve maternal and newborn outcomes. The pre-formed panel was co-organized by Johns Hopkins University, the Bandim Health Project, and the University of Southern Denmark.

## F19.2. Incomplete recall of stillbirths: assuming full information on birth outcomes underestimates stillbirth rates compared with prospective data

### Andreas Jensen^1^, Li Liu^2^, Ane Baerent Fisker^1^, Sanne Marie Thysen^3^, Oides Furtado^4^, Claudino Correia^4^, Jacob Von Bornemann Hjelmborg^1^

#### ^1^University of Southern Denmark; ^2^Johns Hopkins Bloomberg School of Public Health; ^3^Bispebjerg and Frederiksberg Hospital; ^4^INDEPTH Network

*BMC Proceedings 2024*, **18(5):**F19.2

Submission ID #: IMNHC1382

Panel ID# (if applicable): DPYAV411336

**Background**

Stillbirth estimates from low- and middle-income countries are most often based on retrospective survey data. However, these data may underestimate the stillbirth rate (SBR) if women omit children who died from their pregnancy histories.

**Methods**

Bandim Health Project’s national representative Health and Demographic Surveillance System (HDSS) in Guinea-Bissau follows an open cohort of women of fertile age through routine home visits every one, two, or six months (depending on area and year). Since the first HDSS visits in 1990, the project has registered all pregnancies and births of children to be followed prospectively and followed up on previously registered pregnancies and births. Since 2010, we have also registered births to women under surveillance even if the births were not to be followed prospectively. For births registered between 2012 and 2020, we estimated the SBR (a) using the standard Bandim Health Project method including only prospectively followed pregnancies and (b) assuming full information on all births, thus estimating the SBR based on both prospectively and retrospectively registered births.

**Results**

We registered 24,890 children before birth (23,432 live births, 1,458 stillbirths). An additional 4,805 births (4,676 live births, 129 stillbirths) were registered retrospectively. Overall, the SBR was 58.6 (55.7–61.5) per 1,000 births for the prospective method and 53.4 (50.9–56.0) for the method assuming full information. Thus, assuming full information significantly underestimated the SBR by 5.2 (1.3–9.0) stillbirths per 1,000 births (adjusted risk ratio (aRR)=0.91 (0.90–0.92)) The entire difference in rates was limited to the cohort with visits every six months: aRR=0.85 (0.83–0.87) while there was no difference when households were visited every two months (aRR=1.01 (0.97–1.04)) or one (aRR=1.01 (0.98–1.03)) month. The estimated SBRs did not change much over the eight-year period.

**Conclusions**

Adding the retrospectively registered births to the cohorts underestimated the SBR by 9% (8%–10%) overall and 15% (13%–17%) for six-monthly visits. Underestimation is presumably greater with longer interval between household visits.

## F19.3. Identification and assessment of bidirectional stillbirth and neonatal death misclassifications in Tanzania: implications for survey-based mortality estimates

### Alexandria Mickler^1^, Li Liu^2^, Henry Kalter^2^, Alain Koffi^2^

#### ^1^United States Agency for International Development; ^2^Johns Hopkins Bloomberg School of Public Health

*BMC Proceedings 2024*, **18(5):**F19.3

Submission ID #: IMNHC1374

Panel ID# (if applicable): DPYAV411336

**Background**

Accurate measurement of perinatal and neonatal deaths is threatened by misclassification errors in household surveys, the primary data source in high-burden areas. Using linked household surveys, we assessed the incidence, directionality, and correlates of misclassified stillbirths and neonatal deaths in Tanzania.

**Methods**

We used data from the 2015–2016 Tanzania Demographic and Health Survey (TDHS) and the 2016–2017 Tanzania Verbal and Social Autopsy (TVASA) study. The TVASA re-sampled all under-5 deaths identified through the TDHS. A total of 428 stillbirth and neonatal death records were linked. The primary outcome of misclassification was assessed by comparing the death status from the TDHS full birth history to the reference standard of the TVASA, which asked additional questions about vital signs. Using weighted bivariate, multivariate, and multinomial logistic regressions, we assessed sociodemographic, pregnancy/delivery, and neonate characteristics against bidirectional death misclassification. Statistical significance was set at *p*<0.10.

**Results**

Among 428 stillbirths and neonatal deaths, 10.3% (*n*=44) were misclassified. Among stillbirths, 15.7% were misclassified by the TDHS as neonatal deaths; and 5.9% of neonatal deaths were misclassified as stillbirths. Bivariate analyses suggest that misclassification was more common among intrapartum stillbirths than neonatal deaths due to communicable causes, and among women attended to by a doctor, those in higher wealth quintiles, those with fewer antenatal contacts, and those living in urban areas. Multivariate analyses indicate the misclassification is significantly associated with cause of death, provider type, and the absence of birth abnormalities.

**Conclusions**

Our results show that the two-way misclassifications do not necessarily cancel each other out. They also highlight factors associated with misclassification and suggest adjustments to Demographic and Health Surveys-based stillbirth and neonatal mortality estimates. We identified maternal and neonatal factors associated with bidirectional misclassification, indicating areas where the quality of pregnancy and delivery care could be improved. This study builds upon a limited body of misclassification research as the first to detect and assess factors associated with bidirectional misclassification. Further validation studies are critical to optimizing survey estimates, deepening understanding of two-way misclassification, and improving tracking toward global goals.

## F19.4. Classification of perinatal deaths as stillbirths or early neonatal deaths differs between retrospective surveys and prospective surveillance: an assessment and contribution of the lack of provider-mother communications from Guinea-Bissau

### Sabine Margarete Damerow^1^, Li Liu2, Diana Yeung^3^, Justiniano Martins^4^, Ishaan Pathak^3^, Ane Baerent Fisker^1^

#### ^1^University of Southern Denmark; ^2^Johns Hopkins Bloomberg School of Public Health; ^3^Johns Hopkins University; ^4^INDEPTH Network

*BMC Proceedings 2024*, **18(5):**F19.4

Submission ID #: IMNHC1346

Panel ID# (if applicable): DPYAV411336

**Background**

In 2021, 2.3 million children died within the first month of life. To inform child survival programs, accurate classifications of stillbirths and early neonatal deaths (ENNDs) are pivotal. So far, the results on magnitude and direction of misclassification have been mixed, and little is known about the mechanisms underlying misclassification.

**Methods**

We conducted an explanatory sequential mixed-methods study to examine characteristics and magnitudes of misclassifications between stillbirths and ENNDs, and their associations with provider-mother communications in Guinea-Bissau. In the case-control component, we compared the characteristics of 63 cases (discordant ENND/stillbirth classification in two previously collected and linked data sources) and 215 controls (concordant classification) using logistic regressions. We also conducted 14 in-depth interviews with women and five focus group discussions with providers and performed a thematic iterative analysis to understand how communications between providers and mothers are associated with the misclassification.

**Results**

Eighty-three percent of the study births occurred in health facilities, but health care providers only informed 33% of the mothers about the vital status of these births. The proportion of misclassification is higher among facility births (24%) than home births (15%), OR=1.77 (0.75–4.18), and when the vital status was provided by health care providers than not, OR=1.36 (0.76–2.44). In the case-control component, women not knowing whether their child had died pre-, peri-, or postpartum (9% of facility births, 2% of home births) also are more likely to have a misclassification: OR=1.91 (0.72–5.06). The qualitative results show that among women informed by providers of their newborn’s vital status, many were unsure about the timing of the deaths. Additional analyses of the association between misclassification and provider-mother communications are underway.

**Conclusions**

Misclassification between stillbirths and ENNDs appears to be common and may differ by place of birth and provider communication content and styles. A nonnegligible proportion of women reported not knowing their child’s time of death, hampering accurate classification based on retrospective surveys. To inform child survival policies and programs, more knowledge on magnitude and determinants of misclassification is needed.

## F20.1. Emerging opportunities for improving the availability and quality of maternal and newborn health data

### Barbara Rawlins^1^, Gabriela Escudero^2^, Louise Tina Day^3^

#### ^1^United States Agency for International Development; ^2^University of North Carolina at Chapel Hill; ^3^London School of Hygiene & Tropical Medicine

*BMC Proceedings 2024*, **18(5):**F20.1

Submission ID #: IMNHC1079

Panel ID# (if applicable): DQKNH371079

**Panel Description**

High-quality data are essential to improve the quality and responsiveness of maternal and newborn health (MNH) services, yet significant gaps exist in the availability and quality of relevant data. This panel will cover three initiatives to strengthen measurement, address gaps in use of MNH indicators in national routine health information systems (RHISs), and examine quality of care (QoC) across the MNH continuum. The first paper describes a landscape analysis of indicators, including indicators for safe surgery for obstetric care and fistula in seven countries’ RHISs. This landscape analysis identifies indicators currently collected and reported on to better understand QoC at the time of delivery and provides country-specific recommendations for safe surgery. The second paper features country experiences implementing clinical vignettes among clinicians in the Democratic Republic of the Congo and Nigeria as a promising QoC survey measurement tool. The clinical vignettes aim to benchmark provider performance against national MNH guidelines, specifically for malaria in pregnancy, hypertension in pregnancy with signs of gender-based violence, pre-eclampsia, and fetal malpresentation. The final innovation discusses the experience in developing and piloting the Every Newborn-Measurement Improvement for Newborn and Stillbirth Indicators tools. These tools are designed to strengthen country newborn and stillbirth RHIS data to close the gap in quality and use of these data. The three papers will be followed by an interactive question-and-answer session that will dig further into experiences with these emergent measurement methods and their potential for wider application.

## F20.2. Every Newborn-Measurement Improvement for Newborn and Stillbirth Indicators (EN-MINI) tools for routine health information systems

### Jacqueline Minja^1^, Louise Tina Day^2^, Harriet Ruysen^2^, Donat Shamba^1^, Josephine Shabani^1^, Getrud Joseph Mollel^1^, Caroline Shayo^1^, Kimberly Peven^2^, Shema Mhajabin^3^, Honorati Masanja^1^, Ahmed Ehsanur Rahman^3^, Shafiqul Ameen^3^, Tamanna Majid^3^, Barbara Knittel^4^, Gabriela Escudero^5^

#### ^1^Ifakara Health Institute; ^2^London School of Hygiene & Tropical Medicine; ^3^International Centre for Diarrhoeal Disease Research, Bangladesh; ^4^John Snow, Inc.; ^5^University of North Carolina at Chapel Hill

*BMC Proceedings 2024*, **18(5):**F20.2

Submission ID #: IMNHC1099

Panel ID# (if applicable): DQKNH371079

**Background**

Timely and accurate core indicator data are essential to track progress toward ending preventable stillbirths, newborn deaths, and disabilities. Routine health information systems (RHIS) have potential, but an evidence gap for data quality in high-burden settings impedes data use for every newborn to survive and thrive. The purpose of the EN-MINI Tools is to enable countries to improve RHIS data for action in support of the Every Newborn Action Plan. The EN-MINI Tools include: (1) MAP newborn indicator availability, (2) Assess USE of newborn data for decisions, and (3) Identify actions to IMPROVE newborn data quality.

**Methods**

Every Newborn – Birth Indicators Research Tracking in Hospitals (EN-BIRTH) phase 2 researchers designed the EN-MINI Tools. These include a novel indicator mapping tool and adapted Performance of Routine Information System Management (PRISM) tools with open-access digital data collection and analysis platforms. The EN-MINI tools were piloted in public health facilities and district data offices in Tanzania (Tanga and Pangani districts) and Bangladesh (Kushtia district). Respondents included health and data professionals recording, reporting, analyzing, and using newborn/stillbirth data.

**Results**

Most newborn/stillbirth core indicators were available in national electronic RHIS (District Health Information Software 2 [DHIS2]) in Tanzania and Bangladesh. We found in in Tanzania that facility register and summary form completeness varied and gaps existed in district feedback mechanisms. Health workers described low motivation for RHIS tasks and high data burden from duplication and limited actionable discussion at supervisory visits.

Data quality assurance processes were lacking at facility and district levels. Timeliness of receiving reports varied. Data were accurately entered into DHIS2, 100% for 9 among10 indicators assessed. RHIS organizational factors and data use for decision-making was stronger at district level, e.g., data visualization. Respondents’ data confidence was higher than data competence.

**Conclusions**

The novel EN-MINI tools pilot identified strengths and weaknesses in RHIS performance for newborn/stillbirth core indicator data at district and health facility levels. Priority actions to improve data quality and data use include improving data culture in health facilities to enable health worker motivation to capture high-quality data. Pilot testing informed EN-MINI tools and training revisions. The EN-MINI tools are free, easy to use, and generate automated reports for subnational and national use to close the gap for newborn/stillbirths data quality and use.

## F20.3. Using clinical vignettes to assess maternal and newborn health provider knowledge and practice: experience from the Democratic Republic of the Congo (DRC) and Nigeria

### Emmanuel Adegbe^1^, Gabriela Escudero^1^, Kristen Brugh^1^, Janna Wisniewski^2^, Sian Curtis^1^, Paul-Samson Lusamba-Dikassa^3^, Isobo Ekesunnie^4^, Jessica Fehringer^1^

#### ^1^University of North Carolina at Chapel Hill; ^2^Tulane University School of Public Health and Tropical Medicine; ^3^Tulane International at Kinshasa School of Public Health; ^4^Data Research and Mapping Consult

*BMC Proceedings 2024*, **18(5):**F20.3

Submission ID #: IMNHC1092

Panel ID# (if applicable): DQKNH371079

**Background**

Clinical vignettes (CVs) are a promising process quality of care (QoC) measurement tool for large-scale application. The objective of this research is to assess the ability of CVs to benchmark provider performance against national maternal and newborn health (MNH) guidelines and provide sufficient discrimination to determine actionable results. We examine a scoring methodology and analyze longitudinal MNH QoC data to develop recommendations for use of CVs.

**Methods**

Data are from nonexperimental evaluations conducted in DRC and Nigeria. CVs were administered to clinicians in 2019 (DRC) and 2021 (Nigeria and DRC). In Nigeria, CVs addressed malaria in pregnancy and hypertension in pregnancy with signs of gender-based violence, and were administered to 664 providers of antenatal care services from 360 primary health facilities in three states.

In DRC, CVs addressed pre-eclampsia (*n*=627) in 2019 and fetal malpresentation (*n*=521) in 2021, and were administered in six provinces. Both evaluations employed descriptive analyses of single-point process QoC indicators and examined associations between CV results and provider/facility characteristics. The Nigeria evaluation also applied a scoring methodology.

**Results**

The vignettes were well-received by clinicians. Providers in Nigeria performed well in testing, diagnosing, and treating malaria in pregnancy. The antenatal care vignette for hypertension in pregnancy did not perform well in distinguishing between hypertension in pregnancy and pre-eclampsia. Providers did not perform well in identifying risk of gender-based violence. In DRC, diagnosis rates for fetal malpresentation were low at 22%. There were also large differences in quality between provinces.

**Conclusions**

CV data can provide insights on the extent to which clinical guidelines are followed and common missteps in providers’ process of MNH service provision. Three lessons emerged: (1) an expert consultative process and pretesting are critical; (2) high frequencies of “other” responses increased data processing burden; and (3) MNH process QoC results should be interpreted alongside structural QoC and MNH outcome metrics.

## F20.4. What are we missing? Safe surgical indicators for maternal Health in National Health Management Information Systems (HMISs): findings of a landscape analysis

### Karen Levin^1^, Gabriela Escudero^2^, Oona Campbell^3^, Louise Tina Day^3^, Farhad Khan^1^, Renae Stafford1, Maxine Pepper^3^

#### ^1^EngenderHealth; ^2^University of North Carolina at Chapel Hill; ^3^London School of Hygiene & Tropical Medicine

*BMC Proceedings 2024*, **18(5):**F20.4

Submission ID #: IMNHC1089

Panel ID# (if applicable): DQKNH371079

**Background**

Baseline assessment and co-creation activities within the U.S. Agency for International Development-funded MOMENTUM Safe Surgery in Family Planning and Obstetrics project confirmed that lack of timely, relevant information at the country level is a persistent barrier in responding to shifting community and facility needs. Routine monitoring and evaluation systems are not currently capturing adequate data on quality of care and morbidity outcomes. As a result, MOMENTUM Safe Surgery initiated a comprehensive analysis, in project implementation countries, of indicators included in national HMISs and national monitoring and evaluation frameworks for surgical maternal health care to identify gaps and opportunities for advocacy for inclusion of key indicators in future HMIS revisions.

**Methods**

In 2022, a landscape analysis was conducted using comprehensive national HMIS data element and indicator lists for seven project implementation countries (Democratic Republic of the Congo, India, Nigeria, Mali, Mozambique, Rwanda, and Senegal) to (1) identify, by country, what safe surgery indicators were currently being collected and reported for obstetric care and fistula, (2) highlight commonalities and gaps across implementation countries, and (3) provide country-specific advocacy guidance for inclusion of missing key indicators in future HMIS revisions.

**Results**

Findings of the landscape analysis present a comprehensive picture of the current safe surgery indicators being captured in national HMISs in MOMENTUM Safe Surgery implementation countries, highlighting key indicators that are being adequately captured as well as those that are missing from national systems. Indicators that are included but for which data is of exceptionally poor quality or missing are also highlighted. Priority maternal health indicators for HMIS expansion that have already been identified by in-country actors or the project are noted.

**Conclusions**

To optimize national, district, and facility capacity to monitor the availability and quality of maternal health surgical services, and to ensure adequate human resources and budgeting to address service needs, it is crucial to have accurate facility-level data reported consistently into the national HMIS. The findings from this landscape analysis can be translated into concrete advocacy for future inclusion of data elements and indicators currently missing from national systems.

## F21.1. An evidence review of four small and sick newborn care coverage indicators for measurement in Routine Health Information Systems (RHISs)

### Harriet Ruysen^1^, He Tang^2^

#### ^1^London School of Hygiene & Tropical Medicine; ^2^World Health Organization

*BMC Proceedings 2024*, **18(5):**F21.1

Submission ID #: IMNHC1186

Panel ID# (if applicable): DRBEQ411186

**Panel Description**

The Ending Preventable Maternal Mortality (EPMM) and Every Newborn Action Plan (ENAP) partnership includes a mandate for national targets of fewer than 12 newborn deaths per 1,000 live births and fewer than 12 stillbirths per 1,000 live births by 2030. Given that around two-thirds of newborn deaths are associated with intrapartum complications, preterm birth, and infection, the plan includes a priority for improving coverage measurement of neonatal resuscitation, kangaroo mother care, antibiotic treatment of severe neonatal infections, and antenatal corticosteroids. A growing body of evidence suggests there are potential opportunities for more accurately capturing data on complex clinical interventions in RHISs, rather than population-based surveys. In response, we completed an evidence review for the measurement of these four indicators in RHISs. Linked with the updated EPMM/ENAP Measurement Improvement Roadmap, this panel will provide a deep dive into the availability and quality of RHIS data for action. We have less than seven years remaining to meet Sustainable Development Goal targets for ending preventable maternal and newborn deaths and stillbirths. We cannot succeed without improving the availability and quality of data for local use for these four high-impact interventions. The three abstracts in this panel aim to galvanize country-level data use for action, and outline the process and findings for each indicator. The panel discussion includes representatives from across the globe to discuss their experiences of how together, we can overcome barriers to quality and use of RHIS data.

## F21.2. An evidence review of four small and sick newborn care coverage indicators for measurement in Routine Health Information Systems (RHISs): what does this mean for program implementation and what should we do next?

### Harriet Ruysen^1^, He Tang^2^, Shaimaa Ibrahim^3^, Felix Bundala^4^, Isabella Sagoe-Moses^5^, Lily Kak^6^, Joy Lawn^2^

#### ^1^London School of Hygiene & Tropical Medicine; ^2^World Health Organization; ^3^United Nations Children’s Fund; ^4^Ministry of Health, Tanzania; ^5^Ghana Health Service; ^6^United States Agency for International Development

*BMC Proceedings 2024*, **18(5):**F21.2

Submission ID #: IMNHC1206

Panel ID# (if applicable): DRBEQ411186

**Background**

Our recommendations for measuring coverage of newborn resuscitation, kangaroo mother care, antibiotics for newborn infections, and antenatal corticosteroids in RHISs are based on evidence review and expert consultation. We found potential opportunities for more accurately capturing data on these four interventions in RHISs, rather than population-based surveys.

This dynamic panel session showcases country-led work to create and implement targets and metrics, with improved measurement for these critical interventions.

**Methods**

Moderated by Prof. Joy Lawn, panelists from around the world will share their experiences of improving data quality, enabling better RHIS data use, and addressing barriers to local use of these four coverage indicators in Tanzania, Ghana, and in some humanitarian settings.

**Results**

We have invited panelists representing national and global policymakers, donors, and country leaders to share real-world examples of improving RHIS data collection and use. Key discussion topics will include:

- Improving RHIS data quality and use: implementation of the Every Newborn-Measurement Improvement for Newborn and Stillbirth Indicator (EN-MINI) tools (Dr Felix Bundala, Tanzania Ministry of Health)
- RHIS data availability in humanitarian settings: what can we measure now and what are the key gaps (Dr Shaimaa Ibrahim, United Nations Children’s Fund, Iraq)
- What can be done next for scale-up: data systems, technology, tools, and indicator prioritization (Dr Barbara Rawlins, U.S. Agency for International Development)

**Conclusions**

With less than seven years to meet Sustaniable Development Goal targets, improving the coverage and quality of newborn resuscitation, kangaroo mother care, antibiotics for newborn infections, and antenatal corticosteroids is a priority. However, we cannot succeed without improving the availability and quality of RHIS data for local use, quality improvement, and service management. Experiences from Tanzania show the link between RHIS data quality and use. In RHISs, source data for these four indictors is aggregated from registers and requires standardized agreement on data elements. Increasing use of digital technologies will allow scale-up of individually linked systems with further opportunities for tracking measures of effective coverage. Panelists will share their insights into how new tools and innovations can be used in concert to improve the availability, quality, and use of RHIS data to drive progress.

## F21.3. Improving measurement of Antenatal Corticosteroids (ACS) and antibiotics for neonatal infections in Routine Health Information Systems (RHISs)

### He Tang^1^, Harriet Ruysen^2^, Ahmed Ehsanur Rahman^3^, Ayesha De Costa^1^, Samira Aboubaker^1^, Tina Lavin^1^, Shams El Arifeen^3^

#### ^1^World Health Organization; ^2^London School of Hygiene & Tropical Medicine; ^3^International Centre for Diarrhoeal Disease Research, Bangladesh

*BMC Proceedings 2024*, **18(5):**F21.3

Submission ID #: IMNHC1201

Panel ID# (if applicable): DRBEQ411186

**Background**

Every year, more than 10% of newborns are born preterm, and this number is rising. Prematurity and neonatal infections are leading causes of under-5 deaths. The Every Newborn Action Plan (ENAP) framework has adopted ACS and antibiotic treatment of newborn infections as indicators. Both need to be refined for measurement in RHISs.

**Methods**

We synthesized published evidence including a scoping review regarding validation of indicator numerators and denominators in RHISs for tracking coverage of ACS and antibiotic treatment of neonatal infections. Expert groups reviewed these findings and provided recommendations.

**Results**

To measure ACS initiation coverage, we recommend using the number of women who delivered between 24 and 34 weeks’ gestation and received at least one dose of ACS (numerator), divided by the number of women who delivered between 24 and 34 weeks’ gestation (denominator). There are some challenges in measuring this indicator, including obtaining accurate gestational assessment and other obstetric information. Data for ACS coverage may be captured in individually linked clinical records, but would be more challenging in registers/tally sheets. Contextual factors are important.

To calculate coverage of antibiotic treatment for newborn infection using RHIS data, we recommend the following numerator: number of newborns identified as infection cases who also received at least two days of appropriate injectable antibiotics, including cases of possible serious bacterial infection (critical illness or clinical severe infection) in outpatient settings or clinically suspected sepsis in inpatient settings. The denominator is the number of identified infection cases in both inpatient and outpatient settings.

For antibiotic treatment of newborn infections, work is needed to refine case definitions, link more than one dose, and operationalize combining data from inpatient and outpatient systems.

**Conclusions**

RHISs usually involve aggregate data from registers and tally sheets, which are likely to be challenging to collect for ACS and neonatal infection treatment. Individually linked datasets, drawing from patient notes, have potential to capture these indicators, e.g., in a core neonatal inpatient dataset on a newborn ward for infection treatment, or a maternity ward for ACS.

## F21.4. Improving measurement of neonatal resuscitation and Kangaroo Mother Care (KMC) in Routine Health Information Systems (RHISs)

### He Tang^1^, Harriet Ruysen^2^, Ashish KC^3^, Suman Rao^1^

#### ^1^World Health Organization; ^2^London School of Hygiene & Tropical Medicine; ^3^Uppsala University

*BMC Proceedings 2024*, **18(5):**F21.4

Submission ID #: IMNHC1191

Panel ID# (if applicable): DRBEQ411186

**Background**

Every year, 2.4 million neonates die, with preterm birth and intrapartum complications the leading causes of under-5 deaths. KMC and newborn resuscitation are high-impact interventions promoted by the Every Newborn Action Plan (ENAP) as necessary for achieving Sustainable Development Goal targets. Coverage indicators are key and have been validated in Every Newborn – Birth Indicators Research Tracking in Hospitals (EN-BIRTH) study, but need to be clearly defined and adopted for measurement in RHISs.

**Methods**

We synthesized published evidence in a scoping review regarding validation of indicator numerators and denominators in RHISs for tracking coverage of neonatal resuscitation and KMC. Expert groups reviewed these findings and provided recommendations.

**Results**

For KMC coverage in RHISs, we recommend the numerator, number of newborns with birthweight <2500g initiated in kangaroo position (anywhere in a facility), divided by total admitted newborns with a birthweight <2500g (denominator). Kangaroo position is defined as a baby placed skin-to-skin in an upright position with the caregiver. Additionally, quality of KMC metrics could be added with individually linked data regarding KMC duration and exclusive breastfeeding. Improvements in gestational age metrics are required.

For newborn resuscitation coverage, we recommend the following numerator: number of newborns receiving positive pressure ventilation (PPV) (e.g., bag-and-mask ventilation) in the delivery room. The recommended denominator is total number of live births plus stillbirths. Evidence shows that RHIS data collection for the denominator, newborns who require resuscitation, has low accuracy and is challenging to collect. As with interpretation of cesarean section rates, it is important to understand if the newborns who require resuscitation, receive resuscitation. Benchmarking the proportion of newborns anticipated to require PPV at birth with the type of cases received in each facility could be helpful.

**Conclusions**

These coverage indicators are commonly captured by registers and/or tally sheets and are feasible in many countries. Individually linked data from clinical notes would add detail but requires reliable bedside digital technologies. Countries adapting their RHISs will be able to monitor scale-up linked to ENAP.

## F21.5. An evidence review of four small and sick newborn care coverage indicators for measurement in Routine Health Information Systems (RHISs): why does it matter and what did we do?

### He Tang^1^, Harriet Ruysen^2^

#### ^1^World Health Organization; ^2^London School of Hygiene & Tropical Medicine

*BMC Proceedings 2024*, **18(5):**F21.5

Submission ID #: IMNHC1187

Panel ID# (if applicable): DRBEQ411186

**Background**

Linked with the updated Ending Preventable Maternal Mortality/Every Newborn Action Plan (EPMM-ENAP) Measurement Improvement Roadmap, we reviewed the evidence for measurement in RHISs of antenatal corticosteroids use, neonatal resuscitation, kangaroo mother care, and antibiotic treatment of severe neonatal infections in low- and middle-income countries.

Our objectives were to:

- Synthesize evidence for measurement in RHISs of these four indicators.
- Update recommendations for numerators and denominators specific to RHISs.
- Consider additional measures of effective coverage in RHISs.
- Identify outstanding research gaps for measurement in RHISs of these four indicators.

**Methods**

Convened by the World Health Organization (WHO) (2020–2022), a technical steering group of maternal and newborn health (MNH) measurement experts appointed four intervention-specific subgroups to synthesize evidence from our scoping review. The review (October 2020–March 2021) aimed to identify validated RHIS measures for antenatal corticosteroids use, neonatal resuscitation, kangaroo mother care, and antibiotic treatment of severe neonatal infections in low- and middle-income countries. Data regarding indicator definitions (numerators, denominators), data sources, and details of indicator testing were extracted and submitted to the subgroups. The subgroups assessed if indicators were action focused, important, measurable, simple, and valued using pre-defined criteria adapted from the WHO consultation improving measurement of the quality of MNH measurement in health facilities (2013).

The findings were referenced against WHO clinical care guidelines, standards, and the Every Newborn Action Plan framework. Each subgroup undertook technical work to agree on recommendations regarding the definition (numerator/denominator), potential RHIS data sources, and research priorities.

Recommendations were reviewed in consultation with the MoNITOR group, a WHO-led initiative including more than 80 partners, aiming to provide technical support and coordination for efforts to strengthen MNH measurement (July 2022).

**Results**

The Every Newborn – Birth Indicators Research Tracking in Hospitals study included validation of measurement in RHISs for three of the indicators. Results are in 'Results are presented in the two related abstracts showing recommendations for:

- What can be measured now in a RHIS:
  - ◦ Indicator definition
  - ◦ Data source
- What are measurement gaps?
- What next to improve RHIS data availability and quality?

**Conclusions**

RHIS count data have potential for near real-time use to inform health service planning. However, to avoid overburdening the least resourced systems, we need to assess which data can be realistically collected for accurate measurement of which indicators.

## F22.1. Global guidance to country implementation: country experiences with scaling up small or sick newborn care to achieve Every Newborn Action Plan (ENAP) target 4

### Lily Kak^1^, Gagan Gupta^2^, Felix Bundala^3^, Bibek Kumar Lal^4^, Tom Sesay^5^

#### ^1^United States Agency for International Development; ^2^United Nations Children’s Fund; ^3^Ministry of Health, Tanzania; ^4^Ministry of Health and Population, Nepal; ^5^Ministry of Health and Sanitation, Sierra Leone

*BMC Proceedings 2024*, **18(5):**F22.1

Submission ID #: IMNHC517

Panel ID# (if applicable): DRPGF68517

**Panel Description**

Despite impressive reductions in newborn mortality rate (NMR) across the globe, advances are not equal among countries. A Lives Saved analysis revealed the impact of inpatient care of small and sick newborns (SSNB) to be the biggest in averting newborn mortality. Yet, 80% of the 30 million newborns that require inpatient care each year do not receive it. To address this critical gap, ENAP added a fourth target on SSNB for countries to achieve by 2025: 80% of countries have a national implementation plan for SSNB care and 80% of districts have a functional level 2 inpatient unit to care for SSNB with respiratory support, including continuous positive airway pressure. To guide countries’ efforts toward achieving this target, the World Health Organization and the United Nations Children’s Fund (UNICEF) have developed standards for improving the quality of care for SSNB in health facilities and a model of level 2 care.

This session will bring together global and country partners to discuss the global guidance and country implementation of SSNB care. The panel will begin with parents who will share their experience as partners caring for their hospitalized newborns. UNICEF will describe the SSNB model of care and share key insights on scale. A moderated panel discussion will follow, with government officials from Bangladesh, Nepal, Rwanda, Sierra Leone, and Tanzania, to delve into components of country-contextualized models of SSNB care and learn from experiences in organizing systems scale-up of services.

## F22.2. Scaling up small and/or sick newborn care: implementation experience from countries —Bangladesh, Nepal, Rwanda, Sierra Leone, and Tanzania

### Lily Kak^1^, Felix Bundala^2^, Bibek Kumar Lal^3^, Tom Sesay^4^, Francois Regis Cyiza^5^, Ahmedul Kabir^6^

#### ^1^United States Agency for International Development; ^2^Ministry of Health, Tanzania; ^3^Ministry of Health and Population, Nepal; ^4^Ministry of Health and Sanitation, Sierra Leone; ^5^Rwanda Biomedical Center; ^6^Ministry of Health, Bangladesh

*BMC Proceedings 2024*, **18(5):**F22.2

Submission ID #: IMNHC521

Panel ID# (if applicable): DRPGF68517

**Background**

Several countries have made remarkable progress in scaling up level 2 small and sick newborn (SSNB) care. Their experiences have helped build consensus on the core components of the models of SSNB care. During this session, government representatives from five forerunner countries will summarize their experiences and share pragmatic aspects of scale-up in implementing special newborn care at level 2 facilities.

**Methods**

Countries that have demonstrated progress in scale-up of SSNB care have done so by addressing many of the 10 components of the model of care. During this session, the moderator will guide conversations that will allow countries to deep dive into specific components of their models of SSNB care and summarize key learnings from their experiences.

**Results**

All five countries scaled up SSNB care through strong leadership with nationally endorsed costed plans supported through domestic and partner resources. Sierra Leone has clear norms for staffing, while Bangladesh has proposed multidisciplinary staff teams. Rwanda has developed new cadres and staffing models. All countries have prioritized infrastructure, equipment, and commodities through government plans and budgets, with Tanzania’s procurement and maintenance system being exemplary. Strengthened data systems, functional referral systems within networks of care, and specialized health care workers that grew as systems of special newborn care matured, were other critical areas of focus. All countries started small and learned through implementation in select components of care. They added newer components and missing pieces based on emerging needs as they moved ahead.

**Conclusions**

All five participants in the panel represent low- and middle-income countries that were able to move the needle in newborn care. Their learning experience can support countries that have moved from scaling up basic preventative and promotive newborn care to scaling up essential newborn care and newborn resuscitation, and are now embarking on expanding facility-based newborn care that will ensure they achieve their Every Newborn Action Plan and Sustainable Development Goal targets.

## F22.3. What does it take to scale up facility-based newborn care: lessons from forerunner countries

### Lily Kak^1^, Gagan Gupta^2^

#### ^1^United States Agency for International Development; ^2^United Nations Children’s Fund

*BMC Proceedings 2024*, **18(5):**F22.3

Submission ID #: IMNHC519

Panel ID# (if applicable): DRPGF68517

**Background**

The World Health Organization’s *Standards for Improving the Quality Care of Small and Sick Newborns in Health Facilities* and the model of care provide the framework for countries to adapt and contextualize to strengthen the quality of nurturing small and sick newborn (SSNB) care in level 2 facilities. Several countries have taken global guidance and chalked out a path that has led programs to scale. This presentation summarizes key insights about the model of care and the nuts and bolts of scaling up SSNB care.

**Methods**

Understanding and capturing the learning from countries that are on the leading edge of scaling up SSNB facility care was the key methodology used to determine the critical elements for rolling out a nationally scalable model of care.

**Results**

Ten critical components are needed as per WHO-UNICEF supported model for scaling up care for small and sick newborn. They include long-term vision with a change team guiding and tracking the process at national level; an implementation plan that captures all components of the model of care for pilot testing in multiple geographies across the nation; high-level visibility through advocacy, including political attention and leadership; adequate budget lines for establishment and operating expenses; infrastructure planning and standardization; equipment procurement and maintenance systems; human resource requirements for establishing norms and policies that attract and retain capable staff for provision of care; robust data systems to track performance and outcomes across service delivery points; functional referral system, linkages of maternal and newborn care; a system for post-discharge follow-up care; and engagement of families in care right from the start. This model needs to build on a strong foundation of quality maternal and essential newborn care for all mothers and babies.

**Conclusions**

Scaling up inpatient SSNB care requires working simultaneously on different steps and not sequentially. While there are many implementation challenges, plenty of lessons are emerging from multiple countries that can help inform countries that are considering the rollout of SSNB care.

## F22.4. Our baby: the trials and tribulations of living through the early days of life with our small and sick baby in inpatient care

### Lily Kak

#### United States Agency for International Development

*BMC Proceedings 2024*, **18(5):**F22.4

Submission ID #: IMNHC518

Panel ID# (if applicable): DRPGF68517

**Background**

Every birth is a joyous event as parents come face to face for the first time with their bundle of joy. Yet, parents of newborns can also be tired, overwhelmed, and uncertain as they learn to bond with and care for their babies. The stress escalates for parents of small and sick newborns, especially when separated in inpatient care units. Parents separated from their babies are more likely to experience anxiety and have feelings of helplessness. Involvement of mothers/parents in the care of their small and/or sick newborn improves developmental outcomes for their babies and the well-being of parents and prepares them to assume full responsibility after discharge.

**Methods**

In this session, parents who have lived through the experience of having a small and/or sick newborn in inpatient care will share their first-person stories. They will provide insights on what it means to be parents with small and/or sick newborns.

**Results**

One in six women worldwide experiences signs of depression during the postnatal period, which affects her capacity to care for her baby. Parents of newborns who were small and/or sick have noted that being able to participate as partners in the care of their baby enables them to learn to respond to their baby’s needs. Continuous family access, rooming in, and practicing skin-to-skin care ensures that parents are fully enabled to provide nurturing care to their newborn at all times, leading to developmentally supportive care. The hospital environment influences the experience of care for newborns and their families in inpatient care.

**Conclusions**

Being born too soon or too small, or being sick, necessitates extra care for these more vulnerable newborns. This care is best provided directly by the parents, in close partnership with health care professionals.

## F23.1. Leveraging closer-to-reality travel time estimates from big data to address gaps and achieve equity in geographical accessibility to emergency obstetric care in Sub-Saharan Africa’s largest megacity

### Aduragbemi Banke-Thomas^1^, Bosede Afolabi^2^, Kerry Wong^1^, Babatunde Ajayi^3^, Charlotte Yandell Stanton^4^

#### ^1^London School of Hygiene & Tropical Medicine; ^2^College of Medicine University of Lagos; ^3^Lagos State Ministry of Health; ^4^Google

*BMC Proceedings 2024*, **18(5):**F23.1

Submission ID #: IMNHC98

Panel ID# (if applicable): DTXZD3298

**Panel Description**

This panel, comprising stakeholders from the research community, government, and technology sector, will show how closer-to-reality travel time estimates to emergency obstetric care (EmOC) in sub-Saharan Africa’s largest megacity, Lagos, Nigeria, were generated and used to support EmOC service planning and the development of an innovative digital tool to support future planning.

The panel will start with a premiere of a four-minute animated video titled “Mrs X lives in the city too,” which challenges the depiction of difficulty in EmOC access, in a widely acclaimed 1988 short film, as a problem of poor rural dwellers. The new animation will highlight how traffic and insecurity influence travel to EmOC in urban settings, necessitating the need to rethink existing approaches to travel time estimates.

The panel will then discuss research from the On Tackling In-transit delays for Mothers in Emergency (OnTIME) project, which leverages big data to generate closer-to-reality travel time estimates to EmOC. The panel will show how the project combined primary data on health facility functionality with Google Maps to compute closer-to-reality travel time estimates to the first, second, and third nearest public and private hospitals with EmOC capacity in Lagos. The panel will also discuss the application of the evidence generated in supporting policymakers with service planning and considerations identified by policymakers as essential in developing decision-making supportive tools for future service planning and realization of equity in EmOC geographical accessibility in Lagos and other similar low-resource urban settings.

## F23.2. Considerations for enhancing geographical access to emergency obstetric services in Nigeria using innovative digital technology: insights from policymakers

### Aduragbemi Banke-Thomas^1^, Ibukun-Oluwa Abejirinde^2^, Uchenna Chinenye Gwacham-Anisiobi^3^, Olakunmi Ogunyemi^4^, Tope Olubodun^5^

#### ^1^London School of Hygiene & Tropical Medicine; ^2^Women’s College Hospital; ^3^University of Oxford; ^4^OnTIME Consortium; ^5^Federal Medical Centre Abeokuta

*BMC Proceedings 2024*, **18(5):**F23.2

Submission ID #: IMNHC164

Panel ID# (if applicable): DTXZD3298

**Background**

Effective organization of health services ensures that patients requiring emergency services receive timely care that matches their clinical needs and preferences. Recognizing that digital interventions using big data and geographic information systems play a significant role in health care planning and delivery, more so in the era of the United Nations Sustainable Development Goals, our objective in this study was to understand key considerations for optimizing digital technology to support policy decisions on the location of emergency obstetric services.

**Methods**

Twenty-two semi-structured interviews were conducted with policymakers and civil servants involved in health services planning at Nigeria's state and federal government levels. The key informant interviews were conducted in person or virtually to suit the preference and availability of the interviewee. Braun and Clarke’s six-step approach used in this study involved familiarizing ourselves with the data, genrating the initial code, searching for themes, reviewing themes, defining and naming themes, and producing the report.

**Results**

Study findings showed that current considerations for locating service delivery points included persistent community advocacy, identified needs, political pressure, and timely opportunities. To varying extents, decision-makers reported relying on data for service location. Importantly, there was unanimous interest in using big data and technology for evidence-based decision-making. A digital dashboard that is dynamic, reflective of reality, inclusive of public and private providers, incorporates facility characteristics, and is able to test scenarios was deemed particularly valuable. However, the value proposition for using a dynamic digital dashboard extended beyond emergency obstetric care, as stakeholders recognized general challenges to health care access in urban settings and made recommendations for additional data that could be built into the proposed platform to make it more relevant for policy and sustainable.

**Conclusions**

Policymakers recognize and welcome opportunities to leverage big data and technological solutions to inform their decisions regarding access to emergency services and strengthening service delivery. There is a need for multistakeholder engagement to encourage uptake and drive necessary social and infrastructural changes, including those beyond the health sector. This will ensure that the knowledge to action gap is addressed.

## F23.3. Application of evidence from a closer-to-reality assessment of geographical accessibility in planning emergency obstetric services in the urban state of Lagos

### Aduragbemi Banke-Thomas^1^, Bosede Afolabi^2^, Kerry Wong^1^, Babatunde Ajayi^3^, Charlotte Yandell Stanton^4^, Olakunmi Ogunyemi^4^, Tope Olubodun^5^, Lenka Benova^6^, Ibukun-Oluwa Abejirinde^7^

#### ^1^London School of Hygiene & Tropical Medicine; ^2^College of Medicine University of Lagos; ^3^Lagos State Ministry of Health; ^4^OnTIME Consortium; ^5^Federal Medical Centre Abeokuta; ^6^Institute of Tropical Medicine; ^7^Women’s College Hospital

*BMC Proceedings 2024*, **18(5):**F23.3

Submission ID #: IMNHC163

Panel ID# (if applicable): DTXZD3298

**Background**

Governments only minimally use models for planning emergency obstetric care (EmOC) service provision in low-resource settings, as they do not reflect the reality of travel, including traffic and weather conditions. Building on evidence that conclusively showed that navigation software that leverages big data and geographic information systems offers travel time estimates that are closer to reality in urban low-resource settings and can play an important role in health care planning and delivery, the On Tackling In-transit delays for Mothers in Emergency (OnTIME) project has conducted innovative research that captured evidence to inform geographical accessibility assessment in planning EmOC services.

**Methods**

Effective health service organization ensures that patients requiring emergency services receive timely care that matches their clinical needs and preferences. With clear recognition that digital interventions using big data and geographic information systems play a significant role in health care planning and delivery, more so in the Sustainable Development Goals era, our objective in this study was to understand key considerations for optimizing digital technology to support policy decisions on the location of emergency obstetric services.

**Results**

Evidence from the research conducted by the OnTIME Consortium has been used to affirm the decision of the Lagos State Government on the location of the Epe Maternal and Child Care Centre and supported the decision on situating the new Ojo General Hospital, which now includes a dedicated comprehensive EmOC facility. The evidence has also informed the choice of policy options to support pregnant women during travel to care, including the legalization of the use of bus rapid transit lanes and ambulance deployment. Building on this work, a digital dashboard, which will be demonstrated during the conference technical marketplace, will show different visualizations and scenario-building options that are available to guide EmOC service planning.

**Conclusions**

Application of closer-to-reality assessment of geographical accessibility constitutes the next frontier for evidence-based emergency service planning in urban low-resource settings. If well deployed, it will help to guarantee value for money, realize equitable and responsive health systems, and contribute to global efforts to reduce maternal and perinatal mortality in high-burden countries.

## F23.4. Mapping closer-to-reality travel times to obstetric emergency services in Lagos, Nigeria, using google maps navigation API

### Charlotte Yandell Stanton^1^, Aduragbemi Banke-Thomas^2^, Bosede Afolabi^3^, Kerry Wong^2^, Babatunde Ajayi^4^, Peter Macharia^5^, Tope Olubodun^6^, Prestige Tatenda Makanga^7^, Olakunmi Ogunyemi^8^, Daniel Achugo^9^, Jia Wang^10^, Ibukun-Oluwa Abejirinde^11^, Uchenna Chinenye Gwacham-Anisiobi^12^, Lenka Benova^13^, Mansi Kansal^1^, Narayanan Sundararajan^1^

#### ^1^Google; ^2^London School of Hygiene & Tropical Medicine; ^3^College of Medicine University of Lagos; ^4^Lagos State Ministry of Health; ^5^KEMRI-Wellcome Trust Research Programme; ^6^Federal Medical Centre Abeokuta; ^7^Midlands State University, Gweru; ^8^OnTIME Consortium; ^9^Nnamdi Azikwe University; ^10^University of Greenwich; ^11^Women’s College Hospital; ^12^University of Oxford; ^13^Institute of Tropical Medicine

*BMC Proceedings 2024*, **18(5):**F23.4

Submission ID #: IMNHC162

Panel ID# (if applicable): DTXZD3298

**Background**

Methods commonly used to estimate the travel time to health care facilities have been shown to fall short of reflecting real-life travel time due to the failure to consider traffic and road conditions. The issue is particularly pertinent in highly urbanized settings. Our objective was to use the Google Maps travel time algorithm and data on traffic to compute travel time to the first, second, and third nearest comprehensive emergency obstetric care (EmOC) facility based on driving transport mode across Lagos, Nigeria.

**Methods**

We assembled a geocoded master list of comprehensive EmOC facilities in Lagos from multiple sources and physically verified functionalty of facilities before inclusion. To obtain travel time to the included facilities, we implemented the Google Maps Navigation API for driving to a public, private, or either facility type. We derived travel times during peak and non-peak hours and two weather conditions (dry and rainy). We then obtained high-resolution travel time estimates per S2 cell spanning the entire city of Lagos (each covering 0.06 km^2^ of land area) and triangulated them with auxiliary data on cell-level population counts to obtain comprehensive EmOC accessibility metrics at the population level. We obtained population data from the Worldpop constrained United Nations-adjusted 2020 dataset.

**Results**

Preliminary results suggest that approximately 46% of the population lives within 15 minutes of driving time from the nearest public health facility, with the median driving time to the nearest public comprehensive EmOC being 25 minutes. Further, 16% of the population lives more than 30 minutes of driving time from the nearest public comprehensive EmOC facility. Population-weighted average travel times to the first, second, and third nearest facilities varied at the ward and local government levels. The number and type of comprehensive EmOC facilities that can be reached within the same time thresholds also varied.

**Conclusions**

This innovative approach to travel time estimates and EmOC geographical accessibility assessment will aid in accurately identifying communities and areas that require urgent attention, thereby supporting effective service planning and achieving equity in EmOC access and universal health coverage. The approach should be scaled up in other low-resource urban settings in Africa, Asia, and Latin America.

## F23.5. Experiences of pregnant women accessing emergency obstetric care in Africa’s largest megacity: narratives from pregnant women and their careers

### Bosede Afolabi^1^, Aduragbemi Banke-Thomas^2^, Babatunde Ajayi^3^, Tope Olubodun^4^, Ibukun-Oluwa Abejirinde^5^, Mobolanle Balogun^1^

#### ^1^College of Medicine University of Lagos; ^2^London School of Hygiene & Tropical Medicine; ^3^Lagos State Ministry of Health; ^4^Federal Medical Centre Abeokuta; ^5^Women's College Hospital

*BMC Proceedings 2024*, **18(5):**F23.5

Submission ID #: IMNHC99

Panel ID# (if applicable): DTXZD3298

**Background**

The consequences of delays in travel of pregnant women to reach facilities in emergency situations are well documented in the literature. However, their actual experiences of travel to health facilities when requiring emergency obstetric care (EmOC) remain a “black box” of many unknowns to the health system, more so in megacities of low- and middle-income countries, which are fraught with wide inequalities. A 1988 short film animation produced by the World Health Organization titled “Why did Mrs X die?” which was based on a lecture by Prof. Mahmoud Fathallah titled “The maternal death road,” depicts challenges with EmOC access as a problem for poor rural dwellers. Our objective is to depict the experiences of pregnant women who live in urban Lagos, Nigeria.

**Methods**

An animated video was conceptualized and produced based on insights from qualitative research on the experiences of 47 pregnant women accessing care in Lagos, 11 relatives supporting these women, and five obstetricians working in the obstetric emergency rooms of two of the largest maternity units in Nigeria. Thematic analysis was used to identify core emerging themes to infuse into the new animation.

**Results**

The four-minute animated video titled “Mrs X lives in the city too,” which succinctly reflects the experiences of pregnant women in accessing EmOC in an urban setting in Africa, was premiered at the conference. Issues of poor roads, traffic, especially in the daytime, insecurity and scarcity of public transportation, particularly at night, and lack of ambulance at the time of need were highlighted by respondents and are highlighted in the animated video.

**Conclusions**

The key takeaway message is that pregnant women living in urban areas (city or suburb) and even next to large hospitals in cities also have challenges with accessing care and are not precluded from the risk of adverse outcomes in pregnancy. Indeed, the so-called “urban advantage” might be disappearing in low-resource urban settings such as Lagos. We conclude that there is a need to gather evidence that is closer to reality to support urban planning for health services and reorganize health systems to work for low-resource urban settings.

## F24.1. Maternal and newborn data: what now and what next for Ending Preventable Maternal Mortality/Every Newborn Action Plan (EPMM/ENAP) targets? Measurement improvement roadmap (2023–2030)

### Harriet Ruysen^1^, Allisyn Moran^2^, Joy Lawn^1^

#### ^1^London School of Hygiene & Tropical Medicine; ^2^World Health Organization

*BMC Proceedings 2024*, **18(5):**F24.1

Submission ID #: IMNHC74

Panel ID# (if applicable): DUBIC3274

**Panel Description**

Actions to achieve Sustainable Development Goal 2030 targets for maternal and newborn health (MNH) are supported by ENAP and EPMM partnerships. ENAP and EPMM have launched five joint coverage targets and milestones for tracking progress, and the first joint ENAP/EPMM progress report launches at the International Maternal Newborn Health Conference (IMNHC) in May 2023. Data for action is an important ENAP and EPMM milestone and includes strengthening data systems to better track these targets at subnational, national, and global levels. To this end, the ENAP/EPMM partnership has developed a joint Measurement Improvement Roadmap 2023–2025 to list priority indicators with a focus on ENAP/EPMM targets, identify what to do now to improve data (especially in routine systems), and outline what is next for improving measurement and use of data. The previous ENAP Measurement Improvement Roadmap 2015–2020 resulted in improved data guidance and also large multi-country validation studies to inform measurement change.

In this dynamic session, three presentations cover what to measure now and next for indicators regarding impact, coverage/quality, and service availability. A country-led panel discussion follows. Building on evidence from the 2023 EPMM/ENAP report, this session uses the roadmap to show current MNH data availability and how to drive improvements in data quantity, quality, and utility at local, district, and national levels. Panelists include national policymakers, donors, and thought leaders sharing experiences and realities of how we can transform data for action in MNH and move faster, including what can be achieved by the next IMNHC in 2025.

## F24.2. Improving data for action: what to measure now and what next to best monitor progress toward achieving Ending Preventable Maternal Mortality/Every Newborn Action Plan (EPMM/ENAP) targets? Implementing the new measurement improvement roadmap

### Joy Lawn^1^, Harriet Ruysen^1^, Allisyn Moran^2^, Cheikh Mbacke Faye^3^, Mihretab Salasibew^4^, Dalya Eltayeb^5^, Jennifer Requejo^6^

#### ^1^London School of Hygiene & Tropical Medicine; ^2^World Health Organization; ^3^African Population and Health Research Center; ^4^Children's Investment Fund Foundation; ^5^Federal Ministry of Health, Sudan; ^6^United Nations Children’s Fund

*BMC Proceedings 2024*, **18(5):**F24.2

Submission ID #: IMNHC907

Panel ID# (if applicable): DUBIC3274

**Background**

The original Measurement Improvement Roadmap (2015) was conceived as a five-year plan to improve, institutionalize, and use metrics to accelerate progress for women, newborns, and stillbirths. Now together for change, EPMM-ENAP have updated the roadmap, an important accountability milestone, showcasing progress and building consensus on the prioritization of remaining gaps for maternal and newborn health measurement. During this closing abstract, we will provide an overview of the Measurement Improvement Roadmap (2023–2025) and the implications for implementation. This will include a dynamic panel discussion led by multi-agency partners and country leaders.

**Methods**

Panelists from Countdown, the Children’s Investment Fund Foundation (CIFF), government ministries, and the United Nations Children’s Fund (UNICEF) will share their perspectives and experiences implementing the Measurement Improvement Roadmap.

**Results**

Moderated by Prof. Joy Lawn, the panelists, representing country leaders, program managers, measurement experts, and donors, will share their implementation experiences and how we can improve measurement of the indicators presented in previous abstracts, considering:

- Multi-country routine data quality and capacity in DHIS—Dr. Cheikh Faye (Countdown, African Population and Health Research centre (APHRC))
- Funding perspective—Mihretab Salasibew (CIFF)
- Emergency obstetric and newborn care and maternal and perinatal death surveillance and response in Sudan—Dr. Dalya Eltayeb (Ministry of Health, Sudan)
- Where do we go next for use of data and research?—Dr. Jennifer Requejo (UNICEF)

**Conclusions**

The updated EPMM/ENAP Measurement Improvement Roadmap reviews progress for the development of tools, validation of indicators, and institutionalization of metrics. The roadmap highlights ongoing opportunities for strengthening routine health information systems and scaling up digital innovations and systems for crosslinking different data platforms, and galvanizing progress toward individually linked digital data systems. However, gaps remain for disaggregation at local levels and may impede progress for populations and geographies most at risk of poor health outcomes. Change requires intentional transfer of leadership to the countries with the greatest disease burden, and it is imperative to be led by their experiences and priorities.

## F24.3. Measuring availability of emergency care for every woman and every newborn: what can be measured now and what next toward achieving Ending Preventable Maternal Mortality/Every Newborn Action Plan (EPMM/ENAP) targets?

### Allisyn Moran^1^, Joy Lawn^2^, Harriet Ruysen^2^, Gagan Gupta^3^, Jean Pierre Monet^4^

#### ^1^World Health Organization; ^2^London School of Hygiene & Tropical Medicine; ^3^United Nations Children’s Fund; ^4^United Nations Population Fund

*BMC Proceedings 2024*, **18(5):**F24.3

Submission ID #: IMNHC900

Panel ID# (if applicable): DUBIC3274

**Background**

Increasing service provision of emergency obstetric and neonatal care (EmONC) and small and sick newborn care (SSNC) is essential for countries to reach Sustainable Development Goal targets 3.1 (maternal) and 3.2 (newborn). Inequities to accessing high-quality services persist, with ongoing gaps for investment in many low- and middle-income countries. Joint EPMM/ENAP targets (2020–2025) for EmONC and SSNC are defined at district, national, and global levels. More comparable, yet feasible, tracking tools are required.

**Methods**

In support of EPMM/ENAP targets, evidence synthesis and consultations have been undertaken to review measures, tools, and data sources for EmONC and SSNC. These findings have informed the updated ENAP/EPMM Measurement Improvement Roadmap.

**Results**

What can be measured now?

- EmONC signal functions are being updated in line with advances in maternal and newborn health, including proposals for newborn signal functions.
- *Standards for Improving the Quality of Care for Small and Sick Newborns in Health Facilities* (2020) includes measures for tracking SSNC, some that can be measured in routine health information systems now.

Where do the data come from?

- More than 120 countries are expected to complete the ENAP/EPMM tracking tool (February 2023). This includes self-reported progress toward ENAP target 4 (80% of districts with level 2 SSNC services) and EPMM target 4 (80% of women within two hours travel time of an EmOC facility).
- Few countries have up-to-date assessments due to cost and expertise required for EmOC assessments. In 2017, 21/83 “Countdown countries” reported data for EmONC availability. In 2022, only 12 countries were measuring geographic access of population within two hours of travel time. The United Nations Population Fund is developing an EmOC Lite tool.
- The SSNC United Nations Children’s Fund/NEST360 health facility assessment tool can measure if a facility is operational for World Health Organization level 2 SSNC and takes a day to implement. This tool is open source and has been used in more than 100 facilities in six countries so far.

**Conclusions**

What next?

- Improved and simplified tools for assessing EmONC and SSNC availability with modeling for geographical access.
- Strengthened measures of experience of care.
- Increased use of routine health information systems for assessing the availability, quality, and access to EmONC with individually linked data.

## F24.4. Coverage data for maternal and newborn health: what can be measured now and what next to best monitor progress toward achieving Ending Preventable Maternal Mortality/Every Newborn Action Plan (EPMM/ENAP) targets?

### Allisyn Moran^1^, Joy Lawn^2^, Harriet Ruysen^2^, Jennifer Requejo^3^, Agbessi Amouzou^4^

#### ^1^World Health Organization; ^2^London School of Hygiene & Tropical Medicine; ^3^United Nations Children’s Fund; ^4^Johns Hopkins Bloomberg School of Public Health

*BMC Proceedings 2024*, **18(5):**F24.4

Submission ID #: IMNHC892

Panel ID# (if applicable): DUBIC3274

**Background**

To meet Sustainable Development Goal targets for every woman and every newborn, equitable coverage of high-quality antenatal care (ANC), skilled birth attendance (SBA), and postnatal care (PNC) services are essential. Hence, joint EPMM/ENAP targets (2020–2025) have been agreed for these three indicators at district, national, and global levels. Increases in coverage of services are necessary but alone not sufficient to improve maternal and newborn survival or prevent stillbirths. Quality of care is also critical. We present findings in the ENAP/EPMM Measurement Improvement Roadmap regarding opportunities to better measure and use these data.

**Methods**

The United Nations and partners undertook technical reviews, evidence synthesis, and consultation regarding measures of coverage for ANC, SBA, and PNC, and linked effective coverage indicators. We considered measurement feasibility and interpretability. As part of the updated Measurement Improvement Roadmap, the findings inform what is measured now, gaps, and next steps.

**Results**

Where do the data come from?

- Based on the United Nations Children’s Fund data warehouse, the number of countries with available household survey data in 2016–2021 was 90 for ANC 4+, 162 for SBA, 81 for maternal PNC, and 75 for newborn PNC.

What can be measured now and what is the data quality?

- Population representative measures of ANC 4+, SBA, and PNC are collected through household surveys. Given new World Health Organization recommendations, ANC 4+ may be replaced by 8+.
- Country-level variations in the definition of SBA present challenges for standardized and comparable measurement.
- Measures of effective coverage for these services have been developed, but the assessment of more detailed quality of care is likely to be more accurate in routine health information systems than in surveys of maternal recall.

**Conclusions**

What next?

- Household survey programs require continued support, especially in lower-income countries and humanitarian settings.
- Strengthening routine health information systems is essential to ensure more data, more details on quality of care, and faster feedback for national and subnational monitoring. Implementation of global guidance on standardized definitions is key.
- Increased investment in digital technologies with better links between data sources will improve data availability and quality.
- Investment in data capacity and use is also crucial to drive progress toward achieving the Sustainable Development Goals.

## F24.5. Impact data for maternal and newborn health: what can be measured now and what next to best monitor progress toward achieving Ending Preventable Maternal Mortality/Every Newborn Action Plan (EPMM/ENAP) targets?

### Allisyn Moran^1^, Joy Lawn^2^, Harriet Ruysen^2^

#### ^1^World Health Organization; ^2^London School of Hygiene & Tropical Medicine

*BMC Proceedings 2024*, **18(5):**F24.5

Submission ID #: IMNHC873

Panel ID# (if applicable): DUBIC3274

**Background**

Impact data on maternal mortality ratio (MMR), neonatal mortality rate (NMR), and stillbirth rate (SBR) are crucial to track for Sustainable Development Goal progress. These mortality data and linked outcomes, such as low birthweight (LBW) and preterm birth, are typically collected through a variety of data platforms: nationally representative surveys, routine health information systems (RHISs), or civil registration and vital statistics (CRVS) systems.

**Methods**

The United Nations and partners have led evidence synthesis and consultations to review the status of EPMM/ENAP impact indicators to inform the updated Measurement Improvement Roadmap, highlighting the following:

- Where do the data come from?
- Which countries have what data now, and from which data platform?
- What can be measured now and what is the data quality?
- Comparability, e.g., which definitions, quality (data completeness, etc.)
- What next?
- Gaps in research and opportunities for innovation

**Results**

Where do the data come from?

- 157/195 countries for SBR (UN Inter-agency Group for Child Mortality Estimation 2023)
- 158/195 countries for LBW rate (in press)
- 103/195 countries for preterm birth rate (in press)

What can be measured now and what is the data quality?

- Impact indicator definitions are consistently applied for MMR, NMR, and LBW in almost all countries. SBR definition consistency has improved with the World Health Organization definition for international comparison (>28 weeks gestation). Preterm birth rate definitions are consistent but gestational age measurement methods and accuracy vary.
- Many high mortality countries rely on household surveys for data on impact measures. Now that more than 80% of births are in facilities, coverage of facility RHISs and CRVS could be rapidly increased.

**Conclusions**

What next?

- Improve RHIS data quantity and quality for facility births and deaths, linked to maternal and perinatal death surveillance/audits.
- Birth registration for every facility birth.
- Innovations to improve gestational age estimation and birthweight measurement with digital technologies for linking to individual data.
- Investments in widely available, programmatically relevant data for use at local, district, and national levels to drive quality of care for every woman and every newborn.

## F25.1. Building a conceptual and monitoring framework for emergency referral systems, with implementation perspectives from Mozambique and India

### Emily Keyes^1^, Prateek Gupta^2^, Megan Lydon^1^, Aguinaldo Mariano^1^, GV Ramana Rao^3^

#### ^1^FHI 360; ^2^London School of Hygiene & Tropical Medicine; ^3^108 Emergency Management Research Institute Green Health Services

*BMC Proceedings 2024*, **18(5):**F25.1

Submission ID #: IMNHC36

Panel ID# (if applicable): DUPVM2336

**Panel Description**

With timely access to skilled care, most maternal and newborn deaths in low-income countries are preventable. Reaching the right care at the right time remains a challenge, especially when emergency interfacility referral is required to manage complications.

Referral is a critical component of high-quality health systems that requires many elements within and outside of facilities to function properly. This panel brings together implementers and researchers to describe a consolidated conceptual framework and a proposed monitoring framework for emergency referral system performance, aimed to guide health planners and policymakers toward improved system functioning.

Panelists will summarize current evidence around emergency referral systems, including a multi-country analysis of referral system performance, and share perspectives of designing and managing referral systems in different contexts. The aim is to generate discussion of practical approaches to design, implement, and continuously monitor these complex systems to enable timely access to life-saving services in different contexts.

The conceptual framework was developed by members of the management committee for the global Community of Practice for Transport and Referral, based at London School of Hygiene and Tropical Medicine; the multi-country analysis was conducted by FHI 360 with funding from the Averting Maternal Death and Disability Program at Columbia University. Implementation perspectives come from a USAID-funded project in Mozambique and an emergency management institute in India.

## F25.2. Toward a monitoring framework for emergency obstetric and neonatal referral system readiness: a multi-country analysis

### Megan Lydon^1^, Emily Keyes^1^, Prateek Gupta^2^, Bunsoth Mao^3^, Sarah Mercer^4^, Peter Acker^5^, Loveday Penn-Kekana^2^

#### ^1^FHI 360; ^2^London School of Hygiene & Tropical Medicine; ^3^University of Health Sciences, Cambodia; ^4^Columbia University; ^5^Stanford University School of Medicine

*BMC Proceedings 2024*, **18(5):**F25.2

Submission ID #: IMNHC38

Panel ID# (if applicable): DUPVM2336

**Background**

There is a gap in referral system measurement and a need to identify the key elements of referral system inputs and processes that lead to a functioning system. Further, referral systems are complex systems difficult to summarize with any one indicator. Given this, we aimed to develop an initial composite indicator that captures multiple dimensions of referral systems through easily measured indicators that describe system readiness.

**Methods**

Based on the literature, WHO guidelines, and relevant experiences, we identified six key dimensions of referrals through consensus. We mapped existing data from national emergency obstetric and newborn care assessments to these dimensions. Using data from more than 6,000 health facilities across Ghana, Zambia, Cambodia, Ethiopia, and Malawi from 2010–2020, we performed descriptive analysis of indicators aligning with the referral dimensions. We then constructed a composite indicator: we scored and standardized variables, aggregated scores by dimension, weighted each dimension equally, and summed them for a total score presented as a percentage. We used descriptive analysis to compare referral system readiness across factors.

**Results**

The six dimensions comprise (1) transportation readiness, (2) referral efficiency and coordination of care, (3) care during transport, (4) financial accessibility of referral, (5) interfacility relational dynamics, and (6) family-centered care. Existing data aligned with dimensions 1-4; while important, data on dimensions 5-6 are rarely captured and represent a gap in current monitoring efforts. Composite indicator scores ranged from 41% to 54%. Financial accessibility of referral remained the strongest dimension across countries, with scores of 53% in Ghana to 97% in Zambia. Care during transport was consistently the weakest with scores of 10% in Malawi to 32% in Zambia. Across countries, the mean composite score was higher for hospitals than for other health facilities, with a 15% to 19% difference between groups. In most countries, health facilities ranking in the top quintile of referral readiness also represented the largest proportion of monthly deliveries.

**Conclusions**

These results demonstrate a method to succinctly measure referral system readiness. The proposed framework and composite indicator could be routinely used to monitor progress, identify gaps, and guide referral system strengthening efforts.

## F25.3. Title: interfacility obstetric and neonatal transfers: clarifying their fit in theory and in practice

### Prateek Gupta^1^, Megan Lydon^2^, Emily Keyes^2^, Peter Acker^3^, Loveday Penn-Kekana^1^

#### ^1^London School of Hygiene & Tropical Medicine; ^2^FHI 360; ^3^Stanford University School of Medicine

*BMC Proceedings 2024*, **18(5):**F25.3

Submission ID #: IMNHC37

Panel ID# (if applicable): DUPVM2336

**Background**

Referral is a widely used and broad term that covers a range of journeys that women and newborns must make to and between health facilities. Referrals are a complicated concept that rely on the interactions between the personnel and structures of health systems, transportation, clinical guidelines, health-seeking behaviors of patients and caregivers, public and private providers, and sociodemographic factors, among other dimensions. Monitoring an effective interfacility health-related transfer system requires a framework that can conceptualize interactions between dimensions that contribute to the outcome of interest and highlight specific areas of focus for improved performance.

For this work, we have focused on interfacility emergency obstetric and neonatal transfers. We build on existing conceptual frameworks such as the Three Delays model, Social Determinants of Health, and WHO guidance on transport during emergencies, among others, to address aspects of interfacility health-related transfers. Each framework has its limitations largely because of the service use and service organization assumptions that underlie them. These frameworks were designed for different theoretical settings and propose varying operational and measurement approaches.

**Methods**

We conducted a narrative review of frameworks that describe dimensions of interfacility health-related transfers. In addition, content analysis was undertaken to conceptualize how the dimensions of each framework can be synthesized to address considerations for measurement.

**Results**

To inform future efforts to improve interfacility transfers, we propose an integrated conceptual framework that we believe can assist health care managers and researchers in synthesizing evidence, understanding phenomena, and informing attempts to improve service, ensuring that pregnant women and newborns get to the right place at the right time and receive the right care. In addition, we propose using specific terminology to describe aspects of interfacility health-related transfers to assist in harmonizing concepts across study settings, health conditions, and severity.

**Conclusions**

This proposed conceptual framework and common language for interfacility health-related transfers provide an overarching model and language that may help guide the development, validation, implementation, and interpretation of measures and suggests boundaries for the health system’s responsibilities.

## F26.1. Small vulnerable newborns: measurement now and next to enable better care and faster progress to national targets around the world

### Elizabeth A. Hazel^1^, Yemi Okwaraji^2^, Lorena Suarez-Idueta^3^, Mihretab Salasibew^4^, Aris Papageorghiou^5^, Hannah Blencowe^2^

#### ^1^Johns Hopkins Bloomberg School of Public Health; ^2^London School of Hygiene & Tropical Medicine; ^3^Mexican Society of Public Health; ^4^Children’s Investment Fund Foundation; ^5^University of Oxford

*BMC Proceedings 2024*, **18(5):**F26.1

Submission ID #: IMNHC843

Panel ID# (if applicable): DUWEM47843

**Panel Description**

Globally, one in six babies is born with a low birthweight and one in 10 is born preterm. The Global Nutrition Target for 30% reduction in low birthweight by 2025 is off track, and preterm rates are rising in many countries.

Newborns with low birthweight include those born preterm, or small for gestational age, and those that have both conditions. Such infants are at risk of early mortality and poor health throughout their life course. However, birthweight and gestational age are often measured separately, meaning the two types of low birthweight cannot be distinguished; small for gestational age infants may not be recognized because of lack of knowledge of gestational age; and stillbirths are omitted. New approaches are needed.

The Vulnerable Newborn Measurement Collaboration includes > 250 members including governments, implementers, and researchers. The collaborative has enabled a massive dataset of 279.3 million live births from 23 high-income and 14 low- and middle-income countries, 2000 to 2021. This dataset allows newborn types to be determined by combining gestational age (preterm vs. term) and size (small-, appropriate-, and large-for-gestational age), enabling more targeted care and elucidating aetiology to accelerate health of vulnerable newborns globally.

We share novel findings, and then a panel of country representatives and experts will map out the way forward. This panel links to the Lancet Small Vulnerable Newborn series, “Born Too Soon Decade” report and Vulnerable Newborn supplement in BJOG: An International Journal of Obstetrics and Gynaecology, which will be launched at IMNHC. We aim to inform actions before IMNHC 2025.

## F26.2. Policy talk show/panel: measurement now and next to enable better care and faster progress to national targets

### Mihretab Salasibew^1^, Elizabeth A. Hazel^2^, Hannah Blencowe^3^, Robert Black^2^, Joy Lawn^3^, Msandeni Chiume^4^, Aris Papageorghiou^5^, Lucia Hug^6^

#### ^1^Children’s Investment Fund Foundation; ^2^Johns Hopkins Bloomberg School of Public Health; ^3^London School of Hygiene & Tropical Medicine; ^4^Kamuzu University of Health Sciences; ^5^University of Oxford; ^6^United Nations Children’s Fund

*BMC Proceedings 2024*, **18(5):**F26.2

Submission ID #: IMNHC854

Panel ID# (if applicable): DUWEM47843

**Background**

This dynamic TV talk show session will include diverse speakers and involve engaging conversations, enabling inputs from the audience, discussion points from the data presented, plus interactive voting. The focus will be a range of viewpoints on what next for metrics and action, and aiming to be as actionable as possible for change in the next two years before IMNHC reconvenes.

**Methods**

The MC with be Mihretab Salasibew in partnership with Joy Lawn to enable Mentimeter voting. Participants will cover the following topics:

- National routine data systems: National government leaders for routine data from Nepal and Malawi, based on the most senior relevant person present at the conference. These national experts will discuss implication and challenges for changing their national systems to improve data for babies born too soon and/or too small.
- Stillbirth inclusion: UN lead for improving and using stillbirth data, Dr. Danzhen You.
- Interventions for nutrition and what’s next: Professor Bob Black as a world-leading expert on nutrition data for MNH and evidence for interventions.

**Results**

Interactive audience voting using Mentimeter to vote on top actions in the next two years before IMNHC2025.

## F26.3. Small babies, high mortality risk: analyses of neonatal mortality for 125.7 million live births from 24 countries (2000–2020)

### Elizabeth A. Hazel^1^, Hannah Blencowe^2^

#### ^1^Johns Hopkins Bloomberg School of Public Health; ^2^London School of Hygiene & Tropical Medicine

*BMC Proceedings 2024*, **18(5):**F26.3

Submission ID #: IMNHC849

Panel ID# (if applicable): DUWEM47843

**Background**

Babies born too soon (preterm) and/or too small (low birthweight) are more likely to die, but little is known about the mortality risks for other combinations of preterm and size. The Vulnerable Newborn Measurement Collaboration of >250 members analyzed data from 24 countries to estimate the neonatal mortality risks for six newborn types.

**Methods**

We included 125.5 million live births in high-middle-income settings and 225,441 live births in low-and middle-income settings with exposure data and linked neonatal survival, 2000 and 2020. We assigned six types combining gestational age (preterm [PT] or term [T]) and size for gestational age (Small-, Large-, or Appropriate-for gestational age [SGA, LGA, AGA]). Medians and interquartile ranges (IQR) were calculated for prevalence, mortality rate, and relative risk ratio (RR) for four small types (PT+SGA, PT+AGA, PT+LGA, T+SGA), one large (T+LGA), and one reference (T+AGA).

**Results**

As expected, the most common type was T+AGA (median: 68.8% in high- and 54% in lower-income settings). In higher income settings, T+AGA had the lowest mortality rate (median: 0.6 deaths per 1,000 livebirths), and in lower-income settings it was T+LGA (7.2 deaths) followed by T+AGA (11.6 deaths). Preterm newborn faced the highest risks. In high-income settings, the median mortality RR was PT+SGA (68.2, IQR: 56.1-78.0), PT+AGA (34.3, IQR: 23.9-37.5), PT+LGA (28.3, IQR: 22.6-32.3), followed by T+SGA (5.4, IQR: 4.2-6.3). In lower-income settings, preterm babies also had higher mortality risk: PT+SGA (10.0, IQR: 5.5-10.4), PT+AGA (4.5, IQR: 4.3-6.3), PT+LGA (4.9, IQR: 2.5-7.7), followed by T+SGA (2.5, IQR: 2.0-2.7). No additional risk was found for T+LGA in high- (0.8, IQR: 0.8-0.8) or low-income settings (0.9, IQR: 0.8-1.0).

**Conclusions**

Four small vulnerable newborn types all have increased mortality risk in all settings. Preterm types have a much higher risk and account for the largest population attributable risk. PT+SGA babies are rarer, but face the greatest risks. Lower-income settings have a higher mortality risk for all babies, which reduces the RR, but is associated with more deaths in absolute numbers.

## F26.4. Vulnerable newborn types: prevalence of vulnerable newborns and analyses of birth outcome data from 43 countries in six regions (2000–2021)

### Lorena Suarez-Idueta^1^, Elizabeth A. Hazel^2^, Hannah Blencowe^3^, Daniel Erchick^2^, Ellen Bradley^3^

#### ^1^Mexican Society of Public Health; ^2^Johns Hopkins Bloomberg School of Public Health; ^3^London School of Hygiene & Tropical Medicine

*BMC Proceedings 2024*, **18(5):**F26.4

Submission ID #: IMNHC845

Panel ID# (if applicable): DUWEM47843

**Background**

Preterm and small newborns are at higher risk for mortality and long-term adverse health outcomes, yet there are major gaps in national data. Absence of a unified classification system to estimate prevalence of detailed newborn outcomes presents challenges for targeting preventive and therapeutic interventions to the most vulnerable. We analyzed data based on a new classification and present results on prevalence of six newborn types from 43 countries, 2000 to 2021.

**Methods**

This secondary analysis used datasets from 23 countries with strong national data systems (primarily high- and middle-income countries ) and 45 subnational (23 countries), population-based studies with high-quality birth outcome data (primarily low- and middle-income countries ). We defined a set of six newborn types using gestational age (preterm [PT], term [T]) and weight-for-gestational age (small-for-gestational age [SGA], appropriate-for-gestational age [AGA], large-for-gestational age [LGA]) using the Intergrowth 21st newborn standard. We defined small types as any type with PT or SGA. Separately for national and subnational sources, we calculated the prevalence of newborn types by country or study and presented information on data quality, study, and participant characteristics.

**Results**

The median prevalence of six types were T+AGA: 66.9%, T+LGA: 5.5%, T+SGA: 15.1%, PT+LGA: 1.1 %, PT+AGA: 6.5%, and PT+SGA: 0.8%. Median prevalence of small types was 25.9%. Median prevalence of small types by region were: Southern Asia (52.4%); Sub-Saharan Africa (34.9%); and Eastern Asia, Southeastern Asia, and Oceania (21.9%) (excluding Australia and New Zealand); Middle East (15.4%); Latin America and the Caribbean (13.8%); and Northern America, Australia and New Zealand, Central Asia, and Europe (10.5%).

**Conclusions**

Our study is the first to provide multi-country analysis of newborn types, using more than 170 million livebirths from 43 countries across six SDG regions. Identification of specific newborn types was feasible and revealed high prevalence of small newborn types with variations across region, setting, and time. More surprisingly, we found a high rate of large for gestational age in several regions. This novel framework has potential to better inform policies and programs for the most vulnerable and sick newborns.

## F26.5. Measurement past, present, and future for small vulnerable newborns, born too soon and too small

### Eric Ohuma^1^, Elizabeth A. Hazel^2^, Hannah Blencowe^1^, Yemi Okwaraji^1^, Judith Yargawa^1^,

#### ^1^London School of Hygiene & Tropical Medicine; ^2^Johns Hopkins Bloomberg School of Public Health

*BMC Proceedings 2024*, **18(5)**:F26.5

Submission ID #: IMNHC844

Panel ID# (if applicable): DUWEM47843

**Background**

Small vulnerable newborns (SVN), including those born low birthweight (LBW, < 2500g), preterm and/or small-for-gestational age (SGA) account for more than 80% of newborn deaths globally and lifelong loss of human capital. Most of this burden could be averted, but global nutrition and survival targets are off track. Transformation of data availability, quality, and use is crucial to inform investments in prevention and care. As part of UN estimates, Lancet SVN series, and Born Too Soon report, we reviewed national-level outcome data for all UN member states during 20 years to inform data transformation.

**Methods**

National routine administrative data on LBW, preterm birth, and SGA for all 194 WHO member states and the occupied Palestinian territory, including east Jerusalem, were systematically collated in a global database, 2000 to 2020. Data availability and quality were systematically assessed.

**Results**

Most UN member states (124/195, 64%) had data on LBW, compared with 82/195 (40%) for preterm birth and just 8 countries for SGA. Routine data system reporting was highest in North America, Australia, and Europe, where in 2019 ~95% of livebirths had LBW data and > 75% had preterm birth data. In contrast, just 13% of livebirths in Sub-Saharan Africa had national information on LBW and only 8% on preterm birth. In South Asia, 16% of livebirths had national information on LBW and only 5% on preterm birth. Most low- and middle-income country data were collected through hospital-based systems with aggregate data. Data quality was generally high in the North American, Australasian, and European region, although only ~25% of countries reported metadata for gestational age assessment method, commonly reported as Last Menstrual Period.

**Conclusions**

Every baby, including stillbirths, should be counted, weighed, and assessed. Countries in Sub-Saharan Africa and South Asia have data gaps, but also opportunities for rapid progress given the majority of births in facilities, and expansion of electronic health information systems that already include LBW data. Gestational age can be incorporated, linked to improved assessment, which is increasingly feasible. Adding individually linked data will enable countries to track individual quality of care and long-term outcomes, crucial for every family and to track SDG and Every Newborn progress.

## F27.1. Fear: the hidden threat for quality of care in childbirth

### Ana Pilar Betran^1^, Sarah Elaraby^2^, Fadima Inna Kamina Yaya Bocoum^3^, Ameporn Ratinthorn^4^, Sunny Mannava^5^

#### ^1^World Health Organization; ^2^University of Alexandria; ^3^Institut de Recherche en Sciences de la Santé; ^4^Mahidol University, Bangkok; ^5^University of Hyderabad

*BMC Proceedings 2024*, **18(5):**F27.1

Submission ID #: IMNHC332

Panel ID# (if applicable): DWOLL16332

**Panel Description**

Despite great improvements in health care in the past decades, fear is a prevailing emotion in childbirth. It increasingly influences women’s decisions and health care providers’ clinical practices, which can result in suboptimal quality of care.

Women and families fear pain, lack of support, disrespect/abuse during labor and childbirth, and adverse outcomes associated with vaginal delivery. Providers are afraid of the uncontrollable nature of childbirth, pressure from families, financial/judicial litigation, loss of practicing rights, reputational damage, and physical/verbal violence from families in case of adverse outcomes in vaginal birth.

Fears motivated by the belief that cesarean section is a safer option that guarantees the best outcome is one of the factors driving the unprecedented increase of cesarean section use witnessed by contemporary societies worldwide. Although a cesarean is life-saving when needed, it is associated with risks that are higher in women with limited access to obstetric care.

We argue that a constructive dialogue needs to emerge between stakeholders to (i) improve understanding of each other’s views, values and needs; (ii) to challenge misconceptions considering the evidence; and (iii) to reconcile societies’ lower tolerance for negative obstetric outcomes and the uncertain nature of childbirth where ideal outcomes cannot be promised, but high-quality, evidence-based care should be ensured.

This panel brings together the findings of two projects aiming to improve understanding of fear as a driver for cesarean section decision-making (Re-JUDGE) and to adapt and implement non-clinical interventions to optimize the use of cesarean section (QUALI-DEC).

## F27.2. Health system and environmental influences on fear of maternity care litigation in India (Re-Judge Project)

### Sunny Mannava^1^, B.R. Shamanna^1^, Indie Kaur^2^, Gill Moncrieff^3^, Joanna Erdman^4^, Elena Altieri^5^, Sarah Elaraby^6^, Maria Regina Torloni^7^, Ana Pilar Betran^5^, Soo Downe^3^

#### ^1^University of Hyderabad; ^2^Fernandez Foundation; ^3^University of Central Lancashire; ^4^Dalhousie University; ^5^World Health Organization; ^6^University of Alexandria; ^7^São Paulo Federal University

*BMC Proceedings 2024*, **18(5):**F27.2

Submission ID #: IMNHC400

Panel ID# (if applicable): DWOLL16332

**Background**

In the past few decades, India has witnessed a sharp increase in cesarean section (CS) rates (3% in 1992–1993 to 23% in 2019–2021). Although more surgical births were needed to reduce maternal and neonatal mortality and complications, current rates in some India states and hospitals are reaching more than 60%. Overuse of CS without a medical need can put both mother and baby at risk in the short and long term. Health provider fear of litigation has been associated with unnecessary CS worldwide.

**Methods**

The Re-Judge project aimed to use insights from behavioral science to develop a multimedia program for lawyers and judges that could address their knowledge and beliefs about the risks and benefits of CS, in the context of legal and human rights. In the process, we explored the extent to which medical fear of litigation might be a driver for CS in the India. We conducted in-depth interviews with lawyers, obstetricians, and women’s representatives, using a semi-structured interview guide. Framework analysis techniques were used to map interview data to behavioral drivers and mental models developed in the review phase of the project. Two workshops were then held to explore attitudes and intentions of medical and legal participants toward CS because of fear of litigation.

**Results**

Nineteen participants were interviewed. Twenty-four attended the workshops. Mean years of experience was 26 years; median age 52 years. Fear of litigation was a significant concern for the participating doctors. This was a surprise for some of the participating lawyers. However, medical fearfulness extended beyond litigation, including fear of physical attack from disgruntled families, and fear of being shamed and of suffering reputational loss on social media.

**Conclusions**

Although fear of litigation is an important driver of overuse of CS, fear of violence and of social media exposure are also relevant, if an adverse event occurs. Safe discussion spaces between doctors, lawyers, and service user representatives, such as the Re-Judge workshops, may enable different parties to understand and address misconceptions that underpin overuse of medical procedures carried out because of fear, rather than medical necessity or women’s choice.

## F27.3. Fears driving women’s preference and decision-making on mode of birth: experience from Burkina Faso

### Fadima Inna Kamina Yaya Bocoum^1^, Charles Paulin Kaboré^1^, Simon Tiendrebeogo^1^, Saran Barro^1^, Alexandre Dumont^2^, Claudia Hanson^3^, Ana Pilar Betran^4^, Meghan Bohren^5^

#### ^1^Institut de Recherche en Sciences de la Santé; ^2^Institute of Research for Development; ^3^Karolinska Institutet; ^4^World Health Organzation; ^5^University of Melbourne

*BMC Proceedings 2024*, **18(5):**F27.3

Submission ID #: IMNHC398

Panel ID# (if applicable): DWOLL16332

**Background**

In Burkina Faso, cesarean section (CS) rates have increased markedly since 2006 when the government deployed a national subsidy policy for childbirth and emergency obstetric care. There is concern that the policy may have also increased unnecessary CS. Suboptimal communication with health care providers, and women’s lack of information and involvement in decision-making during childbirth, are recognized problems in Burkina Faso. The increase in CS, coupled with the lack of information and communication, may place pregnant women in a vulnerable position. We aimed to explore women’s fears, and factors driving these fears, in Burkina Faso.

**Methods**

This formative qualitative research was conducted in 2020 to inform the QUALI-DEC project, to design, adapt, and implement a strategy to optimize use of CS. We conducted in-depth interviews with pregnant and postpartum women in four hospitals in Burkina Faso and used a thematic analysis approach to summarize findings on fear.

**Results**

A total of 38 in-depth interviews (22 with pregnant and 16 with postpartum women) were conducted. Most participants reported that they experienced fear and anxiety during pregnancy. Although women feared losing their life and having complications during childbirth that could influence outcomes, most did not talk about their fears. It was challenging to discuss fears with a birth companion, because most women did not choose this companion themselves, as they were typically a member of her family or in-laws who were available at the time of birth. Although most women did not discuss fears with providers, spouses, or parents, the few women who did talk about it benefited from the emotional support of providers and spouses, which helped to control their anxieties. Women preferred a vaginal birth and were afraid of needing a CS. This preference was based on fear of failing to be recognized as a “strong woman” by their in-laws.

**Conclusions**

Preferred mode of birth is mainly driven by the fear of undeserved social recognition as a “strong woman”. In particular, recognition by the family in-law is a cause of anxiety among pregnant women. The emotional and social impact of a CS on women should not be ignored.

## F27.4. Behavioral factors associated with fear of litigation as a driver for the increased use of cesarean sections: a scoping review

### Sarah Elaraby^1^, Elena Altieri^2^, Soo Downe^3^, Joanna Erdman^4^, Sunny Mannava^5^, Gill Moncrieff^3^, B.R. Shamanna^5^, Maria Regina Torloni^6^, Ana Pilar Betran^2^, Meghan Bohren^7^

#### ^1^University of Alexandria; ^2^World Health Organization; ^3^University of Central Lancashire; ^4^Dalhousie University; ^5^University of Hyderabad; ^6^São Paulo Federal University; ^7^University of Melbourne

*BMC Proceedings 2024*, **18(5):**F27.4

Submission ID #: IMNHC356

Panel ID# (if applicable): DWOLL16332

Please note the full manuscript published in April 2023 in *BMJ Open*:

(https://bmjopen.bmj.com/content/13/4/e070454)

## F28.1. Can we address the complexity of childbirth and newborn care with multi-component and complex interventions? Experience from the ALERT, Quali-Dec, and SCSL projects from Sub-Saharan Africa, Latin America, and Asia

### Claudia Hanson^1^, Lenka Benova^2^, Jean-Paul Dossou^1^

#### ^1^Karolinska Institutet; ^2^Institute of Tropical Medicine

*BMC Proceedings 2024*, **18(5):**F28.1

Submission ID #: IMNHC65

Panel ID# (if applicable): DXPLD1065

**Panel Description**

The complexity of childbirth care is characterized by three interfaces: First, the two continua of care from pre-pregnancy to postpartum care and community to referral hospital, meaning that the key health systems characteristics and the functioning of the linkages between services points including the referral systems have a major impact. Second, childbirth care includes preventive and promotive, thus routine care as well as time-sensitive emergency care. Third, quality childbirth care is provided by a team of nurses, midwives, obstetricians, as well as neonatologists and pediatric staff, thus a multi-professional team with different pre-service training and professional values and identities, creating frictions. Over- and underprovision of evidence-based interventions are seen in all settings, with the overuse of cesarean sections being a well-known example. Abuse and neglect have been raised and responsiveness during birth is high on the international agenda. Improving quality of care within these complex systems demand careful combination of strategies to address the complexity and the different drivers of this complex system. But complex interventions may also overburden implementers and systems. Complex interventions demand sufficient resources, long-term investments and sufficient patience to mentor and support implementing teams and institutions. Here we summarize our learning from three complex interventions. We apply a scalability and adaptability lens to highlight critical issues.

## F28.2. Effect of collaborative quality improvement on stillbirths, neonatal mortality, and newborn care practices in hospitals of Telangana and Andhra Pradesh, India: evidence from a quasi-experimental mixed-methods study

### Karen Zamboni^1^, Lenka Benova^2^, Claudia Hanson^1^, Zelee Hill^3^, Samiksha Singh^4^, Joanna Schellenberg^5^

#### ^1^Karolinska Institutet; ^2^Institute of Tropical Medicine; ^3^University College London; ^4^Institute of Public Health, India; ^5^London School of Hygiene & Tropical Medicine

*BMC Proceedings 2024*, **18(5):**F28.2

Submission ID #: IMNHC1376

Panel ID# (if applicable): DXPLD1065

Please note the full manuscript published in January 2021 in *BMC*:

(https://implementationscience.biomedcentral.com/articles/10.1186/s13012-020-01058-z#:~:text=We%20found%20no%20evidence%20that,high%20attrition%20from%20participating%20hospitals.)

## F28.3. Quali-Dec: complex intervention to improve decision-making for cesarean section

### Charles Paulin Kaboré^1^, Claudia Hanson^2^, Lenka Benova^3^, Alexandre Dumont^4^, Mac Quo Nhu Hung^5^, Pisake Lumbiganon^6^, Guillermo Carroli^7^, Ana Pilar Betran^8^

#### ^1^Institut de Recherche en Sciences de la Santé; ^2^Karolinska Institutet; ^3^Institute of Tropical Medicine; ^4^IRD-Université de Paris; ^5^Pham Ngoc Thach University; ^6^Khon Kaen University; ^7^Centro Rosarino de Estudios Périnatales Asociación; ^8^World Health Organization

*BMC Proceedings 2024*, **18(5):**F28.3

Submission ID #: IMNHC1211

Panel ID# (if applicable): DXPLD1065

**Background**

The overuse of cesarean section diverts scarce resources and thereby reduces access to health care for women in need. Cesarean section decision-making is a complex process involving multiple actors at different levels. Changing behaviors to reduce unnecessary cesarean sections presents many challenges for fragile and developing health care systems. The appropriate use of cesarean section through QUALIty DECision-making by women and providers (QUALI-DEC) study aims to implement a four-component intervention of i) audits of Cesareansections , ii) opinionleaders and a decision analysis tool, plus emotional support through companionship for women in 32 hospitals in Argentina, Thailand, Vietnam, and Burkina Faso. Here we present implementation challenges and successes encountered when implementing this multi-faceted intervention.

**Methods**

The implementation strategy allows adaptations of the intervention to fit the context. Formative research in the baseline period assessed the main drivers and barriers to intervention implementation. Communication materials were developed to inform and educate the various stakeholders about the four components. The country-level coordinator conducts quarterly visits to each participating hospital to support the providers and collect data of implementation challenges.

**Results**

The formative research has shown that few local clinical protocols used for labor and delivery management are evidence-based. Most providers were comfortable with the idea of a local opinion leader to promote good clinical practice. After a five-day training, opinion leaders introduced WHO algorithms to participating hospitals and audit methods to evaluate clinical practices. The Robson classification is well accepted by providers, but its interpretation remains difficult without continuous support. The decision analysis tool to support an informed choice of the mode of delivery is welcomed by women and providers in both versions: a paper-based and web-based application “Providers are fairly supportive to help women choose a companion during labor and delivery, but lack of space and privacy is a main barrier in many contexts.

**Conclusions**

The combined approach targeting women and providers is necessary to introduce the concept of shared decision-making for cesarean section in health care facilities. Such an approach is a challenge to physician-centered models of care and in settings where women are less empowered and demands of support are high.

## F28.4. The Action Leveraging Evidence to Reduce Perinatal Mortality and Morbidity (ALERT) study targeting high-caseload hospitals of Benin, Malawi, Tanzania, and Uganda: early implementation experience

### Jean-Paul Dossou^1^, Lenka Benova^2^, Peter Waiswa^3^, Kristi Annerstedt^1^, Helle Moelsted-Alvesson^1^, Andrea Pembe^4^, Kidanto Hussein^5^, Mechthild Gross^6^, Effie Chipeta^7^, Claudia Hanson^1^

#### ^1^Karolinska Institutet; ^2^Institute of Tropical Medicine; ^3^Makerere University; ^4^Muhimbili University of Health and Allied Sciences; ^5^The Aga Khan University; ^6^Hannover Medical School; ^7^Kamuzu University of Health Science

*BMC Proceedings 2024*, **18(5):**F28.4

Submission ID #: IMNHC805

Panel ID# (if applicable): DXPLD1065

**Background**

Reductions in perinatal as well as maternal mortality are too slow to reach the ambitious Sustainable Development Goals targets. The complexity of underlying conditions to reduce perinatal mortality as well as the multi-disciplinary team involved in care around the time of birth and postpartum present major challenges.

The Action Leveraging Evidence to Reduce perinatal morTality and morbidity (ALERT) study aims to implement a four-component intervention of co-design, training, quality improvement, and mentoring in 16 hospitals in Benin, Malawi, Tanzania, and Uganda. Here we present implementation challenges and successes encountered when implementing this multi-faceted intervention.

**Methods**

We implemented a comprehensive co-design strategy. We conducted interviews with laboring women, companions, and midwifery providers and observations in half and a context and facility assessment in all 16 hospitals. Our co-design structure allows adaptation of the implementation strategy and provides data of implementation challenges.

**Results**

Assessments of midwifery providers indicated important knowledge and skills deficits of routine labor monitoring, such as the frequency of fetal monitoring and the application of the APGAR score. Laboring women indicated unresponsive contexts, including long waiting time, negligence, and abusive provider. This was more often seen when labor was longer and complicated. Midwifery providers confirmed in reflexive sessions the unresponsiveness and explained being overburdened and their feeling of helplessness. Our competency-based training targeting the needs developed during the co-design phase supporting reflective exercises and to implement changes, such as sign boards and on-site payment to speed up formal admission. Our quality improvement component aiming to implement operational and structural innovations was difficult to implement in several facilities, probably as midwifery providers felt too disempowered and needed time to understand the structured nature of quality improvement. In contrast, mentoring was substantially easier to integrate. Physical mentoring was preferred over WhatsApp, which has cost and time implications.

**Conclusions**

Continued efforts appreciating the complexity of the context are needed to motivate and help midwifery staff to innovate changes for improved quality of care. Such a comprehensive approach is challenging to implement in view of monetary limitations, but also limited time and leadership skills.

## F29.1. Strengthening the Quality of Practice of Maternal and Perinatal Death Surveillance and Response (MPDSR)

### Francesca Palestra^1^, Erna Mulati^2^, Eszter Kismodi^3^, Brenda Kharono^4^, Florina Serbanescu^1^, Mularsih Restianingrum MKM^2^

#### ^1^Centers for Disease Control and Prevention; ^2^Ministry of Health, Indonesia; ^3^Sexual and Reproductive Health Matters; ^4^FHI 360

*BMC Proceedings 2024*, **18(5)**:F29.1

Submission ID #: IMNHC460

Panel ID# (if applicable): DXYCD60460

**Panel Description**

The World Health Organization (WHO) and partners, part of the MPDSR technical working group, are committed together to strengthen implementation of maternal and perinatal death surveillance and response (MPDSR) to enhance the quality of maternal and perinatal care and to improve health outcomes.

A key intervention for improving maternal, perinatal, and neonatal survival is understanding the number and causes of deaths. This panel will show how creating a community of practice of MPDSR data use with specific implementation experiences from Uganda, Indonesia, and other countries is possible in order to actively track number of deaths and respond to these deaths. Moreover, we will present to the audience an important new tool: the manual on “Legislating maternal death surveillance and response.”It is based on a desk-review study of a cross-section of countries that have instituted a legal or regulatory framework for implementing MPDSR systems or are aspiring to develop such a framework. Countries increasingly find legislation as a useful accountability tool that has the capacity to supplement policy through establishing a legal or regulatory mandate for reporting and reviewing maternal deaths. This enhances transparency and promotes MPDSR as a system where confidentiality needs to be always observed and respected.

The panel presented two experiences from Indonesia and Uganda where MPDSR was effectively implemented. Indonesia highlighted the evidence on the increased budget allocation for MNH services due to the MPDSR implementation: In 2021 were allocated 37,738 USD to MNH services, however thanks to the recommendations coming from the MPDSR implementation the budget has been increased to 233,813 USD in 2023. Another example is Uganda where a strong community of practice on MPDSR has been created at national level: based on the recommendations from the MPDSR action plans they have invested in community dialogues for demand creation of MCH services, transport Vouchers for teenage mothers to attend ANC, Clinical mentorships and training, prioritizing nurses and midwives, Collaborative learning sessions and webinars; adoption of QI strategies to improve complication case management, recruitment of critical cadres such as anesthetic officers; Redistribution of supplies and commodities; Increase procurement of emergency supplies at all EmONC healthcare facilities. Advocacy efforts, data on causes of death, underlying contributing factors as well as the recommendations identified in the response plans to improve the health system were pivotal factors in driving resource mobilization.

Our session will actively seek contributions from the audience on what further the World Health Organization with partners and policymakers can do to ensure further improvement and implementation of MPDSR cycle at country and global level.

## F29.2. Monitoring maternal death surveillance efforts: proposed notification and review coverage rate

### Florina Serbanescu^1^, Francesca Palestra^2^, Jean Pierre Monet^3^, Lillian Whiting Collins^4^, Allisyn Moran^2^, Jason Hsia^1^, Michel Brun^3^

#### ^1^Centers for Disease Control and Prevention; ^2^World Health Organization; ^3^United Nations Population Fund

*BMC Proceedings 2024,* **18(5):**F29.2

Submission ID #: IMNHC467

Panel ID# (if applicable): DXYCD60460

Please note the full manuscript published in February 2023 in *BMJ Open*:

(https://bmjopen.bmj.com/content/13/2/e066990)

## F29.3. Creating a community of practice of Maternal and Perinatal Death Surveillance and Response (MPDSR) data use: implementation experiences from Uganda

### Brenda Kharono^1^, Francesca Palestra^2^, Bruno Ssemwanga^3^, Patrick Walugembe^1^,Nicholas Matsiko^1^, Philip Wanduru^4^, Nathan Tumwesigye^1^, Peter Waiswa^5^, Richard Mugahi^3^, Marya Plotkin^1^

#### ^1^FHI 360; ^2^World Health Organization; ^3^Ministry of Health, Uganda; ^4^Makerere University College of Health Sciences; ^5^Makerere University

*BMC Proceedings 2024*, **18(5)**:F29.3

Submission ID #: IMNHC465

Panel ID# (if applicable): DXYCD60460

**Background**

In Uganda, the maternal mortality ratio remains unacceptably high at 336 per 100,000 live births, even though > 75% of births occur in health facilities. The institutional maternal mortality ratio is 92 per 100,000, and deaths among pregnant women occur with shocking regularity in health facilities from preventable causes. Utilization of the MPDSR cycle for quality improvement was prioritized by the Government of Uganda in 2018. As part of this effort, a community of practice (CoP) was convened to ensure discussion of data and follow-up of MPDSR recommendations.

**Methods**

The Ministry of Health Reproductive and Child Health (MOH/RCH), in collaboration with the USAID-funded Uganda Maternal, Child Health, and Nutrition Activity and various MNCH implementing partners started new platforms to increase accessibility/data visualization of MPDSR data and promote the “R” in the MPDSR cycle. In 2021, a forum for data-driven discussions, which provides professional development (CPD) credits for health care providers, was launched.

**Results**

A CoP was created comprising > 200 individuals representing > 50 organizations, including faith-based, public, and private sectors. Weekly MPDSR analyses go out to > 700 individuals; > 90 reports with filterable dashboards have been circulated. Weekly meetings led by MOH/RCH regularly attract > 150 participants. Selected results include: reduced unaudited deaths; improved submission of reports through DHIS2; procurement of refrigerators for blood storage; and inclusion of anesthesiologists in MPDSR committees.

**Conclusions**

The MPDSR CoP in Uganda is thriving and has resulted in trackable improvements, including > 90% on time, completed maternal death audits, quick provision of emergency supplies, and data driven regional mentorships. Inputs necessary to make the MPDSR CoP successful included: MOH-led platform for convening and mobilizing stakeholders; functional, routinely updated reporting system; and timely weekly submission of data, which informs development of interactive dashboards, weekly MPDSR summaries, maps, and detailed reports.

## F29.4. Legislating Maternal Death Surveillance and Response (MDSR)

### Eszter Kismodi^1^, Charles Ngwena^2^, Marcus Stahlhofer^3^, Francesca Palestra^3^

#### ^1^Sexual and Reproductive Health Matters; ^2^University of Pretoria; ^3^World Health Organization

*BMC Proceedings 2024*, **18(5)**:F29.4

Submission ID #: IMNHC464

Panel ID# (if applicable): DXYCD60460

**Background**

Recent global and national efforts have yielded significant progress. Reducing maternal mortality, however, remains a challenge. Establishing accountable MDSR systems that are mandated by law and are consistent with constitutional and human rights obligations and public health commitments of the state, have been highlighted as an important step forward in tackling the persistence of maternal mortality.

**Methods**

WHO has commissioned the development of a manual on “Legislating maternal death surveillance and response,” based on a desk-based study of a cross-section of countries that have instituted a legal or regulatory framework for implementing MDSR systems or are aspiring to develop such a framework. The choice of countries was based on: geographical diversity; history of using legislation or regulation to implement MDSR; history of maternal death-related litigation; development of maternal death-related jurisprudence; domestic proposals to introduce legislation for MDSR systems; and evidence of challenges of using law to implement MDSR. The study is based on what can be found in existing literature and does not engage in an empirical inquiry to confirm the accuracy of what is found in existing literature, for example, by engaging with informants and stakeholders in the respective countries.

**Results**

Countries increasingly find legislation as a useful accountability tool that has the capacity to supplement policy through establishing a legal or regulatory mandate for reporting and reviewing maternal deaths. In this context it has been considered that, apart from promoting accountability and transparency through revealing the magnitude of maternal deaths that may be underreported in death certificates, the normative force of law creates a clear chain of enforceable institutional responsibilities for reporting and enhancing the “surveillance” and “response” components of MDSR.

**Conclusions**

Legislation can facilitate the creation of an institutional framework that has requisite legal powers and duties to conduct surveillance, collate information and intervene timely in respect to modifiable factors at facilities and in the community. The law can be also essential for guaranteeing legal protections to individuals who take part as reviewers or respondents to ensure confidentiality of data and the review process, while ensuring access to remedies that is essential for legal accountability.

## F29.5. Subnational government engagement to strengthen maternal and perinatal death surveillance and response implementation to improve maternal and newborn care in East Sumba District, Indonesia

### Mularsih Restianingrum MKM^1^, Francesca Palestra^2^, Erna Mulati^1^, Esty Febriani^3^, Ratih Indriyani Rakhmawati^3^, Siti Nurul Qomariyah^3^, Tenggudai Littik^3^, Hana K Wadoe^3^, Irwan Saptono^3^, Kusum Thapa^3^, Ni Made Diah Permata Laksmi D^1^, Nida Rohmawati^1^

#### ^1^Ministry of Health, Indonesia; ^2^World Health Organization; ^3^Jhpiego

*BMC Proceedings 2024*, **18(5):**F29.5

Submission ID #: IMNHC462

Panel ID# (if applicable): DXYCD60460

**Background**

Maternal and Perinatal Death Surveillance and Response (MPDSR) is a form of continuous surveillance that links the health information system and quality improvement processes, which include identification, notification, quantification and determination of causes, and avoidability of maternal and perinatal deaths, and use of this information to respond with actions that will prevent future deaths. MOMENTUM Country and Global Leadership with provincial authorities strengthened the capacity of East Sumba MPDSR District committee, Indonesia by building capacity on maternal and perinatal deaths review and formulation of actionable recommendations to prevent future deaths. The recommendations were then shared to both health and non-health sectors for follow-up actions.

**Methods**

Using WHO guidelines, a series of online and offline training, were conducted in preparation for the MPDSR process with specific focus on formulation of Specific, Measurable, Achievable, Relevant, and Timebound (SMART) recommendations. MOMENTUM Country and Global Leadership facilitated use of Maternal and Perinatal Death Notification electronic system by the district team to conduct the process and assisted the death review workshop where actionable recommendations were formulated. Sharing the matrix of recommendations to both health and non-health sectors through consultative meetings was done, which was then analyzed using the three-delays model.

**Results**

During the 2021 MPDSR activity cycle, some of the recommendations included the needs to enhance the capacity of health providers on Emergency Obstetric and Newborn Care, provision of essential equipment, and budget allocation for strengthening the referral system. By April 2022, the local government approved to allocate budget to cover 80% of the recommendations.

**Conclusions**

Improving the capacity of the district MPDSR team in conducting the MPDSR cycle resulted in the formulation of Specific, Measurable, Achievable, Relevant, and Timebound recommendations. Armed with these recommendations, the district team was confident in convincing the stakeholders to take actions. This advocacy work has led to the formulation of the district budget allocation document 2022 for improving MNH quality of care. Routine monitoring is needed to ensure continuation of this quality MPDSR process, and this can be done more effectively using the electronic data system.

## F30.1. Food fortification with folic acid to prevent life-threatening birth defects worldwide

### Vijaya Kancherla^1^, Homero Martinez^2^, Scott Montgomery^3^, Jeffrey Blount^4^

#### ^1^Emory University; ^2^Nutrition International; ^3^Food Fortification Initiative; ^4^University of Alabama-Birmingham

*BMC Proceedings 2024*, **18(5):**F30.1

Submission ID #: IMNHC1051

Panel ID# (if applicable): DYRON901051

**Panel Description**

Birth defects are one of the leading causes of stillbirths and child mortality globally. There is a global inequity in birth defects prevention through proven public health interventions such as staple food fortification with folic acid, an essential micronutrient. We present the epidemiology of neural tube birth defects that are largely preventable, and the method of preventing them through food fortification. Nutrition International and Food Fortification Initiative will present their work in achieving global prevention of neural tube defects. We will also present a story of advocacy, featuring the Global Alliance for Prevention of Folic Acid-Preventable Spina Bifida (GAPSBiF). These are two global organizations that are leading efforts on prevention through active advocacy. All the talks in this panel will cover the importance of primary prevention of major birth defects thus improving child health, especially in low- and middle-income countries.

## F30.2. Food fortification initiative: providing technical guidance to countries to implement large-scale food fortification

### Scott Montgomery^1^, Vijaya Kancherla^2^

#### ^1^Food Fortification Initiative; ^2^Emory University

*BMC Proceedings 2024*, **18(5):**F30.2

Submission ID #: IMNHC1056

Panel ID# (if applicable): DYRON901051

**Background**

Inadequate intake of micronutrients like folic acid can lead to neural tube defects and other serious health consequences. The large-scale fortification of wheat flour, maize flour, and rice with folic acid is a proven, cost-effective intervention to prevent folate deficiency in women of reproductive age and consequences like neural tube defects. The Food Fortification Initiative (FFI) is a public-private-civic partnership that has mapped the opportunity for grain fortification worldwide and provides technical guidance to countries to implement fortification programs. FFI’s goal is to help eliminate micronutrient deficiencies in every country in the world where industrially milled cereal grain is commonly consumed.

**Methods**

Before FFI begins working in a country, FFI uses data to determine two essential requirements: demonstrated need for fortification and the potential to make a positive impact on health through fortified grains. FFI’s data comes from varied sources and methods that include consumption and milling analyses, nutrition needs assessments, market analyses, political readiness assessments, systematic reviews, and partner interviews. Once an opportunity for fortification is determined, FFI uses a four-stage phased approach to help countries plan, implement, and monitor a fortification program that can generate and sustain large-scale impact.

**Results**

When providing technical assistance, FFI uses a four-stage approach: explore and engage, map the context, design and develop, and monitor for compliance and impact. To ensure that fortification programs have a positive health impact on the population and prevent neural tube defects, FFI encourages countries to create standards and mandatory legislation for folic acid fortification. As of September 2022, 92 countries currently mandate the fortification of wheat flour, maize flour, or rice. In 2021, FFI found that 31%, 30%, and 1% of industrially milled wheat flour, maize, and rice, respectively, is fortified.

**Conclusions**

FFI’s strategic approach to scaling grain fortification, which is based on two decades of experience conducting research and providing on-the-ground assistance, offers a replicable method to building and strengthening fortification programs. By relying on data to identify where the needs and opportunities are greatest, FFI helps high-priority countries build mandatory fortification programs that improve the lives of millions and prevent neural tube defects.

## F30.3. Prevention of neural tube defects by improving folate status through large-scale food fortification: a global perspective

### Homero Martinez^1^, Vijaya Kancherla^2^

#### ^1^Nutrition International; ^2^Emory University

*BMC Proceedings 2024*, **18(5):**F30.3

Submission ID #: IMNHC1054

Panel ID# (if applicable): DYRON901051

Note the full manuscript published in *Springer Link* in April 2023:

(https://link.springer.com/article/10.1007/s00381-023-05913-4?code=ff97a0ec-bbb1-4192-b6d0-a9400c423846&error=cookies_not_supported)

## F30.4. Status of global folic acid-preventable Spina Bifida and Anencephaly

### Vijaya Kancherla

#### Emory University

*BMC Proceedings 2024*, **18(5):**F30.4

Submission ID #: IMNHC1052

Panel ID# (if applicable): DYRON901051

**Background**

Spina bifida and anencephaly are major neural tube defects largely preventable through maternal periconceptional intake of folic acid. We estimated the global proportion of folic acid-preventable spina bifida and anencephaly (FAP SBA) prevented through mandatory folic acid fortification of cereal grains, including wheat flour, maize flour, and rice, at the end of year 2020, a point marking the 30th anniversary of the publication of landmark British Medical Research Council (MRC) study providing unequivocal knowledge on folic acid’s FAP SBA prevention potential.

**Methods**

The Food Fortification Initiative database was used to identify countries with mandatory fortification policies with folic acid added to cereal grains. We examined status of FAP SBA prevention assuming mandatory folic acid fortification at 200 mcg/day fully protects against FAP SBA and reduces their prevalence to a minimum achievable rate of 0.5 cases/1,000 live births. The country-specific annual number of FAP SBA cases prevented was quantified as the maximum prevention potential possible for any of the fortified grain examined (wheat flour/maize flour/rice) based on the level of total folic acid consumption from fortified grain examined and the fortification program coverage.

**Results**

Our analysis showed that 61,677 FAP SBA cases were prevented in the year 2020 through mandatory folic acid fortification of cereal grains in 58 countries, translating to 22% prevention of total possible FAP SBA prevention globally. Many countries in Africa, Asia, and Europe are yet to implement fortification. In 2020, 30 years after the Medical Research Council study was published, 218,270 preventable FAP SBA cases still occurred globally.

**Conclusions**

Global prevention efforts for FAP SBA are inadequate even after three decades of knowledge on their prevention. Universal mandatory fortification of staples should be urgently implemented to prevent thousands of FAP SBA and associated elective terminations, stillbirths, and child mortality.

## F31.1. Ending the silence on stillbirths: critical data gaps, challenges, and solutions

### Lucia Hug^1^, Danzhen You^1^, Rakhi Dandona^2^, Allisyn Moran^3^, Imbulana Jayaratne^4^

#### ^1^United Nations Children’s Fund; ^2^Public Health Foundation of India; ^3^World Health Organization; ^4^Ministry of Health, Sri Lanka

*BMC Proceedings 2024*, **18(5):**F31.1

Submission ID #: IMNHC1357

Panel ID# (if applicable): DYSRS771357

**Panel Description**

Every 16 seconds, a baby is stillborn. This amounts to an estimated 2 million stillborn babies globally every year. These deaths reach far beyond the loss of life. Women and their families often face traumatic, long-lasting impacts after experiencing a stillbirth. Profound psychological suffering and stigma from their communities are common.

Even more tragically, most of these deaths could have been avoided with high-quality care during the antenatal period and birth. More than 40 percent of all stillbirths occur during labor; many could have been prevented with improved monitoring and access to emergency obstetric care.

Despite its magnitude, these losses remain a neglected issue. Worldwide, data on stillbirths are largely absent, resulting in serious gaps in service delivery for pregnant women and poor support services for mourning mothers. Among the 195 countries for which the United Nations Inter-agency Group for Child Mortality Estimation (UN IGME) generates stillbirth estimates, nearly a third have either no stillbirth data or no quality data.

These gaps in counting and reporting of stillbirths must be urgently addressed.

The panel will address three key questions:

1. Which critical data gaps must be closed to end preventable stillbirths?
2. Which challenges stand in the way of collecting stillbirth data?
3. Where do we see innovative solutions to improved stillbirth data availability and quality?

The session will bring together governments, international organizations, and civil society to share expertise and experience, highlight best practices, and provide practical recommendations on collecting stillbirth data and improving evidence to end preventable stillbirths.

## F31.2. International Classification of Diseases (ICD) 11 and perinatal audit

### Allisyn Moran^1^, Lucia Hug^2^, Hannah Blencowe^3^

#### ^1^World Health Organization; ^2^United Nations Children’s Fund; ^3^London School of Hygiene & Tropical Medicine

*BMC Proceedings 2024*, **18(5):**F31.2

Submission ID #: IMNHC1442

Panel ID# (if applicable): DYSRS771357

**Background**

There have been persistent challenges in the understanding and application of the standard ICD definitions for the recording of fetal deaths and stillbirths. In view of substantial misclassification between stillbirths and neonatal deaths, and stillbirths and miscarriages, especially around the threshold of viability, collecting comparable data for all births is imperative to monitor data quality and trends over time and an important first step in any data system before progressing to classifying cause of death.

**Methods**

As part of the stillbirth estimation process in 2018, the WHO, UNICEF and the London School of Hygiene & Tropical Medicine reviewed current ICD guidance and commonly used definitions of stillbirth and developed a proposal to clarify definitions in ICD-11 to improve the consistency and comparability of stillbirth data. The proposal was then reviewed and revised by the Core Stillbirth Estimation Group, the technical advisory group of United Nations Inter-Agency Group for Child Mortality Estimation. Further discussions were undertaken by the Mother and Newborn Information for Tracking Outcomes and Results advisory group to achieve a consensus on a revised definition, and a proposal was submitted to ICD-11 for consideration.

**Results**

Internationally it is now advised by WHO and others that vital status, gestational age at birth, and birthweight be recorded for all births—whether liveborn or stillborn. The following recommendations were adapted by ICD-11 to increase the programmatic relevance of stillbirth data and allow for comparable reporting between stillbirths and neonatal deaths.

- Use gestational age at birth criteria in all definitions where available in preference to birthweight.
- Make clearer the distinction between “fetal death” and “stillbirth.”
- Make clearer the guidance regarding signs of life at birth.
- Make explicit that “induced abortions” should not be classified as a “stillbirth,” even when they occur beyond the gestational age reporting threshold for stillbirths.

**Conclusions**

The revision of the ICD-11 definitions and classifications are a step forward for improving data consistency and quality for stillbirths. WHO, UNICEF, and partners are actively working with countries and regions to strengthen data systems, including civil and vital registration, to incorporate these revisions.

## F31.3. Stillbirth surveillance and data compilation in Sri Lanka

### Imbulana Jayaratne^1^, Lucia Hug^2^

#### ^1^Ministry of Health, Sri Lanka; ^2^United Nations Children’s Fund

*BMC Proceedings 2024*, **18(5):**F31.3

Submission ID #: IMNHC1434

Panel ID# (if applicable): DYSRS771357

**Background**

Sri Lanka, being a low- and middle-income country, reports impressive maternal and child health (MCH) indices. Sri Lanka’s civil birth and death registration system, which also registers stillbirths, was started in 1887. A Reproductive Health Information Management System (RHIMS) was introduced in 1986, with the number of stillbirths from field level reported routinely in RHIMS. A systematic hospital-based perinatal death surveillance and response (PDSR) was started in 2006. Registration of stillbirths with civil registration and vital statistics (CRVS) at hospitals was strengthened in 2015. A well-organized field MCH service delivery system is in place countrywide with an average 320,000 live births annually.

**Methods**

Under the PDSR, all specialized hospitals (with an obstetrician/pediatrician) are required to document all stillbirths and early neonatal deaths, review them monthly at a hospital stakeholder meeting, and enter data into a web-based perinatal death registry.

For RHIMS, stillbirth data are collected in a paper-based format by Public Health Midwives (PHM). In the year 2020, RHIMS reported 1,815 stillbirths from 347 divisional health units. We explored the possibility of initiating a population-based stillbirth registry capitalizing on the routine reporting of stillbirths by PHM. A Google Form-based model using a user-friendly tri-lingual interface to upload 2020 stillbirth data was introduced in early 2021. Different categories of field health staff entered data. Stillbirth data reported to CRVS was obtained from Registrar General’s Department.

**Results**

In the year 2020, stillbirths were reported to PDSR (*n*=1288) and CRVS (*n*=899). In RHIMS, all health units entered data for 1,812 (99%) stillbirths. Cases > 28 weeks of gestation or weight > 1000g (*n*=1512) were selected. Fresh (*n*=903, 59.7%) and macerated (*n*=516, 34.1%) stillbirths were in < 33 weeks (*n*=518, 34.3%) and > 37 weeks (*n*=322, 21.3%) of gestation at specialized (*n*=1,413, 93.5%) and private (*n*=45, 3.0%) hospitals and at home (*n*=7, 0.5%). Sri Lanka reported a stillbirth rate of 6.1 per 1,000 births.

**Conclusions**

Sri Lanka has multiple stillbirth data sources when similar countries struggle to have at least a single source. An online simple format is feasible and effective for counting and gathering essential information on stillbirths at population level at low-income country context. Capture of stillbirths at field level appears to be relatively higher. Triangulation of different data sources is effective in compiling valid stillbirth statistics.

## F31.4. Improving documentation of stillbirths in community surveys to facilitate stillbirth prevention

### Rakhi Dandona^1^, Lucia Hug^2^

#### ^1^Public Health Foundation of India; ^2^United Nations Children’s Fund

*BMC Proceedings 2024*, **18(5):**F31.4

Submission ID #: IMNHC1425

Panel ID# (if applicable): DYSRS771357

**Background**

Community surveys are the major source of data on stillbirths in the developing countries. However, there are major data adequacy and quality related challenges in documenting the magnitude of and risk factors for stillbirths in the available large-scale surveys. This critical gap hinders the progress for stillbirth prevention in countries that are estimated to have large burdens of stillbirths.

**Methods**

A variety of community surveys are conducted, including the Demographic and Health Survey, specific mortality surveys that capture stillbirths, and some surveys that capture neonatal deaths, but not stillbirths. We reviewed the design, questionnaire, and data collection methods for major surveys to document issues that hinder robust estimation of stillbirth rate and risk factors for stillbirths.

**Results**

Documentation of stillbirth magnitude is affected by the documentation of gestation period, confirmation of stillbirth, and how a stillbirth is differentiated from a live birth that died soon after birth. Importantly, documentation is also affected by the extent of importance given to stillbirth documentation by the survey leads. Training of interviewers in ascertaining stillbirth in the context of stigma is also important for increasing robustness of stillbirth documentation. It is important to note that stillbirth registration is not documented in most surveys, which hinders understanding of underreporting of stillbirths in the vital registration system. Most surveys do not capture details of pregnancy that resulted in a stillbirth and focus on pregnancies resulting in a live birth, as the primary aim is to address neonatal mortality. Inclusion of pregnancies that resulted in stillbirth and capturing additional risk factors relevant for stillbirths is crucial to address the gap toward stillbirth prevention. Questions to differentiate antepartum and intrapartum stillbirths are needed to facilitate specificity in action. The utility of verbal autopsy in stillbirth capture will be discussed.

**Conclusions**

The countries with large burdens of stillbirths cannot sustain action to reduce stillbirths based on inadequate data. The poor documentation of magnitude and risk factors for stillbirths in surveys that focus only on live births and neonatal deaths are a missed opportunity. Little investment is needed to expand the scope of these surveys to include stillbirths.

## F31.5. Global perspective on stillbirth burden and data gaps

### Lucia Hug, Danzhen You

#### United Nations Children’s Fund

*BMC Proceedings 2024*, **18(5):**F31.5

Submission ID #: IMNHC1370

Panel ID# (if applicable): DYSRS771357

**Background**

Stillbirths are a major public health issue and a sensitive marker of the quality of care around pregnancy and birth. A crucial step to prevent stillbirths is measuring the risk and burden of stillbirths across countries. With our latest United Nations Inter-agency for Child Mortality Estimatio (UN IGME) estimates we estimated levels and trends in stillbirth rates at 28 weeks or more of gestation from 2000 to 2021, assessed progress over time, and identified data gaps.

**Methods**

We gathered all available information relevant to stillbirth rate estimation into a dataset containing 3,200 country-year datapoints from 173 countries, including data from civil registration and health information systems, household surveys, and population studies. We estimated country-specific stillbirth rates for all countries from 2000 to 2021 using a statistical model that also addresses data gaps and data quality issues.

**Results**

In 2021, an estimated 1.9 million babies were stillborn at 28 weeks or more of gestation, with a global stillbirth rate of 14 stillbirths per 1,000 total births. Stillbirth rates varied widely across the world, regions, and countries, and progress in reducing stillbirth rates was slow in many countries and regions in sub-Saharan Africa and South Asia. More than 50 countries are off track to meet the Every Newborn Action Plan (ENAP) target by 2030—many of them in sub-Saharan Africa. Countries and regions with the highest rates and slow progress tend to lack quality data. High quality stillbirth data are in particularly short supply in sub-Saharan Africa and South Asia regions, which accounted for only 18 percent of the stillbirth data used in the model, but bore the burden of three quarters of the estimated stillbirths.

**Conclusions**

Progress in reducing the stillbirth rate has been slow compared with declines in child mortality. Accelerated improvements are most needed in the countries with high stillbirth rates that are at risk of not meeting the ENAP target on time. Increased efforts are needed to raise public awareness of stillbirths, to improve data collection and to prevent stillbirths. It’s not possible to assess progress going forward or understand public health priorities locally without investment in gathering information about stillbirths.

## F32.1. Paving the way forward for intravenous iron treatment for anemia in pregnancy: insights from a hybrid trial in Nigeria

### Bosede Afolabi, Ochuwa Babah, Mobolanle Balogun, Opeyemi Akinajo

#### College of Medicine University of Lagos

*BMC Proceedings 2024*, **18(5):**F32.1

Submission ID #: IMNHC536

Panel ID# (if applicable): DZZIU96536

**Panel Description**

Anemia in pregnancy is a major public health burden with a higher incidence in low- and middle-income countries such as Nigeria. It is mostly caused by iron deficiency and has significant implications on maternal and neonatal health. This panel examines initial findings from an ongoing superiority, randomized controlled trial, “Intravenous versus oral iron for iron deficiency anemia in pregnant Nigerian women (IVON trial),” with both effectiveness and implementation outcomes. In this four-abstract panel, we present recent estimates of prevalence of and risk factors for iron deficiency among pregnant women with moderate or severe anemia in the two most populated states of Nigeria (Kano and Lagos states). This abstract goes on to describe the characteristics of pregnant women with iron deficiency anemia in the study sites. In the second abstract, we capture perception of women on intravenous iron treatment for anemia in pregnancy in the IVON trial. Building on this, we report considerations for acceptability of intravenous iron treatment for iron deficiency anemia in pregnancy in Nigeria based on insights from a qualitative study with pregnant women, family decision-makers, and health care providers. Finally, being that large scale trials such as ours are rare in the low- and middle-income countries maternal health space, we share lessons learned from the conduct of this trial and how contextual solutions to challenges were generated to assure successful trial implementation.

## F32.2. Lessons learned from the conduct of a large clinical trial among pregnant women with anemia in the two most populated states of Nigeria

### Bosede Afolabi^1^, Ochuwa Babah^1^, Mobolanle Balogun^1^, Opeyemi Akinajo^1^, Victoria Adaramoye^1^, Titilope Adeyemo^1^, Aduragbemi Banke-Thomas^2^, Rachel Quao^1^, Ibraheem Abioye^3^, Hadiza Galadanci^4^, Nadia Sam-Agudu^5^, Elin Larsson^6^, Kristi Annerstedt^6^, Claudia Hanson^6^, Lenka Benova^7^

#### ^1^College of Medicine University of Lagos; ^2^London School of Hygiene & Tropical Medicine; ^3^Afya Technologies; ^4^Bayero University; ^5^University of Maryland School of Medicine; ^6^Karolinska Institutet; ^7^Institute of Tropical Medicine

*BMC Proceedings 2024*, **18(5):**F32.2

Submission ID #: IMNHC727

Panel ID# (if applicable): DZZIU96536

**Background**

The ongoing Iron-deficiency Anaemia in Pregnant Nigerian Women (IVON) trial—a superiority, randomized controlled trial recruiting 1,056 pregnant women—is the first of its kind in Nigeria. It aims to compare the effectiveness and safety of intravenous ferric carboxymaltose versus oral ferrous sulphate for pregnancy-related iron deficiency anemia in Lagos and Kano states in Nigeria. We are also measuring implementation outcomes of acceptability, feasibility, fidelity, and cost-effectiveness for intravenous ferric carboxymaltose. We describe the lessons learned before and during the performance of the clinical trial and how they guided our approach in the two states.

**Methods**

We engaged and consulted with government officials overseeing maternal and child health programs in the study states who provided guidance on study approach to suit health system context, before commencing the trial. We also conducted a formative study, assessing for barriers and facilitators of successful intervention implementation in our study states. On commencement of the trial, we recorded and discussed all major issues discovered during our fortnightly virtual meeting sessions and physical monitoring sessions.

**Results**

Advice about ensuring clear information to the participants and outcome assessors was adhered to by developing detailed pictorial study posters and flyers and ensuring more frequent trainings on the study intervention. Discovery of loss to follow up because of home delivery and default in clinic visits were mitigated by developing a protocol for community tracking and verification of locator information at study visits. We also adapted our capacity to allow for collection of samples and data from women at home, where necessary. Continuous engagement and education were provided to study participants and family members to address some misconceptions about the intervention.

**Conclusions**

Large clinical trials in complex low- and middle-income countries like Nigeria require strong stakeholder engagement, innovation, and flexibility to attain success.

## F32.3. Acceptability of intravenous iron treatment for iron deficiency anemia in pregnancy: insights from a qualitative study with pregnant women, family decision-makers, and health care providers in Nigeria

### Opeyemi Akinajo^1^, Bosede Afolabi^1^, Ochuwa Babah^1^, Mobolanle Balogun^1^, Victoria Adaramoye^1^, Titilope Adeyemo^1^, Aduragbemi Banke-Thomas^2^, Rachel Quao^1^, Ibraheem Abioye^3^, Hadiza Galadanci^4^, Nadia Sam-Agudu^5^, Elin Larsson^6^, Kristi Annerstedt^6^, Claudia Hanson^6^, Lenka Benova^7^

#### ^1^College of Medicine University of Lagos; ^2^London School of Hygiene & Tropical Medicine; ^3^Afya Technologies; ^4^Bayero University; ^5^University of Maryland School of Medicine; ^6^Karolinska Institutet; ^7^Institute of Tropical Medicine

*BMC Proceedings 2024*, **18(5):**F32.3

Submission ID #: IMNHC724

Panel ID# (if applicable): DZZIU96536

**Background**

Anemia in pregnancy bears a significant burden of maternal morbidity and mortality in sub-Saharan Africa, with prevalence ranging from 25% to 45% in Nigeria. Current treatment methods involve the use of daily oral iron, which is not always effective and has low adherence. Intravenous (IV) iron administered once offers an alternative treatment to pregnant women with moderate or severe iron deficiency. This qualitative study explores the facilitators and barriers of the acceptability of IV iron for the treatment of anemia in pregnancy in Kano and Lagos, Nigeria.

**Methods**

We purposively sampled stakeholders, including pregnant women, family decision-makers (matriarchs and male partners), and healthcare providers (HCPs). The study was conducted at the pre-intervention phase of a hybrid clinical trial titled Intravenous versus oral iron for iron deficiency anemia in pregnant Nigerian women (IVON trial) in 10 healthcare facilities across three levels of the system. Semistructured topic guides were used in 12 focus group discussions with 140 participants and 29 key informant interviews. Sekhon et al.’s Theoretical Framework of Acceptability was used to guide the analysis, and content analysis was performed.

**Results**

Women and decision-makers stated that the existing trust between pregnant women and the HCPs and the perceived advantages of IV iron over oral therapy, such as immediate response to treatment and no concerns with adherence, are enablers that would facilitate their decision to accept the intervention. Fear of needles and drug reactions, presumed high cost, and health talk not centered around IV iron were deemed potential barriers. HCPs highlighted IV iron as a preferred alternative to blood transfusion, given its advantages over oral iron. However, HCPs emphasized the need for adequate training for safe administration and concerns over additional workload in light of the already overstretched human resources. Potential irregular supply chain for IV iron in the hospital was also a major concern.

**Conclusions**

IV iron, if proven effective, has a high potential to be a preferred method to treat anemia in pregnancy in Nigeria. However, for this treatment to be widely accepted, HCP training, optimization of antenatal care delivery, tailoring existing health talk to include IV iron, and ensuring affordability need to be considered to improve the acceptability of this treatment.

## F32.4. Patients’ perception of intravenous iron treatment for anemia in pregnancy: findings from the IVON trial

### Mobolanle Balogun^1^, Opeyemi Akinajo^1^, Bosede Afolabi^1^, Ochuwa Babah^1^, Victoria Adaramoye^1^, Titilope Adeyemo^1^, Aduragbemi Banke-Thomas^2^, Rachel Quao^1^, Ibraheem Abioye^3^, Hadiza Galadanci^4^, Nadia Sam-Agudu^5^, Elin Larsson^6^, Kristi Annerstedt^6^, Claudia Hanson^6^, Lenka Benova^7^

#### ^1^College of Medicine University of Lagos; ^2^London School of Hygiene & Tropical Medicine; ^3^Afya Technologies; ^4^Bayero University; ^5^University of Maryland School of Medicine; ^6^Karolinska Institutet; ^7^Institute of Tropical Medicine

*BMC Proceedings 2024*, **18(5):**F32.4

Submission ID #: IMNHC717

Panel ID# (if applicable): DZZIU96536

**Background**

Intravenous iron corrects anemia faster and in fewer doses than oral iron, requiring less patient-provider interaction, a situation that is ideal in low- and middle-income countries where loss to follow up is common. Patients’ perception of the medication would determine its uptake in the health care system. This study aims to determine the perception of women who received intravenous iron for the treatment of anemia in pregnancy in the IVON trial.

**Methods**

Exit surveys were conducted among women with anemia in pregnancy randomized to receive intravenous iron in the IVON trial. This analysis presents findings from a subset of study participants who received intravenous iron, completed the study, and were selected in an alternate manner to participate in the survey. The women were approached after their last study visit at six weeks postpartum. Data was collected using interviewer-administered questionnaires by research assistants not affiliated with the trial after obtaining informed consent.

**Results**

As the present stage of the trial, 85 women who received intravenous iron had been approached to participate in the exit survey, and 78 agreed to participate. Most respondents (83.3%) felt that all the time the providers explained what had been done, spoke in an understandable language (87%), explained why they were giving the medicine (89.7%), and took the best care of them as they could (89.7%). Regarding the treatment, most respondents (80.8%) had no challenge with treatment administration and had no side effects (94.9%). Overall, 76.9% of respondents rated the information and support received from the providers as excellent and 71.8% would very strongly recommend intravenous iron to a family member.

**Conclusions**

Most of the women studied had a positive perception about the providers of intravenous iron, the treatment administration, and information about the treatment. Beyond trial settings, it is imperative that future scale-up of intravenous iron use in real world settings are provided in ways that are satisfactory to end-users.

## F32.5. Prevalence of and risk factors for iron deficiency among pregnant women with moderate or severe anemia in Nigeria: a cross-sectional survey

### Ochuwa Babah^1^, Mobolanle Balogun^1^, Opeyemi Akinajo^1^, Bosede Afolabi^1^, Victoria Adaramoye^1^, Titilope Adeyemo^1^, Aduragbemi Banke-Thomas^2^, Rachel Quao^1^, Ibraheem Abioye^3^, Hadiza Galadanci^4^, Nadia Sam-Agudu^5^, Elin Larsson^6^, Kristi Annerstedt^6^, Claudia Hanson^6^, Lenka Benova^7^

#### ^1^College of Medicine University of Lagos; ^2^London School of Hygiene & Tropical Medicine; ^3^Afya Technologies; ^4^Bayero University; ^5^University of Maryland School of Medicine; ^6^Karolinska Institutet; ^7^Institute of Tropical Medicine

*BMC Proceedings 2024*, **18(5):**F32.5

Submission ID #: IMNHC704

Panel ID# (if applicable): DZZIU96536

**Background**

Anemia in pregnancy is a common medical condition associated with increased risk of complications to the woman and her fetus. Its most common cause is iron deficiency. Globally, routine daily oral iron administration in pregnancy is a widespread treatment modality. However, poor adherence to oral supplementation occurs frequently, and there is paucity of studies on dietary risk factors. This study measures the prevalence of iron deficiency anemia (IDA) in pregnant women with moderate or severe anemia in Nigeria (Lagos and Kano states) and examines dietary risk factors for IDA.

**Methods**

This cross-sectional study includes 428 pregnant women attending facility-based antenatal care at 20-32 weeks gestation, diagnosed with moderate or severe anemia (hemoglobin concentration < 10g/dL). Data on sociodemographic and obstetric characteristics were collected on REDCap through an online questionnaire. Frequency of consumption of common food items was assessed using a pre-tested food frequency questionnaire. Participants’ serum ferritin concentration was also assayed to determine their iron status, with cut off set at 30ng/mL for diagnosis of IDA. Risk factors were assessed using multivariable logistic regression. Dietary risk factors were adjusted for confounders such as age, ethnicity, and socioeconomic status. Data analysis was conducted using Stata version 16.0.

**Results**

The mean age of participants in this study was 29 ±6 years. Prevalence of IDA was 41.2% (95% CI: 36.4–46.1). Among women with moderate/severe anemia, frequent ingestion of edible kaolin clay almost every day increased odds of IDA (aOR 4.38, 95% CI 1.28–14.95), frequent consumption of green vegetables almost every day protects against IDA (aOR 0.26, 95% CI 0.07–0.93), and eating red meat at least every 2–3 weeks was found to be protective against IDA (aOR 0.29, 95% CI 0.09–0.89).

**Conclusions**

Prevalence of IDA among pregnant women with moderate or severe anemia in Nigeria is high. Consumption of edible kaolin clay and low intake of red meat and green vegetables are associated with IDA in pregnant women with moderate or severe anemia in Nigeria. Health education and dietary advice and modification could play a significant role in reducing the high prevalence of IDA among this category of women.

## O33. Improving newborn health outcomes in Yemen through community-led kangaroo mother care

### Areej Banajah, Hisham Binraood, Suaad Al-Hetari, Nadia Olson, Herman Willems, Yibeltal Tebekaw Bayou

#### John Snow, Inc.

*BMC Proceedings 2024*, **18(5):**O33

Submission ID #: IMNHC2634

**Background**

The Systems, Health, and Resiliency Project (SHARP) implemented a pilot program called My Community, My Home (MCMH) in the Al-Maqatera District of Lahj Governorate in Yemen to improve newborn health outcomes through community-led kangaroo mother care (KMC). The program aimed to address the significant issue of newborn mortality in Yemen, which is estimated at 27 deaths per 1,000 live births.

**Methods**

The MCMH program was implemented for seven months in a mountainous region with challenges for mothers in accessing health care. The Systems, Health, and Resiliency Project provided training to community midwives and 20 health workers in the nearest health facilities within the district on the use of KMC. Mothers with low birth weight newborns also received a “KMC Kit,” containing items such as a baby carrier.

**Results**

Since the launch of the MCMH program, 69 newborns have been supported, with 50 successfully discharged and with improved health outcomes for every baby. The average length of participation in the KMC program for discharged patients was 112 days, and all discharged babies received exclusive breastfeeding during their participation. Among discharged infants, 62% achieved all six thresholds of continued KMC, weight gain, exclusive breastfeeding, and routine immunizations during the course of 16 weeks. Participating mothers reported increased confidence in the effectiveness and cost-efficiency of KMC and improved health conditions for their newborns.

**Conclusions**

The MCMH program demonstrated that community-led KMC is a cost-effective and effective method of improving newborn health outcomes in resource-limited settings. The program was well-received by participating mothers and their families, who reported improved health conditions for their newborns. The program’s success highlights the importance of community engagement in addressing gaps in maternal and newborn health, particularly in rural, difficult to reach, and conflict-affected areas. This work has implications for similar programs in other resource-limited settings and has significance for addressing gaps in equity in maternal and newborn health care. By training community midwives and engaging local leaders and community members in the promotion of KMC, the program increased access to care for low birth weight newborns in a resource-limited setting, potentially reducing inequities in care for these vulnerable infants.

## O34. From global to local: accelerating access to essential postpartum hemorrhage medicines

### Sara Rushwan^1^, Ahmet Metin Gülmezoglu^1^, Lester Chinery^1^, Rachel Gooden^2^, Joyce Ng'ang'a^3^, Tabeth Chitimbe^3^

#### ^1^Concept Foundation; ^2^The International Federation of Gynecology and Obstetrics; ^3^WACI Health

*BMC Proceedings 2024*, **18(5):**O34

Submission ID #: IMNHC2304

**Background**

Concept Foundation, WACI Heath, the International Federation of Gynecology and Obstetrics (FIGO), and the International Confederation of Midwives (ICM) conducted an advocacy initiative with the purpose of accelerating access to quality-assured heat-stable carbetocin for prevention, and tranexamic acid for treatment of postpartum hemorrhage in a total of fifteen sub-Saharan African countries. The scope of work covered updating national policies, developing contextualized clinical protocols and job aids, and assessing the feasibility of introducing the protocols into health facilities through pilot implementation research. The objectives of the initiative were to raise awareness of the importance of quality maternal health medicines and support the national revision and update of post-partum hemorrhage guidelines and Essential Medicines Lists to include heat-stable carbetocin and tranexamic acid, in line with recent WHO post-partum hemorrhage recommendations.

**Methods**

Sub-Saharan African countries in two Regional Economic Communities were selected based on an assessment of willingness and capacity through engaging with Ministries of Health. Advocacy initiatives for policy change included engagements with Health Secretariats of the Regional Economic Communities, civil society organizations, and conducting virtual regional and national workshops. Activities for national protocol development included publishing a generic PPH prevention and treatment protocol , convening country workshops led by an expert working group to produce a national PPH protocol adapted from the generic protocol, and working with in-country designers to develop complementary job aids.

**Results**

Ten out of fifteen countries updated their national guideline, Essential Medicines List, or both, to include heat-stable carbetocin and tranexamic acid (Burkina Faso, DRC, Ethiopia, Ghana, Ivory Coast, Liberia, Rwanda, Sierra Leone, South Sudan, and Uganda). Six countries (Ethiopia, Ghana, Rwanda, Sierra Leone, South Sudan, and Uganda) developed a national clinical protocol on PPH prevention and management, and seven countries (Burkina Faso, Ethiopia, Ghana, Liberia, Rwanda, Sierra Leone, and Uganda) developed complementary job aids.

**Conclusions**

The significance of the project is a shortened interval in getting WHO post-partum hemorrhage recommendations adopted within countries and starting to close the gap between global and local through updating national policies and tailoring clinical protocol/job aids development to the country context. Forming expert working groups and engaging with civil service organizations helped tackle issues of equitable access to essential medicines by advocating for PPH to remain high on the Ministries of Health’s agenda. The unique value of the project was concurrent multi-sectoral engagement.

Disclaimer: The activities in this project were supported by funding from MSD, through its MSD for Mothers initiative and are the sole responsibility of the authors. MSD for Mothers is an initiative of Merck & Co., Inc., Rahway, NJ, U.S.A

## O35. Effectiveness of a multi-country implementation-focused network on quality of care: delivery of interventions and processes for improved maternal, newborn, and child health outcomes

### Nehla Djellouli^1^, Tim Colbourn^1^, Yusra Shawar^2^, Kasonde Mwaba^1^, Kohenour Akter^3^, Gloria Seruwagi^4^, Asebe Amenu Tufa^5^, Geremew Gonfa^5^, Kondwani Mwandira^6^, Jeremy Shiffman^2^, Mike English^7^

#### ^1^University College London; ^2^Johns Hopkins Bloombeg School of Public Health; ^3^Diabetic Association of Bangladesh; ^4^Makerere University; ^5^Ethiopian Public Health Institute; ^6^Parent and Child Health Initiative; ^7^University of Oxford

*BMC Proceedings 2024*, **18(5):**O35

Submission ID #: IMNHC2258

**Background**

The Network for Improving Quality of Care for Maternal, Newborn and Child Health (QCN) aims to work through learning, action, leadership, and accountability. We aimed to evaluate the effectiveness of QCN in all four of these areas.

**Methods**

Our mixed method evaluation focuses on the global level of QCN and in four of the 11 QCN countries: Bangladesh, Ethiopia, Malawi, and Uganda. Between 2019 and 2022 we conducted two to four iterative rounds of data collection in each country involving stakeholder interviews, observations of best and worst performing hospitals, a survey of QCN members, and document review. All qualitative data was analyzed in NVivo 12 using a common coding framework developed from underlying theories on: network effectiveness; capability, opportunity, and motivation for behavior change; and the QCN proposed theory of change considering interactions with environment, structure, process, and outcomes. Survey data was analyzed with descriptive statistics and linear regression of calculated domain scores capturing different areas of respondents’ perception of QCN.

**Results**

The global level of QCN, led by the WHO secretariat, was effective in bringing together network countries’ governments and global actors by providing online and in-person platforms for communication and learning. Within QCN countries, various interventions were delivered in “learning districts,” though often separately by different partners in different locations and disrupted by the pandemic. Governance structures for quality of care were set-up, some preceding QCN, and were found to be stronger and better (though often externally) resourced at national than local levels. Awareness of operational plans and network activities was lower at local than national levels, though increased from 2019 to 2022. Capacity building efforts, including health worker mentorship, were implemented—yet often dependent on implementing partners and donors. Fifteen quality of care indicators were agreed though data collection was challenging, especially for indicators requiring new or parallel systems including those on experience of care. Accountability through community engagement, scorecards, and ombudsmen was encouraged, but these initiatives remained nascent in 2022.

**Conclusions**

Global and leadership elements of QCN have been most effective to date, with action, learning, and accountability more challenging, partner or donor dependent, remaining to be scaled up, and pandemic-disrupted.

## O36. Prevention of infant Respiratory Syncytial Virus (RSV) illness with a Bivalent RSVpreF vaccine in pregnancy: results from a global efficacy trial

### Eric Simões^1^, Iona Munjal^2^, Beate Kampmann^3^, Shabir Madhi^4^, Barbara Pahud^2^, David Radley^2^, Emma Shittu^2^, Kena A. Swanson^2^, William C. Gruber^2^, Annaliesa S. Anderson^2^, Alejandra Gurtman^2^

#### ^1^Children’s Hospital Colorado; ^2^Pfizer Inc; ^3^London School of Hygiene & Tropical Medicine; ^4^MRC Vaccines and Infectious Diseases Analytics Research Unit

*BMC Proceedings 2024*, **18(5):**O36

Submission ID #: IMNHC2252

**Background**

Maternal vaccination is a promising strategy to protect young infants against RSV illness from birth through the period of highest risk.

**Methods**

In this ongoing phase 3, placebo-controlled study, pregnant ≤49-year-olds between 24to 36 weeks’ gestation were randomized 1:1 to receive one RSVpreF 120 μg (RSV subgroups A and B, 60 μg each) vaccine or placebo dose. Vaccine efficacy (VE) in infants was assessed against RSV-positive, medically-attended severe lower respiratory tract illness (RSV-MA-sLRTI) and medically-attended LRTI (RSV-MA-LRTI) occurring ≤180 days after birth; interim analyses (IA) were planned, and a final analysis conducted if either primary endpoint through 90 days indicated statistically significant efficacy. The study was performed in 18 countries, and efficacy was analyzed by country and country economic status.

**Results**

Overall, 7,358 maternal participants were enrolled; 3,682 received RSVpreF and 3,676 received placebo; 3,570 and 3,558 infants were born to mothers who received RSVpreF or placebo, respectively. VE against RSV-MA sLRTI was 81.8% (99.5% CI 40.6-96.3) in infants within 90 days after birth and remained significant through 180 days (VE 69.4% [97.58% CI 44.3-84.1%]). VE against RSV-MA-LRTI at 90 days after birth was 57.1% (99.5% CI 14.7-9.8), which did not meet CI lower bound > 20% criterion. VE against RSV-MA-LRTI was met at 180 days (VE 51.3% [97.58% CI 29.4-66.8%]). RSVpreF was safe and well tolerated by maternal participants, with no safety signals detected in infants through up to 24 months after birth; adverse event incidences were similar in vaccine and placebo groups for mothers and infants.

**Conclusions**

Maternal vaccination with RSVpreF was efficacious at preventing severe LRTI caused by RSV in infants through 6 months of age. Clinically important efficacy was observed for RSV-MA-LRTI until 1 year of age. No safety concerns were observed.

## O37. Feasibility and Impact of group antenatal care, Atlantique Department, Benin, 2020–2022

### Julie Niemczura De Carvalho^1^, Faustin Onikpo^1^, Manzidatou Alao^1^, Maria Idohou Adeyemi^1^, Alexandre Binazon^1^, Stephanie Suhowatsky^2^, Katherine Wolf^2^, Julie Buekens^1^, Peter Winch^3^, Blaise Guezo Mevo^4^, Cyriaque D. Affoukou^4^, Camille Houetohossou^4^, Ahmed Saadani Hassani^5^, Catherine Dentinger^5^, Aurore Ogouyèmi-Hounto6, Julie R Gutman^5^

#### ^1^MCD Global Health; ^2^Jhpiego; ^3^Johns Hopkins University; ^4^Benin Ministy of Health; ^5^Centers for Disease Control and Prevention; ^6^CHU Cotonou

*BMC Proceedings 2024*, **18(5):**O37

Submission ID #: IMNHC2247

**Background**

Benin recommends four antenatal care visits (ANC4) and at least three doses of intermittent preventive treatment of malaria in pregnancy (IPTp3). However, many ANC attendees do not receive IPTp3. Group antenatal care (G-ANC) can improve ANC attendance and coverage of ANC-delivered interventions. We assessed the feasibility of introducing G-ANC and scaling implementation to increase ANC4 and IPTp3 coverage in Atlantique Department.

**Methods**

Forty health facilities in Atlantique Department, Benin, were randomized 1:1 to control and intervention arms. In intervention facilities, women beginning ANC before 24 weeks gestation were invited to participate in five G-ANC meetings with others of similar gestational age in lieu of attending individual ANC. Baseline and endline household surveys were conducted in 2020 and 2022, respectively; consenting women with a live birth in the past 12 months answered questions on timing and number of ANC visits and IPTp doses taken. Focus group discussions were conducted with providers and pregnant women to identify barriers and facilitators to G-ANC participation.

**Results**

Coverage of G-ANC was low; only 11% (140/1,257) of women surveyed at endline attended G-ANC: 15.6% of women in intervention areas and 6.5% of women in control areas (e.g., women obtained ANC outside of their home health facility). Coverage of both ANC4 and IPTp3 was higher among G-ANC attendees than among those attending standard ANC (65.0% vs. 50.6%, *P*=0.0002; 64.0% vs. 50.6%, *P*=0.0038, respectively). However, there was no difference in overall coverage of either ANC4 or IPTp3 when comparing intervention with control clusters (i.e., at the facility level). Focus-group data revealed that barriers to participation were high provider workloads, fees charged for ANC, delayed first ANC visit because of cultural norms, and duration of G-ANC meetings.

**Conclusions**

G-ANC participation improved coverage of ANC4 and IPTp3 among pregnant women compared with individual ANC. However, there was no difference at the facility level, likely because of low participation in G-ANC. Qualitative findings suggest barriers at patient, provider, and system levels affected enrollment. Additional analyses will highlight facilitators and system requirements to improve likelihood of successful G-ANC implementation.

## O38. Estimating the impact of universal coverage of key maternity services on maternal and neonatal health outcomes in Malawi: an individual-based modeling study

### Joseph Collins^1^, Wingston Ng'ambi^2^, Helen Allott^3^, Fannie Kachale^4^, Emmanuel Mnjowe^2^, Sakshi Mohan^5^, Paul Revill^5^, Asif Tamuri^1^, Tara Mangal^6^, Gerald Manthalu^4^, Timothy Hallett^6^, Valentina Cambiano^1^, Joseph Mfutso-Bengo^2^, Andrew Phillips^1^, Tim Colbourn^1^

#### ^1^University College London; ^2^Kamuzu University of Health Sciences; ^3^Liverpool School of Tropical Medicine; ^4^Ministry of Health, Malawi; ^5^University of York; ^6^Imperial College London

*BMC Proceedings 2024*, **18(5):**O38

Submission ID #: IMNHC2214

**Background**

Despite substantial progress, maternal and neonatal morbidity and mortality remain high in Malawi because of variable coverage and quality of health services. The impact universal coverage of key maternity services (UHCMS) could have in Malawi on health outcomes is currently unknown. To estimate this, we developed a systems-based, data-driven, context-specific model of maternal and neonatal health in Malawi.

**Methods**

As an interdisciplinary group of epidemiologists and computer scientists in the United Kingdom and Malawi, we developed an individual-based simulation model of maternal and neonatal health in Malawi. We explicitly model the processes of pregnancy, labor, and puerperium alongside complications that drive poor outcomes, healthcare seeking, and the effect of interventions on maternal and perinatal health. This model is housed within a novel “whole-system, all-disease” model, which simulates demographic characteristics, diseases contributing to ~85% of morbidity and mortality, and health care delivered through the health system. This allows for representation of indirect causes of pregnancy outcomes including malaria and HIV. We calibrated this model to Malawian data and projected and compared outcomes under current service delivery (CSD) and UHCMS between 2023 and 2030.

**Results**

During the 2023–2030 period, UHCMS could avert a total of 346,565 (45%) and 2,232,190 (35%) maternal and neonatal DALYs respectively compared with CSD. By 2030, UHCMS is predicted to reduce the maternal mortality ratio by 44% leading to a maternal mortality ratio of 192 per 100,000 live births in 2030 compared with 340 per 100,000 if CSD continues, with notable reductions in the rate of complications such as postpartum hemorrhage (23%) and eclampsia (20%). Additionally, the neonatal mortality rate could be reduced by 36% in 2030 at 10.4 per 1,000 live births under UHCMS as a result of, for example, reductions in neonatal encephalopathy (20%) and prematurity (16%).

**Conclusions**

UHCMS in Malawi could lead to substantial reductions in maternal and neonatal morbidity and mortality by 2030. However, additional improvements in coverage of other services, such as postabortion care, would be needed to ensure Malawi meets SDG 3.1 on maternal mortality. Our “all disease” framework aims to capture important details possibly missed by other models routinely.

## O39. Intrapartum oral azithromycin to prevent maternal and newborn sepsis or death: a multinational Randomized Controlled Trial (RCT)

### Alan Tita^1^, Elizabeth McClure^2^, Waldemar Carlo^1^

#### ^1^University of Alabama at Birmingham; ^2^RTI International

*BMC Proceedings 2024*, **18(5):**O39

Submission ID #: IMNHC2157

Note the full manuscript was published earlier this year in NEJM: (https://www.nejm.org/doi/full/10.1056/nejmoa2212111)

## P40. Maternal exposure to intimate partner violence and breastfeeding practices of children 0-23 months: findings from the 2018 Nigeria demographic and health survey

### Tope Olubodun^1^, Anteneh Asefa^2^, Aduragbemi Banke-Thomas^3^, Mobolanle Balogun^4^, Ifeoma Okafor^4^, Oluwakemi Odukoya^4^, Lenka Benova^2^

#### ^1^Federal Medical Centre Abeokuta; ^2^Institute of Tropical Medicine; ^3^London School of Hygiene & Tropical Medicine; ^4^College of Medicine, University of Lagos

*BMC Proceedings 2024*, **18(5):**P40

Submission ID #: IMNHC2144

Note the full manuscript published in May 2023 in *JOGHR*:

(https://www.joghr.org/article/75338-maternal-exposure-to-intimate-partner-violence-and-breastfeeding-practices-of-children-0-23-months-findings-from-the-2018-nigeria-demographic-and-hea)

## O41. Avoid equipment graveyards: rigorous process to improve identification and procurement of effective, affordable, and usable newborn devices in low-resource hospital settings

### Elizabeth Asma^1^, Megan Heenan^1^, George Banda^2^, Rebecca Kirby^3^, Lucky Mangwiro^4^, Ali Khalid^5^, Cliff Osoo^5^, Vince Gate^5^, Claudia Ziegler Acemyan^6^, Kara Palamountain^2^, Philip Kortum^6^, Kondwani Kawaza^4^, Maria Oden^2^, Rebecca Richards-Kortum^2^

#### ^1^Rice360 Institute for Global Health Technologies; ^2^NEST360; ^3^Northwestern University Kellogg School of Management; ^4^Kamuzu University of Health Sciences; ^5^Hatch Technologies; ^6^Rice University Psychological Sciences

*BMC Proceedings 2024*, **18(5):**O41

Submission ID #: IMNHC2133

**Background**

Millions of newborns die annually from preventable causes, with the highest rates occurring in Africa. Reducing neonatal mortality requires investment to scale hospital care, which includes providing appropriate technology to care for small and sick newborns. Medical devices designed for high-resource settings often fail to withstand conditions in low-resource hospitals including severe humidity, dust, frequent user turnover, complex maintenance, lack of stable power, or difficulty sourcing expensive consumables. Rigorous evaluation protocols are needed to identify effective, affordable, rugged, and easy-to-use medical devices appropriate for low-resource settings.

**Methods**

We developed an evidence-based technology review process to identify medical devices suitable for small and sick newborn care in low-resource hospitals. The eight-step process consists of: identifying devices needed; defining Target Product Profiles (TPPs); identifying commercially available products that may meet TPPs; conducting research to evaluate technologies against TPPs; performing technical verification testing under laboratory conditions; verifying technical performance after exposure to heat, humidity, dust, and power loss; performing usability evaluations with nurses, and qualifying devices that pass all steps. Devices were purchased, installed, and monitored in newborn wards across Malawi, Tanzania, Kenya, and Nigeria.

**Results**

Of 271 devices considered, only 45 (16.6%) met corresponding TPPs based on desk research. Thirty-nine were purchased and evaluated in the laboratory; five (12.8%) failed to meet TPPs. Thirty-four products passing laboratory evaluation underwent short-term environmental testing; only one (2.9%) device failed. Thirty-seven products underwent usability testing with 127 clinicians; surprisingly, 14 (37.8%) failed to meet TPPs. Twenty-three products passed all evaluations, and 2,197 devices were installed across 65 newborn wards in Malawi, Tanzania, Kenya, and Nigeria between October 2019 and December 2022. Continuous device monitoring reported 74 device failures, with failed devices typically returned to service within two days, resulting in an average up time of 99%.

**Conclusions**

An evidence based device selection process can improve procurement of effective, affordable, and usable newborn care devices for low-resource hospitals and feedback to manufacturers can improve device quality. Similar processes could be adapted beyond newborn care to identify medical devices suitable for implementation in low-resource settings.

## P42. Examining the quality of informed consent in intrapartum clinical trials: a prospective study in Uganda

### Adeline Boatin^1^, Mehreen Zaigham^1^, Paola Del Cueto^2^, Lisa Bebell^2^, Kaitlyn James^2^, Rohini Dutta^1^, Bizu Gelaye^2^, Henry Lugobe^3^, Rwambuka Godfrey Mugyenyi^3^, Jessica Haberer^1^, Joseph Ngonzi^3^

#### ^1^Harvard Medical School; ^2^Mass General Hospital; ^3^Mbarara University of Science and Technology

*BMC Proceedings 2024*, **18(5):**P42

Submission ID #: IMNHC2126

**Background**

Despite the ethical and scientific importance of obtaining quality informed consent in clinical trials, empirical data is lacking on how participants understand research-related information through current informed consent processes, particularly in intrapartum trials from low- and middle-income countries. We aimed to evaluate the quality of consent among trial participants in Uganda, identify domains at risk of misunderstanding, and explore the impact of sociodemographic characteristics on understanding.

**Methods**

This is a sub-study of a hybrid type II effectiveness-implementation trial of wireless vital sign monitoring after emergency cesarean birth in Mbarara, Uganda. Participants recruited into the parent trial from May 2021 to February 2022 were eligible for inclusion. After completing all parent trial procedures, including a baseline questionnaire, participants completed the Quality of Informed Consent (QuIC) survey to assess objective (QuIC-A) and subjective (QuIC-B) understanding of consent. Survey components were scored from 0-100, with 100 indicating complete understanding. Scores were compared using multivariate logistic regression.

**Results**

A total of 583 surveys were available for analysis. Mean QuIC-A score was 60.0 (SD+-12.4, Range 17-90). Low-scoring components included the experimental nature of the research, data confidentiality, and voluntary participation. Higher scores were correlated with household income (*P*< 0.001). Consent obtained < 30 minutes before delivery was associated with a lower score (*P*< 0.01). Mean QuIC-B score was 36.5 (SD+-25.8, Range 0-100). Low-scoring components included understanding of research purposes, procedures, risks and benefits, confidentiality, and study contact information. QuIC-B scores were strongly correlated with higher education level (*P*< 0.001), but were not correlated with income (*P*=0.41).

**Conclusions**

In the largest study examining the quality of informed consent among intrapartum study participants from a low- and middle-income country, we found overall low levels of objective and subjective understanding of informed consent. Innovative approaches are needed to improve consent processes, particularly for study participants with lower levels of education and from poorer households.

## O43. Participant retention in mobile WACh NEO, a randomized controlled trial on the effect of interactive text messaging on neonatal mortality in Kenya

### Jennifer Unger^1^, Keshet Ronen^2^, Brenda Wandika^3^, Lusi Osborn^3^, Barbra Richardson^2^, Jenna Udren^2^, Olivia Schultes^2^, Esther Choo^2^, Peninah Kithao^3^, June Moraa^3^, Emmaculate Mukenyi Nzove^3^, Millicent Masinde^3^, Manasi Kumar^4^, Anna Hedstrom^2^, Dalton Wamalwa^5^, John Kinuthia^3^

#### ^1^Brown University; ^2^University of Washington; ^3^Kenyatta National Hospital; ^4^The Aga Khan University; ^5^University of Nairobi

*BMC Proceedings 2024*, **18(5):**O43

Submission ID #: IMNHC2124

**Background**

A significant number of neonatal deaths occur because of delays in recognition of illness and decisions to seek care. Studies of community-based interventions to decrease these delays face challenges related to the collection of data on neonatal outcomes, as well as retention of participants, which makes intervention effectiveness difficult to ascertain. Mobile WACh NEO is a recently completed randomized controlled trial in Kenya evaluating the effect of a two-way SMS messaging program between mothers and nurses on neonatal mortality. Our aim is to report the study communication strategies and resulting participant retention and yield of neonatal outcomes reported.

**Methods**

Pregnant women ≥14 years old and 28–36 weeks gestation were recruited from six clinics in Western Kenya and Nairobi. Participants in the intervention arm received automated SMS messages during pregnancy and through six weeks postpartum promoting newborn care and appropriate care-seeking. They could also send messages to the study nurse at any time. All participants were counseled to report delivery promptly via a phone call, “flash” to the study nurse, or SMS. To promote participant retention and reporting of outcomes, the study utilized SMS reminders for intervention participants, phone calls; study visits via phone or in person; or home tracing, as needed.

**Results**

Near the end of the study’s follow-up period (December 12, 2022): 5,013 participants had been randomized and remained eligible. Ninety-nine percent (4,984) of participants reported a delivery date—33% via SMS to the study nurse. Follow-up at six weeks was completed and the neonatal outcome reported for 99% (4,941/5,013) of participants. Forty-three percent of the follow-up visits were conducted over the phone. One percent of participants (72) were lost to follow-up. Among 4,951 live-born infants who have completed follow-up to date, there were 84 deaths for an interim neonatal mortality rate of 17.0 per 1,000 live births.

**Conclusions**

The use of multiple methods of interaction, including SMS and phone interviews, with study participants were associated with minimal loss to follow-up and very high rates of reported neonatal outcomes. These retention strategies can be used to bolster completeness of programmatic data on neonatal outcomes.

## O44. Exploring the influence of fistula-related stigma on post-repair antenatal care utilization

### Aishwarya Natarajan^1^, Hadija Haddy Nalubwama^2^, Justus Barageine^2^, Alison El Ayadi^1^

#### ^1^University of California, San Francisco; ^2^Makerere University College of Health Sciences

*BMC Proceedings 2024*, **18(5):**O44

Submission ID #: IMNHC2106

**Background**

Uganda has one of the highest rates of female genital fistula globally, with 1% of women reporting having experienced fistula-related symptoms in their lifetimes. Fistula repair availability has increased, prompting the need to attend to protecting health and well-being in the post-repair period, which may include safely achieving reproductive goals. Genital fistula symptoms can lead to lasting social, economic, and psychological consequences, including stigma, all of which are linked to domains of successful antenatal care (ANC) utilization, though post-repair pregnancy health behaviors have yet to be studied extensively. This study aims to investigate the relationship between consequences of fistula-related stigmatization and ANC across four key dimensions of stigma: enacted stigma, anticipated stigma, internalized stigma, and perceived community stigma.

**Methods**

Researchers collected demographics and ANC use information before conducting structured, in-depth interviews of Ugandan women (*n*=30) who underwent fistula repair in the prior 10 years in Mulago, Kitovu, and Kamuli hospitals and who had had subsequent pregnancies. Interviews were initially coded using a constructed codebook then evaluated for the dimensions of stigma experienced in the antenatal period for emergent themes. A conceptual framework was constructed based on observations to identify domains of ANC, study their interactions with experiences of stigma, and explore implications for ANC utilization.

**Results**

Narratives revealed that previous instances of enacted stigma in the post-repair period, such as verbal abuse, often preceded other forms of stigma, ultimately impacting social and health care domains of ANC. Multiple narratives linked enacted stigma experienced in health care settings to fear of disclosure of fistula history to both providers and partners. Fear of disclosure was a key manifestation of anticipated stigma across participant experiences. Other consequences included care delays due to referrals resulting from stigmatization by health care providers. Outside of stigmatization, narratives highlighted the impact of transportation access, financial barriers, and overcrowded facilities within ANC experiences.

**Conclusions**

These stories underscore the importance of respectful care within patient provider relationships within the continuum of pregnancy care. Key themes emerging from interactions between ANC domains and stigmatization suggest stigma reduction should be integrated into future maternal health interventions in Uganda.

## O45. Improving early and exclusive breastfeeding through engaging fathers in a humanitarian context in Nigeria

### Alessandro Iellamo, Solomon Atuman, Onesmus Langat, Halima Oji

#### FHI 360

*BMC Proceedings 2024*, **18(5):**O45

Submission ID #: IMNHC2096

**Background**

FHI 360 is working in northeast Nigeria to improve the survival and nutritional status of newborns and infants amid the ongoing conflict. Breastfeeding is a determining factor for the survival, growth, and development of infants in humanitarian contexts. A child who is exclusively breastfed is 14 times less likely to die in the first six months of life; however, cultural barriers and social factors influence recommended breastfeeding practices. The Father-to-Father Support Groups (FtFSGs) initiative was created in recognition of the critical role that men play in decision-making around infant feeding. During the meetings, members share experiences, support each other, and learn from the counseling cards, and the various demonstrations and roleplays.

**Methods**

FHI 360 conducted a quasi-experimental impact assessment to evaluate how FtFSGs influence breastfeeding. The method involved both intervention and comparison groups, and multistage sampling was used based on probability to selected settlements and households in Ngala, Banki, and Damasak, proportionate to population size.

**Results**

Most newborns (93.9%) in households of FtFSG members were reported to have been breastfed within one hour of birth, contrary to 59.2% of non-FtFSG members. Exclusive breastfeeding up to six months in households of FtFSG members was also reported in most cases (96.4%), compared to 57.1% of households without FtFSG members. One of the FtFSG members stated, “The knowledge I acquired during my participation in the support group has improved my life through practice by ensuring I provided food varieties to my family that meet their nutritional needs, ensure any child that is sick is presented to the health facility immediately, and support my wife to breastfeed our children by reducing her work load.”

**Conclusions**

The assessment confirmed the importance of males in breastfeeding support and other care practices, especially in humanitarian contexts. FtFSGs positively influenced early initiation and exclusive breastfeeding practices. The sustainability and scale-up of FtFSGs is critical to achieve recommended breastfeeding practices among vulnerable groups in emergencies.

## O46. Improving access to quality sexual and reproductive health for internally displaced women and girls and vulnerable host communities within the COVID-19 context in the Northwest and Southwest regions in Cameroon: late-breaker

### Lois Ann Enie, Alain Metuge, Agnes Obi, Margaret Dohsen

#### Reach Out NGO

*BMC Proceedings 2024*, **18(5):**O46

Submission ID #: IMNHC2093

**Background**

The six years of armed conflict in the Northwest and Southwest regions of Cameroon facilitated the displacement of skilled health workers. This contributed to decreased access to basic health care for pregnant women (PW). Many internally displaced persons and vulnerable populations lost their income sources and experienced exacerbated needs for sexual reproductive health and rights (SRHR) services including family planning (FP), antenatal care, and delivery services. Funded by GIZ Pro-Passar, Reach Out Cameroon implemented a humanitarian response project aimed to improve access to and uptake of quality SRHR services for women and girls in these conflict-affected regions.

**Methods**

The project was implemented in the Northwest and Southwest regions, from April 2022 to August 2022. Thirty SRHR volunteers (SRHRVs) were selected and trained to communicate the importance of SRHR and psychosocial health services. SRHRVs identified and referred PW for antenatal care, facility delivery, or FP, and provided service vouchers for the most vulnerable. A three-month stock of obstetric and FP supplies was donated to 30 health facilities, where 60 nurses were trained to provide skilled normal and assisted deliveries, effectively plot a partograph, and administer FP services. The project team conducted monthly supervision, data review, and validation missions.

**Results**

SRHRVs sensitized 63,4345 persons (25,978 males and 37,367 females) on the importance and availability of SRHR and psychosocial health services. A total of 184 vulnerable PW were given vouchers, with 319 PW referred. Two months before implementation, the 30 health facilities recorded only 121 of 234 (52%) deliveries were conducted by skilled personnel. After implementation, 518 of 544 (95.2%) deliveries were conducted by skilled personnel and 213 vulnerable PW received vouchers. Free modern FP services were provided to 574 women including 40 nursing mothers.

**Conclusions**

The total number of facility deliveries and those conducted by skilled personnel increased significantly. In conflict settings, barriers to quality SRHR services worsened. It is important that humanitarian responses integrate strategies that increase service demand, quality, and supply. Community-led-communication and cash voucher systems can break existing barriers for the most vulnerable and stimulate demand. Training of health facility staff to provide quality services ensures the population’s access to quality

## O47. Can oxytocin and tranexamic acid be mixed for co-administration by IV infusion for the treatment of postpartum haemorrhage?

### Pete Lambert^1^, Alessandra Tomazzini^2^, Philip Wright^1^, Claire McEvoy^1^, Ioannis Gallos^3^, Anne Ammerdorffer^2^, Lester Chinery^2^, Arri Coomarasamy^4^, Ahmet Metin Gülmezoglu^2^

#### ^1^Monash Institute of Pharmaceutical Sciences; ^2^Concept Foundation; ^3^World Health Organization; ^4^University of Birmingham

*BMC Proceedings 2024*, **18(5):**O47

Submission ID #: IMNHC2084

Note the full manuscript published in January 2023 in *BJOG*:

(https://obgyn.onlinelibrary.wiley.com/doi/10.1111/1471-0528.17398)

## P48. Effect of one prophylactic dose of azithromycin on bifidobacteria infantis colonization in infants from the mumta trial

### Aneela Pasha^1^, Ameer Muhammad^2^, Yasir Shafiq^1^, Waqasuddin Khan^1^, Syed Iqbal Azam^1^, Muhammad Imran Nisar^1^, Najeeha Talat Iqbal^1^, Fyezah Jehan^1^

#### ^1^The Aga Khan University; ^2^Vital Pakistan

*BMC Proceedings 2024*, **18(5):**P48

Submission ID #: IMNHC2071

**Background**

Azithromycin (AZ) is a macrolide antibiotic that is prescribed by the World Health Organization (WHO) for mass drug administration (MDA) in low- to middle-income countries (LMICs) for the treatment of infections. MDA-AZ has been shown to improve child survival and may have a role in improving infant growth outcomes. Bifidobacteria infantis is a key bacterium inhabiting the healthy infant gut. This study aimed to assess the impact of AZ on B. infantis colonization in the infant.

**Methods**

The Mumta study was a 3-arm (1:1:1) randomized controlled trial of lactating women and their infants (*n*=957 dyads). Interventions were lactation counseling (LC) only, LC and balanced energy protein (BEP) sachet for the mother, and LC, BEP with one dose of AZ to the infant at day 42 of birth. Fifty infants from each arm were randomly selected for biomarker and microbiome analysis at day 56 of birth. B. infantis colonization was assessed using quantitative PCR of infant fecal samples.

**Results**

AZ administration was associated with a 1.7-fold (95%CI 1.1-2.6) increase in colonization by B. infantis. The colonization with B. infantis was 1.7 times (95% CI 1.0- 2.8) in infants with a moderate increase in myeloperoxidase level. One dose of AZ had no effect on the enteropathogen count in infants at two months of age. Mode of delivery, maternal age, and anthropometric measures were not associated with B. infantis colonization.

**Conclusions**

To our knowledge, this is the first epidemiological study to report a possible association between AZ and B. infantis colonization. This study lends insight into AZ-associated microbiota perturbations.

## O49. Adaptation of the person-centered maternity care survey in the Dominican Republic: informing policy and practice to support respectful maternity care locally and globally

### Kate Mitchell Balla^1^, Ascanio Bencosme^2^, Eugene Declercq^3^, Monica Onyango^3^, Ruby Barnard-Mayers^3^, Eric Rubenstein^3^, Lucia Osirus^3^, Marletty Batista Solano^3^, German Cantillo-Mackenzie^3^, Nancy Scott^3^

#### ^1^Independent Consultant and Researcher; ^2^Hospital Presidente Estrella Ureña; ^3^Unknown

*BMC Proceedings 2024*, **18(5):**O49

Submission ID #: IMNHC2004

**Background**

The Dominican Republic (DR) has achieved nearly universal coverage of institutional childbirth, yet maternal/infant mortality remain high. The DR has the highest cesarean-section rate in the world and is one of only two countries with rising maternal mortality. However, experiences of childbirth in the DR have not been systematically examined. Global policy relating to respectful maternity care has largely been shaped by experiences from low-resource settings in Africa and Asia. This mixed-methods study presents a practical approach to contextual adaptation/application of the Person-Centered Maternity Care (PCMC) survey to measure respectfulness/responsiveness of care in a middle-income Caribbean country.

**Methods**

We adapted the PCMC survey to measure postpartum people’s and providers’ experiences at a large maternity hospital in Santiago, DR, during the third year of the pandemic and during a time of political instability in neighboring Haiti. We calculated mean PCMC scores, associations with sociodemographic factors, and concordance/discordance among postpartum people (Dominican and Haitian) and providers.

**Results**

Questions regarding contraception, maternal-newborn separation, differential treatment, and cesarean-section decision-making were added to improve the survey’s contextual fit. Those of Haitian origin, speaking Creole, being older, and living farther from the facility were associated with lower PCMC scores (*p*< .001). Gaps in perspectives of postpartum people and providers and disparities in experiences between Dominicans and Haitians were exposed. Nearly 70% of providers reported birthing people were spoken to in an understandable language compared to 29.8% of birthing people. Most providers (91.7%) reported consent being sought before procedures, compared to 58.1% of birthing people. Fewer Haitians, compared to Dominicans, reported friendly treatment (42% v 83%); consent before procedures (52% vs. 71%); and understandable language (14% v 63%). Sixty-one percent of Haitians and 44% of Dominicans reported postpartum maternal-newborn separation greater than six hours.

**Conclusions**

We present an approach to contextual adaptation of the PCMC instruments that can be replicated in various contexts for research and/or routine monitoring. As the first study to apply the PCMC methodology in the DR, we documented the magnitude of inequities in maternity care experiences and identified actionable opportunities to improve care in a context where maternal mortality is worsening.

## O50. Steps for operationalizing global guidance on nutrition interventions in antenatal care: lessons learned in four countries

### Tina Sanghvi

#### FHI Solutions

*BMC Proceedings 2024*, **18(5):**O50

Submission ID #: IMNHC1935

**Background**

A package of evidence-based scalable nutrition interventions is urgently needed in low- and middle-income countries. We aimed to document key steps and lessons learned in Bangladesh, Burkina Faso, Ethiopia, and India for adapting and integrating nutrition interventions into antenatal care (ANC) based on the World Health Organization’s 2016 guidelines.

**Methods**

We assessed the operational feasibility of implementing the nutrition package through ANC services using external randomized controlled evaluations during 2014–2022 and documented the adaptation process. The interventions included: iron and folic acid and calcium supplementation, counseling on dietary diversity and energy intake, weight gain monitoring and counseling, and counseling on preparation for breastfeeding. Country operational models were developed using data and feedback at each stage. Health systems strengthening, community mobilization, and social and behavior change approaches were used to improve the uptake of nutrition interventions. Key steps in adaptation were: 1) ensuring joint planning and commitments from national ANC policymakers and program managers; 2) identifying gaps in service delivery protocols and provider and women’s knowledge, beliefs, and practices; 3) co-designing field-tested content and materials for counseling, micronutrient supplies and distribution, and weight gain monitoring; 4) building capacity and sustaining the motivation of facility- and community-based health workers and managers; and 5) increasing awareness and support of community and household members for pregnant women’s nutrition.

**Results**

Evaluation results showed that the following improved significantly: coverage of nutrition counseling, weighing, and iron and folic acid adherence in four countries; ANC attendance in three of four countries; and dietary diversity and breastfeeding practices in two of the four countries. Challenges included lack of access to nutrient-rich foods, inadequate support for initiating breastfeeding immediately after delivery, and disruptions in ANC services during COVID-19.

**Conclusions**

Lessons learned: importance of streamlining nutrition activities and building on existing health and community structures; jointly using data with stakeholders to address gaps and adjusting interventions continuously; prioritizing micronutrient supplies and time given to counseling (focused on underlying behavioral barriers such as knowledge, self-efficacy, family support, and perceived social norms)., A contextualized nutrition package that addresses anemia, pre-eclampsia, excess and inadequate gestational weight gain, and breastfeeding can be integrated into ANC.

## O51. The challenge initiative’s business unusual approach to adolescent and youth sexual and reproductive health programming and reducing teenage pregnancies

### Njeri Nyamu, Kenneth Owino, Juliet Tumuhairwe, Paul Nyachae, Rose Mnzava, Peter Kagwe

#### Jhpiego

*BMC Proceedings 2024*, **18(5):**O51

Submission ID #: IMNHC1747

**Background**

Teenage pregnancy, or teenage childbearing, is when a girl aged 15–19 is pregnant with her first child or gives birth. Globally, an estimated 15% of young women give birth before the age of 18. In East Africa, teenage pregnancy rates surpass the global average to stand at 18% in Kenya and 25% in both Uganda and Tanzania. Pregnancy in the adolescent period is linked with higher occurrences of adverse maternal and perinatal outcomes. Limited access to adolescent- and youth-friendly sexual and reproductive health services (AYSRH) is a significant driver of teenage pregnancies. The Challenge Initiative (TCI) program supported sub-national governments in East Africa to implement AYSRH high-impact interventions in urban poor settings aimed at reducing too early, unintended pregnancies, delaying the first birth, and increasing spacing after the first birth.

**Methods**

TCI is a platform for impact and sustainable scale in reproductive health programming. The program works with sub-national governments to implement cost-effective AYSRH high-impact interventions, particularly in the urban and peri-urban poor settings. In East Africa (Kenya, Uganda, and Tanzania), TCI now supports 56 sub-national governments through coaching and mentorship on implementation of the high-impact interventions. Through smart advocacy and the process of joint program design, TCI ensures ownership and domestic resource allocation toward AYSRH program activities. for sustainability. Key activities conducted were: ensuring service delivery for youth and adolescents in the community; increasing youth-friendly facilities; providing adolescents with correct and accurate SRH information; and improving age-disaggregated adolescent and youth data visibility and use for programming effeciencies and effectiveness.

**Results**

TCI has successfully advocated with sub-national governments to prioritize AYSRH and make domestic resource commitments of a total of $1,406,383 (Kenya $544,510, Uganda $388,161, Tanzania $473,712) toward AYSRH high-impact interventions. Integrated youth-friendly services in 341 public health facilities. The major outcome of these efforts is the reduction in teen pregnancy rates in TCI-supported geographies by average of 23% (Kenya, 30%, Uganda, 12%, and Tanzania 24%).

**Conclusions**

Governments are capable of committing domestic resources and implementing, impactful AYSRH programs to address the urgent need to combat rising teenage pregnancy rates.

## O52. Impact of midwifery regulatory environments on maternal health outcomes in Low- and Middle-Income Countries (LMICs)

### Emma Clark

#### Vanderbilt University

*BMC Proceedings 2024*, **18(5):**O52

Submission ID #: IMNHC1711

**Background**

We created a measure of midwifery policy and regulatory environments, then correlated a country’s score with access to midwives and maternal health outcomes. Because evidence increasingly shows that access alone is insufficient to transform outcomes, we incorporated quality of care outcomes such as patient experience to measure the impact of the policy and regulatory environment on the care midwives are able to provide. We hypothesized that countries with stronger policy and regulatory environments would have higher rates of access to midwives and improved perinatal outcomes.

**Methods**

To examine the impact of policy and regulatory environments on access to midwives, we conducted secondary data analysis to correlate the scores calculated from State of the World’s Midwifery data with midwifery workforce density data and rates of facility birth in a country. To examine the impact of policy and regulatory environments on perinatal outcomes and maternal and newborn mortality, we used secondary data analysis to determine the association of country scores with perinatal outcomes after adjusting for country income level and per capita spending. We also conducted hierarchical linear regression modelling to control for country income level and per capita spending on health in each country when examining the relationship of MISS scores with outcomes.

**Results**

Final results currently in progress.

**Conclusions**

Maternal health outcomes appear to be correlated with policy and regulatory environments in LMICs.

## P53. A situation analysis of care of small babies in the community in Indonesia - preliminary survey for the introduction of the Little Baby Handbook -

### Ni Made Diah Permata Laksmi D^1^, Eny Yantri^2^, Ricvan Dana Nindrea^2^, Yoko Masaki^3^, Keiko Osaki^3^, Tomoko Hattori^3^

#### ^1^Ministry of Health, Indonesia; ^2^Andalas University; ^3^Japan International Cooperation Agency

*BMC Proceedings 2024*, **18(5):**P53

Submission ID #: IMNHC1709

**Background**

In Indonesia, half of the under-five deaths are neonatal deaths, with low birthweight and preterm babies being the leading cause. As part of the effort to address this issue, focusing on optimizing the care of small babies in the community, the Ministry of Health (MOH) developed the “Little Baby Handbook (LBH)” in 2021, which contains health records and information on the care of small babies. The MOH plans to implement the LBH on a trial basis in FY2022, with a view to nationwide rollout in FY2023. Prior to the pilot implementation, a survey was conducted to assess the current status of health services and the knowledge and behavior of health workers and mothers.

**Methods**

A cross-sectional survey was conducted in May 2022 in all eight pilot health facilities, six in Central Java, and two in West Sumatra. A self-administered questionnaire was used for 51 health workers. A structured questionnaire was used to interview 211 mothers of small babies.

**Results**

Health workers’ knowledge of small baby care has room for improvement, with an average score of 69%. Only 59% of health workers always provide health education, and only 38% were confident in caring for small babies. Mothers’ knowledge was generally low, with a mean score of 62%. Fifty-five percent of the mothers had the possibility of depression (EPDS ≥ 10).

**Conclusions**

Mothers with small babies are often anxious about raising their children, worried, and lose confidence. Furthermore, since the community’s understanding of small babies is still poor, the mothers tend to become isolated without support. As a result, their babies cannot receive optimal care for their growth and development. This survey revealed that mothers are sent home without sufficient health education. This is partly due to the lack of knowledge and confidence of health workers in caring for small babies. The use of the LBH aims to improve the quality of health services for small babies, promote education and emotional support for mothers and families, as well as raise awareness in the community to optimize the growth and development of small babies, thereby contributing to the reduction of infant mortality and health equity.

## O54. Introduction of an Evidence-Based Program of Early Essential Newborn Care (EENC) in Sudan: how can EENC reduce neonatal morbidity and mortality?

### Esmehan El Kheir, Almuhalb Gaffer

#### Federal Ministry of Health, Sudan

*BMC Proceedings 2024*, **18(5):**O54

Submission ID #: IMNHC1700

**Background**

In Sudan, the newborn mortality rate is estimated to be 33/1,000 live births and has declined at a slower rate than older child mortality over the last 10 years. Newborn deaths currently represent 49% (statistical report, World Health Organization [WHO] 2012) of under-five child deaths and 64% of all infant deaths, highlighting that the newborn period remains the period of highest risk for all infants. Sepsis and birth asphyxia are the most important immediate causes of infant mortality, with prematurity and low birthweight (LBW) contributing factors to a high fraction of newborn deaths, 32% of all babies are estimated to be LBW. Skilled birth attendants attend an estimated 78% of all births, although only 28% of these births take place in a health facility.

In this paper, we explored the effort made by Sudan’s Ministry of Health to strengthen newborn care by adopting EENC, a package of evidence-based interventions delivered during delivery and in the immediate newborn period using a WHO coaching approach.

**Methods**

The study employed a structured assessment tool of the 10 EENC heath facilities. Two groups of two from the National Child Health Program were conducted and 10 visits to the 10 health facilities were conducted over a period of four weeks. Ten in-depth interviews with key informants from the health facilities were conducted. Thematic data analysis was used.

**Results**

Overall clinical practices around the birth were satisfactory including direct skin-to-skin contact. There was a reduction in the admission rate to the neonatal intensive care unit in the targeted health facilities.

**Conclusions**

EENC needs to be scaled up toward total coverage of the health facilities that provide delivery services.

## O55. Caught in the middle: understanding the experience of Maternal and Newborn Health (MNH) providers through the lens of organizational theory for mistreatment in childbirth

### Kate Ramsey

#### Scope Impact

*BMC Proceedings 2024*, **18(5):**O55

Submission ID #: IMNHC1698

**Background**

Reports of mistreatment in childbirth have emerged around the globe, reducing trust in health systems at a profound moment of human life. The similar patterns and dynamics indicate its systemic nature. Limited research to date has focused on developing explanatory theory. Organizational sociology provides one lens for theory development, unearthing the experience of providers within the broader system that leads to its persistence.

**Methods**

To develop novel theory, analogical comparison was used comparing mistreatment in childbirth in Tanzania with the shuttle explosion at the U.S. National Aeronautics and Space Agency, from which Diane Vaughan elaborated a theory on the normalization of organizational deviance. For the Tanzania case, a systematic review on maternal health and the health system in Tanzania was conducted. Data were analyzed through a multi-level framework incorporating concepts from Vaughan’s theory and literature on mistreatment. Comparative analysis across system levels and concepts elicited relationships and patterns. These were then compared to Vaughan’s theory, and other theories were explored to explain differences.

**Results**

The experience of providers, who are caught in the middle between an unsupportive system and discontented citizens, emerged as a fundamental factor in the persistence of mistreatment. National goals remained ambitious despite scarcities. Providers’ capacity was thus stretched thin due to insufficient staffing and skills. Insufficient physical resources and psychological support was further exacerbated by expectations to meet unrealistic targets. Providers then faced moral dilemmas when they could not provide the expected standard of care. Citizen’s expectations were also not met, and frontline providers took the blame for system failures. Providers appear to have shifted values or withdrawn to cope with distress at their powerlessness to perform according to standards. As a result, they rationed emotion work, resulting in mistreatment, which was experienced as disrespect by women.

**Conclusions**

Analogical comparison with another case of organizational deviance based on literature enabled a novel approach to elaborate theory. The emergent theory demonstrates that systems will need to not only center women’s needs, but also the well-being and support of providers to ensure that childbirth in health facilities is a positive and respectful experience.

## O56. Small babies and symmetrical growth: comparing two measures of fetal growth restriction on the prevalence and neonatal mortality risk in a cohort of 11,000 Small for Gestational Age (SGA) babies from rural Nepal

### Michael Diaz, Elizabeth A. Hazel, Daniel Erchick, Joanne Katz, Robert Black

#### Johns Hopkins Bloomberg School of Public Health

*BMC Proceedings 2024*, **18(5):**O56

Submission ID #: IMNHC1687

**Background**

SGA newborns have been sub-categorized into asymmetric and symmetric SGA (aSGA/sSGA) to differentiate the timing of fetal growth restriction. aSGA is thought to be due to late fetal growth restriction and has been shown to be protective against neonatal mortality compared to sSGA births. However, there are no standard definitions, systematic reviews to describe the methodologies, or cross comparisons in a large cohort of SGA births.

**Methods**

We conducted a systematic literature review of papers reporting aSGA/sSGA at birth to identify definitions of aSGA/sSGA, and we applied these definitions to estimate the prevalence and neonatal mortality risk for aSGA/sSGA among more than 11,000 SGA births from rural Nepal.

**Results**

We identified 779 papers, 81 met the criteria for full review, and 54 met the full inclusion criteria. We identified two major methods defining aSGA/sSGA: 1) Ponderal index (PI), which divides birth weight by height cubed, accounted for about 65% of the papers with a range of cutoff values from 2.25 to 2.49 g/cm3 x 100; and 2) Head circumference (HC) percentile, accounting for about 25% of papers, using the 10th percentile cutoff. Additional approaches accounted for the remaining 10%. Applying the two main definitions to a dataset of SGA births in Nepal resulted in substantial differences in the estimated prevalences of aSGA/sSGA and associated neonatal mortality risk. Using PI and the range of cutoff values, prevalence of aSGA ranged from 26.6% to 67.6%. In contrast, using the 10th percentile for HC, 41.3% of SGA births were aSGA. The HC definition found sSGA births had a 49% increased risk of neonatal mortality compared to aSGA (Risk Ratio (RR): 1.49 95% Confidence Interval (CI): 1.11-2.00). In contrast, the PI showed a protective to null effect across the range of cutoff values for sSGA with an RR of 0.73 (95% CI: 0.56-0.59) at the lowest and 0.91 (95% CI: 0.68-1.21) at the highest cutoff.

**Conclusions**

Substantial differences in estimates of both aSGA/sSGA prevalence and neonatal mortality risk suggest the historic methods to define these conditions are not interchangeable and more careful validation work is required to create an evidence-based approach to differentiate the range of fetal growth restrictions in SGA births.

## O57. Organisational and individual readiness for change to respectful maternity care practice and associated factors in Ibadan, Nigeria: a survey

### Oluwaseun Esan^1^, Salome Maswime^2^, Duane Blaauw^3^

#### ^1^Obafemi Awolowo University; ^2^University of Cape Town, Western Cape; ^3^University of the Witwatersrand

*BMC Proceedings 2024*, **18(5):**O57

Submission ID #: IMNHC1667

Note the full manuscript published in November 2022 in *BMJ Open*:

(https://bmjopen.bmj.com/content/12/11/e065517)

## O58. Increasing men’s support for and engagement in pregnancy and early childhood: adapting an evidence-based intervention from Rwanda to Ethiopia

### Elizabeth Stones, Heran Abebe, Shailee Ghelani

#### EnCompass LLC

*BMC Proceedings 2024*, **18(5):**O58

Submission ID #: IMNHC1664

**Background**

Ethiopia has experienced significant improvements in maternal and newborn health; however, maternal and infant mortality remain high. To address the gaps in men’s support and engagement in pregnancy and early childhood, the Transform: Primary Health Care Activity conducted formative research and a collaborative stakeholder consultation process to adapt Program P to the Ethiopian context.

**Methods**

The study took place in eight kebeles across Oromia and Southern Nations, Nationalities, and People’s Region from August 2019 to February 2020. Data collectors conducted key informant interviews with eight male leaders and eight health workers as well as focus group discussions and attitudes and behavior surveys with 96 couples. The team analyzed the data using Dedoose 7.0.23 and STATA 15 and triangulated the data during a participatory data analysis and interpretation workshop to develop findings and conclusions.

**Results**

Although the majority of respondents reported that couples make decisions together about the number of children and spacing, most respondents agree that men make the final decision on the number of children to have. Among the sampled population, 60% of women reported their partner accompanying them for at least one antenatal care visit. When asked if they took their youngest child to their vaccination appointment, 91% of women reported they took the child themselves and 6% reported taking the child together. Respondents reported that men’s role in income-generating activities constrained time available for antenatal care and child health visits, whereas caregiving and family tasks are considered the role of women. Several respondents also noted traditional gender roles and norms cause negative attitudes or criticism of male engagement.

**Conclusions**

The final curriculum underwent multiple rounds of revisions through a collaborative adaptation process involving a diverse array of stakeholders. The team made key modifications to enhance adherence to the contextual specificities highlighted by the findings, including emphasizing participant-centered approaches, including more sessions for couples, and adjusting men-only sessions to focus on power, violence, and decision-making. Ethiopia’s Ministry of Health plans to implement, evaluate, and scale the intervention in the coming years. This research serves as a collaborative, participatory, and gender-transformative model for adapting evidence based interventions.

## O59. Development of a community-centred model for reclaiming community reproductive, maternal, and neonatal health in the context of the COVID-19 era in Kenya

### Miriam Carole Atieno Wagoro

#### University of Nairobi

*BMC Proceedings 2024*, **18(5):**O59

Submission ID #: IMNHC1658

**Background**

Access to reproductive, maternal, and neonatal health (RMNH) services is critical to the achievement of Sustainable Development Goal (SDG) 3: good health and well-being and related maternal SDGs such as SDG 1 and 2, which seek to eliminate poverty and hunger, respectively. The outbreak of COVID-19 presented a double tragedy to the lives of women, neonates, and children in Kenya. Transmission prevention measures imposed by Kenya’s Ministry of Health, coupled with fear of contracting COVID-19 and social stigma, disrupted access to basic RMNH services, thereby endangering the lives of pregnant women, mothers in labor, and newborns and threatening the gains made toward SDGs 3.1 and 3.2 on prevention of maternal and newborn mortality, respectively. A customized community model to facilitate access to RMNH services was required not only to reclaim the gains made, but to achieve SDG goals 2.1 and 3.2.

**Methods**

Main objective: To develop a community-centered model for enhancing access to basic RMNH services at the primary health care level in Kenya. The methods used were part of an ongoing mixed-method implementation research in Kitui and Kilifi counties. Nominal group technique was used with 26 groups comprising of 200 community health volunteers, 40 County Health Management Teams, and 20 community health extension workers. Data were collected for two weeks using researcher-administered standardized questions that guided the discussion. Content analysis was used to analyze qualitative data. For quantitative data, descriptive statistics and percentage agreement set at 70% determined the priority categories subsequently used to develop a customized community RMNH model by consensus. The study was conducted within approved dictates of Kenyatta National Hospital-University of Nairobi Ethics and research committee, as well as respective county administrations.

**Results**

A community-centered RMNH model was developed by consensus. Model components included: community engagement strategies that involves understanding the contexts of practice and cultural influences; and community champions who understand team dynamics, universal teams, harmonious intersectoral interactions, and outputs.

**Conclusions**

The community-centered RMNH model is customized to the Kenyan situation and is relevant to community health workers and volunteers as well other primary health care workers.

Recommendation: The community RMNH model that is responsive to the community needs during and post COVID 19 can be extended to other counties in Kenya. There is need to test the model in other counties to determine its effectiveness and adoption nationally and across the region of Africa.

## P60. Understanding household-level sociodemographic and maternal health access factors for zero dose children and their mothers in 82 low- and middle-income countries

### Brooke Farrenkopf, Chizoba Wonodi

#### Johns Hopkins Bloomberg School of Public Health

*BMC Proceedings 2024*, **18(5):**P60

Submission ID #: IMNHC1652

**Background**

In 2021, an estimated 18 million children did not receive any routine immunizations and constitute the population known as zero-dose children. There is growing momentum and investment in reaching zero-dose children and addressing the gross inequity in the reach of health services. To effectively address this inequity, there is an urgent need to characterize the zero-dose population, their mothers, and the barriers they face in accessing care.

**Methods**

Data from Demographic and Health Survey and Mulitple Indicator Cluster Surveys from 2011 to 2020 in low-, lower-, middle-, and upper-middle-income countries were utilized. Zero-dose status was defined as children 12–23 months who had not received any doses of BCG, DTP, polio and measles-containing vaccines. We characterized the zero-dose population by sociodemographic and health access factors. Multivariate logistic regressions were used to determine household-level factors associated with zero-dose immunization status.

**Results**

We included 82 countries in our univariate analyses and 68 countries in our multivariate model. Overall, 7.5% of the population were zero-dose children, with half (51.9%) living in African countries. Zero-dose children were predominantly born to mothers who completed fewer than four antenatal care (ANC) visits (60.1%) and had home births (58.5%). Yet, surprisingly, a substantial proportion of zero-dose children’s mothers received appropriate care during pregnancy (33.5% have mothers with at least four ANC visits). Controlling for other factors, children had three times the odds (OR=3.00, *p*< 0.05) of being zero-dose if their mother had not received tetanus injections, 2.46 times the odds (*p*< 0.05) if their mother had not received ANC visits, and nearly twice the odds (OR=1.87, *p*< 0.05) if their mother had a home delivery, compared to children of mothers who received the recommended care.

**Conclusions**

Poor access to maternal health care was a strong risk factor of zero-dose status and highlights important opportunities to improve the quality and integration of maternal and child health programs. Additionally, because a substantial proportion of the mothers of zero-dose children do receive appropriate care, this highlights an immediate opportunity to bolster the continuum of services received by the mother-child dyad.

## O61. Leveraging private sector partnerships to sustainably connect high-risk mothers to emergency transport and appropriate-level quality care in Kakamega County, Kenya

### Javan Waita, Victor Bwire

#### Jacaranda Health

*BMC Proceedings 2024*, **18(5):**O61

Submission ID #: IMNHC1640

**Background**

Delays in timely access to care drive a third of all maternal deaths in Kenya. Hospitals and health system managers lack means to rapidly identify at-risk mothers and service gaps, and gather clinical information on incoming patients resulting in mothers being “bounced-around” between facilities to find the appropriate level of care. Kakamega County has partnered with Jacaranda and Rescue.co to improve maternal survival by expediting access to emergency care.

**Methods**

A 2019 feasibility assessment identified bottlenecks limiting safe and timely referral as lack of: i) coordinated data to direct ambulances to the right place; ii) basic emergency equipment and trained personnel; and iii) sustainable financing. This finding prompted Jacaranda and Rescue.co to connect their two systems (PROMPTS, which uses AI technology to prioritize urgent cases to hospitals, and Rescue.co’s ambulance dispatch service, which uses Flare technology to link emergency cases to transport) to improve speed and accuracy of the referral pathway.

**Results**

**Faster referral, at scale**

In 10 months, 1,371 women were connected to care with a 90% decrease in response time and, despite the often-critical nature of the transports, the survival rate of newborns transferred was high (+/-90%).

**Visibility into heath systemic gaps**

Targeted investment in facility infrastructure and training: dispatch data inform priority equipment during emergencies, and gaps at referral facilities with 40% of cases needing an operating theatre, and 33% a neonatal intensive care unit and ultrasound to diagnose 30% of cases.

Better management of vital commodities: dispatch data pointed to need of blood in 54% of referral cases, babies need oxygen in 78% of cases, and ~40% of transferred mothers required oxytocin.

**Conclusions**

Improving quality of maternal care requires contextual understanding of the how, where, and why of its access; otherwise, avoidable maternal deaths will continue to stagnate. Maternal referral is a bellwether for wider health systems, given that it requires strong coordination, engagement across multiple health system levels, and coordinated data for decision-making to deliver well. This partnership demonstrates the ability to accurately map a mother’s journey through “passive” data that, when collected and analyzed in quasi real time, acts as a way of measuring the vital signs of health.

## O62. Evaluating readiness for human milk banking and use of donor human milk for achieving exclusive human milk diets for low birthweight infants

### Kimberly Mansen^1^, Kiersten Israel-Ballard^1^, Karim Manji^2^, Christopher Sudfeld^3^, Tisungane Mvalo^4^, Melda Phiri^4^, Roopa Ballad^5^, Sunil Vernekar^6^, Leena Das^7^, Sanghamitra Panda^8^, Linda Vesel^9^, Katherine Semrau^9^

#### ^1^PATH; ^2^Muhimbili University of Health and Allied Sciences; ^3^Harvard T.H. Chan School of Public Health; ^4^Univeristy of North Carolina, Chapel Hill; ^5^KAHER’s J N Medical College; ^6^KLE University; ^7^SCB Medical College; ^8^City Hospital, Bhubaneshwar; ^9^Ariadne Labs

*BMC Proceedings 2024*, **18(5):**O62

Submission ID #: IMNHC1636

**Background**

Optimizing feeding of small and sick newborns (SSN), those born low birth weight (LBW) or preterm, is a critical component to improve quality of care. Donor human milk (DHM) from a human milk bank (HMB) is recommended by the World Health Organization when a mother’s own milk is unavailable, yet evidence and implementation strategies for low- and middle-income country (LMIC) settings are lacking. The Low Birthweight Infant Feeding Exploration (LIFE) Study aimed to understand feeding of LBW infants in LMIC settings. We conducted DHM readiness assessments at hospitals in India, Malawi, and Tanzania to determine implementation readiness and feasibility of HMB integration and/or use of DHM for LBW infants as a systems’ level infant and young child feeding intervention.

**Methods**

Data were collected at four hospitals in India, and two hospitals in both Malawi and Tanzania, through in-person interviews, virtual group discussions, in-facility observations, and “flow of the milk” process mapping. An audit was performed at one site with a pre-existing HMB (India). This built upon the broader LIFE study including in-facility observations at key research sites and studying feeding trends of an observational cohort of LBW mother-infant dyads.

**Results**

Data suggested the prioritization of breastfeeding and use of human milk as the primary diet for LBW neonates at all study locations. Little, if any, expressed breast milk storage is available in the eight facilities and excess milk is discarded at all but one hospital site, which had an existing HMB. Active collection of donor milk is not solicited and lactation support is not optimal. Training for staff on skilled lactation support for SSN needs improvement. All sites identified a need for DHM but lacked adequate stakeholder awareness, resources, and planning for safe and quality HMB systems for sustainable implementation.

**Conclusions**

All study sites desired DHM as an alternative to formula for SSN. Strengthened policies, provision of skilled lactation support, identification of milk donors, and access to devices and tools to support maternal lactation and milk expression, and human milk feeding are all yet to be optimized. Operational models, implementation science, and cost-effectiveness studies are warranted to introduce HMBs in these settings.

## O63. Experience conducting verbal autopsy via phone for neonatal deaths and stillbirths in the mobile WACh NEO randomized controlled trial in Kenya

### Jenna Udren^1^, Brenda Wandika^2^, Keshet Ronen^3^, Peninah Kithao^2^, June Moraa^2^, Georgina Mugodo^2^, Winfred Mwende^2^, Janet Adhiambo Okota^2^, Lusi Osborn^2^, Emmaculate Mukenyi Nzove^2^, Millicent Masinde^2^, Barbra Richardson1, Jennifer Unger^3^, John Kinuthia^2^, Anna Hedstrom^1^

#### ^1^University of Washington; ^2^Kenyatta National Hospital; ^3^Brown University

*BMC Proceedings 2024*, **18(5):**O63

Submission ID #: IMNHC1634

**Background**

Mobile WACh NEO is an ongoing randomized controlled trial to determine the effect of informative and interactive SMS between peripartum participants and a nurse on neonatal mortality in Kenya. The study includes multiple methodologies to gather data on neonatal death or stillbirth, including participant interviews, medical record abstraction and verbal autopsy (VA). VA is typically administered face to face at the participant’s home to determine cause of death (COD) and has not been validated with other forms of administration. During the early COVID-19 pandemic, however, in-person study visits were restricted, challenging timely data collection. We hypothesized that VA could be successfully administered over the phone.

**Methods**

We utilized the Population Health Metrics Research Consortium Shortened Verbal Autopsy Questionnaire and analyzed the results using the SmartVA-Analyze Application. Staff aimed to conduct VA 6–18 weeks after a stillbirth or neonatal death. Completion via phone vs. in-person visit was determined by participant availability.

**Results**

Among the 5,020 study participants as of August 2022, there were 64 stillbirths and 90 infant deaths. VA was completed among 90.5% of those eligible—85.6% among neonatal deaths and 90.6% among stillbirths. A total of 54.5% of VAs were conducted by phone and the rest at the clinic. Median time from the event to VA was 46 days (IQR 42, 52)—47 days via phone and 43 days at the clinic. The SmartVA-Analyze tool determined a COD for 95.5% of all events. The COD was not determined in 4.1% of phone VAs and 4.9% of clinic VAs.

**Conclusions**

We demonstrated that VA can be conducted via phone to supplement data collection in trials evaluating peripartum deaths. Although our findings suggest phone collection is consistent with in-person administration, further evaluation of the validity and reliability of this remote data collection is needed. The sensitive nature of the subject matter suggests we should also explore acceptability for interviewers and participants. The urgency of timely reporting of COD for both research and civil registration purposes supports the adaptation of more modern, flexible methods to VA administration.

## O64. Data-driven decision-making: enabling district health management to strengthen maternal, newborn, and child health services

### Bilal Iqbal^1^, Girum Taye^2^, Mehret Dubale^1^, Joanna Schellenberg^1^

#### ^1^London School of Hygiene and Tropical Medicine; ^2^Ethiopian Public Health Institute

*BMC Proceedings 2024*, **18(5):**O64

Submission ID #: IMNHC1628

**Background**

Use of local data for health system planning and decision-making in maternal, newborn, and child health (MNCH) services is limited in lower- and middle-income countries, despite advances in data-gathering. Among health stakeholders, there is an underdeveloped culture of data-sharing and collaborative planning. Yet, while service delivery processes are a trend of health system research, scant attention is paid to health system-management research—despite this being the key to effective MNCH services.

The Data-Informed Platform for Health (DIPH) is a health system-strengthening intervention that promotes structured decision-making by health administrators and managers using local data. DIPH entails quality decision-making: a) defining problems using a health system framework; b) reviewing data; c) considering alternative options; d) ensuring value-based prioritization; and e) implementing a consultative process to develop, commit to, and follow up on action plans. Central to DIPH is a package of job aids and guidelines, providing tools and knowledge for structured decision-making using available data. DIPH’s strategy was conceptualized, developed, and tested over 10 years.

**Methods**

DIPH was implemented in 12 (out of 24) randomly selected woredas in Ethiopia’s North Shoa zone between October 2020 and June 2022. A cluster-randomized controlled trial was conducted with a difference-in-differences analysis of health system outcomes to compare DIPH and non-DIPH arms.

**Results**

A total of 58 four-monthly DIPH cycles were conducted, each focusing on a specific MNCH health service delivery challenge at the district level. There were significant improvements in district health administrators’ competence in, and practice of, data-driven decision-making to manage services in the DIPH arm. For example, the likelihood of regular reviews of monthly MNCH performance increased by 34% (*p*-value< 0.001), and that of data-based feedback to health facilities increased by 45% (*p*-value< 0.001). Significant improvement was found in district health administrators’ analysis and use of information for MNCH services.

**Conclusions**

DIPH strengthened district health management and administration. By improving data management and appraisal practices, systemizing problem analysis to follow up on action points, and creating a positive stakeholder engagement culture, DIPH improved MNCH services.

## O65. Women’s experience of birth companions and respectful maternity care in Malawi

### Brady Zieman^1^, Charlotte Warren^1^, Abigail Kazembe^2^, Martha Kamanga^2^

#### ^1^Population Council; ^2^University of Malawi Kamuzu College of Nursing

*BMC Proceedings 2024*, **18(5):**O65

Submission ID #: IMNHC1620

**Background**

In Malawi, a family member or friend escorts women to a health facility for childbirth. These “guardians” or companions generally provide food, wash clothes, and support the woman during labor/childbirth. However, systematic use of birth companions (BC) attending women throughout labor and delivery is fragmented and not routine- even though it is Ministry of Health policy. We examine women’s experience including respectful maternity care (RMC) following the introduction of job aids/tools to formalize BCs.

**Methods**

As part of a broader research portfolio on advancing postpartum hemorrhage care, quantitative surveys with postnatal women (473 at baseline and 530 at endline) from 18 health facilities (11 intervention and 7 comparison sites) in Malawi were conducted. These included women’s experiences of a BC and RMC before and after the intervention. Questions from the person-centered maternity care (PCMC) scale were used to assess presence of BCs and women’s experiential quality of care. Data were analyzed using Stata 14.

**Results**

Approximately 37% of all women reported usually or always having a companion present during labor and delivery. At intervention facilities, the proportion of women with companions increased significantly between data collection rounds (27% vs. 47%, *p*< 0.001); the between-round decrease observed in comparison facilities (41% vs 30%, *p*=0.075) was not significant. Difference-in-difference method indicates a significant effect of the intervention (OR: 5.96, *p*< 0.001). Significantly more women reported mostly/always being treated with respect (78% vs. 61%; *p*< 0.001), felt involved in decisions (40% vs. 20%; *p*=0.000), were asked for consent for procedures (45% vs. 36%; *p*=0.007), and the medicines were explained (40% vs 22%; *p*< 0.001) if a BC was present. Women were significantly less likely to report extreme pain (31% vs 41%; *p*=0.001) if a BC was present. Rural and community hospitals had significantly reduced odds of allowing BCs compared to primary health care facilities.

**Conclusions**

The presence of a BC during labor and delivery significantly increased women’s perceptions of quality of care—as measured through the PCMC scale. Where companions are permitted, their presence improves communication between women and providers, resulting in a better experience of care. Empowering providers with basic roles and communication tools to help work.

## O66. Improving access to Maternal, Newborn, and Child Health (MNCH) medical products in low- and middle-income countries: a mapping of registration of mnch medical products in nine countries

### Jane Briggs^1^, Lauren Herzog^2^, Kate Kikule^1^

#### ^1^Management Sciences for Health; ^2^USAID Medicines, Technologies, and Pharmaceutical Services (MTaPS) Program

*BMC Proceedings 2024*, **18(5):**O66

Submission ID #: IMNHC1613

**Background**

Regulatory challenges in resource-limited countries can compromise the availability of quality lifesaving MNCH medical products. Quality-assured products of generic medicines may not be registered or their registration status expires without renewal, leaving a vacuum that may be filled by products that are substandard or falsified.

**Methods**

The U.S. Agency for International Development’s Medicines, Technologies, and Pharmaceutical Services (MTaPS) Program conducted a nine-country study in 2020 to better understand the challenges to registration of MNCH medical products in low- and middle-income countries. To identify barriers, the registration status of 18 MNCH tracer medicines was assessed by reviewing policies, regulations, and other documents and interviewing the countries’ regulators and 11 pharmaceutical manufacturers.

**Results**

Most tracer medicines were registered in most of the nine countries, but the percentage of medicines with at least one registered product ranged from 28%–100%. Some countries only had one or two registered products for some MNCH tracer medicines. Five countries had not registered any of the four World Health Organization-prequalified products. The average registration timeline ranged from six months to four years due to national regulatory authority backlogs, complicated procedures, and insufficient numbers of competent staff. Few countries officially recognized or relied on regulatory decisions from other countries. No country had formal processes embedded in legislative provisions to prioritize and expedite registration of essential MNCH medical products.

**Conclusions**

Solutions to registration impediments involve legal, organizational, and procedural changes. MNCH medicines have specific challenges such as low profit margins due to the ease of production and variety of manufacturers, so an inconvenient registration process is a disincentive for manufacturers. Likewise, buyers who are more price than quality conscious may not procure costlier prequalified medicines.

Catalysts for registration include streamlining processes with electronic management information systems that provide market information, maintaining qualified staffing, and emphasizing reliance on and recognition of other regulatory authority decisions.

In response to the findings of the study, MTaPS is working to strengthen regional collaboration to streamline registration and prioritize the registration of MNCH medicines and supporting countries to build capacity of assessors and render registration procedures more efficient.

## O67. Algorithms to predict newborn complications in the first 28 days of life in Eastern Uganda (N-COP Study)

### Akuze Joseph Waiswa^1^, Paul Mubiri^2^, Frank Namugera^3^, Geraldine Agiraembamazi^2^, Peter Waiswa^3^, Nicole Santos^4^, Harriet Nambuya^5^

#### ^1^London School of Hygiene & Tropical Medicine; ^2^Makerere University School of Public Health; ^3^Makerere University; ^4^University of California, San Francisco; ^5^Jinja Regional Referral Hospital

*BMC Proceedings 2024*, **18(5):**O67

Submission ID #: IMNHC1610

**Background**

Complications following preterm birth cause morbidity and mortality. Globally, newborn complications account for approximately 28% of neonatal deaths. Preemies are between six and 26 times more likely to die during the neonatal period than term newborns. Mathematical and statistical algorithms can be used to predict the risk of development of complications and adverse outcomes among preemies and to drive proactive measures to anticipate, prevent, prepare management, and improve survival in the short and long run. We developed algorithms to predict newborn complications and estimate outcomes within the first 28 days of life.

**Methods**

Neonates were followed up for 28 days at Jinja regional referral and Iganga general hospitals using a prospective cohort study design. Mothers who consented after delivery and their neonates (terms and preterms) enrolled between January–August 2019. Prematurity was assessed using the last normal menstrual period and/or obstetric ultrasound in the first trimester if available. The gestational age was confirmed using the neuromuscular maturity score (Ballard score). Algorithms to predict newborn complications among preemies were developed using negative binomial and machine learning and data-mining techniques (Logistic Regression, Naïve Bayes and Decision trees).

**Results**

The incidence rate of complications was 46% (overall), 57% (preterms), and 34% (term) for infants. Children with chest-in drawing were 30 times more likely to develop a complication at birth than their counterparts without chest-in drawing (*P*< 0.001). Having an increased hemoglobin level at birth, or higher Ballard score, or a higher APGAR score at 1 minute was protective against complications at birth. Comparing the testing error (e=0. 1969) for the Logistic model fitted over the same training data with other fitted models, the Logistic model incorrectly classifies approximately 3, 3, 24, and 27 observations from the test data compared to the Naïve Bayes, SVM, Decision trees, and Random Forest algorithms.

**Conclusions**

The performance of all algothrims was consistently good for predicting any newborn complications; however, the performance of all algorithms that predict the number of newborn complications at birth correctly is 50:50. Further research about predicting newborn complications is need.

## O68. Leveraging digital health coaching to improve exercise regimens in pregnant women

### Esupat Lotasarwaki, Bih-Neh Estella, Divine Akpan, Maria Moosa, Nneka Mobisson

#### mDoc Healthcare Ltd

*BMC Proceedings 2024*, **18(5):**O68

Submission ID #: IMNHC1599

**Background**

Exercise during pregnancy can prevent and limit adverse maternal and fetal morbidities and provide long-term benefits through reduction of maternal weight gain during pregnancy, and improvement in cardiovascular fitness. Regular exercise during pregnancy has shown benefits to the mother and fetus in several ways including: reducing back pain, easing constipation, and decreasing the risk of gestational diabetes, gestational hypertension, pre-eclampsia, and cesarean birth. The World Health Organization suggests that pregnant women should get at least 150 minutes of moderate-intensity aerobic activity every week. In Nigeria, studies have shown that 47.6% of pregnant women have minimal knowledge about exercise, while 15.8% of them showed a negative attitude toward exercise. In this presentation, we will describe how we have leveraged a virtual self-care health coaching platform to support low-income pregnant women in following exercise regimens.

**Objective**

To evaluate the impact of digital health coaching on exercise in pregnant women in Nigeria.

**Methods**

This is a retrospective study using existing data on mDoc Healthcare’s digital platform, CompleteHealth. A total of 729 pregnant women enrolled in CompleteHealth from December 2021 to September 2022 and provided baseline exercise activity details at enrollment. They received virtual health coaching through messaging and voice calls from a primary health coach, with support from a nutritionist, fitness, and behavioral wellness coaches. The members were able to track their metrics and pregnancy progress on the CompleteHealth’s platform.

**Results**

Following asynchronous and synchronous digital nudges from coaches, the average change in exercise value for these 729 women was 38.88 minutes (baseline: 59 minutes, follow-up: 98 minutes), an improvement of 69.9%.

**Conclusions**

Digital health coaching provided has increased the engagement of pregnant women in physical activities. There is an opportunity to harness digital platforms to drive improvements in lifestyle modifications that may translate into positive maternal health outcomes.

## P69. Awareness of risk factors affecting development of infants among mothers and pregnant women in rural settings of Raigad District in Maharashtra State, India

### Rajani Mullerpatan, Triveni Shetty, Sailakshmi Ganesan

#### MGM Institute of Health Sciences

*BMC Proceedings 2024*, **18(5):**P69

Submission ID #: IMNHC1575

**Background**

Health literacy of mothers is pivotal in determining health outcomes of children through the antenatal, birth, infancy, childhood, and adolescent periods. The mother is the best-suited person on the health care team to monitor the child’s development right from intrauterine life. Particularly in resource-constrained settings, the mother can assume a critical role in monitoring a child’s growth and development if she is educated on child development. Hence, the present study was designed to understand the level of awareness of risk factors affecting the development of infants among mothers and pregnant women in the rural setting of Raigad district of Maharashtra state in India.

**Methods**

After giving informed consent, 103 mothers(including 13 pregnant women) in two villages of Raigad district of Maharashtra were interviewed in a door-to-door survey using a semi-structured questionnaire to record knowledge of mothers about antenatal, perinatal, and postnatal risk factors that may affect a child’s development.

**Results**

Average age of women was 27.8 yr (SD: 8.02). A total of 90% did not receive a formal education and 10% had received primary education.Thirty-two percent of the women were unaware that one’s maternal age determined child development; whereas 35% were unaware that premature delivery is a potential risk factor for child development. Twenty-five of the women were unware that iron/folic acid supplements had a role in child development.Seventeen percent were unaware of the importance of a baby’s immediate cry after delivery, whereas 29% were unaware that high-grade fever in infancy can cause regression of development. Twenty percent of the women did not consider development of milestones as an indicator of growth in infants. However, 70% of the mothers were aware that they had to report to community health workers or neighboring primary health care center if the infant presented with delayed head-holding and delayed walking.

**Conclusions**

Preliminary findings of ongoing study warrant an urgent need to educate mothers, including pregnant women about potential risk factors that may affect the development of the newborn and infant.Context-specific health literacy strategies, through indigenously designed mobile-based technology, are being developed to empower women at the primary health care level for early detection of developmental delay in infants from low- and middle-income countries.

## O70. Improved blood pressure outcomes through digital health coaching via completehealth in Nigerian women with hypertensive disorders in pregnancy

### Divine Akpan, Bih-Neh Estella, Esupat Lotasarwaki, Maria Moosa, Nneka Mobisson

#### mDoc Healthcare Ltd

*BMC Proceedings 2024*, **18(5):**O70

Submission ID #: IMNHC1567

**Background**

Hypertensive disorders in pregnancy are known contributing factors to increased morbidity and mortality of both the mother and baby, especially in sub-Saharan Africa. They cause 16% of maternal deaths within the region. Early detection, management, and compliance to treatment are critical to improving pregnancy outcomes. The principle of health coaching has been known to improve patients’ compliance with therapy, including hypertension treatment. The application of digital technologies that offer health coaching services is still an evolving concept.

At mDoc Healthcare, self-efficacy in managing chronic disease is afforded to members registered on the CompleteHealth^TM^ platform through health coaches sending nudges via voice calls and messages. Pregnant women registered on the platform receive these services and also access their imputed health metrics such as weight, blood pressure, and exercise to monitor their health progress.

**Methods**

This retrospective study was done to determine the impact of virtual health coaching through digital technology over the study period (between February and September 2022), and the blood pressure (BP) values of pregnant women with hypertension registered on the CompleteHealth^TM^ platform. Study participants were selected based on the following criteria: 1) Self-reported diagnosis of hypertension in pregnancy; 2) A baseline systolic blood pressure (SBP) > or = 140mmHg; or 3) A baseline diastolic blood pressure (DBP) > or = 90mmHg.

**Results**

Over the study period, 492 pregnant women had hypertension. Of these, 207 (42.07%) had both baseline and follow-up BP values, with average baseline SBP of 141.40 mmHg and DBP of 89.70 mmHg. The average follow-up values were SBP = 129.00 mmHg and DBP = 81.73 mmHg, signifying a change in SBP of -12.41 mmHg and DBP of -7.96 mmHg. There was also a 600% increase in the number of pregnant women with controlled hypertension at follow-up (138) compared to baseline (23).

**Conclusions**

The use of digital health coaching has been demonstrated through CompleteHealth^TM^ to significantly improve BP values of women with hypertension in pregnancy registered on the platform. There is therefore a place to scale up the use of digital health coaching to reduce the morbidity and mortality of hypertensive disorders of pregnancy.

## O71. Human-centered design: an innovative approach to preventing postpartum hemorrhage in selected states in Nigeria

### Adefemi Adewemimo, Temi Filani

#### Co-creation Hub

*BMC Proceedings 2024*, **18(5):**O71

Submission ID #: IMNHC1565

**Background**

The Smiles for Mothers program (SfM) aims to collaborate with the State Governments of Kano, Lagos, and Niger to develop and implement costed roadmaps for the adoption and rollout of 2018 World Health Organization (WHO) recommendations for postpartum hemorrhage (PPH) prevention.

**Methods**

Human-centered design (HCD) is a problem-solving approach that starts with the end-user, with a focus to incorporate their perspective in the process of designing solutions. Critical stakeholders in these states, who are the end-users, were identified to champion the successful adoption and rollout of the 2018 WHO recommendation.

SfM applied HCD in three phases:

- Co-research: We conducted 38 semi-structured interviews with health systems stakeholders, 68 immersion sessions with health care workers (HCWs) and influencers across 12 local government areas (LGAs), HCW surveys, and document reviews across the three states.
- Co-design: More than 300 ideas were co-generated with stakeholders around four prioritized opportunity spaces during the ideation workshops. The ideas were clustered into themes, filtered, and consolidated into implementable solutions.
- Co-refinement: State stakeholders categorized the solutions into high, medium, and low priority based on perceived impact and feasibility. Based on the states’ prioritization, the state stakeholders recommended rolling out 17 prioritized solutions in three waves over two years.

**Results**

Each of the three dates has achieved the following:

Developed and adopted costed roadmaps.

Policy adoption activities including constituting a technical working group for developing state guidelines.

A robust literature review on drug information centers (DICs) with findings on:

Objectives of DICs.

Challenges faced with running a DIC in low- and medium-income countries.

Recommendations to introduce the supply of uterotonics to relevant health facilities.

**Conclusions**

The successful adoption and rollout of the 2018 WHO recommendation for PPH prevention in the three states are linked to the use of the HCD approach. Seventeen implementable solutions were developed across four opportunity areas and these solutions were used to develop state-specific costed roadmaps for the adoption and rollout of 2018 WHO recommendations. Heat Stable Carbetocins (HSC) was included in Nigeria’s drug policies with the potential of contributing to improving the quality of maternal care in Nigeria even in resource-constrained settings.

## O72. Tools to guide school-specific investments in pre-service midwifery education: results from a field test in three African countries

### Nicole Warren^1^, Phelelo Marole^2^, Keoagetse Kgwabi^3^, Etta Forson-Addom^4^, Heloise Adanogou-d'Almeida^5^, Ashley Gresh^1^, Julie Mann^6^, Frances Gange^2^

#### ^1^Johns Hopkins University School of Nursing; ^2^Jhpiego; ^3^ECSACON; ^4^West African College Of Nursing; ^5^Federation of Associations of Midwives of Francophone Africa; ^6^Seed Global Health

*BMC Proceedings 2024*, **18(5):**O72

Submission ID #: IMNHC1555

**Background**

The COVID-19 pandemic exacerbated global challenges to quality pre-service midwifery education. In resource-constrained settings, educational institutions’ needs are complex and investments may not address the specific needs of a school, creating or even worsening inefficiencies. To address this challenge, African midwifery leaders co-created five educational capacity frameworks (ECFs), one for each of the five pre-service education domains: clinical sites, curriculum, faculty, students, and infrastructure/management. The ECFs quantify a school’s capacity, or ability to meet international and/or regional standards, in each domain. They are intended for internal school self-assessment that guides identification of school-specific investment and improvement priorities. The ECFs have recently been field-tested. Our objectives were to:

1. Report the findings from a field test of ECFs for five domains of pre-service midwifery education.
2. Describe participants’ feedback using the ECFs.

**Methods**

Teams in Ghana, Botswana, and Togo field-tested each of the five pre-service midwifery ECFs. Each team included representatives from a regional midwifery organization and a public midwifery school. We summarized each country’s self-assessment data and feedback on using the ECFs using descriptive statistics.

**Results**

In the three countries, capacity levels across domains ranged from 10% to 63%. Botswana reported the highest overall capacity (49%), followed by Ghana (41%), and Togo (24%). Overall, teams reported the highest capacity level for infrastructure and management; the lowest for clinical sites. Preliminary analyses suggest the ECFs are comprehensive, easy to use, and highlight school-specific challenges not previously recognized. Participants indicated the ECFs were acceptable, appropriate and feasible to use. The teams subsequently used the ECF data to draft a specific plan for investment and improvement in their priority domain.

**Conclusions**

The ECFs are a promising tool for midwifery schools to internally assess and prioritize areas for investment and improvement. Individual school results can guide internal budgetary decisions and help prepare for external assessments. Results from groups of schools have the potential to inform systems’ level investment. Additional research is needed to determine the ECFs’ appropriateness in other settings. Findings from this field test are being used to further refine the ECFs for future use.

## O73. Root cause analysis of maternal mortality in Bong County, Liberia: a case series study

### Jody Lori^1^, HaEun Lee^1^, Joseph Perosky^2^, Madison Horton^3^, Aloysius Nyanplu^4^

#### ^1^University of Michigan; ^2^Michigan State University; ^3^Columbia University; ^4^Bong County Health Team

*BMC Proceedings 2024*, **18(5):**O73

Submission ID #: IMNHC1543

Note the full manuscript published in April 2023 in *BMJ Open*:

(https://bmjopenquality.bmj.com/content/12/2/e002147)

## O74. Immediate skin-to-skin contact may have beneficial effects on the cardiorespiratory stabilisation in very preterm infants

### Agnes Linnér^1^, Stina Klemming^2^, Nils Bergman^1^, Siri Lilliesköld^1^, Hanne Markhus Pike^3^, Björn Westrup^1^, Siren Rettedal^3^, Wibke Jonas^1^, Karoline Lode^3^

#### ^1^Karolinska Institutet; ^2^Lund University Hospital; ^3^Stavanger University Hospital

*BMC Proceedings 2024*, **18(5):**O74

Submission ID #: IMNHC1531

Note the full manuscript published in April 2022 in *Acta Paediatrica*:

(https://onlinelibrary.wiley.com/doi/10.1111/apa.16371)

## O75. Safety, immunogenicity, and effectiveness of COVID-19 vaccines for pregnant persons: update from a living systematic review and meta-analysis

### Mabel Berrueta^1^, Agustin Ciapponi^1^, Agustina Mazzoni^1^, Ariel Bardach^1^, Fernando Argento^1^, Federico Rodriguez-Cairoli^1^, Jamile Ballivian^1^, Sara Reidel^1^, Beate Kampmann^2^, Edward Parker^2^, Andy Stergachis^3^, Sabra Zaraa^3^, Xu Xiong^4^, Sami Gottlieb^5^, Pierre Buekens^4^

#### ^1^Institute for Clinical Effectiveness and Health Policy; ^2^London School of Hygiene & Tropical Medicine; ^3^University of Washington; ^4^Tulane University School of Public Health and Tropical Medicine; ^5^World Health Organization

*BMC Proceedings 2024*, **18(5):**O75

Submission ID #: IMNHC1507

**Background**

There is growing evidence from clinical studies and real-world evidence about the safety, immunogenicity, and effectiveness of COVID-19 vaccines in pregnant persons. With the continuous and rapid growth of data, a living systematic review (LSR) is the best way to continuously collect and assess the latest research findings as they become available, and rapidly disseminate up-to-date evidence to assist with clinical and policy decision-making.

**Methods**

We are conducting an LSR and meta-analysis on the safety, immunogenicity, and effectiveness of COVID-19 vaccines for pregnant persons, following Cochrane guidelines, which is ongoing and can provide timely data to clinical and policy decision-makers such as the World Health Organization Strategic Advisory Group of Experts on Immunization. We designed an interactive web-based tool to share up-to-date findings of the LSR (https://safeinpregnancy.org/lsr/). The search is updated every two weeks to incorporate new, relevant records, including peer-reviewed papers and preprints. We are building an interactive and dynamic R-Shiny-based application to share evolving meta-analyses (including forest plots and summary tables) for key outcomes.

**Results**

Between March 1, 2022, and September 25, 2022, 98 studies have been included in the LSR, involving 394,522 pregnant women in 19 countries; only two studies were performed in low- and middle-income countries (LMICs). Ninety-two percent of the studies focused on mRNA vaccines. Primary outcomes are pregnancy complications, maternal and infant outcomes, vaccine effectiveness, immunogenicity, and adverse events following immunization in pregnant persons. We present the aggregate meta-analyses for each comparison and by the following pre-specified subgroups: pregnancy trimester; country income level; region; vaccine platform; dominant variant of SARS-CoV-2; study design; and primary series/booster vaccine. We expect results from many ongoing studies to become available in 2023, including results from studies for non-mRNA vaccines often used in LMICs.

**Conclusions**

Findings from this LSR and meta-analysis will inform evidence-based guidelines and recommendations regarding potential benefits and any harms of COVID-19 vaccines for pregnant persons. Our interactive web-based tool provides a useful paradigm for delivering up-to-date synthesis.

## O76. Demand-side interventions improve adherence to Iron and Folic Acid (IFA) supplements in pregnancy in Bangladesh, Burkina Faso, Ethiopia, and India

### Tina Sanghvi, Sebanti Ghosh, Abdulaziz Ali Oumer, Tamirat Walissa

#### FHI Solutions; FHI 360

*BMC Proceedings 2024*, **18(5):**O76

Submission ID #: IMNHC1486

**Background**

Adequate iron intake during pregnancy is essential to meet the needs of a growing fetus, blood volume expansion, and replacement of blood loss during childbirth. A daily IFA supplement during pregnancy is recommended by the World Health Organization; however, adherence is universally low. Strategies have failed to adequately address adherence. Our aim was to draw lessons from developing and operationalizing demand-side adherence interventions in Bangladesh, Burkina Faso, Ethiopia, and India.

**Methods**

We conducted global reviews on adherence to IFA and other long-term oral drug therapies, country-specific situational analyses, and formative studies to identify challenges and opportunities for developing intervention packages suitable for antenatal care (ANC) services. We based the demand-side interventions on Theory of Reasoned Action, Theory of Planned Behavior, and Stages of Change model. Implementation was facilitated by prioritizing IFA adherence during ANC contacts and engaging community and family networks to generate support for pregnant women. The impacts were measured by external agencies using randomized controlled evaluations.

**Results**

Knowledge increased and adherence was higher in intervention areas at endline: 139 tablets consumed in pregnancy compared to 94 in non-intervention areas in Bangladesh, 133 compared to 115 in Burkina Faso, 125 compared to 101 in Ethiopia, and 60 compared to 54 in India. Key interventions included: 1) promoting timely ANC contacts (considered an important gateway behavior) by involving community and family members; 2) improving access to high-quality counseling and uninterrupted IFA supplies; 3) providing counseling focused on the protocol, benefits, management of side effects and forgetfulness; 4) increasing community awareness about ANC and IFA; and 5) generating family support. Improvements along the program pathway included more ANC contacts; unrestricted free supplies; and pregnant women’s knowledge of anemia, belief in the benefits of IFA, self-efficacy, and family support to obtain and remember daily doses.

**Conclusions**

Demand-side approaches used to improve IFA adherence in ANC proved effective in four low- and middle-income countries and could contribute to maternal and newborn mortality and low birthweight reductions. ANC services may benefit from applying behavioral science theories to formative research and intervention design to strengthen adherence to other ANC interventions as well.

## O77. Every newborn-INDEPTH paradata study: improving measurement of stillbirths and neonatal deaths in standardised surveys

### Akuze Joseph Waiswa

#### London School of Hygiene & Tropical Medicine

*BMC Proceedings 2024*, **18(5):**O77

Submission ID #: IMNHC1484

**Background**

More than 2.4 neonatal deaths and 2.0 stillbirths occur globally. A total of 166 million births are unregistered, and significant data quality issues are faced when registered. Several aspects, including unclear questionnaire wording, context-specific understanding, omission and potential misclassification, affect the data quality. Survey data can improve with survey methodology, industrial statistical methods, machine learning, and content and qualitative methods. We aimed to develop methods for enhancing the quality of household surveys for measuring stillbirths and neonatal deaths using paradata from the Every Newborn-INDEPTH study (*n*=69,176), a randomized comparison of two household survey modules Full Birth History with additional questions on pregnancy loss (FBH+) and Full Pregnancy History (FPH) for measuring stillbirths and neonatal deaths in five Health and Demographic Surveillance Sites in countries between July 2017 and August 2018.

**Methods**

We analyzed the survey qualitative and quantitative data and paradata (>4 million survey audit trails). We developed data quality indices for stillbirths and neonatal death, patterns of missingness, and errors, and reviewed content-specific enablers and barriers for reporting stillbirths.

**Results**

Overall, the relative male risk for stillbirths and the sex ratios for stillbirths were above 100 in all sites. The stillbirth rate and neonatal mortality rate ratios ranged from 0.4 -to-0.8 and 0.4 -to-1.0 in the FBH+ and FPH, respectively. A total of 14%(11,740/86,365) of interviews were rejected at least once by interviewers because of inconsistencies in the interview and missing data. Patterns of missing data showed that at least 80% of the reported deaths were missing data on the day of death. Daily workload, differences in local definitions for stillbirths (terms interchangeably used for stillbirths and miscarriages), privacy concerns during the interviews and respondents’ fatigue were reported to affect the data quality of reporting of stillbirths and neonatal deaths. Good interviewer skills, using enabling mechanisms, including rephrasing questions and crossing earlier information provided in the interview, such as birth dates of siblings, were found to improve reporting of stillbirths and neonatal deaths.

**Conclusions**

Standardized guides are needed when implementing surveys. Understanding contexts and culture, omission, and misclassification of stillbirths in the survey and improving questions and software design can improve data quality.

## O78. Effects of acute exposure to conflict events on service use and birth outcomes among Palestinian refugees: results from electronic medical records linkage across five settings

### Zeina Jamaluddine^1^, Oona Campbell^1^, Hala Ghattas^2^, Miho Sato^3^, Ghada Ballout^4^, Hussam Al-Fudoli^4^, Shatha Albaik^4^, Gloria Paolucci^4^, Akihiro Seita^4^

#### ^1^London School of Hygiene and Tropical Medicine; ^2^American University of Beirut; ^3^Nagasaki University; ^4^United Nations Relief and Works Agency

*BMC Proceedings 2024*, **18(5):**O78

Submission ID #: IMNHC1477

**Background**

Intrauterine exposure to violent conflict potentially threatens birth outcomes. We explored the effects of exposure to conflict on service/intervention use patterns (use and timing of antenatal care and cesarean-section deliveries) and on stillbirth and prematurity among Palestinian refugees living in Jordan, Lebanon, Syria, West Bank, and Gaza.

**Methods**

Data on the location and timing of exposure to armed conflict were obtained from The Armed Conflict Location and Event Data Project. Data on location of residence, timing of pregnancy and birth, service/intervention use, and birth outcomes were obtained for 2017– 2020 from the United Nations Relief and Works Agency (UNRWA) for Palestine Refugees in the Near East’s electronic medical record system. We linked women’s antenatal records to 431,221 birth records to build a cohort and used logistic regression models to explore associations between exposure to any conflict event resulting in at least one fatality and outcomes at two time points: the first trimester of pregnancy and the month before delivery.

**Results**

Palestinian refugees residing in Syria were most exposed to conflict events (83% in the first trimester of pregnancy), followed by Gaza (26%), Lebanon (15%), West Bank (5%), and Jordan (5%). Conflict events before birth were similar. Women exposed to conflict events during the first trimester were 29% less likely to use UNRWA antenatal care services (OR=0.71, CI 0.70-0.72); those using them delayed timing of the first visit by 11.9 days (CI 11.3-12.5). Exposure to conflict events before delivery increased the odds of cesarean-section delivery (OR=1.4, CI 1.4-1.5) among all births. Exposure to conflict before birth also increased the odds of stillbirth (OR=1.4, CI=1.2-1.5), and of extremely preterm (OR=1.2, CI 1.0-1.4), very preterm (OR=1.1, CI 1.0-1.2) and preterm (OR=1.03, CI 1.01-1.07) deliveries (using a multinomial model with term delivery as the reference conflict exposure).

**Conclusions**

The results show acute exposure to conflict affects service use and harms babies, and points to the need to prioritize access to safe maternity services and focus on vulnerable pregnant women. We will further assess the timing/intensity of exposure to conflict in the three trimesters, including on outcomes such as size for gestational age.

## O79. Feasibility of a sustainable emergency obstetric care training system in Nigeria

### Charles A Ameh^1^, Hauwa Mohammed^1^, Eniola Kadir^2^

#### ^1^Liverpool School of Tropical Medicine; ^2^University of Ilorin Teaching Hospital

*BMC Proceedings 2024*, **18(5):**O79

Submission ID #: IMNHC1461

**Background**

As part of a strategy to reduce maternal mortality, skilled health personnel (SHP) competent in emergency obstetric and newborn care (EmONC) working within integrated teams to provide good-quality care are required. Standard EmONC training approaches in Nigeria are the partner funding dependent National Life Saving Skills (LSS) programme and the annual Nursing and Midwifery Council of Nigeria (NMCN) Mandatory Continuous Professional Development Programme (MCPDP) for practice license renewal.

We designed and implemented a Center of Excellence (CoE) for an EmONC approach to be embedded in the College of Nursing and Midwifery, Kwara state, Nigeria. The CoE was equipped for competency-based EmONC training and linked to the MCPDP’s practice license renewal program. We conducted a feasibility study on the CoE approach to train SHP in EmONC in Nigeria.

**Methods**

A mixed methods study design was used, guided by the Kirkpatrick Model for training evaluation (levels 1 and 2) and cost analysis. Specifically designed questionnaires were used to assess reaction to the training and changes in competence. In-depth interviews with participants, trainers, and policymakers were conducted to explore perceptions and experiences with the CoE approach. We compared the cost of training each participant in USD for all three training approaches.

**Results**

Three hundred SHP were trained from May 2021–May 2022, with nearly two-thirds of them nurse-midwives 194 (64%). Ninety-two percent of them found the course enjoyable, and 86.3% had all their expectations met for the course. There was improved confidence to perform both newborn and maternal EmONC skills. The most improved skill was newborn resuscitation (98%). Pre and posttest showed mean difference in score for knowledge at 10.3% (standard deviation [SD] 9.5%) and mean difference in score for skills at 44.5% (SD 18.8%).

The LSS programme runs for 21 days (lectures: 40%, practical: 30%, and clinical posting: 30%, cost per participant [pp] is $1,247). The NMCN EmONC training runs for five days (lectures: 90%, clinical posting: 10%, cost pp: $47). The CoE EmONC training runs for four days (lectures: 25%, simulation-based education and practical: 75%, videos: 5%, cost pp: $47).

**Conclusions**

The CoE model of EmONC training is feasible and a potentially sustainable EmONC training approach in Nigeria.

## P80. Onsite clinical mentoring strengthens Maternal and Newborn Health (MNH) care in Nepal’s devolved health system

### Kamala Shrestha, Shanti Mahendra, Maureen Dar Iang, Alison Dembo-Rath

#### Options Consultancy Services Ltd

*BMC Proceedings 2024*, **18(5):**P80

Submission ID #: IMNHC1452

**Background**

In 2015, Nepal became a federal state, creating seven Provinces and 753 local governments, each accountable for delivering quality health services. In this context of health sector devolution, the Nepal Health Sector Support Programme has worked alongside the Federal Department of Health Services to establish and develop a clinical mentoring program for maternal and newborn care staff. Provincial- and local-level governments implement the program based on the local context.

**Methods**

Onsite clinical mentoring focuses on improving knowledge, decision-making, and clinical skills of nurses at facilities that provide comprehensive or basic emergency obstetric care (CEmONC/BEmONC). There are two stages: potential mentors are identified from across health facilities and given a 7-day training on mentoring skills and refresher training on EmONC. Trained mentors then visit CEmONC/BEmONC facilities at the request of provincial and local governments to mentor maternity staff. During the visits, mentors also facilitate facility assessment for MNH readiness, covering eight domains of quality including patient dignity.

**Results**

Between 2017 and 2022, 330 mentors were trained at nine training sites. Collectively they have mentored more than 4,200 nurses and facilitated assessments and quality improvement plans at over 840 facilities. A total of 61% of mentees are based at facilities in mountains and hills, where the difficult terrain makes emergency referral of patients difficult, and good skills and confidence are crucial. Mentees scoring >85% increased from 26% to 65% by the third onsite mentoring visit. Facilities scoring >80% on service readiness increased from 67% to 78% over the program period. An online monitoring system now tracks implementation progress and improvement in skills. Coaching and mentoring guidelines have also been developed.

**Conclusions**

Clinical supervision and support has been historically weak in Nepal. The mentoring program, designed within the government system and funded through the annual work plan and budgets at all levels of government, is a sustainable method for providing structured, regular support to nursing staff. The program has enhanced communication between mentors and mentees, opening a channel of advice to staff via mobile phones and timely referral of patients from BEmONC facilities. Onsite mentoring avoids staff absences at short-staffed facilities and enables facility readiness.

## P81. Trends and inequalities in use of caesarian sections in Nepal from 1996-2016

### Suresh Mehata

#### Government of Nepal

*BMC Proceedings 2024*, **18(5):**P81

Submission ID #: IMNHC1446

**Background**

Since 2009, Nepal has been implementing a universal free delivery program (free normal delivery, assisted vaginal delivery, and cesarean sections) and also reimbursing travel costs. Nepal has made significant progress toward achieving the Sustainable Development Goals; however, income inequalities in cases of critical maternal health services, such as cesarean section, remain high. This analysis assesses the trends and inequalities in use of cesarean sections in Nepal.

**Methods**

This study uses the data from five rounds of Nepal’s Demographic and Health Surveys from 1996, 2001, 2006, 2011, and 2016. Sample weights were used in order to provide national estimates. All analyses were conducted using STATA 15. To assess the income inequalities rate difference, rate ratios, and concentration index [95% CI] and *p*-value were calculated over time considering the cluster sampling design.

**Results**

The study revealed that use of cesarean section increased from 1% in 1996 to 9% in 2016 [95% CI:7.7-10.6]. However, income inequalities remained high over 20 years, with only 2% of the lower incomes women receiving cesarean section compared to 28% of the higher incomes women. The lower incomes:higher imcomes ratio decreased from 23:00 in 1996 to 11.72 in 2016, whereas an increase in the rate differences were observed: 3.08 in 1996, 11.08 in 2006, 13.16 in 2011, and 25.84 in 2016. An increase in the concentration index was observed over the period, 0.47 (p:0.001); 0.60 (*p*< 0.001); 0.58 (*p*< 0.001) between the periods 1996, 2001, and 2006, respectively, and decreased to 0.50 (*p*< 0.001) in 2011, and 0.43 (*p*< 0.001) in 2016.

**Conclusions**

Use of comprehensive emergency obstetric care has been proven to be very critical in achieving the 2030 maternal mortality ratio targets. Income inequalities in use of cesarean section has been observed, in particular, underutilization by the lower incomes women and overutilization by the higher incomes women. The study emphasizes that the targeted interventions both in demand and supply side would be instrumental to address the gap.

Despite the free delivery services and transport incentives, cesarean section is still underutilized by the lower incomes women. This finding may require that the maternal health policies, strategies, and implementation plans be revisited. Also, overuse of cesarean section (>5%) by the higher incomes women indicates that government should plan to redistribute the scarce resources by developing a pro-poor policy.

## P82. Observed quality of intrapartum and postnatal care in the Dire Dawa administration of Ethiopia

### Bereket Yakob Yota^1^, DDA Maternity Care Pathways Study Fenot Team^2^

#### ^1^The University of British Columbia; ^2^Fenot Project

*BMC Proceedings 2024*, **18(5):**P82

Submission ID #: IMNHC1443

**Background**

Quality maternity care is crucial to enhance women’s positive experiences with care and prevent maternal and child morbidity and mortality. However, such care is always in short supply, especially in developing countries. This study aimed to assess the observed quality of maternity care services in the Dire Dawa Administration (DDA) of Ethiopia.

**Methods**

A cross-sectional study was conducted in the DDA’s nine health facilities (HFs) (two hospitals and seven health centers) from December 2020–May 2021. All women who visited the HFs for labor and delivery were invited to participate in the intrapartum and postnatal care observation and exit interviews. The study’s instruments were adapted from the Services Provision Assessment and Averting Maternal Death and Disability Study. The data were analyzed with STATA vs.17. Ethical clearance for the study was obtained.

**Results**

A total of 883 women aged 15–49 years were observed and interviewed. In the facility audit, 11.0%–33.3% of the signal functions of maternity care were unavailable. Twenty-nine percent of the health centers reported that care was disrupted due to the lack of electricity or water and hospitals experienced a lack of supplies for infection prevention. In the initial assessment of labor, only 7%, 4.9%, 5.4%, and 6.1% of the women were asked if they had vaginal bleeding, fever, severe headaches, or blurred vision, respectively. Similarly, the fetal heart rate (12%), fetal presentation (16%), maternal blood pressure (41%), and maternal pulse (48%) were not checked. In the immediate postnatal care period, the vital signs of 91%–97% of the women were not taken, and only 19% and 30% of newborns had received vitamin K and chlorhexidine, respectively. During their discharge, 40%–67% of the women were not counseled on one or more of the danger signs, exclusive breastfeeding, or postnatal care visits.

**Conclusions**

Considerable gaps in adhering to the standard clinical actions of maternity care were observed. Without quality maternity care, maternal and newborn morbidity and mortality reductions are unachievable. Ethiopia’s health authorities must implement strategies to enhance provider capacity and skills, facility readiness, and logistics systems to improve the quality of maternity care in the DDA.

## O83. The social, economic, emotional, and physical experiences and consequences of informal caregiving among caregivers for women with female genital Fistula in Uganda: a mixed methods study

### Alison El Ayadi^1^, Ashley Mitchell^1^, Hadija Haddy Nalubwama^2^, Suellen Miller^1^, Wagahta Semere^1^, Justus Barageine^2^, Abner Korn^1^, Susan Obore^2^, Ruby Lucas^3^, Josaphat Byamugisha^1^

#### ^1^University of California, San Francisco; ^2^Makerere University College of Health Sciences; ^3^University of Washington

*BMC Proceedings 2024*, **18(5):**O83

Submission ID #: IMNHC1436

**Background**

Informal caregivers remain critical across the continuum of care for women with genital fistula in lower-resource settings, especially where the health workforce is overburdened and underfunded. Similar to other stigmatized conditions, including HIV and tuberculosis, women with fistula navigate complex physical and social consequences and rely on informal caregivers for additional support. Understanding and supporting caregivers’ needs may improve outcomes for the caregiver and their patient. Thus, this convergent mixed-methods study explored the firsthand experiences of informal caregivers of women with fistula in Kampala, Uganda.

**Methods**

Caregivers accompanying women for fistula care at Mulago Hospital between January and September 2015 were recruited to participate in a sociodemographic and caregiving survey and an in-depth interview or focus group. Informed by the Stress Process Model, a binational research team analyzed quantitative findings using descriptive statistics and thematically analyzed qualitative data for meaningful patterns.

**Results**

Of 43 participants, 84% were female, 95% were family members, and most were married and formally employed in addition to caregiving. While 70% reported caregiving for less than 3 months, all described many personal care tasks and household responsibilities, averaging 22.5 hours per day. Four overlapping themes emerged highlighting social, economic, emotional, and physical experiences and consequences of caregiving with notable differences by caregiving length and setting as well as caregiver gender. A conceptual framework was developed depicting the ways unique caregiver circumstances and social context informed the caregiving experience and outcomes. Our findings underscore the duality of the caregiving role as caregivers summarized a mix of positive and negative circumstances often associated with the experiences and well-being of their patient.

**Conclusions**

Caregivers’ lived experiences and consequences are shaped by both individual characteristics of the caregiver and their patient in addition to social factors. Bolstering the health workforce to increase the national capacity for fistula-related care, both within health facilities and the community, is needed though informal caregivers are likely to remain essential in the meantime. Findings from this study may inform programs and policies that increase caregiving supports, while mitigating stressors to enhance the caregiving role and ensure its feasibility, particularly in lower-resourced contexts.

## P84. To what extent are maternal and child health, family planning, and nutrition services integrated in the national-level policies in Burkina Faso, Côte d’Ivoire, and Niger?

### Halima Tougri^1^, Katelyn Bryant-Comstock^2^, Wambi Maurice Yameogo^3^, Abou Coulibaly^1^, Denise Kpebo^4^, Isabelle Bicaba^2^, Marguerite Ndour^2^, Seni Kouanda^1^, Rachidatou Compaore^1^

#### ^1^Institut de Recherche en Sciences de la Santé; ^2^IntraHealth International; ^3^Institut Africain de Santé Publique; ^4^Institut National de Santé Publique

*BMC Proceedings 2024*, **18(5):**P84

Submission ID #: IMNHC1420

**Background**

Integrating maternal and child health (MNCH), family planning (FP), and nutrition services is one of the strategies for reducing maternal, neonatal, and infant mortality. A commitment to integrated care by policymakers can be reflected in its inclusion in policy and strategy documents. We analyzed the presence of integrated services in national policy documents in Burkina Faso, Côte d’Ivoire, and Niger to inform the improvement of current and future policies.

**Methods**

The team conducted a policy review of documents from Burkina Faso, Côte d’Ivoire, and Niger from June to July 2018. The document review was conducted using the READ approach (Ready materials, Extract data, Analyze data, Distill). Data were analyzed according to the target group (mother, child, or mother and child) addressed by the document and the theme (MNCH, FP, and nutrition) covered in the document. The different elements of the document considered in the analysis were the objectives, components, and activities. We defined integration as occurring when the document took into account more than one theme or target.

**Results**

Based on selection criteria, 28 policy and strategy documents on MNCH, FP, and nutrition were selected in all three countries. Findings show that most policy documents were focused on just the mother or child, not both. In all three countries, reproductive health policies did not integrate the new World Health Organization guidelines on postpartum family planning (PPFP). Similarly, the newborn health policies did not link to maternal health and do not include indications on PPFP. Also, no link was established between maternal nutrition and the state of the newborn; some of the nutrition guidelines remain focused on young child nutrition and do not address related issues, particularly FP and maternal nutrition. None of the FP policies integrated nutrition.

**Conclusions**

It is time to update national-level policies by including a vision of integrated service delivery of maternal and child health, FP, and nutrition services for mother-child pairs. Specifically, PPFP orientation must be integrated to newborn health and child nutrition policies.

## O85. Achievement of elective cesarean following surgical repair of female genital Fistula in Uganda

### Alison El Ayadi^1^, Hadija Haddy Nalubwama^2^, Ashley Mitchell^1^, Abner Korn^1^, Florence Nalubega^3^, Andrew Muleledhu^4^, Justus Barageine^2^

#### ^1^University of California, San Francisco; ^2^Makerere University College of Health Sciences; ^3^Kitovu Hospital; ^4^Kamuli Mission Hospital

*BMC Proceedings 2024*, **18(5):**O85

Submission ID #: IMNHC1413

**Background**

Childbearing following recovery from female genital fistula in lower-resource settings is important for some women. Cesarean birth is recommended by fistula caregivers to reduce adverse outcomes, yet is inconsistently achieved. Understanding factors influencing post-repair elective cesarean may inform clinical and counseling interventions. We sought to understand perinatal experiences and care-seeking among Ugandan women who previously underwent fistula repair.

**Methods**

A total of 189 participants in a retrospective cohort study of Ugandan women surgically treated for fistula within 10 years completed post-repair pregnancy. Quantitative measures captured sociodemographics, attitudes, and perinatal care. Thirty participants underwent in-depth interviews to understand nuanced post-repair pregnancy experiences. Quantitative analyses used descriptive statistics and logistic regression. Qualitative analyses were thematic.

**Results**

Half gave birth via elective cesarean (49%); 40% were vaginal, and 11% via emergency cesarean. Achievement of elective cesarean was significantly higher among women compared to young adults (53% vs. 28%), those in relationships without physical violence (52% vs. 21%), and those who had four or more antenatal care visits (56% vs. 42%). Socioeconomic status was positively associated. Other individual and household factors were marginally associated. Individual and provider values on the importance of elective cesarean were key influencers to achievement. Individuals who believed elective cesarean was important had over 12-fold odds of achieving elective cesarean (OR 12.0, 95% CI 2.74-52.9). Antenatal care providers were important influencers; those who reported their provider believed elective cesarean was very important had over 6-fold increased odds of elective cesarean (OR 6.24, 95% CI 1.76-22.10), and those whose antenatal care providers engaged them in birth planning had over 9-fold increased odds (OR 9.21, 95% CI 1.14-74.21). Individual fear of surgery was a barrier. Participant narratives revealed the important influence of partner support and social norms regarding cesarean section on the decision.

**Conclusions**

While fistula patients are routinely counseled on the importance of cesarean delivery post repair, many women do not have elective cesareans. It is critical to understand the varied influences on the cascade of decisions and behaviors required to achieving this guideline-supported birth mode in order to better support women and reduce adverse outcomes following fistula repair.

## P86. Inequities in coverage of maternal and newborn service coverage in nepal and opportunities to enhance equitable service coverage in federal context

### Chahana Singh^1^, Sushil Chandra Baral^2^, Bibek Kumar Lal^3^, Sagar Dahal^3^, Achyut Raj Pandey^2^, Binita Singh^3^

#### ^1^United Nations Children’s Fund; ^2^HERD International; ^3^Ministry of Health and Population, Nepal

*BMC Proceedings 2024*, **18(5):**P86

Submission ID #: IMNHC1403

**Background**

The neonatal mortality rate (NMR) in Nepal decreased from 58 deaths per 1,000 live births in 1990 to 17 deaths per 1,000 live births in 2020. The NMR declined by 3.7% between 1990 to 1999, 3.9% between 2000 to 2009, and 3.6% between 2010 to 2019 indicating that the pace of decline has been relatively slower in recent decades. Although notable progress has been made in reducing maternal and neonatal mortality at the national level, there are disparities across different socioeconomic strata. This study attempts to assess the inequities in coverage of maternal and newborn health (MNH) service coverage and identify the opportunities for equitable service coverage in the federalized context.

**Methods**

This was a multi-method study. We performed further analysis of the 2019 Nepal Multiple Indicators Cluster Survey data to identify the inequities in maternal and newborn health service coverage. We conducted consultative workshops at the provincial (three) and federal levels (one) and key informant interviews of policymakers, program implementers, service providers, academicians, and researchers working on newborn health.

**Results**

Coverage of MNH services (computed as composite index combining antenatal coverage, including blood pressure measurement, urine and blood test, institutional delivery, and postnatal care for newborns) was 73% in Bagmati province and 34 % each in Madhesh and Karnali provinces, demonstrating two-folds difference in service coverage. Development of locally tailored program interventions, micro-planning to cater health needs in underserved areas, efficient procurement and supply of medicines, expansion of services at primary-level facilities, promoting gatekeeping function, reducing caseload at tertiary-level facilities, linking facilities at multiple levels with appropriate referral system, effective implementation of free newborn care, and health insurance schemes were among the key strategies suggested to improve newborn health and overcome inequities in service utilization.

**Conclusions**

Substantial variation exist in MNH service utilization across provinces and based on socioeconomic strata. There are also myriad opportunities to enhance equitable health service coverage in the current federal context where the decision-making authority has been devolved to provincial and local governments, allowing more space for contextualized and locally tailored program implementation.

## O87. Sociodemographic and residential differences in maternal mortality and causes of death in Mozambique: results from a national sample vital registration system for mortality and cause of death

### Celso Monjane^1^, Almamy Kante^2^, Hafizur Rahman^3^, Sheila Nhachungue^1^, Emily Wilson^4^, Azarias Mulungo^1^, Nordino Machava^1^, Aveika Akum^2^, Ivalda Macicame^1^, Agbessi Amouzou^4^

#### ^1^Instituto Nacional de Saúde; ^2^John Hopkins University; ^3^International Centre for Diarrheal Disease Research, Bangladesh; ^4^Johns Hopkins Bloomberg School of Public Health

*BMC Proceedings 2024*, **18(5):**O87

Submission ID #: IMNHC1397

**Background**

Maternal mortality is an important health indicator for the overall health of a population. This study assessed the differences and causes of maternal deaths in Mozambique in the period of 2019–2020.

**Methods**

Data came from the national sample registration system, known as Countrywide Mortality Surveillance for Action (COMSA), that collects vital events and causes of death using verbal and social autopsy method in 700 geographic communities randomly distributed across the 11 provinces. Maternal deaths were extracted from the algorithm method (InterVA). The maternal mortality ratios (MMR) and causes of death distribution were compared by age, place of residence, region of residence, and place of death.

**Results**

A total of 117 maternal deaths were recorded in the COMSA platform in 2019 and 2020. The 2019–2020 national MMR was 391 per 100,000 live births [95% confidence interval: 354-427] with variations by regions: Central region (Zambezia, Tete, Manica, and Sofala provinces) at 418 [364-472], North region (Niassa, Cabo Delgado, and Nampula) at 407 [95%CI: 342-474] and South region (Inhambane, Gaza, Maputo Province, and Maputo City) at 291 [95%CI: 219-363]; by residence area: rural at 408 [95%CI: 366-451] and urban at 330 [95%CI: 260-400]; by place of death: health facility at 303 [95% CI: 263-343] and community at 548 [95%CI: 476-620]; and by age group of the woman at time of death: less than 20 years old at 316 [95%CI: 95% CI: 241-393], between 20–29 years old at 303 [95%CI: 261-347], between 30–39 years old at 588 [95% CI: 495-681], and between 40–49 years old at 886 [95% CI: 586-1187]. The most common causes of death were pregnancy-related sepsis (34%), ectopic pregnancy (24%), hemmorhage (9%), and abortion (6%).

**Conclusions**

The MMR remains high in Mozambique. Local policymakers should continue promoting health facility delivery while assisting as much as needed women who delivered at the community level. More attention is needed for women at higher risk pregnancy and those in Central and North regions. An improvement of quality of delivery care should also be strengthened in order to further reduce MMR at health facilities.

## O88. Implementing the robson Ten Group Classification System (TGCS) to monitor hospital cesarean section rates in Nepal: some early lessons

### Paras Chipalu, Shanti Mahendra, Alison Dembo-Rath

#### Options Consultancy Service Ltd

*BMC Proceedings 2024*, **18(5):**O88

Submission ID #: IMNHC1381

**Background**

Nepal’s population-based cesarean section (CS) rate has increased over the years, but the CS rate of 9% (2016) is still within the World Health Organization (WHO) reference of 5%–15%. Observed rates at the facility level vary due to case mix and that their appropriateness is difficult to assess as they could reflect either overuse or unmet need of CS. Unsafe provision of CS quality of care endangers patient outcomes. The Family Welfare Division (FWD) adapted the WHO Robson TGCS Guideline 2017 and piloted it in four hospitals, and was recently rolled out to 33 hospitals across the country.

**Methods**

With technical support from the Nepal Health Sector Support Programme, the FWD conducted trainings to roll out the Robson Guidelines, and a Central Monitoring Committee was formed for oversight on implementation and monitoring of the facility-based CS rates. A mobile phone-based application for reporting Robson data has been institutionalised within the government system. We present the findings from four pilot hospitals and learning from the technical support provided.

**Results**

Average CS rates from a six-month period showed Group 1 (nulliparous, single cephalic pregnancy, at least 37 weeks’ gestation, spontaneous labor) had the largest contribution to the obstetric population in all four hospitals, followed by Group 3 (multiparous, no previous CS, single cephalic pregnancy, at least 37 weeks’ gestation, spontaneous labor). CS rates per Robson group were higher for almost all groups across hospitals than the WHO reference, indicating a likely inappropriate use of the intervention for some cases in these hospitals. Critical shortage of staff, high workloads, lack of use of partograph, and lack of use of augmentation for non-progress of labor were some of the reasons for the inappropriate use of CS.

**Conclusions**

Robson classification-based monitoring can help rationalize CS rates, but this needs to be integrated into existing quality improvement processes and structures at the hospital. Health system issues, including addressing human resources shortages, supplies, and setting up mechanisms for responses based on evidence, should be addressed to positively influence practices at the hospitals.

## O89. The utility of rapid cycle health facility assessments to Improve Antenatal Care (ANC) service provision

### Prativa Baral^1^, Tsering Pema Lama^2^, Subarna Khatry^2^, Melinda Munos^1^, Joanne Katz^1^

#### ^1^Johns Hopkins Bloomberg School of Public Health, ^2^Nepal Nutrition Intervention Project Sarlahi

*BMC Proceedings 2024*, **18(5):**O89

Submission ID #: IMNHC1362

**Background**

Assessing facility readiness for the appropriate provision of ANC services is important to improve maternal and neonatal outcomes. Although service provision assessments capture facility readiness, they are only conducted every few years, limiting their utility for responsive programmatic and policy choices. Rapid cycle facility assessments provide a unique opportunity to strengthen country-led processes toward facility readiness by collecting and utilizing real-time data.

**Methods**

We collected facility readiness data specific to ANC in five health posts in Sarlahi, Nepal, using a rapid cycle approach (quarterly over a one-year period). Readiness was assessed across several domains: overall ANC inventory, infrastructure, equipment, essential medication, laboratory tests and diagnostics, human resources, and documentation and guidelines. Facility readiness scores were developed using two scoring methods (domain-weighted, simple-additive) and two item selection methods (all items, core items). Descriptive analyses were conducted to assess variability and distribution of readiness scores across time, domains, and facilities.

**Results**

When assessing scores across domains for all facilities and time points, domain-specific ranges of scores indicated high variability in readiness overall: Infrastructure (41%–72%), equipment (45%–71%), essential medication (20%–60%), laboratory tests and diagnostics (0%–85%), and documentation and guidelines (30%–90%), with a higher score indicating better readiness. Facility-specific scores over time pointed to infrastructure being the least variable (average change of 6.70%) compared to laboratory tests and diagnostics being most variable (average change 31.42%). Within each facility, certain items, such as calcium tablets and urine glucose tests, were observed to be most time-variant, whereas the availability of a functional manual blood pressure apparatus, stethoscope, and weighing scale were observed to be stable or time-invariant. Chronic facility challenges across all facilities were observed with regards to the availability of personal protective equipment.

**Conclusions**

In this small pilot study, variations in facility readiness differed by item, facility, time point, and domain. This study highlights the unique opportunity for rapid cycle health facility assessments to capture variability in domains or items over time, such that health systems stakeholders and governments can have timely data to act accordingly and ensure high ANC service quality.

## O90. Improving referral outcomes in Eastern Uganda through a social media digital platform

### Onikepe Owolabi, David Nelson, Allan Kiprop, Joanita Nakazzi, Irene Mirembe, Susan Tino

#### IntraHealth International

*BMC Proceedings 2024*, **18(5):**O90

Submission ID #: IMNHC1359

**Background**

In March 2021, the Serere Health Center IV quality improvement (QI) team observed that they received referrals from lower facilities without prior notification; ambulance movements were uncoordinated; and appropriate pre-referral treatments were not administered to mothers. This contributed to eight avoidable maternal and perinatal deaths from June–December 2020.

**Methods**

With support from the U.S. Agency for International Development’s RHITES-E Activity, led by IntraHealth International, in April 2021, the QI team and the District Health Team (DHT) mapped 15 referring health facilities and assigned and oriented focal persons at each facility on appropriate referral. During the orientation, participants agreed to create a WhatsApp group to address the critical issue of inter-facility communication. In May 2021, the district referral lead created the group and enrolled the 15 referral focal persons, 15 health facility managers, 25 midwives, two medical officers, 20 laboratory staff, and the operating theatre team. He periodically added members deemed important for patient management, including five DHT members, two obstetricians, and three blood bank staff. The referring facilities shared vital observations, general condition of the patient, and reason for referral on the group chat, and sought support in pre- and intra-referral management. The receiving facility prepared to receive and attend to the mothers before arrival. The district referral lead triaged patient referrals and drew a monthly schedule of health workers assigned to the ambulance.

**Results**

For July–December 2021, the proportion of mothers who received appropriate pre-referral treatment/management and escort increased from 21% to 100%; the average waiting time for referred mothers who required a cesarean section reduced from 60–120 minutes to 0–15 minutes; inter-facility referral delays reduced from 6–8 hours to 1–3 hours; the number of maternal deaths reduced from five in January–June 2021 to 1 in July–December 2021, and perinatal deaths from 73 to 59 in the same period across the district.

**Conclusions**

The use of social platforms such as WhatsApp can improve the effectiveness of referrals and lead to higher quality and timely access to care.

## O91. National programs for the prevention and management of postpartum hemorrhage and hypertensive disorders of pregnancy: the 2022 global survey

### Gaurav Sharma, Angie Noreiga, Patricia Gomez, Aleefia Somji, Andre Blockett, Suzanne Stalls

#### Jhpiego

*BMC Proceedings 2024*, **18(5):**O91

Submission ID #: IMNHC1356

**Background**

Postpartum hemorrhage (PPH) and hypertensive disorders of pregnancy (HDP) are leading direct causes of maternal deaths in low- and middle-income countries. USAID’s MOMENTUM projects (MOMENTUM Private Healthcare Delivery [MPHD] and MOMENTUM Country and Global Leadership [MCGL]) jointly implemented a survey to: 1) improve our understanding of changes and sustainability of evidence-based practices since the last global survey in 2012; 2) understand how countries are working to integrate updated World Health Organization global guidelines; and 3) understand the private sector’s role in PPH and HDP management.

**Methods**

From January to May 2022, 31 countries in sub-Saharan Africa, South and Southeast Asia, and Latin America and the Caribbean completed a survey on PPH and HDP practices and policies in the public and private sectors. Using purposive sampling, we identified national experts in maternal health policy, education, procurement and distribution logistics, information systems, and public and private sectors to review nationally available data and policy documents, and reach consensus on survey questions. Composite scores were developed for key quantitative indicators and qualitative data were analyzed using a thematic approach to identify bottlenecks and scale-up opportunities.

**Results**

In summary, 58% and 35% of countries received an overall composite score greater than 80% for PPH/HDP drug availability and 81% and 48%, respectively, for updated national guidelines. We found 65% of countries scored greater than 80% for capacity-building efforts. Countries received lower scores for scopes of practice for midwives (48%), poor integration of indicators into information systems (35%), and quality and procurement policies (65%). We will present results across all survey domains disaggregated by public and private sectors and also report on bottlenecks and opportunities for scale-up.

**Conclusions**

The findings generated several recommendations: prioritize integration of updated global technical guidelines; improve uptake through multiple channels including professional education, mentorship and supervision; strengthen professional associations’ roles; strengthen quality assurance, procurement and supply chains to improve availability and quality of essential drugs and commodities; expand midwives’ scope of practice; systematically engage and improve clinical governance of private sector to strengthen quality and improve reporting of essential maternal and newborn health indicators. We call for increased public-private partnerships, addressing quality-of-care gaps and greater investment in health systems.

## O92. Advanced wearable sensors for comprehensive intrapartum and postpartum monitoring in high- and low-resource settings

### Jessica Walter, Soham Patel, Lian Yu, Jong Yoon Lee, Dhruv Seshadri, Steve Xu

#### Sibel Health Inc.

*BMC Proceedings 2024*, **18(5):**O92

Submission ID #: IMNHC1351

**Background**

Intrapartum and postpartum monitoring of obstetrical patients is critical in the early identification of hemorrhage, hypertensive disorders, and infections.

**Methods**

Sibel Health (Niles, IL, U.S.) has developed the ANNE®One system that includes time-synchronized wireless chest (cleared by the U.S. Food and Drug Administration [FDA]), limb (FDA cleared), and abdominal (FDA pending) patche—the sensors capture and stream all maternal vital signs (heart rate via ECG, respiratory rate, pulse arrival time as a surrogate for continuous blood pressure, pulse oxygenation, temperature, uterine contractility, patient position/laterality, heart sounds, total sleep time, and sleep-related apnea events) and fetal heart rate. The sensors are rechargeable with low-cost consumables, waterproof operation, and extended battery life (>5 days). The sensor system has been deployed in four countries (Ghana, India, U.S., and Zambia) for maternal monitoring on 11,893 pregnant subjects to date.

**Results**

Successful field trials of pregnant women between 25 and 41 weeks’ gestation in both high-resource settings (*n*=91) and low-resource settings (*n*=485) demonstrate the system’s performance, usability, and accuracy compared to gold-standard wired monitoring systems. Mean differences are -0.09 beats per minute (BPM) for heart rate, -0.19% for SpO2, and -0.45 breaths per minute (RPM). The system demonstrates a high degree of agreement with traditional blood pressure cuffs demonstrating an average error of less than 10 mmHg at discrete time points and a mean difference of 0.4 mmHg. Additional studies on a set of 25 healthy adults displayed a mean difference of 0.34 bpm for heart rate (SD: 1.34 BPM) and 0.20 RPM for resting rate. Qualitative results suggest a strong preference for wireless monitoring (84% of *n*=57 pregnant individuals) with no concerns regarding data privacy (96%). Usability results exhibited strong support for the wireless nature of the ANNE®One system (85% of *n*=10 pregnancy-related health care providers reported a positive or very positive experience). No adverse events were reported in any pregnant subject.

**Conclusions**

The results confirm the accuracy, feasibility, and scalability of deploying the ANNE®One system for continuous and wireless monitoring of intrapartum and postpartum obstetrical patients in a wide range of health care settings.

## O93. Landscape analysis of community resilience to disasters by addressing sexual and reproductive health and rights in Gaibandha and Satkhira Districts

### Aminul Haque^1^, Mohammad Mainul Islam^1^, Mahbub Alam^1^, Rabiul Islam^1^

#### ^1^University of Dhaka; ^2^Pathfinder

*BMC Proceedings 2024*, **18(5):**O93

Submission ID #: IMNHC1348

**Background**

Bangladesh is one of the most vulnerable countries to climate change, which has a huge impact on maternal and child health. This study analyzed community resilience to disasters by addressing sexual and reproductive health services (SRHS) and rights of the disaster-affected vulnerable populations in Gaibandha and Satkhira Districts. Following a mixed-method approach, combining both qualitative and quantitative methods, attempted to sketch a holistic picture of socioeconomic and health stresses associated with disaster-related shocks and of people’s decision-making processes and actions toward disaster resilience with crucial attention to sexual and reproductive health care services.

**Methods**

Mixed-method research was conducted on pregnant women who are vulnerable to climate change. A quantitative survey was carried out in Gaibandha and Satkhira Districts for an estimated 645 households. Married women aged 18–49 years old were interviewed from each of the selected households. Key informant interviews were carried out with relevant individuals such as those responsible for delivering health care, managing disasters, and providing disaster-related support, and with those, particularly young men and women, who provided different types of support voluntarily during disasters to the disaster-affected people.

**Results**

Frequent exposure to different types of disasters such as floods, riverbank erosion, cyclones, tidal waves, water logging, and water salinity made the socioeconomic condition of the households very vulnerable. Almost 87% of households in Gaibandha had experienced displacement within the last five years, which is around 36 percentage points lower than the prevalence of displacement in Satkhira. The health of the disaster-exposed population is affected in multiple ways. A vast majority of the respondents perceived that physical and mental health, including SRHS, is affected during natural disasters. Findings show that disasters cause socioeconomic disadvantages and food insecurity and create barriers to SRHS for girls and women when seeking antenatal and postnatal care during disasters.

**Conclusions**

Vulnerability to climate change affects families in multiple ways and the family uses their main resources for coping with shelter and food, and rebuilding their livelihood, which adversely affects SRHS, maternal and child health services, and newborn care. Intensive attention is needed for both the management of livelihood and maternal and child health services in disaster-prone areas.

## O94. Implementing the Supervision, Performance Assessment, and Recognition Strategy (SPARS) approach to improve maternal and newborn commodity management in District pharmacies of Madagascar

### Serge Ramahazomanana^1^, Maya Gershtenson^1^, Faly Razafimahatratra^1^, Tiana Ravelonarivo^1^, Aline Mukerabirori^1^, Patrick Raherinjatovo^1^, Jane Briggs^1^, Mohamed Patrice Diallo^2^, Laurent Kapesa^3^, Azzah Al-Rashid^3^, Patricia Norolalao^3^, Aishling Thurow^1^

#### ^1^Management Sciences for Health; ^2^Population Services International; ^3^United States Agency for International Development

*BMC Proceedings 2024*, **18(5):**O94

Submission ID #: IMNHC1345

**Background**

In Madagascar, the maternal and newborn health (MNH) commodity supply chain faces many challenges: district pharmacies (Pha-G-Dis) encounter gaps related to adhering to the Central Medical Store’s cyclical order schedule, accurately quantifying the commodities they need, and with comprehensive and timely reporting of stock status data. To address these challenges, the USAID-funded Improving Market Partnership and Access to Commodities Together (IMPACT) project adapted the SPARS approach from Uganda and has been implementing it in 78 Pha-G-Dis (of 114) in Madagascar since 2020.

**Methods**

SPARS is a data-driven, multi-pronged strategy that combines supervision, on-the-job training, and provision of tools and guidelines with structured performance reviews to identify and prioritize issues and encourage progress by rewarding improvements. Regional supervisors assess the Pha-G-Dis in stock management, ordering, distribution, and reporting of commodities including for MNH, roughly once a quarter and provide needs-based onsite support to plan and implement local solutions to address identified gaps. All 78 IMPACT-supported Pha-G-Dis received at least one supervision visit between April and September 2020 and at least one follow-up visit between October 2020 and September 2021.

**Results**

Among the 78 Pha-G-Dis, the proportion classified as “performing” (score of 90% or higher) increased from 5% to 27% (four to 21 Pha-G-Dis), and the proportion classified as “weak” (score of 75% or less) decreased from 29% (23 Pha-G-Dis) to 5% (four Pha-G-Dis). These results are supported by the end-user verification survey in 2021, which found that, among the 20 Pha-G-Dis surveyed, stock-out rates were much lower for MNH commodities, including misoprostol (79% stock-out rate in October 2020 compared to 36% in October 2021), chlorhexidine (89% compared to 23%); oxytocin (35% compared to 24%); and gentamicin (15% compared to 8%).

**Conclusions**

These data suggest that SPARS is an effective approach to improve district-level supply chain management of essential maternal and newborn commodities in Madagascar and other similar settings. Further analysis is needed to determine statistical significance of the results.

## O95. Creative storytelling as a tool to foster patient involvement and drive quality improvement in neonatal care: experiences from Kamuzu Central Hospital, Malawi

### Mercy Phiri^1^, Michelle Heys^2^, Deliwe Nkhoma^3^, Esther Harawa^4^, Steve Khenai^4^, Fannie Steven^5^, Regina Lusubiro^6^, Khadija Sanudi^5^, Emma Wilson^2^, Helen Todd^7^, Msandeni Chiume^8^, Lekodi Magombo^4^

#### ^1^Parent and Child Health Initiative Malawi; ^2^University College London; ^3^Global Health Informatics Institute; ^4^ArtGlo; ^5^Kamuzu Central Hospital; ^6^United Nations Children’s Fund; ^7^Independent Consultant; ^8^Kamuzu University of Health Sciences

*BMC Proceedings 2024*, **18(5):**O95

Submission ID #: IMNHC1342

**Background**

Patient and public involvement is key to improving quality of care (QoC). We conducted a public engagement project to address QoC concerns at Kamuzu Central Hospital (KCH), Malawi, and strengthen the relationships between service beneficiaries and health care professionals (HCPs) delivering neonatal care.

**Methods**

We invited 10 mothers whose babies received neonatal intensive care between December 2020 and July 2021. We invited women from both rural and urban areas, with varying levels of literacy and whose babies had a range of conditions and outcomes. Mothers attended two creative storytelling workshops, where they were supported to share their stories of pregnancy, motherhood, and experiences of seeking health care. They captured and documented their chosen story in their communities using participatory arts methodologies (photography, film, and drawing). The mothers showcased these narratives at an exhibition at KCH, attended by Ministry of Health officials, senior KCH management, HCPs, and nongovernmental organization representatives. A final workshop was held between the mothers, and health care and quality improvement professionals to enable critical reflection on the QoC issues highlighted within the mothers’ stories and to identify priority areas for improvement.

**Results**

Participants shared a range of stories including community beliefs surrounding childbirth, community involvement in childcare, as well as experiences of maternal and neonatal care within health facilities. HCPs and mothers devised a joint action plan to address QoC issues within the neonatal intensive care unit. Action points for the HCPs include increasing feeding time, correctly putting babies on CPAP, attending to babies when they cry, eradicating cockroaches in the kangaroo mother care (KMC) unit, supporting mothers to put babies on KMC, and improving patient access to the ombudsman. Action points to be taken forward by mothers include monthly visits to the KMC ward to provide peer support and encouragement to other mothers. The senior matron monitors progress on the action plan on a quarterly basis.

**Conclusions**

Creative storytelling is an effective method to foster dialogue and enable patient-driven quality improvement. Next steps include scaling up this initiative to other hospital departments and building on the trust generated between HCPs and mothers to pilot family-centered interventions.

## O96. Co-creation with health care providers of clinical guidelines for maternity care in Dar es Salaam, Tanzania (The PartoMa Project)

### Natasha Housseine^1^, Tarek Meguid^2^, Brenda Sequeira Dmello^3^, Monica Kujabi^1^, Haika Monica Osaki^1^, Luciana Chamwi^4^, Thomas Wiswa John^1^, Columba Mbekenga^5^, Eunice Pallangyo^4^, Dan Wolf Meyrowitsch^1^, Jos Van Roosmalen^6^, Thomas Van Den Akker^6^, Hussein Kidanto^4^, Nanna Maaloe^1^

#### ^1^University of Copenhagen; ^2^University of Cape Town; ^3^Comprehensive Community Based Rehabilitation Tanzania; ^4^The Aga Khan University; ^5^Herbert Kairuki Memorial University; ^6^VU University Amsterdam

*BMC Proceedings 2024*, **18(5):**O96

Submission ID #: IMNHC1335

**Background**

Universal clinical practice guidelines (CPGs) are often unachievable, particularly in low-resources settings. Despite this, adaptation of CPGs to local settings rarely happens. We describe the process of co-adapting CPGs for health care providers of maternity care in five hospitals in Dar es Salaam, Tanzania.

**Methods**

The co-creation was based on a participatory approach and took place between June and December 2021. It was based on a prototype of CPGs developed during the PartoMa pilot phase in Zanzibar and involved: 1) a multi-stakeholder team of co-creators; 2) review of emerging global evidence and assessment of contextual realities; 3) iterative cycles of feedback from focus group discussions (FGDs) with co-creators, followed by modifications of the guidelines until no additional concerns were expressed. Input from women obtained from exit interviews was also incorporated.

**Results**

A total of 100 co-creators (78 skilled birth attendants from the frontline and maternity unit in-charges, 10 representatives from Dar es Salaam’s health management teams, and 12 national/international maternal health experts) participated in the co-creation through rounds of 11 FGDs/workshops and individual reviews of the CPGs. The PartoMa intervention resulted in a simple 24-page infographic pocket-sized book and associated on-the-job training. The integrated content spans the intrapartum and immediate postpartum periods and contains routine and emergency obstetric and newborn care aligned with best possible practices in these overburdened facilities. Unique features include reduced frequency of routine assessment and information load; red, yellow, and green codes to enable rapid response to complications; and clearer guidance on management of prolonged labor and indications of obstetric interventions. The Dar es Salaam version of PartoMa includes new content on for example, respectful maternity care, decision support for cesarean section, photographs, and language modifications. Health care providers at the five hospitals have been trained in the use of these CPGs.

**Conclusions**

Context-adapted CGPs are warranted to assist skilled birth attendants in providing best possible evidence-based and respectful care in resource-constrained settings. The process of adapting guidelines is time and resource consuming; however, early continuous engagement of end-users and other stakeholders appeared crucial for intervention acceptability, ownership, and sustainability.

## P97. An evaluation of indicators in routine DHIS2 data and prediction modelling for neonatal mortality to inform data improvement in Tanzania

### Josephine Shabani^1^, Joy Lawn^2^, Nahya Salim^3^, Christine Bohne^4^, Louise Tina Day^2^, Eric Ohuma^2^

#### ^1^Ifakara Health Institute; ^2^London School of Hygiene & Tropical Medicine; ^3^Muhimbili University of Health and Allied Sciences; ^4^NEST360

*BMC Proceedings 2024*, **18(5):**P97

Submission ID #: IMNHC1331

**Background**

Globally, an estimated 2.4 million newborn deaths and >2 million stillbirths occur every year. The neonatal mortality rate (NMR) in Tanzania is ~20 per 1,000 live births. The Tanzanian Government has prioritized improving maternal and newborn care and committed to the Sustainable Development Goals (SDGs), notably SDG3.2 for fewer than 12 neonatal deaths per 1,000 live births by 2030. However, the quality of newborn data, including neonatal mortality in routine health information systems has not been systematically assessed.

**Methods**

Analysis of data quality of reported national neonatal indicator data from Tanzania District Health Management Systems-2 (DHIS2) from 2015–2020 was evaluated. The World Health Organization quality-of-care framework was used to map neonatal indicators according to availability, and internal, and external consistency dimensions. Subgroup analysis of neonatal deaths data from 28 regional hospitals and 184 district/council hospitals in Tanzania was done to understand the gaps in neonatal mortality data reporting.

**Results**

A total of 10,326 records were extracted from DHIS2 labor and delivery and postnatal care records between 2015–2021. Moreover, 48,468 neonatal death records were extracted from death registry records. Twent-four Every Newborn Action Plan indicators were mapped, but analyses focused on eight indicators with data in Tanzania DHIS2. NMR was consistently underreported—the NMR reported for 2015–2021 was 5.1 on average [range 3.3–6.2] over seven years. Moreover, the reported NMRs and stillbirth rates from DHIS2 is consistently lower compared to the Tanzania Demographic and Health Survey and United Nations-Inter Agency Group for Child Mortality Estimation.

District hospitals reported 35%–44.2% proportion of neonatal mortality between 2015–2021, while regional hospitals reported 27.0%–39.9%. Although the dispensary level has the lowest proportions for reported deaths (below 5%) between 2015 to 2021, they reported high proportions of deliveries ranging between 30% to 40%, followed by health centers, which shows an increasing trend for reporting deliveries (22% in 2015 to 33% in 2021).

**Conclusions**

Neonatal indicator data reporting and tracking should be strengthened in Tanzania’s Routine Health Systems, especially at lower-level health facilities where most births occur. Low reporting of NMR indicates a major data gap in data capture and reporting.

## P98. The workload of health care providers in three Sub-Saharan African countries: Burkina Faso, Côte d’lvoire, and Niger

### Denise Kpebo^1^, Katelyn Bryant-Comstock^2^, Antarou Ly^3^, Wambi Maurice Yameogo^4^, Sujata Bijou^2^, Halima Tougri^3^, Marguerite Ndour^2^, Orsot Tetchi^1^, Sablé Stéphane Parfait^1^, Seni Kouanda^3^

#### ^1^Institut National de Santé Publique; ^2^IntraHealth International; ^3^Institut de Recherche en Sciences de la Santé; ^4^Institut Africain de Santé Publique

*BMC Proceedings 2024*, **18(5):**P98

Submission ID #: IMNHC1329

**Background**

Most primary health care facilities in sub-Saharan Africa are staffed using the provider-population ratio or fixed staffing norms by facility type. These methods fail to account for variation in workload depending on service utilization and cannot adequately match the human resource needs of different health facilities. The Workload Indicators of Staffing Need (WISN) method uses workload to determine staffing needs in a given facility. The aim of this study was to assess the current workload and staffing needs of maternal and child health services in 12 primary health care facilities in Burkina Faso, Niger, and Côte d’Ivoire using the WISN method.

**Methods**

Using document reviews, in-depth interviews with health care providers, and observations to obtain the data needed, we used the WISN methodology to estimate the required number of staff in a given facility. Then, we calculated both the WISN difference (current − required staff), and the WISN ratio (current staff/required staff), indicating understaffing (WISN ratio < 1), overstaffing (WISN ratio > 1), or adequate staffing (WISN ratio = 1). Using the WISN ratio, we assessed the work pressure that health workers experience ((1 − WISN ratio) * 100)—the lower the WISN ratio, the higher the workload pressure.

**Results**

In Niger and Côte d’Ivoire, maternity services in three out of four health facilities were understaffed (WISN ratio < 1), with very high workload pressure (41%–60%), while in Burkina Faso they were either overstaffed or at least equally staffed (WISN ratio ≥ 1). In Niger, all dispensary services were understaffed, and in Côte d’Ivoire, rural services were understaffed. In Burkina Faso, most of these services were either overstaffed or adequately staffed. Workload pressure was very high in rural health facilities in Niger and Côte d’Ivoire (41%–60%), and extremely high in one rural health facility in Burkina Faso (>60%).

**Conclusions**

The study results show that most health facilities were understaffed, with a geographic disparity between rural and urban areas. Although this is a pre-intervention study, it confirms the need for health authorities to implement regular and systematic WISN assessment studies for a better allocation of health staff.

## O99. Bubble Continuous Positive Airway Pressure (CPAP) with a Blender (Vayu) is associated with immediate improvement in physiologic and clinical parameters

### Emily Ahn^1^, Mathew Mselle^2^, Aisa Shayo^3^, Anna Sechu^3^, Jeffrey Perlman^1^

#### ^1^Weill Cornell Medicine; ^2^Kilmanjaro Medical Center Hospital; ^3^Kilmanjaro Christian Medical University

*BMC Proceedings 2024*, **18(5):**O99

Submission ID #: IMNHC1324

**Background**

Respiratory distress syndrome (RDS) often complicates the clinical course of premature infants <2000 grams, with progressive respiratory failure if untreated. CPAP has been shown to modulate the clinical course. A novel compact bubble CPAP system with an ambient/oxygen blender developed by Vayu has the potential to modify the course of RDS. The objective was to determine whether use of the Vayu CPAP will be associated with improvement in physiologic and clinical parameters—i.e., heart rate (HR), O2 saturation, FiO2 requirement, respiratory rate (RR), respiratory distress assessed by Silverman score (SS)—and whether it modifies the clinical course of premature infants with RDS.

**Methods**

The observational study was conducted at KCMC between March and August 2022. Premature infants <2000g with early RDS were eligible for Vayu CPAP. Data collected included pre-/post-CPAP HR, RR, FiO2, O2 saturation, and SS, and data collection was repeated at one, six, 12, and 24 hours. Data were analyzed using paired and student’s t-test.

**Results**

We evaluated 52 infants. Mean birthweight (BW) and gestational age (GA) were 1423g and 31.2 weeks, respectively. Infants who died (*n* = 16) were of lower BW than survivors (*n* = 36) (1253g versus 1545g) but comparable GA (*p* = 0.15). At one hour, the HR ↓ by a median 4 BPM (*p* = 0.01), O2 saturation ↑ by 4.9 ± 7.6% (*p* = 0.00005), FiO2 decreased by 2 ± 1.04 (*p* = 0.05), and SS ↓ by 0.45 ± 0.67 (*p* = 0.00005). These differences were amplified at 24 hours. Starting pressure decreased when comparing the initial 26 versus the last 26 infants (*p* = 0.04). CPAP was initiated later for the initial 26 versus the last 26 infants—21 versus two hours (IQ range 43, 22) (*p* = 0.01). The initial temperature was lower for infants who died versus survivors—median 35.8 versus 36.4 (*p* = 0.01)—and duration of CPAP less—74 versus 121 hours (*p* = 0.06). A positive blood culture was noted in 5/16 infants who died.

**Conclusions**

Vayu CPAP was associated with immediate improvement in physiologic and clinical parameters with progressive improvement over 24 hours. Provider use of CPAP was optimized over time. Death occurred in 30% of infants and was associated with infection in 30%. While CPAP improves initial respiratory status, a larger care bundle is necessary to have a greater impact on neonatal mortality.

## O100. Embedding inclusive, Supportive, and Dignified Maternity Care (SDMC) in public health facilities: an evaluation of a theory-driven service delivery intervention package

### Bilal Iqbal^1^, Shaikh Waqas Hameed^2^, Bushra Khan^3^, Muhammad Asim^2^, Sarah Saleem^2^, Sameen Siddiqi^2^

#### ^1^London School of Hygiene and Tropical Medicine; ^2^The Aga Khan University; ^3^University of Karachi

*BMC Proceedings 2024*, **18(5):**O100

Submission ID #: IMNHC1322

**Background**

Disrespect, discrimination, abuse, and lack of emotional support characterize intrapartum care in many low- and middle-income countries. Although the World Health Organization (WHO) provides affirmative guidelines to address this issue, no operational model has effectively incorporated those guidelines into routine public health services. Therefore, we developed and evaluated a theory-driven service delivery intervention to promote inclusive SDMC in public health systems.

Our SDMC intervention worked with maternity teams in a participatory, consensus-driven process, theoretically underpinned by the COM-B framework (capability, opportunity, and motivation drivers for respectful maternity behavior). Components included (a) capacity strengthening of staff (respectful care, rights-based care, medical ethics, values clarification, psychosocial support) and (b) improved governance (enhanced management information systems, care coordination) and accountability mechanisms (client feedback, performance review). Technical content drew from WHO’s intrapartum care guidelines, WHO’s mental health GAP strategy, and contextual evidence.

**Methods**

Employing a pre-/post-evaluation design, SDMC was implemented in six secondary-level health facilities in two districts of Pakistan. Research with service providers informed its development. Following a three-day training for 122 maternity staff, implementation lasted six months. Evaluation entailed a pre-/post-comparison (*n* = 313) of women’s experiences of SDMC during childbirth, and a post-intervention assessment with service providers about implementation feasibility.

**Results**

Formative research identified factors impeding SDMC: capacity—institutional guidelines and training opportunities were missing; opportunity—no systematic mechanism for eliciting clients’ psychosocial needs and preferences and limited companion engagement; and motivation—clients deemed uncooperative, normalized mistreatment, and no monitoring or accountability. Nevertheless, SDMC training assessment showed significant improvement in knowledge (15%) and attitudes (18%). Supportive supervision enabled effective integration of SDMC components within maternity services. Postnatal women reported substantially less mistreatment, with a relative change of 50% (*p*-value < 0.001). Similar changes were recorded across all mistreatment domains. Post-intervention, service providers also confirmed a positive impact on components of the health system.

**Conclusions**

This pioneering intervention links the improved capacity of maternity teams with information systems, governance measures, and accountability. Given its promise for promoting inclusive, supportive, and dignified childbirth in health facilities, a large-scale effectiveness

## O101. Concordance of a Modified Downes’ score by physicians and nurses when assessing respiratory distress syndrome in the Ethiopian neonatal network

### Danielle Ehret^1^, Mahlet Gizaw^2^, Bogale Worku^3^, Asrat Demtse^4^, Matebe Hailu^5^, Gesit Metaferia^2^, Amanuel Hadgu Berhe^6^, Kaitlin Kessler^7^, Ryan Kessler^7^, Marie Dunn^8^, Agneta Golan^9^, Miroslav Stavel^10^, Jaroslava Belava^11^, Erika Edwards^1^, Jeffrey Horbar^1^, Michael Dunn^8^

#### ^1^Vermont Oxford Network; ^2^St. Paul’s Hospital Millennium Medical College; ^3^Ethiopian Pediatric Society; ^4^Addis Ababa University; ^5^Gondar University; ^6^Mekelle University; ^7^University of Vermont Children’s Hospital; ^8^Sunnybrook Health Sciences Centre; ^9^Ben Gurion University of the Negev; ^10^Royal Columbian Hospital; ^11^Vancouver Coastal Health

*BMC Proceedings 2024*, **18(5):**O101

Submission ID #: IMNHC1315

**Background**

Respiratory distress syndrome (RDS) is the leading cause of mortality of preterm infants in low- and middle-income countries (LMICs). Lack of diagnostic tools along with scarcity of human resources highlight the need for objective clinical assessments of RDS that can be implemented by multidisciplinary newborn care providers. Our objective was to assess the correlation of modified Downes’ scores (MDS) assigned by physicians and nurses in the Ethiopian Neonatal Network (ENN).

**Methods**

We included preterm infants admitted from June 2020–2021 to four tertiary neonatal intensive care units (NICUs) of the ENN presenting with respiratory distress and treated with respiratory support within 24 hours of admission. Nurses and physicians received training on the MDS and national guideline for initiation of continuous positive airway pressure (CPAP), with treatment prompted by MDS of 4 or greater with an oxygen requirement. The MDS was incorporated into admission assessments, and when feasible, concurrent and independent assessments were recorded by a nurse and physician. We calculated the kappa statistic to determine the nurse and physician correlation for each component of the MDS and the total score, and evaluated the concordance of scores above and below the treatment threshold of 4.

**Results**

Of the 1,016 eligible infants admitted, 1,005 (99%) had MDS recorded on admission; 818 (81%) had scores reported by nurses; 997 (98%) had scores reported by physicians; and 817 (80%) had scores reported concurrently by both. The kappa statistic for MDS components ranged from 0.88 to 0.92 and was 0.89 for the total score. Of the 817 infants concurrently assessed and scored, 98% had concordance above or below the score-based treatment threshold of 4.

**Conclusions**

Incorporation of an objective clinical assessment on admission for preterm infants with RDS was feasible in tertiary NICU settings of the ENN. The kappa statistics of the components of the MDS and total score showed near-perfect agreement between nurse and physician assessments, translating to a high degree of concordance in score-based treatment recommendations. As LMIC NICUs prioritize improving RDS care and quality of care, these findings reinforce nursing empowerment and task shifting standardized assessments and initiation of CPAP within the context of multidisciplinary teams.

## O102. Mothers’ and caregivers’ perceived acceptability of a digital data capture and clinical decision support intervention (Neotree) in neonatal intensive care units in two low-resource settings

### Albert Dube^1^, Michelle Heys^2^, Emma Wilson^2^, Fabiana Lorencatto^2^, Tarisai Chiyaka^3^, Deliwe Nkhoma^4^, Tim Hull-Bailey^5^, Msandeni Chiume^6^, Simbarashe Chimhuya^7^, Kristina Curtis^2^

#### ^1^Malawi Epidemiology and Intervention Research Unit; ^2^University College London; ^3^Biomedical Research and Training Institute, Zimbabwe; ^4^Global Health Informatics Institute; ^5^Neotree Charity; ^6^Kamuzu University of Health Sciences; ^7^University of Zimbabwe

*BMC Proceedings 2024*, **18(5):**O102

Submission ID #: IMNHC1308

**Background**

Neotree is a digital intervention that aims to improve quality of newborn care in low-resource settings by supporting health care professionals to complete an electronic health care record for every admitted baby, conduct standardized clinical assessments, and adhere to evidence-based clinical guidelines in the diagnosis and management of neonates. Few digital interventions co-developed with health care professionals in low-resource settings have been evaluated from the client/ caregiver perspective. Yet this is critical to ensure care is client centered. Our aim was to assess the acceptability of Neotree from the perspective of mothers and caregivers of neonates (end users) admitted to three neonatal intensive care units (NICUs) in Malawi and Zimbabwe.

**Methods**

We conducted 28 semistructured interviews with parents/carers of neonates who had received care at three pilot intervention sites. Interview guides were informed by the Theoretical Framework of Acceptability (TFA). We adopted a purposive sampling strategy to capture perspectives from first-time and more experienced mothers/caregivers, from both rural and urban areas. We conducted a combined framework and thematic analysis. We deductively coded participant responses to domains of the TFA, and inductively generated themes within each domain.

**Results**

We identified 25 acceptability themes. The purpose of Neotree as a data capture and clinical decision support tool was generally well understood (TFA: intervention coherence), and Neotree was perceived to be effective in ensuring a reliable record of care, supporting health care professionals to make decisions and filling knowledge gaps (TFA: perceived effectiveness). For most, answering questions for data capture was perceived as low burden (TFA: burden), and Neotree was regarded as good fit with participants’ values toward the digitalization of health care (TFA: ethicality). Barriers to acceptability included understanding the tablet to be a personal phone (TFA: intervention coherence), feeling too ill/tired to answer questions (TFA: burden), lacking skills/knowledge around digital tools in health (TFA: self-efficacy), and having a preference for paper-based systems (TFA: opportunity costs).

**Conclusions**

While the pilot intervention requires optimization to address identified barriers, overall Neotree was highly acceptable to parents and caregivers of neonates, which bodes well for both intervention’s sustainability and equity of care.

## O103. Measuring the causes and circumstances of deaths beyond 42 days postpartum in Kenya, The Gambia, Malawi, and South Africa: implications for global monitoring and postpartum care

### Ursula Gazeley^1^, Georges Reniers^1^, Clara Calvert^2^, Julio Romero Prieto^1^, Kobus Herbst^3^, Momodou Jasseh^1^, Sammy Khagayi^4^, Veronique Filippi^1^

#### ^1^London School of Hygiene & Tropical Medicine; ^2^University of Edinburgh; ^3^Africa Health Research Institute; ^4^Kenya Medical Research Institute

*BMC Proceedings 2024*, **18(5):**O103

Submission ID #: IMNHC1306

**Background**

Our previous research demonstrated that mortality remains elevated until four months postpartum in sub-Saharan Africa, but little is known about the causes of pregnancy-related deaths (PRDs) beyond the standard 42-day postpartum period. This evidence gap is severe in sub-Saharan Africa, where cause of death information largely fails to meet international standards. We are now investigating the causes of deaths associated with this prolonged risk and the implications for measurement and clinical care.

**Methods**

We used prospective, longitudinal Health and Demographic Surveillance System and verbal autopsy data from Kenya, The Gambia, Malawi, and South Africa to estimate the cause of death for PRDs that occurred within one year of pregnancy. Using multinomial regression, we compared the most likely cause of death for PRDs occurring within versus beyond 42 days postpartum.

**Results**

From 1998 to 2020, 1,444 deaths occurred up to one year postpartum, of which 516 occurred beyond 42 days. Across all ages, HIV and tuberculosis were the second leading cause after direct obstetric causes for PRD within six weeks postpartum and were the dominant cause of PRD beyond six weeks. Relative to deaths from hemorrhage within six weeks postpartum, deaths beyond six weeks had 18.6 (95% CI 26.8–215.5) and 17.3 (18.0–156.3) times higher odds of HIV/tuberculosis and other infectious causes, respectively, and these deaths were most frequently related to knowledge gaps and health system failures. Consistent with the obstetric transition, there was a shift from infectious causes of PRD within six weeks in 1998–2009 to cardiovascular and hypertensive diseases in 2010–2020.

**Conclusions**

The dominance of HIV, tuberculosis, and other infectious diseases in PRDs in the (extended) postpartum period suggests more research is needed to know how long pregnancy might aggravate (co-)infection from infectious diseases. Postpartum care guidelines must emphasize potential vulnerabilities in the extended postpartum period to unresolved chronic conditions and infectious disease comorbidity. The limitations of verbal autopsy data to differentiate late maternal from late PRD emphasize the need for better cause of death data beyond six weeks postpartum in sub-Saharan Africa.

## O104. The effect of e-Registration and mHealth on institutional deliveries in the hazard-prone areas of Southern Bangladesh: an open-label two-arm nonrandomized controlled cluster trial

### Anika Tasneem Chowdhury^1^, Ahmed Ehsanur Rahman^1^, Sabrina Jabeen^1^, Zeeba Zahra Sultana^2^, Shams El Arifeen^1^, Ahmed Hossain^3^

#### ^1^International Centre for Diarrhoeal Disease Research, Bangladesh; ^2^North South University; ^3^University of Sharjah

*BMC Proceedings 2024*, **18(5):**O104

Submission ID #: IMNHC1300

**Background**

Increasing facility delivery is mandatory to reach the goal of bringing down the maternal mortality ratio to less than 70 deaths per 100,000 live births by 2030. In the era of digitalization, the introduction of e-registration and mHealth may aid the Government of Bangladesh to reach this target. The southern part of Bangladesh is a hazard-prone area and service uptake at health facilities is lower there. This study aimed to determine the effect of an e-registration tracking system and mHealth counseling on institutional deliveries in hazard-prone areas of Southern Bangladesh.

**Methods**

This was a six-month open-label two-arm nonrandomized controlled cluster trial conducted in 2022. Chandpur Sadar, Faridganj, and Kachua subdistricts of Chandpur district served as intervention areas and Bhola Sadar, Charfesson, and Lalmohan subdistricts of Bhola district were control areas. We collected data at baseline and endline of the study period using a structured questionnaire. After screening using the inclusion and exclusion criteria, we enrolled 700 pregnant women from intervention and control areas, each registered in the Family Welfare Assitant register. We followed the women until their delivery. Per-protocol analysis was done and random-intercept mixed-effect logistic regression was performed to explain the relationship of e-registration and the mHealth package with institutional delivery.

**Results**

274 mothers from the comparison and 298 mothers from the intervention arms entered the final analysis. In both arms, the majority of the women were 21–25 years old, had completed their secondary level of education, were unemployed, and had at least one child. About 38% of women in the comparison group and about 71% of women in the intervention group had institutional deliveries. After adjusting for potential confounders, the likelihood of institutional deliveries was around three times more (AOR: 3.23; 95% CI: 1.69, 6.19; *p*-value <0.001) among the women who received the e-registration and mHealth intervention package compared to those who did not receive the intervention.

**Conclusions**

Institutional delivery is still uncommon in Southern Bangladesh despite several interventions. Innovative approaches such as e-registration and mHealth counseling will be helpful to bring women to health facilities.

## O105. Investing in our future: developing a multidimensional service package to support return to school of pregnant and mothering learners

### Jane Kelly^1^, Chelsea Coakley^1^, Lucie Cluver^2^, Elona Toska^1^, Abigail Ornellas^3^, Janina Jochim^3^, Lulama Sidloyi^3^, Hlokoma Mangqalaza^3^

#### ^1^University of Cape Town; ^2^University of Oxford; ^3^Independent Consultant, Cape Town, South Africa

*BMC Proceedings 2024*, **18(5):**O105

Submission ID #: IMNHC1298

**Background**

The education sector is well placed to respond to the support needs of adolescent mothers, as recognized in the South African Department of Basic Education (DBE)’s recently launched Policy on the Prevention and Management of Learner Pregnancy. In collaboration with the DBE, this project aims to develop an evidence- and stakeholder-informed, multidimensional service package for pregnant and mothering learners in South African secondary schools, focused on achieving school retention during pregnancy and return post-birth, school progression, and timing future pregnancies.

**Methods**

We adopted a three-pronged approach in the development of this support package:

- We conducted secondary data analysis of a cohort study of 1,046 adolescent mothers and their children residing in the Eastern Cape province of South Africa to test the effect of multiple policy-aligned protective provisions on select goals of the DBE policy.
- We reviewed evidence from systematic reviews and local intervention models that target pregnant and adolescent mothers.
- We undertook participatory research with 13 young research advisors (ages 19–24) with recent or current secondary schooling, pregnancy, and parenting experience.

**Results**

Preliminary findings from our three-pronged approach suggest that an ideal service package to support return to school of pregnant and mothering learners would include three primary components that would begin in the antenatal period and continue after birth: 1:1 case management, peer-based support groups, and onward referrals to/linkages with relevant health and social services, including available childcare services, social grants, and antenatal and postnatal care. The young research advisors also suggested that participatory methodology enabled them to express themselves more easily, and that participatory methods enabled social support and critical dialogue between young people on the issue of young parenthood.

**Conclusions**

Education is a clear social determinant of health, and it is thus critical to find ways to promote the continued school engagement of pregnant and mothering learners as a way to strengthen health and social outcomes for both them and their children. It will be important to consider how the identified services and provisions, and their synergies, can be successfully integrated into existing support structures to ensure that adolescent mothers are optimally supported.

## O106. Evaluating the implementation of an integrated health systems approach to increase the use of chlorhexidine for newborn care in Madagascar

### Ingabire Magera^1^, Maya Gershtenson^1^, Elmard Rabotovaosolo^1^, Voahirana Ravelojaona^1^, Bonaventure Nzeyimana^1^, Feno Rakotoarimanana^1^, Lovahasina Vahatriniaina^2^, Vololoniaina Rasoanandrasana^3^, Azzah Al-Rashid^4^, Patricia Norolalao^4^, Aishling Thurow^1^, Serge Xueref^1^, Laurence Laumonier-Ickx^1^, Tamar Chitashvili^1^

#### ^1^Management Sciences for Health; ^2^Direction de la Santé Famille; ^3^Ministry of Public Health, Madagascar; ^4^United States Agency for International Development

*BMC Proceedings 2024*, **18(5):**O106

Submission ID #: IMNHC1294

**Background**

The World Health Organization recommends umbilical cord care with chlorhexidine (CHX) in high newborn mortality settings. In Madagascar, the Ministry of Public Health has adopted this standard, but certain cultural practices and health system bottlenecks have hindered its implementation. The U.S. Agency for International Development-funded ACCESS program supports the Ministry of Public Health to promote facility-level CHX cord care in 11 regions. ACCESS supported the Ministry of Public Health in identifying root causes of limited CHX use and finding context-specific solutions, including orientation of community health volunteers and health workers on the importance of CHX, development of CHX job aids, supporting community health volunteers in educating the community, advocacy with stakeholders for sustainable procurement and distribution, and engaging expectant mothers in Savings and Internal Lending Communities to purchase CHX. This analysis aims to determine the potential effect of these interventions on CHX use in Madagascar.

**Methods**

Using national District Health Information Software II (DHIS2) data from 1,350 health facilities in 11 ACCESS-supported regions, we compared the number of newborns who received CHX at birth between January–June 2020 and January–June 2022 using a paired series mean comparison analysis.

**Results**

There was a significant increase in the mean proportion of newborns who received CHX (*p* < 0.001) from 15.6 (CI 95% [14.1–17.1]) in the first period to 67.2 (CI 95% [65.2.7–69.0]) in the second within ACCESS-supported regions. While this analysis focused on 11 ACCESS-supported regions, the increase in CHX cord care was also observed at the national level (from 19.1 to 64.4, *p* < 0.001) during the same period, as these activities were led by the Ministry of Public Health and scaled up by other partners throughout Madagascar.

**Conclusions**

CHX cord care improved both in intervention and nonintervention regions from the start of the intervention (2020). These results suggest the importance of implementing needs-based, multilevel holistic health system approaches to improve CHX cord care. Working with and through local systems and building local capacities to identify and address local problems has the potential to scale up CHX cord care and reduce preventable newborn mortality from infectious complications in Madagascar and similar settings.

## O107. The state of midwifery education, regulation, and practice in East and Southern Africa Region, 2022

### Muna Abdullah Ali, Jyoti Tewari

#### United Nations Population Fund

*BMC Proceedings 2024*, **18(5):**O107

Submission ID #: IMNHC1291

**Background**

A *Lancet* article published in 2020 shows that achieving universal coverage of midwives could avert 67% of maternal deaths by 2035^1^. Data and evidence-based programming is instrumental to guide and track investment on midwifery programs. This assessment is the second regional assessment (the first being in 2017) of the state of midwives in the East and Southern Africa (ESA) region commissioned by the United Nations Population Fund (UNFPA) ESA regional office. It provides a comprehensive assessment of the current state of the region’s sexual, reproductive, maternal, newborn, and adolescent health (SRMNAH) workforce in 23 countries. It gives clear evidence of the region’s progress and identifies bottlenecks and challenges that must be addressed.

**Methods**

The study relies on the methodology developed for the State of the World’s Midwifery Report 2014, co-chaired by UNFPA, the World Health Organization, and the International Confederation of Midwives. An online survey was sent to UNFPA country focal persons and midwifery associations who engage the Human Resources for Health directorate of ministries of health for the responses.

**Results**

Across the 23 countries in the ESA region, there are just over 800,000 SRMNAH workers. Over half (53%) are nurses without additional training in midwifery and 11% are doctors (general practitioners, obstetricians/gynecologists, and pediatricians). Just under one in five (18%) are professional or associate professional midwives or nurse-midwives.

The region needs more than 300,000 more midwives, and current education and deployment levels are not keeping up with rising needs. Availability of midwives varies significantly between countries, for example, the midwife to 10,000 population ratio is 33.2 in Seychelles and 0.1 in Burundi.

Only 12 countries have distinct regulatory processes for midwives. Only 10 countries require proof of continued professional development for re-licensing

**Conclusions**

Countries in the region will need to accelerate their progress in order to meet Sustainable Development Goal (SDG) targets. Investing in midwives is a key accelerator in the effort to achieve the SDG target of maternal mortality ratio of 70 per 100,000 live births by 2030. This study brings out gaps and challenges in the midwifery education, regulation, and practice in 23 countries in the ESA region that need to be addressed urgently.

**Reference**

1. Nove A, Friberg IK, de Bernis L, et al. Potential impact of midwives in preventing and reducing maternal and neonatal mortality and stillbirths: a Lives Saved Tool modelling study. *Lancet Glob Health*. 2020; (published online Dec 1.) 10.1016/S2214-109X(20)30397-1

## O108. Provider communication and coaching for parents of hospitalized newborns and sick young infants in Kenya: provider and caregiver perspectives

### Chantalle Okondo, Charity Ndwiga, Timothy Abuya, Pooja Sripad, Charlotte Warren

#### Population Council

*BMC Proceedings 2024*, **18(5):**O108

Submission ID #: IMNHC1283

**Background**

The relationship between health care providers and caregivers in the management of hospitalized newborns and sick young infants (SYIs) is an essential element of quality care. Meaningful participation in caring for infants during hospitalization has demonstrated improved caregiver-infant bonding, reduced parental stress, and greater confidence in caring for their children once at home. We developed a provider behavior change (PBC) approach to improve experiences of care for caregivers of hospitalized newborns in Kenya. The intervention oriented providers on communication skills and how to coach caregivers on nurturing care: optimizing nutrition, safeguarding sleep, protecting skin, minimizing stress and pain, positioning, and handling.

**Methods**

This analysis sits within a broader PBC activity. It draws on in-depth interviews with 15 parents of hospitalized newborns and SYIs and 16 health care providers from five Kenyan hospitals in August 2021. Inductive and deductive thematic analysis was used to describe the nature of provider-caregiver interactions. The data was triangulated based on the study sites and respondents to find similarities and differences across themes.

**Results**

Providers were able to confidently coach caregivers on three of the five elements: optimizing nutrition, including breastfeeding or how to feed using a nasogastric tube; safeguarding sleep and protecting skin, including keeping the baby warm and practicing skin-to-skin contact. The other two elements of minimizing stress and pain and positioning and handling were practiced less often. Some caregivers reported providers were attentive, listening to caregivers, while others were harsh, unfriendly, or dismissive of their opinions. Few caregivers could correctly state their newborn’s treatment plan or describe instances of shared decision-making with providers. The variability in care was associated with provider attitudes, increased workload, and insufficient support from administration to create spaces for caregivers to participate in care.

**Conclusions**

While many caregivers were satisfied with the care they received, some provider attitudes resulted in mixed reception and unwillingness to partner with caregivers in providing newborn care. While providers may work in unyielding environments, efforts to support providers, avail reference materials to standardize care, and improve infrastructure are integral to enhancing communication with caregivers and overall quality of care.

## O109. Lessons learned from community referral and follow-up of sick young infants with possible severe bacterial infection in Turkana County, Kenya

### Samuel Mbugua^1^, Laura Oyiengo^2^, Peter Mwaura^1^, Wilson Laimbila^3^, Andrew Emuria^4^, Daniel Gatungu^1^, Jesse Gitaka^1^

#### ^1^Mount Kenya University; ^2^United Nations Children’s Fund; ^3^Population Council; ^4^Ministry of Health, Kenya

*BMC Proceedings 2024*, **18(5):**O109

Submission ID #: IMNHC1277

Note the full manuscript published in June 2023 in *Global Pediatrics*:

(https://www.sciencedirect.com/science/article/pii/S2667009723000167)

## O110. Beyond respectful maternity care: a research priority exercise and working group process on respectful newborn care

### Hagar Palgi-Hacker^1^, Emma Sacks^2^

#### ^1^George Washington University School of Public Health; ^2^Johns Hopkins Bloomberg School of Public Health

*BMC Proceedings 2024*, **18(5):**O110

Submission ID #: IMNHC1275

Note the full manuscript published in February 2022 in *GHSP:*

(https://www.ghspjournal.org/content/10/1/e2100292.full)

## P111. Viral load monitoring among women initiating antiretroviral therapy through the Elimination of Mother-to-Child Transmission of HIV (eMTCT) program in Mafeteng, Lesotho

### Matholoana Lenkoane, Rachel Chinyakata, Mamakamane Nyapisi, Leseli Masuku, Mojalefa Mosoeu, Tebeli Sekoai, Tokelo Molise, Moeti Moleko, Nakululombe Kwendeni, Petronella Chirawu, Mpolokeng Mohloai

#### mothers2mothers

*BMC Proceedings 2024*, **18(5):**P111

Submission ID #: IMNHC1269

**Background**

HIV prevalence among pregnant and breastfeeding women (PBFW) in Lesotho (22.8%) indicates that if elimination of mother-to-child transmission of HIV (eMTCT) interventions are not scaled up, pediatric HIV infections will continue to reverse gains made through child survival programs. PBFW with suppressed viral load are less likely to infect their infants with HIV. During the review period, in Mafeteng district, the viral load suppression rate was >95% among PBFW, and the average infant HIV infection rate was 1.5%, well below the target of 5%.

**Methods**

We conducted a retrospective viral load monitoring review of eligible PBFW using data extracted from consultation HIV care cards for the period July 2021 to June 2022. PBFW eligibility for viral load testing was defined by the 2019 Lesotho antiretroviral therapy (ART) and 2020 eMTCT guidelines: viral load suppression was monitored when PBFW were on ART for six months, then every three months from their previous test. Suppression was considered <1,000 copies/mL during the review period.

**Result**

Of the 1,283 women eligible for viral load testing in July 2021, 1,069 (83%) were reached and 1,046 (98%) were suppressed. In December 2021, 1,307 women were eligible for testing, 1,248 (95%) were reached, and 1,219 (98%) were suppressed. In March 2022, 1,375 women were eligible for testing, 1,287 (94%) were reached, and 1,265 (98%) were suppressed. In June 2022, 1,923 women were eligible for testing, 1,862 (97%) were reached, and 1,835 (99%) were suppressed. Thus, there was a meaningful improvement in viral load test coverage from July 2021 (83%) to June 2022 (97%).

**Conclusions**

Strategies to improve viral load monitoring include mentorship on the importance of viral load monitoring for every health care provider who will be in contact with PBFW, as clients need accurate and appropriate information before adhering to given health appointments; intensification of viral load blood drawn in the community for PBFW who missed their clinic appointment; and promoting demand creation for viral load services.

## P112. National mandatory grain fortification legislation decreases anemia prevalence among non-pregnant women of reproductive age: findings from multiple demographic and health surveys

### Vijaya Kancherla^1^, Kelsey Rondini^1^, Wanqing Xu^2^, Yan Chai^3^, Helena Pachon^4^

#### ^1^Emory University; ^2^Harvard TH Chan School of Public Health; ^3^University of California; ^4^Food Fortification Initiative

*BMC Proceedings 2024*, **18(5):**P112

Submission ID #: IMNHC1268

**Background**

Two billion people are affected by anemia globally. Most are women of reproductive age (WRA) and those residing in low- and middle-income countries (LMICs). Maternal anemia is associated with adverse outcomes for both the mother and the baby. Large national population-representative studies examining the impact of national grain fortification policies on the prevalence of anemia among WRA for recent years are lacking. The objective of our study was to determine whether mandatory national grain fortification policies reduce the prevalence of anemia among non-pregnant WRA.

**Methods**

We examined national food fortification policy characteristics from the Global Fortification Data Exchange (GFDx) database and anemia prevalence data from the Demographic and Health Surveys (DHS). In total, 21 LMICs, with and without national grain fortification policies, completing at least two DHS between 2000 and 2018, met study eligibility. We applied the difference-in-differences approach to compare changes in the prevalence of anemia among WRA in 10 countries with and 11 countries without fortification between each DHS year. Odds ratios (ORs) and average marginal effects, along with 95% confidence intervals (CIs), were calculated and adjusted for individual-, household-, and country-level factors.

**Results**

Our analytic study sample included 96,334 and 874,984 WRA in countries with and without fortification, respectively. Overall, countries with fortification in the pre- versus post-fortification period showed 27% decreased odds of anemia (aOR=0.73, 95% CI=0.63, 0.85) and a 7.47 percentage point decrease in the mean anemia prevalence (average marginal effect: −7.47, 95% CI=−11.03, −3.92), compared to countries without fortification, after controlling for selected individual-, household-, and country-level factors.

**Conclusions**

Our findings, using nationally representative DHS data and applying a recommended analytic method to measure policy effectiveness, suggest significant reductions in anemia prevalence in WRA in countries with mandatory grain fortification compared to those without. Implementing national mandatory grain fortification in LMICs would effectively reduce anemia resulting from micronutrient deficiencies among WRA. This has the potential for improved maternal and child health outcomes and reduced morbidity and mortality in newborns.

## O113. How much do vulnerable groups count in the global financing facility? Content analysis of stillbirths and maternal-newborn investments in policy documents for 10 African countries

### Joël Arthur Kiendrébéogo^1^, Mary Kinney^2^, Meghan Kumar^3^, Kafando Yamba^4^, Issa Kaboré^4^, Asha S. George^2^, Joy Lawn^3^

#### ^1^University Joseph Ki-Zerbo; ^2^University of the Western Cape; ^3^London School of Hygiene & Tropical Medicine; ^4^Recherche pour la Santé et le Développement

*BMC Proceedings 2024*, **18(5):**O113

Submission ID #: IMNHC1260

**Background**

The Global Financing Facility (GFF) aims to accelerate progress to meet 2030 Sustainable Development Goals for reproductive, maternal, newborn, child, and adolescent health and nutrition (RMNCAH-N). Our study examines how GFF policy documents address maternal and newborn health (MNH) plus stillbirths, given the high burden this group bears relative to investments made.

**Methods**

We undertook a content analysis of national GFF documents (investment cases [ICs] and project appraisal documents [PADs]) for 10 African countries: Burkina Faso, Côde d’Ivoire, Ethiopia, Kenya, Liberia, Malawi, Nigeria, Senegal, Tanzania, and Uganda. The analysis framework considered a progression from mentions of related outcomes (maternal and newborn mortality and stillbirths) and mindset (framing and content) to measures (indicators) and money allocated (investment).

**Results**

Mentions of maternal and newborn mortality are robust in all country ICs and included as targets or indicators, whereas stillbirth is only included as an indicator in three ICs and mentioned in six ICs. PADs mention MNH outcomes less overall and with variability—maternal mortality are mentioned in all, but stillbirths are not mentioned in four countries and newborn mortality are not mentioned in one. The MNH mindset commonly reflects the continuum of care approach, with more attention to maternal interventions. Some MNH components are rarely mentioned across documents, such as abortion care, respectful maternity or family-centered care, breastfeeding, and small and sick newborn care. Results frameworks in most ICs have standard measures aligned to Every Newborn Action Plan/ Ending Preventable Maternal Mortality coverage targets; however, MNH measures reduce in most PADs, e.g., newborn health measures are not in four countries. Across all countries, PAD investments total US$1,552 million (US$266 million from GFF), and differing approaches to funding descriptions prevent meaningful comparisons of MNH-specific allocations.

**Conclusions**

While included in GFF country documents, MNH content fades from mentions to mindset, measures, and money, despite being a stated priority by the GFF. Major gaps exist, particularly for stillbirths and specific programmatic areas. The GFF is a great opportunity to make a catalytic difference, but it will need to address these gaps to deliver its goals. Disconnects between stated priorities for MNH in ICs and allocated resources in PADs suggest the need for further investigation.

## O114. Use of mHealth to strengthen and support Prevention of Mother-to-Child Transmission (PMTCT) service delivery uptake during the COVID-19 pandemic in Lesotho

### Pelaelo Motanyane, Rachel Chinyakata, Mamakamane Nyapisi, Mojalefa Mosoeu, Moeti Moleko, Nakululombe Kwendeni, Petronella Chirawu, Mpolokeng Mohloai

#### mothers2mothers

*BMC Proceedings 2024*, **18(5):**O114

Submission ID #: IMNHC1256

**Background**

The emergence of the COVID-19 pandemic challenged different sectors to face disrupted health services and come up with innovative ways to deliver services to clients through electronic systems. mothers2mothers (m2m) introduced peer support via a phone app (PvP) to monitor, support, and improve clients’ lives. m2m’s frontline workers were able to connect with their clients via phone. They used mHealth tools to improve access, retention, follow-up, and monitoring of clients even in remote and industrial areas. This analysis investigates the usefulness of technology during the COVID-19 pandemic in reaching m2m clients in order to retain them in care and monitor them while they are still at their homes.

**Methods**

Client reach was investigated using data obtained from the m2m DHIS2 tracker (PvP). A total of 30,394 antenatal (AN) and postnatal (PN) clients were enrolled and active in m2m care as of December 2020.

**Results**

In March 2020 to December 2020, when movement was restricted due to the COVID-19 pandemic, mentor mothers were able to reach 17,258 (57%) active clients using PvP. Out of those clients, 5,850 (34%) AN clients and 11,408 (66%) PN clients were reached at the height of movement restrictions. During this period, no interactions, community visits, or close contact were allowed; clients could only be reached through PvP. Clients were able to receive support, and m2m’s mandate to give psychosocial support to clients was still maintained and PBFW were supported. Currently, in the period after strict COVID-19 lockdowns, clients who are not routinely reached during community work (such as clients living in hard-to-reach places and industrial areas) are still reached through PvP. This is an indication that eService delivery still plays a pivotal role in maternal service delivery coverage and it can be adopted or integrated into a service delivery model.

**Conclusions**

eService or mHealth tools are an important way to complement face-to-face service delivery especially where distance or movement restrictions play a role in clients’ access to services and information. m2m’s eservice delivery is an essential part of supporting HIV prevention and retention in care.

## O115. Self-Applied Technique for Quality Health (SATH): an advocacy tool developed by care Nepal for improved maternal and child health services in Nepal

### Santa Kumar Dangol, Adweeti Nepal

#### CARE

*BMC Proceedings 2024*, **18(5):**O115

Submission ID #: IMNHC1249

**Background**

Women living in poor or marginalized communities often face barriers to accessing health information and services in Nepal. To address this equity gap in health, CARE Nepal introduced a social mapping tool, Self-Applied Technique for Quality Health (SATH), in health mothers’ groups. The approach aims to increase women’s access to and use of health services and information by making health mothers’ groups the primary generators of information and encouraging women to take action based on their learning. The tool has been used in more than 3,100 health mothers’ groups across 42 of 77 districts through various CARE Nepal projects.

**Methods**

This paper aims to explore the effectiveness of the SATH tool in enhancing women’s engagement, particularly those from poor and marginalized communities, to strengthen local health systems and improve maternal and child health. The analysis is based on the final evaluation reports of CARE projects, and a qualitative perspective of health workers, different stakeholders, clients, and CARE staff. More specifically, the paper discusses the effectiveness of the SATH tool in linking to the four major aspects of the health mothers’ group concept: (1) participation, (2) empowerment, (3) access to services, and (4) equity in health service use.

**Results**

The analysis found that the tool contributes significantly to strengthening community health systems through use by health mothers’ groups. Similarly, it supports demand generation and use of health services by facilitating advocacy and providing women with opportunities to discuss barriers to service use and solutions to address those barriers. Additionally, it provides a key pathway for community members to connect with health service providers, which improves access to services. Given these outcomes, the Government of Nepal adopted the tool in the Equity, Access and Utilization Program in 2021 for improving maternal and child health service coverage among marginalized communities and has now expanded it across the country.

**Conclusions**

Adoption of the SATH tool by the Government of Nepal indicates it is a promising approach to ensure access, equity, participation, and empowerment—principles of health mothers’ groups—to achieve positive maternal and child health outcomes in Nepal. However, it is critical to ensure availability of quality services for sustaining the outcomes.

## O116. Systematic, quality, on-the-job, peer-led clinical mentorship improves health worker knowledge, quality of RMNCAH Services, patient satisfaction, health service utilization, and positive health outcomes in rural Sierra Leone

### Marie Hallissey, Mustapha Kallon, Mohamed Eisa

#### GOAL Global

*BMC Proceedings 2024*, **18(5):**O116

Submission ID #: IMNHC1245

**Background**

Sierra Leone has one of the highest maternal and child mortality rates. Lack of quality along with disrupted delivery of reproductive, maternal, newborn, child, and adolescent health (RMNCAH) services have been identified as the main contributing factors. While the high rate of teenage pregnancy and delays in seeking care contribute to the problem, pregnant women often do not receive adequate treatment at health facilities. In 2017, 79% of maternal deaths occurred in a health facility, suggestive of poor quality of service delivery. Limited access to training, particularly for rural health workers, has been identified as a limiting factor to the delivery of quality health care in Sierra Leone.

**Methods**

Working with a district health team, GOAL, an International humanitarian response agency with headquarters in Ireland, US and UK, designed and piloted an innovative, decentralized, peer-led, on-the-job package of clinical mentorship to improve RMNCAH service quality at peripheral health units (PHUs) in rural Kenema. Chiefdom health supervisors and midwives were trained to provide mentoring at PHUs under their supervision. This approach was intended to integrate mentorship into mentors’ routine roles to enhance relevance, effectiveness, and sustainability.

**Results**

An evaluation provides strong evidence that the approach has contributed to improvements in the skills and capacity of mentees, as well as increased client satisfaction. The maternal and neonatal mortality ratio in project communities declined over the period and the health service utilization rate increased. Responses from stakeholders interviewed provide evidence that the project has also increased adoption of family planning and prompt referral to secondary facilities in cases of emergency.

**Conclusions**

The clinical knowledge and skills acquired by mentees because of the mentorship can translate into quality maternal and child health care if the various components of a quality maternal and child health program are incorporated into the intervention. District health management teams, with the support of GOAL, are designing a scale-up of this project using lessons learned from the pilot.

## O117. Prediluted-prefilled color-labeled medication syringes decrease time to administration and dosing error in neonatal critical care units

### Gul Ambreen, Kashif Hussain, Muhammad Sohail Salat, Ali Shabbir, Adnan Mirza

#### The Aga Khan University Hospital

*BMC Proceedings 2024*, **18(5):**O117

Submission ID #: IMNHC1243

**Background**

The incidence of errors associated with injectable medications is higher than other formulations. Studies suggest that half of all harmful medication errors originate during drug administration. Of those errors, about two-thirds involve injectables, and may result in potentially life-threatening outcomes, particularly for pediatric clients when dosing requires weight-based calculations. Novel medication delivery systems of ready-to-administer medications may reduce dosing errors significantly.

**Objective**

Our goal was to evaluate novel, prediluted and prefilled color-labeled medication syringes ready for infusion, compared with conventional medication administration, in pediatric and neonatal critical care units.

**Methods**

An inotropic agent, dopamine, was selected by the pharmacy and nursing team to be dispensed in ready-to-administer prefilled and labeled syringes prepared by the pharmacy instead of nurses in client care units. We performed a prospective crossover study in which neonatal and pediatric nurse teams were observed administering dopamine to 40 clients, using either prefilled, color-coded syringes (intervention) or conventional drug administration methods (control). Data were extracted by blinded-independent reviewers.

**Results**

The median time to delivery of all doses for the conventional and color-coded delivery groups were six minutes (95% CI:3, 7) and three minutes (95% CI:1,4), respectively (difference = 3 minutes; 95% CI:2, 6). With the conventional method, 45 doses were administered, with five critical dosing errors (11%); with the color-coded method, 50 doses were administered, with zero critical dosing errors (difference=11%; 95% CI:5%, 33%).

**Conclusions**

A novel color-coded, prefilled syringe decreased time to medication administration and significantly reduced critical dosing errors by pediatric and neonatal nurses. The results suggest expending the project to other medications which are diluted by nurses on critical care pediatric and neonatal units.

## O118. Decentralization works! Improving Antenatal Care (ANC) utilization and coverage of essential interventions in 368 health centers and health posts in Ethiopia

### Solomon Woldeamanuel

#### Jhpiego

*BMC Proceedings 2024*, **18(5):**O118

Submission ID #: IMNHC1242

**Background**

Maternal infection, anemia, and poor nutrition are important causes of low birthweight in newborns. The Enhancing Nutrition and Antenatal Infection Treatment for Maternal and Child Health project in Ethiopia aimed to increase newborn birthweight through improved utilization and quality of antenatal care (ANC) with a focus on early ANC enrollment and improved ANC nutrition counseling, anemia prevention, and point of care (POC) testing and management of maternal infections and anemia.

**Methods**

The project was implemented from September 2018 to March 2022 in 65 health centers and 303 health posts in two regions of Ethiopia, Amhara and Oromia. Project activities included strengthening capacity of ANC providers, including health extension workers; strengthening availability of POC diagnostic tests; and improving quality of ANC nutrition counseling and POC screening and management of anemia, syphilis, and asymptomatic bacteriuria. The project also supported introduction of community-level POC pregnancy testing and strengthened two-way referral between health posts and centers.

**Results**

The project measured several improvements in ANC use and quality in the 368 supported sites. The percent of women enrolling in ANC at gestational age less than 12 weeks increased from 6% to 37% and the percent of women receiving at least one ANC visit and four or more ANC visits increased from 74% to 91% and 44% to 57%, respectively. The percent of women attending ANC who received iron and folic acid (90+) supplementation and deworming increased from 16% to 78% and 5% to 64%, respectively. In the final 12 months of the project, 94%, 96%, and 100% of women received ANC POC testing for anemia, syphilis, and asymptomatic bacteriuria, respectively, as compared to zero percent at baseline. Sixty-three percent of women tested for pregnancy in the community had a positive test at less than 12 weeks gestation age.

**Conclusions**

Decentralization works to improve ANC use and coverage of essential interventions for reducing low birthweight. Bringing ANC and POC diagnostic testing closer to the community contributes to earlier enrollment and retention in ANC. Task shifting ANC POC testing to health extension workers in health posts improves early identification and management of maternal anemia and selected infections, benefitting women and their newborns.

## O119. Social norms around delays in disclosing pregnancy and implications for antenatal care initiation: a qualitative study among Ugandan women

### Hadija Haddy Nalubwama^1^, Alison El Ayadi^2^, Cynthia Harper^2^, Josaphat Byamugisha^3^, Dilys Walker^2^, Alexander Tsai^4^, Blake Erhardt-Ohren^5^, Umar Senoga^3^, James Moody^6^, Paul Krezanoski^2^, Carol Camlin^2^, Alison Comfort^2^

#### ^1^Makerere University College of Health Sciences; ^2^University of California, San Francisco; ^3^Makerere University; ^4^Massachusetts General Hospital; ^5^University of California, Berkeley; ^6^Duke University

*BMC Proceedings 2024*, **18(5):**O119

Submission ID #: IMNHC1230

**Background**

Delaying to disclose pregnancy to one’s social networks can reduce individuals’ ability to access social support, which facilitates perinatal care access. There are limited in-depth and contextualized data exploring social norms around delays in pregnancy disclosure, their reasons, and the potential impact on antenatal care seeking in Uganda. By better understanding the factors influencing this practice, we can account for this behavior when designing interventions to ensure pregnant individuals initiate antenatal care early enough in pregnancy to optimize health. Therefore, we collected qualitative data about antenatal care decision-making from Ugandan pregnant women at their first antenatal care visit and norms around disclosing pregnancy.

**Methods**

We conducted in-depth interviews in Luganda or English with 31 pregnant women of varying partnership status and parity attending their first antenatal care visit at Kawempe National Referral Hospital between August and October 2020. Transcribed and translated data were analyzed thematically using both deductive and inductive coding.

**Results**

Delaying pregnancy disclosure to social networks was common among study participants, with most preferring to delay sharing information until the second or third trimester. Common reasons for delayed pregnancy disclosure included fear of witchcraft or curses often associated with adverse maternal and neonatal health outcomes and concerns about gossip. Several participants anticipated stigma from family members, friends, and even neighbors for becoming pregnant due to social norms around unplanned pregnancy or judgment if involved in socially undesirable partnerships. Consequently, some participants confirmed failing to initiate antenatal care early in pregnancy due to delayed or nondisclosure of their pregnancy status to those in their social networks. Several said that delayed pregnancy disclosure reduced the financial and informational support they received from these individuals.

**Conclusions**

Pregnant individuals in this study in Uganda tended to delay disclosing pregnancies to their social networks until later in pregnancy. Delayed pregnancy disclosure contributes to late initiation of antenatal care, which impedes efforts to improve maternal and neonatal health. Targeted interventions to address the negative social perceptions and factors contributing to delayed pregnancy disclosure are important so that women are supported to access care during their pregnancies and to optimize maternal and neonatal outcomes.

## O120. Systematic review of national continuous professional development processes and systems for midwifery educators in Low- and Middle-Income Countries (LMICs)

### Duncan Shikuku^1^, McCauley McCauley^1^, Sarah Bar-Zeev^2^, Charles A Ameh^1^

#### ^1^Liverpool School of Tropical Medicine; ^2^United Nations Population Fund

*BMC Proceedings 2024*, **18(5):**O120

Submission ID #: IMNHC1227

**Background**

Midwifery educators play a critical role in strengthening the midwifery workforce in LMICs for achieving the maternal-newborn health Sustainable Development Goal targets. However, midwifery educator development is grossly under-invested in LMICs, with variation in quality, insufficient/poorly trained educators, limited scope of practice, weak regulation, and lack of opportunities for educators to update their knowledge or skills competencies. Participating in relevant continuous professional development (CPD) is a key strategy to ensure that midwifery educators maintain their competence for quality midwifery graduates and workforce. This systematic review describes the current approaches, content, and effectiveness of midwifery educator CPD programs in LMICs.

**Methods**

A structured search through EBSCO Discovery Services of electronic databases (MEDLINE, CINAHL, Global Health, PubMed) and Google Scholar was performed. Papers published in English between 2000 and 2021 on CPD for midwifery educators in LMICs were reviewed. A narrative synthesis and the Preferred Reporting Items for Systematic Reviews and Meta-Analyses (PRISMA) was used. The protocol was registered in PROSPERO.

**Results**

Twelve papers met inclusion criteria although many did not report on CPD directly but focused on midwifery educator development initiatives. No formal CPD program managed by a national regulator or international midwifery body was identified. Common CPD delivery approaches identified were twinning (international collaborations employing the train-the-trainer approach) and informal programs guided by an international medical body. Content included curriculum design and review, teaching, learning and assessment, leadership in health, project management and evaluation, and education research training. Programs contributed to personal development, professional growth, and health systems improvements in maternal-newborn health.

**Conclusions**

There is low evidence of effective midwifery educator development initiatives to maintain competence. There is a need to design regulator-accredited midwifery educator CPD programs to improve the quality of midwifery education and number of competent skilled health personnel in LMICs.

## O121. Predictors of respectful maternity care and influence of HIV status among women giving birth in Kilimanjaro, Tanzania

### Mariam Barabara^1^, Susanna Cohen^2^, Gileard Masenga^3^, Linda Minja^1^, Maya Stephens^2^, Pendo Mlay^3^, Gaudensia Olomi^4^, Janeth Mlay^1^, Virginie Marchand^5^, Anya Weglarz^2^, Olivia Hanson^2^, Blandina Mmbaga^3^, Melissa Watt^2^

#### ^1^Kilimanjaro Clinical Research Institute; ^2^University of Utah; ^3^Kilimanjaro Christian Medical Center; ^4^Kilimanjaro Regional Department of Health; ^5^Duke University

*BMC Proceedings 2024*, **18(5):**O121

Submission ID #: IMNHC1214

**Background**

Respectful maternity care (RMC) is a rights-based approach to childbirth that centers on the dignity, autonomy, and well-being of the birthing woman. This study aimed to examine factors associated with RMC among women giving birth in Tanzania, and to examine whether HIV status was associated with self-reported RMC.

**Methods**

Between March and July 2022, we enrolled 229 postpartum women in six clinics in Kilimanjaro Region, Tanzania. Participants completed a survey within 48 hours after birth, prior to being discharged. RMC was measured using a 30-item scale with three subscales (dignity and respect, supportive care, and communication and autonomy), each standardized at 0 – 100. Univariable and multivariable regression models examined factors associated with RMC.

**Results**

Participants’ self-reported RMC was generally positive. The median (Q1, Q3) of the full RMC score of participants was 74.4 (64.2, 83.3), and the subscales were as follows: dignity and respect, 83.3 (66.7, 94.4); supportive care, 75.6 (68.3, 84.4); and communication and autonomy, 66.7 (51.9, 77.8). RMC did not differ by HIV status (67 vs. 67, *p* = 0.889). In univariable analysis, illiteracy (*β* = −4.5; 95% CI: −8.1, −0.8), middle wealth quintile (*β* = −4.5; 95% CI: −8.7, −0.3), and delivering in a public health facility (*β* = −3.5; 95% CI: −6.6, −0.4) were associated with lower RMC scores. No factors remained significant after controlling for selected confounders in the final multivariable model.

**Conclusions**

The high levels of self-reported RMC in this population of postpartum women are encouraging and reflect client satisfaction with birthing services in the study facilities. The communication and autonomy subscale had the lowest rating, indicating the need for additional training in clinical empathy and consent. There are indications that RMC is lower among women with lower socioeconomic status (low literacy and wealth) and in public facilities. This may reflect implicit bias in clinical care, as well as burnout among providers in under-resourced clinical settings. Ongoing support and training of clinical providers to deliver evidence-based and respectful maternity care to all women is important to promote the best outcomes among birthing women.

## O122. Introduction of Heat-Stable Carbetocin (HSC) for improving the active management of the third stage of labor in Nigeria: Murtala Muhammad specialist Hospital Kano as a case study

### Hadiza Abubakar Salele^1^, Paulette Ibeka^1^, Olufunke Fasawe^1^, Uchenna Igbokwe^2^, Olatunde Amode^1^, Damilola Oyedele^1^, Omaye Negedu^1^

#### ^1^Clinton Health Access Initiative; ^2^Solina Center for International Development and Research

*BMC Proceedings 2024*, **18(5):**O122

Submission ID #: IMNHC1213

**Background**

Postpartum hemorrhage (PPH) contributes to about 29% of maternal deaths in Nigeria. PPH management is hindered by systemic barriers, including lack of appropriate storage and, consequently, low quality of uterotonics, especially oxytocin. HSC, a uterotonic recommended for PPH prevention, is non-inferior to oxytocin in preventing PPH in settings without optimal cold storage. The Smile for Mothers (SfM) Project, funded by MSD for mothers, supports the introduction of HSC in Kano, Lagos, and Niger states. We studied the introduction in Murtala Muhammad Specialist Hospital (MMSH), which has the highest volume of deliveries in Kano state and is an intervention site.

**Methods**

SfM supported the Kano State Ministry of Health in incorporating the latest World Health Organization recommendations on uterotonics into its existing PPH guidelines, including a new uterotonic option, HSC, for use in the public healthcare sector. Using the revised guidelines, 32 healthcare workers in MMSH Kano were trained in emergency obstetric and newborn care, and these healthcare workers subsequently trained and mentored an additional 78 healthcare workers. In addition, the project supported the state to update and disseminate its revised PPH prevention algorithms, which included HSC, to guide appropriate use and strengthen documentation of uterotonics use. Weekly monitoring visits were conducted to address gaps in health workers’ use of uterotonics and improve documentation.

**Results**

Of the 110 health care workers trained and mentored, 98 have safely administered HSC and demonstrated appropriate uterotonics use during childbirth. Since its introduction in June 2022, HSC has been issued to 70% (of 2,445) parturient women during active management of labor, with zero PPH cases reported in clients who received HSC. Additionally, only one adverse drug reaction has been observed from the administration of HSC, demonstrating a good safety profile.

**Conclusions**

HSC can potentially improve PPH prevention in resource-constrained settings, especially where the quality and storage of oxytocin are not guaranteed. The experience from MMSH shows that HSC can be safely introduced into the maternal health supply chain system, and health workers can acquire the knowledge and skills to administer HSC safely.

## O123. Feasibility and acceptability of integrating mobile phone sms into antenatal, maternity, postnatal, and preschool consultations to improve mothers’ access to health information in the COVID-19 context: cluster randomized controlled trial in Lubumbashi

### Abel Ntambue Mukengeshayi^1^, Angèle Musau Nkola^1^, Françoise Malonga Kaj^1^, Joris Michielsen^2^, Bart Criel^2^

#### ^1^Université de Lubumbashi; ^2^Institut of Tropical Medicine

*BMC Proceedings 2024*, **18(5):**O123

Submission ID #: IMNHC1208

**Background**

Our objective was to assess the feasibility and acceptability of integrating SMS into antenatal, maternity, postnatal, and preschool consultations to improve mothers’ access to health information in the context of COVID-19.

**Methods**

We conducted a cluster randomized controlled trial comparing the use of antenatal care in 10 health facilities that had integrated SMS into antenatal care to sensitize women on COVID-19 (Arm 1), with 10 others that had not integrated SMS for sensitization (Arm 2) in the Kenya health zone in Lubumbashi, Democratic Republic of the Congo. We included 400 pregnant women cared for by 10 maternity providers in each arm (800 pregnant women and 20 providers). To assess acceptability and feasibility, we compared the level of women’s knowledge of COVID-19 between the two arms. We compared the percentage of providers who sent the SMS and collected the opinions of women and providers on the use of SMS.

**Results**

The use of the usual SMS was unsuitable for sending messages of more than 100 words and resulted in an excessive workload for the nurses. We have created and integrated an elementary algorithm to contact women by pregnancy age group. This algorithm was adopted and used by 30%, 86.5%, and 100% of care providers, respectively, after the first, fourth, and sixth week of intervention. The latter reported that this algorithm made it possible to raise awareness more quickly and more regularly among mothers, and to interact more closely with them. These text messages were accepted by the mothers and enabled 63%, 85.5%, and 93% of the women, respectively, to respond to their next prenatal visit. They also reported that the text messages improved their essential family care practices and raised interest in health within the household.

**Conclusions**

The elementary algorithm has been accepted by mothers and adopted by health care providers to improve access to health information.

## O124. Knowledge and acceptance of family planning: a cluster randomized controlled trial of group antenatal care in Ghana

### Georgina Amankwah^1^, Ruth Zielinski^2^, Vida Ami Kukula^1^, Veronica Esinam Awo Apetorgbor^1^, Elizabeth Awini^1^, John Williams^1^, Nancy Lockhart^2^, Cheryl Moyer^2^, Jody Lori^2^

#### ^1^Ghana Health Service; ^2^University of Michigan

*BMC Proceedings 2024*, **18(5):**O124

Submission ID #: IMNHC1205

**Background**

More than 300,000 women die from complications related to pregnancy and childbirth each year. In areas where pregnancy is most risky, there is often low utilization of family planning (FP). Greater utilization of FP improves outcomes by preventing unplanned pregnancies and short pre-pregnancy intervals. Antenatal care (ANC) visits are an optimal time to discuss FP, but many women do not attend antenatal visits regularly and visits are often limited to risk assessment. Therefore, the purpose of this study was to test an innovative model of group ANC for pregnant women in Ghana and knowledge/acceptance of FP.

**Methods**

A study randomized by facilities (14) in eastern Ghana was conducted comparing group and routine ANC. Midwives at intervention sites were trained in facilitating group ANC, which included clinical assessment, education, and social support. Between July 2019 and November 2021, trained research assistants recruited women presenting for ANC at all sites and created groups by gestational age at intervention sites. Baseline data (T0) were collected with follow-up data collection at the third trimester (T1), and six weeks (T2), six months, and one year post-birth. The intervention consisted of eight group ANC meetings with the seventh meeting focused on FP. Results are presented comparing FP data across timepoints in both the intervention and control groups.

**Results**

Women (1,761 at T0 and 1,285 at T1) in both groups understood the importance of pregnancy spacing (>97%). Participants in group ANC had a greater increase in knowledge of FP methods (increase of mean = 2.2 [T0] to 3.9 [T1]) versus controls (mean = 2.01 [T0] to 2.37 [T1], *p* < 0.0001). The percent of women in group ANC intending to use FP increased from 40.5% at T0 to 61.0% at T1 then dropped slightly to 54.9% at T2 (six weeks post-birth), while the percentage in the control group remained unchanged, indicating a significant difference in the intervention group (*p* < 0.0001).

**Conclusions**

The uptake of FP methods is multifactorial and includes knowledge, access, and cultural factors. Group ANC has the potential to increase knowledge and acceptance of FP, which will, in turn, improve maternal and newborn outcomes globally.

## O125. Invisibility of neonatal deaths in the civil registration system: evidence from India

### Rakhi Dandona, Anilkumar Gopinathan Nair, Sibin George, Md Akbar, Siva Prasad, Arpita Paul

#### Public Health Foundation of India

*BMC Proceedings 2024*, **18(5):**O125

Submission ID #: IMNHC1195

**Background**

Birth and death registrations have seen improvements in India recently but they continue to be poor in the state of Bihar, which accounts for a significant proportion of neonatal mortality in India. We undertook an assessment to understand the current status of and barriers to improving the coverage of birth and neonatal death registration.

**Methods**

The survey was designed to capture a sample of 10,000 live births representative of rural Bihar between January 2019 and July 2020 using a multistage sampling strategy. For each identified birth, a detailed interview was conducted with the child’s mother or the most knowledgeable person to document the place of delivery, whether the birth or neonatal death was registered, and the availability of the respective certificate. For the births and deaths where a relevant certificate was not available, the reasons for nonavailability were documented. Data collection was completed between August 2020 and April 2021.

**Results**

Detailed interviews were available for 8,696 live births (82.9% participation) and 208 neonatal deaths (82% participation). Birth certificate availability was reported for 5,919 live births (68.8%; 95% CI 67.8–69.7), and this coverage was significantly higher for live births delivered at public sector facilities as compared with the private sector and home births. It was also significantly higher for girls than boys. The birth certificate coverage for live births who did not survive the neonatal period was 2.8 times lower (24.6%; 95% CI 17.6–33.3) than for those who survived the neonatal period. Overall, 48.1% of neonatal deaths were at home irrespective of place of delivery. None of the neonatal deaths had a death certificate. The most common reason provided was the baby died immediately after birth or too young (33.2%), followed by no need for it (42.8%) and did not think about it (25%). Three-fourths of the neonatal deaths had neither a birth nor a death registration certificate.

**Conclusions**

Of the three million live births annually in Bihar, only one in three children exist officially and none of the estimated 70,000 neonatal deaths will have death certificate. This has implications for tracking Sustainable Development Goals 2030 progress.

## O126. Understanding the influence of traditional, socio-cultural gender norms and practices on the uptake of maternal health services provided to women and young girls in Zambia’s Eastern and Southern Provinces

### Olatubosun Akinola^1^, Miyanda Maila^1^, Rabson Zimba^1^, Cindy Chirwa^1^, Mpala Nkonkamalimba^2^, Masauso Chirwa^3^, Hilda Shakwelele^1^

#### ^1^Clinton Health Access Initiative; ^2^Independent Consultant; ^3^University of Zambia

*BMC Proceedings 2024*, **18(5):**O126

Submission ID #: IMNHC1193

**Background**

Gender is a key driver of health outcomes for everyone—women, men, girls, and boys. Gender-related factors compounded by traditional, socio-cultural gender norms and practices can limit provision and uptake of maternal and newborn health (MNH) services that improve health outcomes. The extent to which these affect provision and uptake of health services remains unknown in Zambia’s Eastern and Southern provinces. A gender analysis study was conducted to identify and understand the effects of traditional, social, and cultural norms and religious beliefs on provision and uptake of MNH services.

**Methods**

The study was qualitative and used a gender analysis framework with four domains. This abstract focuses on the cultural norms and beliefs domain. Four districts were purposively sampled, and data were collected through desk reviews, 22 focus group discussions (women, men, girls, and boys), and 36 key informant interviews (civil society organizations and national and subnational participants within the Ministry of Health), and analyzed using NVivo software.

**Results**

The study established that traditional, religious, social, and cultural norms were affecting the uptake of MNH services. In Eastern province, women held strong beliefs that one is bound to miscarry a pregnancy if disclosed in the first trimester. Further, women believed taking traditional medicines provided a quick and smooth childbirth, and that traditional medicines would offer newborns similar protection as vaccines, resulting in shunning of immunization services. Other women preferred home delivery as it is perceived to preserve dignity and respect the demands of a marriage setup. Traditional initiation ceremonies (Chinamwali in Eastern province), used to teach and guide girls’ sexual orientation once they reach puberty, facilitate adolescent girls’ early sexual debut, exacerbating the prevalence of teenage pregnancies and increased fistula cases as girls are eager to practice the teachings. Lastly, the study found that religious beliefs had a negative effect on safe abortion as health care staff refused to provide services to women due to strong beliefs that abortion is a sin and murder.

**Conclusions**

The study revealed that religious, traditional, social, and cultural norms and beliefs were impacting provision and uptake of MNH services. These findings will facilitate design of culturally appropriate interventions for reproductive, maternal, newborn and child health in Zambia.

## P127. Preliminary effectiveness of an mHealth delivered health education and social support intervention in the postpartum period: maternal/infant nutrition

### Mona Duggal^1^, Garima Singh Verma^1^, Alison El Ayadi^2^, Rashmi Bagga^1^, Vaibhav Miglani^1^, Navneet Gill^1^, Naveen Mutyala^1^, Lakshmi Gopalakrishnan^2^, Pushpendra Singh^3^, Alka Ahuja^1^, Ankita Kankaria^4^, Vijay Kumar^5^, Nadia Diamond-Smith^2^

#### ^1^Postgraduate Institute of Medical Education and Research; ^2^University of California, San Francisco; ^3^Indraprastha Institute of Information Technology Delhi; ^4^AIIMS, Bhatinda; ^5^SWACH

*BMC Proceedings 2024*, **18(5):**P127

Submission ID #: IMNHC1189

**Background**

Providing information and social support after pregnancy can significantly impact the health of new mothers and their infants, including improving maternal nutrition and breastfeeding practices. In this paper, we evaluate the impact of a mobile health intervention for postpartum women to educate and strengthen social support on maternal and infant dietary practices (breastfeeding and complementary feeding).

**Methods**

The intervention lasted until six months postpartum and included weekly group calls and interactive voice response or app-based audios with education on various topics. The intervention was provided to 160 perinatal women in Mohali, Punjab, with 20 women as controls. Pre- and post-surveys were conducted to measure changes in knowledge and behaviors.

**Results**

There was a statistically significant increase in the proportion of women who responded correctly about the timing of introducing complementary foods for six out of nine food items. Endline comparisons between the groups found that women who received the intervention were more likely to have higher dietary diversity for themselves, and there was some evidence of longer breastfeeding duration.

**Conclusions**

These findings suggest that a low-cost, light touch, mHealth intervention not only increases knowledge but also impacts behavior related to maternal diet and potentially breastfeeding practices as well.

## O128. Impact of family care training for postnatal outcomes in India

### Seema Murthy, Shirley Yan, Sahana SD

#### Noora Health

*BMC Proceedings 2024*, **18(5):**O128

Submission ID #: IMNHC1188

**Background**

The Care Companion Program (CCP) is an in-hospital multitopic skills-based training program provided to families to improve postdischarge maternal and neonatal health. The structured program uses the existing health workforce—mainly nurses or health counselors—within the government health systems to give regular group training sessions while the mother is still in the hospital. The content and tools needed for this training are contextualized to specific geographies. Involving the entire family and including multiple families together is hypothesized to improve behavior uptake. The states of Punjab and Karnataka in India piloted the program in 11 district hospitals in July 2017, and we report results from the first evaluation of this program.

**Methods**

We compared self-reported maternal and neonatal care practices and health outcomes before and after the launch of the CCP program in 11 facilities. This data were collected at the end of the neonatal period over the phone, using structured questionnaires, for both groups. Families in the pre-intervention group delivered between May and June 2017 (*N* = 1,474), whereas those in the post-intervention group delivered between August and October 2017 (*N* = 3,510). Program effects were expressed as adjusted risk ratios obtained from logistic regression models.

**Results**

At two weeks post-delivery, the practice of dry cord care improved by 4% (RR = 1.04, 95% CI 1.02–1.06) and skin-to-skin care by 78% (RR = 1.78, 95% CI 1.37–2.27) in the post-intervention group as compared with pre-intervention group. Furthermore, newborn complications reduced by 16% (RR = 0.84, 95% CI 0.76–0.91), mother complications by 12% (RR = 0.88, 95% CI 0.79–0.97), and newborn readmissions by 56% (RR = 0.44, 95% CI 0.31–0.61). Outpatient visits increased by 27% (RR = 1.27, 95% CI 1.10–1.46). However, the practice of exclusive breastfeeding, unrestricted maternal diet, hand hygiene, and being instructed on warning signs were not statistically different.

**Conclusions**

Postnatal care should incorporate predischarge training for families. Our findings demonstrate that it is possible to improve maternal and neonatal care practices and outcomes through a family-centered program integrated into public health facilities in low and middle-income countries.

## O129. Evaluating pregnant women’s experiences with respectful maternity care in a midwifery-led antenatal care service in Karachi, Pakistan

### Nida Salman Yazdani^1^, Kaniz Amna Haider^2^, Amna Khan^2^, Ali Zaidi^2^, Uzma Khan^2^, Akbar Rajani^1^, Muhammad Imran Nisar^2^, Fyezah Jehan^2^, Zahra Hoodbhoy^2^

#### ^1^Vital Pakistan Trust; ^2^The Aga Khan University

*BMC Proceedings 2024*, **18(5):**O129

Submission ID #: IMNHC1174

**Background**

Client satisfaction is one of the most critical aspects for developing a healthy relationship between health care providers and their clients. In low- and middle-income countries where there is a shortage of care providers and access to tertiary facilities is challenging, midwives are the frontline care providers for pregnant women. Their experiences are key to the use or non-use of health care services. Hence, this study sought to understand pregnant women’s perceptions of the quality of antenatal care services provided by midwives at primary health centers in Karachi, Pakistan.

**Methods**

This was a cross-sectional survey conducted from January to December 2021 at Rehri Goth and Ibrahim Hyderi, two primary health centers located in peri-urban communities in Karachi, Pakistan. A structured questionnaire was administered to women in their third trimester to assess their perception of access to care, experience regarding the visit, person-centered approach, and general satisfaction with the facility. These themes were then mapped onto five of the seven universal Respectful Maternity Care Charter themes. Results for each of these themes (i.e., nonconsented, nonconfidential, nondignified, and denial or abandonment of care) were reported as frequencies and percentages.

**Results**

A total of 904 pregnant women participated in the survey. Women reported high satisfaction with operating hours (94%, *n* = 854) and cleanliness at the facility. The majority of the clients (>90%) reported highly on privacy, dignified care, and nondiscriminatory treatment by the midwives. However, only 60% (*n* = 542) of women reported informed consent before a procedure. Seventy-five percent (*n* = 677) of pregnant women reported high satisfaction with counseling related to a healthy pregnancy and 44% with nutritional counseling (*n* = 400), while only 13% (*n* = 120) of women were satisfied with sessions on birth preparedness.

**Conclusions**

The majority of pregnant women were satisfied with the facility’s ambience, respect, and care received. However, women reported dissatisfaction with certain pregnancy counseling services provided. Regular respectful maternity care and technical trainings would benefit midwives’ interactions with women and potentially lead to higher client satisfaction and improved maternal and newborn health outcomes.

## O130. Increase in the neonatal mortality rate during the COVID-19 pandemic: population-based evidence from India

### Anilkumar Gopinathan Nair, Sibin George, Md Akbar, Moutushi Majumder, Siva Prasad, Arpita Paul, Rakhi Dandona

#### Public Health Foundation of India

*BMC Proceedings 2024*, **18(5):**O130

Submission ID #: IMNHC1167

**Background**

We report on neonatal mortality rate (NMR) and cause of neonatal deaths during the COVID-19 pandemic, and the change in NMR between the pre-pandemic (2016) and pandemic periods from a population-based assessment in the Indian state of Bihar.

**Methods**

All live births that occurred between July 2020 and June 2021 were listed from 261,124 households (91.5% participation) representative of Bihar. Live births in the seven months with the most COVID-19-related deaths in India were considered as COVID-19 peak period births. Detailed interviews, including verbal autopsies, were conducted for all neonatal deaths and for a 25% random sample of all live births who survived the neonatal period. We estimated overall NMR, and separately for COVID-19 peak and non-peak periods per 1,000 live births. The change in NMR for Bihar from 2016 to 2020–2021 was estimated.

**Results**

We identified 831 neonatal deaths in 30,426 live births (91.5% participation) with an estimated NMR of 27.9 (95% CI 26.0–29.8) in 2020–2021. NMR was significantly higher for private facility (38.6; 95% CI 34.4–43.3) and home (36; 95% CI 31.5–41.1) deliveries than for public facility (20.4; 95% CI 18.4–22.7) deliveries. It was similar for COVID-19 peak (28.3; 95% CI 25.9–30.9) and non-peak (27.2; 95% CI 24.5-30.3) periods. Birth asphyxia and preterm delivery accounted for 35.8% and 35.7% of neonatal deaths followed by pneumonia (20.7%); this pattern varied by place of delivery and age at death, but not by COVID-19 peak and non-peak periods. A significant increase of 12.8% (95% CI 1.8–23.8) in overall NMR and 34.8% (95% CI 19.0–50.7%) in 0–2 days NMR was documented between 2016 and 2020–2021. A significant increase was seen in private facility births and decrease in public facility and home births over this period.

**Conclusions**

This study documented an increase in NMR during the pandemic as compared with the pre-pandemic period. The findings highlight the opportunities and challenges of private sector delivery of maternal and newborn care during the pandemic, and the need to monitor the shift in care seeking, which may be adding to the burden of adverse outcomes.

## O131. Malnutrition in infants aged under six months attending community health centers: a cross-sectional survey

### Melkamu Berhane Arefayine

#### Jimma University

*BMC Proceedings 2024*, **18(5):**O131

Submission ID #: IMNHC1163

**Background**

Poor understanding of the malnutrition burden is a common reason for not prioritizing the care of small and nutritionally at-risk infants aged under six months (hereafter infants) in low- and middle-income countries. This study aimed to estimate the prevalence of anthropometric deficit in infants attending health centers in the Oromia region, using the Composite Index of Anthropometric Failure (CIAF), and to assess the overlap of single indicators underweight and low mid-upper arm circumference (MUAC) with stunted, wasted, and CIAF prevalence.

**Methods**

We undertook a two-week-long survey of all infants visiting each of 18 health centers located in Ethiopia’s Oromia region. We measured weight, length, and MUAC; and calculated weight-for-length z-score (WLZ), length-for-age z-score (LAZ), and weight-for-age z-score (WAZ). We defined wasted, stunted, and underweight as WLZ, LAZ, and WAZ < −2, respectively; and low MUAC as MUAC < 11.0 cm if the infant is aged < six weeks and < 11.5 thenafter. We defined CIAF as any infant with WAZ, LAZ, or WLZ < −2.

**Results**

Overall, 21.7% (95% CI 19.2; 24.3) of infants presented CIAF and 10.7% (95% CI 8.93; 12.7) had multiple anthropometric deficits. Low MUAC overlapped with 47.5% (95% CI 38.0; 57.3), 43.8% (95% CI 34.9; 53.1), and 42.6% (95% CI 36.3; 49.2) of the stunted, wasted, and CIAF prevalence, respectively. Underweight overlapped with 63.4% (95% CI 53.6; 72.2), 52.7% (95% CI 43.4; 61.7), and 59.6% (95% CI 53.1; 65.9) of the stunted, wasted, and CIAF prevalence, respectively. Underweight and a low MUAC, as single indicators, appears to capture most infants that are concurrently wasted and stunted.

**Conclusions**

Anthropometric deficits, single and multiple, are prevalent in infants attending health centers in Ethiopia. To identify any form of anthropometric deficit, as classified by wasted, stunted, underweight, CIAF, or Composite Index of Severe Anthropometric Failure, WAZ appears to perform better than MUAC, while both are good at identifying infants under age six months with multiple anthropometric deficits. Further research is needed to understand which criterium or criteria would be best for future programs managing small and nutritionally at-risk infants under age six months and to understand the associated functional and clinical outcomes, notably short-term risks of mortality and morbidity.

## O132. Strengthening a referral system through a community-owned maternal and newborn transport model using motorbike ambulances in Luwingu, Lupososhi, and Lunte Districts, Northern Province, Zambia

### Caren Chizuni^1^, Aniset Kamanga^2^, Timothy Silweya^2^, Angel Mwiche^1^, Morrison Zulu^2^, Oluwaseun Aladesanmi^2^, Andrew Storey^2^, Hilda Shakwelele^2^, Margaret Prust^2^

#### ^1^Ministry of Health, Zambia; ^2^Clinton Health Access Initiative

*BMC Proceedings 2024*, **18(5):**O132

Submission ID #: IMNHC1153

**Background**

Less than 25% of Zambia’s population live within 15 km of an emergency obstetric facility, amplifying inequities for women accessing lifesaving care during pregnancy and delivery. In 2018, the Ministry of Health and Clinton Health Access Initiative implemented a community referral and transport intervention as part of the integrated reproductive, maternal, and neonatal health program in Northern province covering 143 health facilities. Northern province is sparsely populated, and characterized by long distances to health facilities, poor terrain, and inadequate public transport. There have been numerous community interventions to bridge gaps in maternal transport, but they are often not sustained beyond partner support.

**Methods**

To address these barriers, 20 community motorbike ambulances (MBAs) were deployed in Luwingu, Lupososhi, and Lunte districts to transport women and newborns to health facilities during pregnancy and delivery. MBAs were deployed using a model focused on community ownership and sustainability beyond partner support. Multiple engagements were conducted between district health teams, health workers, and communities on community-led funding and operating mechanisms. Forty volunteer riders were trained to operate the MBAs and perform basic maintenance and were equipped with protocols, pathways, and communication, linking riders with other community workers and health facilities. An oversight and accountability system involving surrounding health facilities and district health teams was implemented to monitor operations of the MBAs.

**Results**

Between December 2018 and December 2021, 4,509 women and 260 newborns were transported to health facilities using these MBAs. Reasons for maternal transport include hemorrhage, prolonged labor, seizures, and fever, while reasons for newborn transport include fever, difficulties in breathing, convulsions, and poor/no sucking. During the same period, health facilities surrounding these communities reported a 95% increase in antenatal care attendance and 25% increase in institutional deliveries. Following project completion, communities continue to fund MBA fueling, maintenance, and rider incentives and have expanded the scope of use to other emergencies.

**Conclusions**

Community-owned and managed motorbike ambulances can strengthen the referral system and increase access to care for women and newborns and serve as a model for sustainability beyond the life of program.

## O133. Stress outcomes of “Zero Separation” in the mother-newborn Dyad in neonatal intensive care units

### Leena Kaushik^1^, Kiran Nehra^1^, Nitya Wadhwa^2^, Tushar K Maiti^3^, Sugandha Arya^4^, Harish Chellani^5^

#### ^1^DCR University of Science & Technology; ^2^Translational Health Science and Technology Institute; ^3^Regional Centre for Biotechnology, India; ^4^VMMC & Safdarjung Hospital; ^5^Center for Health Research and Development: Society for Applied Studies

*BMC Proceedings 2024*, **18(5):**O133

Submission ID #: IMNHC1147

**Background**

Conventionally, small sick newborns are separated from their mothers for care in neonatal intensive care units (NICUs) globally. A recent trial showed that newborns provided continuous kangaroo mother care commenced immediately after birth with zero separation from the mother reduced neonatal mortality by 25%. We hypothesized that psychometric and biochemical stress would be lower among mother-newborn dyads roomed in together as compared to conventional care.

**Methods**

We conducted a cohort study in Safdarjung Hospital, Delhi, India, involving mothers and their preterm (gestational age 28 to < 37 weeks), low birthweight (birthweight < 1.8 kg) newborns, who after birth were either roomed in together in the mother-newborn care unit (MNCU) or provided conventional care with the mother in the postnatal ward and her newborn in the NICU. We measured the weight, length, and head circumference of the babies at birth and discharge from the MNCU or NICU. Saliva was collected from the mothers and their newborns at the time of admission and discharge. Psychometric stress was evaluated using the Edinburgh Postnatal Depression Scale (EPDS) and Premature Infant Pain Profile (PIPP) scale. Salivary cortisol reactivity for biochemical assessment of stress was measured using commercially available ELISA kits. The primary outcome was stress levels measured using the psychological instrument and the biochemical evaluation in the mother-newborn dyad.

**Results**

We enrolled 260 mother-newborn dyads from August 2020 to March 2021. There were 56 twins. Psychometric stress was defined as an EPDS score of >12 among mothers and a PIPP score of >6 in the neonates. Saliva samples were collected both at admission and discharge from 260 mothers and 100 newborns. There was a significant reduction (*p* < 0.001) in psychometric stress scores and salivary cortisol levels among mothers and their newborns who were admitted to MNCU as compared to the conventional care group admitted in NICU.

**Conclusions**

This study concluded that keeping mothers and newborns together with zero separation lowered stress among mothers and babies as cortisol levels were lower in the MNCU group. MNCU babies showed better weight gain.

## O134. Stillbirths: contribution of novel newborn types in 15 countries using 126,424,772 Births from nationwide records from 2000 to 2020

### Yemi Okwaraji^1^, Lorena Suarez-Idueta^2^, Eric Ohuma^1^, Hannah Blencowe^1^, Joy Lawn^1^, Vulnerable Newborn Measurement Collaborative Group^3^

#### ^1^London School of Hygiene & Tropical Medicine; ^2^Mexican Society of Public Health; ^3^Multiple Organizations

*BMC Proceedings 2024*, **18(5):**O134

Submission ID #: IMNHC1143

**Background**

Each year, an estimated two million babies are stillborn, using the World Health Organization definition for international comparison of ≥28 weeks gestation. Few studies have examined the differing risks for stillbirth based on combinations of gestational age (preterm [PT] vs. term [T]) and attained size for gestational age using INTERGROWTH-21st standards (i.e., small for gestational age [SGA], appropriate for gestational age [AGA], and large for gestational age [LGA]) in the same baby. In this study, as part of the Vulnerable Newborn Collaborative, we identified high-quality national stillbirth datasets and analyzed risks by gestational age and attained size for gestational age.

**Methods**

We included 15 national datasets from Latin America, Europe, North America, and Australia with 647,923 stillbirths beyond 22 weeks gestation and 126,424,772 total births (2000–2020). Stillbirths were categorized by gestational age groups—<28^+0^ weeks, 28^+0^–31^+6^ weeks, 32^+0^–33^+6^ weeks, 34^+0^–36^+6^ weeks, 37^+0^–41^+6^ weeks (reference), and ≥42^+0^ weeks—and six types—T+AGA, T+LGA, T+SGA, PT+LGA, PT+AGA, and PT+SGA. We described the distribution of the six types and calculated relative risk ratios for each group compared to T+AGA as a reference.

**Results**

Among 126,424,772 million pregnancies at risk, 647,923 (0.51%) resulted in stillbirths. The majority of stillbirths were preterm, including PT+AGA (48.1%) followed by PT+SGA (16.2%), T+AGA (16.6%), and PT+LGA (9.9%). Term stillbirths totaled fewer than 10% T+SGA (4.9%) and T+LGA (4.2%). The median relative risk of stillbirth was highest for those most preterm, with a dose-response by gestational age group (<28^+0^ weeks, 130.9; 28^+0^–31^+6^ weeks, 54.4; 32^+0^–33^+6^ weeks, 22.8, 34^+0^–36^+6^ weeks 6.6; and ≥42^+0^ weeks, 7.7). The median relative risk of stillbirth was 81.1 for PT+SGA, 26.1 for PT+AGA, 25.9 for PT+LGA, 5.3 for T+SGA, and 0.9 for T+LGA, compared to T+AGA as a reference.

**Conclusions**

In these high-quality data from high-/middle-income countries, more than half of stillbirths were preterm, and stillbirth relative risk was steeply associated with lower gestational age. More analyses are needed using high-quality datasets from low-income settings, especially with higher rates of SGA. Further analyses are needed to better understand patterns of gestation-specific risk in these populations, and also patterns in lower-income contexts, especially those with higher rates of intrapartum stillbirth and SGA

## O135. Associations between maternal well-being and infant growth status: a cross-sectional study

### Mubarek Abera^1^, Tsinuel Girma^1^, Alemseged Abdissa^2^, Kiddus Yitbarek^1^, Nahom Abate^3^, Hatty Barthorp^3^, Marie McGrath^4^, Emma Beaumont^5^, Carlos Grijalva-Eternod^5^, Marko Kerac^5^

#### ^1^Jimma University; ^2^The Armauer Hansen Research Institute; ^3^GOAL Global; ^4^Emergency Nutrition Network; ^5^London School of Hygiene & Tropical Medicine

*BMC Proceedings 2024*, **18(5):**O135

Submission ID #: IMNHC1137

**Background**

Both poor maternal well-being (MW) and child stunting are unacceptably high in many low- and middle-income countries. Links between maternal well-being and child nutritional status are plausible but evidence is sparse, especially in infants under age six months. We thus aimed to explore the associations between these variables in a cross-sectional survey.

**Methods**

We conducted a health facility-based cross-sectional study of 1,060 infants in Jimma zone and Deder district, Ethiopia, between October 12, 2020, and January 29, 2021. The main exposure was MW, score of 0 for good MW and ≥1 for risk of depression on the Patient Health Questionnaire (PHQ-9), and the outcome variable was infant nutritional status: length-for-age Z-score (LAZ), weight-for-age Z-score (WAZ), weight-for-length Z-score (WLZ), body mass index Z-score (BMIZ), and mid-upper arm circumference (MUAC). Findings from a linear regression analysis adjusted for potential confounders were reported.

**Results**

Mean (standard deviation) for infant age was 13.3 (6.3) weeks, LAZ −0.5 (1.4), WAZ −0.8 (1.3), WLZ −0.5 (1.3), BMIZ −0.7 (1.3), and MUAC 124.2 (12.9) mm. Poor MW was independently associated with infant LAZ (β = −0.24, 95% CI −0.45, −0.04), but not with other parameters. Other factors associated with higher LAZ score were female infant sex (β = 0.22, 95% CI 0.02, 0.41), exclusive breastfeeding (β = 0.2, 95% CI 0.001, 0.40), higher wealth index (β = 0.08, 95% CI 0.02, 0.14), and higher maternal age (β = −0.04, 95% CI −0.07, −0.01). Comparing the strength of associations, the estimate from MW is highest. In a separate regression model, we checked if MW was actually accounted for/explained by family wealth status, and confirmed that there is no association between the two exposure variables.

**Conclusions**

MW is associated with infant linear growth. MW is a key component of an integrated care pathway for small and nutritionally at-risk infants under age six months (MAMI Care Pathway Package) being tested in a randomized controlled trial in Ethiopia. Our findings indicate the potential for impact on stunting reduction, a critical strategic commitment of the Ethiopian government (Seqota Declaration).

## O136. Strengthening management of emergency maternal health complications in the emergency units of public health facilities in Bangladesh

### Rafiul Alam^1^, Zubair Shams^2^, Bal Ram Bhui^2^, ASM Moniruzzaman^1^, Afsana Karim^2^, Joseph De Graft-Johnson^2^

#### ^1^Jhpiego; ^2^Save the Children

*BMC Proceedings 2024*, **18(5):**O136

Submission ID #: IMNHC1134

**Background**

In Bangladesh, ante- and postpartum hemorrhage (APH, PPH) and preeclampsia/eclampsia (PE/E) contributed about 55% to overall maternal deaths (Bangladesh Maternal Mortality and Health Care Survey, 2016). Improved detection and management of maternal complications is crucial to avert these preventable deaths. The U.S. Agency for International Development’s MaMoni Maternal and Newborn Care Strengthening Project supported district- and subdistrict-level hospitals with the formation and training of emergency management teams and the availability of essential drugs through the government system to manage maternal complications in the emergency department. After initial stabilization, mothers with complications are referred to inpatient wards of hospitals or to other facilities for further management. Ongoing supervision helped to resolve technical and logistical issues and strengthen recording and reporting. There were no pre-referral case management and reporting systems in the emergency units prior to this intervention.

**Methods**

This study examined the number and percentage of maternal complications managed and referred out by comparing two quarters six months apart—the baseline period October–December 2021 and the post-intervention period of April–June 2022—through the project-initiated supplemental record and reporting system for two key obstetric complications (PE/E, hemorrhage) reported in the emergency units of 14 district-level and 101 subdistrict-level hospitals across the project’s intervention areas.

**Results**

At baseline, of the 1,864 (615 PE/E, 1,249 hemorrhage) complication cases identified from emergency service units, 74% were admitted to inpatient wards, and the remaining 26% were referred out. At six months post-intervention, of the 1,605 (523 PE/E, 1,082 hemorrhage) complication cases, 80% were admitted to inpatient wards, and 12% were referred out. Furthermore, 5% of cases were managed in the emergency units and sent home.

**Conclusions**

Enabling emergency units to receive and triage maternal health complications leads to prompt and better care for women. All hospitals in Bangladesh should consider having the emergency units able to improve care and survival for women with maternal complications.

## O137. Case study on delivering nurturing care during Antenatal Care (ANC) and Postnatal Care (PNC) visits in Indonesia

### Romilla Karnati^1^, Ester Lucia Hutabarat^2^

#### ^1^Save the Children; ^2^Jhpiego

*BMC Proceedings 2024*, **18(5):**O137

Submission ID #: IMNHC1133

**Background**

The Nurturing Care Framework, launched in 2018, is a guide for multisectoral actors on improving early childhood development outcomes by supporting caregivers to provide nurturing care for newborns and young children, with a focus on the first 1,000 days when a child’s brain is developing rapidly.

In Indonesia, 45% of children under age 5 years are at risk of poor health, growth, and development. Although the Government of Indonesia has issued a policy, Holistic and Integrative Early Childhood Education and Development (HI-ECD), to promote comprehensive quality services for young children from survival to development, the policy practices are still dominated by interventions for children aged 4–6 years old, while interventions for the 0–3 age group remain limited. To address this gap, MOMENTUM Country and Global Leadership initiated a case study to examine the needs and opportunities of delivering nurturing care during antenatal and postnatal care visits as well as other health service touchpoints at the community level.

**Methods**

This case study collected information from three villages in Manggarai Barat District, representing urban, semi-urban, and remote-rural. Interviews, focus group discussions, and observations were done with caregivers and frontline health workers. Policy perspectives were gathered from village chiefs and district, provincial, and national stakeholders. The qualitative approach examined the enabling environment for nurturing care and current counseling practices, including the use of the maternal and newborn health (MNH) handbook to deliver nurturing care during visits to puskemas and posyandus.

**Results**

Preliminary findings suggest inadequate knowledge among frontline health workers to counsel on responsive care and early learning and limited training and use of the MNH handbook. Particularly, at the village level, health workers’ knowledge of child development was limited to nutrition and growth with little to no knowledge of responsive care and early learning aspects of nurturing care. Furthermore, maternity classes during antenatal care can be used to counsel on nurturing care.

**Conclusions**

Findings propose stronger advocacy and support for HI-ECD coordinating mechanisms at the local level, expansion of maternal, newborn, and child health interventions to include children’s growth and development and promoting the HI-ECD service package for children aged 0–3 years old.

## O138. Mycoplasma genitalium and other reproductive tract infections in pregnancy associated with an increased risk of adverse birth outcomes in a high-burden setting

### Michelle Scoullar^1^, Philippe Boeuf^1^, Elizabeth Peach^1^, Ruth Fidelis^1^, Pele Melepia^1^, Catriona Bradshaw^2^, Arthur Elijah^3^, Andrew Vallely^4^, Lisa Vallely^4^, Freya Fowkes^1^, William Pomat^5^, Brendan Crabb^1^, Christopher Morgan^6^, James Beeson^1^

#### ^1^Burnet Institute; ^2^Monash University; ^3^University of Papua New Guinea; ^4^Kirby Institute; ^5^Papua New Guinea Institute of Medical Research; ^6^Jhpiego

*BMC Proceedings 2024*, **18(5):**O138

Submission ID #: IMNHC1128

**Background**

Being born too small is a major risk factor for neonatal and infant mortality and childhood stunting. Sexually transmitted infections (STIs) are preventable and curable infections that can contribute to poor pregnancy outcomes. *Mycoplasma genitalium* is increasingly recognized as an STI with high prevalence in some populations and has a high propensity to develop antimicrobial resistance. Little data are available on the impact of M. *genitalium* on adverse birth outcomes, particularly in the context of other STIs. In Papua New Guinea, we previously demonstrated a high burden of multiple STIs (M. *genitalium*, *Chlamydia trachomatis*, *Neisseria gonorrhoeae*, *Trichomonas vaginalis*, and bacterial vaginosis). Here, we have investigated the association between M. *genitalium* and other STIs and birthweight, preterm birth, and perinatal mortality.

**Methods**

Six hundred and ninety-nine pregnant women in rural and semi-urban Papua New Guinea were recruited at their first antenatal clinic and followed to evaluate birth outcomes. Fundal height was used for gestational age estimates as per national guidelines. Vaginal swabs were tested for M. *genitalium*, C. *trachomatis*, N. *gonorrhoeae*, and T. *vaginalis*, and a vaginal smear was examined for bacterial vaginosis. Linear and logistic regression models examined the association between STIs and adverse birth outcomes.

**Results**

Many women experienced adverse birth outcomes, with 11.5% of babies under 2.5 kgs, a stillbirth rate of 21/1,000 pregnancies, and 23% (123/535) of babies were preterm. In multivariable analysis, bacterial STIs (M. *genitalium*, C. *trachomatis*, and N. *gonorrhoeae*) were all associated with decreased mean birthweight with varying effect sizes. There was some association between bacterial vaginosis and an increase in mean birthweight.

**Conclusions**

To our knowledge, this is the first study to demonstrate an association between M. genitalium and birthweight whilst adjusting for other STIs, highlighting the negative impact of bacterial STIs in pregnancy. Prevention is an urgent priority and increasing access to prompt diagnosis and treatment in pre-pregnancy and early in pregnancy would likely have important benefits for women. Detailed results will be published shortly.

## O139. Management of noncommunicable diseases in the context of pregnancy, childbirth, and postnatal care

### Joshua Vogel^1^, Tabassum Firoz^2^, Beth Pineles^3^, Jenny Jung^1^, Eshreena Karwal^4^, Nishika Navrange^5^, Steve McDonald^4^, Tari Turner^4^, Doris Chou^6^

#### ^1^Burnet Institute; ^2^Yale New Haven Health; ^3^University of Texas Health Science Center at Houston; ^4^Monash University; ^5^New York University; ^6^World Health Organization

*BMC Proceedings 2024*, **18(5):**O139

Submission ID #: IMNHC1116

**Background**

The World Health Organization is in the process of developing global guidelines for the management of noncommunicable diseases (NCDs) during pregnancy, childbirth, and postnatal care by focusing on priority conditions that are leading causes of maternal mortality and morbidity. The prioritization process included complementary reviews on NCDs to (1) understand how NCDs are defined/operationalized and (2) map existing guidelines and their recommendations for management of NCDs.

**Methods**

Two scoping reviews were conducted. The first review searched 12 databases and relevant websites for sources providing contextual information on NCDs published from 2000 onward and used a charting approach to synthesize the data. The second review searched four databases and 165 relevant websites for guidelines providing recommendations on management of NCDs published from 2011 to 2021. The characteristics of included guidelines and recommendations pertaining to management of NCDs were analyzed and mapped.

**Results**

In the first review, 172 peer-reviewed papers and 15 grey literature sources were included. Seven sources defined NCDs. NCDs were often defined as chronic but with varying temporality. A broad spectrum of conditions were included as NCDs, including pregnancy-specific conditions and infectious diseases. Most publications were from academic institutions in high-income countries and focus predominantly on the preconception and antepartum periods.

In the second review, we identified 6,026 articles, of which 405 guidelines were included. Current patterns of guidance were identified: recommendations largely focused on antenatal care, with few addressing intrapartum and postnatal care. There was a lack of guidance focusing on NCDs as a holistic topic. More than 90% of guidelines were published from high-income countries.

**Conclusions**

We found that there is a need for standardizing the definition of NCDs in the maternal health setting. Future guidelines should take a comprehensive, client-centered approach that addresses NCD multimorbidity and shared risk factors to improve pregnancy outcomes and reduce preventable morbidity and mortality. Guidelines specific to resource-limited settings and those focusing on postpartum care are needed. As part of the World Health Organization guideline development process, attention to equity and inclusion, and inclusion of the input and perspectives of low-resource settings will be critical.

## O140. Adaptation of the group antenatal care model to the Indian setting: lessons learned from Rajasthan, India

### ChandraShekhar Joshi, Ghanshyam Goklani, Gulnoza Usmanova, Yashpal Jain, Pankaj Grover, Dande Sireesha, Deepchand Jangid, Vinod Sain, Shiv Pratap Singh

#### Jhpiego

*BMC Proceedings 2024*, **18(5):**O140

Submission ID #: IMNHC1115

**Background**

Antenatal care (ANC) is an essential bundle of services to identify high-risk conditions. Despite increased use, ANC quality in India is known to be suboptimal. Group ANC has been suggested in the context of rigorous research (World Health Organization, 2016) as a novel way to encourage ANC attendance in groups that also promotes empowerment, peer interaction, and health literacy. In this paper, we report on the process undertaken to contextualize and adapt two group ANC models in Rajasthan and explore the feasibility and acceptability of a heterogeneous model where pregnant women of different gestational ages and a homogeneous model where women with similar gestational ages are grouped together.

**Methods**

The adaptation process included conceptualization, preparation, implementation, and adjustment phases. Two group ANC models were piloted in eight sites during March 2019–2020: the heterogenous model in two subcenters and the homogenous model in one primary and one subdivisional health center. Women were enrolled and group ANC sessions were conducted by health care providers. We assessed enrollment numbers, attendance records, and mean group strength and collected feedback from health workers and clients to understand the feasibility and acceptance of the two models.

**Results**

We designed a contextualized implementation package including a facilitator’s guide, meeting, and self-assessment cards. Health workers were trained and continuous mentorship was provided. Feedback from pregnant women and health workers was incorporated to better tailor our interventions. We found that the heterogeneous model had better attendance, adherence, and mean strength at 81%, 87%, and 9 compared to the homogeneous model at 51%, 59%, and 6.

**Conclusions**

Based on adherence and attendance data and field insights from time spent by women and interest and feedback from health workers and clients, the heterogeneous group ANC model appears to be most acceptable and feasible. Scaling up this model is likely to promote a more holistic women-centered maternal and newborn health care pathway. We found several challenges with the homogenous model, such as difficulty in forming groups and lower rates of attendance and adherence, however, further research is needed in settings where women are likely to receive all ANC care in one facility.

## O141. Zero separation of mothers and newborns in the newborn intensive care unit and physiological stabilization and sleep state of newborns

### Suruchi^1^, Sugandha Arya^1^, Harish Chellani^2^

#### ^1^VMMC & Safdarjung Hospital; ^2^Center for Health Research and Development:Society for Applied Studies

*BMC Proceedings 2024*, **18(5):**O141

Submission ID #: IMNHC1103

**Background**

A multicountry randomized controlled trial conducted in five countries (India, Ghana, Tanzania, Nigeria, and Malawi) coordinated by the World Health Organization (iKMC study) showed a 25% reduction in mortality by immediate kangaroo mother care (iKMC) in neonates with birthweight 1 to <1.8 kg. To implement the iKMC intervention, mother and baby need to be together continuously, which led to the concept of the mother-newborn care unit (MNCU). An MNCU is a facility where sick and small newborns are cared for with their mothers 24x7, with all facilities of level 2 newborn care and provision of postnatal care to mothers.

The neuroscience of zero separation suggests that neonates in skin-to-skin contact have better physiological stabilization and sleep state. Therefore, this study was planned to assess the physiological parameters and sleep state among low birthweight babies following iKMC in an MNCU.

**Methods**

In MNCUs, neonates receive KMC for 16 to 18 hours per day. The physiological parameters (heart rate, respiratory rate, oxygen saturation [SpO2]) and sleep state of low birthweight babies (weight 1 to 1.8 kg) admitted to the MNCU were recorded in 130 neonates before initiating iKMC and one hour after iKMC. Sleep state was assessed using the Neonates Sleep State Assessment Observational Checklist.

**Results**

The mean heart rate (per minute) pre-KMC was 141.3 ± 7.4 and post-KMC was 137.8 ± 6.9. The mean respiratory rate (per minute) pre-KMC was 42.6 ± 3.5 and post-KMC was 40.9 ± 3.0. The mean SpO2 (%) pre-KMC was 96.6 ± 1.1 and post-KMC was 97.7 ± 0.9. The mean temperature (°C) pre-KMC was 36.7 ± 0.2 and post-KMC was 36.9 ± 0.1. This change in all physiological parameters was statistically significant (*p* < 0.001). The mean sleep state score pre-KMC was 3.3 ± 1.0 and post-KMC was 2.3 ± 0.9. This change in sleep state was also statistically significant (*p* < 0.001).

**Conclusions**

iKMC in MNCUs leads to better physiological stabilization (post-KMC lower heart rate and respiratory rate, higher oxygen saturation, and higher temperature) and sleep state in sick low birthweight neonates.

## O142. Changes in maternal health service provision during the COVID-19 pandemic at the community health center (Puskesmas) level in Indonesia

### Herwansyah Herwansyah^1^, Katarzyna Czabanowska^1^, Peter Schröder Bäck^2^, Stavroula Kalaitzi^3^

#### ^1^Care and Public Health Research Institute Maastricht University; ^2^University of Applied Sciences for Police and Public Administration in North Rhine-Westphalia; ^3^Richard M.Fairbanks School of Public Health, Indiana University

*BMC Proceedings 2024*, **18(5):**O142

Submission ID #: IMNHC1101

**Background**

Providing comprehensive maternal health services at the community health centre (puskesmas) level is considered one of the essential efforts to reduce maternal mortality and improve the quality of women’s health. In Indonesia, puskesmas play an important role in providing primary healthcare for the community. Puskesmas is known as the first entry point to the healthcare system in the country. Therefore, public health-based interventions are necessarily required to address maternal health issues. However, the global COVID-19 pandemic has brought many changes to public health service provision, including maternal health services.

**Methods**

This study describes the use of “the most significant change” (MSC) technique to evaluate the impact of the COVID-19 pandemic on the provision of maternal health services from the healthcare provider perspective. We conducted interviews with 10 midwives and five heads of puskesmas focused on domains of change in maternal health services at five puskesmas capable of PONED, which provide basic emergency obstetric neonatal services in the Municipality of Sungai Penuh, Indonesia. Stories were analyzed qualitatively for content related to the domain of changes observed.

**Results**

The stories of midwives who routinely provide regular maternal health services were selected as the stories with most significant change. Their stories highlighted changes in the provision of maternal health services during the pandemic. They expressed that these changes affected the quality, standard operating procedure and number of visits as well as the services provided to the community.

**Conclusions**

In conclusion, the MSC technique enables policymakers to view raw data and evaluate the impact of the COVID-19 pandemic on maternal health service provision at primary healthcare settings from healthcare professionals’ perspectives. In addition, policymakers are able to develop future improved maternal health programs based on the experiences of healthcare providers. The application of the MSC technique is limited to maternal health service provision programs at the puskesmas level in the selected research sites, and no similar studies have been published in other academic journals.

## O143. Using a combination of high-impact interventions to improve maternal health outcomes: results from Mara Region, Tanzania

### Godlisten Martin^1^, Alice Christensen^1^, Joseph Massenga^1^, Lusekelo Njonge^1^, Rita Noronha^1^, Juma Mfanga^2^, Leah Daniel^2^, Ahmad Mohamed Makuwani^2^, Ntuli Kapologwe^3^, Defa Wane^4^, Miriam Kombe^4^

#### ^1^Jhpiego; ^2^Ministry of Health, Tanzania; ^3^Tanzania Office of the President of the Regional Administration and Local Government; ^4^United States Agency for International Development

*BMC Proceedings 2024*, **18(5):**O143

Submission ID #: IMNHC1097

**Background**

According to the Tanzania Health and Demographic Survey 2015–2016, Tanzania’s maternal mortality ratio had increased to 556/100,000 live births, missing Millennium Development Goal 4. Tanzania’s Mara region reported 66 maternal deaths out of 49,800 deliveries in 2015, with only 10% of antenatal care attendance before 12 weeks and 43% of mothers with four or more antenatal care visits. The average rate of institutional delivery was only 50%. Research shows that quality antenatal care visits with timely identification of risk factors and pregnancy-induced complications reduce maternal morbidity and mortality.

**Methods**

The U.S. Agency for International Development Boresha Afya Lake and Western project conducted a root cause analysis showing that maternal deaths were related to a lack of identification of complication risk factors during antenatal care, poor management of labor and obstetric emergencies, and poor monitoring of postnatal mothers. Low rates of utilization of facility-based maternal services were due to poor community involvement, leading to low demand for services.

The project, alongside Mara’s local government, introduced a combination of high-impact interventions to improve maternal outcomes from 2015 to 2020. To increase community involvement, the project worked with civil society organizations to strengthen demand creation by engaging community health workers and using community scorecards, which allowed the community to provide feedback and led to improved quality and use of maternal health services. At the facility level, to improve critical skills identified in the root cause analysis, the project used on-the-job, low-dose/high-frequency training that focused on improving the quality of antenatal care, routine and emergency childbirth, and postnatal care.

**Results**

The use of maternal services increased for both antenatal care before 12 weeks and four or more antenatal care visits from 10% in 2015 to 35% in 2020 and from 43% in 2015 to 91% in 2020, respectively. Institutional deliveries also increased, from 49,800 (69%) in 2015 to 88,046 (82%) in 2020. The number of maternal deaths decreased from 66 (2015) to 56 (2020).

**Conclusions**

Identifying root causes and then implementing well-coordinated high-impact maternal interventions improved the quality and use of maternal health services in the Mara region, which resulted in the reduction of maternal deaths

## O144. Mother-Newborn Care Unit (MNCU): family perception of quality of care

### Rashmi Dandriyal^1^, Harish Chellani^2^, Pratima Anand^1^, Sugandha Arya^1^

#### ^1^VMMC & Safdarjung Hospital; ^2^Center for Health Research and Development: Society for Applied Studies

*BMC Proceedings 2024*, **18(5):**O144

Submission ID #: IMNHC1086

**Background**

A multicountry randomized controlled trial conducted in five countries (India, Ghana, Tanzania, Nigeria, and Malawi) coordinated by the World Health Organization (iKMC study) from November 2017 to January 2020 showed a 25% reduction in mortality by immediate kangaroo mother care (iKMC) in neonates with birthweight 1 to <1.8 kg. This study was published in the *New England Journal of Medicine* (May 2021). To implement the iKMC intervention, mother and baby need to be together continuously, which led to the concept of the mother-newborn care unit (MNCU).

Admission of a baby to the neonatal intensive care unit (NICU) is a stressful experience for mothers. The presence of the mother with her sick baby in the NICU can improve maternal satisfaction and lead to better neonatal outcomes. The present study was conducted to evaluate the family-centered neonatal intensive care in the MNCU in a tertiary care hospital as perceived by mothers.

**Methods**

A tertiary hospital-based cross-sectional study was conducted over an 18-month period. Mothers whose newborns were admitted to the MNCU and conventional NICU were enrolled. A structured questionnaire was presented to mothers at discharge and responses were recorded in different domains and scaled from 1 to 4 (worst to best). Three hundred mothers were enrolled in each group.

**Results**

Information and education about neonatal care were rated as good by 89.57% of MNCU mothers and 81.4% of NICU mothers. In addition, 84.7% of MNCU mothers and 62.3% of NICU mothers judged sufficient parental involvement in newborn care, and 89% of MNCU mothers and 81% of NICU mothers were satisfied with their providers’ performance. Interpersonal relationships with health care workers were rated as good by 77.6% of MNCU mothers and 39% of NICU mothers. The physical environment was rated as satisfactory by 98.3% of MNCU mothers and 95% of NICU mothers, and 99% of MNCU mothers and 70% of NICU mothers were satisfied that physicians educated parents about postdischarge care, danger signs, and follow-up advice.

**Conclusions**

An MNCU facility has the potential to reduce communication gaps between parents and health care providers, improve maternal knowledge of neonatal care, and better prepare mothers and families for hospital discharge.

## O145. Establishing an integrated human Milk Banking model for feeding preterm, small, and sick newborns: the Kenya experience

### Kiersten Israel-Ballard^1^, Kimberly Mansen^1^, Elizabeth Kimani-Murage^2^, Taddese Zerfu^2^, Milka Wanjohi^2^, Calistus Wilunda^2^, Emily Njuguna^1^, Mary Waiyego^3^, Faith Njeru^3^, Laura Kiige^4^, Nelson Langat^2^, Betty Samburu^5^, Thomas Ngwiri^6^, Waithera Mirie^7^, Rachel Musoke^7^, Minnie Kibor^8^

#### ^1^PATH; ^2^African Population and Health Research Center; ^3^Pumwani Maternity Hospital; ^4^United Nations Children’s Fund; ^5^Kenya Ministry of Health; ^6^Gertrude’s Children’s Hospital; ^7^University of Nairobi; ^8^Children’s Investment Fund Foundation

*BMC Proceedings 2024*, **18(5)**:O145

Submission ID #: IMNHC1072

**Background**

The World Health Organization recommends donor human milk (DHM) from a human milk bank (HMB) for small and sick newborns (SSNB) when the mother’s own milk is not available. Global implementation guidelines, especially for low- and middle-income country (LMIC) settings, do not exist, making HMB expansion a challenge. The Kenya Ministry of Health sought PATH’s technical assistance to pilot the Mother Baby Friendly Initiative Plus (MBFI+) integrated HMB model with the objective of developing an LMIC-appropriate model for providing DHM and enhanced lactation support to improve exclusive human milk diets for SSNB and reduce neonatal mortality.

**Methods**

A validated phased approach was used, with leadership from the county, hospital, and Ministry of Health, to establish a Kenya-specific MBFI+ model for HMB, integrated with breastfeeding support and specialized lactation for prioritizing mothers’ own milk at Pumwani Maternity Hospital in Nairobi County, Kenya. This five-year process included three phases:

- Phase 1: Learning and building technical competency in the community through formative assessments to determine acceptability and facility readiness, learning exchanges to Vietnam and South Africa, guidelines and standard operating procedures development, quality control, and HMB trainings.
- Phase 2: Implementing the pilot program through facility and equipment improvements, capacity strengthening, coaching, and quality assurance.
- Phase 3: Research and stabilization through baseline and endline assessments to determine impact.

**Results**

The first HMB in Kenya launched in March 2019 and has remained operational, overseen by the county and Ministry of Health. Integrating the HMB with other breastfeeding support services, such as kangaroo mother care, was key in ensuring effectiveness and sustainability, as well as the appropriate use of DHM. Strong government commitment to protect, promote, and support breastfeeding created a conducive breastfeeding environment, with intensive mentorship, follow-up, and audits for quality improvement. Initial year 1 achievements (March 2019–2020) included: 8,912 mothers were provided breastfeeding counseling in the neonate ward; 1,402 mothers were supported to express milk; and 138 vulnerable infants received DHM.

**Conclusions**

Optimizing nutrition and exclusive human milk diets for SSNB is an essential component within the standards of SSNB care. Integrated models, such as MBFI+, are feasible in LMIC settings and expansion is needed as a core strategy to ensure that SSNB survive and thrive.

## O146. Experiences, perceptions, and preferences about facility-based delivery care in Rural Mexico: a qualitative study

### Zeus Antonio Aranda Remon, Mariana Yulian Montano Sosa, Viviana Caamal

#### Partners In Health

*BMC Proceedings 2024*, **18(5):**O146

Submission ID #: IMNHC1063

**Background**

The nongovernmental organization Compañeros En Salud (CES) has been attending births since 2017 at a birthing center located in the rural area of the Sierra Madre de Chiapas, Mexico. The main purpose of the center is to achieve the best birthing experience for its users. To gain insight into how to accomplish this, we explored the experiences, perceptions, and preferences about childbirth care of women living in the area of influence of the CES birthing center.

**Methods**

From June to August 2022, we conducted 20 semistructured interviews with women from the Sierra Madre region who had given birth by natural childbirth to a live newborn in the six months prior to the interview in a health facility in Chiapas. The sample was purposively sampled to be representative of women of different ages, parity, area of residence, and place of last delivery. The median age of the women interviewed was 29 years and the parity was three. We used the eight dimensions of the World Health Organization health system responsiveness framework to develop the interview guide. We analyzed the interviews using thematic analysis.

**Results**

We identified three main themes, aligned with some of the dimensions of the World Health Organization framework used in the interview guide. Women often experienced several barriers to accessing health facilities with delivery care, most notably long distances and lack of transportation. Delivery care was perceived as poor quality by several women as a result of obstetric violence, manifested in the form of disrespectful care, lack of confidentiality and privacy, and overriding women’s autonomy. Women also highlighted the need to integrate traditional knowledge into delivery care, involving both traditional practices and providers.

**Conclusions**

There is a profound need to improve the quality of delivery care in health facilities in Chiapas and align it with the preferences of the local population to ensure respect for the human rights of clients and improve their experience and satisfaction with care. Although the study included women from specific regions of the state due to convenience, these findings are relevant to similar low-resource rural areas in the state and in Mexico.

## O147. Routine measurement of reproductive, maternal, and newborn health programs in the pacific region: a review of Health Management Information System (HMIS) indicators and data availability

### Zeshi Fisher^1^, Sandra Paredez^2^, Titilola Duro-Aina^2^, Caroline Homer^1^

#### ^1^Burnet Institute; ^2^United Nations Population Fund

*BMC Proceedings 2024*, **18(5):**O147

Submission ID #: IMNHC1055

**Background**

Routine service data are necessary for informing reproductive, maternal, and newborn health (RMNH) services management, including human resource capacity, domestic resource mobilization, and quality improvement. They also play a key role in national and international program monitoring. However, many national HMISs in the Pacific Region do not generate quality data on RMNH. The purpose of this project was to benchmark data availability in six Pacific Island countries against international and national RMNH indicators with an HMIS data source to identify gaps and opportunities for improvement.

**Methods**

The project was undertaken by UNFPA Pacific Sub-Regional Office, Burnet Institute, and Ministry of Health partners in Fiji, Kiribati, Samoa, Solomon Islands, Tonga, and Vanuatu. It was completed in two parts: (1) the development of reference lists of indicators with an HMIS preferred or alternative data source defined in international initiatives and national strategic and planning frameworks in each country; and (2) a review of HMIS monitoring tools and periodic reports to determine the collection and availability of RMNH data elements and data on key populations required to generate defined indicators.

**Results**

The key finding is that there are significant gaps in the availability of RMNH data for indicators in existing frameworks, and these are only a fraction of the information needed for effective decision-making and reporting by national programs. The lack of data on key populations is also significant. In five of six countries, RMNH data are not collected on people with a disability. Data on adolescents is also limited—RMNH indicators with an HMIS data source disaggregated by adolescent age groups 10–14 and 15–19 are not routinely published on a quarterly or annual basis.

**Conclusions**

This review provides insights into challenges faced by countries in the Pacific Region in effectively monitoring their RMNH programs as well as reporting to international initiatives. It will be used to inform recommendations on the selection of indicators and the standardization of monitoring tools used in national HMISs. Countries need additional guidance in best practices for facility-based monitoring approaches, assessing information needs, and selecting indicators relevant to RMNH in their national context.

## P148. Tripartite consultations for improving accountability and quality of care

### Vinit Sharma

#### United Nations Population Fund

*BMC Proceedings 2024*, **18(5):**P148

Submission ID #: IMNHC1053

**Background**

Policymakers, service providers, and users constitute the three angles of the triangle in sexual and reproductive health (SRH) programs. Most of the research has focused on these issues in isolation and there have been limited efforts to study the three dimensions together. A lack of coordination, collaboration, and communication among the different stakeholders has limited the success of national SRH programs. While sound policies and legal frameworks create an enabling environment, an understanding is lacking about the reasons why women and couples are not using a particular health service.

**Methods**

Inclusiveness and collaboration are at the heart of an innovative concept known as the Tripartite Consultation, which brings together the three key stakeholders to promote quality SRH programs. The three parties—policymakers, health service providers, and users—come together for a meaningful dialogue that leads to practical action plans subject to joint review. The process can be divided into three distinct phases: (1) the consultation, (2) planning and implementation of activities, and (3) joint review after one year. The first phase of the Tripartite Consultation includes a national workshop organized by the national government. The first day of the workshop involves presentations by the three stakeholders, each highlighting its own contributions to the program and the perceived reasons for dissatisfaction with the program. Each group also analyzes the strengths, weaknesses, and limitations at the three levels.

**Results**

The first Tripartite Consultation in Bangladesh succeeded in identifying expectations, needs, and desired outcomes from all categories of stakeholders and the three groups prepared action plans to be reviewed jointly after one year.

**Conclusions**

This comprehensive model encourages individual and community empowerment and responds to community needs. The model endeavors to reduce health inequities by promoting inclusiveness and well-being and promotes accountability and better quality of care.

## O149. How simulation and team training can enable a newly Minted Cadre of Midwives as educators and mentors in India

### Seema Handu^1^, Radha Reddy^2^, Indie Kaur^2^, Erin Cole^3^, Susanna Cohen^3^, Narender Goswami^1^, Nidhi Subramaniam^1^, Divya Vincent^1^, Liya Jose^1^, Rakesh Ghosh^4^, Alisa Jenny^4^, Dilys Walker^4^

#### ^1^PRONTO International; ^2^Fernandez Foundation; ^3^University of Utah; ^4^University of California, San Francisco

*BMC Proceedings 2024*, **18(5):**O149

Submission ID #: IMNHC1046

**Background**

The Government of India launched the Guidelines on Midwifery Services in 2018 to create a new cadre of nurse practitioners in midwifery (NPMs) and build the capacities of NPM educators (NPMEs). The Fernandez Foundation trained the first cohort of NPMEs selected by the Telangana state government in 2021. The Fernandez Foundation is responsible for training NPMEs in how to train NPMs in midwifery competencies as well as developing them as educators. To fulfill the educator competencies, the Fernandez Foundation, with UNICEF’s support, invited PRONTO to offer simulation-based training to build NPMEs’ educator, mentoring, and communication skills.

**Methods**

PRONTO, the University of California San Francisco, and the University of Utah conducted a five-day simulation educator training for Telangana’s NPMEs. The training included interactive modules on how to create a safe learning space, facilitate and debrief simulation with reflective learning, use low-tech simulators, integrate teamwork and communication and respectful maternity care practices into clinical learning, and navigate hierarchy and interprofessional conflict.

Surveys evaluated acceptability, feasibility, and adoptability of PRONTO training on a four-point Likert scale. We also measured facilitation, debriefing, and teamwork/communication knowledge through a 27-point pre-post questionnaire.

**Results**

Eighteen NPMEs participated in the training and responded to the surveys. All (100%) participants believed the training was adoptable and over 90% felt it was appropriate. Accessibility was achieved by providing learners with PRONTO’s robust online training materials, all of which were noted to be successfully accessed by the learners. Knowledge increased in multiple topic areas, especially teamwork and communication (81%–96%) and simulation and debrief (60%–93%). All participants responded that they gained experience in organizing simulations, building case scenarios, and handling “real” clinical emergencies. All learners indicated that they plan to use simulations in their training sites.

**Conclusions**

The training addressed specific gaps in the current educator curriculum, namely simulation and debriefing with reflective learning, which build educator competencies, and simulation training skills that will improve the overall quality of the NPMEs and broadly strengthen the educator core competencies, assuring a high-quality NPME workforce, which is critical to the long-term goal of improving quality of care for women and babies in India.

## O150. Assessing the impact of COVID-19 on the coverage of maternal health services in Burkina Faso

### Abdoulaye Maïga^1^, Moussa Bougma^2^, Emily Wilson^1^, Melinda Munos^1^, Théodore S. Kaboré^2^, Gildas G. Tou^2^, Safia Jiwani^1^, Agbessi Amouzou^1^

#### ^1^Johns Hopkins Bloomberg School of Public Health; ^2^Université Joseph Ki-Zerbo

*BMC Proceedings 2024*, **18(5):**O150

Submission ID #: IMNHC1043

**Background**

Like most countries, Burkina Faso activated their epidemic emergency contingency, preparedness, and response plans during the COVID-19 pandemic. While these measures were designed to prevent and control the spread of the pandemic, they also can cause major disruptions to health service utilization. We assessed the impact of COVID-19 on the coverage of selected maternal health services and inequalities in Burkina Faso.

**Methods**

We analyzed data from two household surveys in two provinces in Burkina Faso, one urban (Kadiogo) and the other rural (Boulkiemde). The first coverage survey was conducted immediately before the pandemic in February–March 2020 on a sample of 3,375 households. The second survey was carried out in May–June 2022 in the same provinces using a similar sampling design and sample size. We assessed the impact of the pandemic on antenatal care (ANC), delivery, and cesarian section, with a focus on the number and timing of care-seeking episodes, and source and content of care.

**Results**

Coverage of ANC and delivery care was generally high in both provinces (over 76% and 94% for four or more ANC visits and delivery care, respectively) and unaffected by restrictions due to the pandemic. We found only small and non-statistically significant changes in timely initiation of ANC, at least one visit with a skilled attendant, four or more ANC contacts, and ANC contents before and during the COVID-19 period across the two provinces. The largest drop was in the timely initiation of ANC of 11 percentage points in the rural province. No systematic downward trends were noticed in the receipt of ANC content interventions. Similarly, only minor changes were observed for institutional delivery, skilled attendant at birth, and cesarean section. A downward change was observed in the rural province, where institutional delivery decreased from 96% to 84% and cesarian delivery from 5% to 2%, although non-statistically significant.

**Conclusions**

Contrary to the early warnings, COVID-19 restrictive measures and related disruptions did not appear to have significantly decreased the coverage of maternal health services in these areas where the coverage of these services was already high. Coverage levels have been maintained in both urban and rural provinces.

## O151. Costing Zambia’s commitment to reach the every newborn action plan target 4

### Getrude Sibuchi Miyanda^1^, Gae Mundundu^1^, Kunda Mutesu-Kapembwa^1^, Eliana Jimenez Soto^2^, Mary Nambao^3^, Alison Morgan^3^

#### ^1^Ministry of Health, Zambia; ^2^Independent Cosultant; ^3^Global Financing Facility

*BMC Proceedings 2024*, **18(5):**O151

Submission ID #: IMNHC1038

**Background**

In the context of an increasing neonatal mortality trend for Zambia, the government has embarked on mobilizing resources for the scale-up of level 2 in patient care for small and sick newborns (SSNB). Global guidelines and standards have been adapted to ensure best practices inform service delivery so that the scale-up leads to both increased coverage and improved quality of care. Costing “what it takes” to scale up quality of care in the country highlights pragmatic considerations and trade-offs that are usually off-sight when costings are estimated based on global parameters.

**Methods**

To strengthen existing health care packages and scale-up plans, the Service Standards for Health Institutions Providing Neonatal Care were produced in 2020. They cover infrastructure, human resources, referrals, and equipment for all levels of care, including district hospitals delivering SSNB care, and provide an important number of parameters to estimate scale-up costings. Although less detailed in terms of implementation guidance, the current adaptation of World Health Organization standards for improving the quality of care for SSNB provide additional nuances to be considered. A costing tool was developed to estimate required scale-up resources and inform future output-based financing arrangements.

**Results**

Producing local costs of scale-up plans driven by local standards and health system specificities contributes to more realistic plans and costs. For example, although detailed guidance was produced in 2020, due to the rapidly evolving evidence base, some standards such as floor space requirements need reconsideration to implement a no-separation policy. Hidden costs such as the time and effort required by national and regional government officials for managing the scale-up are also brought to the surface.

**Conclusions**

We present the experience of Zambia to plan and budget for the scale up of level 2 in patient care for SSNB to “tell it as it is” and show the opportunities and challenges of making Every Newborn Action Plan target 4 a reality in the current environment of tightening fiscal space and competing priorities. Lessons learned will cover the nuances of translating global guidance into costed scale-up plans and the trade-offs facing policymakers to

## O152. Seroprevalence and risk factors for severe acute respiratory syndrome Coronavirus 2 infection in women and children in a rural district of Bangladesh: a cohort study

### Rasheda Khanam^1^, Md. Shafiqul Islam^2^, Mohammad Sayedur Rahman^3^, Salahuddin Ahmed^2^, Asmd Ashraful Islam^2^, Tarik Hasan^2^, Emran Hasan^2^, Nabidul Chowdhury^2^, Arunangshu Roy^2^, Iffat Jaben^2^, Asim Nehal^2^, Sachiyo Yoshida^3^, Alexander Manu^4^, Rubhana Raqib^5^, Eric McCollum^6^, Abdullah Baqui^1^

#### ^1^Johns Hopkins Bloomberg School of Public Health; ^2^Projahnmo Research Foundation; ^3^Uppsala University; ^3^World Health Organization; ^4^University of Ghana School of Public Health; ^5^International Center for Diarrheal Disease Research, Bangladesh; ^6^Johns Hopkins University

*BMC Proceedings 2024*, **18(5):**O152

Submission ID #: IMNHC1032

**Background**

Bangladesh reported its first COVID-19 case on March 8, 2020. Despite lockdowns and promoting behavioral interventions, as of December 31, 2021, Bangladesh reported 1.5 million confirmed cases and 27,904 COVID-19-related deaths. To understand the course of the pandemic and identify risk factors for severe acute respiratory syndrome coronavirus 2 **(**SARs-CoV-2) infection, we conducted a cohort study from November 2020 to December 2021 in rural Bangladesh.

**Methods**

After obtaining informed consent and collecting baseline data on COVID-19 knowledge, comorbidities, socio-economic status, and lifestyle, we collected data on COVID-19-like illness and care seeking weekly for 54 weeks for women (*n* = 2683) and their children (*n* = 2433). Between March and July 2021, we tested all participants for SARS-CoV-2 antibodies using ROCHE’s Elecsys® test kit. We separately calculated seropositivity rates and 95% confidence intervals (95% CI) for women and children. Using log-binomial regression models, we calculated unadjusted and adjusted relative risk (RR) and 95% CI of seropositivity for different age and risk groups.

**Results**

Overall, about one-third of women (35.8%, 95% CI = 33.7–37.9) and one-fifth of children (21.3%, 95% CI = 19.2–23.6) were seropositive for SARS-CoV-2 antibodies. The seroprevalence rate doubled for women and tripled for children between March 2021 and July 2021. Compared to women and children with the highest household wealth tertile, women and children from poorer households had a lower risk of infection (RR, 95% CI = 0.83 [0.71–0.97]) and (0.75 [0.57–0.98]), respectively. In addition, the risk of infection among women was higher if she reported chewing tobacco (RR = 1.19, 95% CI = 1.03–1.38) and if her husband had an occupation requiring him to work indoors (RR = 1.16, 95% CI = 1.02–1.32). The risk of infection was higher among children if paternal education was >5 years (RR = 1.37, 95% CI = 1.10–1.71).

**Conclusions**

We provided prospectively collected population-based data, which could contribute to designing feasible strategies against COVID-19 tailored to high-risk groups. The most feasible strategy may be promoting preventive care practices; however, collecting data on reported practices is inadequate. A more in-depth understanding of the factors related to adoption and adherence to practices is essential.

## O153. How Zambia reduced inequalities in under-five mortality rates over the last two decades: a mixed-methods study

### Choolwe Jacobs^1^, Mwiche Musukuma^1^, Brivine Sikapande^2^, Fernando C. Wehrmeister^3^, Ovost Chooye^2^, Ties Boerma^4^, Charles Michelo^5^, Andrea Blanchard^4^

#### ^1^The University of Zambia; ^2^Zambia Ministry of Health; ^3^International Center for Equity in Health - Federal University of Pelotas; ^4^University of Manitoba; ^5^Nkwazi University

*BMC Proceedings 2024*, **18(5):**O153

Submission ID #: IMNHC1031

**Background**

Zambia experienced one of the fastest reductions in under-five mortality rates (U5MR) in sub-Saharan Africa, with major improvements in socioeconomic disparities. This study aimed to understand the extent to which and how Zambia has reduced socioeconomic and geographic inequalities in U5MR since 2000 through mixed-methods research.

**Methods**

Using nationally-representative data from the last four Zambia Demographic and Health Surveys (ZDHS, 2001/2, 2007, 2013/14, and 2018), we examined equity trends in U5MR and related influences—including reproductive, maternal, newborn, and child health (RMNCH) intervention coverage; household water and sanitation; and fertility—between wealth, rural-urban, and education groups. We conducted in-depth reviews of policy and program documents and health system data relevant for improving equity in child survival between 1990 to date. The study was led by researchers at the University of Zambia and Ministry of Health within the Countdown to 2030 country collaboration.

**Results**

Under-five mortality declined from 168 to 64 deaths per 1,000 live births between 2001/2 and 2018 ZDHS rounds, with major reductions in inequalities between wealth, education, and urban-rural residence groups. Yet reductions in under-five mortality inequalities were not accompanied by reduced gaps between wealth groups in absolute levels of income or education. However, inequalities reduced markedly for coverage of RMNCH, malaria, and HIV interventions but less so for water or sanitation and fertility levels. Several interrelated policy and health systems drivers were identified as contributors to reducing RMNCH inequalities, including policy commitments to equity in RMNCH, financing with a focus on disadvantaged groups, multisectoral partnerships and horizontal programming, expansion of infrastructure and human resources for health, and involvement of community stakeholders and service providers

**Conclusions**

Zambia’s major progress in reducing inequalities in child survival between the poorest and richest people appeared to be driven primarily by government policies and programs that centrally valued equity, despite ongoing gaps in absolute income and education levels. The study is valuable for informing Zambia’s efforts to further improve child survival for all and providing guidance for other countries on the importance of underpinning RMNCH programs with comprehensive health policy and systems strengthening that puts equity at the center.

## O154. Individual and facility-level factors that affect quality of care: self-reported provision of respectful care among providers in Malawi

### Carolyn Smith Hughes^1^, Martha Kamanga^2^, Alisa Jenny^1^, Brady Zieman^3^, Charlotte Warren^3^, Dilys Walker^1^, Abigail Kazembe^2^

#### ^1^University of California, San Francisco; ^2^University of Malawi Kamuzu College of Nursing; ^3^Population Council

*BMC Proceedings 2024*, **18(5):**O154

Submission ID #: IMNHC1021

**Background**

Over the past decade, there has been increased attention on disrespect and abuse in childbirth and development of interventions to improve women's experiences. However, most studies focus on women’s perspectives. The nature of disrespect and abuse is complex, and health care professionals often work in suboptimal and high-stress conditions without routine training and support to effectively care for patients. We describe self-reported provision of respectful maternity care (RMC) among providers and identify areas for workforce support.

**Methods**

We surveyed health care providers (*n*=288) working in maternity and neonatal care wards in 25 health facilities in Malawi. Providers were interviewed to assess their knowledge and experiences regarding maternity care. We used the 15-point validated person-centered maternity care (PCMC) scale to describe supportive care, dignity and respect, and communication and autonomy. We analyzed mean PCMC scores as a continuous variable (range: 0–45), and used multi-level mixed-effects regression to assess associations between mean PCMC score and selected provider (e.g., age, cadre, training) and facility/system factors (e.g., workplace culture, including burnout, relationship with manager, unscheduled overtime, facility type, sector).

**Results**

In our sample, 31% of providers were male, most were nurses or midwives (85%), and 57% were age 30 or younger. Mean PCMC score was 33.2 (standard deviation: 5.8; range: 16–45). Few providers reported physically abusing patients (5%). Only 16% said they always introduce themselves to women before providing care; less than half (36%) reported frequently allowing women to labor in a position of their choice. We found evidence of statistically significant associations between PCMC score and provider cadre, time in training, burnout, and poor relationship with manager.

**Conclusions**

While self-reported physical abuse was low, measures related to communication and autonomy indicate opportunities to improve how providers engage with birthing women. Associations between PCMC and provider and workplace factors, including provider cadre and burnout, may indicate that RMC interventions that are inclusive of interprofessional mentoring, team relationships, and communication may support providers and promote RMC. Our findings have important implications for the development of interventions to improve experiences for providers and women in Malawi and similar settings.

## O155. Intrapartum use of oxytocin injection: a survey of health care providers’ practice in Nigeria

### Chioma Ejekam^1^, Ifeoma Okafor^1^, Kehinde Okunade^1^, Jude Nwokike^2^, Uchenna Igbokwe^3^

#### ^1^College of Medicine University of Lagos; ^2^U.S. Pharmacopeial Convention; ^3^Solina Center for International Development and Research

*BMC Proceedings 2024*, **18(5):**O155

Submission ID #: IMNHC1018

**Background**

Studies have demonstrated that more women who want to deliver babies have undergone inappropriate use of oxytocin for labor induction and augmentation at all levels of the health system, including primary health care facilities in many parts of the world, including sub-Saharan Africa. This study assessed the pattern of intrapartum administration of oxytocin among different cadres of health care providers in Nigeria.

**Methods**

This was a descriptive cross-sectional study, conducted in 2019, of 6,299 health care providers who offer obstetrics and gynecological services recruited from 1,894 health facilities in the public and private sectors in 12 states across Nigeria. Providers were doctors, nurses/midwives, and community health workers (CHWs). Data were collected using an electronic questionnaire, analyzed using SPSS, and presented in frequencies and percentages.

**Results**

Findings showed that oxytocin was administered by 85.5 % of nurses/midwives, 58.0% of doctors, and 38.1% of CHWs. A high percentage of oxytocin administrations by CHWs were done in public facilities (70.0%) compared to private facilities (30.0%). Seventy-eight percent of the providers used oxytocin for prevention of postpartum hemorrhage, 68% for augmentation of labor, and 53.0% for induction of labor. Specifically, nurses/midwives reported use of oxytocin for augmentation (69.6%) and induction of labor (56.6%); among CHWs, 48.8% reported use of oxytocin for augmentation and induction of labor. The most commonly used route of administration was intravenous infusion (92.0%) for both induction and augmentation, followed by intramuscular for induction (10.3%) and for augmentation (9.4%), and intravenous push for both induction and augmentation of labor (8%). The doses commonly used for induction of labor were 5IU (38.0%), 10IU (47.0%), and 20IU (5.0%). For augmentation of labor, the doses often used were 5IU (48.0%), 10IU (44.0%), and 20IU (4.0%).

**Conclusions**

Our study highlighted the unauthorized and high intrapartum use of oxytocin among CHWs. Attention is needed to address quality of care and use of uterotonics for induction and augmentation. As new uterotonics are introduced it is important to ensure clear communication of the right indications for use and the appropriately skilled manpower.

## O156. OTIP: an innovative obstetric triage implementation package to reduce delay and improve the quality of care at referral hospitals in Ghana

### Martin Owusu Boamah^1^, Erin Pfeiffer^2^, Mary Ashinyo^3^, Fiona Bryce^2^, Rohit Ramaswamy^4^, Sylvia Deganus^5^, Cecilia Tetteh^6^, Medge Owen^2^

#### ^1^Ghana Health Service; ^2^Kybele; ^3^Ministry of Health Ghana; ^4^Cincinnati Children’s Hospital Medical Center; ^5^World Health Organization; ^6^Greater Accra Regional Hospital

*BMC Proceedings 2024*, **18(5):**O156

Submission ID #: IMNHC1014

**Background**

Institutional delivery reduces maternal and neonatal morbidity and mortality. However, hospitals in low-resource countries commonly operate on a first-come, first-served basis, resulting in laboring women waiting hours before assessment by providers. This waiting period, “the third delay,” escalates obstetric complications and contributes to mortality. Timely assessment and treatment improve outcomes, yet no documented obstetric triage system exists in low-resource countries.

**Methods**

Humanitarian non-profit organization Kybele and the Ghana Health Service partnered to develop the Obstetric Triage Implementation Package (OTIP) to promote timely, quality care. Establishment of a Ghanaian technical advisory group (TAG) ensured local ownership and integration into the health system. A toolkit introduced interactive training content, an implementation manual, triage space and equipment guidelines, treatment protocols, a simulation game to role-play case scenarios, and monitoring tools.

From January 2019 to March 2022, OTIP was scaled to eight referral hospitals, cascading training two facilities at a time. The TAG established a national training team and prioritized target hospitals, each identifying clinical champions for training. Champions received a two-day intensive course on triage concepts and quality improvement and then co-led local training and implementation. Data was collected at baseline, 3, 6, and 12 months post-implementation to assess timeliness of care, documentation thoroughness, and accuracy of risk assessment.

**Results**

The introduction of OTIP resulted in training 510 frontline health workers in eight Ghanaian hospitals, serving over 50,000 women and newborns. Waiting time dramatically decreased. At baseline, only 6% (22/400) of women were evaluated within 10 minutes of arrival, the internationally recommended standard. This improved to 85% (367/431) 12 months following implementation. Thoroughness of documentation improved from 68% to 84% and risk status was correctly assigned in 83% (394/474) of patients. Midwives reported that OTIP improved workflow and enhanced communication with doctors.

**Conclusions**

National scale up of OTIP in Ghana is being promoted due to its success. OTIP has expanded into Liberia led by Ghana’s National Triage Trainers. OTIP is evidence-based, tested, and context adaptable and is led and sustained by local frontline health care providers. The model is applicable to high-volume hospitals in resource-constrained settings around the globe and could impact childbirth outcomes worldwide.

## O157. Implementing the World Health Organization (WHO) labour care guide to reduce the use of cesarean section in four hospitals in India: a pragmatic, stepped-wedge, cluster-randomized pilot trial

### Joshua Vogel^1^, Veronica Pingray^2^, Fernando Althabe^3^, Luz Gibbons^2^, Mabel Berrueta^2^, Rocio Rodriguez^2^, Yeshita Pujar^4^, Manjunath Somannavar^4^, Sunil Vernekar^4^, Saraswati Welling^4^, Elizabeth Armari^1^, Shivaprasad Goudar^4^

#### ^1^Burnet Institute; ^2^Institute for Clinical Effectiveness and Health Policy, Argentina; ^3^World Health Organization; ^4^KLE Academy of Higher Education and Research

*BMC Proceedings 2024*, **18(5):**O157

Submission ID #: IMNHC1013

**Background**

The WHO Labour Care Guide (LCG) is a new clinical tool that reflects WHO’s latest guidelines for effective, respectful care during labor and childbirth. Implementing the LCG into routine care requires a strategy that effectively improves health care provider practices so that the tool is used and interpreted correctly. Such a strategy may optimize the use of cesarean section, along with other intrapartum interventions, health outcomes, and women’s experience of care. This trial aimed to 1) develop and optimize a strategy for implementing the LCG, and 2) evaluate the implementation of the LCG strategy compared with usual care.

**Methods**

A six-month mixed-methods formative phase was used to co-design the LCG strategy, which was comprised of provider training centered on low-dose, high-frequency principles, encouraging supportive supervision, and integrating monthly audit and feedback meetings to monitor cesarean section rates using Robson Classification. This strategy was then evaluated within a stepped-wedge, cluster-randomized controlled trial in four public hospitals in India over 12 months. The control condition was usual care, and the primary outcome was the cesarean section rate in women in Robson Group 1. We conducted a mixed-methods process evaluation to explore fidelity, reach, dose, and any adaptations of the LCG strategy.

**Results**

Between 1 July 2021 and 15 July 2022 all four hospitals were randomized, during which 27,114 women gave birth to 27,684 babies. Presentation of the primary analysis will explore the effects of the LCG strategy on cesarean section rates and effects on secondary outcomes—clinical and process of care outcomes—and women’s experience of care. We will also present process evaluation findings, which used in-person standardized facility assessments, 43 in-depth provider interviews, a survey of 100 providers, audits of 654 randomly sampled LCGs, and document review.

**Conclusions**

This is the largest trial of WHO's new LCG. Findings will guide clinicians, administrators and policy-makers on how to effectively implement the LCG into clinical practice and the effects the LCG strategy has on cesarean section rates and the process of care and health and experience outcomes. The trial findings will inform the rollout of LCG internationally.

## O158. Improving quality of care through private sector integration for the new maternal and newborn health package in Indonesia

### Kaela Barna, Inraini Fitria Syah, Astara Lubis, Tamara Chikhradze

#### Results for Development

*BMC Proceedings 2024*, **18(5):**O158

Submission ID #: IMNHC1012

**Background**

In Indonesia, midwives provide 84.8 % of all antenatal care (ANC) and carry out 67.7% of deliveries, yet only a disproportionately low number of private midwives contracted with the national health insurance scheme. This limits the financial protection of mothers, contributes to lower quality care, and disrupts continuity of care throughout pregnancies due to the limited access that private midwives, and the facilities they work in, have to publicly funded health care goods and equipment.

**Methods**

Through a locally led approach, the Health Financing Activity has supported the Government of Indonesia in developing a strategic health purchasing program that integrates private midwives into a maternal and newborn health care package.

**Results**

Since 2019, the Health Financing Activity has provided strategic oversight to build Government of Indonesia’s capacity to conduct strategic health purchasing through the establishment of a participatory, multi-agency governance structure, gathering and analysis of data, development of evidence-based recommendations, and facilitation of multi-level technical discussions to coordinate policy design.

**Conclusions**

This has resulted in new regulations that establish public-to-private provision of essential health care goods and organizes a cross-sectoral referral system for services, such as ultrasound, that are currently being piloted in the Serang district and Serang City, Banten, Indonesia. The pilot will determine the efficacy of the novel supply chain logistics, referral pathways, coordination mechanisms, and strengthened public role in monitoring service delivery before the potential scale up to the national level. This effort is anticipated to ensure that pregnant people can obtain maternal and newborn health care that meets service standards regardless of the facility they attend, build health system capacity, and improves district health office stewardship of care.

## O159. Timing of stillbirths and neonatal deaths by gestational age in two hospitals in Kenya: secondary data analysis of the PRECISE-DYAD cohort study to inform evidence-based interventions for improving future outcomes

### Sanjukta Amrita Singh^1^, Hannah Blencowe^1^, Akuze Joseph Waiswa^1^, Marleen Temmerman^2^, Grace Mwashigadi^2^, Angela Koech^2^

#### ^1^London School of Hygiene & Tropical Medicine; ^2^The Aga Khan University

*BMC Proceedings 2024*, **18(5):**O159

Submission ID #: IMNHC1009

**Background**

In Kenya, the stillbirth and neonatal mortality remains high. There is limited data on cause of these deaths globally, and therefore, improving understanding of chronological timing (antepartum/intrapartum/early and late neonatal), gestational age timing (preterm, term, post-term) and their contributing risk factors are first steps towards prevention. Misclassification of perinatal deaths by skin changes at birth can impact this understanding and requires further exploration.

**Methods**

Secondary data analysis of the PRECISE-DYAD prospective cohort study of women giving birth in two facilities in Kenya between 2019-2022 was undertaken. Stillbirths were classified as antepartum or intrapartum based on fetal heart rate (FHR) on admission and as early (before 28 completed weeks) and late (after 28 weeks) gestation stillbirths. Neonatal deaths were classified as early (before day 7) and late (day 7–28) neonatal deaths. Stillbirths were considered potentially misclassified if reported skin appearance at birth was discordant with FHR status on admission. Evidence of association of misclassification by method of FHR was explored. Key contributing risk factors associated with these deaths were explored.

**Results**

52 stillbirths (38.5% antepartum, 38.5% intrapartum, and 23% unknown) and 14 neonatal deaths (10 early) were reported among 2,090 total births. Nearly three-quarter (73.8%) of stillbirths and 80% of early neonatal deaths were born at 28 or more weeks of gestation. Misclassification of intrapartum stillbirths was observed when skin appearance was used at birth instead of FHR on admission, and it varied depending on FHR method. The odds of skin changes of maceration/peeling reported for intrapartum stillbirths was 2.3 times higher when Pinard stethoscope was used. Preterm gestational age (*p*-value less than 0.001) and multiple gestation (*p*=0.02) were important risk factors for these deaths.

**Conclusions**

Improving reporting of timing of perinatal deaths is critical in reducing knowledge gaps about quality of intrapartum monitoring in low-resource settings. Preterm gestation is strongly associated with stillbirths and neonatal deaths and necessitates identification of care pathways for women at risk. Misclassification of intrapartum stillbirths is an area of concern and needs deeper exploration. Larger representative studies in this population are needed for predictors of perinatal deaths.

## O160. Leveraging geospatial intelligence/evidence to inform Maternal and Newborn Health Care (MNHC) infrastructure investment

### Emmanuel Katyoka^1^, Rabson Zimba^1^, Olatubosun Akinola^1^, Jason Wamulume^2^

#### ^1^Clinton Health Access Initiative; ^2^Zambia Ministry of Health

*BMC Proceedings 2024*, **18(5):**O160

Submission ID #: IMNHC1004

**Background**

Like other resource-constrained settings, Zambia is characterized by high maternal and infant mortality rates. This is inspite of recent progress made towards reducing mortality rates, from 398 per 100,000 live births in 2014 to 278 per 100,000 live births in 2018. The major factors contributing to high maternal and infant mortality rates in Zambia are associated with the three delays in accessing health services: the delays in deciding to seek care, the delay in accessing a facility, and the delay in accessing services of a skilled health worker. Within the context of the three delays, the choice of facility location is critical. However, identifying and prioritizing the precise location with the greatest potential of increasing access to maternal services is in practice not a straightforward process, more so in resource-constrained settings where the need for infrastructure is pervasive. A baseline study conducted in Eastern and Southern provinces of Zambia established that 72% of eligible facilities in Eastern and 65% in Southern had no capacity to support maternal services. Estimates of the investment outlay required to close capacity gap was $41.8 million

**Methods**

Using a cross-sectional approach to assess the availability of sexual, reproductive, maternal, newborn, child, and adolescent health and nutrition services and infrastructure data was collected from a total of 327 facilities in Eastern Province and 365 facilities in Southern Province. Infrastructure gaps were established by assessing available bed spaces against patient service volumes, as measured by total deliveries. Using baseline and HMIS data, geospatial modeling was conducted to map infrastructure capacity against maternal and infant mortality data to identify the critical few sites accounting for adverse mortality outcomes.

**Results**

Using geospatial intelligence, we identified 27 sites that had infrastructure challenges and were among key drivers of maternal and newborn mortality in Eastern and Southern provinces. The program prioritized 27 sites for infrastructure improvements, reducing the required outlay from $41.8 million to $2 million.

**Conclusions**

Geospatial intelligence can be used effectively to pinpoint locations where infrastructure investments have the greatest potential to address access barriers that, once addressed, have the potential to reduce maternal and infant mortality rates.

## O161. Implementing a multifaceted approach to reduce fresh stillbirths in Mbarara District, Uganda

### Agatha Nshabohurira^1^, Beatrice Bainomugisha^2^

#### ^1^Ministry of Healt, Uganda; ^2^Jhpiego

*BMC Proceedings 2024*, **18(5)**:O161

Submission ID #: IMNHC1000

**Background**

The fresh stillbirth (FSB) rate in Uganda in 2019/2020 was 12 per 1,000 births; in Mbarara District, it was at 7.3 per 1,000 births. These rates exceed the national target of less than 5/1,000. Research shows that 40% of the FSBs in Uganda can be prevented by improving the quality of service.

**Methods**

To improve the quality of obstetric care, the Mbarara district health team analyzed maternal child health data from 10 facilities (7 public and 3 private) to determine FSB trends between October 2021 to June 2022. The analysis revealed that unaudited FSBs were occurring in facilities. To address this gap, a district continuous quality improvement team worked with health facility leadership to audit all FSBs at their facilities. This process identified modifiable factors contributing to FSBs. These factors were then prioritized and used to inform the development of facility action plans. Actions included supporting mentorship for midwives on labor monitoring, newborn resuscitation and educating pregnant women on birth preparedness and harmful practices. Facility data was continuously reviewed to track changes in the number of FSBs on a weekly basis and to identify other influencing factors that could be addressed.

**Results**

During the intervention period, facilities recorded a marked decrease in FSBs (from 22 registered in FY2020/21 [5.6/1,000] to 10 FSBs in FY 2021/2022 [2.6/1000]) a decrease of 54% in nine months. As a result of this effort, coordination between district leadership and other stakeholders has improved. There is also a greater commitment from both technical and political leadership to ending preventable maternal and perinatal deaths as demonstrated through increased budget allocation to the maternal child health section, recruiting and facilitating placement of support staff to bridge service delivery gaps. Midwives have improved their skills in monitoring labor and have expanded health education talks given to women

**Conclusions**

This affordable, integrated intervention demonstrates that a significant number of FSBs that happen in facilities in Mbarara and Uganda are preventable. Data use for decision making, prompt auditing of FSBs, on-the-job mentorship, and advocacy by district leadership not only improve the quality of health services offered but also improve pregnancy outcomes.

## O162. Postnatal and postpartum utilization among women receiving group antenatal care vs. routine individualized care: a cluster-randomized controlled trial in Ghana

### Elizabeth Awini^1^, Jody Lori^2^, Veronica Esinam Awo Apetorgbor^1^, Vida Ami Kukula^1^, Georgina Amankwah^1^, John Williams^1^, Bidisha Ghosh^2^, Ruth Zielinski^2^, Nancy Lockhart^2^, Cheryl Moyer^2^

#### ^1^Ghana Health Service; ^2^University of Michigan

*BMC Proceedings 2024*, **18(5):**O162

Submission ID #: IMNHC996

**Background**

The purpose of this study is to assess behavioral differences in care-seeking patterns related to postnatal and postpartum care among women randomized to group-based antenatal care (intervention) or routine individual antenatal care (control) in Eastern region of Ghana. We hypothesized that mothers and infants of mothers randomized into the intervention group receiving group antenatal care will have better attendance at postnatal and postpartum visits than women in in the control group.

**Methods**

A cluster-randomized controlled trial was designed using 14 rural health facilities in the Eastern region of Ghana. Eligibility included: (1) less than 20 weeks’ gestation at first ANC visit; (2) speaks Dangme, Ga, Akan, Ewe, or English; (3) over 15 years of age; (4) not considered a high-risk pregnancy upon enrolment. We recruited 1,761 participants between July 2019 and November 2021. Data were collected at baseline and six weeks postpartum and entered into a secure, web-based software. We used Pearson’s chi-square to compare differences between the control and intervention groups for attendance at 6-7 day and 6 week postnatal/postpartum visit for newborns and mothers. All analyses were performed with SAS 9.4

**Results**

Newborns of mothers who attended group antenatal care were significantly more likely to attend the 6-7 day postnatal visit than newborns of mothers receiving routine individual care (81.55% vs. 77.44%; *p*= 0.05). There were no significant differences between control and intervention groups for the 6-week newborn postnatal visit (*p*=0.3), for mothers attending the 6-7 day (*p*=0.07) or 6 week postpartum visit (*p*=0.10).

**Conclusions**

Postnatal care provides a critical opportunity to examine the newborn for danger signs—including difficulty breathing, poor feeding, lethargy, fever, and jaundice—and to counsel mothers to return if danger signs are observed. Postnatal care also provides an avenue to educate mothers on vital topics such as newborn health, nutrition, and early childhood development. Newborns of mothers who attended group ANC were more likely than newborns of women who attended routine, individual care to attend postnatal care at 6-7 days. Group ANC had no impact on whether or not women attended postpartum care.

## O163. A new tool for measuring facility-level infection prevention and control and the impact on Maternal and Newborn Health (MNH) in a humanitarian setting

### Atoo Mercy Otika

#### International Rescue Committee

*BMC Proceedings 2024*, **18(5):**O163

Submission ID #: IMNHC993

**Background**

To lower incidence of hospital-acquired infections, a minimum threshold for nosocomial infection prevention and control (IPC) and an adaptable tool for measuring precautions are critical to improving maternal and neonatal mortality. To improve IPC across IRC-supported health facilities globally, the IRC adapted the WHO/UNICEF WASHFIT tool to capture and evaluate standard IPC measures, flag issues for action, and monitor improvements to identify best practices.

**Methods**

The WASHFIT tool was revised for COVID-19 and primary level care across all countries. The revised tool was piloted and built in CommCare, a mobile data platform.The tool was made available in four languages—English, French, Spanish, and Arabic—and 200+ staff were trained to use it. Ultimately, 1,100+ primary health centers and hospitals in 23 countries were assessed. A real-time dashboard for visualization of results was created using PowerBI and made accessible to both IRC and facility staff. Facilities were assessed up to three times, allowing for comparative analyses of performance over time and for cross-comparison between facilities.The tool assessed facilities, including maternal and newborn health (MNH) services, across five categories: screening, cleaning and PPE, water, sanitation, and management. A threshold of 80% was set for meeting minimum global IPC standards.

**Results**

Comparative analyses showed an increase in facilities that met the 80% threshold over time, including a 9% increase between the second and third rounds,implying an improvement in quality of care (including MNH).The greater improvements were noted in screening/triage, sanitation, and management and in Asia, Great Lakes, and MENA regions, with slower progress noted in cleaning/PPE and water supply and in East and West Africa. Staff have identified several ongoing barriers to improving IPC with implications for MNH: lack of sufficient funding for IPC staff, conducting regular assessments, or initiating expensive infrastructure improvements.

**Conclusions**

Results from the assessments continue to highlight the need for ongoing advocacy for IPC budgeting and funding, ongoing refresher trainings on IPC, and monitoring to maintain and improve minimum standards.As disease outbreaks and COVID continue to contribute to higher mortality rates, the MNH community needs to place greater emphasis on IPC and infrastructure, particularly in humanitarian setting where risks are higher.

## O164. Use of story-based education videos for breastfeeding counselling in primary health care facilities in the Western Cape of South Africa

### Nophiwe Job^1^, Liezel Engelbrecht^1^, Kira-Leigh Kuhnert^1^, Jamie Johnston^2^

#### ^1^Digital Medic South Africa; ^2^Stanford Center for Health Education

*BMC Proceedings 2024*, **18(5):**O164

Submission ID #: IMNHC990

**Background**

To successfully promote and support breastfeeding, the World Health Organization (WHO) recommends investment in breastfeeding counselling. As mHealth interventions incorporating entertainment-education approaches have gained traction in the training of health workers, Digital Medic, an initiative of the Stanford Center for Health Education, created a series of 14 short (two to five minutes) story-based videos focused on infant feeding and nutrition, available in English, isiXhosa, and Afrikaans. This study aims to understand how these videos were used by breastfeeding counsellors in public health institutions, following usage over a three-year period. Our exploration draws on key constructs of the technology acceptance model, i.e., perceived and actual usefulness and ease of use.

**Methods**

Tablets preloaded with videos were provided to a conveniently-selected sample of breastfeeding counsellors and dietetics students (*N*=15) working in breastfeeding promotion across 10 primary health care facilities in the Southern/Western District, Western Cape in South Africa. Counsellors were instructed to use videos voluntarily at their own discretion. Data are being collected in two phases. In Phase 1, we conducted two focus group discussions with counsellors, three months after tablets were delivered. As counsellors are still actively using the tablets, we are now conducting a Phase 2, long-term set of focus group discussions in 2023 (three years following introduction of the tablets) and analysing video platform analytics to better understand use of the educational content in counselling sessions over time.

**Results**

At Phase 1, videos were being used regularly in counselling sessions. Videos diffused the initial apprehension and awkwardness associated with firsttime counselling interactions and encouraged discussions. The videos bridged language barriers between mothers and counsellors and were relatable, positive, and understandable, particularly when watched in native languages. Deterring factors for use included the initial fear of using technology and the need to ensure that devices were fully charged daily.

**Conclusions**

The intervention seemed well adopted for the counsellors’ work routine in the short term. However, it would be beneficial to understand whether the breastfeeding counsellors still perceived the videos to be useful in their work beyond their initial novelty. We aim to report insights from Phase 2 in May 2023.

## P165. Comparing Algorithm- and Clinician-Assigned Causes of Under-Five Deaths Using Verbal Autopsy in Kara, Togo

### Attisso Komlan Désiré Dabla^1^, Komivi Badohoun^1^, Hyacinthe Awizi^2^, Laté Lawson-Ahluivi^1^, Emily Hansman^3^, Reise Sample^4^, Jessica Haughton^1^

#### ^1^Integrate Health; ^2^Médecins Sans Frontières Suisse; ^3^David Geffen School of Medicine; ^4^University of Washington

*BMC Proceedings 2024*, **18(5):**P165

Submission ID #: IMNHC979

**Background**

The World Health Organization (WHO) recommends the use of verbal autopsies (VA) to ascertain causes of death (CODs) in low- and middle-income countries (LMICs). Togo, like many LMICs, does not have complete registration and vital statistics systems to determine COD and set health priorities targeted at reducing mortality. Our objective is to test the validity of three VA algorithms in northern Togo.

**Methods**

The 2016 WHO VA instrument was translated into French with geographical information for Togo added. We reviewed deaths of nine children under 5 years occurring between February and July 2022 in northern Togo, for whom no COD had been determined. A trained researcher accompanied by local community health workers’ supervisors conducted face-to-face interviews with kin of the deceased. Three VA analysis algorithms —InterVA, InSilicoVA, and Tariff 2.0— were used to determine COD for each of nine deaths. Three clinicians independently reviewed VA data to determine a clinician-assigned COD and assess face validity. CODs were coded to match the WHO 2022 Cause of Death List for Verbal Autopsy to allow comparison. Cohen’s Kappa was used to assess inter-rater reliability both among clinicians and between each instrument and clinicians. Sensitivity and specificity of each instrument were assessed for the most common CODs.

**Results**

There was moderate agreement among clinicians (kappa=0.46) on COD. Tariff 2.0 had the best performance with substantial agreement with clinician-assigned COD (kappa=0.61). InterVA and InSilicoVA had moderate agreement (kappa=0.35 and kappa=0.36 respectively) with clinician-assigned COD. Sensitivity and specificity varied by COD and by instrument. Among the most common CODs, Tariff 2.0 had high sensitivity and specificity for diarrheal disease and respiratory infection and lower sensitivity for birth asphyxia and neonatal sepsis.

**Conclusions**

Tariff 2.0 had the best performance of three VA analysis algorithms when compared to clinician-assigned COD. Several studies in the literature have also reported a better reliability of Tariff 2.0, though this varies by context. In view of these elements, we plan to launch a broader pilot with Tariff 2.0 in order to determine feasibility of wide-spread use in northern Togo.

## O166. Closing the gap of uncounted children born in facilities globally: literature review and qualitative research

### Masudah Paleker^1^, Dorothy Boggs^2^, Debra Jackson^2^, Louise Tina Day^2^, Joy Lawn^2^

#### ^1^Western Cape Department of Health, South Africa; ^2^London School of Hygiene & Tropical Medicine

*BMC Proceedings 2024*, **18(5)**:O166

Submission ID #: IMNHC976

**Background**

Identity registration is crucial in providing children with important rights including access to health care and education as well as a legal identity and access to other essential services. Globally, approximately 166 million (just under 25%) children under five are unregistered, yet more than 80% of births in most low- and middle-income countries (LMIC) occur within health facilities. There is a gap between high rates of facility births and low birth registration rates, which highlights the opportunity for facility-based initiatives to address this gap. This study, conducted in association with UNICEF, reviews facility-based birth registration initiatives and provides recommendations to close this gap between high rates of facility births and lower birth registration rates in LMIC.

**Methods**

A literature review of academic and grey literature was conducted to identify facility-based initiatives to improve birth registration rates and address barriers that prevent birth registration. For further insight, six semi-structured in-depth interviews with key stakeholders were conducted by audio call to further identify initiatives and enablers and barriers to birth registration and provide recommendations on closing the gap.

**Results**

Through a literature search of academic and grey literature, 21 studies met the pre-determined inclusion criteria. Barriers preventing birth registration were identified and grouped into three themes: health system, governmental, and societal. Findings suggest that birth registration initiatives implemented at facility level have been successful in increasing birth registration rates. Effective initiatives involved intersectoral collaboration between civil registration ministries and the health sector by placing civil registration offices within health facilities or allowing medical doctors to act as registrars. In addition, health promotion within communities also increased the demand for birth registration. The interview respondents supported the data found in the literature review and provided a further depth of understanding.

**Conclusions**

Low birth registration rates in LMICs can be increased through the use of facility-based birth registration initiatives. For birth registration rates to improve, initiatives need to address both supply and demand side for birth registration. Key to success is intersectoral collaboration within governments and strategic alignment with multiple global and local stakeholders.

## O167. Afghan Locally Lead Emergency Nutrition (ALLEN): a case study in humanitarian settings

### Alessandro Iellamo

#### FHI 360

*BMC Proceedings 2024*, **18(5):**O167

Submission ID #: IMNHC969

**Background**

Following 40 years of war and an already dire situation of increasing hunger, economic decline, price increases for food and essential items, and rising poverty, Afghanistan faced intensified conflict, the withdrawal of international forces, and eventually the takeover by the Taliban. In 2022, the deteriorating context resulted in 24.4 million people with humanitarian needs and 4.7 million children and women suffering from malnutrition. In response, FHI 360 engaged the Nutrition Cluster to identify the needs and mobilized organizational funds to start responding. FHI 360 partnered with national nongovernmental organizations (NGOs) to strengthen their capacity to deliver nutrition services for women and children. This response is the ALLEN initiative.

**Methods**

FHI 360 partnered with two national NGOs, CAF and JACK, to implement maternal and infant nutrition (MIYCN) in the provinces of Kabul and Logar in 20 health facilities. FHI 360 and partners collaboratively designed the interventions that commenced with assessing the NGOs MIYCN capacity. The findings were used during three workshops with the implementers to plan activities to improve MIYCN services. The fourth workshop was with community agents and focused on designing interventions to increase access to treatment for malnourished children.

**Results**

Between April and September 2022, 160 field workers were engaged in the activities; more than 50% were female. Over the same period, 51 high-risk villages and 5,200 households were reached by field workers with MIYCN services. All 20 health facilities established IYCF counselling services. A total of 4,110 children under five years of age were screened for malnutrition and 542 were admitted for treatment.

**Conclusions**

The FHI 360 ALLEN initiative helped operationalize localization aspirations in humanitarian and fragile settings by supporting national NGOs to improve and expand the provision of MIYCN services. ALLEN is a strategic example where localization, diversity, equity, and inclusion efforts were effectively integrated by advocating for female participation as field workers. The experience confirms the importance of investing in national NGOs and local communities to provide MIYCN services in humanitarian and fragile contexts.

## O168. Optimizing Pre-discharge care: development and testing of a postnatal screening and counselling tool to identify high-risk mothers and babies

### Syeda Nabin Ara Nitu^1^, Melanie Yahner^1^, Sarah Elaraby^2^, Victoria Lwesha^1^, Imran Hossain^1^

#### ^1^Save the Children; ^2^University of Alexandria

*BMC Proceedings 2024*, **18(5):**O168

Submission ID #: IMNHC956

**Background**

In Bangladesh, rising institutional delivery rates offer opportunities to optimize pre-discharge counselling and screening to identify needed follow-up for those with complications and encourage continuity of care including postnatal care (PNC). Few existing resources are available to guide pre-discharge counselling and to identify care needed for high-risk mother-baby dyads.

**Methods**

We adapted a pre-discharge checklist to include an algorithm for screening risk factors among mothers/babies and to guide providers to counsel, refer, or delay discharge based on identified risk. To inform the algorithm, a scoping review identified risk factors for the major causes of maternal/neonatal mortality and underlying risk factors for non-use of PNC. Published, peer-reviewed studies conducted in LMICs within the last 10 years that tested for association between risk factor and outcome were included. During small-scale implementation, we gathered feedback over 12 months through pause-and-reflect sessions with providers and mothers, routine monitoring, and supervision visits.

**Results**

The review identified clinical and non-clinical factors predicting poor postnatal outcomes. Proximal factors include age (under 19, over 35), parity (primigravida), previous history (postpartum hemorrhage, pre-eclampsia) and marital status, and distal factors including household socio-economic status, education, urban/rural residence. Small-scale testing in Bangladesh identified promising findings and areas for refinement. Most mothers who delivered in facilities and received pre-discharge counselling reported obtaining clear guidance about PNC. Providers felt that the checklist was useful, with value in providing written documentation of mother/baby status at discharge. Heavy workload, busy discharge times, short facility stays, and complex referral cases resulted in de-prioritization of checklist utilization.

**Conclusions**

A pre-discharge screening and counselling tool can optimize facility birth and advance equity by guiding providers to prioritize mother-baby dyads with clinical and non-clinical risk factors. Our findings show that incorporating risk screening into pre-discharge counselling is acceptable to mothers and providers. Longer term efforts are needed to improve staffing levels, strengthen referral systems, and encourage longer facility stays. While clinical factors can be clearly linked to needed provider actions, non-clinical risks (e.g., age, socio-economic status) were challenging to operationalize.

## O169. Breastfeeding counselling: a qualitative evidence synthesis on the views of women and health workers on timing, frequency, mode, and preferred providers

### Emma Sacks^1^, Anne Batchelder^2^, Sakshi Jain^3^, Tessa Moll^4^, Elizabeth Bastias-Butler^5^

#### ^1^Johns Hopkins Bloomberg School of Public Health; ^2^Johns Hopkins University School of Nursing; ^3^Wilfrid Laurier University; ^4^University of Witwatersrand; ^5^Inter-American Development Bank

*BMC Proceedings 2024*, **18(5):**O169

Submission ID #: IMNHC953

**Background**

Breastfeeding counselling, in which a healthcare workers provides individual or family support to improve practices and manage challenges, is an important strategy to improve breastfeeding initiation and continuation. The purpose is to review how breastfeeding counselling is experienced by women and health workers

**Methods**

A qualitative evidence synthesis of factors relating to the uptake of breastfeeding counselling was undertaken. Databases searched were: Medline, Embase, CINAHL, PsycINFO, Global Health Ovid, Global Index Medicus, Cochrane Library, Web of Science, and the International Clinical Registry Platform. About 8,800 studies were screened for inclusion. Eligible papers were included based on inclusion criteria of qualitative studies that addressed counselling, excluding those that identified problems alone. The papers were assessed for quality and data were extracted. Findings were categorized into themes, stratified by respondent, and assigned confidence levels based on the GRADE-CERQual method. The protocol was registered in the PROSPERO database.

**Results**

Sixty-five papers were included, representing 18 countries. Women considered breastfeeding counselling helpful, but wanted to feel more prepared and supported. Both women and providers agreed that more time and more follow-up visits are needed. Women differed in their preferences for when, how, and who gave breastfeeding counselling and therefore preferred personalized counselling. Continuity of care is needed; interacting with different health workers, conflicting advice, and rapport were challenges for breastfeeding support. Underrepresentation of low- and middle-income countries is a limitation.

**Conclusions**

Breastfeeding counselling is valued but could be further improved to increase breastfeeding rates and the experience of women.

## O170. Experiences with implementing an essential package of neonatal care and reporting system in the public hospitals of KwaZulu-Natal, South Africa, 2018-2022

### Ruth Davidge

#### KwaZulu-Natal Department of Health

*BMC Proceedings 2024*, **18(5):**O170

Submission ID #: IMNHC947

**Background**

In 2015, an external audit of neonatal care was conducted in all 51 public hospitals of the KwaZulu-Natal (KZN) Province, South Africa, revealing varying levels of quality of care and mortality rates. To ensure equitable access to an appropriate standard of neonatal care and service delivery platform, and to embed a system of self-assessment and quality improvement in all hospitals, the Provincial Department of Health (DOH) developed an essential package of neonatal care (EPOC).

**Methods**

Through a review of existing resources and using a consultative process, the DOH task team developed tools to monitor and evaluate factors relating to infrastructure, systems, and care. EPOC tools included evaluation checklists, skills and clinical record audits, maternal interviews, standardized clinical records, and systems resources. Tools were rolled out to all hospitals from September 2017 to December 2018 after facility orientations. Trained facilitators within DOH supported implementation. Reporting commenced in April 2019 using a monthly facility dashboard of 41 indictors sent quarterly to the provincial DOH and shared with facility managers. Experiences with implementation were assessed through a structured questionnaire, facilitators’ reports, and verbal feedback.

**Results**

Implementation of EPOC has improved slowly. In June 2019, 20 hospitals submitted quarterly dashboard reports with an average of 74%; in June 2022, 47 hospital reported quarterly with an average of 81%. Scores ranged between 36%–90%. Despite initial complaints that the tools were overwhelming with the limited staffing, they are now well accepted. Communicating scores has encouraged compliance and healthy competition between hospitals. Facility staff have a greater appreciation of the roles and responsibilities of team members through use of the tools. Clinicians express an increased sense of confidence in clinical practice. Implementation barriers include competing provincial priorities, inadequate leadership and accountability, computer and printing challenges, demoralisation, and inadequate buy-in from all role players. Enablers include strong facilitator support, individual capacity, scheduling and ownership of audits, and observing tangible changes due to EPOC.

**Conclusions**

There has been overall success with EPOC scale-up signalling the value of standardized clinical governance systems to ensure equitable access. Further research is required to assess the impact of these systems.

## P171. Community health volunteers’ impact on the quality of maternal and newborn health in Kenya

### Winnie Owade^1^, Niharika Jha^1^, Lincoln C. Pothan^1^, Priyanka Shrestha^1^, Eunice Sijenje^1^, Evelyn Waweru^2^, Violet Apondi^3^, Mercy Amulele^2^, Tiara Ranson^1^, Agatha Olago^4^, Arianna Rubin Means^1^, Bryan Weiner^1^, Beatrice Wasunna Wasunna^2^, Ferdinand Mukumbang^1^, John Kinuthia3, Keshet Ronen^1^

#### ^1^University of Washington; ^2^Medic; ^3^Kenyatta National Hospital; ^4^Kenya Ministry of Health

*BMC Proceedings 2024*, **18(5):**P171

Submission ID #: IMNHC941

**Background**

Access to quality and timely care for peripartum women (PPW) and newborns and positive care-seeking behavior are critical to improve maternal and newborn survival. In Kenya’s health system, community health volunteers (CHVs) encourage, remind, refer/accompany PPW to the health facility and provide maternal and child health related health education, among other roles. This formative qualitative analysis was conducted as part of the CHV-NEO research study (NCT05187897). We aimed to elicit stakeholders’ perspectives and feedback on the impact of CHVs on maternal and newborn health.

**Methods**

Between April and August 2022, 16 key informant interviews with health workers and policy-makers and 12 focus group discussions (FGD) with CHVs and PPW were conducted in western Kenya. Interviews were facilitated by trained Kenyan social scientists using semi-structured guides in participants’ preferred languages (English, Kiswahili, or Dholuo). They were audio recorded, transcribed, and translated into English. Thematic analysis was used to identify stakeholders’ perspectives on the impact of the CHV program, experiences, and recommendations for future use.

**Results**

Stakeholders relayed the importance and positive impact of CHVs in health care service delivery, particularly key maternal and child health indicators, including skilled delivery and antenatal/postnatal care. Stakeholders found CHVs helpful in improving the health and knowledge of PPW, including strategies for identifying newborn and antenatal danger signs and appropriate interventions. Stakeholders felt that CHVs facilitate improved uptake and access to skilled delivery and antenatal/postnatal care through education during home visits and create trust and bridge gaps between communities and the health system. CHVs expressed their motivations to work included their impact and skills building through training. Other stakeholders stated monthly stipend and recognition from the community and health system as other CHV motivations. Stakeholders recommended additional training to increase capacity and motivate CHVs and integration of digital health platforms to ensure extensive community coverage and enhance communication.

**Conclusions**

Stakeholders perceived that CHVs improve maternal and newborn health in Kenya through health education and referrals, by addressing barriers to timely health care services and facilitating positive care-seeking behavior among PPW. Providing and sustaining CHVs’ motivation through service improvement, such as digitalization and integration, can improve their performance.

## O172. Estimating COVID-19 pandemic effects on maternal mortality

### Jennifer Faith, Michael Arndt, Nicholas Kassebaum, Maegan Dirac

#### Institute for Health Metrics and Evaluation, University of Washington

*BMC Proceedings 2024*, **18(5):**O172

Submission ID #: IMNHC939

**Background**

The Global Burden of Disease (GBD) 2021 estimated around 15 million COVID-19 deaths and 4.5 million other pandemic-related deaths globally in 2020 and 2021. The effect of the pandemic on excess maternal mortality requires further exploration and this study builds upon the GBD 2021 to estimate this effect.

**Methods**

We estimated excess maternal mortality using two approaches. First, we used a population attributable fraction approach, which utilized GBD estimates of COVID-19 deaths and pregnancy prevalence to estimate the number of COVID-19 deaths in people who were pregnant. We then estimated the proportion of these deaths attributable to the pregnancy by applying an attributable fraction estimated from literature on the relative risk of death in pregnant versus non-pregnant COVID-19-positive women of reproductive age. Second, we used an excess mortality approach, which utilized processed vital registration data for direct and indirect maternal deaths separately for 30 countries from 2000–2020 and GBD-estimated COVID-19 mortality rates. Maternal mortality rates were used as inputs in meta-regression, Bayesian, regularized, trimmed, with geographic cascading splines for the random effect of time, and a fixed effect for COVID-19 mortality, to predict excess indirect maternal mortality for observed and counterfactual COVID-19-free scenarios. The ratio of observed versus counterfactual predictions was then modeled as a function of COVID-19 mortality for all locations and applied as correction factors to adjust GBD 2021 indirect maternal death estimates.

**Results**

No effect was found for COVID-19 and excess mortality from direct maternal mortality. Globally, the population attributable fraction and excess mortality approaches estimated approximately 1,650 and 5,000 excess indirect maternal deaths, respectively, in 2020 and 2021 combined. Excess mortality results indicated the largest percentage increase in indirect maternal deaths were in Andean Latin America and Central Latin America in 2020 and southern sub-Saharan Africa and Andean Latin America in 2021. The largest estimates for excess indirect maternal deaths in 2021 were in India and Ethiopia.

**Conclusions**

The COVID-19 pandemic has negatively impacted maternal mortality by increasing indirect maternal deaths globally by around 8% in 2020 and 17% in 2021, raising concerns about the continued impact of the pandemic on maternal health.

## O173. Enabling Traditional Birth Attendants (TBAs) as community-level first responders to advance maternal and neonatal survival in Northern Nigeria

### Alana Garvin, Farahat Bello, Funlola Osinupebi, Badiya Magaji, Desmond Ogunbor, Remilekun Peregrino, Owens Wiwa, Olufunke Fasawe

#### Clinton Health Access Initiative

*BMC Proceedings 2024*, **18(5):**O173

Submission ID #: IMNHC930

**Background**

Globally, Nigeria contributes the greatest number of maternal and second greatest number of neonatal deaths, and the third greatest number of stillbirths. In northern Nigeria, most births occur at home and are often assisted by untrained TBAs. To ultimately accelerate impacts on maternal and newborn survival, we aimed to improve the knowledge, skills, and confidence of TBAs.

**Methods**

We evaluated a program on training and equipping TBAs from July 2020 to August 2021. In Kaduna and Katsina, 212 TBAs were allocated to two equal-sized arms: equipped and unequipped (standard of care). Both arms were trained on identification, emergency management, and referral for pregnancy and childbirth danger signs. TBAs in the equipped arm were supplied with essential equipment and commodities to prevent and stabilize complications. A difference-in-difference analysis was used to compare unequipped and equipped knowledge acquisition, retention, and confidence, assessed pre/post-training and +12 months. Using case scenarios and checklists, equipped TBAs’ skills to use commodities and equipment were observed post-training and +12 months and analyzed using paired t-tests.

**Results**

Within each arm, mean TBA knowledge scores significantly increased from pre- to post-training and 12 months post-training (unequipped: 25%, 77%, 71%; equipped: 18%, 67%,74%; [*p*< 0.001]). Pre- to 12-months post-training, difference-in-difference results showed the mean knowledge of equipped increased 10% more than unequipped (*p*< 0.001). For equipped, mean skills scores were high post-training with some loss at 12 months (misoprostol administration: 90% to 85%, *p*=0.042; non-pneumatic anti-shock garment application: 92% to 87% *p*=0.078; newborn resuscitation--suction bulb use: 95% to 83%, *p*< 0.001; ambu bag use: 80% to 75%, *p*=0.207). Pre- to 12-months post-training, TBA confidence significantly increased across all 19 activities within each arm (*p*< 0.05), but difference-in-difference found no significance between arms.

**Conclusions**

Results indicate TBAs can learn and retain knowledge to identify danger signs in pregnancy and be safely trained and equipped with essential commodities and equipment to act as first responder. By improving TBA knowledge, skills, and confidence, TBAs will be able to 1) identify pregnancy danger signs and refer women for facility deliveries, 2) prevent childbirth complications, and 3) stabilize women and newborns experiencing complications before facility referral.

## O174. Strengthening leadership and governance for maternal and perinatal death surveillance and response in a low-income urban setting: experiences from Kampala, Uganda

### Martin Kasendwa

#### FHI 360

*BMC Proceedings 2024*, **18(5):**O174

Submission ID #: IMNHC927

**Background**

Robust leadership at all levels and a culture of accountability are key factors for the successful implementation and sustainability of maternal and perinatal death surveillance and response (MPDSR). The U.S. Agency for International Development Maternal Child Health and Nutrition (MCHN) activity supported Kampala Capital City Authority (KCCA) to establish and functionalize a citywide MPDSR committee to conduct confidential inquiries on select maternal and perinatal deaths and ensure timely implementation of agreed-upon actions.

**Methods**

MCHN supported KCCA to establish a multidisciplinary Kampala regional MPDSR committee in March 2022, comprising 17 core members from referring and referral sites, medical bureaus, implementing partners, and the private sector; oriented the Committee on MPDSR processes and terms of reference; and provided weekly analytics, coordination, and logistics support for the convening of weekly City-wide MPDSR accountability meetings. In April 2022, MCHN supported the Committee to establish a clinical mentorship hub-and-spoke model, which factored in proximity and functional capacity for emergency obstetric and newborn care to strengthen capacity from tertiary to lower-level health facilities. Since then, the Committee has held weekly data-driven meetings to review deaths, develop recommendations to prevent future deaths, and track their implementation.

**Results**

The percentage of maternal deaths reviewed in Kampala increased from 79% (October to December 2021) to over 91% (January – March 2023), and the percentage of perinatal deaths reviewed increased from 58% (October to December 2021) to 81% (January – March 2023). The committee has strengthened governance and response in MPDSR for private and public facilities through accountability letters to facility management to trigger action and improved coordination and alignment of priorities among maternal and newborn partners. Resulting actions include enhanced referral coordination, and fast-tracked recruitment of 2 medical and 5 anesthetic officers by KCCA to ensure 24-hour Comprehensive Emergency Obstetric and Neonatal Care functionality and procurement of 7 new ambulances by KCCA, and galvanized public-private partnerships for health through the “Corporate Society for Safe Motherhood” platform.

**Conclusions**

Strengthening the leadership and governance has improved MPDSR reviews and response in Kampala. However, to significantly reduce the high numbers of maternal and perinatal deaths in the city, this work needs to extend to the nearby districts of Wakiso and Mukono from which the most mothers and newborns who die in Kampala are referred.

## O175. Population segmentation as a tool to identify vulnerable populations and prioritize women’s health programs

### Elisabeth Root^1^, Tracy Johnson^1^, LinChiat Chang^2^, Steve Kretschmer^3^, Melanie Wendland^4^, Allie Gugliotti^2^, Helen Olsen^1^, Claire-Helene Mershon^1^

#### ^1^Bill & Melinda Gates Foundation; ^2^Independent Consultant; ^3^Desire Line; ^4^Sonder Collective

*BMC Proceedings 2024*, **18(5):**O175

Submission ID #: IMNHC917

**Background**

Encouraging facility-based delivery to reduce maternal and neonatal mortality has been a global health priority for decades. The proportion of women who deliver in health facilities has steadily increased in every region of the world, though gains have not been uniform. In many low- and middle-income countries, women are accustomed, and often prefer, to deliver at home. Identifying what makes certain groups vulnerable and understanding why women deliver at home is critical for customizing interventions to maximize impact. Population segmentation is a tool that allows us to categorize populations into distinct subgroups based on multiple dimensions of vulnerability so we can identify, prioritize, and target specific needs.

**Methods**

We used a mixed-methods approach to develop population segments based on bio-psychosocial, economic, environmental, and structural vulnerabilities faced by women and their families. A household survey was conducted in Kenya (*N*=4,174). K-means and K-medoid (PAM) clustering were applied to the survey data to divide households into segments with similarities across multiple variables measuring different dimensions of vulnerability. Multivariate regression models were used to examine the probability of home birth across population segments and underlying vulnerabilities driving both women’s preference for home delivery and home birth.

**Results**

Population segments were highly predictive of home birth (84% accuracy; 95% CI: 80.5-86.3), suggesting they capture key vulnerabilities driving place of delivery. Multivariate analyses showed that preference for a home delivery was the single largest predictor of home birth (OR=18.1; 95% CI: 8.8-37.2). Furthermore, trust in doctors, active engagement across the health system, the means to access health care (transportation, communication, cost), and supportive household environments all reduced probability of home birth.

**Conclusions**

Women’s decisions about where to give birth, and their ability to access facility-based care, are influenced by a complex set of cultural, social, economic, environmental, and structural factors. Population segmentation is a tool that simplifies these factors so we can more easily identify, prioritize, and target the needs of vulnerable populations; it can be applied to any data set that includes vulnerability variables. Additional analyses elucidate specific vulnerabilities that can inform programmatic approaches to reducing poor health outcomes.

## O176. Improving governance of Maternal and Newborn Health (MNH) programming through co-creation: a case of nine devolved county governments in Kenya

### Lilian Mutea^1^, Wangui Muthigani^1^, Peter Kaimenyi^1^, Evans Osembo^1^, Yvonne Musa^2^

#### ^1^United States Agency for International Development; ^2^Ministry of Health, Kenya

*BMC Proceedings 2024*, **18(5):**O176

Submission ID #: IMNHC915

**Background**

Kenya ushered in a devolved system of governance that created 47 semi-autonomous county governments in 2010. Delivery of health services was the major component transferred to the county governments while the national government retained policy and regulatory functions. While there has been improvement in health structural development attributed to devolution, counties continue to experience insufficient management and technical staff capacity to conduct devolved tasks.

There have been substantial investments in capacity building for human resources for health and equipping facilities for maternal and newborn health (MNH) in the last decade. Subsequently, access to antenatal care, facility deliveries, and postnatal care has significantly improved. However, there remains mixed results in measures of impact for maternal morbidity and mortality. We present results on the effect of utilizing a co-creation process to improve MNH service delivery and outcomes in nine counties in Kenya.

**Methods**

Using the U.S. Agency for International Development health systems approach to implement the reproductive, maternal, newborn, child, and adolescent health program, participatory consultations involving reflections on specific county MNH priorities, services delivery data, and annual operation plans were conducted. The aim is to align the Ministry of Health policies and strategies to the county governments plans for improved governance and accountability. Jointly with the county governments departments, community, and local implementing partners, practical solutions were co-created addressing socio-demographic MNH health determinants. Consensus was built on joint funding for integrated health plans with measurement indicators and feedback loops through technical working groups.

**Results**

County governments demonstrated improved competence in leadership to manage devolved health functions. There is strengthened collaboration between diverse stakeholders. County health departments have made conscious efforts to increase domestic financing and adopt local sustainable solutions to MNH interventions. There is also heightened social accountability mechanisms by community members who hold county governments responsible for delivery of quality MNH programs. There is demonstrable improvement in MNH indicators across the board and improved quality of services.

**Conclusions**

County health leadership decision-making abilities may be strengthened through co-creation approaches since they provide for authentic innovation in addressing local challenges. While unpredictable and disruptive, it should be embraced in all program development and implementation processes to improve governance results.

## P177. Systematic identification and differential expression profiling of preterm birth associated plasma ncRNAs in Low- and Middle-Income Countries (LMICs) cohort

### Waqasuddin Khan, Javairia Khalid

#### The Aga Khan University

*BMC Proceedings 2024*, **18(5):**P177

Submission ID #: IMNHC909

**Background**

Preterm birth (PTB), defined as delivery < 37 weeks of gestation, occurs in 11.1% of pregnancies worldwide, and is associated with significant neonatal morbidity and mortality. The objective of this study was to identify differentially expressed ncRNAs expression in whole blood samples of pregnant women enrolled in the AMANHI biorepository cohort between term (>37 weeks of gestation) and PTB.

**Methods**

Sequencing data were pre-processed using sRNAnalyzer and aligned to multiple human RNAs and ncRNAs databases. We removed RNAs and ncRNAs with 0 read counts in > 50% of samples, or a mean read count of < 20 producing a final dataset. Significantly associated differentially expressed RNAs and ncRNAs with PTB was identified if they exhibited a Benjamin-Hochberg adjusted q value of < 0.05 and a log2 fold change of >1 or <-1. Putative ncRNA gene targets were detected using the quantitative model by TargetScan, which has the best predictive performance compared with the other comparable tools. ncRNA target-gene relationship was identified in controls and cases by examining the correlations between each ncRNA, and its proposed target genes. Gene set enrichment analysis was performed for mRNA targets using two-sided hypergeometric tests conducted on Gene Ontology biological process gene sets using Gene Ontology gene set visualization application ClueGO within the Cytoscape environment. We also compared the expression of ncRNAs in preterm controls and cases to previously published data.

**Results**

17 ncRNAs were identified that may play a role in the identification of pregnant women who deliver prematurely; serving as an important foundation for subsequent analyses. Biological pathways were annotated for up- and down-regulated genes.

**Conclusions**

This work highlights the importance of discovering important biomolecular mechanisms by which ncRNAs need to be functionally validated in independent cohorts of pregnant women on key tissues, such as, placenta and myometrium.

## O178. Using machine learning to determine the association of maternal characteristics and maternal serum-related biomarkers with newborn outcomes

### Samiah Kanwar; Javairia Khalid, Farrukh Qazi

#### The Aga Khan University

*BMC Proceedings 2024*, **18(5):**O178

Submission ID #: IMNHC905

**Background**

In 2018, around 40% of children under 5 years of age were stunted in South Asia, which makes the prevalence of stunting higher in this region than others worldwide. Stunting contributes towards poor health outcomes later in life and is strongly correlated with impaired cognitive development. In most cases, stunting starts in utero, which is why prenatal identification of children at risk for stunting at birth is crucial. The aim of this study was to identify the maternal characteristics and maternal serum biomarkers that are strong predictors of stunting at birth.

**Methods**

Height-for-age, weight-for-age, and weight-for-height z-scores and presence of small for gestational age (SGA) of children at birth were obtained from two peri-urban sites in Karachi. Maternal characteristics and maternal serum biomarkers were measured at the 24–28 weeks pregnancy visit. A descriptive analysis of maternal clinical factors and neonatal growth measurements was performed. Random forest model was used to assess the importance of different maternal characteristics and maternal serum biomarkers in predicting stunting, wasting, underweight, and SGA at birth.

**Results**

Of the 1,059 children, 29.7% children were SGA at birth with no difference between genders. About 20.9% children were stunted, with more males than females. Nearly 12.2% were observed as wasted and distribution of wasting across gender was slightly more for males than females. Only 22.8% of studied population were under weighted with distribution for under weight slightly more in male children than female children. The random forest classifier identified mother’s age, mid upper arm circumference and body mass index as the strongest predictors for stunting, wasting, underweight, and SGA at birth. Of the 12 biomarkers, placental growth factor was identified as the strongest predictive maternal serum biomarker for all four phenotypes. The model predicted the growth outcome with 76% accuracy.

**Conclusions**

We demonstrated that maternal characteristics and systemic pro-inflammatory and inflammatory maternal serum biomarkers are associated with stunting, wasting, underweighting, and SGA at birth. Biomarkers such as C-reactive protein, ferritin, and pregnancy related hormones such as placental growth factor and SFLT are strong predictors of malnourishment birth.

## O179. Centers of excellence strategy improves the provision of high-quality reproductive, maternal, newborn, and child health care across the community to hospital continuum

### Desselew Emaway^1^, Biruhtesfa Shiferaw^1^, Nebreed Fesseha^2^, Gizachew Tiruneh^2^, Wuleta Betemariam^1^

#### ^1^John Snow, Inc.; ^2^JSI Research & Training Institute, Inc.

*BMC Proceedings 2024*, **18(5):**O179

Submission ID #: IMNHC897

**Background**

Between October 2017 and June 2021, JSI Research and Training Institute Inc., the Last Ten Kilometers (L10K) project supported four regions in Ethiopia to establish one center of excellence (CoE) per region that comprises general and primary hospitals and primary health care units and all health posts under the selected primary health care units. The CoE is a model service delivery site and learning hub demonstrating high-quality reproductive, maternal, and newborn health RMNCH) clinical care provision and community engagement with other regions and facilities. To establish the CoE, L10K project, and the health system implemented community engagement activities, mentorship, supervision, training, and established comprehensive RMNCH skills laboratories.

**Methods**

We conducted formative and follow-up operation research using a cross-sectional health facility survey and qualitative study designs to study the implementation strength of CoE initiatives and to design and improve its implementation intensity in April 2018 and October 2019, respectively.

**Results**

The establishment of functional CoE resulted in the strengthening of the community health system, strengthening of functional linkages between facilities across the continuum, improvement of the culture of learning and innovation, and provision of client-centered high-quality RMNCH care. Findings from health posts showed that the implementation of the social accountability system improved from 27% to 94%, the mean percentage of equipment and supplies availability from 47% to 75%, and health posts’ home visit trend from 33% to 71%. The functional facility linkage percentage scores were 91% between health centers and their satellite health posts and 89% between hospitals and health centers. In addition, the health centers’ essential and emergency obstetric and newborn signal functions improved from an average of 5.5 in April 2018 to 6.75 in October 2019. Likewise, all hospitals were fully functional emergency obstetric and neonatal care centers. Furthermore, key informants noted that providers’ skills, confidence, and motivation were enhanced through skill-building initiatives, mentoring, training, and skills lab.

**Conclusions**

The CoE initiative improved the functional linkages between health facilities and the provision of client-centered, high-quality RMNCH care. We also effectively demonstrated the operationalization of CoE strategy in the field. Therefore, we recommend further implementation research to examine the effectiveness and feasibility of the CoE strategy in different contexts to inform its nationwide scale-up.

## O180. Metabolomics of a neonatal cohort from the Alliance for Maternal and Newborn Health Improvement (AMANHI) biorepository: effect of gender, gestational age, weight, and time of sampling on reference intervals

### Javairia Khalid

#### The Aga Khan University

*BMC Proceedings 2024*, **18(5):**O180

Submission ID #: IMNHC893

**Background**

Dried blood spot (DBS) testing for newborn screening (NBS) in Pakistan is not the standard of care and there is non-availability of country specific reference interval. This study was conducted to determine refernce interval and evaluate the effect of preanalytical variables on concentrations of amino acids, acylcarnitines, and succinylacetone from DBS samples of a cohort of neonates enrolled in the AMANHI biorepository, Pakistan.

**Methods**

DBS samples from neonates in peri-urban communities of Karachi, Pakistan, were collected within 48–72 hours of birth. Samples were analyzed for amino acids, acylcarnitines, and succinylacetone on a tandem mass spectrometer (by Waters, Eschborn, Germany) at the University of Iowa. Reference intervals were determined parametrically as analytes were normally distributed. Comparison of analyte distributions across categorical variables such as gender, time of sampling, weight, and gestational age were performed.

**Results**

A total of 610 reference samples were selected based on exclusion criteria; 53.2% being females. The overall mean gestational age of mothers at the time of delivery was 38.7 ± 1.6 weeks; 24.5% neonates were low birthweight and 14.3% were preterm. Out of the total 610 neonates, 23.1% (*n*=141) were small for gestational age. Reference intervals were generated for eleven amino acids, thirty-two acylcarnitines and succinylacetone concentrations in DBS. Markers were evaluated with respect to the influence of gender, gestational age, weight, and time of sampling and statistically significant minimal differences were observed for some specific biomarkers.

**Conclusions**

Reference interval for amino acids, succinylacetone, and acylcarnitine on DBS has been established for healthy neonates from the community, which could be of use in the clinical practice. Reference intervals offer the potential to assist laboratorians and pediatricians in interpreting test results more accurately and thereby lead to improved diagnosis of inherited metabolic disorders and reduced patient risk.

## O181. Strengthening perinatal and neonatal quality of care in Rwandan District and Provincial Government Hospitals

### Lisine Tuyisenge^1^, Julia Battle^2^, Marie Bontoux^3^, Emmanuel Manzi^2^, Clemence Muzard Banes^3^, Hassan Sibomana^4^, Sebastian Taylor^3^, Susan Broster^3^

#### ^1^Rwanda Paediatric Association; ^2^United Nations Children’s Fund; ^3^Royal College of Paediatrics and Child Health; ^4^Rwanda Biomedical Centre

*BMC Proceedings 2024*, **18(5):**O181

Submission ID #: IMNHC891

**Background**

Global and regional progress on maternal and newborn mortality has stagnated. In Rwanda, success in increasing service access revealed shortfalls in quality of facility-based maternal and neonatal care as a key progress obstacle, becoming a national health priority.

**Methods**

Between 2017 and 2022, the Rwanda Paediatric Association, the UK Royal College of Paediatrics and Child Health, and the Rwanda Biomedical Center, supported by UNICEF, delivered a neonatal quality improvement program in 12 hospitals, expanding in 2020 to a perinatal program in 19 provincial and district hospitals and 86 health centers. The program reflects an integrated approach to quality improvement focusing on maternity and neonatal units (NNUs), including (1) in-situ low-dose/high-frequency clinical training for antenatal, intrapartum, postpartum and neonatal care; (2) continuous on-site practical mentorship; (3) service layout, staffing, and management redesign; (4) coaching on medicines/maternal and newborn health equipment use and procurement; and (5) strengthening quality-of-care data for continuous planning, improvement, and case monitoring.

**Results**

In NNUs (2017-May 2022), neonatal mortality fell from 12% to 8.5%, a reduction of 30%, with most reduction in moderately low birth-weight babies. Nurses and doctors passing the “sick newborn scenario” (capacity to assess, recognize and manage the sickest and smallest newborns) increased from 29.2% to 93%. Frequency of newborn observations rose from an average 1.7 to 3.5 times in 24 hours, indicating improvement in nurse-led care. Uptake of kangaroo mother care rose from 65% to 89%. Hypothermia on NNU admission fell from 45% to 6.6%; correct IV fluid administration increased from 61% to 98%. In maternity units (2019-2022), correct labor monitoring rose from 15% to 91%; appropriate administration of uterotonics went from 67.5% to 95.8%; and hypothermia in delivery suite fell from 39.9% to 19%. Importantly, anecdotal evidence suggests communication and coordination between the two units improved.

**Conclusions**

A holistic approach with on-site support can improve quality of care, reducing mortality, even within resource-constrained settings. It is essential to address facility-level issues across the continuum of mother-baby care. Opportunities for future improvement include strengthening community-based care, intensifying respectful maternity care at all levels, streamlining referral between primary and secondary levels, and advancing care for sick/small newborns at referral level.

## O182. Labor augmentation with oxytocin in low- and lower-middle-income countries: a systematic review and meta-analysis

### Monica Kujabi^1^, Emmeli Mikkelsen^2^, Natasha Housseine^3^, Josephine Obel^1^, Brenda Sequeira Dmello^4^, Dan Wolf Meyrowitsch^1^, Kidanto Hussein^3^, Jeppe Bennekou Schroll^5^, Flemming Konradsen^1^, Jos Van Roosmalen^6^, Thomas Van Den Akker^6^, Nanna Maaloe^1^

#### ^1^University of Copenhagen; ^2^Aarhus University Hospital; ^3^The Aga Khan University; ^4^Comprehensive Community Based Rehabilitation Tanzania; ^5^Copenhagen University Hospital Hvidovre; ^6^Athena Institute

*BMC Proceedings 2024*, **18(5):**O182

Submission ID #: IMNHC886

**Background**

Despite worldwide use, reviews of oxytocin for labor augmentation include mainly studies from high-income countries. Besides having potential benefits on labor progress, oxytocin may be harmful to the fetus, with potentially higher risks in low-resource settings. We therefore conducted a systematic review and meta-analysis of practices, benefits, and risks of oxytocin for labor augmentation in low- and lower-middle-income countries (LLMIC).

**Methods**

PubMed, Embase, Psycinfo, Index Medicus, Cochrane, and Google Scholar were searched for publications until January 1, 2022. All studies evaluating oxytocin augmentation rates or the association between oxytocin augmentation and maternal and perinatal outcomes were included. Data were extracted by two researchers using a modified Newcastle-Ottawa Scale. Generic inverse variance outcome and a random-effects model were used. Adjusted or crude effect measures with 95% confidence intervals (CIs) were used.

**Results**

In total, 42 studies were included, presenting data from 885 health facilities in 25 LLMICs (124,643 women). Rates of oxytocin for labor augmentation varied from 0.7% to 97.0%, exceeding 30% in 14 countries (56%). Four studies investigated timing of oxytocin augmentation and found 89.5% (2,745) of labors augmented with oxytocin did not cross the partograph’s action line. Four cohort and seven case-control studies assessed perinatal outcomes. Though limited by confounding by indication, meta-analysis revealed that oxytocin was associated with: stillbirths and day-1 neonatal mortality RR 1.45 (95% CI 1.02-2.06, *N*=84,077, six studies); low Apgar scores RR 1.54 (95% CI 1.21-1.96, *N*=80,157, four studies); neonatal resuscitation RR 2.69 (95% CI 1.87-3.88, *N*=86,750, three studies); and neonatal encephalopathy RR 2.90 (95% CI 1.87-4.49, *N*=1,383, two studies). No studies evaluated rates of cesarean section or uterine rupture.

**Conclusions**

This review discloses a disconcerting level of oxytocin use, which in many cases did not fulfill criteria for dystocia. Oxytocin appears associated with increased perinatal risks, which are likely mediated by lack of appropriate fetal monitoring in the busy low-resource contexts. Robust implementation research in real-world labor wards is warranted to bridge the gap between current universal oxytocin guidelines and clinical realities, particularly where most of the world’s preventable deaths occur.

## O183. Missed nursing care and informal task shifting within kenyan newborn units and the relationship with nurse staffing levels are key quality concerns

### Abdulazeez Imam^1^, David Gathara^2^, Jackson Maina^3^, Martin Aluvaala^4^, Vincent Kagonya^3^, Onesmus Onyango^3^, Mike English1

#### ^1^University of Oxford; ^2^London School of Hygiene and Tropical Medicine; ^3^KEMRI-Wellcome Research Programme; ^4^University of Nairobi

*BMC Proceedings 2024*, **18(5):**O183

Submission ID #: IMNHC885

**Background**

Hospital ward staffing levels in resource-constrained low middle-income countries are extreme. Ethnographic evidence suggests nursing tasks are missed with increased informal task shifting by nurses to caregivers and unsupervised nursing students to cope with care demands. The extent of informal task shifting and how this changes with nurse staffing levels has not been quantified. This study determines the magnitude of missed nursing care and informal nurse task shifting across eight neonatal units in Kenya and examines the relation with nurse staffing.

**Methods**

Using direct bedside observations of care and a structured checklist, we identify whether expected care is provided to a newborn and by whom and which critical components of care are missed completely. We used the nursing care hours per patient shift to determine average nursing time per patient per 12-hour shift as a measure of nursing input. We examine how this measure of nurse input is associated with extent and nature of missed nursing care and informal task delegation using logistic regression analysis.

**Results**

Preliminary observation data on 290 babies across 80 12-hour nursing shifts in four hospitals show 67.6% of expected care is not provided by professional nurses. Unsupervised nursing students conduct between 26% to 100% of vital sign monitoring. Between 88% and a 100% of nasogastric tube feeding was conducted by unsupervised mothers who missed critical steps such as checking if the tube was in-situ pre-feed. On average, over a 12-hour shift period, a nurse could at best devote 24 minutes per baby under their care assuming they allocated time equally to all babies. Further data collection in eight hospitals between October to December 2022 will yield data on care provided to a further 500 babies and be used to present updated analyses including on the link between nurse staffing and the extent of informal task shifting.

**Conclusions**

Nurse staffing levels are low within the study context. Caregivers and unsupervised students provide a significant proportion of care, sometimes missing critical steps. Demonstrating an association between staffing levels and increased informal task shifting will be important in providing evidence for better nurse staffing

## P184. Assessing factors associated with newborn death due to birth asphyxia: a case study of kawempe national referral hospital

### Nathan Lubowa, Patrick Walugembe, Nathan Tumwesigye, Sharon Tsui

#### FHI 360

*BMC Proceedings 2024*, **18(5):**P184

Submission ID #: IMNHC880

**Background**

The perinatal mortality rate (PMR) is estimated at 36 deaths per 1,000 total births in Uganda, exceeding the Ugandan and Every Newborn Action Plan (ENAP) target of 24/1,000 total births. Birth asphyxia (BA) accounts for nearly one-third of perinatal deaths in Uganda and is commonly a consequence of delayed decisions to intervene during the intrapartum period. In the Kampala Metropolitan Area, most neonates with BA are referred from lower-level health facilities and hospitals to Kawempe National Referral Hospital (KNRH) for management. KNRH’s institutional PMR is 110 deaths per 1,000 births. This study aims to identify factors associated with death due to BA at KNRH.

**Methods**

This study is a retrospective cross-sectional analysis of perinatal deaths from July 2021 to June 2022 using the Ministry of Health’s maternal and perinatal death surveillance and response (MPDSR) form, which includes data on maternal and fetal/newborn characteristics, maternal health seeking, and pre- and post-delivery service provision. MPDSR data from DHIS2 were cleaned for outliers and inconsistent entries, and continuous variables were categorized into binary variables. Factors associated with death from BA were assessed for collinearity and analyzed using descriptive statistics and multivariate logistic regression with Stata-14.

**Results**

There were 22,728 deliveries, 2,500 perinatal deaths, 1,498 cases of BA. BA accounted for 37% of 455 newborns 0–7 days death reviewed. Among BA deaths 0–7 days, bivariate analyses indicated a significant relationship between BA-related death and low birthweight, increased parity and number of living children, delayed health seeking, low APGAR, resuscitation, and inappropriate intervention. Adjusting for covariates, multivariate analyses found that low birthweight (OR=4.96, 95% CI 3.15, 7.80), delayed health seeking (OR=1.99, 95% CI 1.09, 3.64), and inappropriate intervention (OR=1.84, 95% CI 1.15, 2.95) remained significantly associated with BA among neonatal deaths within 0–7 days.

**Conclusions**

Neonatal fatality due to BA is associated with low birthweight, delayed health seeking, and BA managed with inappropriate intervention. These findings emphasize the importance of improved quality of care and appropriate clinical management of BA, and efforts to reduce delays to health seeking by educating women on danger signs and supporting birth preparedness.

## O185. Strengthening maternal and perinatal death surveillance and response data use at Kawempe National referral hospital, Kampala, Uganda

### Nathan Lubowa, Patrick Walugembe, Sharon Tsui, Nathan Tumwesigye

#### FHI 360

*BMC Proceedings 2024*, **18(5):**O185

Submission ID #: IMNHC872

**Background**

In 2012 the Uganda Ministry of Health (MOH) adopted maternal-perinatal death surveillance and response (MPDSR), an essential quality improvement intervention to identify, notify, quantify, and determine the causes of maternal deaths, stillbirths, and early neonatal deaths to prevent future mortality. Since 2020, the U.S. Agency for International Development Maternal Child Health and Nutrition (MCHN) Activity has supported functionalization of MPDSR at the national level and in Kampala district, including Kawempe National Referral Hospital (KNRH).

**Methods**

MCHN allocates two data clerks to KNRH to ensure an adequate supply of reporting tools and provide technical support for timely entry of death and perinatal review data in the DHIS2 system. MCHN also provides monthly and weekly data analytics on MPDSR, using data from the monthly HMIS report, weekly mTRAC reports, and real-time MPDSR review data. The analytics are used to support KNRH’s quarterly performance review where stakeholders discuss referrals, cause of death, supply chain issues, case management, and generate action plans to improve maternal and perinatal service delivery at facility and community levels. Between July 2021 to June 2022, MCHN supported four quarterly performance review meetings.

**Results**

The percentage of maternal deaths reviewed improved by 31% (from 69% in July 2021 to 100% in June 2022), and perinatal deaths reviewed improved by 14% (from 41% in July 2021 to 55% in June 2022), both exceeding national targets. Increased death audits at KNRH can be attributed to improved data accessibility and utilization by key stakeholders at both national and facility levels. Data utilization includes active tracking of timely death reviews by MOH representatives who attend the national weekly MPDSR review meeting, as well as health worker reflections on common causes of death, how to address gaps in human resources and supplies, improve timely communication, and manage referrals to prevent future deaths.

**Conclusions**

Increased MPDSR data accessibility has strengthened data use among relevant stakeholders, resulting in improved accountability reviewing maternal and perinatal deaths by the MOH and increased action at the facility level to address barriers to quality service delivery.

## O186. Process evaluation of introducing non-pneumatic anti-shock Garment (NASG) in Northern Province of Zambia

### Oluwaseun Aladesanmi^1^, Aniset Kamanga^1^, Naomi Medina-Jaudes^2^, Morrison Zulu^1^, Caren Chizuni^3^, Hilda Shakwelele^1^, Angel Mwiche^3^, Andy Carmone^1^, Margaret Prust^1^

#### ^1^Clinton Health Access Initiative; ^2^Independent Consultant, ^3^Ministry of Health Zambia

*BMC Proceedings 2024*, **18(5):**O186

Submission ID #: IMNHC867

**Background**

A disproportionate burden of maternal deaths occur in low- and middle-income countries, and obstetric hemorrhage (OH) is a leading cause of mortality. In Zambia, the largest proportion of maternal deaths are directly caused by OH. The NASG is a first aid tool that uses compression to the abdomen and lower body to stop and reverse hypovolemic shock secondary to OH. We describe the process and experiences of introducing the NASG into the Zambia public health system to inform decisions about implementation scale-up.

**Methods**

We conducted an observational study of NASG introduction to 143 Clinton Health Access Initiative program-supported health facilities in Northern Province, organizing observations into the five dimensions of the RE-AIM evaluation framework: reach, effectiveness, adoption, implementation, and maintenance. The NASG study commenced in August 2020 with the introduction evaluated for 18 months. Data on healthcare worker training, mentorship, cases of OH, and NASG availability and use during the study period were collected and analyzed

**Results**

The NASG was successfully introduced and integrated into the health system, and appropriately used by healthcare workers when responding to cases of OH. Sixteen months after introduction, NASGs were available and functional at 99% of study sites and 88% reported ever using a NASG. Of the 68 cases of recorded OH where a NASG was applied, 66 were confirmed as clinically appropriate. Feedback from healthcare workers revealed that 97% thought introducing the NASG was a good decision, and 92% felt confident in their ability to apply the NASG after initial training. The RE-AIM average for this study was 0.65, suggesting an unequivocal public health impact and that NASG introduction had a positive population-based effect

**Conclusions**

A successful NASG demonstration took place over the course of 18 months in the health system of Northern Province, suggesting that incorporation of NASG into the standard of care for obstetric emergency in the Zambia public sector is feasible and can be maintained with minimal external support. The Zambian government is using these results and experiences to guide scale-up of use of the NASG to manage OH across other geographies

## O187. Implementation experiences of the Group Antenatal Care (G-ANC) model for adolescent girls and young women in public health facilities in Kampala, Uganda: a qualitative study

### Doris Kwesiga^1^, Gertrude Namazzi^1^, Darius Kajjo^1^, Peter Waiswa^1^, Joy Angulo^2^, Sharon Tsui^3^

#### ^1^Makerere University School Of Public Health; ^2^EnCompass LLC, ^3^FHI 360

*BMC Proceedings 2024*, **18(5):**O187

Submission ID #: IMNHC864

**Background**

Teenage pregnancies are a public health problem in Uganda, associated with adverse maternal and perinatal outcomes. Pregnant teenagers have special reproductive health needs and social and emotional requirements, yet there are limited adolescent-friendly maternal services. G-ANC is provided to groups of adolescent girls and young women (AGYW) with similar gestation and/or client age and is designed to provide health education, social support, and training on self-care to address the unique needs of AGYW.

**Objective**

The U.S. Agency for International Development Maternal Child Health and Nutrition (MCHN) Activity seeks to strengthen maternal and newborn health service delivery to adolescent mothers by functionalizing G-ANC in seven public facilities in Kampala. The purpose of documenting the implementation experiences of G-ANC is to inform scale-up.

**Methods**

MCHN conducted interviews with 34 key informants involved in G-ANC and group postnatal care (PNC) in June 2022 (24 AGYW, 5 peer mothers, and 5 midwives) at three public health facilities (1 health center III, 1 health center IV, 1 hospital). The interviews probed benefits, challenges, and best practices of G-ANC, and were audio recorded, transcribed and analyzed using content thematic analysis.

**Results**

Participants shared the advantages of G-ANC. These included health education and learning through experience sharing and peer support where members provided and received emotional and in-kind support and learned to handle stigma, thus increased self-esteem and confidence. The challenges of G-ANC were long waiting times due to members who come late and a limited number of midwives to provide physical examination and manage complications; lack of professional counsellors to address more complex issues faced by adolescents; lack of privacy in the group setting to discuss more sensitive/personal challenges; unmet expectations of incentives and skilling in income generating activities; heavy workload for the few peer mothers and midwives; and inadequate space for group sessions.

**Conclusions**

G-ANC is an important mechanism to link pregnant AGYW to maternal services and provide health education and psychosocial support. Gaps in human resource and facility space should be addressed to optimize service provision.

## P188. A structured approach to scale-up of Kangaroo Mother care for low birthweight newborns in Kenya: a critical review of the journey and progress

### Sarah Williams, Teresa Akun, Rita Muchoma, Andrew Clarke, Lynn Kanyuuru, Tewodros Gebremichael

#### Save the Children

*BMC Proceedings 2024*, **18(5):**P188

Submission ID #: IMNHC853

**Background**

Preterm birth is the leading cause of death in children under five globally. Kangaroo mother care (KMC), caring for a premature baby on the chest of their mother or other caregiver, skin-to-skin, and exclusively feeding breastmilk is recommended by the World Health Organization. Compared with conventional care, KMC is associated with a 40% lower risk of mortality at discharge. KMC is poorly adopted in many countries. In 2014 KMC was not an established part of care for preterm newborns in Kenya, we collaborated with Kenya’s Ministry of Health (MOH) on an eight-year programme to pilot, scale, and embed KMC.

**Methods**

Simultaneous interventions were adopted to secure political commitment, a supportive policy environment, a better equipped health workforce, and receptive community expectations towards sick and small newborns. Collaboration with MOH and the Neonatal Technical Working Group supported national guideline development. Policy-makers were exposed to experiences in Tanzania and Malawi. Facility readiness was assessed and five centers of excellence established as resources. National and local-level trainers were trained and supportive supervision and mentoring systems embedded. This was complemented by support to a national champions network, community awareness-raising about KMC, and prematurity and advocacy for KMC data inclusion in national Kenya health information system (KHIS) and supervision tools.

**Results**

In 18 program counties, from a baseline of zero, 85 health facilities now provide dedicated KMC services for low birthweight babies and their mothers. Between 2017 and 2021, the percentage of eligible newborns initiated on KMC rose from 8% to 54%. By 2021, 40 counties out of 47 in Kenya routinely report on KMC indicators within the KHIS.

**Conclusions**

Coordinated, targeted support facilitates piloting and scaling. Globally relevant lessons from a multi-stakeholder reflection exercise will be shared including importance of collaboration across health systems, development and provision of national guidelines, and incorporation of KMC indicators into HIS for visibility and QI. Support from managers and policy-makers enable necessary resources (space, staff, and equipment). Sharing lessons from neighbouring countries is valuable. Training alone is not enough; supervision and mentorship were critical, helping mitigate staff turnover. Maternal/carers ability to provide KMC is affected by family, community support, and cultural sensitisation.

## O189. ReJudge: development and testing of an intervention to reduce Cesarean Section (CS) carried out due to fear of litigation

### Gill Moncrieff^1^, Soo Downe^1^, B.R. Shamanna^2^, Sunny Mannava^2^, Indie Kaur^3^, Joanna Erdman^4^, Maria Regina Torloni^5^, Ana Pilar Betran^6^

#### ^1^University of Central Lancashire; ^2^University of Hyderabad; ^3^Fernandez Foundation; ^4^Dalhousie University; ^5^São Paulo Federal University; ^6^World Health Organization

*BMC Proceedings 2024*, **18(5):**O189

Submission ID #: IMNHC851

**Background**

Obstetrics is a leading area for litigation worldwide, which can result in defensive health care practices including the use of intervention to protect against litigation. CS rates are over 80% in some regions of India, which is likely to result in medically generated harms. Existing interventions that aim to reduce unnecessary CS include educational interventions and clinical guidelines. Fear of litigation is recognized as a factor that stimulates unnecessary CS. We were unaware of an existing intervention specifically targeted to this driver of unnecessary CS.

The ReJudge project was designed to develop an intervention to reduce CS carried out due to fear of litigation.

**Methods**

We explored attitudes and knowledge relating to CS carried out due to fear of litigation over three phases. In phase one, we carried out scoping reviews to derive associated behavioral drivers and mental models. In phase two, we interviewed obstetricians, lawyers, and women’s representatives and mapped interview data to the behavioral drivers and mental models. In phase three, we held a seminar and vignette-based workshop with lawyers and obstetricians. A before and after survey tested knowledge and attitudes and whether the workshop altered these. An educational tool was designed based on the findings.

**Results**

CS due to fear of litigation is driven by complex social, environmental, and cognitive drivers. Social drivers include actual, perceived, and perpetuated social norms, including women’s choice. Environmental drivers include legal and obstetric structures that facilitate CS, including informed consent as a mechanism of distribution of responsibility and the lack of requirement to defend against unnecessary CS. Cognitive drivers include the consequences of adverse outcomes, including legal processes and violence, and resulting potential professional and/or psychological demise.

Our workshop intervention provided a valuable opportunity for interdisciplinary reflection. Striking variations in knowledge and attitudes were evident between professionals that need to be further explored and resolved.

**Conclusions**

The ReJudge intervention provided an opportunity for interdisciplinary knowledge sharing and offers the potential to develop solutions to similar challenges. Further refinement could provide a valuable tool to explore beliefs and unify relevant knowledge between obstetric and legal professionals and beyond.

## O190. Novel, reusable postpartum blood loss monitoring tray: acceptability study and randomized trial

### Mandisa Singata-Madliki, Justus Hofmeyr

#### Universities of Witwatersrand

*BMC Proceedings 2024*, **18(5):**O190

Submission ID #: IMNHC848

**Background**

Monitoring blood loss after birth saves lives by prompting early treatment of postpartum hemorrhage. We developed a reusable blood loss monitoring tray with a wedge, which slips under the mother’s buttocks after birth, and two calibrated blood collection chambers. The first overflows to the second at 500ml to signal postpartum hemorrhage.

**Methods**

We evaluated acceptability and functionality among 41 women giving birth at the Frere Hospital Midwives Obstetric Unit, South Africa, using a structured questionnaire.

We plan to conduct a randomized trial comparing the tray with the disposable Brass V drape.

**Results**

The most positive option was chosen by 95% or more of midwives and mothers for all questions:

Mothers:

- Was it easy or difficult to have the tray in place? 39 (95%) Very easy; 2 (5%) Fairly easy
- Did you feel comfortable with the tray in place? 39 (95%) Very comfortable; 2 (5%) A little comfortable
- Did you feel that the tray helped to keep you clean? 41 (100%) Helped a lot
- Would you choose to use the tray for a future birth? 41 (100%) Yes I would be keen to use it

Midwives:

- Was it easy or difficult to put the tray in place? 40 (98%) Very easy; 1 (2%) Fairly easy
- Did the tray help with placement of the delivered placenta?40 (98%) Helped a lot; 1 (2%) Helped a little
- Did the tray help you to monitor the ongoing volume of blood loss? 41 (100%) Helped a lot
- Did the tray help to keep the mother clean? 41 (100%) Helped a lot
- Would you choose to use the tray for every birth you attend? 41 (100%) Yes I would be keen to use it.

**Conclusions**

The Maternawell tray shows promise as a cost-effective, user-friendly device for monitoring blood loss after birth. To achieve sustainable global access, we have partnered with Maternova, a health technology company (U.S.) and Umoya, a social enterprise (South Africa) to commercialize the tray.

**Disclosure**

GJH declares a financial interest

**Reference**

1. Singata-Madliki M, Hofmeyr GJ. A novel, re-usable ‘Safe birth Tray’ for postpartum blood loss monitoring: A preliminary acceptability assessment. Int J Gynaecol Obstet. 2021 Dec;155(3):553-555

## O191. Health protection schemes and maternal and newborn health: role of the Sehat Sahulat programme in Pakistan

### Sabeen Afzal, Mishal Javed, Muhammad Arshad

#### Ministry of National Health Services Regulations and Coordination, Pakistan

*BMC Proceedings 2024*, **18(5):**O191

Submission ID #: IMNHC846

**Background**

To improve health outcomes and alleviate the financial burden of ill-health, the federal and provincial governments have introduced a key social health protection program, Sehat Sahulat Program (SSP) to cover over 170 million Pakistanis, in addition to the existing relatively very small social health protection schemes under Zakat and Bait-ul-Mal. SSP, previously known as Prime Minister National Health Program, was introduced in 2015 to pave the way for universal health coverage (UHC). It was initially implemented in 40 districts and is currently functional in over 90 districts of Azad Jammu and Kashmi, Gilgit-Baltistan, Islamabad Capital Territory, Punjab, Newly Merged Districts of Khyber Pakhtunkhwa, Khyber Pakhtunkhwa, and Tharparkar district of Sindh, in Pakistan SSP provides financial health protection to identified indigent citizens against catastrophic health expenditure. Initially Program was using data from National Socioeconomic Registry and defined poverty as families with daily income lower than USD 2. Now all individuals of aforesaid covered areas registered with National Database Registration Authority are automatically enrolled in SSP, with the eventual goal of universal coverage across Pakistan

**Methods**

Currently, covers almost all inpatient health services and does not include any form of co-payments. The reproductive, maternal, newborn, and child health (RMNCH) package includes normal delivery, cesarean section, three antenatal visits, one postnatal visit, counselling for immunization, family planning and nutrition, and one long-term family planning intervention. The objective of the study was to assess the role of Sehat Sahulat program in improving RMNCH outcomes by comparing the UHC service coverage score (UHC SCI) and RMNCH score of 2015 to 2021 in the initial SSP 40 districts and percentage change in UHC SCI in the initial SSP 40 districts. Both primary and secondary data was used to calculate the score.

**Results**

Since the implementation of SSP, the expenditure incurred on maternal health services is approximately PKR 10.25 billion. In the districts targeted initially, except for Kohat & Khuzdar, UHC SCI and RMNCH scores have increased significantly from 2015 to 2021. The greatest increase in UHC SCI and RMNCH scores, was observed in Khyber Pakhtunkhwa; increasing from 39.0% to 49.5%. In other areas, increase in UHC SCI ranged from 26% in Gilgit-Baltistan to 29.9% in Balochistan. The percentage change ranges from 26 to 37.7 being greatest in Khyber Pakhtunkhwa

**Conclusions**

The increase in UHC SCI and RMNCH scores suggests improved coverage of essential health services, particularly maternal health services through SSP, leading to improved health outcomes. However, there is a need to consider expanding the benefit package in terms of both services and population coverage with a shift from health protection to health insurance scheme. The limitations study is that it only looks at change in UHC SCI and did not look at the utilization of services and Financial protection of the target population. Therefore, there is need to explore a detailed member cohort analysis by membership duration as the Program expands (e.g. newer versus older members) with a multivariate statistical analysis.

## O192. Evaluating service readiness for small and sick newborns to inform tracking of the Every Newborn Action Plan (ENAP) coverage target: baseline results of a health facility assessment from 65 facilities in four African countries

### Rebecca Penzias^1^, Christine Bohne^2^, Samuel Ngwala^2^, Evelyn Zimba^2^, Ekran Rashid^2^, Edith Gicheha^2^, Opeyemi Odedere^2^, Olabisi Dosunmu^3^, Robert Tillya^4^, Josephine Shabani^4^, David Gathara^1^, James Cross1, Eric Ohuma^1^, Joy Lawn^1^

#### ^1^London School of Hygiene & Tropical Medicine; ^2^NEST360; ^3^APIN Public Health; ^4^Ifakara Health Institute

*BMC Proceedings 2024*, **18(5):**O192

Submission ID #: IMNHC827

**Background**

Each year, an estimated 30 million newborns are vulnerable users of a health system. Tools for evaluating service readiness are important for assessing the capacity of hospitals to provide quality care. Unfortunately, existing health facility assessment tools do not include key items and interventions specific to small and sick newborn care (SSNC).

**Methods**

A health facility assessment tool was co-designed with four African governments and multiple experts. Data were collected in 69 neonatal units at 65 Newborn Essential Solutions and Technologies (NEST360) implementing hospitals in Malawi, Kenya, Tanzania, and Nigeria between September 2019 and March 2021. We applied two summary scoring approaches: a) standards based, including all items required for service readiness for SSNC, and b) level-2 with respiratory support, including all items required for World Health Organization level-2 clinical interventions. For each of the scoring approaches, items were scored according to availability and functionality—allocating 0 points if not available, 1 point if available, and 2 points if available and functional. Health facility assessment summary scores from each approach were aggregated and summarised using heatmaps for each hospital, by health system building blocks or level-2 clinical intervention, and for each country.

**Results**

Of 5,051 items, 1,795 (36%) were included in standards-based scoring. Hospitals had a median of 51% (interquartile range [IQR] 48–57%) of items, with some variation by country: 49% (IQR 46–51%) in Malawi, 62% (IQR 59–66%) in Kenya, 55% (IQR 53–62%) in Tanzania, and 50% (IQR 42–58%) in Nigeria. Medical device (43%, IQR 38–48%) and family-centered care (27%, IQR 18–40%) scores were low across most hospitals at baseline. Hospitals in Kenya had higher scores for information systems (74%, IQR 68–80%), than hospitals in other countries (54%, IQR 48–66%). Level-2 scores revealed gaps in readiness to provide level-2 clinical interventions.

**Conclusions**

Two scoring approaches reveal key gaps in readiness to provide SSNC. Medical device and family-centered care scores were low across most hospitals. Addressing these health systems gaps could lead to improvements in SSNC. Future work could link service readiness data for SSNC to newborn mortality and other outcomes data.

## O193. Improving the management of obstetric emergencies in the fragile context of Gao, Mali

### Lazare Coulibaly^1^, Aminata Traore^2^, Demba Traore^1^

#### ^1^John Snow, Inc; ^2^JSI Research & Training Institute, Inc.

*BMC Proceedings 2024*, **18(5):**O193

Submission ID #: IMNHC826

**Background**

In Mali, despite decades of health system strengthening efforts, maternal, neonatal, and infant mortality remain alarming at 325/100,000 live births, 33/1,000 live births, 101/1,000 live births respectively. In Gao, MOMENTUM Integrated Health Resilience, funded by the U.S. Agency for International Development, improves the provision of emergency obstetric and neonatal care (EmONC) by focusing on strengthening the capacity of maternity health care providers.

**Methods**

A needs assessment first identified providers with a need for training in EmONC. Then over 10 days, providers were trained, including simulation with anatomical models and mannequins and clinical internships in the field. Following the training, a program of practical internships has been established in collaboration with the supervisors. Ongoing monitoring and evaluation are done through virtual group meetings to share lessons learned. Challenges and local initiatives are discussed, and obstetric emergency cases are debriefed with the supervisors. Data are recorded in the EmONC and childbirth registers and then entered in DHIS2.

**Results**

Eighty-three percent (10/12) of primary health facilities were able to be fully or partially endowed with EmONC materials/equipment with their own funds thanks to MOMENTUM’S advocacy. Obstetric emergencies treated increased from 25 (January-April 2022) to 143 (20 May-15 August 2022). Reference/evacuation from primary health facilities to district hospitals decreased from 29 to 115 for the same periods. The average acceptable level of knowledge increased from 29% in the pre-test to 88% in the post-test. However, for individual skills on task trainers (suction cup and newborn resuscitation), the acceptable average level of performance established by national guidelines is 80%. At present, the group trained is still performing at less than 70%.

**Conclusions**

In Gao, human health resources are scarce, migratory, and often require more support to offer quality care. Ongoing capacity building of providers and care teams is essential and feasible to reduce maternal and neonatal mortality and strengthen the resilience of the broader health system. While knowledge increased during training, ongoing support/coaching is needed to translate knowledge into practical application.

## O194. Theory of change to accelerate scale up of high-quality small and sick newborn care in Kenya, Malawi, Nigeria, and Tanzania with NEST360

### Christine Bohne^1^, Evelyn Zimba^1^, Norman Lufesi^2^, Aba Asibon^1^, Nahya Salim^3^, Honorati Masanja^4^, Chinyere Ezeaka^5^, Grace Irimu-Thinwa^6^, David Gathara^7^, Rebecca Richards-Kortum^1^, Maria Oden^1^, Lawrence Cooley^8^, Queen Dube^9^, Joy Lawn^7^

#### ^1^NEST360; ^2^Ministry of Health, Zambia; ^3^Muhimbili University Of Health And Allied Sciences; ^4^Ifakara Health Institute; ^5^University of Lagos; ^6^University of Nairobi; ^7^London School of Hygiene and Tropical Medicine; ^8^Management Systems International; ^9^Ministry of Health, Malawi

*BMC Proceedings 2024*, **18(5):**O194

Submission ID #: IMNHC815

**Background**

The Sustainable Development Goal (SDG) era is halfway through, yet national targets to reduce neonatal mortality rates to < 12 per 1000 live births (SDG3.2) are off track, especially in high-burden countries. To accelerate progress, the Every Newborn Action Plan (ENAP) calls for 80% of districts in each country to have at least one facility offering level-2 small and sick newborn care (SSNC), including respiratory support, by 2025. Despite policy momentum, impact can only be achieved through an integrated health systems package and high-quality coverage. We co-developed a theory of change (ToC) to operationalize this complex systems change for facility- and national-level scale-up to accelerate progress for ENAP and SDG targets.

**Methods**

A formal ToC workshop, using Medical Research Council methodology, was held in Malawi with diverse participants. The process involved focus groups and interactive voting on inputs and critical components. The ToC was further refined by implementers linked to the NEST360-UNICEF Newborn Implementation Toolkit, including working groups organized by WHO health system building block, and implementation learnings from scaling SSNC to 65 facilities across Kenya, Malawi, Nigeria, and Tanzania.

**Results**

ToC workshop in June 2019 was attended by 49 multi-country and multidisciplinary stakeholders including national and district policy-makers, doctors, nurses, engineers, researchers, and program implementors. The majority were from Malawi, but Kenyan, Nigerian, and Tanzanian stakeholders were included. A graphic for facility- and national-level scale of inpatient level-2 SSNC was developed showing impact on neonatal survival, outcome of universal high-quality SSNC, and inputs organized by health systems building blocks, also depicting underlying rationale (addressing technology, market, education, and implementation gaps), assumptions, activities, and indicators. This ToC was further refined through input by >300 implementers and experts from >100 countries linked to the toolkit.

**Conclusions**

This ToC is a practical framework for scaling SSNC and has been used in 65 facilities to systematically improve care, as well as inform integration into national plans, investment cases, design of data tools, quality improvement processes, and other global public goods. It continues to guide the complex evaluation of NEST360, enabling synthesis of program learnings.

## O195. Fast progressors in low birthweight national routine data systems in low- and middle-income countries

### Judith Yargawa^1^, Yemi Okwaraji^1^, Lorena Suarez-Idueta^2^, Eric Ohuma^1^, Ellen Bradley^1^, Hannah Blencowe^1^, Joy Lawn^1^, Vulnerable Newborn Measurement Collaborative Group^3^

#### ^1^London School of Hygiene & Tropical Medicine; ^2^Mexican Society of Public Health; ^3^Multiple Organizations

*BMC Proceedings 2024*, **18(5):**O195

Submission ID #: IMNHC809

**Background**

Measurement of low birthweight (LBW) is key in tracking Sustainable Development Goals’ mortality and health targets, but impeded by major gaps in data coverage and quality, especially in the highest burden regions. Some low- and middle-income countries (LMICs), however, have achieved remarkable progress in recent decades to capture LBW in national data systems. This study aimed to identify LMICs with improvements in capturing LBW data in national routine data systems over two time periods (2000–2004 and 2015–2019).

**Methods**

As part of the Vulnerable Newborn study, we analysed live birth data with LBW information collated in a UN routine administrative database for 2000–2019 using descriptive analyses. We considered all LMIC countries outside Europe meeting these criteria: 80% facility birth coverage, >10,000 livebirths per year, 80% World Population Prospects coverage in any year within 2015–2019, and having 4 years of LBW data within 2015–2019. We assessed relative change in LBW data by comparing mean coverage across earlier (2000–2004) and latter years (2015–2019).

**Results**

Thirty-two LMICs met the inclusion criteria. Of these, 10 countries had no reported LBW data between 2000–2004 but had achieved the 80% coverage threshold by 2015–2019: four in sub-Saharan Africa (Burundi, Malawi, Namibia, Zimbabwe), three in Latin America/Caribbeans (El Salvador, Guyana, Nicaragua), and three in Asia (Iraq, Lebanon, Turkey). For countries reporting some coverage in earlier years, the highest rates of relative change were achieved in sub-Saharan Africa and Latin America/Caribbeans, with the top five countries being Burkina Faso (170.4%), Mozambique (127.3%), Peru (43.3%), Benin (26.1%), and Paraguay (24.7%). Included countries from Asia had the highest median coverage in earlier years among all LMICs (93.5% compared to 53.6% in sub-Saharan Africa and 86.8% in Latin America/Caribbeans).

**Conclusions**

LBW data coverage has increased over time in some LMICs, however missed opportunities remain. Further studies are needed to understand drivers of change within these fast progressor countries to glean lessons to accelerate change in other LMIC settings. Increased LBW data coverage over time in LMICs shows promise for capture of other neonatal outcomes such as stillbirths and preterm births.

## O196. Effect of a community-based kangaroo mother care package on neonatal mortality among preterm and low birthweight infants in Rural Pakistan: a cluster-randomized controlled trial

### Shabina Ariff, Saleema Khowaja, Atif Habib, Zahid Ali Memon, Tariq Samejo, Ikramullah Maznani, Amjad Hussain, Muhammad Jawwad, Arjumand Rizvi, Sajid Soofi, Zulfiqar Bhutta

#### The Aga Khan University

*BMC Proceedings 2024*, **18(5):**O196

Submission ID #: IMNHC807

**Background**

Kangaroo mother care (KMC) is a low-cost intervention that can reduce neonatal mortality. However, its effectiveness in community settings in Pakistan is still unknown. We aimed to assess effect of community KMC (cKMC) on neonatal mortality. The secondary outcomes included frequency of exclusive breastfeeding, weight gain, and incidence of possible serious bacterial infection (PSBI).

**Methods**

A cluster-randomized control trial was conducted in rural Pakistan. Twenty-four clusters were randomized to intervention and control arms. Pregnant women were identified through a pregnancy surveillance system instituted in study areas. Stable neonates weighing ≥ 1200 to < 2500g were recruited within 72 hours of birth. Neonates in intervention clusters received cKMC whereas those in control received essential newborn care. The intervention package comprised KMC calendar in local language and KMC kit with diapers, caps, socks, towels, soap, and sanitary pads. Community mobilization included one-to-one and group sessions with flip-cards, wall mounts, and a self-explanatory video to maximize use of KMC in intervention clusters. Peer-to-peer learning through KMC champions was also practiced in the intervention clusters. An independent team collected data on study outcomes.

**Results**

Between April 2019 and October 2021, 4,977 neonates were enrolled, 2,468 from intervention and 2,510 from control clusters. Majority of parents (99.8%) in intervention clusters reported performing KMC for 26.9 ±3.8 days with a mean duration of 7.8 ±3.7 hours/day. Risk of neonatal mortality was significantly lower in intervention clusters (hazard ratio:0.66, 95% CI:0.44–0.99, *p*=0.045). Proportion of exclusive breastfeeding at 6 months was 36.5% in intervention and 6.8% in control clusters, p< 0.001. Between enrolment and 59 days, 15.1% and 39.9% infants in the intervention and control arm, respectively had symptoms of PSBI, *p*=0.002. Risk of stunting at 6 months (risk ratio [RR]:0.76, 95% CI:0.62–0.93 *p*=0.007) was lower among infants in the intervention arm. However, risk of wasting was similar in both groups (RR:0.73, 95% CI:0.51–1.06 *p*=0.095).

**Conclusions**

Our study findings suggests that KMC is feasible in community context and can reduce neonatal mortality, increase exclusive breastfeeding rates, and decrease risk of PSBI and stunting. We recommend that cKMC be scaled up to improve neonatal outcomes in Pakistan and other

## O197. Does the continuous presence of mothers in a newborn intensive care unit to provide immediate kangaroo mother care increase neonatal sepsis?

### Sugandha Arya^1^, Harish Chellani^2^, Rajiv Bahl^3^, Nitya Wadhwa^4^, Pratima Anand^1^, Ebunoluwa Adejuyigbe^5^, Helga Naburi^6^, Queen Dube^7^, Kondwani Kawaza^8^, Samuel Newton^9^, Gyikua Phlange-Rhule^9^, Siren Rettedal^10^, Nils Bergman^11^, Suman Rao^3^

#### ^1^Safdarjung Hospital; ^2^The Centre for Health Research and Development: Society for Applied Studies; ^3^World Health Organization; ^4^Translational Health Science and Technology Institute; ^5^Obafemi Awolowo University Teaching Hospital Complex; ^6^Muhimbili University of Health and Allied Sciences; ^7^Ministry of Health, Malawi; ^8^Kamuzu University of Health Sciences; ^9^Kwame Nkrumah University Of Science And Technology; ^10^Stavanger University Hospital; ^11^Karolinska Institutet

*BMC Proceedings 2024*, **18(5):**O197

Submission ID #: IMNHC800

**Background**

Multi-country randomized controlled trial conducted in five countries (India, Ghana, Tanzania, Nigeria, Malawi,) coordinated by the World Health Organization (iKMC study) from Nov 2017–Jan 2020 showed 25% reduction in mortality by immediate kangaroo mother care (KMC) in neonates with birthweight 1 to< 1.8 kg. This study was published in *New England Journal of Medicine* (May 2021). To implement immediate KMC (iKMC) intervention, mother and baby needed to be together continuously, which led to the concept of mother-newborn care unit (MNCU). Health care providers and administrators were concerned about the potential for increased infections by the presence of mothers/surrogates in MNCU. We present post-hoc analysis of iKMC study to see effect of iKMC on neonatal sepsis.

**Methods**

The intervention was KMC initiated immediately after birth and continued until discharge compared to conventional care with KMC initiated after meeting stability criteria. The outcomes were rates of neonatal sepsis, case fatality of neonatal sepsis, and profile of bacterial isolates.

**Results**

1,602 newborns were randmoized to control and 1,609 to intervention group. Mean gestational age, mean birthweight, maternal and neonatal care related variables, which can be risk factors for neonatal sepsis, were similar in two groups.

Suspected neonatal sepsis was 18% lower in the intervention group (22.9%) than the control group (27.8%) risk ratio (RR) 0.82 (CI 0.73–0.93) and sepsis-related mortality was 36% less in the intervention group (19.4%) than the control group (25.1%), RR 0.64 (CI 0.48– 0.85); both were statistically significant. The intervention group had fewer Gram-negative isolates (9) than gram-positive isolates (16). While control group had more gram-negative isolates (18) than gram-positive (12). Five multi-drug resistant gram-negative isolates were cultured out of which one was in intervention group and four were in control group.

**Conclusions**

Continuous presence of mothers/surrogates in neonatal intensive care units to provide iKMC does not increase neonatal sepsis but rather reduces it. The profile of bacterial isolates was different in iKMC and control groups; sepsis-related mortality was lower in the iKMC group.

## O198. Measuring care for small and sick newborns: co-design of a minimal neonatal inpatient dataset and multi-country learning to inform tracking of every newborn targets

### James Cross^1^, Christine Bohne^2^, Samuel Ngwala^2^, Evelyn Zimba^2^, John Wainaina^3^, Olabisi Dosunmu^4^, Chinyere Ezeaka5, Josephine Shabani^6^, Robert Tillya^6^, Irabi Kassim^6^, Rebecca Penzias^1^, David Gathara^1^, Eric Ohuma^1^, Joy Lawn^1^

#### ^1^London School of Hygiene & Tropical Medicine; ^2^NEST360; ^3^KEMRI-Wellcome Research Programme; ^4^APIN Public Health; ^5^University of Lagos; ^6^Ifakara Health Institute

*BMC Proceedings 2024*, **18(5):**O198

Submission ID #: IMNHC794

**Background**

Every Newborn Action Plan (ENAP) coverage targets require national scale-up of level 2+ small and sick newborn care (SSNC) in over 90 countries. Routine neonatal inpatient data is fundamental for measuring quality of care, identifying equity gaps, and enabling data-based action at individual and national level. Current neonatal inpatient data tool options vary in purpose, size, complexity, and collection processes. We describe the co-design and operationalization of a core inpatient dataset to drive quality of care and track outcomes for small and sick newborns in low- and middle-income settings.

**Methods**

A three-step systematic, evidence-based, and pragmatic framework was used to co-design and operationalize this novel neonatal inpatient dataset in four countries (Kenya, Malawi, Tanzania, Nigeria) participating in the Newborn Essential Solutions and Technologies (NEST360) Alliance. Existing global and national data tools were identified and systematically mapped according to variable categories. A priori considerations for variable inclusion were determined by clinicians and policy-makers from the four African governments. These included: a parsimonious variable list, electronic data entry, and prioritizing data relating to clinical care pathways and newborn outcomes. The tool was further refined by > 40 global experts during a multi-stakeholder workshop and later online interactions.

**Results**

Among existing national and international tools, size ranged to > 400 variables, often relating to study-specific initiatives or maternal history. The co-designed neonatal inpatient dataset (NID) includes 63 core variables organized in six modules: (1) birth details/maternal history; (2) admission details/identifiers; (3) complications and observations; (4) interventions; (5) discharge outcomes; and (6) diagnosis or cause of death. The NID tool has been implemented at 69 neonatal units in 65 NEST360 implementing hospitals in four African countries and is being connected to national routine data systems, respecting record confidentiality. Data links into a real-time facility dashboard, tracking care pathways and informing quality improvement.

**Conclusions**

This NID is a novel, parsimonious tool, feasible for routine systems, to inform quality of care for inpatient SSNC. This tool is an open-source, adaptable, global public good that aims to enable facility and country-level comparisons and accelerate progress

## P199. Why do women still deliver at home when a national health systems strengthening initiative promotes free facility births? A mixed-methods assessment of drivers and barriers from Rural Guinea-Bissau

### Sabine Margarete Damerow^1^, Helquizine Da Gioa Mendes Lopes^2^, Giuliano Russo^3^, Morten Skovdal^4^, Jane Brandt Sørensen^4^, Ane Baerent Fisker^1^

#### ^1^University of Southern Denmark; ^2^INDEPTH Network; ^3^Wolfson Institute of Population Health, Queen Mary University of London; ^4^University of Copenhagen

*BMC Proceedings 2024*, **18(5):**P199

Submission ID #: IMNHC789

**Background**

Global health practice is increasingly focusing on encouraging facility births to improve maternal-perinatal survival through health systems strengthening (HSS). Yet, in resource-constrained settings, facility birth coverage often remains suboptimal despite extensive HSS initiatives. We explored the factors behind women’s utilization of facility and home births during the implementation of a national HSS initiative in rural Guinea-Bissau.

**Methods**

Using an explanatory sequential mixed-methods approach nested in the Bandim Health Project’s rural Health and Demographic Surveillance System (HDSS), we conducted 258 structured interviews with women who had recently given birth in 19 randomly selected HDSS villages. These were complemented by 12 in-depth interviews to explore emerging patterns. Data was collected in 2021. We analysed data using descriptive statistics and thematic network analysis guided by theories of social practice.

**Results**

In the structured interviews, most women (146/258, 57%) mentioned facilitators to facility births, mostly concerning safety benefits at health facilities. Nevertheless, only half of the women had given birth at a health facility (128/258), and 48% at home (125/258). Twenty-eight percent of the women mentioned barriers to facility births (73/258), mostly concerning geographical and financial constraints. When further probing into barriers during in-depth interviews, women added material prerequisites that need to be in place to access facility births but did not necessarily denote these as barriers. Despite facility births being officially free of charge, such prerequisites predominantly concerned direct and indirect payments. Accordingly, out-of-pocket payments were reported by 71% of women who had given birth at a health facility in the structured interviews (91/128), but only three of the 91 women who paid called such payment a barrier.

**Conclusions**

Ubiquitous direct and indirect payments suggest a phenomenon of commodification of facility births in rural Guinea-Bissau, such that individual purchasing power remains key to utilization. As the vast majority of our interviewees did not recognize such payments as barriers to care, we hypothesize that women do not feel entitled to free-of-charge facility births. Our findings raise important equity concerns, and call for integrating assessments of persistent barriers to care into routine monitoring of HSS initiatives.

## O200. What do health workers know and do about developmentally supportive care for preterm infants in a low-income setting: evidence from Uganda

### Zelee Hill^1^, Victoria Nakibuuka^2^, Fiona Denison^3^

#### ^1^Institute for Global Health, University College London; ^2^Nsambya Hospital, Kampala; ^3^University of Edinburgh

*BMC Proceedings 2024*, **18(5):**O200

Submission ID #: IMNHC784

**Background**

Premature birth disrupts in-utero brain development, and preterm infants are at risk of long-term neurodevelopmental issues. The neonatal unit (NICU) can be stressful for a preterm infant at a time of rapid brain growth and plasticity, this may contribute to poorer developmental outcomes. Modifying the environment to protect against negative sensory experiences and ensure positive stimuli from caregivers is the standard of care in many high-income settings. Little is known about developmentally supportive care in low or middle-income settings, yet this is where 81% of pre-terms are born. To add to this sparse knowledge base we determined current knowledge and practice relating to developmentally supportive care among health workers in Uganda.

**Methods**

We surveyed 135 health workers in four NICUs to determine knowledge and practice of developmentally supportive care. We complimented this with direct observations and sound measurements.

**Results**

Knowledge of developmentally supportive care was low; only 36% of respondents reported that stress, and 21% that parental interaction, can affect brain development. Loud noises, hunger, and painful procedures were most frequently perceived to cause stress. Kangaroo care (KMC), parental contact, sleep, nesting, positioning, handling, and lighting were less frequently mentioned. Eighty-four percent of respondents reported actions were taken to protect infants from excessive light (window shades/curtains and phototherapy masks), 33% from excessive sound (turning off/limiting alarms and radios), and 69% to protect sleep (nesting). There was a wide variation in knowledge and practice across the four NICUs, with the large volume NICUs scoring poorly.

Mothers engaged in practical tasks such as feeding and changing nappies, with the main benefit perceived as reducing parental stress (67%); infection risk was perceived as a negative of their involvement (71%). KMC knowledge was high and 64% had KMC training; 88% reported KMC in the unit in the last seven days, although KMC was rarely seen during observations. The large volume NICU had the highest sound readings (89–99 dB), and all had readings over the recommended < 65dB.

**Conclusions**

More needs to be done to improve knowledge and practice of developmentally supportive care, which can build on the good practices in some NICUs.

## O201. Community Experiences and Beliefs Related to Exclusive Breastfeeding (EBF) and early supplementation in urban slums of Karachi, Pakistan: a qualitative study

### Kheezran Ahmed^1^, Sana Qaiser^1^, Maryam Mansoor^1^, Benazir Baloch^1^, Ameer Muhammad^2^, Sajid Iqbal^1^, Yasmin Parpio^1^, Yasir Shafiq^1^, Muhammad Imran Nisar^1^, Valerie Flaherman^3^, Fyezah Jehan^1^

#### ^1^The Aga Khan University; ^2^Vital Pakistan; ^3^University of California, San Francisco

*BMC Proceedings 2024*, **18(5):**O201

Submission ID #: IMNHC771

**Background**

Exclusive breastfeeding (EBF) until six months of age is recommended as it contributes to childhood growth and development and protects against infections. Establishing EBF in certain settings can be difficult especially when women do not have access to basic antenatal or postnatal care, which is a critical window for education about breastfeeding. Myths and beliefs make way instead, and socio-economic practices influence and vary across cultures. In settings of infant growth failure or maternal undernutrition, early supplementation is preferred. While it is important to understand local experiences regarding EBF it is also critical to understand the community practices for early supplementation to improve EBF.

**Methods**

A qualitative study was conducted in four urban slums of Karachi, Pakistan. Focus group discussions were conducted with mothers, fathers, and grandmothers of infants as well as community health workers. Minor mothers and those who refused consent were excluded.

**Results**

Eighty-four participants (68 mothers, 8 fathers, and 8 mothers-in-law) were recruited and 9 focus group discussions were conducted. Although overall knowledge about the benefits of breastfeeding was sufficient, many mothers were not aware of proper breastfeeding techniques. Mothers’ health and inadequate milk production were considered key drivers or barriers influencing EBF. A lack of psychosocial support and extended family members being the key decision-makers also interfered with mothers’ ability to EBF. Only a few mothers did not practice early supplementation or pre-lacteal feeding. In situations where breastfeeding was not possible, early complementary feeding was a preferred option as compared with breast milk expression or formula feeding.

**Conclusions**

Caregivers know the recommended feeding practices, yet adherence remains a challenge. A nuanced approach is required to explore these barriers so policy-makers and awareness campaigns can target specific reasons hindering EBF. There is a need to strengthen evidence related to whether to provide early supplementation in conditions where challenges arise owing to the availability or ability to breastfeed. Lastly, maternal counselling and guidance regarding breastfeeding techniques should be inculcated at regular antenatal and postnatal checkups.

## P202. Economic burden for maternity care during COVID-19 pandemic in Nepal

### Ashish KC^1^, Omkar Basnet^2^, Pratiksha Bhattarai^2^, Avinash K Sunny^2^, Honey Malla^2^, Rejina Gurung^2^, Prajwal Paudel^3^, Mats Malqvist^1^

#### ^1^Uppsala Unicversity; ^2^Golden Community; ^3^Paropakar Maternity and Women’s Hospital

*BMC Proceedings 2024*, **18(5):**P202

Submission ID #: IMNHC762

**Background**

The COVID-19 pandemic has caused disruptions in global health and economic stability. In Nepal, before the pandemic, more than 50% of health care costs were out-of-pocket expenditure (OOPE). This study aimed to assess the OOPE for maternity care during before and during the COVID-19 pandemic in Nepal.

**Methods**

We conducted a cohort study from 1 March 2019 to 31 Dec 2020 at nine hospitals in Nepal. We compared a 12-month pre-pandemic period with a 9-months of pandemic period. OOPE for hospital charges and other expenses was collected through a semi-structured interview with 53,864 mothers during discharge. We analyzed OOPE for overall mode of delivery and wealth quintile. Inferential statistics was used to compare findings between the two time periods.

**Results**

The OOPE for childbirth increased by 19% during pandemic, with a rise in cost of care from 21.5 USD to 25.6 USD (*p*< 0.001). The cost of laboratory diagnosis increased by 14% for women who had spontaneous birth during the pandemic. Costs increased by 22.4% from 12.7 to 17.4 USD (*p*< 0.001) for spontaneous vaginal birth; for instrumental births, costs increased by 39.9% with a rise from 20.8 to 29.1 USD (*p*< 0.001), and for cesarean sections, costs increased by 5.7% with a rise from 34.9.5 to 36.9 USD (*p*< 0.001). The OOPE among the poorest wealth group increased by 14%, whereas the costs for the wealthier quintiles increased by up to 61% during the pandemic period.

**Conclusions**

Out-of-pocket expenditure for maternity care increased during the COVID-19 pandemic in Nepal. In addition to increasing costs for all modes of delivery during the pandemic, the rate of cesarean sections increased, and thus the overall cost of care. Mitigation efforts to secure affordable maternal health care services are required.

## O203. Setting up a cadre of local trainers as sustainable skilled human resources for maternal and newborn health care: lessons learned from Rural Maharashtra, India

### Shilpa Karvande^1^, Vidula Purohit^1^, Matthews Mathai^2^, Subhasri Balakrishnan^3^, Prashant Kulkarni^4^, Helen Allott^5^, Reeta Jha^6^, Elisabeth Serle^3^, Shubhro Mullick^3^, Rajendra Kale^1^, Nerges Mistry^1^

#### ^1^Foundation for Medical Research, India; ^2^Unknown; ^3^Independent Consultant; ^4^Johns Hopkins India Private Limited; ^5^Liverpool School of Tropical Medicine; ^6^Royal Free London Hospitals Foundation Trust

*BMC Proceedings 2024*, **18(5):**O203

Submission ID #: IMNHC752

**Background**

Pre-service training of public health providers is inadequate to provide quality-based primary health care. Hence, locally available skilled resources for undertaking need-specific in-service training is vital for their continuous professional development. Intervention research on capacity building in maternal and newborn healthcare (MNH) for public health providers in two districts of Maharashtra, India, was undertaken during 2017–22. It aimed at preparing a sustainable group of local trainers with essential clinical and teaching competencies to deliver skills-based training to adult learners.

**Methods**

District A (pilot phase) trainers (*n*=8) were supported by UK-based technical partners to train District B trainers (*n*=32) in clinical and non-clinical skills and training pedagogy with complementary mix of directed and self-directed learning strategies. The six-step process included 1) pre-selection structured assessment of potential candidates; 2) training with pre- and post-training assessment; 3) staggered induction of selected candidates as trainers, start with the “best,” pair them with the “moderate”; 4) regular quality assessment by the UK experts; 5) continuous hand-holding to selected District B trainers by District A trainers; and 6) virtual refresher training. Qualitative interviews of stakeholders were assessed thematically for the process and effect of training.

**Results**

District A trainers gained competency and confidence as core trainers. The six-step process with in-built multiple iterations of skills-based learning helped to achieve optimum clinical competency and teaching qualities. The three-fold effects of training included 1) improved clinical competencies for routine and high-risk/complicated case management, 2) confidence as trainer resulting from increased competency and experience of training, 3) getting recognition as a trained expert by their supervisors and colleagues. The District B trainers with the support from District A significantly improved clinical knowledge and skills (66.1%–79.3%; 33.5%–78.1%) of 463 health workers.

**Conclusions**

A continuous process of developing and strengthening trainers helped to generate a pool of skilled health resources. Creation of learning and teaching opportunities can further maintain momentum of their competency for quality MNH. Application of the training pedagogy demonstrated by this project can be extended to comprehensive primary health care training.

## O204. The parent voices initiative: the first global registry of stillbirth parent support

### Vicki Ponce Hardy^1^, Rakhi Dandona^2^, Hannah Blencowe^3^, Claire Storey^1^, Paula Quigley^4^, Sofia Saterskog^1^, Mary Kinney^5^, Susannah Leisher^1^

#### ^1^International Stillbirth Alliance; ^2^Public Health Foundation of India; ^3^London School of Hygiene & Tropical Medicine; ^4^DAI; ^5^University of the Western Cape

*BMC Proceedings 2024*, **18(5):**O204

Submission ID #: IMNHC750

**Background**

To date, there has been no comprehensive cataloguing of organizations that support parents after a stillbirth, nor any review of the challenges that those organizations face. The International Stillbirth Alliance aimed to (1) identify organizations and, in countries with few or no such organizations, individuals, that support parents following a stillbirth—support providers, and (2) collate this information into a public global registry. We also investigated key challenges faced by support providers.

**Methods**

We conducted a systematic online search between July and September 2020 via Google, Facebook, Meetup, and Reddit, supplemented by a snowball search, to identify support providers. We collected data on support providers’ locations, objectives, types of support offered, and populations reached. Permission was requested to include support providers’ details in the global registry. The search was supplemented by a survey (93 respondents) and semi-structured interviews (15 interviewees) about support providers’ successes and challenges in relation to six areas: stigma, funding, reach, policy, workforce, and advocacy. Qualitative data were analysed thematically.

**Results**

We identified 499 organizations and 120 individuals—point persons—from 75 countries that provide support to parents after a stillbirth. Overall, 60% of organizations identified were in Northern America and Europe, with no providers in Oceania (excluding Australia and New Zealand). In the six countries with the largest numbers of stillbirths—India, Nigeria, China, Ethiopia, Pakistan, and the the Democratic Republic of the Congo—we found just eight support organizations. As of September 2022, the global registry of stillbirth parent support organizations includes 147 support providers. The main challenges identified by support providers related to accessing funding, reaching key populations, training staff, navigating policies around bereavement care, and overcoming silence and stigma around stillbirth.

**Conclusions**

This is the first investigation of available global stillbirth parent support providers and the challenges they face. There remain large gaps in support, especially in regions with the highest numbers of stillbirths. Challenges faced by support providers are complex and often structural. Overcoming these challenges will require collaboration, effort, and political will at all levels. The International Stillbirth Alliance plans to extend the global registry to include bereavement support care for parents with newborn deaths.

## O205. Coaching and mentoring as a supplemental approach to building capacity of health workers in primary care facilities in Lumbini and Karnali Province of Nepal

### Deepak Paudel^1^, Robert McPherson^2^, Adweeti Nepal^3^, Hom Nath Subedi^4^, Samikshya Singh^4^

#### ^1^Save the Children; ^2^Indpendent Consultant; ^3^CARE; ^4^Abt Associates

*BMC Proceedings 2024*, **18(5):**O205

Submission ID #: IMNHC748

**Background**

Knowledge and skills of health service providers must be routinely refreshed and practiced. One alternative to relying solely on classroom or clinic-based training is to strengthen knowledge and skills through on-site coaching and mentoring. On-site coaching allows participants to improve their skills and creates opportunities to make broader improvements in the workplace. Health workers and managers in Karnali and Lumbini Provinces of Nepal were provided with regular coaching sessions to enhance their skills. A qualitative review was conducted to assess the feasibility and effectiveness of coaching and mentoring.

**Methods**

After implementing coaching sessions for more than two years, we interviewed 64 mentors, mentees, and management staff from nine municipalities on their experience. Interviews were transcribed, translated, and analyzed using QDA Miner Lite 2.0 in accordance with pre-defined themes.

**Results**

Coaching staff were provided with training, guidelines, and tools in support of their coaching activities. Guidelines were based on existing government protocols, where available, and/or developed by the project team. Health workers interviewed felt that their coaches had satisfactory technical skills, although some noted that skill levels were varied. On-site coaching appears to be operationally feasible, although issues related to remoteness, which also affects availability of health workers, create scheduling challenges. There is no one model of coaching that is suitable for all geographic zones and areas of technical skill. Overall, however, health workers and health managers expressed strong interest in learning and refreshing their skills and knowledge through coaching. They recognized the advantages of learning and practicing their skills on the job and found it very effective as a follow-up to formal training.

**Conclusions**

On-site coaching and mentoring are valued by health workers, and this appears to be an effective capacity-building strategy, both independently and as a follow-on to formal training. Sustainability of coaching initiatives in peripheral health facilities depends on the interest and capability in the municipal health offices. On-site coaching and technical assistance have the potential to strengthen both governance of local health systems and the skills and abilities of clinical service providers and public health officials in Nepal.

## O206. Intrapartum care measures and indicators for monitoring the implementation of World Health Organization (WHO) recommendations for a positive childbirth experience: a scoping review

### Lauren Vallely^1^, Anna Shalit^1^, Renae Nguyen^1^, Fernando Althabe^2^, Veronica Pingray^3^, Mercedes Bonet^2^, Meghan Bohren^4^, Caroline Homer^1^, Joshua Vogel^1^

#### ^1^Burnet Institute; ^2^World Health Organization; ^3^Institute for Clinical Effectiveness and Health Policy; ^4^University of Melbourne

*BMC Proceedings 2024*, **18(5):**O206

Submission ID #: IMNHC746

**Background**

In 2018, WHO released 56 evidence-based intrapartum care recommendations to improve quality of routine intrapartum care for women and newborns. Standardized monitoring of the implementation of clinical guideline recommendations is essential to the delivery of evidence-based care. Historically, indicators of intrapartum care, used for national reporting and international comparisons, have not measured quality of care nor reflected women’s experiences or satisfaction with care provided. We conducted a scoping review with the aim of identify all available studies describing measures or indicators used to monitor 41 intrapartum care practices described in the 2018 WHO recommendations.

**Methods**

We conducted a scoping review to identify studies reporting measures or indicators of intrapartum care published between 1 January 2000 and 28 June 2021. We searched MEDLINE, EMBASE, CINAHL, Cochrane Library, the Maternity and Infant Care Database, Global Index Medicus, and grey literature using structured search terms related to the 41 included recommendations, focusing on respectful and supportive care, and clinical practices performed throughout labor and birth. Data were extracted on study characteristics and indicator/measure characteristics (definition, numerator and denominator, application and measurement level, measurement approach). Indicators/measures identified were catalogued according to WHO recommendation for comparison across the 41 included WHO recommendations.

**Results**

150 studies were included, which described 1,331 intrapartum care measures. Measures were identified for 35 of the 41 included recommendations and across all domains of the WHO recommendations (care throughout labor and birth, first stage of labor, second stage of labor, third stage of labor). A total of 40.1% (534/1,331 measures) of measures were related to respectful maternity care. Most studies utilized a questionnaire or survey measurement approach (522/1,331 measures, 39.2%).

**Conclusions**

This scoping review presents a database of existing intrapartum care indicators/measures used to monitor the quality of intrapartum care globally. There is no clear consensus on a core set of measures for evaluating the practice of WHO’s intrapartum care recommendations. This review provides a foundation to support the development of a core set of internationally standardized intrapartum care measures for the WHO intrapartum care recommendations, highlighting key areas requiring consensus and validation, and measure development.

## O207. The indirect effects of the COVID-19 pandemic on maternal and newborn health services and outcomes in Latin America

### Erica Felker-Kantor, Annie Preaux, Arachu Castro

#### Tulane University School of Public Health and Tropical Medicine

*BMC Proceedings 2024*, **18(5):**O207

Submission ID #: IMNHC741

**Background**

The indirect effect of COVID-19 on maternal and newborn health outcomes is largely unknown. In Latin America, thousands of excess maternal deaths were estimated to take place as a result of the reduction in coverage of health care services during the early efforts to contain COVID-19 transmission, ranging from 1,200 to 8,000 excess deaths during the first year; thousands more neonatal deaths would be expected. The aim of this study was to estimate the average indirect effect of COVID-19 on maternal and newborn health services and outcomes in Latin America between 2019–2021. The study findings may be used to inform future policies and programs that advocate for strengthening maternal and newborn health services during a global health crisis.

**Methods**

We conducted secondary analysis of publicly available maternal and newborn health service and birth outcome and mortality data from Brazil, Colombia, Costa Rica, Dominican Republic, Ecuador, Mexico, and Peru for 2019–2021. We aggregated indicators to the province or department level and analyzed descriptively to examine time trends by month for each country. We employed an interrupted time series design using generalized estimating equations to estimate the average change in outcomes after the onset of the pandemic in 2020 (April 2020–December 2020) and in 2021 (January 2021–December 2021). We also examined monthly changes to estimate the percent change in expected versus observed outcomes since March 2020.

**Results**

Although the pandemic has likely impacted the quality of birth and death registration data in the studied countries, we found statistically significant effects for several outcomes since the pandemic began. The number of live births outside of health facilities, increased significantly in Colombia, Costa Rica, and Ecuador. The cumulative number of live births with low birth weight was between 6% and 13% lower than expected in Brazil, Colombia, Ecuador, Mexico, and Peru. In the Dominican Republic, the number of low birth weight births was 40% higher than expected, and the risk of stillbirth increased by 21%. Finally, the number of maternal deaths was significantly higher than expected in Colombia (63% higher), the Dominican Republic (40% higher), Ecuador (152% higher), and Mexico (80% higher).

**Conclusions**

The reduction in coverage that resulted from the efforts to contain COVID-19 transmission has impacted achievements made in the past two decades on maternal and neonatal health and mortality. Additional analysis should focus on determining which populations may have been disproportionately impacted during these periods.

## O208. Community follow-up to prevent recurrence of fistula

### Iyeme Efem, Kabiru Atta

#### EngenderHealth

*BMC Proceedings 2024*, **18(5):**O208

Submission ID #: IMNHC726

**Background**

Female genital fistula remains a global burden, and in Nigeria there are an estimated 12,000 new cases annually. Attention has focused on surgical repairs, community mobilization, and government guidelines. These target the condition, but not the “whole person.” Women with fistula may experience psycho-social consequences, and difficult deliveries that leave women with nerve damage. Noncompliance with post-repair recommendations can result in fistula recurrence. Integration of community follow-up into holistic fistula care can help to address these needs.

**Methods**

The USAID-funded Fistula Care Plus project reviewed the challenges of fistula recurrence in 2018 and initiated a strategy to target this issue at community level, in partnership with the Institute of Social Works of Nigeria (ISOWN). ISOWN medical social workers (MSWs) worked with surgeons to understand patients’ psychosocial needs and link them with community social workers (CSWs) for follow-up in their communities upon discharge. The strategy was piloted in Ilesa Osun State, and involved three components:

1. MSWs participate in patient history taking at admission, complementing clinical team questions with questions around socio-economic issues, such as employment, marital relationship, and family and/or community dynamics.
2. MSWs and CSWs triage information on the types of support needed after repair.
3. CSWs contact patients at home, confirm plans for visits, and speak with family members. During visits, CSWs educate the family on causes and recurrence of fistula. CSWs emphasize recurrence prevention as a collective family and community effort through understanding and supporting women for planned clinic follow-up visits

**Results**

Forty-five fistula clients were repaired and followed up by CSWs. Twenty had continued interactions with CSWs, returning to the center for follow-up visits. To date, there have been no reports of fistula recurrence among this group. One client returned to the facility for delivery. As a result of the home visits, a number of male partners of fistula patients became advocates for fistula prevention in the community.

**Conclusions**

Engaging social workers in targeted community follow-up of fistula patients can help prevent fistula recurrence and help address clients’ needs for holistic fistula intervention.

## O209. Last mile interventions to eliminate mother-to-child transmission in post-disaster settings: experience from rural Haiti

### Lourdes Marie Sandra Destin Damour, Brittany Aryeh, Anatole Manzi

#### Partners in Health

*BMC Proceedings 2024*, **18(5):**O209

Submission ID #: IMNHC725

**Background**

Despite global ambition to end the AIDS epidemic, women from countries with civil and political conflicts live a different reality. With over 150,000 adults living with HIV, Haiti has the largest epidemic in the Caribbean. Haitian women pay the highest price, constituting 60% of people living with HIV. Following the 2010 earthquake in Haiti, political instability, collective fear, and poor readiness of public health facilities limited access to HIV testing, which contributed to persistently high mother-to-child transmission of HIV. In collaboration with Haiti’s Ministry of Health, Zanmi Lasante launched a breakthrough project to increase access to HIV testing at hard-to-reach facilities. We describe a model intervention to increase early HIV diagnosis among pregnant women and changes in HIV incidence among exposed infants.

**Methods**

A landscape analysis was conducted between December 2020-March 2021 to assess the state of HIV testing among pregnant women from Saint-Marc District. Frequencies and percentages were used to describe coverage and facility readiness for HIV diagnosis and treatment. Thereafter, we launched a breakthrough improvement intervention, which consists of engaging women through dynamic outreach and sensitization, building competencies for lab technicians and community health care workers, and equipping new remote testing centers. Run charts were used to track changes over time.

**Results**

Between April-December 2021, 2083 pregnant women were tested for HIV—an almost three-fold increase in HIV testing. The percent of women tested during the first trimester increased from 50% at baseline to 71% after the intervention, *p*=0.012. The overall seropositivity was 5%, and the rate was as low as 2% among women who had their test during their first trimester. The positivity among exposed infants declined by 50%. The percent of exposed infants with positive PCR reduced from 10% to 0%. At three peripheral health facilities providing testing kits and lab reagents, building staff competencies in elimination of mother-to-child transmission of HIV, and establishing simplified care pathways all improved early diagnosis of HIV among pregnant women.

**Conclusions**

Political conflicts and other humanitarian crises are always associated with fear and anxiety, limiting utilization of health services. Interventions to eliminate mother-to-child transmission of HIV should include forming or equipping first-level facilities and pay attention to hard-to-reach communities.

## O210. Clinical evaluation of a novel, low-cost continuous respiratory rate and apnea monitor for newborns

### Joshua Coyle^1^, Maureen Mebo Valle^2^, Joseph Bailey^1^, Prince Mtenthaonga^2^, Rowland Mjumira^2^, Lucky Mangwiro^2^, Megan Heenan^1^, Jennifer Carns^1^, Noel Wilson^3^, Molly McCabe^3^, Robert Miros^3^, Maria Oden^1^, Rebecca Richards-Kortum^1^, Kondwani Kawaza^2^, Queen Dube

#### ^1^Rice360 Institute for Global Health Technologies; ^2^Kamuzu University of Health Sciences; ^3^3rd Stone Design; ^4^Ministry of Health, Malawi

*BMC Proceedings 2024*, **18(5):**O210

Submission ID #: IMNHC707

**Background**

Preterm birth interrupts physiological development, leaving newborns vulnerable to complications that require respiratory rate (RR) monitoring. Complications include: (1) respiratory distress syndrome, a leading cause of death in preterm newborns; (2) and apnea of prematurity, which affects 50% of preterm newborns and can indicate conditions such as sepsis, hypoglycemia, or anemia. When patient monitors (PM) are unavailable, the World Health Organization recommends counting breaths for 60 seconds to measure neonatal RR. In low-resource settings, patient monitors are often prohibitively expensive, and counting can be impractical due to high nurse-to-patient ratios.

Rice360 developed a low-cost, continuous RR/apnea sensor to accurately monitor RR in low-resource settings. The device detects breathing motion through two inertial measurement unit (IMU) sensors attached opposite the centerline of an abdominal belt. This study clinically evaluates the device against reference visual counting and compares performance to existing PM technology.

**Methods**

Preterm newborns at risk for apnea or needing respiratory support at Queen Elizabeth Central Hospital in Blantyre, Malawi, were enrolled for respiratory monitoring. Participants received continuous breath monitoring from the novel device and PM impedance pneumography (plus electrocardiogram, pulse oximeter, and temperature) for comparison against reference breath counting. A webcam recorded video which researchers annotated to count breaths and track the presence of motion or caregiving. Markings on the belt aid visual counting and automated image-processing counting for reference comparison. Reference breath counting and PM signal comparison inform and optimize respiratory detection algorithms on the novel device. Rice University’s Institutional Review Board and the College of Medicine Research and Ethics Committee in Malawi approved this study.

**Results**

Six newborns averaging 33 weeks [32–34 weeks] gestational age and enrollment weight of 1591g [1030–2023g] were monitored for an average of 73 minutes [59–92 minutes]. Six newborns had respiratory distress syndrome and four simultaneously received oxygen and aminophylline treatment. Fifteen one-minute epochs were manually selected among participants for preliminary comparison between total device-detected breaths and video-counted breaths. Average device error was -1.3±1.7 breaths.

**Conclusions**

This novel device can detect breaths consistent with visual counting. Clinical enrollment at Queen Elizabeth Central Hospital is ongoing to support device development.

## O211. Is delivery conducted in private health facilities and through cesarean section associated with higher neonatal mortality? Evidence from North Indian States

### Abhishek Kumar

#### Population Council

*BMC Proceedings 2024*, **18(5):**O211

Submission ID #: IMNHC690

**Background**

In India, institutional delivery increased from 42% in 2005–06 to 90% in 2019–21. This has had a huge impact on reducing neonatal mortality rate (NMR) in the country, from 37 per 1,000 live births in 2005 to 22 in 2019. However, this progress is uneven. In states like Bihar and Uttar Pradesh, which make up about one-fourth of the country population, NMR is more than 30 per 1,000 live births. Due to the insufficient and subpar public health system in these states, private health sectors are increasingly providing obstetric care and conducting C-section deliveries, which may have a negative impact on child health if the quality of service is compromised. The present study, therefore, examined the effect of private sector and C-section deliveries on NMR and early neonatal mortality rate (ENMR) in India and these two states.

**Methods**

This study used data from the National Family Health Survey, conducted 2019–2021. Last birth order children born in the last five years are the unit of the analysis. ENMR and NMR are outcome variables. Delivery in private health facilities and through C-section are predictors. Descriptive statistics and Cox proportional hazard models are applied in the analysis.

**Results**

In India and the states, about 30% deliveries are conducted in private health facilities, of which half are through C-section. ENMR and NMR are significantly higher among deliveries conducted in private health facilities—in Uttar Pradesh, ENMR and NMR is 18 vs. 29 per 1,000 live births and 22 vs. 33 per 1,000 live births, respectively, for public and private sector deliveries. The ENMR and NMR among C-section deliveries are higher than non-C-section. Hazards ratio depicts similar results—risk of neonatal death is significantly more likely among private sectors and C-section births.

**Conclusions**

In India, achieving the Sustainable Development Goals of reducing NMR to 12 per 1,000 live births by 2030 will highly depend on NMR reduction in Bihar and Uttar Pradesh. Private health sectors in these states are compromising with their quality of services. While public-private partnership for health care is a need of the hour, it needs continuous monitoring and review.

## O212. Promoting caregiver well-being as part of holistic nurturing care programming in Ghana and the Kyrgyz Republic

### Cat Kirk^1^, Lesley Oot^1^, Cholpon Abdimitalipova^1^, Joyce Jambeidu^1^, Kelsey Torres^1^, Romilla Karnati^2^, Enam Aidam^1^, Begimai Zhumgalbekova^1^, Fauzia Abukari^1^, Malia Uyehara^1^, Kathryn Beck^1^, Peggy Koniz-Booher^1^, Lutuf Abdul Rahman^1^, Andrew Cunningham^1^, Veronica Varela^1^, Tim Williams^1^

#### ^1^United States Agency for International Development; ^2^Save the Children

*BMC Proceedings 2024*, **18(5):**O212

Submission ID #: IMNHC671

**Background**

The stress of parenting can have negative consequences on caregiver well-being, and the period immediately after birth is a particularly high-risk period for maternal depression. The Responsive Care and Early Learning Addendum is designed for integration with nutrition and health counseling packages to promote holistic nurturing care. One training module focuses on supporting caregivers to engage in self-care and support-seeking—for their own well-being and also because a child’s healthy development largely depends on their caregiver’s capacity and well-being, particularly mothers. This study aims to understand the feasibility and acceptability of this training module among health workers and community volunteers, as well as initial effectiveness in reducing parenting stress in Ghana and the Kyrgyz Republic.

**Methods**

We are conducting mixed-methods implementation research across three regions in Ghana and two regions in the Kyrgyz Republic. A household survey of 478 caregivers of children ages 0–24 months was collected in March–July 2022 (baseline); endline was January–March 2023. Caregiver stress was measured using the Parenting Stress Index Short Form. Health worker and community volunteer experiences of being trained on the caregiver well-being module were captured in post-training qualitative interviews and surveys.

**Results**

At baseline, 26% of caregivers in Ghana and 7% in the Kyrgyz Republic reported high levels of parenting stress. Health worker/community volunteer feedback on the caregiver well-being module was positive and the majority felt prepared to counsel on caregiver well-being (*N*=792 health workers and *N*=1,900 volunteers trained). Many participants shared that there is a lack of focus on supporting caregivers in other trainings and in current services. Qualitative data provide insights into the feasibility of delivering the caregiver well-being component of the package and examining barriers (e.g., gender norms) and enablers (e.g., personal connection of the counselors to the importance of the topic) to its effective implementation.

**Conclusions**

The study provides evidence on the feasibility and acceptability of integrating caregiver well-being into nutrition counseling. Forthcoming research will provide evidence on the initial effectiveness of implementing the Responsive Care and Early Learning Addendum caregiver well-being component in the two health systems.

## O213. Investment case for small and sick newborn care in low- and middle-income countries: systematic analyses in Tanzania

### Rosemary Kamuyu^1^, Alice Tarus^1^, Felix Bundala^2^, Georgina Msemo^3^, Donat Shamba^4^, Nahya Salim^5^, Catherine Paul^6^, Robert Tillya^4^, Maria Oden^6^, Rebecca Richards-Kortum^6^, Timothy Powell- Jackson^1^, Joy Lawn^1^

#### ^1^London School of Hygiene & Tropical Medicine; ^2^Ministry of Health, Tanzania; ^3^Global Financing Facility; ^4^Ifakara Health Institute; ^5^Muhimbili University of Health and Allied Sciences; ^6^NEST360

*BMC Proceedings 2024*, **18(5):**O213

Submission ID #: IMNHC668

**Background**

Worldwide, 2.3 million neonates die during their first 28 days. Scale-up of Small and Sick Newborn Care (SSNC) has a high impact and is necessary to meet Sustainable Development Goals and Every Newborn targets. Many countries have committed to scaling up the World Health Organization’s Level 2+ newborn care unit for 80% of districts. There is demand for data-based investment cases to inform budgeting and resource mobilization. Tanzania has SSNC targets in their National One Plan III, requiring additional investment.

**Methods**

This investment case was spearheaded by Tanzania’s Ministry of Health and followed five steps, adapted from Global Financing Facility framework. First, national policy frameworks and guidelines were reviewed. Second, potential impact of scaling up SSNC were estimated using the Lives Saved Tool for 2025 and 2030. Third, set-up and running costs were estimated using the Activity-Based Sosting approach. Two scenarios were simulated: scenario A included costs of all new according to government standards, and Scenario B assumed half new and half-way to government standards. Fourth, we estimated the return on investment. Fifth, potential financing opportunities were identified and targeted.

**Results**

Total incremental cost was estimated at US$166 million for scenario A and US$90 million for scenario B. Set-up costs were driven by infrastructure (83%) and running costs by human resources (60%). At 85% endline coverage, estimated cumulative lives saved were 36,600 by 2025 and 80,000 by 2030. Neonatal mortality rate (NMR) was forecast to fall from 20 to 13 per 1000 live births, superseding the government 2025 target of 15, but falling short of the 2030 target of <12. The annual average incremental cost of scenario A represented 2.3% increase of total health expenditure with a potential return of between US$8–12 for every dollar invested.

**Conclusions**

Returns on investment for SSNC scale up are substantial and higher than other health investments given many deaths averted and long lifespan, noting that disability averted is not included. SSNC scale-up is considered affordable within the Tanzanian health budget, and the government has already allocated additional funding. Our proposed five-step SSNC investment case has potential for countries wanting to accelerate progress.

## O214. Newborn device planning and costing tool: systematic tool development to inform scale-up of small and sick newborn care in low-resource settings

### Alice Tarus^1^, Georgina Msemo^2^, Rosemary Kamuyu^1^, Donat Shamba^3^, Kara Palamountain^4^, Rebecca Kirby^4^, Edith Gicheha^5^, Meghan Kumar^1^, Timothy Powell- Jackson^1^, Christine Bohne5, Sara Liaghati Mobarhan^6^, Alison Morgan^2^, Maria Oden^5^, Rebecca Richards-Kortum^5^, Joy Lawn^1^

#### ^1^London School of Hygiene & Tropical Medicine; ^2^Global Financing Facility; ^3^Ifakara Health Institute; ^4^Northwestern University Kellogg School of Management; ^5^NEST360; ^6^United Nations Children’s Fund

*BMC Proceedings 2024*, **18(5):**O214

Submission ID #: IMNHC666

**Background**

High-quality neonatal care, a marker of health system function, is dependent on functional medical devices with context-specific specifications, adequate numbers, and ongoing maintenance. Devices designed for high-income settings may break, lack consumables, or be unused. Currently, systematic tools for device planning and costing that allows health system managers to utilize data to allocate budgets and mobilize resources at district and/or national levels are lacking. To close this gap, we developed a tool to support countries working towards Every Newborn target for scale-up of the World Health Organization’s (WHO’s) Level 2 neonatal care.

**Methods**

We followed a three-step process. First, we reviewed relevant tools and co-designed a tool focused on set-up costs for devices and furniture in a Level 2 newborn care unit with 10-bed unit capacity. Second, we determined unit costs and quantities of devices and furniture from multiple sources: guides from WHO and the United Nations Children’s Fund (UNICEF), multi-country facility assessment data, and guidelines and procurement price lists from three African countries. Third, the tool was refined with input from multi-disciplinary users and applied to estimate set-up costs for 146 districts and 25 regional referral hospitals in Tanzania.

**Results**

We adapted the stepwise approach of UNICEF’s Oxygen Planning Tool, which was the closest to our tool’s remit. Identified guidelines lacked data quantifying numbers of devices and instead estimated according to bed numbers. Our excel-based tool covered: (1) furniture and fixtures (18 default but editable items); (2) neonatal devices (16 items with 11 qualified for LMIC contexts); and (3) device installation training and accompanying dashboard displaying costing results. Tool usage in Tanzania showed feasibility of use in less than a week for data collection and generation of cost reports. Cost to set up furnishings and provide devices for a new WHO Level 2 newborn unit in Tanzania was estimated at US$77,000 and cost per capita was US$0.32.

**Conclusions**

This costing tool has the potential to enable data-based planning and budgeting for scaling up Every Newborn Action Plan/WHO Level 2 small and sick newborn care, based on national plans and guidelines. More data on device-to-baby ratios and unit costs from other regions will improve the tool’s default estimates.

## O215. Using a Lot Quality Assurance Sampling (LQAS) survey to prioritize interventions to impact reproductive, maternal, and child health-seeking behaviors in Manicaland Province, Zimbabwe

### Absolom Mbinda^1^, Elikem Togo^1^, Jephiter Tsamwi^1^, Nyaradzo Debra Muhonde^1^, Gladwin Muchena^1^, Lucia Gumbo^2^, Tendai Lincoln Nyafesa^3^, Catherine Packer^1^, Mario Chen^1^, Alissa Bernholc^4^, Marya Plotkin^1^

#### ^1^FHI 360; ^2^United States Agency for International Development; ^3^Ministry of Health and Child Care, Zimbabwe; ^4^FHI Clinical

*BMC Proceedings 2024*, **18(5):**O215

Submission ID #: IMNHC662

**Background**

In Manicaland Province, Zimbabwe, maternal mortality is 505 deaths per 100,000 live births. Approximately 42% of the population belongs to the Apostolic faith, which prohibits accessing modern health services, impacting women’s health. The USAID-funded Mhuri/Imuli project, led by FHI 360, works with the Ministry of Health and Child Care to improve maternal, newborn, and child health (MNCH) in Manicaland Province. Mhuri/Imuli conducted a community-based Lot Quality Assurance Sampling (LQAS) survey to assess RMNCH-related care-seeking behaviors in Manicaland.

**Methods**

The LQAS survey was conducted in 2019 among 924 women aged 16–49, who delivered between May 1, 2018 and April 30, 2019, living in catchment areas of 47 project-supported health facilities. Interviews were conducted in or near women’s homes by trained researchers. Following LQAS methodology, 19 eligible women per area were selected randomly and a minimum number of acceptable responses was set to determine which catchment areas failed to meet performance thresholds. We also obtained province-level estimates of each indicator with corresponding 95% CIs. Sampling weights were used to account for different sizes of catchment area populations.

**Results**

The average age of respondents was 28.3 years. Most (92.4%) were married and of the Apostolic faith (67.2%). All 47 catchment areas demonstrated lower than acceptable levels of 3+ doses of intermittent preventive treatment of malaria during pregnancy. A third (30.2%, 95% CI: 26.8–33.6) of the women had 3+ doses. All catchment areas were below the acceptable threshold for having a birth plan; 35% of respondents indicated having a birth plan. Twenty-four catchment areas failed to meet the threshold for giving birth in a health facility. Women’s role in decision-making was low regarding how many children to have (17.6%, 95% CI: 14.7–20.5). Findings indicate that critical health services are being missed during and after pregnancy.

**Conclusions**

The survey highlighted care-seeking behaviors, which impact MNCH outcomes. Based on findings, behavior change and norms shifting programs were launched, including engaging Apostolic faith leaders and a family life school approach, which addresses RMNCH knowledge and gender norms. The LQAS methodology was useful in determining program gaps and should be considered in other settings.

## O216. Factors associated with labor companionship in Rwanda

### Emmy Basonga^1^, Gerard Kaberuka^2^, Uwamariya Josee^3^, Kathryn Mimno^3^

#### ^1^University of Rwanda; ^2^Independent Consultant; ^3^Intrahealth International

*BMC Proceedings 2024*, **18(5):**O216

Submission ID #: IMNHC661

**Background**

Evidence showed that labor companionship is beneficial for childbirth experience and outcomes. However, little is known about this practice in Rwanda. This study sought the perspectives and experiences of women and health care providers regarding labor companionship and possible associated factors in four hospitals in Rwanda (Muhima, Remera Rukoma, Kacyiru, and the University Teaching Hospital of Kigali).

**Methods**

A prospective mixed-method study was used. A total of 393 mothers at 48 hours postpartum were recruited using a simple random sampling method. Data were collected from January to March 2022. Key informant interviews were conducted with 23 health care providers and 16 mothers. Participants’ demographic information was presented using frequencies and percentages. The Chi-square test and logistic regression analyses were used to study the relationship between a companion’s presence during labor and the delivery and labor experience. Thematic analysis was applied to interpret qualitative data

**Results**

Of the 393 mothers who participated in the study, 97% went to health facilities with their companions of choice, 47% (*n*=200) were allowed to have their companions during labor, and only 11.20% (*n*=42) of mothers had their companions during delivery. The benefits of labor companionship described by those mothers who had their companion during delivery included increased happiness (81.90%), reduced loneliness (88.98%), prevention of abuse (36.44%), reduced labor pain (32.20%), and shortened labor length (11%). The barriers to labor companionship cited by women who didn’t want to stay with their companion were fear of embarrassment (47%), fear of gossip (14%), and desire to maintain privacy (40%). A high level of companionship during labor and delivery was associated with advanced mother’s age (>36 years), high level of education, high economic status, and planned pregnancies. Both mothers and health care providers perceived birth companionship as important. Lack of space to allow companions in the delivery room and poorly arranged infrastructure to maintain women’s privacy—especially during delivery—were cited as key challenges to companionship.

**Conclusions**

Despite the benefits of labor and childbirth companionship, the practice is still low in Rwanda. The elaboration and implementation of companionship policies along with social behavior change interventions can improve companionship practice, childbirth experience, and outcomes.

## O217. The unrecognized maternal mental health crisis among Female Sex Workers (FSWs): the toll of suicides during pregnancy

### Brian Willis, Wendy Macias-Konstantopoulos

#### Global Health Promise

*BMC Proceedings 2024*, **18(5):**O217

Submission ID #: IMNHC657

**Background**

There are many studies on mental health among female sex workers (FSW), including suicide ideation and suicide attempts, but no data on actual deaths by suicide. Notably, there are three major gaps in the literature and data: studies on FSW do not identify deaths by suicide; most studies on mental health of FSW do not assess the maternal mental health of FSW; and studies on death of women by suicide, including maternal suicides, do not include FSW. Further, the risks of maternal suicide may be increasing during the pandemic and the current food insecurity crisis, and additionally the high rates of global inflation.

**Methods**

The study used the Community Knowledge Approach to identify causes of mortality among FSW in eight low- and middle-income countries. This approach was employed to identify deaths of any cause among communities of FSW. Study participants, recruited by in-country sex worker organizations, provided detailed information about FSW deaths in their communities. Deaths across 2014–2019 were combined for each country and age group. Based on reported details, deaths were coded and classified as abortion, murder, HIV/AIDS, suicide, accidents, and other causes.

**Results**

174 of 288 (60.4%) deaths by suicide were maternal deaths (MD): 103 while pregnant, 35 were <60 days postpartum, and 36 were 2–12 months postpartum.

The leading cause of the 1,320 MD reported was abortion, resulting in 750 deaths (56.8% of all MD), followed by suicide (12.8% of all MD).

Of the 174 maternal suicides, 103 (59.2% of all MD) occurred during pregnancy, 35 (20.1% of all MD) within two months postpartum, and the remaining 36 (20.7% of all MD) suicides are late maternal deaths that occurred 2–12 months postpartum.

**Conclusions**

Compared to other populations, we found most maternal deaths by suicide among FSW occurred during pregnancy, in contrast to most maternal deaths by suicide which occurred during the postpartum period. These findings indicate an urgent need for maternal mental health services for FSW, which must also include food support. This study helps to address gaps in equity because FSW are severely underrepresented in mental health interventions and do not have equitable access to mental health services.

## O218. Maternal acceptability and perceptions of infant drug exposure from the dapivirine vaginal ring for HIV prevention

### Imogen Hawley^1^, Marie Stoner^1^, Prisca Mutero^2^, Florence Mathebula^3^, Doreen Kemigisha^4^, Zayithwa Fabiano^5^, Linly Seyama^5^, Rachel Scheckter^6^, Mei Song^7^, Ivan Balan^8^, Ariane Van Der Straten^9^, Elizabeth T. Montgomery^1^

#### ^1^Research Triangle Institue International; ^2^University of Zimbabwe; ^3^Wits Reproductive Health and HIV Institute; ^4^Makerere University; ^5^Kamuzu University of Health Science; ^6^FHI 360; ^7^Magee-Womens Research Institute; ^8^Florida State University College of Medicine; ^9^ASTRA Consulting and Center for AIDS Prevention Studies UCSF

*BMC Proceedings 2024*, **18(5):**O218

Submission ID #: IMNHC656

**Background**

The dapivirine vaginal ring (“ring”) for HIV prevention is approved in several African countries, and safety trials are ongoing to expand its indication for pregnant and breastfeeding people. We qualitatively explored acceptability of the ring among trial participants in Africa, who were at a late stage of pregnancy or breastfeeding, to inform future ring rollout considerations for these populations.

**Methods**

Between 2020–2021, in two Phase 3b clinical trials of the ring among pregnant and breastfeeding participants—MTN-042/DELIVER (safety, pregnancy) and MTN-043/B-PROTECTED (safety and drug detection, breastfeeding)—in-depth interviews were conducted to assess participants’ experiences with and attitudes toward study products. A subset of participants assigned to the ring were randomly selected for interviews in MTN-042 (~38 weeks gestation and two weeks after product dispensation, *n*=33) and purposively selected in MTN-043 (~12 weeks after product dispensation, *n*=40) in Malawi, South Africa, Uganda, and Zimbabwe. Audio recordings were transcribed, translated, coded, and analyzed thematically.

**Results**

Overall, participants believed the ring protected themselves and their babies against HIV. Reported ring use was motivated by participants’ desire to safeguard their own health to take care of their family. Many participants demonstrated an inaccurate understanding of how their babies were protected: they assumed the baby directly received protective levels of dapivirine via the umbilical cord or breastmilk. Despite beliefs that the ring was protective to the baby, there were concerns about the potential long-term impacts of drug exposure on the baby, particularly among pregnant participants. Concerns included fears that the baby would be born with “birth defects,” “abnormalities,” “disabilities,” or have developmental delays. Additional qualitative data from MTN-042 participants at earlier stages of pregnancy are forthcoming.

**Conclusions**

If the ring is found safe to use in pregnancy and breastfeeding, efforts to promote ring use should address comprehension of how the ring protects the user, and indirectly prevents vertical HIV transmission, as well as concerns about potential long-term health impacts for babies exposed to dapivirine during pregnancy or breastfeeding. Efforts to address misconceptions around babies’ exposure to dapivirine may increase ring acceptability and prevent the potential spread of misinformation.

## O219. Causes of maternal mortality among female sex workers: results of first ever multi-country study

### Brian Willis, Wendy Macias-Konstantopoulos

#### Global Health Promise

*BMC Proceedings 2024*, **18(5):**O219

Submission ID #: IMNHC655

**Background**

The majority of studies on female sex workers (FSW) focus on morbidity, while data on mortality are scarce. In low- and middle-income countries, where civil registry and vital statistics data are often incomplete and FSW may not be identified as such in official registries, identifying causes of mortality among FSW has proven challenging.

**Methods**

The study used the Community Knowledge Approach to identify causes of mortality among FSW in eight low- and middle-income countries. This approach was employed to identify deaths of any cause among communities of FSW. Study participants, recruited by in-country sex worker organizations, provided detailed information about FSW deaths in their communities. Deaths across years 2014–2019 were combined for each country and age group. Based on reported details, deaths were coded and classified as abortion, murder, HIV/AIDS, suicide, accidents, and other causes.

**Results**

1,280 FSW participated in 165 group meetings, through which 2,112 FSW deaths were identified. Of these reported deaths, 57.9% occurred in 2019 and 57.2% were among women aged 20–29. Causes of death included: abortion (35.5%), other maternal causes (16.6%), suicide (13.6%), murder (12.5%), unclassified causes (11.6%), HIV/AIDS (7.9%), and accidents (3.2%). A total of 3,659 children lost their mothers.

**Conclusions**

The current study is the first multi-country study on FSW mortality and is the single largest study of FSW deaths ever reported. Maternal death comprised the leading cause of FSW mortality in our sample. The findings of this study address a knowledge gap about causes of mortality among FSW and underscore the urgent need for country-specific programs to prevent FSW deaths. This methodology can be used by sex worker organizations and other nongovernmental organizations to identify unrecognized patterns and clusters of FSW deaths in near-real time and urgently steer targeted preventive strategies, most notably maternal health interventions. This study helps to address gaps in equity because FSW are severely underrepresented in public health interventions and do not have equitable access to health care services that would have prevented these causes of death. Additional research is urgently needed to identify any changes in mortality patterns among FSW due to the pandemic.

## O220. The clinical presentation and detection of tuberculosis during pregnancy and in the postpartum period in low- and middle-income countries: a systematic review and meta-analysis

### Grace Simpson, Moira Philip, Joshua Vogel, Stephen Graham, Alyce Wilson

#### Burnet Institute

*BMC Proceedings 2024*, **18(5):**O220

Submission ID #: IMNHC650

**Background**

For women infected with Mycobacterium tuberculosis (TB), pregnancy is associated with an increased risk of developing or worsening TB disease. TB in pregnancy increases the risk of adverse maternal and neonatal outcomes; however, the detection of TB in pregnancy is challenging. We aimed to identify and summarize the findings of studies regarding the clinical presentation and diagnosis of TB during pregnancy and the postpartum period (within six months of birth) in low-and middle-income countries.

**Methods**

A systematic review was conducted searching Ovid MEDLINE, Embase, CINAHL, and Global Index Medicus databases (21/07/2020). We included any primary research study of women diagnosed with TB during pregnancy or the postpartum period in low-and middle-income countries that described the clinical presentation or method of diagnosis. Meta-analysis was used to determine pooled prevalence of TB clinical features and health outcomes, as well as detection method yield.

**Results**

Sixty-two studies of 2,664 women from 23 countries were included; 75.5% of women were from South Africa or India. Of those with known HIV status, 47.2% were HIV-positive. For 1,615 women where TB type was reported, pulmonary TB was most common (80.5%). Most studies did not report the prevalence of presenting clinical features. Where reported, the most common were cough (62%), fever (36%), or a history of prior TB diagnosis (32%). Having a recent TB contact was found in 24% of women. Only four studies screened for TB using diagnostic testing for asymptomatic antenatal women and included mainly HIV-positive women; 76% of women with bacteriologically confirmed TB did not report symptoms and only one was HIV-negative. Chest X-ray had the highest diagnostic yield: 57% abnormal results of 2,934 women tested.

**Conclusions**

Screening pregnant women for TB-related symptoms and risk factors is important but detection yields are limited. Chest radiography and bacteriological detection methods can improve this, but procedures for optimal utilization remain uncertain in this at-risk population.

## O221. Immediate kangaroo mother care: process and costs for implementation readiness at five hospitals in Uganda

### Melissa Medvedev^1^, Victor Tumukunde^2^, Charity Kirabo-Nagemi^2^, Elizabeth Ekirapa-Kiracho^3^, Giulia Greco^4^, Ivan Mambule^2^, Kenneth Katumba^2^, Peter Waiswa^3^, Cally Tann^4^, Diana Elbourne^4^, Elizabeth Allen^4^, Catherine Pitt^4^, Joy Lawn^4^

#### ^1^University of California, San Francisco; ^2^Medical Research Council; ^3^Makerere University; ^4^London School of Hygiene & Tropical Medicine

*BMC Proceedings 2024*, **18(5):**O221

Submission ID #: IMNHC649

**Background**

Preterm birth complications result in over 1 million child deaths annually, mostly in low- and middle-income countries (LMICs). A World Health Organization (WHO)-led trial in hospitals with intensive care reported reduced mortality at 28 days among newborns weighing 1,000–1,799g who received immediate kangaroo mother care (iKMC) compared to those who received standard care, prompting calls for scale-up of iKMC. Evidence is needed regarding the process and costs of implementing iKMC, particularly in hospitals without intensive care.

**Methods**

We describe actions undertaken to safely implement iKMC, estimate financial and economic costs of essential resources and infrastructure improvements, and assess readiness for newborn care after these improvements at five hospitals participating in the OMWaNA trial in Uganda. We estimated costs from a health service provider perspective using hospital and project records, and explored cost drivers and cost variation across hospitals. We assessed readiness to deliver care for small and sick newborns (WHO Level 2) using a health facility assessment tool developed by Newborn Essential Solutions and Technologies, in partnership with United Nations Children’s Fund, and co-designed with four African government teams.

**Results**

Following the addition of space to accommodate adult beds for iKMC, floor space in the neonatal units ranged from 42–106 square meters. Total costs of improvements were lowest at the larger, national referral hospital (financial: $31,354; economic: $46,193; in 2020 US$), and varied across the four smaller hospitals (financial: $68,330–$95,796; economic: $99,278–$160,926) according to the amount of remodeling or construction required. Even after improvements, neonatal care capacity and availability of essential equipment and supplies varied widely.

**Conclusions**

These five Ugandan hospitals required substantial resource inputs to safely implement iKMC. A comparable 20-bed neonatal unit could be expected to require broadly similar investments to the four smaller hospitals in our study. Before widespread scale-up of this intervention in LMICs, the affordability and efficiency of this investment must be assessed, considering variation in costs across hospitals and levels of care. These findings should help inform planning and budgeting as well as decisions about if, where, and how to implement iKMC, particularly in LMICs where space, devices, and specialized staff for newborn care are often unavailable.

## O222. Increasing immunization coverage and reducing zero dose among children aged 0 to 59 months through digitally empowered community health workers in Uganda and Kenya

### Kezia K’Oduol^1^, Sarah Lindsay^1^, Stella Kanyerere^1^, Zipporah Moraa Nyangacha^1^, Agnes Watsemba^1^, Harriet Andrews^1^, Jennifer Hyman^1^, Molly Christiansen^1^, Charles Syengo^2^, Isaac Malonza^2^, Emilie Chambert^1^, Raymond Mutisya^2^

#### ^1^Living Goods; ^2^Urban Research And Development Centre for Africa

*BMC Proceedings 2024*, **18(5):**O222

Submission ID #: IMNHC646

**Background**

Despite efforts to attain full immunization coverage, many children in underserved communities in Uganda and Kenya remain unvaccinated or under-vaccinated partly due to individual- and community-related barriers in accessing services, including failure to adhere to immunization schedules and misconceptions about vaccines. Between July 2018 and December 2020, with Gavi funding, Living Goods collaborated with Ministries of Health to implement a digitally-enabled community health worker (CHW) project to increase vaccine uptake and access for children aged 0–59 months in 19 Ugandan districts and five Kenyan counties.

**Methods**

The intervention entailed: (1) training CHWs and their supervisors on the under-five immunization package; (2) CHWs using a mobile phone application that integrated immunization modules to assess under-five vaccination status, refer, and follow-up; (3) health education, awareness creation, and SMS vaccination reminders to caregivers; and (4) supervision visits and financial incentives to CHWs; and 5) vaccination outreach. Data was obtained from a two-time serial cross-sectional community-based survey at baseline (Kenya: *n*=1,718; Uganda: *n*=1,994) and endline (Kenya: *n*=1,727; Uganda: *n*=2,999) that used a three-stage sampling design and structured questionnaires adapted from national demographic surveys. Data were analyzed using descriptive statistics and cross-tabulations.

**Results**

There was a significant increase in full immunization coverage of children aged 12–23 months from 50% ([95% CI: (46.3, 53.7)] to 67.9% [95% CI: (65.5, 70.1)] in Uganda and from 43.9% [95% CI: (37.9, 50.2)] to 74% [95% CI: (70.4,77.4)] in Kenya. During this period, the proportion of children aged 6 weeks to 59 months who never received Penta-1 (zero dose) significantly declined from 13.2% to 7% in Uganda and 5.2% to 0.7% in Kenya. There was increased adherence to vaccination schedules; immunization dropout rate for Penta 1–3 declined from 18.3% to 10.9% in Uganda and 6.9% to 5.1% in Kenya; unvaccinated children (birth to 59 months) declined from 5.9% to 2.6% in Uganda and 0.2% to 0.06% in Kenya.

**Conclusions**

Digitally equipped CHW programs could potentially increase immunization coverage and adherence to vaccination schedules, and reduce zero dose cases in underserved communities, offering a high-impact approach to reduce morbidity and mortality from vaccine-preventable diseases in young children.

## O223. Improving health literacy through group antenatal care: results from a cluster randomized controlled trial in Ghana

### John Williams^1^, Jody Lori^2^, Vida Ami Kukula^1^, Cheryl Moyer^2^, Georgina Amankwah^1^, Nancy Lockhart^2^, Veronica Esinam Awo Apetorgbor^1^, Bidisha Ghosh^2^, Liya Liu^2^, Elizabeth Awini^1^, Katherine James^2^, Ruth Zielinski^2^

#### ^1^Ghana Health Service; ^2^University of Michigan

*BMC Proceedings 2024*, **18(5):**O223

Submission ID #: IMNHC643

**Background**

The purpose of this study was to address the enormous gaps in health literacy and the resulting poor outcomes among pregnant women in Ghana to determine the efficacy of group-based antenatal care (ANC) on increasing health literacy and improving maternal and newborn outcomes. The objective was to examine women’s understanding of health messages, their appraisal of health information, and their engagement with the health system using a multi-dimensional, validated tool—the Maternal Health Literacy 12-point scale. We hypothesized that pregnant women randomized into group ANC will exhibit a greater increase in health literacy than women who received routine, individual ANC.

**Methods**

We conducted a five-year cluster randomized controlled trial at 14 health facilities in the Eastern Region of Ghana. These health facilities were randomized into either group-based or routine, individual care. The women were recruited into one of the groups depending on the facility they used for their initial ANC visit. The 12-item version of the Maternal Health Literacy scale was used to collect data at two time points: baseline data (T0) prior to ANC intervention, and post-birth (T2) at six months postpartum. Logistic regression was conducted to compare changes in health literacy from T0 to T2 within and between groups using Stata 17.0.

**Results**

Women in both the intervention and control groups improved their composite health literacy scores over time (*p*=< 0.0001). Women in the intervention group scored significantly higher on five individual items and on overall composite scores (*p*=< 0.0001).

**Conclusions**

The effectiveness of ANC depends on the multidimensional concept of health literacy. Initially considered only as a patient’s ability to read and understand written information, it is now more broadly defined as a person’s ability to acquire or access information, understand it, and use the information in ways that promote and maintain good health. While health literacy scores improved for all women attending ANC, women randomized into group ANC exhibited greater improvement in overall health literacy postpartum compared to those receiving routine individual care. Lifesaving information provided during ANC must be presented in an understandable format to prevent women and newborns

## O224. Engaging women in research on assisted vaginal birth

### Maria Regina Torloni^1^, Ana Pilar Betran^2^, Mercedes Bonet^2^, Newton Opiyo^2^, Meghan Bohren^3^

#### ^1^São Paulo Federal University; ^2^World Health Organization; ^3^University of Melbourne

*BMC Proceedings 2024*, **18(5):**O224

Submission ID #: IMNHC635

**Background**

Assisted vaginal birth (AVB) can be a lifesaving intervention that can avoid second stage cesarean section and its associated risks, especially in settings with limited access to comprehensive obstetric care. Important knowledge gaps persist on how to safely reintroduce/increase AVB, and improve/maintain AVB knowledge/skills in low- and middle-income countries, and how to customize in-country training/support. It is crucial to understand women’s views about research in this area. We report the findings of interactive workshops convened by the World Health Organization (WHO) to gather women’s views on this topic.

**Methods**

WHO first led qualitative and quantitative evidence syntheses on interventions to increase AVB use, and associated barriers/facilitators. WHO then convened a technical consultation with professionals to identify research gaps to safely increase AVB use. Finally, WHO conducted four interactive workshops with women’s representatives to gather their views/perspectives about the research gaps proposed by the professionals. We report here the results of these workshops.

**Results**

The four virtual workshops, conducted in three languages, collected the views of 30 women’s representatives from 27 countries. The women viewed research on AVB safety as a priority, including prevention of physical and mental short- and long-term adverse maternal and perinatal outcomes. They emphasized the importance of reframing labor/childbirth as a physiological phenomenon with individual variability that should be respected, with women and babies at center stage, and where interventions (including AVB) should be used rationally, only if and when needed. To women, health providers managing labor/birth should be trained to use effective and respectful communication, including how to engage women in shared decision-making, to ensure trusting relationships between providers and women who need AVB. Women’s representatives emphasized the importance of including women in establishing core outcomes for AVB research. Additional research priorities included assessing, and effective interventions to improve, women’s knowledge/attitudes about AVB risks and benefits, appropriate use of informed consent, and how to inform women about their rights to respectful maternity care and choice of route of delivery.

**Conclusions**

Women have clear views and concerns about priorities in AVB research. Women representatives need to be included at the planning stage of future studies in this area.

## O225. An innovative strategy to address social norms among health care providers and community members to improve maternal, newborn, and child health in Nampula Province, Mozambique

### Ester Sumbana^1^, Edgar Bernardo^2^, Megan Lydon^1^, Fulgencio Estrada^1^, Geoffrey Ezepue^1^

#### ^1^FHI 360; ^2^HOPEM Network

*BMC Proceedings 2024*, **18(5):**O225

Submission ID #: IMNHC630

**Background**

In Mozambique, discriminatory social and gender norms underlie many maternal, newborn, and child health (MNCH) challenges, including lack of access, disrespectful care, gender-based violence, and lack of male involvement. The Alcançar Project, a five-year, USAID-funded activity in Nampula Province, integrated a novel gender and social inclusion (GESI) approach, prioritizing synchronized female empowerment and male engagement.

**Methods**

In September 2019, a GESI analysis was conducted to identify policies and norms that affect MNCH in Nampula. The team developed a GESI toolkit—tailored to address local norms and identified gaps that included guided dialogue, reflection, and education sessions—designed for community and health care settings. Regular sessions have been held with health facility and community groups across 11 districts to help participants better understand how social norms affect MNCH. The team regularly collects feedback from participants following completion of three-month dialogue sessions.

**Results**

Since October 2020, 1,064 participants have completed the community-based dialogue series and another 537 are currently engaged. Additionally, 46 providers, including doctors and district directors, have participated in facility-based sessions. Community members described better understanding the harmful effects of certain social norms on their health, such as home delivery. Women shared that they felt happier and their husbands were more involved in family health matters. Providers explained that they better understood the importance of respectful care, male engagement, and protecting patients from bribery and obstetric violence. When designing a GESI toolkit, the Alcançar team recommends as a best practice that sessions do not aim to change culture but rather support participants on a journey of self-discovery. Through implementation, the team learned that GESI sessions in health facility settings are more successful with foundational policies, guidelines, and leadership supporting shifting norms.

**Conclusions**

The GESI toolkit shows promise in shifting social norms and related behaviors in health care and community settings to improve MNCH. Provincial and district authorities have shown interest in this intervention, and it is currently being scaled up to the remaining 12 districts in Nampula Province and six districts in neighboring Zambezia Province. It is critical to incorporate social norms interventions in MNCH programs to address underlying barriers.

## O226. Improving quality of care for mothers and babies in Kenya through a digital SMS-based feedback platform to amplify client voices in their health care experience

### Anneka Wickramanayake

#### Jacaranda Health

*BMC Proceedings 2024*, **18(5):**O226

Submission ID #: IMNHC626

**Background**

Access to respectful maternity care for women is a fundamental right, yet remains a challenge in many contexts. In Kenya, disrespectful attitudes from providers have been described by women as barriers to care-seeking.

Jacaranda Health’s Promoting Mums through Pregnancy Through SMS (PROMPTS), a two-way SMS-based platform, is a channel for women to provide feedback on the quality of care they receive during maternal health visits. This data is aggregated and shared with facilities and counties so they are able to reflect and act upon it, improving quality maternal health care and amplifying mothers’ voices as they interact with Kenya’s health care system.

**Methods**

Jacaranda Health is undergoing a randomized control trial and a process evaluation, assessing the impact of its package of interventions, including PROMPTS and in-facility mentorship for emergency obstetric and newborn care. Twenty facilities across eight counties were enrolled to receive the intervention. PROMPTS enrolls mothers when they seek maternity care at a study hospital and asks users if they received respectful care at their last visit to a health center. Feedback on respectful care was captured from January to August 2022. The process evaluation qualitatively captured the perspective of providers about how feedback has changed their actions. Guidelines were developed by an external consultant and approved by an institutional review board.

**Results**

Results from PROMPTS’ quality of care survey demonstrate an increase in the percent of women who reported respectful care from 92% to 97% in study facilities over the eight-month period. Qualitative inquiry indicated that providers were receptive to feedback, motivated by positive feedback, and encouraged to improve in response to criticism. Providers understand that word of mouth encourages patients to attend a specific health center, and therefore want to enhance the perception of their facilities.

**Conclusions**

Respectful maternity care encourages health-seeking behavior during women’s pregnancy journey. Feedback from clients is an effective way to inform facility leadership of the perception of the quality delivered in their facilities and identify areas for improvement. The PROMPTS platform provides a direct link to mothers and can be used to prioritize improvements in quality of care in Kenyan hospitals.

## O227. Maternal death surveillance and response and perinatal death surveillance and response in Sierra Leone: towards an integrated MPDSR approach

### Abibatu Kamara^1^, Tom Sesay^1^, Zainab JuhehBah^1^, Edwin Mangala^2^, Mariama Mustapha^2^, Musu Rachael Cole^1^, Sattu Issa^1^, Pity Kanu^1^

#### ^1^Ministry of Health and Sanitation, Sierra Leone; ^2^United Nations Children’s Fund

*BMC Proceedings 2024*, **18(5):**O227

Submission ID #: IMNHC623

**Background**

In Sierra Leone, the maternal mortality ratio is estimated at 717 per 100,000 live births, and the neonatal and perinatal mortality rates at 31 per 1,000 live births and 34 per 1,000 total births, respectively. These are among the highest in the world. Sierra Leone established a nationwide maternal death surveillance and response (MDSR) system in 2016 to address these poor indicators. The MDSR is a platform for collecting, analyzing, and reviewing data that will inform actions to prevent maternal deaths. The perinatal death surveillance and response (PDSR) system was established in four regional hospitals in 2019 and rolled out to 12 additional hospitals in 2021. Currently, both systems operate independently.

**Methods**

District health management teams are notified of maternal deaths within 24–48 hours of occurrence. Maternal deaths are investigated within 72 hours and reviewed monthly. A line-listing consisting of demographic, antenatal, intrapartum, and postpartum information of all confirmed maternal deaths occurring in the district is generated monthly and submitted to the national level. The hospital PDSR committee is notified of perinatal deaths and at least two deaths are reviewed weekly.

**Results**

Maternal death notification has increased by over 300% between 2014 and 2021. Proportion of maternal deaths investigated and reviewed has increased from 61% in 2016 to 99% in 2021. MDSR has contributed to an improvement in maternal health coverage and impact indicators. Some notable gains between 2013 and 2019 include: increased institutional deliveries (from 54% to 83%), skilled birth attendance (60% to 87%), postnatal care within two days of delivery (73% to 86%), and almost 40% reduction in maternal mortality. The PDSR system is established in 14 out of 16 district hospitals, and 90% of hospitals are conducting weekly reviews and submitting monthly PDSR data to the national level.

**Conclusions**

Integrating the MDSR and PDSR processes is critical to ensuring a more robust response and accelerating progress in maternal and perinatal health indicators in the country. The integrated process needs to be part of quality improvement initiatives at all levels.

## O228. Validation of maternal report of iron folic acid supplementation during pregnancy in Rural Nepal

### Joanne Katz^1^, Emily Bryce^2^, Tsering Pema Lama^3^, Subarna Khatry^3^, Melinda Munos^1^

#### ^1^Johns Hopkins Bloomberg School of Public Health; ^2^Jhpiego; ^3^Nepal Nutrition Intervention Project Sarlahi

*BMC Proceedings 2024*, **18(5):**O228

Submission ID #: IMNHC617

**Background**

Measuring and tracking coverage of nutrition-related services provided during antenatal care (ANC) is important for improving evidence-based interventions. For many low-income countries, the Demographic and Health Surveys provide nationally representative data. However, it is unclear whether women can report accurately what services they received during pregnancy, often with a recall period of months to years.

**Methods**

We enrolled pregnant women at their first ANC visit at five health posts in Sarlahi District, Nepal. Women from the community were trained to observe all ANC visits the enrolled women attended at these health posts and record services received (including whether iron folic acid [IFA] supplements were provided and if so, how many tablets). At six months postpartum, enrolled women were visited at home and asked about whether they received any IFA in pregnancy and how many tablets. The reported data were compared to observed. Individual-level validity was measured by sensitivity, specificity, and area under the curve (AUC). Population validity was measured by the inflation factor (the survey prevalence divided by the observed prevalence).

**Results**

Almost all women (95.8%) were observed to receive IFA and 96.2% reported receiving them. Women were observed to receive a mean of 73 tablets in pregnancy. However, women over-reported the number of tablets they received by a mean of 45 tablets (they under-reported the number at low levels of true receipt and over-reported at 120 or more tablets observed to be received). Only 2.9% of women were observed to receive 180 or more tablets but 19.4% reported receiving this many or more. The AUC did not exceed 0.60 for any IFA receipt or when stratified by number of tablets received, indicating poor individual-level accuracy.

**Conclusions**

In this population, there was poor individual- and population-level accuracy of household survey estimation of number of IFA tablets received in pregnancy. The household survey overestimated the number of IFA tablets given to women in pregnancy compared with the gold standard of observed receipt of IFA. Improved measures of IFA receipt are needed to accurately track IFA coverage in pregnancy.

## O229. Culture gap: antibiotic versus blood culture use for 61 facilities with NEST360 in Kenya, Malawi, Nigeria, and Tanzania

### Sarah Murless-Collins ^1^, Kondwani Kawaza^2^, Nahya Salim^3^, Chinyere Ezeaka^4^, William Macharia^5^, Msandeni Chiume^2^, Robert Tillya^6^, Opeyemi Odedere7, Martin Aluvaala^8^, Donat Shamba^6^, Georgia Jenkins^9^, Elizabeth Molyneux^2^, James Cross^1^, Joy Lawn^1^

#### ^1^London School of Hygiene & Tropical Medicine; ^2^Kamuzu University of Health Sciences; ^3^Muhimbili University of Health and Allied Sciences; ^4^University of Lagos; ^5^The Aga Khan University; ^6^Ifakara Health Institute; ^7^NEST360; ^8^University of Nairobi; ^9^RICE 360

*BMC Proceedings 2024*, **18(5):**O229

Submission ID #: IMNHC613

**Background**

Globally, 30 million small and sick newborns require admission annually, many being prescribed antibiotics without blood cultures, the current “gold standard” for neonatal infection detection. Low neonatal blood culture use hampers antibiotic stewardship, fueling antimicrobial resistance which threatens newborn survival. This study analyzes the gap between blood culture use and antibiotic prescribing for newborns admitted to facilities implementing with Newborn Essential Solutions and Technologies (NEST360) in Kenya, Malawi, Nigeria, and Tanzania.

**Methods**

This observational study design used prospective, individually-linked neonatal inpatient data and cross-sectional health facility assessment data. All newborns admitted to facilities with NEST360 were eligible for inclusion (July 2019–2022). We described facility-level antibiotic prescribing and blood culture use. Facilities were then categorized into three performance tiers based on assessment of laboratory and microbiology service availability, coupled with neonatal blood culture coverage (defined as blood cultures performed for newborns administered antibiotics).

**Results**

A total of 144,146 newborn records from 61 facilities were analyzed. Despite a mean facility rate of antibiotic prescription at 70.4% (facility range=25.1–100.0%), mean blood culture use was only 6.3% (facility range=0.0%–55.6%). Of the total 10,575 blood cultures performed, 10.4% (95%CI 9.8–11) were positive, but only 23.9% (23.1–24.8) had a documented result. Although all facilities had a laboratory, 24 (39.3%) performed no cultures for newborns. Tier 1 classified facilities were those that reported inability to perform blood culture (21/61). Most facilities (38/61) were classified as Tier 2, defined as sites reporting a microbiology service but <50% neonatal blood culture coverage. Only two facilities with microbiology had >50% culture coverage (Tier 3).

**Conclusions**

We found a major gap between antibiotic prescription and blood culture use for neonates in the four countries, but this could be closed rapidly. Positive facility outliers (Tier 3) highlight that higher blood culture coverage is possible but more research into local barriers and enablers is required. Tier 2 facilities are missing opportunities for infection detection, and quality improvement strategies by implementors and governments could increase coverage. Closing this culture gap and advancing locally-driven antimicrobial stewardship programs are priorities for reducing infection-related neonatal deaths and antimicrobial resistance.

## O230. Point-of-care quality improvement intervention able to reduce stillbirths: a case of Chancho primary Hospital, Ethiopia

### Aynalem Hailemichael Frew

#### Pathfinder International

*BMC Proceedings 2024*, **18(5):**O230

Submission ID #: IMNHC612

**Background**

Stillbirth is often defined as fetal death after 24 weeks of gestation. Most stillbirths occur in full-term pregnancies. Globally, between 1.7 and 2.5 million (median estimate 2.1 million) stillbirths occur every year, and most of them in developing countries. In Ethiopia, World Health Statistics 2013 revealed a stillbirth rate of 26 per 1,000 deliveries, which is third highest in the east African countries after Djibouti and Somalia (with stillbirth rates of 34 and 30 per 1,000 births, respectively) and seventh among the 10 countries that account for two-thirds of all third trimester stillbirths in the world. Although many countries have managed to reduce stillbirths, Sub-Saharan African countries still have high stillbirth rates. According to the Ethiopian Emergency Obstetric and Newborn Care Report (2016), nationally, the institutional stillbirth rate was 15 per 1,000 deliveries. In the baseline assessment of Chancho Primary Hospital in January 2020, the stillbirth rate was found to be 47 per 1,000 live births. After identification of the problem, the quality improvement (QI) team planned to improve the stillbirth rate in their hospital.

**Methods**

The QI team conducted self-assessment/clinical service audit. Based on findings, the QI team developed the following change ideas for testing starting January 2020: (1) strengthen danger sign counseling for pregnant women during ANC visit, (2) update on correct use of partograph during labor/delivery, (3) use regular chart/partograph review, and (4) use data for decision-making with the aim of improving the stillbirth.

**Results**

After stepwise testing of change ideas, with regular monitoring of the data, the team observed reduction in still birth from 47 per 1,000 live births to 9 per 1,000 live births. The run chart also showed significant reduction in stillbirth. Since then, the QI team has continued to implement the change ideas and monitor data regularly to maintain their gains.

**Conclusions**

The hospital QI team has managed to reduce stillbirth by applying change ideas during QI project implementation. We learned that strengthened danger sign counseling during ANC visit, updates on correct/consistent use of partograph, and regular chart/partograph review and technical support when needed are critical element to reduce stillbirth.

## O231. Effect of group antenatal care versus individualized antenatal care on birth preparedness and complication readiness: a cluster randomized controlled study among pregnant women in Eastern Region of Ghana

### Vida Ami Kukula^1^, Cheryl Moyer^2^, Veronica Esinam Awo Apetorgbor^1^, Elizabeth Awini^1^, Georgina Amankwah^1^, John Williams^1^, Nancy Lockhart^2^, Bidisha Ghosh^2^, Katherine James^2^, Ruth Zielinski^2^, Jody Lori^2^

#### ^1^Ghana Health Service; ^2^University of Michigan

*BMC Proceedings 2024*, **18(5):**O231

Submission ID #: IMNHC610

**Background**

As utilization of individualized antenatal care has increased throughout sub-Saharan Africa, prolonged waiting times, lack of caregiver continuity, and over-stretched staff have combined to limit its effectiveness in many settings. We implemented a trial of group-based antenatal care (G-ANC), an alternative service delivery model, to determine the impact on birth preparedness and complication readiness (BPCR) among pregnant women in Eastern Region, Ghana.

**Methods**

Using a cluster randomized controlled trial, we compared G-ANC to routine antenatal care among pregnant women in 14 health facilities between July 2019 to November 2021 in the Eastern Region of Ghana. We recruited women in their first trimester to participate in eight two-hour interactive group sessions throughout their pregnancies. Meetings were facilitated by midwives trained in G-ANC methods. Clinical assessments were conducted in addition to group discussions and activities around pregnancy-related topics. Data were collected at baseline, third trimester, and then at six weeks, six months and one year postpartum. Results are presented comparing baseline to third trimester for an 11-point additive scale of BPCR, as well as individual items comprising the scale.

**Results**

A total of 1,285 participants completed baseline and third trimester assessments (*N*=668 control, *N*= 617, intervention). Overall, BPCR improved for both the control group and the intervention group over time, however the increase was significantly greater in the intervention group. (Control: 1.4–1.7; Intervention: 1.4–2.9 (*p*< 0.0001). The elements of BPCR that were the most striking included arranging for emergency transport (which increased from 1.5% to 11.5% in the control group vs. 2.2% to 41.0% in the intervention group [*p*< 0.0001]) and saving money for transportation (which increased from 19.2% to 32.0% in the control group vs. 18.6% to 72.9% in the G-ANC intervention group [*p*< 0.0001]). Identifying someone to accompany the woman to the facility rose from 1.2% to 3.1% in the control group vs. 2.3% to 20.3% in the G-ANC intervention group (*p*< 0.001).

**Conclusions**

G-ANC significantly increased BPCR among women in rural Eastern Region of Ghana when compared to routine antenatal care. Given the success of this intervention, future efforts that prioritize the implementation of G-ANC are warranted

## O232. Validity of using a low-tech, handheld icterometer to screen for potential neonatal Jaundice

### Ashura Bakari^1^, Cheryl Moyer^2^, Benjamin Otoo^3^, Rexford Amoah^3^, Ann Wolski^4^

#### ^1^Ghana Health Service; ^2^University of Michigan; ^3^Suntreso Government Hospital; ^4^University of Cinncinnati

*BMC Proceedings 2024*, **18(5):**O232

Submission ID #: IMNHC606

**Background**

Neonatal jaundice (NNJ) remains a leading cause of newborn mortality in much of sub-Saharan Africa. NNJ is difficult to recognize without special, hospital-based equipment, particularly in dark-skinned infants. Previous work in developing hand-held, low-tech icterometers to assess jaundice have focused mostly on light-skinned infants or the tool’s diagnostic capability, i.e, whether it assesses the exact level of hyperbilirubinemia. We sought to examine the validity of using a hand-held icterometer in dark-skinned infants as a screening tool—that is, whether it is accurate in determining which children need further assessment.

**Methods**

We recruited 341 newborns aged 0–2 weeks who had not been managed for NNJ with phototherapy or exchange blood transfusion at a government hospital in Kumasi, Ghana. Caregivers watched a video demonstrating how to use a hand-held icterometer, after which they blanched the skin of the infant’s nose and compared it with the yellow shades numbered one to six on the icterometer. Each neonate was also assessed with a Drager JM-103 transcutaneous bilirubin meter. Two assessors trained in icterometer use (research assistants and health care workers) screened the same neonates, recorded their scores separately, and were blinded to each other’s reading.

**Results**

There was a significant correlation between values obtained via transcutaneous bilirubin meter, a standard screening tool for NNJ, and non-invasive icterometer scores given by caregivers (0.66), research assistants (0.79), and health care workers (*r*=0.77). A kappa coefficient of 0.63 indicated the agreement between caregivers and health care workers when using the hand-held icterometer. The kappa was 0.59 when comparing caregivers and research assistants using the icterometer. There was also a significant correlation (*p* value <0.001) between caregiver icterometer score and standard treatment recommendation as per the “Billi App,” which uses age-adjusted bilirubin thresholds to create treatment recommendations. (Paediatrics.co.uk)

**Conclusions**

A hand-held, low tech icterometer is an important potential mechanism for improving early jaundice identification. Further studies of the acceptability, feasibility, and cost-effectiveness of the icterometer’s use in home screening are warranted.

## O233. Zero separation. together for better care! Infant and family-centered developmental care in times of COVID-19: a global survey of parents’ experiences

### Johanna Kostenzer, Charlotte Von Rosenstiel-Pulver, Julia Hoffmann, Aisling Walsh, Luc J.I. Zimmermann, Silke Mader

#### European Foundation for the Care of Newborn Infants

*BMC Proceedings 2024*, **18(5):**O233

Submission ID #: IMNHC605

**Background**

The COVID-19 pandemic has created exceptional challenges, especially for the care of small and sick newborns. While most restrictions were necessary to stem virus transmission, some have impacted the provision and quality of health care, including infant and family-centred developmental care (IFCDC). This research explores parents’ experiences regarding the impact of the restrictions on key characteristics of IFCDC, including prenatal care, parental access, infant nutrition and breastfeeding, health communication, and mental health, during the first year of the pandemic.

**Methods**

Data was collected globally between August and November 2020. Parents of sick or preterm infants born during the first year of the pandemic and receiving special/intensive care were eligible for participation. Data analysis included descriptive statistics and statistical testing based on different levels of restrictive measures.

**Results**

2,103 participants from 56 countries were included in the study. Severe implications for the provision of IFCDC were identified. It was found that more than half of the respondents were not allowed to have another person present during birth. Percentages increased with the extent of COVID-19 related restrictions in the respondents’ country of residence (*p* = 0.002). Twenty-one percent of the participants indicated that no one was allowed to be present with the newborn receiving special/intensive care, with no opportunity to provide kangaroo mother care and skin-to-skin care. The more restrictive the COVID-19 related policy measures were, the more the respondents worried during pregnancy and after birth.

**Conclusions**

COVID-19 related restrictions severely challenged evidence-based cornerstones of IFCDC, such as separating parents and their newborns. There is an urgent need to reconsider separation policies, particularly in neonatal care. The application of an IFCDC approach must be strengthened worldwide to ensure that the 2030 Development Agenda is achieved. The findings must therefore be considered by public health experts and policymakers alike to reduce unnecessary suffering, calling for a zero separation policy.

## O234. Reducing missed opportunities for first Antenatal Care (ANC) visits through linkage of general Outpatient Department (OPD) to ANC services in Rural Rwanda

### Assumpta K Ayinamura Mwali^1^, Laban Bikorimana^2^

#### ^1^Intrahealth International; ^2^Mount Kenya University Rwanda

*BMC Proceedings 2024*, **18(5):**O234

Submission ID #: IMNHC604

**Background**

Timely referral from general outpatient department (OPD) and linkage to antenatal care (ANC) services has been found to be a promising strategy to reduce missed opportunities by improving first ANC attendance. This study aimed at exploring barriers and facilitators for linking pregnant women identified at OPD with ANC services.

**Methods**

The study used a mixed-methods approach and involved 797 pregnant women identified at OPD between January and March 2021 in six health centers in Rwamagana District in Rwanda. Demographic and clinical data were collected using the chart review data extraction technique. The association between early ANC visit and women’s demographic characteristics were assessed using the chi-square test. Using a cut-off of *p*-value = 0.2, we selected variables to include in a multivariate logistic regression model and tested variables that independently predicted early ANC visits. We conducted 18 interviews with health care providers and one focus group discussion with pregnant women to understand their perceptions on barriers and facilitators of OPD and ANC clinic linkage

**Results**

Of the 797 pregnant mothers identified in OPD, 72% (*n*=575) were referred to ANC services. Of those referred to ANC services, 44.52% (*n*=256) received ANC services the same day as their OPD visit, while the remaining 55.48% (319) received ANC services later. We found that 74% (*n*=428) of those referred to ANC services had an early ANC visit (attended within 12 weeks’ gestation). In a univariate analysis, early first ANC was associated with being legally married (OR=4.79, *p*=0.01) and being accompanied by a partner at ANC (OR=3.60, *P*=0.01). During interviews with clients and providers, the following issues were reported as significant barriers to successful linkage from OPD to first ANC visit: high workload due to understaffing in health centers, lack of health insurance, and unavailability of ANC services on certain weekdays.

**Conclusions**

OPD linkage to ANC services presents a unique opportunity for increasing uptake of ANC services in Rwanda. For this integration to work, efforts to improve early ANC visits should prioritize social behavior interventions that encourage partners to accompany pregnant women during ANC visits, ensure consistent availability of ANC services, and improve staffing levels at health centers.

## O235. Task shifting in the health workforce to improve quality and expand obstetric fistula care towards its elimination in Ethiopia: experience of Pathfinder International Ethiopia (PIE)

### Ketsela Desalegn, Bekele Belayihun Tefera

#### Pathfinder International

*BMC Proceedings 2024*, **18(5):**O235

Submission ID #: IMNHC588

**Background**

The Ministry of Health of Ethiopia is committed to intensifying efforts to increase access to treatment and prevention of new cases of obstetric fistula (OF) in order to eliminate obstetric fistula by 2025. This effort embarks on a two-pronged approach of prevention of new cases, and treatment of accrued survivors. However, the effort to decentralize and expand OF care faced an outstanding shortage of trained providers. To address this challenge, PIE and partners designed and implemented a competency-based training curriculum for mid-level providers (MLPs) on OF identification, diagnosis, and referral to treatment.

**Methods**

Task shifting is one of the strategies the Ministry of Health has relied on in its commitment to reduce maternal mortality and morbidity, including elimination of OF, since the era of the Millennium Development Goals. PIE supported the clinical skill training on OF to contribute to this task-shifting strategy. All sessions were hosted at Hamlin Centers, and senior OF consultants provided theoretical and clinical skills to the MLPs as per the curriculum. This survey covered the period: January 2017 to December 2021. Data for the survey was collected from DHIS2 and the project report.

**Results**

PIE supported the training of 1,048 MLPs from 901 facilities in four regions for six years. Trained MLPs on OF have since then received the role of senior providers to successfully screen, confirm, and refer OF survivors. Following this task-shifting, trained MLPs have identified a total of 3,110 new OF suspected cases, of whom 2,903 (93%) were confirmed, 2,848 (98%) of those confirmed got referred by the providers, and 2,508 (88%) of the referred received treatment.

**Conclusions**

Task-shifting for OF care to MLPs at the primary health care level from the conventional, centralized, and senior-providers-based care is a feasible strategy that expands access to service by addressing gaps in equity in Ethiopia. This task-shifting strategy can be further scaled-up in the public health system in support of elimination of OF in Ethiopia and can be tested in low- and middle-income countries as a feasible strategy.

## O236. Factors influencing delivery of the recommended service package for first antenatal care visit in Rwanda: a mixed-method study

### Assumpta K Ayinamura Mwali^1^, Egide Freddy Muragijimana^1^, Nicolas Karugahe^2^, Christian Mazimpaka^1^, Amedee Fidele Ndibaza^1^, Dieudonne Ndatimana^1^, Valentine Uwamahoro^1^, Donatille Mujawayezu^1^, Domina Asingizwe^3^

#### ^1^Intrahealth International; ^2^Zenysis; ^3^University of Rwanda

*BMC Proceedings 2024*, **18(5):**O236

Submission ID #: IMNHC586

**Background**

Rwanda has made great progress in improving access to antenatal care (ANC) services and neonatal mortality reduction. However, little is known about the quality of ANC services. This study assessed the completeness of delivering the recommended package of services for the first ANC visit and associated barriers and facilitators.

**Methods**

A facility-based, mixed-methods study was conducted from January to –March 2022. A two-stage cluster sampling was adopted; the first four districts were randomly selected. These are Gasabo, Rubavu, Rwamagana, and Ruhango, assuming one district per province. Next, two health centers with high ANC attendance were purposively selected from each district. A total of 2,242 records of ANC new registrant women were extracted from ANC registers from October to December 2021. We used the Rwandan Ministry of Health’s ANC package checklist to measure completeness. Provision of ANC package was reported using frequencies and percentages. Using thematic analysis, barriers and facilitators were reported from 12 interviews conducted among health care providers.

**Results**

Generally, 23.5% of women received more than 90% of the full ANC package, 75.6% received 50%–89% of the full package, and 0.89% received less than 50% of the full package. Most services that were provided during the first ANC included the provision of iron supplements (94.9%), the provision of tetanus vaccine (93.4%), and HIV testing (88.8%). Of the 2,242 pregnant women, 47.9% were not screened for malnutrition; 41.1% were not screened for anemia; 39.7% did not receive deworming tablets, and 39.6% were not screened for urinary tract infection. Most health care providers reported that stock-outs of reagents, health commodities not being reimbursed by health insurance, and high workload as barriers, while supervision and training were reported as facilitators to providing the recommended full ANC package.

**Conclusions**

Although Rwanda has improved ANC coverage over time, the provision of recommended ANC packages to women attending their first ANC is uneven. A lack of health commodities and high workload are the major barriers to providing the recommended package of ANC services to women attending their first visit. These shortcomings can be averted through proper financing, forecasting and quantification, and health workforce allocation.

## O237. Women’s lived experience during COVID-19 in India

### Aseema Mahunta Behra, Mercy Manoranjini, Tina Ravi, Aparajita Gogoi

#### Centre for Catalyzing Change

*BMC Proceedings 2024*, **18(5):**O237

Submission ID #: IMNHC581

**Background**

Systematic advocacy, based on women’s lived experiences, was needed to highlight how they were being affected by COVID-19 and the required measures to address the short-term and long-term implications of stigma and discrimination, violence against women, and the socio-economic impact on vulnerable populations, among other issues. To understand and act upon the ground-level realities of women of the reproductive age group (18–49 years), the Centre for Catalyzing Change (C3) conducted a survey across five Indian states of Assam, Madhya Pradesh, Rajasthan, Uttar Pradesh, and West Bengal in June 2020.

**Methods**

The individual interviews were administered through an android-based application in three Indian languages: Hindi, Assamese, and Bangla. The SRI-IRB committee certified the study protocol and tools. The total sample covered 11,154 women comprising pregnant women, women who delivered during COVID-19, and women in the 18–49 age group. The findings were from select community voices and cannot be generalized to the entire state population.

**Results**

Three-fourths of the women faced the family’s loss of livelihood, and 63% faced financial hardships. One in eight pregnant women did not receive even one ANC check-up. The reasons cited were lack of access to transportation (54%) and fear of COVID-19 infection (78%). One-third of the women (32%) said their child did not receive immunization services. Overall, 55% of women said they used family planning methods. The qualitative study findings indicated an increase in incidences of domestic and gender-based violence. Overall, 37% felt that such incidences were more than usual. Only one in three women was aware of the women’s helpline.

**Conclusions**

The findings were shared for action around critical areas for increasing knowledge and right-based access to reproductive, maternal, newborn, and child health services. The use of television, mobile phones, and social media can be explored for community engagement. New skills-building programs can help women and families with income generating opportunities that support improved agency, health and nutrition, and poverty alleviation. There is a need to create awareness among women to access prenatal care services regularly and also ensure linkages with Frontline Workers. Interventions should focus on increasing awareness of women’s helpline numbers, to report domestic and gender-based violence.

## O238. Which low- and middle-income countries have midwife-led birthing centres and what are the main characteristics of these centres? A scoping review and scoping survey

### Andrea Nove^1^, Oliva Bazirete^2^, Emily Callander^3^, Kirsty Hughes^1^, Vanessa Scarf^4^, Sabera Turkmani^5^, Mandy Forrester^6^, Bhagyashree Mandke^6^, Sally Pairman^6^, Caroline Homer^5^

#### ^1^Novametrics Ltd; ^2^University of Rwanda; ^3^Monash University; ^4^University of Technology Sydney; ^5^Burnet Institute; ^6^International Confederation of Midwives

*BMC Proceedings 2024*, **18(5):**O238

Submission ID #: IMNHC580

**Background**

Evidence about the benefits of midwife-led care during childbirth has led to midwife-led settings being recommended for women with uncomplicated pregnancies. However, most of the research on this topic comes from high-income countries. Relatively little is known about the availability and feasibility of midwife-led birthing centres (MLBCs) in low- and middle-income countries (LMICs). This study aimed to identify which LMICs have MLBCs, and to document their main characteristics. It is the first part of a project which will document what works and why for MLBCs in LMICs.

**Methods**

The study included a scoping review of peer-reviewed and gray literature, and a scoping survey of midwives’ associations. The review used nine databases and the Google search engine. We located 101 items published between 2012 and 2022. Information about each item was recorded and analyzed. The survey used a structured online questionnaire. Responses were received from 77 of the world’s 137 LMICs. The work was conducted in English, French, and Spanish.

**Results**

We found at least one piece of evidence of MLBCs from 57 of the 137 LMICs. The evidence was strong for 24 of them, i.e., we found evidence from at least two of the three types of sources (peer-reviewed literature, gray literature, and survey). Only 14 LMICs featured in the peer-reviewed literature. Low- and lower-middle-income countries were most likely to have MLBCs, but most of the peer-reviewed literature was from upper-middle-income countries. The most common type of MLBC was freestanding. Public-sector MLBCs were most common in middle-income countries. Some were staffed entirely by midwives and some by multidisciplinary teams. We identified challenges to the midwifery philosophy of care and to effective referral systems.

**Conclusions**

The peer-reviewed literature does not provide a comprehensive picture of MLBCs in LMICs. Many of our findings echo those from high-income countries and indicate that MLBCs could be an effective way to improve availability of skilled childbirth care in LMICs and reduce mortality and morbidity. Some findings are specific to some or all LMICs, with implications for scaling up this model of care in LMICs.

## O239. Fear as an underlying perception determines midwifery care provision in Southern Tanzania: a qualitative study using co-design

### Regine Unkels^1^, Andrea Pembe^2^, Helle Moelsted-Alvesson^1^, Effie Chipeta^3^

#### ^1^Karolinska Institutet; ^2^Muhimbili University of Health and Allied Sciences; ^3^Kamuzu University of Health Science

*BMC Proceedings 2024*, **18(5):**O239

Submission ID #: IMNHC566

**Background**

High-quality midwifery care plays a vital role in reducing maternal and perinatal mortality as well as increasing facility-based delivery rates. Midwifery care providers work in a complex and often unpredictable environment even more so in settings with high morbidity and mortality, low staffing levels, poor equipment, and frequent commodity stock-outs. The aim of this study was to understand how this environment is shaping care provision including the decision-making and communication in midwifery care in Southern Tanzania.

**Methods**

We used a qualitative study design which was part of the formative assessment for the “Action Leveraging Evidence to Reduce Perinatal Mortality and Morbidity in Sub-Saharan Africa” (ALERT) study. We conducted: (1) 16 interviews, (2) two focus group discussions with midwifery care providers; and (3) 48 hours of observations in maternity wards of two rural hospitals between February and June 2021. We applied reflexive thematic analysis based on the socio-ecological framework.

**Results**

Midwifery care providers were afraid of being unable to deal with situations that may culminate in a bad outcome. They feared to be blamed and held accountable for unfavorable outcomes, like perinatal death occurring during their shift irrespective of the circumstances. Mothers who did not abide by the tacit rules of the labor ward or behaved in an unpredictable way triggered this fear. This led to disrupted communication between midwifery care providers and mothers or their companions, often culminating in disrespectful care. Midwifery care providers felt unsupported and unprotected against social or legal reprimands in case of negative birthing outcomes.

**Conclusions**

Decision-making, service provision, and communication of midwifery care providers were often driven by fear of bad birthing outcomes. Providers and mothers then entered a spiral of increasing fear on both sides, with decreased cooperation and impaired communication which may contribute to abuse and even negative birthing outcomes. Policymakers and health care managers should acknowledge the influence of working in constrained environments on maternity workers’ capacity of decision-making, communication, and mental health.

## O240. Development of risk-prediction models for maternal and neonatal complications using machine learning across the continuum of care in a resource-constrained environment

### Gulnoza Usmanova, Anunaya Jain, Yashpal Jain, Erica Troncoso

#### Jhpiego

*BMC Proceedings 2024*, **18(5):**O240

Submission ID #: IMNHC562

**Background**

To improve maternal and neonatal outcomes, health systems must identify individuals at risk to make informed decisions about priority clinical interventions and resource allocation. Our objective was to design risk stratification algorithms across the continuum of care.

**Methods**

Data recorded during antenatal and intrapartum care from the state of Rajasthan, India was used. Patient records (60,336 for intrapartum and 5,805 for antenatal periods) were analyzed. Surrogate markers for adverse events were identified. The data was split to create training: testing datasets. Synthetic Minority Oversampling Technique (SMOTE) and Adaptive Synthetic Sampling Approach (ADASYN) was used to augment prevalence of predicted events. Pre-processing was used to impute values for missing variables.

Regression, classification (early classifier, non-myopic classifier) and deep-learning models were tried. Relative feature importance was evaluated to choose models with clinically appropriate features for final prediction.

**Results**

Models were created for maternal risk (postpartum hemorrhage [A] and prolonged labor[B]) and perinatal risk (stillbirth [C] and low birth weight [D]). Models C and D were created for antenatal risk stratification (ANC), whereas all four models were created for labor room admission (LR) risk stratification. The overall accuracy of models on the test dataset was:

The Sn, Sp, PPV, NPV and AC of the models were as below.

- A(LR) 60.5%, 86.8%, 48.3%, 91.5%, 77.1%
- B(LR) 93.5%, 88.6%, 3.8%, 100%, 88.6%
- C(ANC) 97.8%, 99.1%, 74.6%, 99.9%, 99.1%
- C(LR) 39.6%, 100%, 100%, 98.9%, 98.9%
- D(ANC-3 levels) -, -, -, -, 95.4%
- D(LR-3 levels) -, -, -, -, 95.9%

Using the risk scoring in ANC, the AUC (to predict adverse outcome) was 0.932 (0.903, 0.944, and 0.957 for trimester 1, 2, and 3, respectively), while for the LR admission the AUC (to predict adverse outcome) was 0.728 (0.696 for neonatal complications and 0.725 for maternal complications).

**Conclusions**

We provide proof of concept that machine learning algorithms and resultant risk scores can be potentially used to stratify pregnant women throughout the continuum of care. These need to be further validated. More comprehensive risk stratification models (including sepsis, eclampsia, neonatal asphyxia, and admission to neonatal intensive care unit) must also be created. However, risk scoring will not influence outcomes unless backed by care.

## O241. Big babies and mortality risk: how to identify neonatal vulnerability?

### Lorena Suarez-Idueta^1^, Joy Lawn^2^, Hannah Blencowe^2^, Eric Ohuma^2^, Ellen Bradley^2^, Yemi Okwaraji^2^, Judith Yargawa^2^, Joanne Katz^3^, Robert Black^3^, Daniel Erchick^3^, Elizabeth A. Hazel^3^, Michael Diaz^3^, Anne CC Lee^4^, Vulnerable Newborn Measurement Collaborative Group

#### ^1^Mexican Society of Public Health; ^2^London School of Hygiene & Tropical Medicine; ^3^Johns Hopkins Bloomberg School of Public Health; ^4^Harvard Medical School

*BMC Proceedings 2024*, **18(5):**O241

Submission ID #: IMNHC552

**Background**

Large for gestational age (LGA, >90^th^ centile) and macrosomia (≥4000g regardless of gestational age) have been associated with short-term complications including birth trauma, and long-term conditions such as overweight and obesity. To date, no worldwide systematic assessment of the proportion of big babies has been performed. We aimed to examine the prevalence and neonatal mortality risk of term (>37 weeks) LGA and macrosomic babies.

**Methods**

We identified 115.6 million nationwide records from 2000 to 2020, collected in 15 countries with high completeness (>80%) on birthweight, sex, and gestational age to assess size for gestational age (appropriate for gestational age [AGA], 10-90^th^ centiles or LGA) using INTERGROWTH-21^st^ standards. We excluded small-for-gestational-age or preterm livebirths. We calculated prevalence and relative risk (RR) of neonatal mortality among livebirths born at term + LGA vs term + AGA and macrosomic (≥4000g; ≥4500g; ≥5000g regardless of gestational age) vs 2500g-3999g.

**Results**

Almost a fifth of livebirths were term + LGA (median: 18.2%, Interquartile Range [IQR], 13.5, 22.0) and overall were associated with lower neonatal mortality risk compared to AGA (median RR, 0.75; IQR, 0.74, 0.82). Around one in ten babies were ≥4000g (median prevalence 9.6% (IQR 6.4, 13.3), with 1.2% (IQR 0.7, 2.0) ≥4500g and 0.2% (IQR 0.1, 0.2) ≥5000g. Overall macrosomia ≥4000g was not associated with increased neonatal mortality risk (median RR, 0.77; IQR, 0.67, 0.97), however a higher risk was observed for those ≥4500g (median RR, 1.10, IQR, 1.01, 2.44 ) and ≥5000g (median RR, 3.8, IQR, 1.6, 6.9), compared to those 2500g-3999g.

**Conclusions**

In this population, birthweight ≥4500g was the most useful marker for early mortality risk. Future research is needed to clarify the best categories to identify big newborns at the highest risk of dying, such as those on the right tail for size (>97th centile) or gestational age (post-term) distributions.

## O242. Journey to 9: transforming care through patient accompaniment and community engagement

### Marc Julmisse, Meredith Casella Jean-Baptiste, Monide Thamar Vital Julmiste

#### Partners in Health

*BMC Proceedings 2024*, **18(5):**O242

Submission ID #: IMNHC551

**Background**

Haiti has the highest maternal and neonatal mortality rates in the western hemisphere, at 480 per 100,000 and 25 per 1,000 live births respectively; according to the World Bank. In 2018,

Partners In Health and Zanmi Lasante developed Journey 9 (J9) to reduce maternal and neonatal morbidity and mortality in Haiti’s Central Plateau through the detection of early warning signs in pregnancy and the neonatal period. J9 uses interprofessional patient accompaniment and community engagement and awareness to holistically address the needs of mothers and infants by providing high-quality clinical care while also building trust in the health system.

**Methods**

J9 accompanies women and newborns through four pillars: g1) group prenatal care throughout pregnancy, and postpartum; (2) group pediatric care of newborns through the first year of life; (3) psychosocial support and counseling throughout all aspects of the program including for PTSD and depression screening prenatally and post-partum; and (4) community-based care via home visits.

**Results**

J9: improves facility-to-community connections through home visits; creates supportive communities through group antenatal care and group pediatric care; and improves facility-level care by improving patient knowledge, promotion of early identification of obstetrical complications, building clinical competency and addressing gaps in lifesaving solutions to ensure excellent antepartum, intrapartum, and postpartum care. J9 increases antenatal care and facility-based deliveries, reduces maternal complications, reduces patients wait times, and improves birth outcomes.

J9 drives rapid improvements in health outcomes: 97% of women in the J9 pilot delivered at a facility, compared to 35% nationally, with 95% adherence to eight antenatal care visits and 84% adherence to six pediatric well child visits. The program has impacted more than 2,000 women and newborns, with graduates from J9 becoming champions of the model.

**Conclusions**

J9’s innovation is through an integrated, community-based care and accompaniment approach to create bonds among women and with care providers while providing supportive, high quality care. It is unique for a maternal health program to focus on women’s experiences through support groups through pregnancy with home and health facility interactions. This new accompaniment approach leads to earlier identification of warning signs, durable behavior change, an improved ability to address multiple social determinants, and improved access to lifesaving intrapartum and neonatal care.

## O243. Quality of postabortion care in two African hospitals in fragile and conflict-affected settings: application of an adapted World Health Organization (WHO) Maternal and Newborn Health (MNH) Quality of Care Framework (AMoCo Study)

### Estelle Pasquier^1^, Onikepe Owolabi^2^, Richard Ngbale^3^, Mariette Claudia Adame Gbanzi^3^, Timothy Williams^1^, Daphne Lagrou^1^, Claire Fotheringham^1^, Tamara Fetters^4^, Catrin Schulte-Hillen^1^, Huiwu Chen^1^, Olivier Degomme^5^, Veronique Filippi^6^, Lenka Benova^7^, Bill Powell^4^

#### ^1^Medecins Sans Frontieres; ^2^IntraHealth International; ^3^Ministre de la Santé et de la Population, Central African Republic; ^4^Ipas; ^5^Ghent University; ^6^London School of Hygiene & Tropical Medicine; ^7^Institute of Tropical Medicine

*BMC Proceedings 2024*, **18(5):**O243

Submission ID #: IMNHC549

**Background**

Abortion-related complications remain a main cause of maternal mortality. Despite global guidance that post-abortion care (PAC) should be introduced as early as possible in an emergency, fragile, and conflict settings suffer from limited access to PAC, poor quality care, and lack of research on how to address these challenges. Using data from the Abortion-related Morbidity and mortality in fragile or Conflict-affected settings (AMoCo) study, we measured the quality of PAC in two hospitals supported by Médecins Sans Frontières (MSF) in Jigawa State (Nigeria) and Bangui (Central African Republic, CAR).

**Methods**

We analyzed data collected between November 2019 and July 2021 from a sample of 360 and 362 hospitalized women in Nigeria and CAR, respectively. We adapted the World Health Organization’s Maternal and Newborn Health Quality of Care framework to measure PAC process (provision of care according to guidelines and experience of care) and outcome (facility-based prevalence of abortion-near-miss happening more than 24h after presentation chlled “health-care-related abortion-near-miss”).

**Results**

Provision of care: in both hospitals, 98% of women were treated with an appropriate technology when receiving instrumental uterine evacuation. Over 80% received blood transfusion or curative antibiotics when indicated. However, antibiotics were given to about 30% of patients without indication. Although 97% of women in Nigeria received prophylactic antibiotics when indicated, only 44% did in CAR. Almost all (99%) of discharged women in CAR received contraceptive counseling but only 44% in Nigeria.

Experience of care: Over 80% of women in both hospitals reported that staff provided them good care, but only 49% in Nigeria and 59% in CAR said they were given explanations about their care and 15% felt capable of asking questions during treatment.

Outcome: The prevalence of health-care-related abortion-near-miss was 1.1% (95 CI: 0.3–2.8) in CAR and 2.8% (95 CI: 1.3–5.0) in Nigeria.

**Conclusions**

The two MSF-supported hospitals had good provision of care (when compared with hospitals in similar stable contexts), and high satisfaction expressed by women. This likely accounts for the low prevalence of health-care-realted abortion-near-miss. However, hospitals need to improve provider communication and would benefit from instituting antibiotic stewardships to prevent nosocomial infections and antibiotic resistance.

## O244. Integrating maternal near-miss case reviews with the pre-existing maternal death surveillance and response system: learning with three Malawian Hospitals

### Luis Gadama^1^, Monica Patricia Malata^1^, Jessie Khaki^1^, William Peno^2^, Effie Chipeta^1^, Alisa Jenny^3^, Dilys Walker^3^

#### ^1^Kamuzu University of Health Sciences; ^2^Queen Elizabeth Central Hospital; ^3^University of California, San Francisco

*BMC Proceedings 2024*, **18(5):**O244

Submission ID #: IMNHC537

**Background**

The World Health Organization recommends inclusion of maternal near-miss case reviews along with the Maternal Death Surveillance and Response System (MDSR) as quality improvement initiatives with the aim to improve availability of useful metrics, promote accountability, and improve reporting of severe maternal outcomes. This study evaluates the process of integrating maternal near-miss case reviews within the pre-existing MDSR system in three hospitals in Malawi.

**Methods**

The study used an implementation science research design piloted in three hospitals in Malawi. Maternal near-miss data were collected using adapted near-miss criteria for a period of nine months. The study also used Likert Scales and in-depth interviews to measure and assess provider perspectives on acceptability, appropriateness, feasibility, and sustainability of integrating maternal near-miss case reviews within the MDSR. The study participants were district health managers, service providers, and members of hospital MDSR committees.

**Results**

Across three health facilities, we recorded 12,253 live births, 236 maternal near-miss cases, and 63 maternal deaths representing a maternal near-miss ratio of 19 per 1,000 live births and a maternal mortality ratio of 514 per 100,000 live births. Obstetric hemorrhage was the leading cause of both maternal near-miss cases (51.7%) and maternal deaths (20.6%). Hypertensive disorders and obstetric infections were the second and third leading causes of both maternal near-misses and deaths. Obstetric related infections had the highest case fatality ratio (25%), followed by hypertensive disorders (19%), while the ratio for obstetric hemorrhage was 10.7%.

**Conclusions**

The study found including maternal near-miss reviews to be acceptable by service providers. Midwives welcomed the integration as it shifted the focus from the “blame game” nature of maternal death reviews to highlighting positive elements of care where lives were saved. However, participants reported that reviewing maternal near-miss cases increased their workload. In addition, they reported that time allotted to audit cases, draw up recommendations, and implement action points was dependent on the commitment of hospital managers. Nevertheless, participants were hopeful that the integration was sustainable.

## O245. Remodeling the government resource allocation to address inequities in Sexual, Reproductive, Maternal, Newborn, Child, and Adolescent Health and Nutrition (SRMNCAHN) in Zambia

### Namakando Mukelabai^1^, Rabson Zimba^1^, Olatubosun Akinola^1^, Hilda Shakwelele^1^, Samson Lungu^2^

#### ^1^Clinton Health Access Initiative; ^2^Ministry of Health

*BMC Proceedings 2024*, **18(5):**O245

Submission ID #: IMNHC531

**Background**

Resource allocation is the distribution of resources among competing groups of people or programs. Historically, the Ministry of Health in Zambia used the incremental budgeting method before shifting to a population-based resource allocation criteria. The population-based resource allocation criteria was later enhanced to include deprivation or poverty and discretionary proportional allocation based on demographic characteristics of the district. The current factors applied in the resource allocation are heavy on the demand side, with no or little focus on the supply side. The resource allocation criteria did not address the capacity of districts to provide SRMNCAHN services. The objective of this paper is to develop a hybrid resource allocation formula that incorporates needs and equity into the resource allocation formula with a view to allocating more resources to areas with higher capacity constraints to provide SRMNCAHN services.

**Methods**

In 2021, the peace health program of the government of the Republic of Zambia conducted a baseline survey to ascertain the needs of health facilities in Eastern and Southern Province of Zambia. Furthermore, the University of Zambia conducted an equity analysis in Eastern, Luapula, Muchinga, and Southern Provinces. Accordingly, the traditional resource allocation was adjusted to incorporate the needs and equity analysis results. Traditional weights were also adjusted to create a balance on the demand and supply sides of the resource allocation.

**Results**

The revision of the resource allocation resulted into some changes in allocations. Districts that originally received less funds were now getting more funds, thereby enabling better offer of SRMCAHN services.

**Conclusions**

The traditional resource allocation formula was biased towards population size and demand-side factors. This means that districts with more population but less needs would often receive more resources. The introduction of the needs and equity factors provides a balance between demand- and supply-side factors and results into equitable resource allocation. It is hereby recommended that the needs and equity analysis be scaled up countrywide and adjust the national resource allocation for government to include needs and equity analysis results. A needs and equity analysis would be required regularly to ensure equitable resource allocations over time.

## O246. Targeting postnatal care to mothers and babies most at risk: factors that increase the risk of poor outcomes in the postnatal period identified through a scoping review

### Preston Izulla^1^, Michael Kiragu^1^, Angela Muriuki^2^, Syeda Nabin Ara Nitu^3^, Joseph De Graft-Johnson^3^, Melanie Yahner^3^

#### ^1^Adroitz Consultants; ^2^Find Diagnostics; ^3^Save the Children

*BMC Proceedings 2024*, **18(5):**O246

Submission ID #: IMNHC530

**Background**

Maternal and newborn deaths in the early postnatal period can be averted with the provision of timely, quality, postnatal care (PNC), including postnatal home visits from community health workers (CHWs). Yet in many low- and middle-income countries (LMICs), coverage of early postnatal home visits (within the first 72 hours of life) has been hampered by under-resourced and over-burdened CHW cadres. A targeted PNC approach that prioritizes home visits to mother-baby dyads with identified risk factors could optimize limited resources. This study sought to determine the key risk factors that predict poor outcomes for mothers and newborns in LMICs during the postnatal period.

**Methods**

In 2021, a systematic review was conducted in PubMed, Scopus, CINAHL, and PsycINFO to determine: (1) the underlying risk factors for the major causes of maternal and neonatal mortality, and (2) the underlying risk factors for non-use of PNC services. Published, peer-reviewed studies conducted in LMICs within the last 10 years and that included a test of association between risk factor and outcome were included.

**Results**

Of the 6,200 citations identified, 894 full text studies were assessed and 60 included in the study. The risk factors identified were grouped into proximal and distal factors. Proximal factors include age (<19, >35), parity (primigravida), previous history (postpartum hemorrhage, pre-eclampsia), and marital status. Distal factors included household socioeconomic status, education, and place of residence (urban, rural). These risk factors informed the development of an algorithm that guides CHWs in Bangladesh to screen mother-baby dyads through phone calls after delivery, and prioritize home visits to those meeting clinical and non-clinical risk factors. Concurrently, facility-based providers screen mothers delivering in health facilities and delay discharge or provide referrals for those with identified risk factors.

**Conclusions**

In resource-constrained settings, an evidence-informed, targeted postnatal care approach can be used to pinpoint at-risk mother baby dyads for prioritization of early, postnatal home visits. Evidence-based risk criteria for both facility-based and community-based providers must be determined with consideration of both contextual relevance and feasibility of operationalization.

## O247. Effectiveness of safecare methodology in improving quality of maternal and newborn care: case study from Saving Mothers Giving Lives (SMGL) 2.0 project in Kaduna, Nigeria

### Ogochukwu Kehinde, Benjamin Asemota, Obafemi Omole, Paulina Akanet, Amina Aminu Dorayi

#### Pathfinder International

*BMC Proceedings 2024*, **18(5):**O247

Submission ID #: IMNHC511

**Background**

High quality of care is the bedrock of improved health outcomes. With a maternal mortality ratio of 452.6/100,000, Kaduna State’s neonatal mortality rate of 47/1,000 and low coverage (27%) of deliveries assisted by a skilled provider are worse than the national average. These outcomes are exacerbated by poor quality of care due to unskilled providers, lack of equipment, and inadequate basic amenities among other factors. SMGL 2.0, led by Pathfinder International in collaboration with PharmAccess Foundation and Kaduna State Government, is implementing a quality improvement (QI) program using the SafeCare methodology to ensure the delivery of quality-secured care for mothers and their newborns.

**Methods**

SafeCare is a stepwise QI program that enables facilities to measure and improve quality, safety, and efficiency of services. It is internationally accredited and based on the World Health Organization’s Quality of Care framework. SafeCare’s mobile application assesses facilities across four domains—management, clinical services, clinical support, and ancillary services—to identify quality gaps and prioritize areas for improvement.

SMGL’s QI program was implemented in 25 project-supported private facilities in Kaduna State. The facilities were assessed using SafeCare digital tools, which generated an automated report and QI plan for the facilities. Facility QI teams were trained in QI and onboarded into the interactive Quality Platform for Providers. The project provided onsite and virtual mentoring/supportive supervision to providers as well as capacity building, job aids, and quality guidelines. Regular review of QI results and feedback were used to monitor QI program implementation.

**Results**

After one year of implementation, maternal deaths decreased by 63%, newborns with birth asphyxia successfully resuscitated increased by 36%, partograph-monitored deliveries increased by 81%, and births in supported facilities increased by 17% when compared to baseline data.

**Conclusions**

The improvement in maternal and newborn health outcomes demonstrates that to improve quality of care, an innovative QI program like SafeCare, when used with focused mentoring and supportive supervision, can improve quality of care and health of mothers and newborns.

## O248. A blended learning approach to improve the quality of integrated HIV, TB, and Malaria services during antenatal and postnatal care in Low- and Middle-Income Countries (LMICs): a feasibility study

### Alice Ladur^1^, Florence Mgawadere^1^, Elizabeth Kumah^1^, Uzochukwu Egere^1^, Marion Ravit^1^, Christopher Murray^1^, Sarah White^1^, Hauwa Mohammed^1^, Rael Mutai^1^, Lucy Nyaga^1^, Leonard Katalambula^2^, Rukia Bakar^3^, Charles A Ameh^1^

#### ^1^Liverpool School of Tropical Medicine; ^2^University of Dodoma; ^3^The State University of Zanzibar

*BMC Proceedings 2024*, **18(5):**O248

Submission ID #: IMNHC510

**Background**

The blended learning (BL) approach to training health care professionals is increasingly adopted in many countries because of high costs and disruption to service delivery in the light of severe human resource shortage in low-resource settings. The COVID-19 pandemic increased the urgency to identify alternatives to traditional face-to-face (f2f) education approach. A four-day f2f antenatal care and postnatal care continuing professional development course was repackaged into a three-part BL course: (1) self-directed learning (16 hours); (2) facilitated virtual sessions (2.5 hours over two days); and (3) two-day f2f sessions. This pilot study assessed the feasibility and acceptability of implementing a BL course for quality improvement of integrated HIV, TB, and malaria services during antenaral and postnatal care.

**Methods**

A mixed-methods design was used. A total of 90 health care professionals were purposively selected. Quantitative data was collected through an online questionnaire and descriptive analysis carried out. Qualitative data was collected through key informant interviews and focus group discussions, analyzed using thematic analysis.

**Results**

The majority of participants (86%) accessed the course using a mobile phone from home and health facilities. The median (IQR) time for completing the self-directed component was 15 (7.5–24) hours. A multi-disciplinary team—comprising 40% nurse-midwives, 29% doctors, 19% clinical officers, and 12% other health care professionals—completed the BL course. Participants liked the BL approach due to its flexibility in learning, highly educative content, less time spent away from work, and link to continuing professional development points. Aspects that were noted as challenging were related to personal log-in details and network connectivity issues during the self-directed learning and virtual sessions, respectively.

**Conclusions**

The BL approach is flexible, feasible, and acceptable to health care providers. A study to compare the effectiveness of BL vs. the traditional f2f approach is needed.

## O249. Listen to us! A women-led campaign during the COVID-19 pandemic

### Smita Bajpai^1^, Aparajita Gogoi^2^, Tina Ravi^2^

#### ^1^CHETNA India; ^2^Centre for Catalyzing Change

*BMC Proceedings 2024*, **18(5):**O249

Submission ID #: IMNHC508

**Background**

The COVID-19 pandemic posed a challenge in access to maternal health services, despite being included in the list of essential services. The White Ribbon Alliance in Rajasthan, India, initiated a campaign, Hamari Awaz Suno, to advocate for quality and respectful maternal health services during April 11–20, 2021.

**Methods**

The campaign spanned across 99 villages from 21 blocks of 11 districts. The campaign strategy was women-led, in which women leaders collected demands from women and submitted them to government officials. Eleven members of civil society organizations were provided a virtual orientation by Centre for Health, Education, Training and Nutrition Awareness (CHETNA). A total of 165 women leaders, including elected representatives of the local self-government, were oriented to collect womens’ demands for quality care. They reached out to 695 women who delivered between April 2020 and March 2021. This information was then analyzed and a charter for demands was prepared. Dialogues were organized in hybrid mode and the charter of demands was submitted to government health officials. Media partnered with the initiative and published around 26 stories in 21 newspapers and online media with circulation ranging from 120,000 to 3,000. A national-level webinar was organized on May 28, 2021 in partnership with the White Ribbon Alliance India to share the experiences and wants of women. COVID-19 protocols were maintained as per government of India guidelines.

**Results**

The campaign has resulted in action by the health officials to fulfill the demands made by women in 10 blocks of the state and has provided women leaders an opportunity to interact with the government health officials.

**Conclusions**

Maternal health services are a part of essential health services and need to be delivered even during difficult situations. Listening to what women want enables the health system to ensure delivery of quality maternal health services and needs to be integrated in the design and implementation of health programs worldwide.

## O250. Educational Interventions targeting pregnant women to optimize the use of cesarean section: what are the essential elements? A qualitative comparative analysis

### Rana Islamiah Zahroh^1^, Katy Sutcliffe^2^, Dylan Kneale^2^, Martha Vazquez Corona^1^, Ana Pilar Betran^3^, Newton Opiyo^3^, Caroline Homer^4^, Meghan Bohren^1^

#### ^1^The University of Melbourne; ^2^University of College London; ^3^World Health Organization; ^4^Burnet Institute

*BMC Proceedings 2024*, **18(5):**O250

Submission ID #: IMNHC507

Note the full manuscript published in September 2023 in *BMC Public Health*:

(https://bmcpublichealth.biomedcentral.com/articles/10.1186/s12889-023-16718-0#:~:text=We%20identified%20four%20key%20essential,to%20interact%20with%20health%20providers.)

## O251. Interventions targeting health care providers to optimize the use of cesarean section: a qualitative comparative analysis to identify important intervention features

### Rana Islamiah Zahroh^1^, Katy Sutcliffe^2^, Dylan Kneale^2^, Martha Vazquez Corona^1^, Ana Pilar Betran^3^, Newton Opiyo^3^, Caroline Homer^4^, Meghan Bohren^1^

#### ^1^The University of Melbourne; ^2^University of College London; ^3^World Health Organization; ^4^Burnet Institute

*BMC Proceedings 2024*, **18(5):**O251

Submission ID #: IMNHC506

Note the full manuscript published in December 2022 in *BMC Health Services Research*:

(https://bmchealthservres.biomedcentral.com/articles/10.1186/s12913-022-08783-9)

## O252. Use of digital technology for real-time reporting of maternal deaths: development of a Maternal and Perinatal Death Surveillance and Response (MPDSR) tool - an experience from Pakistan

### Sabeen Afzal^1^, Afifa-binte Irfan^1^, Qudsia Uzma^2^, Ellen Mpangananji Thom^2^

#### ^1^Ministry of National Health Services Regulations and Coordination, Pakistan; ^2^World Health Organization

*BMC Proceedings 2024*, **18(5):**O252

Submission ID #: IMNHC501

**Background**

Maternal mortality is considered a key indicator of a country’s health status and its socio-economic development. Over the decades, Pakistan has managed to reduce the maternal mortality ratio from 276/100,000 (2006) to 186/100,000 (2018). However, subnational data shows variation being 140/100,000 in Punjab and 298/100,000 in Balochistan. Unfortunately, maternal mortality data is only available through population-based surveys and there is a dire need to have real-time data reporting with identification of the cause of mortality to guide programmatic decision making. The MPDSR system offers a workable solution and is being implemented in Pakistan since 2015.

**Methods**

Under the MPDSR system implementation, national protocols and training package were developed in 2017. Maternal death reporting forms 1, 2, and 4 were adapted in Pakistan for reporting of maternal deaths at facility level. A mobile application was also developed for real-time and paperless reporting. Training of hospital teams including obstetricians, nurses, administrators, anesthetists, pediatricians and statisticians were conducted on the manual as well as on the mobile app (web-based tool) involving sixteen (16)public sector hospitals mainly tertiary care teaching hospitals. This tool was then revised and customized by the national TWG on Reproductive, Maternal, Newborn, Child and Adolescent Health per professional feedback. Data is now being regularly received from the participating hospitals and uploaded to the MPDSR dashboard. Recently, the form on perinatal deaths including stillbirths and a response tracking sheet has also been added.

**Results**

With the introduction of the MPDSR mobile app tool, the data collected under MPDSR system including notification of deaths, status of the deceased at time of admission, status at the time of death (antenatal, natal, and postnatal), antenatal history, examination record, laboratory investigations, status of the fetus/neonate (gestational age, outcome of birth, vaccination, breastfeeding, resuscitation) and any interventions during antepartum, intrapartum, and postpartum. . The case summary along with challenges faced in the provision of care to the patient are also recorded. On the basis of this information, the maternal death review committees undertake the in-depth review, assign the cause of death and prepare response/ action plan for addressing the contributory factors. All of this information is available online through the dashboard linked to the mobile app. Since the introduction of the mobile app, the number of maternal deaths reported during 2021, 2022 and June 2023 are 87, 521 and 367, respectively. Postpartum hemorrhage was the most reported cause.

**Conclusions**

Digital technology can be used for informed decision-making and improvement in quality of care through provision of real-time data.

## O253. An example of too much too soon? A review of cesarean sections performed in the first stage of labor in Kenya

### Helen Allott^1^, Fiona Dickinson^1^, Nassir Shaban^2^, Sheila Sawe^3^, Evans Ogoti^3^, Michael Odour^4^, Stephen Karangau^5^, Ephraim Ochola^6^, Charles A Ameh^1^

#### ^1^Liverpool School of Tropical Medicine; ^2^Msambweni County Referral Hospital; ^3^Moi University; ^4^Bondo Subcounty Hospital; ^5^Muriranjas Subcounty Hospital; ^6^Homa Bay Subcounty Hospital

*BMC Proceedings 2024*, **18(5):**O253

Submission ID #: IMNHC500

**Background**

Cesarean section (CS) is the most commonly performed major surgical procedure, with global rates rising. When performed for appropriate indications, CS can be lifesaving for mothers and babies. However, the procedure has potential short- and long-term complications, with reported rates of CS-associated death in sub-Saharan Africa at 10.9 per 1,000 procedures. In many health facilities, decisions to perform CS are made by non-specialist doctors, without support from experienced obstetricians. This can result in sub-optimal decision-making and inappropriate surgery. Our study assesses decision-making in CS performed in the first stage of labor.

**Methods**

Data extracted from 87 case notes, randomly selected from a series obtained from seven referral hospitals in five Kenyan counties over six months in 2020, was reviewed by a panel of seven UK and Kenyan expert obstetricians. Following a preliminary review of the data and an email discussion, an online panel was convened to discuss outstanding cases.

**Results**

There was consensus on all but five cases. In 41.3% cases, CS was considered appropriate by all assessors. The panel concluded that the CS was necessary but performed too late in 8% cases. The decision to delivery interval was longer than two hours in 58.6% cases, including 16 cases where the main reason given for the CS was non-reassuring fetal status. In 10.3% cases, it was considered that due to the decision to delivery delay, further reassessment should have occurred. In 9.1%, it was considered that the CS was done too soon and those providing care could have waited longer or attempted augmentation of labor. The quality of some records resulted in insufficient information to make a full assessment in 21.8% of cases and in 11.5% the CS was considered inappropriate.

In cases where agreement was not reached, meconium stained liquor was the stated reason for CS. Some panel members considered closer monitoring of the fetal heart was indicated, whereas others felt this was unlikely to be feasible due to staffing constraints.

**Conclusions**

This review demonstrates a need for improved support for decision-making, coupled with improved record-keeping and more timely surgery when necessary.

## P254. Factors influencing appropriate use of interventions for management of women experiencing preterm birth: a mixed-methods systematic review and narrative synthesis

### Rana Islamiah Zahroh^1^, Alya Hazfiarini^1^, Katherine Eddy^2^, Joshua Vogel^2^, Özge Tunçalp^3^, Nicole Minckas^3^, Fernando Althabe^3^, Olufemi Oladapo^3^, Meghan Bohren^1^

#### ^1^The University of Melbourne; ^2^Burnet Institute; ^3^World Health Organization

*BMC Proceedings 2024*, **18(5):**P254

Submission ID #: IMNHC499

Note the full manuscript published in August 2023 in *PLOS Medicine:*

(https://journals.plos.org/plosmedicine/article?id=10.1371%2Fjournal.pmed.1004074)

## O255. Interventions co-designed by providers and clients for improving their therapeutic relationships in maternal and child health care in rural Tanzania

### Kahabi Isangula, Eunice Pallangyo, Eunice Ndirangu-Mugo

#### The Aga Khan University

*BMC Proceedings 2024*, **18(5):**O255

Submission ID #: IMNHC497

Note the full manuscript published in September 2023 in *BMC Nursing:*

(https://bmcnurs.biomedcentral.com/articles/10.1186/s12912-023-01472-w)

## O256. Strengthening post-discharge follow-up for small and sick newborns in Bangladesh

### Munia Islam^1^, Zubair Shams^1^, Bal Ram Bhui^1^, Mohammad Morshad Alam^2^, Muhammad Ismail Hasan^2^, Afsana Karim^1^, Mohammod Shahidullah^3^, Joseph De Graft-Johnson^1^

#### ^1^Save the Children; ^2^Ministry of Health & Family Welfare, Bangladesh; ^3^Bangabandhu Sheikh Mujib Medical University

*BMC Proceedings 2024*, **18(5):**O256

Submission ID #: IMNHC492

**Background**

In Bangladesh, more than two-thirds of neonates die within seven days of birth, and half of them die within 24 hours of birth due to infections, birth asphyxia, prematurity, and low birth weight. USAID’s MaMoni MNCS project assists the Ministry of Health and Family Welfare in scaling up kangaroo mother care (KMC) and special care newborn unit (SCANU) services for small and sick newborns in public facilities across the country to end preventable newborn deaths. Post-discharge follow-up is a known challenge in the care of these babies. The project implemented and tested a combined post-discharge phone call and community follow-up mechanism for KMC and SCANU babies in selected public facilities.

**Methods**

A mixed-method study was conducted in one district hospital and one upazila health complex to examine the effect of the post-discharge strategy. Data was extracted from the national health management information system, facility registers, KMC community follow-up, and phone follow-up record books for the baseline (October 2018–September 2019), and for the post-intervention period (January 2020–July 2021). Twenty-seven key informant interviews with hospital providers, six focus group discussions with community health workers, and seven group interviews with mothers/family members of KMC/SCANU babies were conducted.

**Results**

The results show that 48% of discharged SCANU babies (post-intervention) received a first facility follow-up, compared to 0% at baseline. First post-discharge facility follow-up visits for discharged KMC babies increased from 47% to 76% in the district hospital and from 20% to 78% in the upazila health complex. Furthermore, 52% of discharged KMC babies from the district hospital and 89% from the upazila health complex received their first community follow-up visits. Mothers/family members and providers expressed that phone follow-up and counseling during discharge by nurses increased facility follow-up for both SCANU and KMC babies. Community follow-up by community health workers also prompted the families to complete KMC follow-up on time.

**Conclusions**

The combined phone and community follow-up strategy improved facility post-discharge follow-up for SCANU and KMC babies. The Ministry of Health and Family Welfare should consider adopting this approach to ensure optimal care and survival of sick newborns.

## O257. Influence of expectations and intentions during pregnancy on postnatal care utilization among adolescent and young first-time mothers: lessons from a qualitative study in Bangladesh

### Bidhan Krishna Sarker^1^, Sarah Elaraby^2^, Syeda Nabin Ara Nitu^3^, Musfikur Rahman^1^, Tasnia Ishaque^1^, Victoria Lwesha^3^

#### ^1^International Centre for Diarrhoeal Disease Research, Bangladesh; ^2^University of Alexandria; ^3^Save the Children

*BMC Proceedings 2024*, **18(5):**O257

Submission ID #: IMNHC489

**Background**

In Bangladesh, 43% of women have started childbearing before turning 18. Despite high antenatal care coverage, less than a third of adolescent and young mothers receive postnatal care (PNC) services. We explored the intention to seek PNC for 15–24 years-old first-time mothers (FTMs), their expectations of postnatal services, and how expectations influence service utilization.

**Methods**

Using a longitudinal qualitative exploratory design in 2022 in Noakhali, Bangladesh, we conducted two in-depth interviews with 22 FTMs, during the third trimester and then within 42 days postpartum. Within and cross-case analyses were conducted to illustrate the influence of expectation on PNC utilization among those who sought care and those who did not.

**Results**

While the majority of FTMs interviewed sought multiple antenatal care visits, most did not recall receiving any counseling or information regarding PNC services. During pregnancy, most participants had no specific expectations of PNC due to lack of awareness of their existence and no previous birth experience. Almost all participants either did not consider seeking services postnatally, or only intended to seek PNC if there is a problem with mother or infant’s health. Some FTMs were able to cite some specific complications that would motivate them to seek PNC, including excessive bleeding or pain. A key theme was lack of decision-making power by FTMs on where to deliver their child and whether to seek care, where the decision lay with the spouses or in-laws. Intentions during pregnancy directly influenced FTM’s care-seeking behavior. Most FTMs receiving PNC solely sought it to address issues with their child’s health. In a few instances, negative experiences during a facility delivery dissuaded FTMs from seeking additional PNC. Others wanted to seek PNC but did not know where to receive services or were prevented by their husband’s families.

**Conclusions**

Increasing PNC utilization among FTMs is crucial to improve maternal and child outcomes. Improved antenatal care counseling quality regarding PNC availability and importance impacts expectations, intention, and utilization of services. Family engagement, particularly with husbands and in-laws, is essential to improve PNC coverage as they influence maternal decision-making.

## O258. Maternal depressive symptoms increase risk for pregnancy loss, preterm birth, and low birthweight among Kenyan mother-infant pairs: a prospective cohort study

### John Kinuthia^1^, Anna Larsen^2^, Jillian Pintye^2^, Felix Abuna^3^, Amritha Bhat^2^, Julia Dettinger^2^, Lauren Gomez^2^, Mary Marwa^3^, Nancy Ngumbau^1^, Ben Odhiambo^3^, Amanda Phipps^2^, Barbra Richardson^2^, Salphine Watoyi^3^, Joshua Stern^2^, Grace John-Stewart^2^

#### ^1^Kenyatta National Hospital; ^2^University of Washington; ^3^University of Nairobi

*BMC Proceedings 2024*, **18(5):**O258

Submission ID #: IMNHC479

Note the full manuscript published in April 2023 in *Paediatr. Perinat. Epidemiol.:*

(https://onlinelibrary.wiley.com/doi/10.1111/ppe.12978)

## O259. Programmatically feasible gestational age assessment of newborns in Low- and Middle-Income Countries (LMICs)

### Sachiyo Yoshida^1^, Rajiv Bahl^1^, Anne CC Lee^2^

#### ^1^World Health Organization; ^2^Harvard Medical School

*BMC Proceedings 2024*, **18(5):**O259

Submission ID #: IMNHC474

**Background**

Approximately 36% of neonatal deaths are due to preterm birth complications. An accurate assessment of gestational age (GA) is critical to identify preterm birth and provide adequate care. However, in many LMICs, GA dating is often inaccurate or unknown, leaving the estimation of the GA after the baby is born. In this study, we developed programmatically feasible approaches to estimate newborn GA in low-resource settings.

**Methods**

Pregnant women less than 20 weeks gestation by ultrasound dating were enrolled from population-based cohorts in five countries (Bangladesh, Ghana, Pakistan, Tanzania, and Zambia) under the World Health Organization’s Alliance for Maternal and Newborn Health Improvement (AMANHI) study. Newborn examinations were conducted for neuromuscular and physical signs, anthropometry, and feeding maturity. Ensemble models were constructed using machine-learning techniques and areas under the receiver operating curve (AUC), and Bland-Altman analysis was performed to assess diagnostic accuracy.

**Results**

We enrolled 7,428 liveborn infants (of which 7.2% were preterm). A model including 10 newborn characteristics (birth weight, head circumference, chest circumference, foot length, breast bud diameter, breast development, plantar creases, skin texture, ankle dorsiflexion, and infant sex) estimated GA with no bias, 95% LOA ±17.3 days and an AUC=0.88 for classifying the preterm infant. A model that included last menstrual period (LMP) with the 10 characteristics had 95% LOA ±15.7 days and high diagnostic accuracy (AUC=0.91). An alternative, simpler model including birth weight and LMP had 95% LOA of ±16.7 and an AUC of 0.88.

**Conclusions**

A model, including 10 neonatal characteristics and LMP, estimated GA within ±15.7 days of early ultrasound dating and was able to classify preterm infants 91% of the time. Other simpler two-characteristic models also performed well and can be used as potential alternatives to identify preterm infants. In moving forward, with funding support from the Bill & Melinda Gates Foundation, we plan to translate these research findings to innovation, a simple, easy-to-use software application that uses machine-learning algorithms to estimate accurate newborn GA in both clinical and research use with limited resources.

## O260. Improving quality and access to safe cesarean section: Comprehensive Emergency Obstetric and Newborn Care (CEmONC) mentorship and onsite simulation-based training in developing regional states of Ethiopia

### Rahel Demissew Gebreyohannes^1^, Mekdes Daba Feyissa^1^, Alemnesh Tekleberhan Reta^2^

#### ^1^Ethiopian Society of Obstetricians and Gynecologists; ^2^Laerdal Global Health

*BMC Proceedings 2024*, **18(5):**O260

Submission ID #: IMNHC469

**Background**

The World Health Organization estimates that around 295,000 women die due to complications of pregnancy and childbirth. Cesarean section (CS) is an essential part of comprehensive emergency obstetric and newborn care (CEmONC) and a lifesaving intervention for women and newborns when it is done safely, timely, and with absolute indication. Access to safe CS is still sub-optimal in low- and middle-income countries including Ethiopia. Nonavailability of competent providers to provide safe CS is a major barrier. The Ethiopian Society of Obstetricians and Gynecologists (ESOG) collaborated with partners and the Ministry of Health to improve CEmONC services in selected hospitals.

**Methods**

The project period was between September 2019 and December 2020, and between March 2021 and May 2022. A baseline assessment followed by clinical mentorship was implemented in 11 primary and general hospitals located in remote and hard-to-reach areas of the country. The mentorship program encompassed simulation-based training using the MamaBirthie CS simulator, one-on-one case management, case scenario discussion, and telephone follow-up. In addition, a practical faculty guide for performing safe CS and managing intra-operative complications was prepared to advance training using MamaBirthie CS simulator. Advanced in-service and pre-service trainings using the training guide and the simulator were provided.

**Results**

The projects improved teamwork, communication, knowledge, skill, and confidence of providers with observed decreased perinatal mortality in the project hospitals. It also increased the institutionalized use of the World Health Organization’s safe surgery checklist and surgical outcome. CEmONC signal functions increased from 60% at baseline to 90%. The faculty guide helped to standardize simulation-based training and improved skills, competency, and communication between mentors and mentees. In Tanzania, the faculty guide was used for simulation-based approaches to CS training and was approved for accreditation by the medical council of Tanzania.

**Conclusions**

Onsite mentorship improves teamwork and interpersonal communication, which results in improved quality of care and patient outcome. The use of simulation-based training is very important in skills development and decision-making. The MamaBirthie CS simulator provides an opportunity to demonstrate and practice skills and engage in teamwork, communication, and decision-making in obstetric management.

## O261. A Multidisciplinary team-based approach to strengthen the quality and safety of cesarean sections in Makueni County, Kenya

### Daisy Ruto, John Varallo, Mary Muthengi, Freda Nyaga

#### Jhpiego

*BMC Proceedings 2024*, **18(5):**O261

Submission ID #: IMNHC463

**Background**

Women in African countries continue to suffer high rates of complications and death from unsafe cesarean section (CS) due to suboptimal technical and non-technical skills. A multidisciplinary team-based approach is an essential, effective, and patient-centered service delivery approach that promotes positive health outcomes.

The Johnson & Johnson-funded Obstetric Safe Surgery (OSS) project in Makueni County, Kenya, aimed to overcome barriers to safe, timely, and quality CS by using a multidisciplinary team-based approach tailored to the local context. The interventions included improvement of non-technical skills of surgical teams (e.g., to improve teamwork and communication) along with standardizing clinical care and creating a patient safety culture.

**Methods**

The OSS intervention was implemented in five health facilities in Makueni County using a hub-and-spoke model. Multidisciplinary surgical teams (consisting of five members each, representing surgery, anesthesia, and nursing) were trained using simulation and live cases. Training and ongoing mentoring focused on both the technical (e.g., infection prevention bundles, CS standardization, and essential newborn care at CS) and non-technical skills, along with patient safety processes, such as the World Health Organization’s Surgical Safety Checklist (SSC). Data was collected from March 2021 to April 2022 using REDCap from analysis of existing database and service statistics.

**Results**

Eighty-eight health care providers from five facilities were trained and mentored using a multidisciplinary, team-based, hands-on approach to introduce and implement the OSS interventions.

During 14 months of the project implementation, 4,051 CS were conducted. The SSC adherence rate increased from a baseline of 0% to being used in nearly every CS (96.6%), while surgical site infection rates decreased from 3.5% to 1.1%, and maternal perioperative mortality rates declined (0.4% to 0.35%). Immediate newborn care practices at CS markedly improved from baseline: delayed cord clamping increased from 0.0% to 81.9%, immediate skin-to-skin from 0.0% to 88.1%, and breastfeeding within one hour of birth improved from 18.1% to 83.6%.

**Conclusions**

Our tailored, multidisciplinary, team-based approach helped breakdown technical silos and hierarchies, fostering a much stronger sense of team within and across facilities, as well as improved ownership and use of data. This led to improved team performance, patient safety, and overall quality of CS care.

## O262. Group antenatal care implementation: providing technical assistance for innovative and sustainable maternal and child health service delivery in a Northern Nigeria State

### Abiola Ajibola^1^, Francis Ogirima^1^, Abimbola Phillips^1^, Collins Imarhiagbe^1^, Ibidun Jolaoso^1^, Saude Sagir^1^, Victor Obagunlu^1^, Pius Christopher Izere^1^, Lilian Anomnachi^2^, Layi Jaiyeola^2^, Adetosoye Adebanjo^2^, Damilola Olaniyan^2^, Neyu Iliyasu^3^, Sunday Joseph^4^, Bolanle Oyeledun^1^

#### ^1^Center for Integrated Health Programs; ^2^Technical Advice Connect; ^3^State Primary Health Care Board, Nigeria; ^4^State Ministry of Health, Nigeria

*BMC Proceedings 2024*, **18(5):**O262

Submission ID #: IMNHC451

**Background**

Nigeria has a low antenatal care (ANC) coverage rate, leading to poor uptake of critical lifesaving maternal, newborn, and child health (MNCH) interventions. While progress has been around antenatal care (ANC) visits, gaps remain in: quality of care, completion of ANC with the recommended eight visits, uptake of delivery by skilled birth attendants, poor uptake of intermittent preventive treatment of malaria during pregnancy (IPTp) and postpartum contraceptives. Group antenatal care (G-ANC) has the potential to improve uptake of MNCH services in low-income countries but has been scarcely implemented in Nigeria. This study describes the implementation and outputs of the G-ANC model in Kaduna State, one of the pioneered states that scaled up G-ANC after a successful randomized control trial in Nigeria and Kenya.

**Methods**

G-ANC implementation commenced in January 2020 in 485 primary health care facilities with technical assistance from Centre for Integrated Health Programs. Following multilevel stakeholders’ engagements, health facilities were selected based on predetermined criteria. A total of 1,428 health care workers were trained to facilitate cohort sessions using the low-dose, high-frequency capacity building approach. Pregnant women were grouped in 5-20 per cohort according to their gestational age (GA). A take-home picture booklet with key messages related to pregnancy and birth experiences family planning, and postnatal care was developed for discussions during cohort meetings at the facility and for home use.

**Results**

A total of 26,769 G-ANC sessions were held between January 2021 and April 2022 for 24,385 cohorts of pregnant women. Post-intervention, there was substantial increase in uptake of MNCH services compared to baseline including: uptake of four doses or more of IPTp (260%), first ANC visit before 20 weeks GA (166%), acceptance of postpartum contraceptives (148%), and fourth ANC visit (80%). The pictorial booklet encouraged the interests of husbands in their spouses’ pregnancy experiences.

**Conclusions**

Implementation of the G-ANC model is feasible in Northern Nigeria. It provides an innovative and sustainable approach to improving MNCH outcomes, and increases active male partner participation in maternal health. Scale-up of this approach should be extended to other states and countries.

## P263. Examining the emergency surgical obstetrics services and factors related to maternal health outcomes in india: an analysis of national family health survey 2019–2021

### Ajay Khera, Akash Porwal

#### EngenderHealth

*BMC Proceedings 2024*, **18(5):**P263

Submission ID #: IMNHC449

**Background**

Globally, around 800 women die from pregnancy- or childbirth-related complications every day despite a continuous decrease in the maternal mortality ratio. India has witnessed a significant increase in cesarean section (CS) rates and crossed the World Health Organization threshold of 15%. When medically indicated, CS can effectively prevent maternal and perinatal mortality and morbidity. However, the risks and costs associated with CS are significant, particularly where there is no medical indication for the procedure. This study examines CS rates in India, with a focus on differentials and determinants.

**Methods**

The study uses data from the National Family Health Survey between 2019–2021. The analysis was conducted on data from 230,870 mothers who had live births during the five years preceding the survey. Descriptive statistics as well as the results of bivariate analysis and binary logistic regression models with 95% confidence intervals are presented.

**Results**

Twenty-two percent of live births were delivered through CS with a significant state-level differential (5% in Nagaland to 60% in Telangana). Of these, 9% of CS were decided on after the onset of labor pain, compared to 13% that were decided on before the onset of labor pain (4.4% in Meghalaya compared to 38% in Telangana). Out of the total CS deliveries, 48% were done in private health facilities compared to 14% in public health facilities. CS rates were higher for the first birth (28%; odds ratio [OR] = 1.5), births in urban areas (32%; OR = 2.3), among mothers with more years of formal schooling (27%; OR = 1.9), and among those at high wealth strata (39%); OR = 2.2).

**Conclusions**

The CS rate is high at the national level with variations across states and districts. With a wide public-private sector differential, it is imperative to devise an audit system of CS deliveries happening across the country. Tailored policies and regulations are needed to rationalize both elective and non-elective CS deliveries across the public and private sectors. Further research is needed to understand the factors underlying the high CS rates in private health facilities and to document the maternal and perinatal outcomes associated with CS, particularly non-indicated CS in India.

## O264. Post-discharge tracking of surgical site infections among cesarean section clients in Makueni County, Kenya

### Mary Muthengi, John Varallo, Freda Nyaga

#### Jhpiego

*BMC Proceedings 2024*, **18(5):**O264

Submission ID #: IMNHC430

**Background**

Most surgical site infections (SSIs) after cesarean section (CS) occur following discharge of the mother from the hospital. In Kenya, poor follow-up of the client after she leaves the facility, lack of self-care awareness of post-CS women, and delayed care-seeking are major gaps in improving detection and timely management of SSIs, as well as in providing accurate estimation of SSI rates. The Johnson & Johnson-funded Obstetric Safe Surgery (OSS) project in Makueni County, Kenya, aimed to strengthen community-level follow-up of mothers and newborns at Day 7 and Day 30 post CS.

**Methods**

The OSS program was implemented in five health facilities in Makueni County. Improving prevention and management of SSIs included active post-discharge tracking of clients coupled with standardized SSI management. Prior to discharge from the facility, clients were educated on post-CS self-care and warning signs. Community health volunteers reinforced the messages during household visits. Guided by standardized interview scripts, nurses conducted post-CS follow-up via telephone calls at Day 7 and Day 30 post-CS, with suspected cases of SSI evaluated at the health facility. From June 2021 to April 2022, post-CS SSI data were collected and uploaded to REDCap.

**Results**

Baseline assessment, before the training and initiation of reporting tools, showed minimal data being collected on SSI. After the clinical training—which included SSI screening, diagnosis, classification, and treatment per protocols—new registers and reporting tools were introduced to capture and summarize data in the facilities. From June 2021 to April 2022, 2,793 CS were performed at the five facilities. Maternity nurses tracked 81% (2,265) and 73% (2,052) of mothers after CS at Day 7 and 30 days, respectively. Of the women tracked, 3.4 % and 2.4% were diagnosed with SSIs at day 7 and 30 respectively post discharge and managed at the facility. At facility level, SSI rate decreased from 4.4% in Quarter 2 of 2021 (April to June) to 3.3% in Quarter 1 of 2022 (January to March).

**Conclusions**

Community-level tracking of women post-CS is critical and feasible with small investments. Improved communication between nurses, clients and Community Health Volunteers creates additional opportunities to track SSIs and to discuss other important health issues of mothers and the babies, while fostering trusting relationships.

## O265. Vulnerable newborn types: analysis of subnational, population-based birth cohorts for 541,285 live births in 23 countries, 2000 to 2021

### Daniel Erchick^1^, Elizabeth A. Hazel^1^, Joanne Katz^1^, Anne CC Lee^2^, Michael Diaz^1^, Lee Shu-Fune Wu^1^, Robert Black^1^, Vulnerable Newborn Measurement Collaborative Group^3^

#### ^1^Johns Hopkins Bloomberg School of Public Health; ^2^Harvard Medical School; ^3^Multiple Organizations

*BMC Proceedings 2024*, **18(5):**O265

Submission ID #: IMNHC429

Note the full manuscript published in May 2023 in *BJOG:*

(https://obgyn.onlinelibrary.wiley.com/doi/10.1111/1471-0528.17510)

## P266. MomCare: a value-based health care program to improve Maternal, Newborn, and Child Health (MNCH) in Kenya and Tanzania

### Julie Fleischer, Emma Waiyaiya, Rowena Njeri, Liberatha Shija, Johnson Yokoyana, Jonia Bwakea, Mark Van Der Graaf, Nicole Spieker

#### PharmAccess Group

*BMC Proceedings 2024*, **18(5):**P266

Submission ID #: IMNHC428

**Background**

In Sub-Saharan Africa, 200,000 women die annually from pregnancy-related complications, representing 68% of maternal deaths worldwide. Value-based health care (VBHC) could help improve maternal and neonatal health outcomes by promoting a data-driven and patient-centered approach. This presentation will highlight the impact, lessons, and feasibility of implementing VBHC for (MNCH) in low- and middle-income countries.

**Methods**

Improving MNCH outcomes requires early and regular antenatal care, facility-based delivery, and postnatal care, tailored to the needs of individual mothers. Therefore, PharmAccess launched *“MomCare”* in Kenya in 2017, followed by Tanzania in 2019. Using the VBHC framework, MomCare incorporates three dimensions of care: (1) financing for a package of maternal care, (2) quality standards (SafeCare) for health care providers, and (3) actionable data to incentivize patient and provider behaviors. Mothers received a digital wallet (M-TIBA in Kenya) that entitled them to a care bundle encompassing the entire pregnancy journey including postnatal and neonatal care. Quality assessment was done through SafeCare during onboarding and repeated every year. Women consented to data collection through claims/data entry systems, SMS surveys, and call surveys. This allowed us to track utilization behavior, well-being, care experience, and outcomes throughout the journey, as well as risk mitigation by clinics. We implemented a pay-for-performance model and created dashboards with insights and benchmarking information. Clinics received actionable feedback including risk stratification and an overview of mothers to call to encourage facility-based delivery. Field teams supported clinics through periodic, data-based feedback sessions to stimulate continuous quality improvement.

**Results**

MomCare has been implemented in more than 70 clinics across Tanzania and Kenya, supporting more than 55,000 mothers. Throughout the program, we measured improved adherence to maternal care, better SafeCare scores, and improved risk mitigation. Providers actively engaged with data-based insights, actionable feedback, and the pay-for-performance system. We were able to identify unmet needs like mental health support for (teenage) mothers or breastfeeding support and adapted the care bundle accordingly. Importantly, the program brought transparency on risk mitigation and costs.

**Conclusions**

MomCare is proof that VBHC can be successfully implemented in low- and middle-income countries to improve maternal care.

## O267. Efficacy of oral melatonin in preventing necrotizing enterocolitis among preterm infants in a level III neonatal intensive care unit in the Philippines: a double-blind randomized controlled trial

### Navid Roodaki, Daisy Evangeline Garcia, Maria Theresa Concepcion Zubiri

#### Ilocos Training and Regional Medical Center

*BMC Proceedings 2024*, **18(5):**O267

Submission ID #: IMNHC419

**Background**

Currently there are no established therapeutic options to prevent necrotizing enterocolitis (NEC), one of the most devastating problems that can develop in preterm infants. Melatonin has been shown to enhance NEC outcomes and may be used as a preventive therapy. This study determined the efficacy of oral melatonin in preventing NEC among high-risk preterm infants.

**Methods**

This is a double-blind randomized controlled trial conducted in a Level III neonatal intensive care unit in the Philippines. Newborn preterm infants (28–36 weeks).

**Results**

100 preterm neonates equally divided into two groups were included with no drop out. A statistically significant difference in the hemoglobin level, white blood cell count, platelet count, and absolute neutrophilic count (*p* value < 0.05), and a lower risk of cytopenia among those given melatonin were noted. There was an 80% lower risk of developing NEC among those given melatonin (RR 0.20 [95%CI 0.10–0.41], *p*=< 0.001; NNT:1.852) and lesser number of days before full feeding (RR 0.32 [95%CI 0.18–0.56], *p*=< 0.001; NNT:2.17). Furthermore, there was a 58% lower risk of mortality among those given melatonin (RR 0.42 [95%CI 0.17–1.02], *p*=0.05; NNT:6.25).

**Conclusions**

This study has shown that melatonin, when given as prophylactic treatment among preterm infants weighing <1500 grams, can prevent NEC, shorten time to full feeding, improve blood parameters, and lower mortality rate.

## O268. Multi-pronged Strategy to Improve Maternal and Newborn Quality of Care: A Case Study from Saving Mothers Giving Lives (SMGL) 2.0 Project in Kaduna State, Nigeria

### Paulina Akanet, Obafemi Omole, Benjamin Asemota, Ogochukwu Kehinde, Fatima Fanna Mairami

#### Pathfinder International

*BMC Proceedings 2024*, **18(5):**O268

Submission ID #: IMNHC417

**Background**

Despite significant investment, Nigeria still carries the second highest burden of maternal mortality in the world, contributing about 15% of the total global maternal deaths. Kaduna State in Northern Nigeria has a neonatal mortality rate of 47/1,000 livebirths, higher than the national average of 34/1,000 livebirths. To contribute to a reduction of maternal and neonatal mortality in the country, SMGL 2.0 in collaboration with Kaduna State Government is implementing a multi-pronged strategy to ensure pregnant women have access to safe and quality delivery services in project-supported facilities.

**Methods**

Among 42 private facilities assessed at baseline, the project selected 25 as intervention facilities based on their availability of providers, availability of basic drugs and equipment, and geographical spread and coverage within the state to ensure equitable access to services. Multi-pronged interventions included training on emergency obstetric and newborn care, essential newborn care, and postpartum family planning, as well as supportive supervision of providers. Job aids and guidelines were provided to support quality improvement efforts. A digital health service, askNivi WhatsApp platform, was employed to drive demand for services, alongside trusted traditional birth attendants to escort pregnant women to hospitals. SMGL 2.0 also implemented the innovative SafeCare quality improvement methodology, regular review meetings for learning, and maternal and perinatal death surveillance and response in facilities.

**Results**

Within two years of intervention (September 2020 to June 2022), maternal deaths in the 25 facilities decreased by 65%, while newborn deaths decreased by 5% from baseline. Women who received uterotonics in the third stage of labor increased from 34% to 100%, newborns breastfed and kept warm within one hour of birth increased from 43% to 100%, newborns with asphyxia who were resuscitated increased from 61% to 83%, and women receiving postnatal care within seven days of delivery increased by 77%.

**Conclusions**

Results suggest that the multi-pronged strategy improved health outcomes for both mothers and newborns at supported facilities and can contribute to maternal and newborn mortality reductions in Kaduna State.

## O269. Which first trimester risk-estimation method for pre-eclampsia is most suitable? A model-based impact study

### Luc Smits^1^, Lynn Strijbos^1^, Manouk Hendrix^2^, Salwan Al-Nasiry^2^, Liesbeth Scheepers^2^

#### ^1^Maastricht University; ^2^Maastricht University Medical Centre

*BMC Proceedings 2024*, **18(5):**O269

Submission ID #: IMNHC395

Note the full manuscript published in July 2023 in *AJOG+MFM:*

(https://www.sciencedirect.com/science/article/pii/S2589933323001167?via%3Dihub)

## O270. Technology for Retinopathy of Prematurity (ROP) screening in resource-limited settings: development of a Target Product Profile (TPP)

### Rebecca Kirby^1^, Aeesha Malik^2^, Kara Palamountain^1^, Clare Gilbert^2^

#### ^1^Northwestern University Kellogg School of Management; ^2^London School of Hygiene & Tropical Medicine

*BMC Proceedings 2024*, **18(5):**O270

Submission ID #: IMNHC385

Previously published: https://bmjophth.bmj.com/content/8/1/e001197

## O271. Setting students up for success: an innovative orientation to clinical placement in Sierra Leone

### Jennifer Neczypor, Julie Mann, Chrisencia Owoko, Jenny Rose Wilson, Isha Beckie Sesay

#### Seed Global Health

*BMC Proceedings 2024*, **18(5):**O271

Submission ID #: IMNHC378

**Background**

Sierra Leone has one of the world’s highest maternal mortality ratios. There is currently a critical shortage of midwives to address this situation. The government of Sierra Leone is investing in strengthening and expanding midwifery education. Major challenges hampering progress include inadequate student oversight and a lack of hands-on clinical experience. Numerous factors affect students’ clinical learning; however, the absence of supportive learning environments is a significant barrier.

To strengthen clinical midwifery education and create a more supportive learning environment at Makeni Regional Hospital, a comprehensive, three-day student orientation to clinical practice was developed. Content consisted of teamwork and communication concepts, clinical assessment and documentation, rotation objectives and expectations, self-care strategies, medical ethics, respectful maternity care, and informed consent. Orientation culminated with postpartum hemorrhage and neonatal resuscitation simulation and debrief to promote practical application of all learning content.

**Methods**

Over a 12-month period, 126 midwifery students completed the orientation. After each training day, students anonymously completed a survey evaluating knowledge attainment and offering feedback. A mixed-methods design was used to capture both qualitative and quantitative data.

**Results**

The majority of students reported a good or very good understanding of the topics, with the greatest knowledge attainment in vital signs, neonatal resuscitation, and simulation/debriefing. Students reported that the most useful topics covered were clinical documentation, communication/teamwork strategies, and vital signs. Qualitative data revealed increased awareness in respectful maternity care practices and increased confidence participating in emergency situations such as neonatal resuscitation.

**Conclusions**

Based on these results, the orientation is currently expanding to other midwifery schools nationwide. It is also being adapted for the maternity staff at the regional hospitals that are used as clinical placement sites.

## O272. Interdisciplinary education for newborn care: building dynamic teams to drive collaborative, coordinated, effective implementation

### Elizabeth Molyneux^1^, Josephine Langton^1^, Edith Gicheha^2^, Sara Liaghati Mobarhan^3^, June Madete^4^, George Banda^2^, Jennifer Werdenberg-Hall^5^, Christina Samuel^2^, Chinyere Ezeaka^6^, Nahya Salim^7^, Grace Irimu-Thinwa^8^, Josephat Mutakyamilwa^9^, Opeyemi Odedere^2^, Grace Tahuna Soko^1^, Dolphine Mochache^10^

#### ^1^Kamuzu University of Health Sciences; ^2^NEST360; ^3^United Nations Children’s Fund; ^4^Kenyatta University; ^5^Texas Children’s Hospital; ^6^University of Lagos ; ^7^Muhimbili University of Health and Allied Sciences; ^8^University of Nairobi; ^9^Ifakara Health Institute; ^10^Kemri Wellcome Trust

*BMC Proceedings 2024*, **18(5):**O272

Submission ID #: IMNHC377

**Background**

Global interest has pivoted to support improvement in quality of newborn care. Women are encouraged to deliver in health units attended by a skilled team. But who are the team, how are they trained, and is appropriate equipment available? Traditionally, the team is doctors and nurses, but they are members of a large group of interdependent experts from other disciplines. Each discipline trains separately, yet the goal of good neonatal care is common to all. To achieve this goal we must learn together, understand and value each other’s roles, and become a cohesive, effective team.

**Methods**

NEST360, an international alliance united to end preventable newborn deaths in African hospitals, is operational in Malawi, Tanzania, Kenya, and Nigeria. Biomedical and clinical experts from these countries reviewed national pre- and in-service teaching materials for clinicians, nurses, and biomedical engineers. Findings were discussed at national stakeholder meetings attended by representatives from every authoritative body, training school, and project involved in newborn care. NEST360 received enthusiastic support to develop context-specific teaching materials to fill the gaps, especially in the use and maintenance of medical devices. An interdisciplinary writing team drafted evidence-based materials that were reviewed by a wider NEST education group, followed by external expert review before being finalized. From these generic clinical and technical modules, job aids for available devices were produced, explanatory videos made, scenarios and quizzes written, and an interdisciplinary generic instructors’ course developed.

**Results**

The clinical modules are embedded into all four countries’ neonatal guidelines and training courses. Job aids have been distributed with every device to all NEST newborn units. More than 35 short videos demonstrate how and when to use a device. Scenarios are used in training and mentoring. Technical and clinical modules and a NEST biomedical course have been included in curricula. Technical and clinical personnel together learn different methods and skills in adult teaching in a Generic Instructor Course.

**Conclusions**

Learning together is an enriching experience. Interactive learning makes theory relevant and puts knowledge into practice. No single method of training suits everyone; materials must be adaptable and available in different formats. An expert does not always make a good teacher; we need to be taught how to teach.

## O273. Digitization and adaptation of the World Health Organization (WHO) integrated management of newborn and childhood illness for an electronic clinical decision support tool for sick young infants

### Gillian Levine

#### Swiss Tropical and Public Health Institute

*BMC Proceedings 2024*, **18(5):**O273

Submission ID #: IMNHC374

**Background**

Adherence to evidence-based clinical guidelines is poor in many low- and middle-income countries, obstructing progress in ending preventable newborn and young infant deaths. Electronic clinical decision support algorithms (CDSA) have improved care quality for older children, but few have been tested for young infants. We developed digital adaptations of evidence-based guidelines for implementation research of CDSA with and without point-of-care diagnostics, to improve appropriate management of sick young infants.

**Methods**

CDSAs were based on the WHO Integrated Management of Newborn and Childhood Illness Management of the Sick Young Infant Aged Up to 2 Months 2019 (IMNCI), and adapted for Tanzania, Rwanda, Kenya, Senegal, and India. Country-specific IMNCI adaptations and relevant national guidelines were collated, summarized, and translated from narrative into human-readable algorithms for digital use. Inconsistencies, gaps, and questions on interpretation were identified and addressed with national expert panels, who were consulted to determine scope and ensure consistency with local guidelines, health worker training, and local care system structures and services. Experts reviewed clinical workflow diagrams and content summaries. Tools were piloted with experts and health workers using clinical vignettes and supervised and unsupervised clinical consultations.

**Results**

Digitization and adaptation resulted in unique, country-specific CDSA content. Inconsistencies and gaps in national guidelines often resulted from incomplete or inconsistent adoption of global guideline updates and from integrating components of several different guidelines, such as signs of possible serious bacterial infection, and weight and age cut-offs from the WHO Pocket Book of Hospital Care for Children and Integrated Management of Pregnancy, Childbirth, Postpartum and Newborn Care. Experts recommended integrating additional content, such as eye problems, birth anomalies, and point-of-care tests (e.g., glucose testing), and identified local organizational considerations, such as when HIV services were managed in specialized clinics rather than primary care. Health workers identified pragmatic considerations, such as commonly-understood terminology, and treatment availability such as antibiotic formulations. Content revisions and software developments addressed country-specific needs.

**Conclusions**

Despite global guidelines, countries have unique needs that must be addressed when digitizing tools at national level. Robust engagement with local stakeholders for adaptation and digitization is essential to ensure quality improvements and sustainability.

## O274. Experiences of pregnancy and childbirth care during the COVID-19 pandemic in Nampula Province, Mozambique

### Megan Lydon^1^, Joaquim Vilanculos^1^, Carter Crew^1^, Américo Barata^2^, Emily Keyes^1^

#### ^1^FHI 360; ^2^Instituto Nacional de Saúde, Mozambique

*BMC Proceedings 2024*, **18(5):**O274

Submission ID #: IMNHC363

**Background**

Pandemic-related health service adaptations raised concerns about provision of quality, respectful maternity care globally. Despite this, little research has focused on the experiences of clients using antenatal and intrapartum care during the pandemic. This study aimed to elevate the voices and document the experiences of pregnant and birthing people in Nampula Province, Mozambique during the COVID-19 pandemic.

**Methods**

In this longitudinal qualitative study, we conducted 24 in-depth interviews with pregnant participants in March 2021 to explore their experience of antenatal care. We conducted follow-up in-depth interviews with 17 participants who had a vaginal live birth through August 2021 to explore their experience of labor and delivery care. Interviews were conducted in Makua and Portuguese, were audio-recorded, transcribed verbatim, and translated into English. We applied thematic content analysis.

**Results**

Participants did not express major concerns about COVID-19 or related service adaptations when describing their experiences of antenatal or intrapartum care. Some noted its negative effects on elements of respectful care such as restricting birth companions and limiting movement during labor to adhere to social distancing policies. Overcrowding became more concerning due to the threat of infection. While unclear if affected by the pandemic, all participants who gave birth at a health facility reported experiencing at least one form of mistreatment, some recounting threats of cesarean delivery. Most explained that they and their newborns received care without their consent, especially regarding enemas and episiotomies. At the same time, respondents described a range of intrapartum experiences that included both respectful and disrespectful care. Most recalled positive verbal communication with their providers and many described receiving continuous attentive care. Participants explained that their satisfaction with childbirth services was tied to their birth outcome and their experience of respectful care.

**Conclusions**

Steadfast commitments to quality of care are critical to ensure families benefit from high-quality, respectful care at all times. The findings from this study signal the ramifications of the COVID-19 pandemic and the need for tighter connections between maternal health and emergency preparedness stakeholders, as well as improvements in the ability to rapidly adapt to health shocks.

## O275. Maternal mid-upper arm circumference as a predictor of low birthweight outcome among newborn deliveries of adolescents in a tertiary level hospital

### Avegail Cardinal^1^, Ma Emma Alesna-Llanto^2^, Vanessa-Maria Torres-Ticzon^2^

#### ^1^Baguio General Hospital and Medical Center; ^2^Philippine General Hospital

*BMC Proceedings 2024*, **18(5):**O275

Submission ID #: IMNHC355

**Background**

Maternal malnutrition is a major cause of low birthweight (LBW) newborn outcome among adolescent mothers. However, getting an accurate nutritional status is challenging in the absence of pre-pregnancy weight among adolescents who often delay their first antenatal visit. Maternal mid-upper arm circumference (MUAC) was proven to be a good proxy measure of acute malnutrition; however, there is no global consensus on what cut-off point to use among pregnant adolescents. Finding the optimal MUAC cut-off could facilitate early recognition and intervention, and this could eventually break the intergenerational cycle of malnutrition.

**Methods**

A cross-sectional study was conducted among adolescent ages 10–19 years who delivered babies in a tertiary hospital in the Philippines for a period of six months. Maternal MUAC and LBW outcome were documented, and their association determined using a logistic regression analysis. To measure diagnostic accuracy, the sensitivity, specificity, and area under the curve were taken for each MUAC point. A receiver operating characteristic (ROC) curve was used to aid the MUAC cut-off determination.

**Results**

Out of 237 newborn deliveries, 35% had LBW, while 65% had normal birth weight. The crude association for the MUAC cut-offs <23.00cm, <23.50cm, and <24.00cm and LBW showed a significant value of 2.19, 2.25, and 2.39 at 95% CI, respectively. However, it is only the cut-off <24.00 cm that showed significant result for adjusted association. The MUAC cut-off <24.00cm also showed a better trade-off value between the sensitivity and specificity. Furthermore, the optimal maternal MUAC measurement that predicts LBW newborn outcome points to <24.00cm cut-off based on the ROC curve.

**Conclusions**

This study shows that the maternal MUAC is predictive of low birth weight outcome among adolescent deliveries. A MUAC cut-off of <24.00 cm was superior to lower cut-offs studied. The pregnant adolescents might need higher MUAC cut-off than adults to allow timely intervention and prevention of poor neonatal outcome. By doing this simple screening test, suspected pregnant adolescents can be easily identified and referred for further confirmatory testing.

## P276. Delivery of intermittent preventive treatment of malaria in pregnancy at the community level to reduce the access barriers of care: results of a cluster-randomized trial in Malawi

### Sarah Birse^1^, Rudolph Thetard^1^, Tamar Chitashvili^1^, John Munthali^2^, Ethel Chilima^1^, Jobiba Chinkumba^3^, Tyson Volkman^4^, Monica Bautista^4^

#### ^1^Management Sciences for Health; ^2^Population Services International; ^3^Malaria Alert Center; ^4^United States Agency for International Development

*BMC Proceedings 2024*, **18(5):**P276

Submission ID #: IMNHC338

**Background**

Malaria in pregnancy is associated with poor maternal and perinatal health outcomes. The World Health Organization (WHO) recommends administering three or more doses of intermittent preventive treatment of malaria with sulfadoxine-pyrimethamine (IPTp-SP) for all pregnant women at least one month apart, starting after 13 weeks of pregnancy. In collaboration with key local stakeholders, the USAID ONSE Health Activity explored the feasibility of community-based delivery of IPTp-SP (cIPTp) via community health workers (CHWs) to address inequities in antenatal care (ANC) attendance and IPTp3+ coverage.

**Methods**

A cluster-randomized, controlled trial was conducted to assess the effect of delivery of IPTp by CHWs on the coverage of IPTp3+ and ANC visits in Malawi. cIPTp was implemented within two districts over a 21-month period (November 2018–July 2020). In control sites, IPTp was delivered at health facilities. Representative samples of women who delivered in the prior 12 months were surveyed at baseline (*n*=370, December 2017) and endline (*n*=687, August 2020). A difference-in-differences analysis was conducted to assess the change in coverage of IPTp and ANC over time, accounting for clustering at the health-facility level.

**Results**

At baseline, women received a mean of 2.3 IPTp doses across both arms; at endline, women received a mean of 2.8 doses. Despite overall increase in coverage, the change in IPTp3+ coverage was not significantly different between intervention and control groups. ANC4+ coverage increased significantly in the intervention group compared with the control group and the baseline (25.3%). Detailed results will be presented upon acceptance and include analysis of IPTp and ANC utilization data with difference-in-differences analysis and confidence intervals.

**Conclusions**

Updated WHO recommendations suggest CHWs and other delivery methods to improve the uptake of IPTp, while emphasizing ANC contacts as an important platform for providing IPTp. Study findings offer potential solutions to strengthen linkages between community- and facility-based services to address persistent barriers of seeking and receiving ANC through CHWs. Further research is needed, however, to identify the systemic requirements and implementation approaches to operationalize the updated WHO guidance on expanding IPTp service delivery by CHWs.

## O277. Complementary feeding and growth among low birthweight infants in the second half of infancy: results from a multisite observational cohort

### Linda Vesel^1^, Christopher Duggan^2^, Bhavna Koppad^3^, Karim Manji^4^, Kate Miller^5^, Tisungane Mvalo^6^, Melda Phiri^6^, Friday Saidi^6^, Latha Shamanur^7^, ES Siddhartha^8^, Christopher Sudfeld^9^, Katherine Semrau^1^

#### ^1^Ariadne Labs; ^2^Boston Children’s Hospital; ^3^KLE Academy of Higher Education and Research; ^4^Muhimbili University of Health and Allied Sciences; ^5^Stanford University; ^6^UNC Project Malawi; ^7^SS Institute of Medical Sciences and Research Center; ^8^Women and Children Hospital, India; ^9^Harvard T.H. Chan School of Public Health

*BMC Proceedings 2024*, **18(5):**O277

Submission ID #: IMNHC328

**Background**

This study was designed to address the high prevalence of infant undernutrition among low birthweight (LBW, <2.5kg) infants in the second half of infancy, and the dearth of evidence on the optimal transition and provision of complementary feeding and growth of LBW infants. We aimed to understand the complementary feeding profile and growth outcomes in the second half of infancy.

**Methods**

The results presented in this abstract are part of the formative, multisite, observational Low Birthweight Infant Feeding Exploration (LIFE) cohort study conducted in India, Malawi, and Tanzania from September 2019 to July 2021. This study collected data on over 1,100 moderately LBW infants throughout infancy including a six, nine, and 12-month visit. Descriptive and multivariate models were used to analyze the data.

**Results**

A total of 1,114 infants and their mothers were enrolled in the observational study. More than 95% of infants were fed breastmilk at six, nine, and 12 months. At nine and 12 months, respectively, 48% and 31% of infants were not achieving a minimum acceptable diet, highlighting gaps in dietary diversity. A lack of dietary diversity was exacerbated among infants born to younger, less educated and/or multiparous mothers. Proteins were more limited than any other food group; only 66% and 70% of infants were fed animal source foods at nine and 12 months, respectively. Prevalence of stunting (>30%) increased from six to 12 months, while underweight (26%) and wasting (10%) remained consistent; regional differences were observed. Importantly, poor growth outcomes at six months were predictive of those at 12 months even when adjusted for key covariates (stunting RR=2.84, *p*<0.001; underweight RR=6.41, *p*<0.001; wasting RR=4.89, *p*<0.001).

**Conclusions**

Given gaps in optimal quality of complementary feeding and the predictive relationship between six- and 12-month growth outcomes, efforts are needed to promote feeding counseling before and during the transition to complementary feeding and to address key barriers to the provision of a diverse diet. In addition, growth monitoring and proactive intervention at six months or earlier could serve to prevent poor outcomes at one year of life. These new learnings can impact current health system provision

## O278. Social determinants of antenatal depression among women in rural Bangladesh: a cross-sectional study

### Nafisa Insan^1^, Simon Forrest^2^, Aqil Jaigirdar^3^, Reduanul Islam^3^, Judith Rankin^1^

#### ^1^Newcastle University; ^2^Durham University, ^3^Maternal Aid Association

*BMC Proceedings 2024*, **18(5):**O278

Submission ID #: IMNHC326

Note the full manuscript published in January 2023 in *Int. J. Environ. Res. Public Health:*

(https://www.mdpi.com/1660-4601/20/3/2364)

## O279. A quality improvement collaborative to enhance Maternal and Newborn Health (MNH) care delivery by adopting global guidance within local contexts in low-resource settings

### Anuradha Pichumani^1^, Pichumani Parthasarathi^1^, Rose Molina^2^, Katherine Semrau^2^

#### ^1^Sree Renga Hospital; ^2^Ariadne Labs

*BMC Proceedings 2024*, **18(5):**O279

Submission ID #: IMNHC322

Note the full manuscript published in November 2023 in *GHSP:*

(https://www.ghspjournal.org/content/11/Supplement_1/e2300007)

## O280. Harnessing the power of behavioral science and co-design to improve the quality of maternity care in rural facilities in Madagascar

### Jana Smith^1^, Maddie Kau^1^, Sara Flanagan^1^, Tina Razfinimanana^2^, Marie Sandra Lennon^3^, Ingabire Magera^4^

#### ^1^ideas42; ^2^United Nations Children’s Fund; ^3^NGO Saint Raphael Madagascar; ^4^Management Sciences for Health

*BMC Proceedings 2024*, **18(5):**O280

Submission ID #: IMNHC321

**Background**

Postpartum hemorrhage (PPH) is the leading direct cause of maternal deaths worldwide, and women in low-income countries have an increased likelihood of dying from PPH-related consequences. Despite clear clinical guidelines, research has often highlighted gaps in consistent application these practices.

**Methods**

We applied the behavioral design methodology to identify the barriers inhibiting providers in rural facilities in Madagascar from following PPH care best practices and co-design solutions to address these barriers. In the diagnosis phase, we conducted 47 qualitative, in-depth interviews with health care providers, postpartum women, traditional birth attendants, and others. In the design phase, we engaged in co-design activities with district and national government officials and nonprofit partners, followed by iterative co-design with 30 individuals across 17 health facilities. Interventions were implemented in 10 facilities in two districts and implementation research was conducted to assess feasibility, desirability, adoption, and promise for impact.

**Results**

The solutions from this process included: (1) a risk visualization exercise; (2) family task badges; (3) an oxytocin timer, a custom device to remind providers of the one-minute window to administer oxytocin after birth; and 4) a glow algorithm poster. Results from implementation research suggested that all of the solutions were feasible to implement and adopted by intended users. The timer, badges, and glow poster were all deemed to be very desirable by providers and family members. While the study design did not allow impact on provider behavior to be empirically assessed, qualitative feedback from providers suggested a shift in timely administration of oxytocin and improved communication with family members.

**Conclusions**

Efforts to improve quality of care have not always systematically considered challenges from a behavioral lens or employed behavioral evidence to design intervention approaches. This work highlights the promise of applying behavioral science to identify underlying drivers of gaps in clinical practice and to develop innovative and desirable solutions to address those gaps.

## O281. Improving maternal health outcomes by enhancing provider performance through innovative technologies in low-resource settings

### Jana Smith^1^, Maddie Kau^1^, Sara Flanagan^1^, Tina Razfinimanana^2^, Marie Sandra Lennon^3^, Ingabire Magera^4^

#### ^1^ideas42; ^2^United Nations Children’s Fund; ^3^NGO Saint Raphael Madagascar; ^4^Management Sciences for Health

*BMC Proceedings 2024*, **18(5):**O281

Submission ID #: IMNHC320

**Background**

According to the World Health Organization, oxytocin is best given within one minute after birth, making timely administration of oxytocin the most important intervention to reduce postpartum hemorrhage. Despite these guidelines, health providers do not consistently adhere to this best practice. Psychological research supports that feedback can improve clinical performance. In low-resource settings, opportunities for regular supervision and mentoring may be limited, and alternative sources of feedback that are appropriate, cost-effective, and sustainable are critical.

**Methods**

We developed a custom “oxytocin timer” that reminds busy providers in low-resource facilities how long they have to administer oxytocin with beeps and a simple countdown display. The timer uses minimal energy and can be charged with solar power. Providers activate it through the touch of their elbow, making it easy to use when attending deliveries alone. The device also registers time of birth and has extra timer functions for other clinical tasks. Data dashboards could be built to give tailored performance feedback to clinicians and support performance improvement and also fill a gap in supervisory support.

**Results**

Results from implementation research suggested high levels of adoption of the timer, with almost 80% of observed facilities having used the timer in the last 24 hours. Providers remarked on the feasibility of incorporating the timer into existing care and highlighted the utility of the timer in reminding them to apply oxytocin in a timely fashion. While the study design did not allow impact on provider behavior to be empirically assessed, qualitative feedback from providers suggested a shift in timely administration of oxytocin.

**Conclusions**

Behavioral science can be used to conceptualize, design, and test innovative technologies that can shift clinical behavior with a focus on improving health outcomes. High rates of adoption and reported desirability of the timer in our study point to the importance of co-designing solutions together with providers so that providers derive utility from them and are motivated to use them consistently—thus strengthening their potential for impact.

## O282. Nigeria’s adaptation of World Health Organization (WHO) clinical guidelines on health care for women subjected to intimate partner violence or sexual violence to strengthen post-gender-based violence service provision

### Chioma Oduenyi, Hannatu Abdullahi, Sylverius Obafemi, Myra Betron

#### Jhpiego

*BMC Proceedings 2024*, **18(5):**O282

Submission ID #: IMNHC282

**Background**

It is widely reported that health care professionals are often the first point of contact for survivors of sexual and gender-based violence (GBV). Hence, strengthening providers’ capacity for quality, survivor-centered post-GBV care is a pre-requisite for effective GBV response. In 2021, Nigeria’s Federal Ministry of Health (FMoH) and partners adapted the WHO clinical guidelines on health care for women subjected to intimate partner violence or sexual violence for countrywide use. The MOMENTUM Country and Global Leadership Project (MCGL) became the first partner to train providers on the adapted clinical guidelines in Sokoto and Ebonyi States.

**Methods**

The FMoH convened several meetings to adapt the guidelines to Nigeria’s context, with key technical and financial support from WHO and MCGL. MCGL trained providers using the adapted National GBV clinical response guidelines and provided job aids, screening checklists, registers, counseling flipbooks, client booklets, and posters. These were followed with regular supportive supervision and mentoring using a structured supervision tool that assessed fidelity to first-line clinical response and regular health-facility register reviews for proper documentation. Gaps identified were addressed through facility-based, on-the-job training and coaching.

**Results**

Nigeria’s adaptation refocused on broader GBV service provision, a shift from WHO’s women-focused guidelines. Of the 112 nurses/midwives trained, 105 (or 94%) initiated GBV services immediately after training. Six months after regular supervision and mentoring to strengthen skills and competencies further to screen, identify, and respond to GBV cases, all trained providers had started providing GBV services. Providers screened 89,582 clients, identified 2,092 cases, provided treatment to 1,799 clients, and referred 19 for non-clinical GBV services. This activity, therefore, ensured that 100% of MCGL-supported health facilities now provide GBV survivor-centered services, compared to 0.4% before the MCGL program as measured by formative assessment.

**Conclusions**

Contextualizing the WHO guidelines helped to standardize and reinforce provider skills and knowledge to provide quality post-GBV care and initiate GBV screening and first-line clinical support. Scaling up GBV training to more Nigerian states using the adapted guidelines would strengthen the health sector response to intimate partner and sexual violence.

## The Respectful Maternity Care (RMC) charter: universal rights of women and newborns to respectful care - india adaptations and endorsements

### Tina Ravi, Aparajita Gogoi

#### Centre for Catalyzing Change

*BMC Proceedings 2024*, **18(5):**

Submission ID #: IMNHC244

**Background**

Respectful maternity care (RMC) is a universal human right that is due to every childbearing woman in every health system around the world. To ensure that women do not undergo disrespect and abuse while availing of maternity care services, and to advocate that women and their newborn have equal rights to respectful care, the Centre for Catalyzing Change (C3)/White Ribbon Alliance India (WRAI) field-tested, adapted, and disseminated the global Charter: Universal Rights of Women and Newborns to Respectful Care.

**Methods**

The RMC Charter was tested in two states of India, with about 500 stakeholders comprised of women, health care providers, civil society organization partners, and decision-makers. Subsequent to the field testing, a national-level committee of maternal health advocates deliberated and outlined an RMC Charter, that was suited to the Indian context. This revised Indian Charter has 12 rights of childbearing women and their newborns, two more than the global version. Once the RMC Charter was finalized, it was imperative to generate endorsement for it and create public salience on the 12 rights in it. This need was addressed through a social media campaign with the hashtag #baarahaqhamara (Our 12 Rights). To provide a comprehensive platform for this campaign, a micro-site—https://www.c3india.org/wrai-rmc-charter—was created and populated with a tool kit, the RMC Charter in multiple languages, static posts, a provision for organizations to submit their logos as their endorsements, and films on the 12 rights. The films were made in a format that does not require a voice over or on-screen text, and are thus not limited by language restrictions and can be used across geographies.

**Results**

To date, the charter has been endorsed by 73 organizations that include national and international organizations, hospitals, and medical and nursing colleges. The campaign reached approximately two million people on social media. The charter has also been incorporated into many C3/WRAI RMC training programs

**Conclusions**

The charter’s field testing, adaptation, dissemination, and endorsement established it as an important advocacy tool for RMC. The charter centralizes the issue of disrespect and abuse in the conversations around quality of care, thus ensuring that women and newborns receive care that is respectiful, equitable, and dignified.

## O284. Target product profiles for neonatal care devices: systematic development and results with NEST360 and the United Nations Children’s Fund (UNICEF)

### Rebecca Kirby^1^, Kara Palamountain^2^, Elizabeth Molyneux^3^, Beverly Bradley^4^, Cindy McWhorter^4^, Martha Gartley^5^, Chinyere Ezeaka^6^, Nahya Salim^7^, Grace Irimu-Thinwa8, Queen Dube^9^, Jennifer Werdenberg-Hall^10^, Maria Oden^2^, Rebecca Richards-Kortum^2^

#### ^1^Northwestern University Kellogg School of Management; ^2^NEST360; ^3^Kamuzu University of Health Sciences; ^4^United Nations Children’s Fund; ^5^Clinton Health Access Initiative; ^6^University of Lagos; ^7^Muhimbili University of Health and Allied Sciences; ^8^University of Nairobi; ^9^Ministry of Health, Malawi; ^10^Texas Children’s Hospital

*BMC Proceedings 2024*, **18(5):**O284

Submission ID #: IMNHC241

**Background**

Medical devices are critical to providing high-quality, hospital-based newborn care, yet many of these devices are unavailable in low- and middle-income countries (LMICs) and are not designed to be suitable for these settings. Target Product Profiles (TPPs) are often utilized at an early stage in the medical device development process to enable user-defined performance characteristics for a given setting. TPPs can also be applied to assess the profile and match of existing devices for a given context.

**Methods**

We developed initial TPPs for 15 newborn devices for LMIC settings. A Delphi-like process was used to develop the TPPs. Respondents completed an online survey where they scored their level of agreement with each of the proposed performance characteristics for each of the 15 devices. Characteristics with <75% agreement between respondents were discussed and voted on using *Mentimeter*^*TM*^ at an in-person consensus meeting.

**Results**

The TPP online survey was sent to 180 people, of which103 responded (57%). The majority of respondents were implementers/clinicians (51%, 53/103), with 50% (52/103) from LMIC. Across the 15 TPPs, 403 (60%) of the 668 performance characteristics did not achieve >75% agreement. Areas of disagreement were reviewed by 69 participants at an in-person consensus meeting, with consensus achieved for 648 (97%) performance characteristics. Only 20 (3%) performance characteristics did not achieve consensus, most (15/20) relating to quality management systems. UNICEF published the 15 TPPs in April 2020, accompanied by a report detailing the online survey results and consensus meeting discussion, which has been viewed 7,039 times (as of January 2023).

**Conclusions**

These 15 TPPs can inform developers and enable implementers to select neonatal care products for LMIC. Over 2,400 medical devices and diagnostics meeting these TPPs have been installed in 150 hospitals in Nigeria, Tanzania, Kenya, and Malawi through the NEST360 alliance. Twenty-three medical devices identified and qualified by NEST360 meet nearly all performance characteristics across 11 of the 15 TPPs. Eight of the 23 medical devices are also available in the UNICEF Supply Catalogue. Some developers have also adjusted their technologies to meet these TPPs. There is potential to adapt the TPP process eyond newborn care.

## O285. An integrated quality of care package coupled with a regional approach significantly reduced mortality due to prematurity in Eastern Uganda and Migori County, Kenya

### Gertrude Namazzi^1^, Peter Waiswa^1^, Paul Mubiri^1^, Phillip Wanduru^1^, Darius Kajjo^1^, Nicole Santos^2^, Kevin Achola^3^, Phelgona Otieno^4^, Dilys Walker^2^

#### ^1^Makerere University School of Public Health; ^2^University of California, San Francisco; ^3^Kabarak University; ^4^Kenya Medical Research Institute

*BMC Proceedings 2024*, **18(5):**O285

Submission ID #: IMNHC232

**Background**

Mortality due to prematurity is the leading cause of neonatal deaths worldwide. Intrapartum and immediate postnatal care offers a window of opportunity for reducing most of these deaths. The Preterm Birth Initiative (PTBi) study aimed to implement and assess the effect of an integrated quality of care package of interventions on fresh stillbirths and neonatal mortality due to prematurity in Busoga, Uganda, and Migori County, Kenya.

**Methods**

The PTBi assessed an integrated package of interventions through a pair-matched cluster-randomized control trial in 20 health facilities in Migori County, Kenya, and the Busoga region in eastern Uganda from 2016–2019. The quality of care package consisted of: (1) simulation-based training and mentorship, (2) quality improvement collaboratives, (3) a modified World Health Organization Safe Childbirth Checklist, and (4) data strengthening. Process data were collected weekly during the quality improvement activity periods and shared bimonthly among the staff. Pre/post training tests and video analysis of the simulations were recorded. Data from maternity registers over the implementation period were collected monthly, and 2,938 preterm newborn babies and fresh still births were analyzed using Stata version 14.

**Results**

The results showed a 34% reduced odds of combined fresh stillbirths and 28-day mortality among preterm infants (OR=0.66, 95% CI: 0.54–0.81) in the intervention sites compared to control facilities. The process data revealed improvement in evidence-based practices: administration of antenatal cortical steroids in eligible mothers, use of a partograph to monitor labor, and kangaroo mother care for preterm and low birth weight babies improved from 20% to 90%, 45% to 90%, and 60% to 89%, respectively. The average pre/post-test training scores also markedly improved, from 48% to 70%. Lessons learned included the value of complementary, mutually reinforcing interventions, the importance of leadership engagement throughout, and the benefit of a regional approach, which strengthened a network of study facilities that learned from each other.

**Conclusions**

Evidence-based practices promoted through a regionally-led integrated intervention package with mutually reinforcing components can improve the clinical care for preterm babies, prevent fresh stillbirths and reduce mortality of these infants in a resource-constrained setting.

## P286. Health care providers’ knowledge and practices of detection and management of postpartum hemorrhage following vaginal birth: qualitative evidence from Kenya, Nigeria, and South Africa

### Shahinoor Akter^1^, Gillian Forbes^2^, Suellen Miller^3^, Hadiza Galadanci^4^, Zahida Qureshi^5^, Sue Fawcus^6^, Justus Hofmeyr^7^, Neil Moran^8^, Mandisa Singata-Madliki^7^, Alfred Osoti^5^, Ioannis Gallos^9^, Arri Coomarasamy^10^, Fernando Althabe^9^, Fabiana Lorencatto^2^, Meghan Bohren^1^

#### ^1^The University of Melbourne; ^2^University College London; ^3^University of California; ^4^Bayero University; ^5^University of Nairobi; ^6^University Cape Town; ^7^University of the Witwatersrand; ^8^KwaZulu-Natal Department of Health; ^9^World Health Organization; ^10^University of Birmingham

*BMC Proceedings 2024*, **18(5):**P286

Submission ID #: IMNHC231

**Background**

Postpartum hemorrhage (PPH) is the leading cause of maternal death globally. Most deaths from PPH can be avoided with early detection and timely management; however, critical challenges exist in adherence to clinical guidelines in facilities in both low-resource and high-resource setting areas. A multi-country, cluster-randomized trial (E-MOTIVE) will introduce a clinical care bundle for early detection and first-response PPH management in hospital settings. This formative qualitative study aimed to explore health care providers’ knowledge and practices of PPH detection and management following vaginal birth in Kenya, Nigeria, and South Africa, to inform design and implementation of E-MOTIVE.

**Methods**

A semi-structured qualitative interview topic guide was developed to reflect on clinical relevance of PPH detection/management and associated knowledge and skills. Forty-five midwives, nurses, and doctors working in maternity wards across Kenya, Nigeria, and South Africa were interviewed. A thematic analysis approach was used.

**Results**

Five key themes were identified, which varied across countries: limited in-service training on PPH, limited knowledge of PPH, challenges with PPH detection, different (or varied) current practices of PPH management, and challenges of managing primary PPH. Although participants considered PPH an obstetric emergency, knowledge about PPH varied among different cadres of health workers. The subjective nature of visual estimation of blood loss was recognized as the key barrier to early detection of PPH. Waiting for the doctor’s arrival or lack of prescription authority created delays initiating PPH management. Shortages of skilled staff and essential resources and late referrals were common management barriers.

**Conclusions**

There is a need to address context-specific barriers of early detection and timely implementation of PPH management. Findings of this study indicate an urgent need to improve PPH detection using objective and reliable methods. Developing evidence-informed implementation strategies and upskilling health care providers on PPH care guidelines might improve PPH management. These findings have been used to develop evidence-informed implementation strategies as part of the E-MOTIVE trial.

## O287. Outborn newborns drive birth asphyxia mortality rates: a nine-year analysis at a rural level 2 nursery in Uganda

### Anna Hedstrom^1^, Paul Mubiri^2^, James Nyonyintono^3^, Harriet Nambuya^4^, Josephine Nakakande^3^, Hilda Namakula^5^, Brooke Magnusson^5^, Molly MacGuffie^5^, Mushin Nsubuga^3^, Peter Waiswa^2^, Maneesh Batra^1^

#### ^1^University of Washington; ^2^Makerere University School Of Public Health; ^3^Kiwoko Hospital; ^4^Jinja Regional Referral Hospital; ^5^Adara Development

*BMC Proceedings 2024*, **18(5):**O287

Submission ID #: IMNHC214

**Background**

Birth asphyxia is a leading cause of global neonatal mortality, with the vast majority of cases in low- and middle-income countries. While the global death rate from asphyxia has decreased over the last 30 years, it is increasing in Uganda and makes up half of neonatal deaths. Improved understanding of the risk factors associated with mortality among these patients is needed.

**Methods**

We report outcomes from admissions captured in an electronic dataset of a well-established rural Ugandan level two unit from 2013 through 2021. Patients admitted after 28 days of age or with unrecorded birth location were excluded. “Inborn” patients were born at the hospital studied and “outborn” were born at another facility or home and then admitted to the hospital studied. Doctors assigned the patient’s primary diagnosis at death/discharge. We performed a Poisson model regression of factors associated with mortality among patients with asphyxia.

**Results**

The study included 8,777 patients, with 53% inborn and 47% outborn. Eighteen percent (1,565) had birth asphyxia. The proportion of outborn birth asphyxia admissions rose from 12% to 73% over nine years. Mortality among all birth asphyxia patients increased over the same period from 9% to 27%. Factors associated with increased death among birth asphyxia patients were outborn status (RR 2.1, [CI: 1.5–2.9]), high or low admission temperature (low: RR 1.9 [CI: 1.4–2.6]); high: RR 1.5 [CI: 1.0–2.2]) and maternal primigravida (RR 1.6 [CI: 1.0–2.6]).

**Conclusions**

Increased birth asphyxia mortality at this referral unit is primarily attributed to increasing admission of outborn patients. Patients born at another facility and transferred face unique challenges. Increased capacity building at lower-level birth facilities could include improved staffing, availability of equipment for labor monitoring, delivery support, and newborn resuscitation as well as training on the timely identification of newborns with birth asphyxia and resources for transfer. These changes may reduce incidence of birth asphyxia, improve outcomes among birth asphyxia patients and help reach global targets for newborn mortality.

## O288. Evaluation of a maternal and infant health intervention in southern nations, nationalities and peoples’ region, Ethiopia

### Muna Gezahegn Wegaso, Russell Dowling

#### ChildFund International

*BMC Proceedings 2024*, **18(5):**O288

Submission ID #: IMNHC212

**Background**

Antenatal care (ANC), skilled care during childbirth, and postpartum care are all necessary tools to reduce maternal and neonatal morbidity and mortality. However, coverage of these services varies considerably in Ethiopia. A recent survey shows ANC coverage at 74%, while access to skilled birth attendants and postnatal care are much lower at 48% and 34%, respectively. The goal of this project was to increase the number of pregnant women who receive quality maternal, newborn, and child health (MNCH) services in intervention districts. The purpose of this evaluation is to assess the impact and sustainability of the intervention.

**Methods**

This intervention was implemented from September 2018 to November 2020. The evaluation was conducted in Dilla Zuria and Wenago Districts from April to June 2021. Survey results were compared to a baseline assessment conducted in 2018. A mixed-method design was used to evaluate the impact and effectiveness of the project. A quantitative study of 125 women of reproductive age with children less than two was implemented, followed by a qualitative assessment including nine key informant interviews and seven focus group discussions. Random sampling was used to identify both quantitative and qualitative study participants. In-person surveys were conducted to collect data that was evaluated in SPSS (v. 2021) and thematic assessment was used to interpret qualitative data.

**Results**

Self-reported ANC service utilization increased from 56.8% at baseline to 92.8% at project completion among participants. Additionally, institutional delivery increased from 79% at baseline to 90.4%. The number of women receiving a postnatal check-up within two days of delivery is slightly higher after the intervention (44.0%) than at baseline (39.2%). The qualitative study showed that the project participants benefited from improved knowledge of MNCH. Additionally, capacity building of health workers and construction of maternal waiting rooms in health centers were well-regarded and perceived as a potential route to sustain service utilization in the absence of additional funding.

**Conclusions**

This evaluation shows that the project has made substantial contributions to the improvement of MNCH services in two districts in Ethiopia.

## O289. Reducing intrapartum stillbirths among pregnant women in Southeastern Liberia: a nursing- and midwifery-led quality improvement initiative

### Garmai A. Forkpah^1^, Marshall Sackey^1^, Daniel Maweu^1^, Yassah Moluwoi^2^, Merab Nyishime^1^, George Methodius^2^, Ibrahim Sanoe^3^, Viola Karanja^1^, Sarah Anyango^1^, Sonnie Kollie^2^

#### ^1^Partners In Health; ^2^Ministry of Health, Liberia; ^3^J.J. Dossen Memorial Hospital

*BMC Proceedings 2024*, **18(5):**O289

Submission ID #: IMNHC209

**Background**

Of the 2.6 million stillbirths globally, 98% occur in low- and middle-income countries. Roughly half of stillbirths happen during labor, referred to as fresh stillbirths (FSBs), the majority of which are preventable. In 2019, J.J. Dossen Memorial Hospital in Liberia reported 46 FSBs per 1,000 births. The hospital has a target of a stillbirth rate of 12/1,000 births or less by 2030. This quality improvement project aimed to reduce FSB rates by 56.5% from 46/1,000 births in 2019 to 20/1,000 births in 2021, by focusing on gaps in clinical care.

**Methods**

A baseline survey of midwifery practices was conducted in 2019 prior to the start of the project. A fishbone analysis was used to identify key causes of FSB. Quality improvement interventions included training and ongoing clinical mentorship in basic emergency obstetric and newborn care. Admission, management of labor, and handover practices policies were updated for obstetric patient care. Direct observation and chart reviews were used to collect post-intervention data. The first Plan-Do-Study-Act (PDSA) cycle ran between January 2020 to December 2021. Stillbirth audits were done for all cases to identify contributing factors.

**Results**

The baseline survey found main causes of FSBs were poor shift handover, delays in partograph documentation, and challenges calling physicians during emergencies. Pre-intervention, bedside handover of fetal heart tones during midwife shift turnover was 0%, timely and consistent use of partograph was 75%, and average physician emergency response turnaround time was 30 minutes. Post-intervention, FSBs dropped by 60.9% to 18/1,000 livebirths in 2021 compared to 46/1,000 livebirths in 2019. Timely partograph use increased to 100%, fetal heart tone assessment during shift turnover improved by 92%, and average physician turnaround time for emergency response dropped to 10 minutes.

**Conclusions**

Most causes of FSBs are related to gaps in timely diagnosis and intervention by providers. Proper monitoring of labor, timely emergency response, and bedside assessment of fetal heart tones during shift turnover for laboring patients are key for reducing FSBs. Training and mentorship in basic and emergency comprehensive care are necessary for obstetric providers. With dedicated focus, Liberia can reduce stillbirth to 12/1,000 births or less by 2030.

## O290. The impact of longitudinal midwifery mentorship on the availability of essential drugs and supplies in five primary health care facilities in Blantyre District, Malawi

### Oveka Jana^1^, Richard Malirakwenda^1^, Luseshelo Simwinga^1^, Kimberly Baltzell^2^, Alden Blair^2^, Miranda Rouse^2^, Anna Muller^2^

#### ^1^Global AIDS Interfaith Alliance; ^2^University of California, San Francisco

*BMC Proceedings 2024*, **18(5):**O290

Submission ID #: IMNHC188

**Background**

Nurses and midwives form the backbone of maternal and newborn health care delivery in Malawi; as such, they should be at the core of interventions aimed at improving respective health outcomes. The University of California San Francisco Global Action in Nursing (GAIN) project aims to improve maternal and newborn health by supporting nurses and midwives with intensive training and longitudinal bedside mentorship. GAIN activities include empowering nurses and midwives to monitor and advocate for the availability of essential drugs and supplies to enable high-quality health care delivery.

**Methods**

Data on the availability of 13 locally-prioritized essential drugs and supplies was collected on a bi-weekly basis from five health facilities via CommCare from April 2019 to July 2022 and amalgamated into quarterly reports as percent availability at each facility. Analyses looked at overall availability, changes over time, facility-level trends, and was compared to qualitative information such as district-level stock-outs and other influencing factors.

**Results**

Following GAIN trainings, all facilities showed immediate and significant improvement in availability of the 13 essential drugs and supplies—from an average of 38% at the start (range: 5%–50%) to 95% by the third quarter (range: 80%–100%). Over time, there was also a decrease in variability of resources across all facilities. Compared to drugs, supplies showed the most variability and were more difficult and costly to replace (e.g., autoclaves and anti-shock garments). Additionally, anti-shock garments were less available after the intervention due to uptake in use. Routine supplies, like urine dipsticks, had the most variability by facility. Trends seen across facilities reflected district-wide shortages. The facility with the lowest patient volume had consistently higher availability of supplies across all quarters (81%), while the two facilities with highest patient volume were more sporadic (56% and 47%).

**Conclusions**

Empowering nurses and midwives through mentorship and training that included the importance of monitoring drugs and supplies led to more consistent availability. While limitations in resource procurement remain, frontline providers can recognize need and advocate for the availability of essential resources. Continuing to uplift nurses and midwives is critical to enhancing health care delivery.

## O291. A qualitative exploration of strategies for improving uptake of postnatal care services in Thyolo, Malawi

### Alinane Linda Nyondo-Mipando^1^, Marumbo Chirwa^1^, Sangwani Salimu^1^, Andrew Kumitawa^1^, Jacqueline Chinkonde^2^, Tiyese Chimuna^2^, Martin Dohlsten^3^, Bongani Chikwapulo^4^

#### ^1^Kamuzu University of Health Sciences; ^2^United Nations Children’s Fund; ^3^World Health Organization; ^4^Malawi Ministry of Health

*BMC Proceedings 2024*, **18(5):**O291

Submission ID #: IMNHC185

**Background**

Although postnatal care services form a critical component of the cascade of care in maternal, newborn, and child health, the uptake of the services has remained low worldwide. Malawi has made remarkable progress in the proportion of women attending antenatal care (95%) as well as delivering at a facility (90%), but has failed to realize the same rates with postnatal care (PNC), which is below 50%. This study explored and prioritized the strategies for optimizing the uptake of PNC services uptake in Thyolo, Malawi.

**Methods**

A qualitative descriptive study was conducted from July to December 2020 in Thyolo District, Malawi, followed by a nominal group technique in October 2021 to prioritize the strategies that were identified. We conducted focus group discussions among postnatal mothers, fathers, health personnel, elderly women, and grandmothers. We conducted in-depth interviews with midwives and key health personnel. We held a nominal group technique among postpartum women, men, midwives, health surveillance assistants, clinicians, and health managers to prioritize the main strategies for provision of PNC. All qualitative data were managed using NVivo and analyzed following a thematic approach.

**Results**

The strategies for optimizing PNC services at the health-system level include: training of health providers; improving clinic operations with attention paid to task-shifting, hours of operation, having appointment date reminders, linkage to care, and provision of free health passport books; having infrastructure for the services; and having services delivered near where end-users reside. At the community level, the strategies include community awareness campaigns that consist of drama groups and health mentors, and male involvement. Additionally, we found that the priority strategies include refresher training and improvement in the clinic operations especially on hours of operation, having appointment date reminders, and linkage to care.

**Conclusions**

Optimization of PNC services will require implementation of strategies that are acceptable and relevant in the context where services are provided. The current education channeled towards the health care workers and the community on maternity services should include an awareness of PNC services that are tailored to address the gaps as outlined by the intended providers and users of the services.

## P292. How Does the Group Antenatal Care (G-ANC) model affect intermittent preventive treatment of Malaria in Pregnancy (IPT-p) uptake in real-world settings?

### Olusesan Makinde^1^, Nchelem Ichegbo^1^, Layi Jaiyeola^2^, Sunday Joseph^3^, Nura Garba^4^, Meg Micheal^1^, Damilola Olaniyan^2^, Adetosoye Adebanjo^2^, Joshua Odunayo Akinyemi^5^, David Okunlola^1^

#### ^1^Viable Knowledge Masters; ^2^Technical Advice Connect; ^3^State Ministry of Health, Nigeria; ^4^Kano State Ministry of Health; ^5^University of Ibadan

*BMC Proceedings 2024*, **18(5):**P292

Submission ID #: IMNHC174

**Background**

Controlled studies have shown that group antenatal care (G-ANC) is associated with increased uptake of IPTp. G-ANC has also been linked to improved health outcomes, and increased demand for health care services. There is limited evidence of real-world implementation of G-ANC, as most documented studies were in controlled environments. This implementation research study investigated how G-ANC affected IPTp uptake in real-world settings after its adoption in Kaduna and Kano States of Nigeria.

**Methods**

The implementation research adopted a mixed-method investigation that collected data prospectively and retrospectively. Data collection was between April and December 2021. Quantitative data was extracted from the records of pregnant women enrolled in G-ANC and those not enrolled in G-ANC prior to its introduction in the same health facilities. Qualitative methods were used to understand challenges that may influence the study outcomes. The data extracted include malaria status through pregnancy and history of IPTp use from both the G-ANC group and the control group. Univariate and bivariate analysis was done on the data using R programming. Qualitative data was thematically analyzed.

**Results**

There was a significant increase in IPTp uptake in Kano State (83% to 94%) after G-ANC was introduced (*p* <0.05). However, there was no significant change following G-ANC introduction in Kaduna State (48% vs 48%). Women in Kano State reported that they were provided IPTp-SP at the facilities at no cost, while this was not the case in Kaduna State. Qualitative data revealed that the provision of IPTp-SP free of cost in health facilities was not a state priority and thus, the state was not investing in this direction.

**Conclusions**

The G-ANC model can improve IPTp compliance in real-world settings. However, this might be influenced by the ability of the client to receive the medications at no cost to them. Financial barriers are a possible risk to IPTp uptake. Strategies for reducing maternal mortality associated with malaria must continue to advocate for removal of financial barriers that limit women from getting medications that will help them and their unborn children.

## O293. Behavior change impact at scale: results and lessons learned from a large, integrated health Social and Behavior Change (SBC) program for Reproductive, Maternal, Newborn, and Child Health (RMNCH) behaviors in Tanzania

### Kara Tureski^1^, Waziri Nyoni^1^, Prisca Rwezahura^1^, Joseph Msofe^1^, Claire Gillum^1^, Mark Lwakatare^1^, JohnBoscoe Basomingera^1^, Theresia Mrema^2^, Lulu Msangi^3^, Donna McCarraher^1^

#### ^1^FHI 360; ^2^Johns Hopkins Center for Communication Programs; ^3^United States Agency for International Development

*BMC Proceedings 2024*, **18(5):**O293

Submission ID #: IMNHC152

**Background**

USAID Tulonge Afya catalyzes opportunities for Tanzanians to improve their health status by transforming sociocultural norms and supporting the adoption of healthier behaviors. The project supports the government of Tanzania to use a life-stage approach and a branded, long-running SBC platform called NAWEZA (or ”I Can”) to integrate promotion of priority RMNCH, family planning (FP), and malaria behaviors with a focus on gateway behaviors.

**Methods**

NAWEZA increases community voice in SBC program design and implementation and holistically addresses families’ needs. Formative and baseline research informed the design of the platform, which targeted pregnant couples and caregivers of young children. NAWEZA was implemented from 2019 to 2021 to address these audiences’ needs, using mutually reinforcing channels like national mass media, and community mobilization, interpersonal communication, and mid-media activities in 29 districts.

**Results**

Significant changes were found in most NAWEZA priority RMNCH, FP, and malaria behaviors, nationally, from baseline to end line. Those who recalled specific NAWEZA messages were significantly more likely than those who did not to report uptake of key behaviors, like early and four-plus antenatal care visits, postnatal care, and MCM use. Service delivery data also demonstrated increased uptake of critical products and services, like MCMs, at key points in time, aligned with intensive SBC programming. Promising approaches employed by the project included the use of an integrated SBC strategy, routine data use for quality improvement, audience co-design/co-delivery of activities, and strong linkages with service delivery activities.

**Conclusions**

Integrated SBC programs have the potential to shift behaviors at a national scale, thereby contributing to healthier RMNCH related norms. Our holistic SBC approach employed three primary means of advancing integrated SBC: (1) use of a life-stage framework, (2) an umbrella brand, and (3) prioritization of gateway behaviors. The endline and use of routine data enabled adjustment of NAWEZA strategies over time and understanding of promising approaches for future replication.

## O294. What makes a difference in a maternal and newborn care programme in improving maternal and newborn care outcomes? Experiences from the West Nile Region, Uganda

### Simon Muhumuza^1^, Grace Latigi^2^, Chimwemwe Msukwa^2^, Anne-Marie Bergh^3^

#### ^1^Makerere University School Of Public Health; ^2^United Nations Children’s Fund; ^3^University of Pretoria

*BMC Proceedings 2024*, **18(5):**O294

Submission ID #: IMNHC148

**Background**

The West Nile region in Uganda has experienced some of the poorest maternal and newborn health (MNH) services. Bottlenecks to MNH services included weak governance and leadership structures, insufficient health workforce, poor infrastructure, presence of a high number of refugees, and barriers such as long distances to health facilities, negative cultural beliefs, gender relations, and widespread poverty. In 2018, UNICEF with the Ministry of Health and partners implemented an integrated package of high-impact MNH interventions to tackle these challenges. Here, we document some of the best practices and key results of the MNH program.

**Methods**

This was an exploratory mixed-methods study in which qualitative and quantitative approaches of data collection were used. The qualitative study captured perspectives of stakeholders at district, health facility, and community levels. Data were collected in four districts through: document review; interviews with key informants at three levels of the health system; focus group discussions with parents and caretakers and with community health workers; and interviews with community individuals whose lives had been impacted by the MNH program. Quantitative data was based on a retrospective analysis of key MNH indicator data from the national health information system.

**Results**

Stakeholders from multiple levels perceived the following interventions and activities as having made a difference in the MNH program: (1) data use for evidence-based decision-making; (2) provision of water, sanitation, and hygiene facilities at health facilities; (3) establishment of neonatal intensive care units and high-dependency maternity units at district hospitals; (4) capacity building of health care workers in various aspects of MNH service delivery; and (5) community referral of pregnant women through a commercial motorcycle voucher referral system. Overall, between 2018 and 2021, the district health system capacity scores increased from 63.5% to 73.4%; client satisfaction with health services significantly improved from 3.4% to 39.8%; maternal deaths reduced by 71%, and stillbirths/1,000 deliveries reduced from 8.2% to 6.9%.

**Conclusions**

The holistic and system-wide approach adopted by the MNH program could have contributed to the key MNH program results in the West Nile region. These interventions should be sustained and considered for scale-up nationally.

## O295. Barriers limiting access to caffeine for preterm infants with apnea of prematurity in Low- and Middle-Income Countries (LMICs): findings from a landscaping conducted in Nigeria, Ethiopia, Kenya, South Africa, and India

### Oluwaseun Aladesanmi^1^, Andrew Storey^1^, Ryan Fu^1^, Zelalem Demeke^1^, Isa Adamu^1^, Betty Wariari^1^, Mansharan Seth^1^, Unathi Beku^1^, Hema Magge^2^, Amanda Cafaro^2^

#### ^1^Clinton Health Access Initiative; ^2^Bill and Melinda Gates Foundation

*BMC Proceedings 2024*, **18(5):**O295

Submission ID #: IMNHC142

**Background**

Every year 15 million babies are born preterm. Preterm complications are the leading cause of death among children less than five years of age, and responsible for one million deaths in 2015. A driver of premature deaths is apnea of prematurity (AOP), which is the cessation of breathing lasting more than 15 seconds. A drug, caffeine citrate, listed in the World Health Organization’s Essential Medicines List and recommended as drug of choice in neonatal guidelines, can prevent and treat AOP. While caffeine is the mainstay of prevention and treatment in high-income countries, it is not widely used in low-income countries where more preterm deaths occur. An alternative, aminophylline, is more commonly used in LMICs, even though evidence shows that caffeine has a better side effect profile, lower toxicity, and requires less monitoring.

**Methods**

A landscaping was conducted across Nigeria, Ethiopia, Kenya, South Africa and the States of Delhi, Bihar, Uttar Pradesh, Telangana, Madhya Pradesh in India to determine barriers limiting access to caffeine for preterm infants with a focus on policy, awareness, availability, use, and price. 107 Ministry of Health officials, neonatologists, pediatricians, doctors, and nurses in 44 public and private neonatal intensive care unit hospitals were interviewed. In addition, we engaged five SRA caffeine manufacturers and 11 in-country distributors/suppliers.

**Results**

Caffeine was listed in Essential Medicines Lists, registered, and recommended in guidelines in all countries surveyed. Awareness of caffeine as the preferential drug for AOP was highest in India (100%) and Nigeria (100%), compared to Ethiopia (50%), Kenya (56%), and South Africa (33%). Across all countries, awareness was mostly higher among pediatricians and neonatologists, compared to other neonatal intensive care unit health professionals. Availability and utilization levels were low across all countries, with India an exception. In contrast, aminophylline was widely available in all countries, with a marked price difference compared to caffeine. Only India and South Africa had caffeine on tender and regulated price, and none of the countries had a plan to scale up usage.

**Conclusions**

Barriers limiting access to caffeine include unfavorable enabling environment, limited and fragmented demand with low visibility, inefficient production, a cheaper alternative, and in-country fees inflating price. Demand and supply interventions are needed to increase access for preterms.

## O296. Factors affecting the implementation of calcium supplementation strategies during pregnancy to prevent pre-eclampsia: a mixed-methods systematic review

### Gabriela Cormick^1^, Rana Islamiah Zahroh^2^, Helen Moraa^3^, John Allotey^4^, Thais Rocha^4^, Shakila Thangaratinam^4^, Ana Pilar Betran^5^, Meghan Bohren^2^

#### ^1^Centro de investigaciones en Epidemiología y salud pública; ^2^The University of Melbourne; ^3^University of Nairobi; ^4^University of Birmingham; ^5^World Health Organization

*BMC Proceedings 2024*, **18(5):**O296

Submission ID #: IMNHC127

**Background**

Hypertensive disorders of pregnancy are among the leading causes of maternal and neonatal morbidity and mortality globally. The World Health Organization recommends calcium-containing supplements daily from 20 weeks’ gestation for women at high risk of pre-eclampsia/eclampsia and for all women from populations with low calcium intake. This research aims to improve the understanding of factors (barriers and facilitators) influencing the uptake and use of calcium supplementation during pregnancy to prevent pre-eclampsia.

**Methods**

We conducted a mixed-method systematic review. We searched MEDLINE, EMBASE, CINAHL, and Global Health databases from the database inception to September 2022. We included qualitative and quantitative studies that explored views of women, health care providers, community members, and other relevant policy-makers about calcium supplementation during pregnancy. We used the Theoretical Domains Framework (TDF) and Capability, Opportunity, and Motivation of Behavior (COM-B) models to identify barriers and facilitators of calcium supplementation implementation to prevent pre-eclampsia/eclampsia. We used the GRADE-CERQual approach to assess the confidence of each qualitative finding and mapped quantitative findings to qualitative themes.

**Results**

We included 16 studies. There was limited knowledge about calcium-containing supplements and pre-eclampsia. Fears and experiences of side effects, varying tablet preferences, dosing, and challenges due to routine were the barriers to calcium supplement use for women. Information regarding pre-eclampsia and the safety of calcium supplementation from reliable sources, options of daily doses, reminders and support from family and community may help increase women’s calcium uptake. Early initiation of antenatal contacts and the provision of calcium supplements at no cost might increase adherence to calcium supplement uptake. Consistent messages, training, and ensuring that an adequate number of human resources and calcium dosage forms could encourage the providers’ use of calcium supplements.

**Conclusions**

When formulating interventions and policies on calcium supplement use, relevant stakeholders should consider the identified barriers and facilitators to optimize benefits. Findings from this study can inform implementation considerations to ensure effective and equitable implementation and scale-up of the provision of calcium-containing supplements in public health.

## O297. Afghan refugee women’s narratives of pregnancy and birth while “On the Move” through Serbia

### Esther Sharma

#### London School of Hygiene & Tropical Medicine

*BMC Proceedings 2024*, **18(5):**O297

Submission ID #: IMNHC126

**Background**

There is a dearth of research exploring the maternal health needs of women during their migratory journeys. Large numbers of Afghan refugees have passed through Serbia during overland journeys to the European Union (EU), often finding themselves “stuck” for months or years in Serbia as the EU has enacted increasingly restrictive border regimes. This study aims to document the lived perinatal experiences of Afghan women in Serbia and explore the provision of maternity care and support for Afghan women in Serbia during the perinatal period.

**Methods**

Using a qualitative study design, data was collected between August 2021 and August 2022 both remotely and during visits to Serbia, to understand the context for Afghan women transiting through Serbia. In addition to unstructured field observations, 11 narrative interviews with Afghan women who had given birth in Serbia were conducted. Narrative analysis was employed to analyze the data.

**Results**

Findings suggest: (1) EU border restrictions are a source of gendered harms and thus have a detrimental effect on Afghan women during the perinatal period; (2) pregnancy and motherhood play a key role in (im)mobility; (3) perinatal mental health and postnatal care, including infant feeding support, are insufficiently addressed among Afghan women in this context; and (4) migratory journeys do not curtail pregnancy and therefore there is a greater need for maternal and newborn health needs to be considered as part of the refugee response in Serbia.

**Conclusions**

This study highlights the challenges created by the EU border regime for Afghan women who are “on the move” during the perinatal period, pointing to an urgent need to address maternal and newborn health needs, provided in a timely manner and incorporating quality of care, for refugee women during migratory journeys.

## O298. Implementing effective quality improvement plans at health facilities to improve maternal and neonatal services in South Africa: identifying key components for replication in resource-constrained settings

### Willem Odendaal^1^, Xanthe Hunt^2^, Terusha Chetty^1^, Mark Tomlinson^2^, Yages Singh^1^, Azukile Nzuzo^3^, Joyce Makgatho^3^, Themba Chauke^3^, Yogan Pillay^3^, Manala Makua^4^, Lutfiyya Khan^3^, Shuaib Kauchali^1^, Ameena Goga^5^

#### ^1^South African Medical Research Council; ^2^Stellenbosch University; ^3^Clinton Health Access Initiative; ^4^South African National Department of Health; ^5^World Health Organization

*BMC Proceedings 2024*, **18(5):**O298

Submission ID #: IMNHC110

**Background**

Maternal and neonatal mortality and stillbirths in South Africa remain relatively high. To address this, South Africa’s National Department of Health launched a multi-partner, facility-based, quality improvement (QI) program (2018–2022), called Mphatlalatsane, in 21 resource-constrained facilities across three high-burden provinces. The program’s aim was to work with facility staff to establish QI teams. These teams identified key areas to improve maternal and newborn health (MNH) services, then developed and implemented the QI plans (QIPs).

**Methods**

An evaluation was conducted (2020–2022), assessing Mphatlalatsane’s impact on maternal and neonatal mortality, still births, maternal experiences of care, quality of care, and implementation processes to inform the replication of effective QIPs. We report the results from the qualitative evaluation of facility-level implementation processes. We purposively selected 14 of the 21 facilities. Three rounds of interviews were conducted (May 2021–September 2022) with QI team leaders and members (health care workers in the participating facilities); regular debriefings were conducted with QI advisors (technical experts supporting the QI teams); and program documentation was reviewed. All data were thematically analyzed.

**Results**

Across the facilities, 28 effective QIPs were developed and sustained, addressing a range of MNH services: promoting early booking of antenatal care visits (five facilities); triaging patients in labor admission and antenatal care high-risk clinics (six facilities); improving completeness of records such as the partogram (five facilities); TB screening of antenatal care patients (three facilities); and QIPs implemented in only one facility (e.g., postpartum family planning). Key components in successful implementation were: involving community health workers and traditional practitioners when needed; upskilling staff to use standard tools and protocols; and developing user-friendly tools to monitor effectiveness. The team leader and QI advisor were key drivers for successful teams. Staff shortages and attrition, and the high-pressured environment of MNH services were barriers to successful implementation.

**Conclusions**

The Mphatlalatsane QI teams developed an inventory of replicable QIPs to improve MNH services in similar settings. The first steps were recently taken with spreading these QIPs in neighboring facilities. Its outcomes are to be carefully monitored to optimise the potential of these QIPs.

## O299. Assessing the needs for Emergency Obstetric and Newborn Care (EmONC) health facilities in Burundi to support Maternal and Newborn Health Service Delivery Redesign (MNH-Redesign)

### Desire Habonimana

#### University of Burundi

*BMC Proceedings 2024*, **18(5):**O299

Submission ID #: IMNHC100

**Background**

Despite Burundi having formed a network of 112 EmONC health facilities, the country continues to struggle with high rates of maternal and newborn deaths. This study generated evidence on the needs of EmONC health facilities to improve maternal and newborn survival.

**Methods**

We conducted a series of cross-sectional surveys on: (1) EmONC health facilities to appraise the capacity in essential EmONC human resources, equipment and supplies; (2) delivery care professionals to assess skills and self-confidence to manage complications; and (3) mothers (via exit interviews) to appraise the quality of care received as well as experience of care.

**Results**

Of 49 health districts, 48 have at least one EmONC health facility but only 18 districts have three or more EmONC facilities. Nationally, less than 49% of the population have access to a functional EmONC facility within two hours. Half of EmONC facilities do not have a midwife, and 30% do not have a nurse trained in basic EmONC. Plus, there is a dearth of EmONC-trained doctors with 70% of facilities lacking at least one. Lack or stock-outs of essential EmONC drugs such as antibiotics and anticonvulsants are widespread at higher proportions and so are stock-outs of basic EmONC equipment such as the neonatal resuscitation kit. Also, while all comprehensive EmONC facilities are functional, 75% of basic EmONC facilities are not. The vast majority of delivery professionals have not yet managed a maternal or neonatal complication including the deadliest complications such as hemorrhage, eclampsia, breech delivery, or neonatal asphyxia. Mother exit interviews revealed that 8% of pregnant women waste 30 minutes on average trying to find the maternity ward and further wait another 11 to 30 minutes to be seen by delivery personnel. Despite the above, mothers are satisfied with the services and their experience of care is positive.

**Conclusions**

The Burundian EmONC network is a promising initiative that can help to improve maternal and newborn survival and boost the country towards achieving the bold Sustainable Development Goal maternal and newborn health targes. However, the study revealed that this network needs adequate and competent human resources and essential equipment and supplies.

## O300. Effect of community-based interventions on preventing stillbirths in Sub-Saharan Africa (SSA): a mixed-method systematic review and meta-analysis

### Uchenna Chinenye Gwacham-Anisiobi^1^, Yebeen Ysabelle Boo^1^, Adetola Oladimeji^2^, Charles Opondo^3^, Nia Roberts^1^, Jennifer Kurinczuk^1^, Manisha Nair^1^

#### ^1^University of Oxford; ^2^Solina Center for International Development and Research, Nigeria; ^3^London School of Hygiene & Tropical Medicine

*BMC Proceedings 2024*, **18(5):**O300

Submission ID #: IMNHC84

**Background**

Globally, there were two million stillbirths in 2019, with 98% occurring in low- and middle-income countries. The burden was highest in Sub-Saharan Africa (SSA) despite efforts to reduce the incidence. We conducted a mixed-methods systematic review and meta-analysis to investigate the types, effectiveness, and acceptability of community-based interventions for preventing stillbirths in SSA.

**Methods**

Seven databases (MEDLINE, Embase, Cochrane CENTRAL, Global Health, Web of Science, CINAHL and GIM) and four grey literature sources were searched for relevant quantitative and qualitative studies (PROSPERO, CRD42021296623). Studies were included if interventions were community-based with or without a health facility component. Main outcomes were types of community-based interventions, risk of stillbirths in intervention versus control communities, and themes related to intervention acceptability. Study quality was assessed using Cochrane risk of bias and National Heart, Lung and Blood Institute’ tools. We calculated pooled odds ratio (OR) using random-effects models and conducted qualitative analysis of acceptability using a theoretical framework.

**Results**

Twenty-seven studies from 17 SSA countries were eligible for inclusion. Four types of interventions were identified: nutritional, infection prevention, access to skilled childbirth, and health knowledge/behavior of women. These were implemented using eight strategies: mHealth, women’s groups, community midwifery, home visits, mass media, traditional birth attendant training, community health worker training, and community mobilization. Odds of stillbirth did not vary significantly between community-based intervention and control groups (OR 0.90; 95% CI 0.74-1.08), but interventions that had both community and health facility components significantly reduced the odds of stillbirth (OR 0.83; 95% CI 0.79-0.87). Quality of the 10 studies included in the meta-analysis were graded as poor (*n*=1), fair (*n*=6), and good (*n*=3). Studies reported high acceptability of the interventions, but most only explored health workers’ perception. The few that explored women’s perceptions, omitted key constructs of acceptability such as ethicality and burden of the intervention.

**Conclusions**

Community-based interventions alone, without strengthening the quality and capacity of health facilities, may not have a substantial effect on reducing stillbirths in SSA. There is also a need to holistically explore acceptability of interventions by women and families to maximize impact.

## O301. Caring for Providers to Improve Patient Experience (CPIPE): mixed-methods evaluation results

### Patience Afulani^1^, Jaffer Okiring^2^, Edwina Oboke^3^, Beryl Ogolla^3^, Monica Getahun^1^, Joyceline Kinyua^4^, Osamuedeme Odiase^1^, Iscar Oluoch^5^, James Odour^6^, Linnet Ongeri^4^

#### ^1^University of California, San Francisco; ^2^The Infectious Diseases Research Collaboration; ^3^Global Programs for Research and Training; ^4^Kenya Medical Research Institute; ^5^County Executive Committee, Migori; ^6^Migori County Department of Health

*BMC Proceedings 2024*, **18(5):**O301

Submission ID #: IMNHC71

**Background**

The “Caring for Providers to Improve Patient Experience (CPIPE)” intervention was developed to improve person-centered maternal care (PCMC) by addressing two intermediate factors—provider stress and bias—contributing to poor PCMC and disparities. CPIPE has five key strategies (provider training, peer support, mentorship, embedded champions, and leadership engagement) and was successfully piloted over six months in two health facilities in Migori County, Kenya. The evaluation seeks to assess the acceptability and preliminary effectiveness of CPIPE.

**Methods**

We used a pre/post-test, non-equivalent control group design, embedded within a mixed-methods approach to evaluate the intervention. This included quantitative data collection using surveys with 80 providers—40 in intervention and 43 in control arms—at baseline (before intervention) and endline (six months later), and in-depth interviews with 30 providers at endline. Quantitative analysis includes a difference-in-difference analysis to assess intervention effects and thematic analysis of qualitative data to assess acceptability.

**Results**

Perceived stress scores changed from 20.9 (SD=3.9) at baseline to 18.6 (SD=5.3) at endline in the intervention group (*p*=0.033); and from 20.6 (SD=4.5) to 20.1 (SD=5.6) in the control group (*p*=0.645). Burnout levels changed from 3.6 (SD=1.0) to 2.9 (SD=1.0) in the intervention group (*p*=0.003); and from 3.7 (SD=1.1) to 3.4 (SD=1.1) in the control group (*p*=0.255). Implicit and explicit bias scores decreased in both the intervention and control arms, but the difference was not statistically significant. A difference-in-difference analysis indicated that 82% and 34% of the change in stress and burnout scores respectively can be attributed to the intervention. Qualitative results indicate high degree of acceptability, with most respondents calling for expansion of the intervention to the whole county. Qualitative themes also illustrate how the intervention has contributed to reduced stress and bias and improved responsive and respectful care to mothers.

**Conclusions**

The CPIPE intervention is acceptable and preliminary analysis shows it is effective in improving providers stress and burnout, with potential to reduce bias. Improvements in these outcomes will lead to improvements in PCMC, especially for the most vulnerable women.

## O302. Caring for Providers to Improve Patient Experience (CPIPE): intervention development process

### Patience Afulani^1^, Edwina Oboke^2^, Beryl Ogolla^2^, Monica Getahun^1^, Joyceline Kinyua^3^, Iscar Oluoch^4^, James Odour^5^, Linnet Ongeri^3^

#### ^1^University of California, San Francisco; ^2^Global Programs for Research and Training; ^3^Kenya Medical Research Institute; ^4^County Executive Committee, Migori; ^5^Migori County Department of Health

*BMC Proceedings 2024*, **18(5):**O302

Submission ID #: IMNHC70

Note the full manuscript published in December 2023 in *Glob Health Action:*

(https://www.tandfonline.com/doi/full/10.1080/16549716.2022.2147289)

## O303. Service loss during COVID-19 for maternal health and immunization services in Bangladesh

### Aminur Rahman Shaheen^1^, Sadika Akhter^1^, Farzana Basher^1^, Iqbal Anwar^2^, Manzoor Ahmed Hanifi^1^

#### ^1^International Centre for Diarrhoeal Disease Research, Bangladesh; ^2^Obstetrical and Gynaecological Society of Bangladesh

*BMC Proceedings 2024*, **18(5):**O303

Submission ID #: IMNHC66

**Background**

The indirect effect of COVID-19 pandemic upon mortality has been poorly monitored in low- and middle-income countries like Bangladesh, and the concern is that this effect might be substantial. In this context, our objective was to quantify and explore support the extent of service loss in routine immunization and preventive maternal health care services from health sector facilities in Bangladesh due to COVID-19 pandemic.

**Methods**

A mixed-method approach was applied to conduct this study. Secondary data was collected from the national data server District Health Information System 2 (DHIS2) and icddr,b surveillance data for the year March 2020–June 2022 and several key informant and in-depth interviews were performed from two subdistricts in Bangladesh. The uptake of all routine antigens and maternal health care services at facility level were evaluated to assess the loss in maternal and child health service uptake due to COVID-19 through comparing mean differences between three time periods (March to December during 2020 and June 2022) using ANOVA test.

**Results**

It is evident from our findings that during the COVID-19 pandemic, utilization of maternal care services (antenatal care, postnatal care, facility delivery, and c-section delivery) and routine immunization services (measles-rubella, BCG, pentavalent, and tetanus toxoid vaccine) significantly reduced from pre-COVID condition (*p* <0.05). Service utilization started to reduce from March 2020, further stepped down in April and May (in some cases June), and then started to rise upward from July. Maternal health care services was more affected in comparision to child immunization services. These finding were supplemented by qualitative data that the health managers and service providers iterate the strength of immunization services more than maternal health services in Bangladesh.

**Conclusions**

The COVID-19 pandemic had profound, negative impact on routine maternal, newborn, and child health services in Bangladesh. The strength of the immunization program was better than maternal health services. Policy-makers should take lessons from the strengths of immunization services in Bangladesh for maternal health services to continue service during pandemic-adverse situation like COVID-19 periods.

## O304. Experiences of midwives regarding provision of culturally competent care to women receiving maternal care in South Africa

### Khumoetsile Daphney Shopo, Tinda Rabie, Antoinette Du Preez, Petra Bester

#### North-West University

*BMC Proceedings 2024*, **18(5):**O304

Submission ID #: IMNHC57

Note the full manuscript published in January 2023 in *Midwifery:*

(https://www.sciencedirect.com/science/article/pii/S0266613822002765?via%3Dihub)

## O305. Community-based peer support to mitigate social isolation and stigma of adolescent motherhood in Harare, Zimbabwe

### Chiwoneso Tinago^1^, Edward Frongillo^2^, Andrea Warren^2^, Vivian Chitiyo^3^, Tiara Jackson^4^, Ashley Cifarelli^5^, Shannon Fyalkowski^6^, Victoria Pauline^7^

#### ^1^West Chester University of Pennsylvania; ^2^University of South Carolina; ^3^The Organization for Public Health Interventions and Development; ^4^NORC at the University of Chicago; ^5^Penn Medicine; ^6^AstraZeneca Foundation; ^7^National Council for Mental Wellbeing

*BMC Proceedings 2024*, **18(5):**O305

Submission ID #: IMNHC55

**Background**

Adolescent mothers often feel isolated, lacking coping skills, resources, social support, and educational opportunities. Unless addressed, these circumstances may have negative consequences for their mental health. To inform the development of a peer-support intervention to mitigate social isolation and stigma of adolescent motherhood, focus groups were conducted from October to December 2018 with adolescent mothers aged 14–18 years, community health workers, and key community stakeholders in Harare, Zimbabwe.

**Methods**

Community health workers (*n*=12) and peer educators (*n*=12) were trained to co-facilitate the peer groups in February 2019. The intervention and control arms included 142 and 105 adolescent mothers (aged 15–18 years) respectively from two low-income high-density communities in Harare. Peer support groups (*n*=12) met in-person twice a month at local community clinics and completed 12 peer-group sessions from May to August 2019. Session plans addressed participant-identified topics that included income generation, gossip, and mental health. WhatsApp Messenger was used to schedule meetings and further discuss topics. Key community stakeholders in the intervention community met in May 2019 (*n*=25; two meetings) and August 2019 (*n*=25; two meetings) to discuss project progress and recommendations to improve the health of adolescent mothers. The intervention and control arms completed surveys to measure and test differential changes over baseline (March 2019), midline (May 2019), and endline (August 2019) in mental health and social support. Sociodemographic and survey data were analyzed with Stata 15 software between March and October 2019.

**Results**

The intervention arm reported lower prevalence of depressive symptoms and common mental disorders and higher overall, significant other, family, and friends support, when compared to the control arm. The intervention arm was more likely to report feeling more engaged with their peers, knowing who and where to turn to for help, and having coping, parenting, and communication strategies to manage challenges.

**Conclusions**

The intervention leveraged peer support, technology, community health workers, and key community stakeholders to develop coping, parenting, and communication skills to mitigate potential stressors and stigma of adolescent motherhood. Recommendations include expanding the intervention to similar communities and integration of these groups to existing services such as prenatal and postnatal care.

## O306. Evolution of reproductive health indicators with the recruitment of community midwives

### Assitan Baya Sidibe, Dramane Traore

#### Direction régionale de la santé, Mali

*BMC Proceedings 2024*, **18(5):**O306

Submission ID #: IMNHC330

**Background**

Human resources for health are insufficient in number and quality and are unevenly distributed throughout the country of Mali, particularly with regard to reproductive health staff. In fact, in 2020, the ratio of health care professionals (doctors, midwives, and nurses) to the population was two per 10,000 inhabitants at the national level and that of the Sikasso region was four per 10,000 inhabitants. The aim of this study is to assess key reproductive health indicators in the second half of 2020 and 2021 in the community health centers after the introduction of community-recruited midwives in the Sikasso region.

**Methods**

The descriptive study and the study site included seven districts (Sikasso, Kadiolo, Kolondièba, Bougouni, Kignan, Nièna, and Koutiala). The criteria defined for the selection of community health centers benefiting from midwives were accessibility and number of deliveries. The data were extracted from the DHIS2 platform for the period starting from the second half of 2020 before the deployment of midwives and six months after their deployment in 2021.

**Results**

The postnatal care (PNC) 1 rate rose from 70.74% in 2020 to 75.18% in 2021. The PNC4 rate rose from 14.99% in 2020 to 17.71% in 2021. The effective PNC rate fell by 2.45 percentage points between 2020 (18.51%) and 2021 (16.06%). The rate of births attended by skilled personnel increased by 14.02 percentage points, from 2020 to 2021. The postnatal consultation rate rose from 23.26% in 2020 to 27.33% in 2021. The prevalence rate increased by 6.36% from 2020 to 2021.

**Conclusions**

The availability of qualified human resources is a key indicator for the improvement of the quality of care and services. However, human resources need to be maintained in the health facilities, especially at the community level.

## O307. Community midwifery deployment initiative: analysis of indicators at one year of implementation in six regions of Mali

### Aminata Cisse, Sidi Mohamed Ben Moulaye Idriss, Ousmane Sylla, Ntji Keita, Seydou Diallo, Nana Kadia Coulibaly, Aïssata Sangare, Amadou Baba Diarra, Brahima Kone

#### Office National de la Santé de la Reproduction, Mali

*BMC Proceedings 2024*, **18(5):**O307

Submission ID #: IMNHC299

**Background**

In Mali, the ratio of health professionals is 2.16 per 10,000 people compared to the World Health Organization’s recommendation of 23 per 10,000 people. The initiative to deploy 200 community midwives from January 2021 to December 2024 is one of the responses to the findings of a joint mission to assess reproductive health/family planning needs in January 2020 and the repercussions of the multidimensional crisis (security, health, COVID-19). This is one of the strategic aims of the eighth Mali-UNFPA cooperation program (CPD8), in line with Mali’s national priorities.

**Objective**: Describe the implementation process of the community midwives’ deployment initiative, challenges, and lessons learned.

**Methods**

Descriptive cross-sectional study was conducted from January 2021 to June 2022. Several meetings were held between the Sexual and Reproductive Health and Rights/General Directorate of Health and Public Hygiene, the Regional Health Directorates, the Order and Association of Midwives of Mali, and UNFPA. A first wave of 50 midwives was recruited and trained between March and May 2021. The midwives were deployed in the six regions: Koulikoro (4), Sikasso (8), Ségou (8), Mopti (8), Timbuktu (11), and Gao (11) in June 2021. A midwifery training mission was conducted in all six regions. The midwives completed an internship in the reference district hospitals, which lasted for about one month. Several supervision missions were carried out to monitor implementation of the initiative.

**Results**

Integration into the communities and the appropriation of work in collaboration with local staff: The retention rate of midwives on site was 70%, but this varied by region. Reproductive health indicators at the various duty stations improved. On average, the number of deliveries in the centers increased by 34% in all six regions. The number of deliveries increased from 712 in 2020 to 1,488 in 2021 in the Sikasso health centers. The Timbuktu centers had a total of 6,201 new family planning users in 2020, compared with 7,386 in 2021.

**Conclusions**

Improvements in retaining qualified personnel in rural areas and in reproductive health indicators suggests that the initiative should be extended to all regions, prioritizing humanitarian areas.

## O308. Experience sharing on the strategy to increase the number of midwives in the health system of the Democratic Republic of the Congo (DRC)

### Aline Mulunda, Achu Lordfred

#### United Nations Population Fund

*BMC Proceedings 2024*, **18(5):**O308

Submission ID #: IMNHC253

**Background**

The shortage of midwives is one of DRC’s major problems in the fight against maternal mortality, which stands at 846/100,000 live births. DRC currently has less than 1 midwife for every 10,000 inhabitants. However, it is important for DRC’s health care system to have adequate supply of well-trained midwives capable of delivering high-quality care. This experience sharing describes the strategy developed by DRC’s government with technical and financial support from UNFPA to increase the number of midwives in the health system.

**Methods**

UNFPA is supporting the midwifery training program in DRC by setting up an 18-month program to retrain qualified nurses as midwives. The use of the retraining pathway to increase the number of midwives targets both nurses already working in hospitals within the health system and nursing teachers at midwifery schools. A pilot midwifery training school has been supported by UNFPA to run the retraining program. The support provided consisted respectively of teacher training, good governance of the school authorities, equipping a technical room, and the setting up a simulation clinic. The first cohort of learners were maternity nurses and nursing teachers from the pilot school. With a pilot school meeting the required standards, the program has been replicated in three provinces: Kasai, Kasai Central, and Sankuru, where 15 nursing teachers have been converted into midwifery teachers at the pilot school. With well-trained midwifery instructors in all three provinces, UNPFA supports the implementation of the retraining program and has trained 45 nurses in two midwifery schools.

**Results**

This strategy has resulted in maternity units having greater availability of midwifery care and an increase in the number of midwifery teachers in the schools.

**Conclusions**

We need to invest more in increasing the number of midwives.

## O309. Rapid analysis of the impact of COVID 19 on access to reproductive health services in Mali

### Sidi Mohamed Ben Moulaye Idriss, Aminata Cisse, Ousmane Sylla, Brahima Kone, Ntji Keita, Seydou Diallo, Aïssata Sangare, Nana Kadia Coulibaly, Amadou Baba Diarra

#### Office National de la Santé de la Reproduction, Mali

*BMC Proceedings 2024*, **18(5):**O309

Submission ID #: IMNHC291

**Background**

Mali is faced with several challenges but has been able to record significant progress. Between Demographic and Health Survey HS M V and DHS M VI, the maternal mortality ratio fell from 364 to 325 per 100,000 live births, and the neonatal mortality rate fell from 34 to 33 per 1,000. Nevertheless, it is feared that all these efforts will be jeopardized by the advent of COVID-19, one of the biggest challenges of the health care system today. This study analyzed the impact of COVID-19 on the use of reproductive health services, with a view to guiding interventions to ensure service continuity.

**Methods**

A mixed-methods study took place in six regions and the district of Bamako. Six tools were used for data collection. Field data collection was carried out from February 1–15, 2021. Excel and SPSS were used to process qualitative data, while QDA_ MINER was used for qualitative data.

**Results**

The postnatal care (PNC) 1 rate in the third quarter (Q) 2019 and 2020 in control areas shows an increase from 72% to 78%, while a decrease from 75% to 72% is observed in study areas. The same trend is confirmed when we consider the two rates in 2019 and 2020 from 82% to 75% in the study areas. Assisted deliveries also saw a decline between Q2 2019 and Q2 2020 in control areas (52% to 47%) and in the study area (79% to 73%), but no major changes were recorded in Q3 2019 compared with Q3 2020 in both areas (45.9% in 2019 and 45% in 2020 in control zones and 76.5%–74.3% in areas zones).

**Conclusions**

The study revealed a negative impact of the pandemic on the use of reproductive health services through certain key indicators. However, the rate of change varies from one health facility to another.

## O310. Overcoming socio-cultural and gender barriers to improve access to RMNCAH services in three regions of Burkina Faso

### Francine Ouedraogo

#### Jhpiego

*BMC Proceedings 2024*, **18(5):**O310

Submission ID #: IMNHC832

**Background**

The IHS (Integrated Health Services) project is implemented by a consortium of nongovernmental organizations (NGOs) with Jhpiego as lead and covers three regions of Burkina Faso. Following the observation that the low level of many health indicators in Burkina Faso could be due to gender-related barriers, a socio-cultural and gender study was carried out by the IHS project. The results of this study will be used to develop intervention strategies based on a sensitive and transformative gender approach. This approach targets both the community and health care providers in the project’s three intervention zones.

**Methods**

Qualitative data collection through focus groups, individual interviews, and a literature review: The study targeted health service providers and clients, community and religious authorities, and associations/NGOs). Twelve experienced interviewers collected data from October 10 to 30, 2022, in the regions covered by the project. Six provinces and 20 communes, including 8 urban and 12 rural, were involved. Qualitative data were analyzed using a systemic approach with the help of NVIVO 12, a qualitative data analysis software package.

**Results**

A restrictive perception and understanding of sexual and reproductive health by the populations interviewed in the three regions: Gender mainstreaming in sexual and reproductive health means that men have the final say in health decisions for their families. Most women either lack information or neglect health issues. The late use of prenatal health services is the result of certain cultural myths. The negative influence of certain religions on the adoption of modern family planning methods by women rather than men. The poor reception and stigmatizing behavior by some health care providers limits adolescents’ access to sexual or reproductive health services adapted to the needs of young people.

**Conclusions**

Gender norms and socio-cultural barriers affect people’s understanding of, access to, and use of reproductive, maternal, newborn, child, and adolescent health services.

## O311. Improving supportive care during childbirth: a qualitative study in four Benin Hospitals

### Thérèse Delvaux^1^, Armelle VIGAN^2^, Jean-Paul Dossou^2^, Christelle Boyi Hounsou^2^

#### ^1^Institute of Tropical Medicine; ^2^Centre de Recherche en Reproduction Humaine et en Démographie

*BMC Proceedings 2024*, **18(5):**O311

Submission ID #: IMNHC1649

**Background**

In 2017, maternal mortality in Benin was 391 per 100,000 live births while neonatal mortality was 30 per 1,000 live births. The quality of care and monitoring of mothers and newborns during childbirth is crucial to reducing these deaths. Our study aimed to identify problems in the care provided between the onset of labor and immediately after delivery, to suggest levers for action to improve the quality of care and reduce maternal and neonatal mortality in Benin hospitals.

**Methods**

We conducted a qualitative study in four hospitals in Benin. Data were collected from health care providers, women in childbirth, birth attendants and caregivers through 63 in-depth interviews, eight observations, and four focus groups. Participants were selected on the basis of reasoned choice. A thematic content analysis was carried out using the World Health Organization’s guide to the organization of childbirth health care as a theoretical framework.

**Results**

The majority of women are ineffectively accompanied during labor. On the one hand, their caregivers provide little of the recommended support, such as breathing or relaxation exercises during contractions. They sometimes indulge in potentially dangerous practices, such as providing the woman with pharmacopoeia products (herbal teas, roots, etc.) to speed up labor. These practices are linked to a lack of knowledge of actually support the woman, but also to the fear of cesarean section, high expenses, and sometimes to the lack of “user- friendly” hospital environment. On the other hand, vital parameters (contractions, fetal heart rate) are not systematically monitored by health care workers. This is due to the high workload, lack of equipment, and sub-optimal organization.

**Conclusions**

The current context of childbirth in the hospitals studied does not always guarantee favorable maternal and fetal outcomes. Challenges are encountered by families and providers alike. Based on these results, simple interventions (teaching families to better support women during childbirth) can help reduce maternal and neonatal mortality in Benin.

## O312. Integrating maternal, neonatal and child care, nutrition, and family planning in francophone West Africa increases resilience to COVID-19 and universal coverage by seizing opportunities to offer services

### Marguerite Ndour^1^, Katelyn Bryant-Comstock^1^, Robert Bambara^1^, Valerie Marcella Zombre Sanon^2^, Issoufou Harou^3^, Gnou Tanoh^4^, Isabelle Bicaba^1^

#### ^1^IntraHealth International; ^2^Ministere de la sante et de l’hygiene publique, Burkina Faso; ^3^Niger Ministry of Health; ^4^Ministere de la sante et de l’hygiene publique, Cote D’Ivoire

*BMC Proceedings 2024*, **18(5):**O312

Submission ID #: IMNHC1302

**Background**

Most West African countries have made it part of their policy to provide integrated, person-centered primary health care to meet the challenges of maternal, neonatal, and infant mortality, as recommended by the World Health Organization. As of 2019, the INSPiRE Initiative intends to seize opportunities to offer services in the crucial prenatal and postnatal period by further engaging countries to make this an operational strategic priority.

**Methods**

An integration approach being implemented at 11 sites in Burkina Faso, Côte d’Ivoire, and Niger includes the following: development by a national technical group of an operational reference framework and indicators for integrating services into the health information system and reorganization of service provision (time/space). It also includes skills building for the systematic provision of a package comprising postpartum family planning (PPFP); maternal, neonatal and infant care, and nutrition services at all points of contact in the mother-child care continuum (prenatal care [PNC]); delivery and postnatal care; and healthy infant consultation/vaccination, applying the new WHO guidelines on PPFP. Data were collected through DHIS2 and analyzed comparing pre (2018), post (2019) and trends observed from 2020 to 2021.

**Results**

There has been an increase in the use of health services at all 11 sites: the number of monthly PNC visits has increased by 188% from 2018 to 2019 (182 to 525), then by 57.3% from 2020 to 2021 within the COVID-19 context; the number of healthy growth monitoring visits for newborns has increased by 329% (660 to 2,173, then 2,595). This commitment of mothers has helped to reduce the proportion of infants showing signs of severe acute malnutrition from 25% to 8% between 2018 and 2019, and increase the PPFP adoption rate from 29.7% in 2018 to 60% in 2021.

**Conclusions**

This concerted approach, which integrates services at all stages of mother and child care will definitely help in achieving the goals of universal health coverage and equitable access to health services. It also serves as a basis for accountability and institutionalization to better seize missed opportunities.

## O313. Improving newborn survival by reducing the transmission of health care-associated infections in the neonatology Department at the University Hospital in Haiti

### Monide Thamar Vital Julmiste, Rose Myriam Beauvil, Meredith Casella Jean-Baptiste

#### Partners In Health

*BMC Proceedings 2024*, **18(5):**O313

Submission ID #: IMNHC442

**Background**

According to UNICEF data, the neonatal mortality rate in Haiti is 32 deaths per 1,000 live births. At the University Hospital (HUM), the neonatal intensive care unit (NICU) had an average death rate of 36% per month between September 2021 and January 2022. Death from septic shock accounted for around 31% of NICU admissions. The most common cause of death from septic shock was health care-associated infections. According to a literature review, the risk of hospital-acquired infections is much higher in low-income countries, increasing the risk of death from sepsis. This study of a quality improvement project aimed to reduce the rate of newborn deaths due to health care-associated infections and present the lessons learned during implementation.

**Methods**

An interprofessional quality improvement team was created in January 2022. The priority matrix was used to identify major problems following a cause-of-death analysis using an Ishikawa diagram. The most important problems were addressed using PDSA (Plan-Do-Study-Act) cycles. Ideas for change were proposed and tested, including the establishment of a cleaning roster for the department, the installation of a unique vital signs machine for each bed, and systematic blood culture training for staff.

**Results**

*Klebsiella pneumoniae, Staphylococcus aureus,* and *Escherichia coli* were identified as the causative agents that caused a peak of 58% deaths among NICU admissions between September and October 2021. The (median) percentage of deaths was significantly reduced to 25% after 4 to 10 months of implementation. Nevertheless, we have noted an increase in deaths due to other health care-associated infections. Other germs including *Burkholderia cepacia; Streptophomonas maltophilia,* and *Staphyloccus epidermidus* have been isolated as causative agents.

**Conclusions**

The use of team-based quality improvement strategies has yielded results in the face of our major problem of neonatal deaths due to health care-associated infections in the NICU at the HUM. More specifically, interventions were related to hygiene, infection prevention, and compliance with infection prevention standards.

## O314. Using cell phones to promote favorable maternal and child health behaviors and practices in a context of integrated health service delivery in Burkina Faso

### Francine Ouedraogo^1^, Issa Ouedraogo^1^, Mathurin Bonzi^1^, Blami Dao^1^, Rita Sylvie Kabore^2^

#### ^1^Jhpiego; ^2^Viamo

*BMC Proceedings 2024*, **18(5):**O314

Submission ID #: IMNHC819

**Background**

The Integrated Health Services (IHS) project supports the government of Burkina Faso to significantly reduce malaria mortality and morbidity and improve the health and nutrition of women, mothers, newborns, children, and adolescents. Implemented in 585 health facilities, IHS is increasing access, equity, and the utilization of quality integrated health services. One of the project’s objectives is to strengthen high-impact social and behavior change interventions with innovative approaches.

**Methods**

The IHS project is being implemented in the Centre-Ouest, Centre-Est, and Sud-Ouest regions from August 2021 to August 2026 by a consortium led by Jhpiego. Activities include capacity building and the development of innovative communication approaches. A toll-free number 321 has been set up with pre-recorded voice messages in local languages on malaria, prenatal care, newborn monitoring, and vaccination. It is accessible to subscribers of the Orange telephone system. Data on caller demographics and message relevance are recorded with their consent and in accordance with current legislation. Information about the 321 toll-free service is disseminated by radio stations and project stakeholders.

**Results**

From June to July 2022, 40,409 listeners accessed the content. Malaria remains the most listened-to topic, winning the attention of over 80% of listeners. This is because malaria is the main reason for consultations, hospitalization, and death. Those most vulnerable to the disease are pregnant women and children under 5. The high level of awareness of malaria messages helps to reinforce knowledge and improve practices in disease prevention. The most frequently heard malaria messages concern prompt treatment, availability, consultations, and use of mosquito nets.

**Conclusions**

Within two months, the 321 toll-free service recorded 40,409 calls. The number of people listening to messages about malaria confirms the need for information about how to manage the disease.

## O315. Analysis of cesarean section by Robson classification in the gynecology and obstetrics department of Allada Zone Hospital in Benin from 2018 to 2020

### Tchimon Yea Setchegnon Vodouhe

#### College National Des Gynecologues Obstetriciens Du Benin

*BMC Proceedings 2024*, **18(5):**O315

Submission ID #: IMNHC523

**Background**

Medically justified cesarean section is an important means of reducing maternal and perinatal mortality and morbidity. Robson’s classification enables us to objectively analyze our practice and compare it with that of other health facilities. The aim of the study was to assess the practice of cesarean section in our department using Robson’s classification.

**Methods**

Retrospective descriptive study, from August 24, 2018, to December 31, 2020, (28 months) in the gynecology and obstetrics department of Allada Zone Hospital in Benin. The study focused on the records of pregnant women who delivered by cesarean section during the study period. Systematic sampling was carried out.

**Results**

The cesarean section rate was 41.5%. The average age of the patients was 26, and 24.5% had at least one previous cesarean section. The average parity was 3.1. Most were referrals (76.9%). Operated patients mainly belonged to groups 5 (18.4%), 1 (18.1%) and 3 (14.9%). In group 5, 56.5% of cesarean sections were performed during labor. In most cases, cesarean sections were performed as emergencies (90.3%), with the main indications being fetal asphyxia (24.4%), severe pre-eclampsia and eclampsia (15.7%), and multicatric uterus (11.4%).

**Conclusions**

The cesarean section rate was high. The procedure was mainly performed on full-term pregnant women with a scarred uterus. Robson’s classification, coupled with a study of cesarean section indications, enables us to perceive how much scarred uteri and hypertensive complications determine the delivery route adopted in the department.

## O316. Implementing a collaboration for improving the quality of maternal care amidst the COVID-19 pandemic – lessons from the Alcançar project in Mozambique

### Jacqueline Torres^1^, Maureen Tshabalala^1^, Fernando Faraco^1^, Daniela Feitosa^1^, Kelly Rosenfeld^1^, Sodzi Sodzi-Tettey^1^, Geoffrey Ezepue^2^

#### ^1^Institute for Healthcare Improvement; ^2^FHI 360

*BMC Proceedings 2024*, **18(5):**O316

Submission ID #: IMNHC665

**Background**

Alcançar is a U.S. Agency for International Development-funded initiative to reduce maternal and newborn mortality in Mozambique. The initiative adapted the Institute for Healthcare Improvement’s Quality Improvement Collaborative (QIC) model. This study describes the first phase of the QIC implementation.

**Methods**

The QIC method includes measurable goals, improvement indicators, theory of change, and rapid testing, using PDSA (Plan, Do, Study, Act). QIC started in April 2020 and was affected by COVID-19. The theory of change was revised, focusing on a simplified care package for the prevention and management of postpartum hemorrhage, puerperal sepsis, and preeclampsia. The goal is to reduce the institutional maternal mortality ratio by 30% by March 2024. The strategy started with 35 health units (HU), 14 hospitals, and 28 peripheral units, the goal is to reach 120 HU by 2024.

**Results**

One hundred health workers were trained on QIC. Seven leaders, one per district, were selected as a reference and received manuals, checklists, and simulation videos for technical assistance to the teams. We adopted monthly virtual learning sessions in response to COVID-19, to offer low-dose high frequency sessions, and four, monthly hybrid learning sessions. We also used a gender approach, realistic simulation for managing complications, an electronic partogram, and cell phone messages for health education. The adherence to simplified care package is measured monthly through process indicators. The project outcome indicators are taken from the national database and showed a 62% reduction in maternal mortality ratio from 124 per 100,000 live births in the baseline period (May–October 2019) to 47 per 100,000 (September 2020–May 2022) in the 35 HU included in the collaborative.

**Conclusions**

The 35 HUs routinely use the QIC Model, which includes an active quality improvement team, quality improvement meetings for the review of indicators, root cause analysis of identified problems, planning new theories of change, completion of monthly reports, and sharing of knowledge with other HU in the district. This routine, together with the other actions of the Alcançar project, may be associated with the observed reduction in MMR.

## O317. Using MEWOS and care packages to reduce maternal mortality – a success story from the USAID-funded Alcançar project in Mozambique

### Jacqueline Torres^1^, Maureen Tshabalala^1^, Fernando Faraco^1^, Daniela Feitosa^1^, Kelly Rosenfeld^1^, Sodzi Sodzi-Tettey^1^, Geoffrey Ezepue^2^

#### ^1^Institute for Healthcare Improvement; ^2^FHI 360

*BMC Proceedings 2024*, **18(5):**O317

Submission ID #: IMNHC678

**Background**

The Modified Early Warning Score (MEWOS) is for obstetric patients and is based on the monitoring of vital signs. The care package, or bundle, consists in a set of up to five evidence-based interventions that should be applied to 100% of patients in a defined population. Together, they ensure the timely treatment of established or impending critical conditions. We report a success story of adherence to MEWOS and four obstetric bundles (OB): 1) prevention and 2) management of postpartum hemorrhage; 3) puerperal sepsis; and 4) preeclampsia, in two health facilities in Memba, Mozambique.

**Methods**

Memba is a rural district with 367,242 inhabitants and 14 health facilities. The lead doctor was trained as an Improvement Advisor by the Alcançar project and volunteered to test the use of MEWOS and OBs at the Memba Health Center (CSM) and the district hospital. The CSM has 11 obstetric beds, seven mid-level maternal and child health (MCH) nurses, two general practitioners and carries out about 90 deliveries per month. The district hospital has 15 obstetric beds, 10 MCH nurses, and two general practitioners and carries out 80 deliveries per month, on average. The Memba’s Improvement Advisor trained the CSM and district hospital nurses in the use of MEWOS OBs and initially tested the use of the instrument in a single shift at the district hospital, applying it to a patient with hypertension. With the test’s success, the team gradually expanded its use to the entire service and began to monitor the registration of MEWOS in medical records, with good adherence.

**Results**

The team emphasizes that they no longer wait for the case to worsen before taking action. They report three cases of hemorrhage, one case of pre-eclampsia, and one case of sepsis that they were able to rescue after starting MEWOS and OBs before the patients worsened. MCH nurses show good adherence and satisfaction with the tools. They improved the continuous monitoring of critically ill patients, the recording of vital signs in medical records, and the appropriate identification and treatment of complications.

**Conclusion**

The use of MEWOS and OBs proved to be a simple, cheap, easy to understand, and highly effective strategy.

## O318. Factors contributing to the increase in maternal and perinatal mortality during the COVID-19 pandemic in Latin America

### Evelina Chapman^1^, Virginia Camacho^2^, Silvina Ramos^1^, Jim Ricca^3^, Mariana Romero^1^, Guido Sciurano^1^

#### ^1^Centro de Estudio de Estado y Sociedad; ^2^United Nations Population Fund; ^3^Jhpiego

*BMC Proceedings 2024*, **18(5):**O318

Submission ID #: IMNHC1681

**Background**

In 2020, COVID-19 infection in pregnant women was linked to higher mortality ratios, especially in women suffering from comorbidities. Maternal deaths also increased as a result of the disruption in the supply and underutilization of health services due to reductions in access and quality. A multi-country study was conducted in Chile, Colombia, and Ecuador to investigate the determinants of maternal mortality and its relationship with policies in the period between March 2020 and July 2021.

**Methods**

Qualitative techniques were applied to analyze information from all policy documents issued by the highest health authority in each country that could have affected the provision of maternal, sexual, and reproductive health services (*n*=62); interviews with decision-makers (*n*=21) and health care providers (*n*=30); and interviews with family members of women who died of maternal causes during the period analyzed (*n*=28). The data collected were coded according to dimensions chosen from the SURE (Supporting Use of Research Evidence) model for policy implementation analysis. Information was subsequently sorted according to the effects on access to and use of health services, according to the Three Delays model.

**Results**

The analysis showed that vulnerability due to socioeconomic status combined with the lack of policy implementation elements designed for pregnant women. The effectiveness of measures taken, such as telemedicine and home visits, was limited. The decentralization and fragmentation of health management, as well as problems in communicating measures to society and health teams, hindered the provision of maternal health and sexual and reproductive health services. The disruption of primary health care and the pregnant women’s fear of attending health centers had a negative effect on the first delay. The analysis identified deficits regarding care for health personnel and the use of evidence.

**Conclusions**

Different conditions affected the availability, use, and quality of maternal, reproductive, and perinatal health services. Access to timely and quality care was the most affected.

## O319. Quality improvement project: increasing comprehensive screening for vulnerable pregnant women in their first contact with health services in Chiapas, Mexico

### Nastassia Donoho, Zeus Antonio Aranda Remon

#### Partners In Health

*BMC Proceedings 2024*, **18(5):**O319

Submission ID #: IMNHC1069

**Background**

Compañeros en Salud (CES) is an organization dedicated to the delivery of free health services to vulnerable populations in Chiapas, Mexico. Prenatal care is offered in 10 clinics that attend to more than 400 pregnant women annually. Since complementary exams and specialized care services are difficult to access, CES offers a set of screenings based on rapid diagnostic tests that, if performed at the first contact with the pregnant woman, allow for a timely determination of risk. For cases requiring specialized services, these tests offer the best possible timeframe for the organization to provide social support and obtain the best results for pregnant women.

**Methods**

We performed a retrospective analysis regarding the percentage of pregnant women who received a complete screening during their first contact with health services. We analyzed data from 16 months prior to the start of the analysis, obtaining a median of 20%. The health staff identified three areas of work to improve results: 1) reducing the workload by adding a new member in three clinics; 2) improving the availability of supplies through a change in the purchasing model; and 3) improving the health provider user experience and data collection by addressing deficiencies in the electronic medical record system.

**Results**

In the months following implementation, two improvements were noted in the supply of inputs to the care services. In terms of complete screening, an improvement was observed. When analyzing only the services that included staff, in addition to an improvement, we observed data above the median sustained for 10 months. In these clinics, the median screening rate increased by 37%. After these changes, the user experience of electronic medical records was reported to be was significantly better.

**Conclusions**

It is vital to carry out objective and time-based analyses of interventions. We hope to share the results with health staff and continue to implement their proposals for improvement of our interventions, which have been reflected in the results.

## O320. Training and Incorporation of University Midwifery Assistants into the Guatemalan health system as a strategy to reduce maternal and neonatal mortality

### Hector Romeo Menendez^1^, Dora López^2^, Jose Roberto Molina Barrera^3^, Maggie Fischer^2^, Miguel Marroquín^2^

#### ^1^United States Agency for International Development; ^2^Jhpiego; ^3^Programa Nacional de Salud Reproductiva, Guatemala

*BMC Proceedings 2024*, **18(5):**O320

Submission ID #: IMNHC407

**Background**

According to the National Plan for the Reduction of Maternal and Neonatal Mortality 2021–2025, Guatemala has a strategy for training university midwifery assistants (TUPs, from its Spanish acronym), a human resource qualified to provide maternal and neonatal care. The strategy started in 2018 with the formal recognition by the Ministry of Public Health and Social Assistance (MSPAS) and in partnership with the Da Vinci Universities of Guatemala and San Martin de Porres of Lima, Peru, and with the support of the Health and Nutrition Project funded by the U.S. Agency for International Development.

**Methods**

MSPAS facilitated the training of TUPs from communities with the highest maternal and neonatal mortality indicators in Guatemala. The training takes three years and is based on global midwifery curriculum recommendations. The first group of 32 TUPs graduated in 2021 and were integrated into second-level health services in eight districts and one district hospital for a period of six months. During this time, the health management information system reported an increase in prenatal care and delivery services.

**Results**

In December 2021, after six months of integration of the TUPs, a significant increase in comprehensive and integrated maternal and neonatal care was seen: prenatal care increased from 79% to 96% and childbirth care in a health service from 39% to 64%.

**Conclusions**

TUPs have demonstrated their impact on quality of care, increase in institutional childbirth, offer of contraceptive methods, culturally relevant care, and respectful treatment.

