# Correction: Proceedings of the 2023 International Maternal and Newborn Health Conference

**DOI:** 10.1186/s12919-024-00293-2

**Published:** 2024-04-17

**Correction: BMC Proc 18 (Suppl 5), 6 (2024)**

**https://doi.org/10.1186/s12919-024-00289-y**

Following publication of the original article [1], the authors reported that abstract F8.2 author names were erroneously omitted. The authors and affiliations are given below and the original article [1] has been corrected.

Gloria Mutimbwa Siseho^1^, Thubelihle Mathole^1^, and Debra Jackson^1,2^

^1^ University of the Western Cape, South Africa, ^2^London School of Hygiene and Tropical Medicine, London
