# Correction: Proceedings of the 2023 International Maternal and Newborn Health Conference

**DOI:** 10.1186/s12919-024-00296-z

**Published:** 2024-07-01

**Correction: BMC Proc 18 (Suppl 5), 6 (2024)**

**https://doi.org/10.1186/s12919-024-00289-y**

Following publication of the original article [1], it was reported that abstract F30.1 has an error; the International Federation for Spina Bifida and Hydrocephalus should not have been included within the abstract.

The incorrect abstract is:

Submission ID #: IMNHC1051

Panel ID# (if applicable): DYRON901051

**Panel Description**

Birth defects are one of the leading causes of stillbirths and child mortality globally. There is a global inequity in birth defects prevention through proven public health interventions such as staple food fortification with folic acid, an essential micronutrient. We present the epidemiology of neural tube birth defects that are largely preventable, and the method of preventing them through food fortification. Nutrition International and Food Fortification Initiative will present their work in achieving global prevention of neural tube defects. We will also present a story of advocacy, featuring the Global Alliance for Prevention of Folic Acid-Preventable Spina Bifida (GAPSBiF) and the International Federation for Spina Bifida and Hydrocephalus. These are two global organizations that are leading efforts on prevention through active advocacy. All the talks in this panel will cover the importance of primary prevention of major birth defects thus improving child health, especially in low- and middle-income countries.

The correct abstract is:

Submission ID #: IMNHC1051.

Panel ID# (if applicable): DYRON901051.

**Panel Description**

Birth defects are one of the leading causes of stillbirths and child mortality globally. There is a global inequity in birth defects prevention through proven public health interventions such as staple food fortification with folic acid, an essential micronutrient. We present the epidemiology of neural tube birth defects that are largely preventable, and the method of preventing them through food fortification. Nutrition International and Food Fortification Initiative will present their work in achieving global prevention of neural tube defects. We will also present a story of advocacy, featuring the Global Alliance for Prevention of Folic Acid-Preventable Spina Bifida (GAPSBiF). These are two global organizations that are leading efforts on prevention through active advocacy. All the talks in this panel will cover the importance of primary prevention of major birth defects thus improving child health, especially in low- and middle-income countries.

The original article [1] has been updated.
